# Supplementary material for: Redox regulation of PTPN22 affects the severity of T-cell-dependent autoimmune inflammation
Source: eLife. 2022 May 19;11:e74549. doi: 10.7554/eLife.74549 (PMC9119677; doi:10.7554/eLife.74549)
Supplement: Figure 1—source data 1. [file elife-74549-fig1-data1.docx]

**Raw data(triplicates) 1000nM WT& C129S PTPN22**

**Raw data(triplicates) 1000nM WT PTPN22**

**0.20**

**0.15**

**0.10**

**A405**

**0.05**

**0.00**


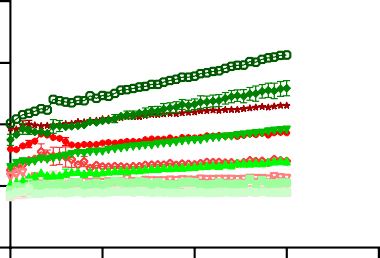


**0 5 10 15 20**

**Time(min)**

WT 0

WT 0.25mM WT 1mM WT 2.5mM WT 5mM WT 7.5mM MUT 0

MUT 0.25mM MUT 1mM MUT 2.5mM MUT 5mM MUT 7.5mM

**0.20**

**0.15**

**0.10**

**A405**

**0.05**

**0.00**


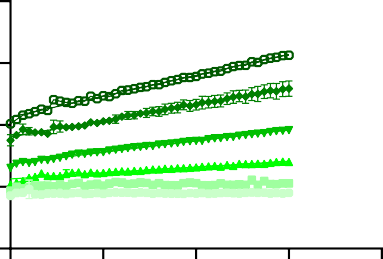


**0 5 10 15 20**

**Time(min)**

WT 0

WT 0.25mM WT 1mM WT 2.5mM WT 5mM WT 7.5mM

**Raw data(triplicates) 1000nM C129S PTPN22**

**Raw data(triplicates) 1000nM C129S PTPN22**

**0.15**

**0.10**

**A405**

**0.05**

**0.00**


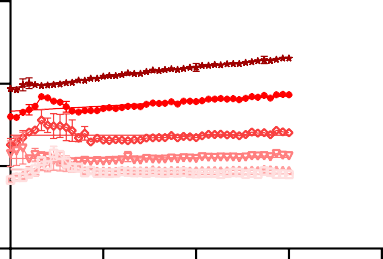


**0 5 10 15 20**

**Time(min)**

MUT 0

MUT 0.25mM MUT 1mM MUT 2.5mM MUT 5mM MUT 7.5mM

**0.15**

**0.10**

**A405**

**0.05**

**0.00**


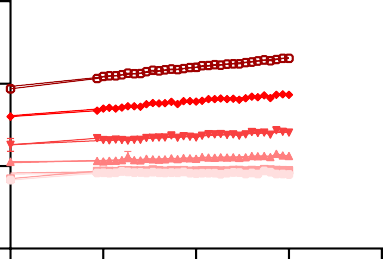


**0 5 10 15 20**

**Time(min)**

MUT 0

MUT 0.25mM MUT 1mM MUT 2.5mM MUT 5mM MUT 7.5mM

**WT**

**0.004**


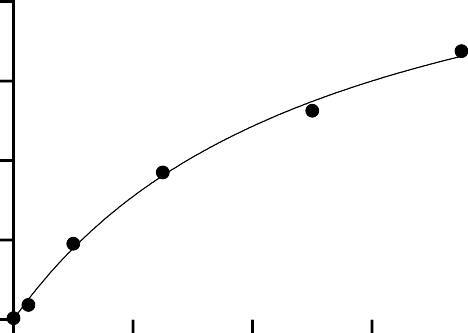


Km=5.311

**0.0015**

**C129S MUTANT**

**0.003**

**0.0010**

**slop**

**0.002**

**slop**

**0.001**

**0.0005**

**0.000**

**0 2 4 6 8**

**mM(p-NPP)**

**0.0000**

**0 2 4 6 8**


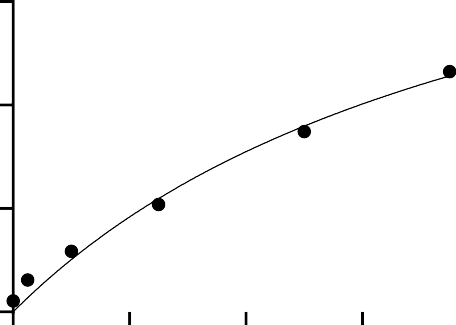


Km=8.826

**mM(p-NPP)**

Measurement Wavelength 405 nm

Bandwidth 9 nm

Number of Flashes 5

Settle Time 0 ms

Part of Plate F1-H12

Start Time: 2020 09-29 15:22:09

Cycle Nr.

1

2

3

4

5

6

7

8

9

10 11 12 13 14 15 16 17 18 19 20 21 22 23 24 25 26 27 28 29 30 31 32 33 34 35 36 37 38 39 40 41 42 43 44 45 46

|  |  |  |  |  |  |  |  |  |  |  |  |  |  |  |  |  |  |  |  |  |  |  |  |  |  |  |  |  |  |  |  |  |  |  |  |  |  |  |  |  |  |  |  |  |  |  |
| --- | --- | --- | --- | --- | --- | --- | --- | --- | --- | --- | --- | --- | --- | --- | --- | --- | --- | --- | --- | --- | --- | --- | --- | --- | --- | --- | --- | --- | --- | --- | --- | --- | --- | --- | --- | --- | --- | --- | --- | --- | --- | --- | --- | --- | --- | --- |
| Time [s] | 0 | 20 | 40 | 60 | 80 | 100 | 120 | 140 | 160 | 180 | 200 | 220 | 240 | 260 | 280 | 300 | 320 | 340 | 360 | 380 | 400 | 420 | 440 | 460 | 480 | 500 | 520 | 540 | 560 | 580 | 600 | 620 | 640 | 660 | 680 | 700 | 720 | 740 | 760 | 780 | 800 | 820 | 840 | 860 | 880 | 900 |
| Temp. [°C] | 27,4 | 27,6 | 27,7 | 27,6 | 27,6 | 27,9 | 27,8 | 27,7 | 27,6 | 27,9 | 27,9 | 28 | 27,9 | 27,9 | 27,6 | 28 | 27,7 | 27,9 | 27,8 | 27,9 | 27,8 | 28 | 28,1 | 27,8 | 27,9 | 28,1 | 27,8 | 27,9 | 27,8 | 28 | 27,9 | 28 | 27,8 | 28,1 | 28 | 27,9 | 27,9 | 28,1 | 27,9 | 28 | 28,1 | 28,1 | 28,2 | 28,2 | 27,9 | 28,3 |
| F1 | 0,0427 | 0,0435 | 0,0437 | 0,0436 | 0,0436 | 0,0438 | 0,0437 | 0,0441 | 0,0443 | 0,0438 | 0,0441 | 0,044 | 0,0441 | 0,0441 | 0,0443 | 0,0442 | 0,0442 | 0,0443 | 0,0442 | 0,0445 | 0,0442 | 0,0443 | 0,0444 | 0,0445 | 0,0447 | 0,0441 | 0,0443 | 0,0445 | 0,0441 | 0,0447 | 0,0444 | 0,0447 | 0,0443 | 0,0445 | 0,0443 | 0,0442 | 0,0443 | 0,0443 | 0,0447 | 0,0445 | 0,0447 | 0,0448 | 0,0446 | 0,0444 | 0,0444 | 0,0448 |
| F2 | 0,0427 | 0,0442 | 0,0444 | 0,0566 | 0,044 | 0,0437 | 0,0452 | 0,0442 | 0,0445 | 0,044 | 0,0446 | 0,0445 | 0,0443 | 0,0443 | 0,0445 | 0,0443 | 0,0449 | 0,0449 | 0,0459 | 0,0455 | 0,0452 | 0,0454 | 0,0455 | 0,0443 | 0,045 | 0,0454 | 0,0444 | 0,0445 | 0,0441 | 0,0447 | 0,0444 | 0,0449 | 0,0443 | 0,0444 | 0,0444 | 0,0456 | 0,0452 | 0,0444 | 0,0459 | 0,0449 | 0,0453 | 0,0448 | 0,0446 | 0,0449 | 0,0446 | 0,045 |
| F3 | 0,0429 | 0,0472 | 0,0475 | 0,0434 | 0,0442 | 0,0431 | 0,0447 | 0,0456 | 0,045 | 0,0443 | 0,0456 | 0,0467 | 0,0439 | 0,0449 | 0,0459 | 0,0443 | 0,046 | 0,0469 | 0,0455 | 0,0457 | 0,0449 | 0,0466 | 0,0456 | 0,0442 | 0,0451 | 0,0442 | 0,0447 | 0,0444 | 0,0445 | 0,0451 | 0,0449 | 0,045 | 0,0443 | 0,0448 | 0,0459 | 0,0454 | 0,0448 | 0,0448 | 0,0448 | 0,0467 | 0,045 | 0,0462 | 0,0448 | 0,0452 | 0,0449 | 0,0454 |
| F4 | 0,0478 | 0,0465 | 0,0465 | 0,0491 | 0,05 | 0,0476 | 0,0496 | 0,0491 | 0,05 | 0,0499 | 0,051 | 0,0531 | 0,0496 | 0,0519 | 0,0492 | 0,0489 | 0,0532 | 0,0542 | 0,0537 | 0,0512 | 0,0531 | 0,0534 | 0,0527 | 0,0497 | 0,0534 | 0,0498 | 0,0509 | 0,0496 | 0,0533 | 0,0537 | 0,0525 | 0,0505 | 0,0504 | 0,0505 | 0,0532 | 0,0517 | 0,0513 | 0,051 | 0,0511 | 0,0553 | 0,051 | 0,0537 | 0,0513 | 0,0513 | 0,0518 | 0,0521 |
| F5 | 0,047 | 0,0523 | 0,0477 | 0,0493 | 0,0492 | 0,0491 | 0,0515 | 0,0507 | 0,0533 | 0,049 | 0,0513 | 0,0546 | 0,0508 | 0,0536 | 0,0557 | 0,0536 | 0,0541 | 0,0547 | 0,0554 | 0,0551 | 0,0523 | 0,0565 | 0,0547 | 0,0539 | 0,0547 | 0,0531 | 0,052 | 0,0524 | 0,0542 | 0,0553 | 0,0553 | 0,0511 | 0,0513 | 0,052 | 0,0536 | 0,0515 | 0,052 | 0,0521 | 0,0514 | 0,0586 | 0,0517 | 0,058 | 0,0531 | 0,0525 | 0,0527 | 0,0537 |
| F6 | 0,0454 | 0,0505 | 0,0517 | 0,0504 | 0,0535 | 0,0543 | 0,0515 | 0,0512 | 0,0523 | 0,0524 | 0,0551 | 0,0517 | 0,0516 | 0,0497 | 0,0513 | 0,0501 | 0,0506 | 0,0528 | 0,0512 | 0,0504 | 0,0523 | 0,0502 | 0,052 | 0,0512 | 0,0508 | 0,0513 | 0,051 | 0,0514 | 0,0512 | 0,051 | 0,0511 | 0,0524 | 0,0514 | 0,0517 | 0,0514 | 0,0518 | 0,0517 | 0,0525 | 0,0521 | 0,0521 | 0,0522 | 0,0522 | 0,0524 | 0,0527 | 0,0537 | 0,0525 |
| F7 | 0,054 | 0,0554 | 0,0577 | 0,06 | 0,0591 | 0,0641 | 0,0617 | 0,0614 | 0,0609 | 0,0642 | 0,0644 | 0,0627 | 0,0619 | 0,0625 | 0,062 | 0,0627 | 0,0636 | 0,0631 | 0,0633 | 0,064 | 0,0641 | 0,0641 | 0,0645 | 0,0654 | 0,0653 | 0,066 | 0,0658 | 0,0662 | 0,0667 | 0,0666 | 0,0669 | 0,068 | 0,0678 | 0,0679 | 0,0676 | 0,0686 | 0,0683 | 0,0699 | 0,0694 | 0,0694 | 0,07 | 0,0697 | 0,0704 | 0,0709 | 0,0717 | 0,0711 |
| F8 | 0,0512 | 0,0524 | 0,0529 | 0,0552 | 0,057 | 0,0601 | 0,0577 | 0,0572 | 0,0574 | 0,0591 | 0,0604 | 0,0611 | 0,0591 | 0,0609 | 0,0596 | 0,0606 | 0,0607 | 0,0607 | 0,0617 | 0,0613 | 0,0617 | 0,0621 | 0,0626 | 0,0633 | 0,0636 | 0,0641 | 0,0639 | 0,0647 | 0,0643 | 0,0644 | 0,0649 | 0,0652 | 0,0658 | 0,0662 | 0,0655 | 0,0668 | 0,066 | 0,0679 | 0,0677 | 0,0675 | 0,0679 | 0,0676 | 0,0687 | 0,0692 | 0,0694 | 0,0691 |
| F9 | 0,0508 | 0,0526 | 0,0531 | 0,0564 | 0,0579 | 0,0586 | 0,0571 | 0,0578 | 0,0579 | 0,0584 | 0,0597 | 0,0613 | 0,0596 | 0,0613 | 0,061 | 0,0606 | 0,0613 | 0,0632 | 0,0628 | 0,0622 | 0,0631 | 0,0623 | 0,0636 | 0,0635 | 0,0636 | 0,0642 | 0,0642 | 0,0644 | 0,0641 | 0,065 | 0,0656 | 0,0655 | 0,0663 | 0,0666 | 0,0661 | 0,0672 | 0,0664 | 0,0679 | 0,068 | 0,0683 | 0,0685 | 0,0685 | 0,0689 | 0,07 | 0,07 | 0,07 |
| F10 | 0,0643 | 0,0707 | 0,0732 | 0,0701 | 0,0702 | 0,072 | 0,0712 | 0,0728 | 0,074 | 0,0741 | 0,0767 | 0,0761 | 0,0775 | 0,0773 | 0,0775 | 0,0779 | 0,0786 | 0,0805 | 0,08 | 0,0803 | 0,0818 | 0,0815 | 0,0829 | 0,0827 | 0,0831 | 0,084 | 0,0845 | 0,0853 | 0,0847 | 0,0862 | 0,0869 | 0,0867 | 0,0882 | 0,0884 | 0,0885 | 0,0896 | 0,0897 | 0,091 | 0,0912 | 0,0913 | 0,0926 | 0,0926 | 0,0936 | 0,0945 | 0,0949 | 0,0953 |
| F11 | 0,0658 | 0,0687 | 0,0695 | 0,0699 | 0,0706 | 0,0718 | 0,0715 | 0,0725 | 0,0731 | 0,0752 | 0,076 | 0,0768 | 0,0768 | 0,0777 | 0,0785 | 0,0785 | 0,0802 | 0,0801 | 0,0812 | 0,0825 | 0,0822 | 0,0834 | 0,0834 | 0,0835 | 0,0851 | 0,0851 | 0,0858 | 0,086 | 0,0869 | 0,0873 | 0,0875 | 0,0878 | 0,089 | 0,09 | 0,0902 | 0,0909 | 0,0911 | 0,0922 | 0,0926 | 0,0935 | 0,094 | 0,0941 | 0,095 | 0,0957 | 0,0964 | 0,097 |
| F12 | 0,0655 | 0,0661 | 0,0673 | 0,0679 | 0,069 | 0,0714 | 0,0717 | 0,0719 | 0,0731 | 0,0746 | 0,0749 | 0,0775 | 0,0757 | 0,0777 | 0,0773 | 0,0772 | 0,0792 | 0,079 | 0,0805 | 0,0808 | 0,0817 | 0,0821 | 0,0824 | 0,0825 | 0,0843 | 0,0841 | 0,0847 | 0,0849 | 0,0869 | 0,0869 | 0,0866 | 0,0865 | 0,0881 | 0,0886 | 0,089 | 0,0899 | 0,0899 | 0,0905 | 0,0913 | 0,0922 | 0,0921 | 0,0931 | 0,094 | 0,0947 | 0,0946 | 0,0952 |
| G1 | 0,0922 | 0,0933 | 0,101 | 0,0955 | 0,0945 | 0,0972 | 0,0953 | 0,1049 | 0,1038 | 0,1013 | 0,101 | 0,1021 | 0,1025 | 0,1051 | 0,1048 | 0,1056 | 0,1067 | 0,1085 | 0,1093 | 0,1113 | 0,1112 | 0,1123 | 0,1139 | 0,1148 | 0,1151 | 0,1173 | 0,1181 | 0,1191 | 0,1206 | 0,1202 | 0,121 | 0,1237 | 0,1236 | 0,1243 | 0,1246 | 0,1265 | 0,1279 | 0,1282 | 0,1289 | 0,1308 | 0,1319 | 0,1326 | 0,1339 | 0,1342 | 0,1353 | 0,1361 |
| G2 | 0,0873 | 0,0885 | 0,0925 | 0,0917 | 0,0926 | 0,0922 | 0,0913 | 0,0947 | 0,0949 | 0,0962 | 0,097 | 0,0967 | 0,0979 | 0,1001 | 0,0994 | 0,1011 | 0,1012 | 0,1023 | 0,1045 | 0,1054 | 0,105 | 0,1069 | 0,1066 | 0,1079 | 0,1075 | 0,1088 | 0,1105 | 0,1104 | 0,1126 | 0,1115 | 0,1125 | 0,1136 | 0,1144 | 0,1146 | 0,1151 | 0,1171 | 0,1177 | 0,1188 | 0,1183 | 0,1206 | 0,1197 | 0,1216 | 0,1223 | 0,1219 | 0,1234 | 0,1237 |
| G3 | 0,0833 | 0,0932 | 0,0958 | 0,0977 | 0,0948 | 0,0926 | 0,0924 | 0,0959 | 0,0976 | 0,0967 | 0,0971 | 0,0978 | 0,0985 | 0,1009 | 0,1004 | 0,1022 | 0,1022 | 0,1034 | 0,1062 | 0,1066 | 0,1074 | 0,1088 | 0,1085 | 0,1099 | 0,11 | 0,1116 | 0,1122 | 0,113 | 0,1153 | 0,1143 | 0,1156 | 0,117 | 0,1174 | 0,1183 | 0,1186 | 0,1204 | 0,1224 | 0,1234 | 0,1217 | 0,1236 | 0,1235 | 0,1268 | 0,1269 | 0,1256 | 0,1282 | 0,1284 |
| G4 | 0,1017 | 0,1062 | 0,1083 | 0,1104 | 0,1122 | 0,1151 | 0,113 | 0,1209 | 0,1204 | 0,1185 | 0,1189 | 0,1206 | 0,1208 | 0,1246 | 0,1224 | 0,1245 | 0,1244 | 0,1267 | 0,1296 | 0,1289 | 0,1303 | 0,1317 | 0,1323 | 0,1334 | 0,1337 | 0,1361 | 0,1372 | 0,1386 | 0,1394 | 0,1399 | 0,1406 | 0,1425 | 0,1429 | 0,1446 | 0,1447 | 0,1467 | 0,1476 | 0,1485 | 0,1494 | 0,1519 | 0,1515 | 0,1535 | 0,1546 | 0,1565 | 0,157 | 0,1574 |
| G5 | 0,1018 | 0,1031 | 0,108 | 0,1082 | 0,1103 | 0,1124 | 0,1122 | 0,1219 | 0,1207 | 0,1189 | 0,1178 | 0,1199 | 0,1192 | 0,1229 | 0,1215 | 0,1241 | 0,1233 | 0,1254 | 0,1276 | 0,129 | 0,1298 | 0,1309 | 0,1313 | 0,1334 | 0,133 | 0,1347 | 0,1363 | 0,1371 | 0,1392 | 0,1389 | 0,1399 | 0,142 | 0,1429 | 0,1439 | 0,1444 | 0,1466 | 0,1485 | 0,149 | 0,1492 | 0,152 | 0,1521 | 0,1538 | 0,1558 | 0,1557 | 0,1572 | 0,1576 |
| G6 | 0,0992 | 0,104 | 0,1076 | 0,1086 | 0,1098 | 0,1108 | 0,1101 | 0,1183 | 0,1169 | 0,1172 | 0,1162 | 0,1182 | 0,1177 | 0,1225 | 0,1198 | 0,1221 | 0,1213 | 0,1234 | 0,1266 | 0,1269 | 0,1274 | 0,1288 | 0,1293 | 0,1311 | 0,1308 | 0,1323 | 0,134 | 0,1345 | 0,1368 | 0,1363 | 0,1375 | 0,1391 | 0,1402 | 0,1408 | 0,1417 | 0,1436 | 0,1455 | 0,1466 | 0,1464 | 0,1493 | 0,1484 | 0,1515 | 0,1521 | 0,152 | 0,154 | 0,1543 |
| G7 | 0,0419 | 0,0427 | 0,0426 | 0,0439 | 0,0483 | 0,0554 | 0,0517 | 0,0621 | 0,0587 | 0,0544 | 0,0513 | 0,0497 | 0,0464 | 0,0474 | 0,0459 | 0,046 | 0,0455 | 0,0458 | 0,0469 | 0,0466 | 0,0459 | 0,0461 | 0,0457 | 0,0464 | 0,0459 | 0,046 | 0,0463 | 0,046 | 0,0465 | 0,0458 | 0,0457 | 0,046 | 0,0458 | 0,0459 | 0,0449 | 0,0457 | 0,0465 | 0,0461 | 0,045 | 0,0457 | 0,0451 | 0,047 | 0,0463 | 0,0448 | 0,0452 | 0,045 |
| G8 | 0,0415 | 0,0454 | 0,044 | 0,0494 | 0,0522 | 0,0537 | 0,0495 | 0,0535 | 0,0537 | 0,0485 | 0,0462 | 0,0475 | 0,0455 | 0,0467 | 0,0459 | 0,0461 | 0,0459 | 0,0462 | 0,0469 | 0,0465 | 0,0463 | 0,0462 | 0,0458 | 0,0461 | 0,0455 | 0,0458 | 0,0458 | 0,0458 | 0,0463 | 0,0452 | 0,0451 | 0,0453 | 0,0455 | 0,0454 | 0,045 | 0,0454 | 0,0456 | 0,0459 | 0,0448 | 0,0453 | 0,0449 | 0,0465 | 0,046 | 0,0451 | 0,0449 | 0,0445 |
| G9 | 0,0414 | 0,0415 | 0,0429 | 0,044 | 0,0442 | 0,0502 | 0,0507 | 0,058 | 0,0581 | 0,0545 | 0,0502 | 0,05 | 0,0464 | 0,0477 | 0,0457 | 0,0457 | 0,0455 | 0,0457 | 0,0468 | 0,0463 | 0,046 | 0,0462 | 0,0461 | 0,0466 | 0,0463 | 0,0458 | 0,046 | 0,0462 | 0,0467 | 0,046 | 0,0455 | 0,0458 | 0,0456 | 0,0458 | 0,0452 | 0,0458 | 0,0464 | 0,0462 | 0,0457 | 0,0464 | 0,0457 | 0,0477 | 0,0465 | 0,0455 | 0,0453 | 0,0453 |
| G10 | 0,0427 | 0,0431 | 0,0447 | 0,0451 | 0,0467 | 0,0514 | 0,052 | 0,0602 | 0,0567 | 0,0558 | 0,0517 | 0,0515 | 0,0483 | 0,0488 | 0,0475 | 0,0478 | 0,0476 | 0,0475 | 0,0484 | 0,0481 | 0,048 | 0,0481 | 0,0479 | 0,0478 | 0,048 | 0,0474 | 0,0478 | 0,0476 | 0,048 | 0,0473 | 0,0476 | 0,0476 | 0,0477 | 0,0476 | 0,0474 | 0,0476 | 0,0481 | 0,048 | 0,0476 | 0,048 | 0,0478 | 0,0486 | 0,0481 | 0,0479 | 0,0478 | 0,0475 |
| G11 | 0,0426 | 0,0435 | 0,0444 | 0,0468 | 0,047 | 0,0502 | 0,0533 | 0,0576 | 0,0551 | 0,0522 | 0,0488 | 0,0499 | 0,0473 | 0,0479 | 0,0473 | 0,0473 | 0,0471 | 0,047 | 0,0478 | 0,0476 | 0,0473 | 0,0476 | 0,0474 | 0,0478 | 0,0478 | 0,0472 | 0,0478 | 0,0477 | 0,0481 | 0,0477 | 0,0475 | 0,0478 | 0,0475 | 0,0477 | 0,0473 | 0,0474 | 0,048 | 0,0481 | 0,0475 | 0,0478 | 0,0476 | 0,0487 | 0,0484 | 0,048 | 0,0477 | 0,0477 |
| G12 | 0,0422 | 0,044 | 0,0443 | 0,0501 | 0,0461 | 0,0478 | 0,046 | 0,0464 | 0,0463 | 0,0463 | 0,0464 | 0,0464 | 0,0462 | 0,0464 | 0,0465 | 0,0466 | 0,0464 | 0,0464 | 0,0466 | 0,0465 | 0,0465 | 0,0464 | 0,0465 | 0,0483 | 0,0466 | 0,0469 | 0,047 | 0,0469 | 0,0466 | 0,0467 | 0,0469 | 0,0471 | 0,0471 | 0,0469 | 0,0473 | 0,0469 | 0,0468 | 0,0469 | 0,0467 | 0,0471 | 0,047 | 0,0474 | 0,0473 | 0,0473 | 0,0473 | 0,0471 |
| H1 | 0,0523 | 0,0529 | 0,0562 | 0,0525 | 0,0524 | 0,0525 | 0,0514 | 0,0521 | 0,0523 | 0,0522 | 0,0525 | 0,0527 | 0,0525 | 0,0526 | 0,0529 | 0,0526 | 0,0529 | 0,0533 | 0,0545 | 0,0594 | 0,0535 | 0,0533 | 0,0546 | 0,0538 | 0,054 | 0,054 | 0,0545 | 0,0544 | 0,0554 | 0,0544 | 0,0545 | 0,0547 | 0,0547 | 0,0548 | 0,055 | 0,0551 | 0,0551 | 0,0551 | 0,0555 | 0,0558 | 0,056 | 0,0561 | 0,056 | 0,0576 | 0,0562 | 0,0563 |
| H2 | 0,0539 | 0,0556 | 0,0546 | 0,0553 | 0,0594 | 0,0569 | 0,0581 | 0,0538 | 0,0542 | 0,0535 | 0,0521 | 0,0524 | 0,0537 | 0,0523 | 0,0531 | 0,0529 | 0,0531 | 0,0531 | 0,053 | 0,0533 | 0,0535 | 0,0534 | 0,0541 | 0,054 | 0,0539 | 0,0542 | 0,0545 | 0,054 | 0,0542 | 0,0545 | 0,0542 | 0,0554 | 0,0547 | 0,0548 | 0,0549 | 0,055 | 0,0552 | 0,0547 | 0,0551 | 0,0558 | 0,0558 | 0,0558 | 0,0551 | 0,0568 | 0,0561 | 0,0558 |
| H3 | 0,0664 | 0,0692 | 0,072 | 0,0551 | 0,059 | 0,058 | 0,0557 | 0,0549 | 0,0544 | 0,0543 | 0,0529 | 0,0533 | 0,0541 | 0,0536 | 0,0536 | 0,0535 | 0,0539 | 0,0538 | 0,0535 | 0,0546 | 0,054 | 0,0541 | 0,0549 | 0,0547 | 0,0543 | 0,0543 | 0,0553 | 0,0546 | 0,0551 | 0,0555 | 0,055 | 0,0565 | 0,0565 | 0,0561 | 0,0564 | 0,056 | 0,0559 | 0,0554 | 0,0559 | 0,0571 | 0,0565 | 0,0565 | 0,0557 | 0,0584 | 0,0574 | 0,0566 |
| H4 | 0,0674 | 0,0674 | 0,0716 | 0,07 | 0,0721 | 0,071 | 0,07 | 0,0657 | 0,0665 | 0,0645 | 0,0642 | 0,0646 | 0,0658 | 0,0651 | 0,0651 | 0,0648 | 0,0658 | 0,0656 | 0,0658 | 0,0661 | 0,0659 | 0,0661 | 0,0669 | 0,0674 | 0,0672 | 0,0672 | 0,0683 | 0,0672 | 0,0687 | 0,0685 | 0,0683 | 0,0687 | 0,0693 | 0,0695 | 0,0696 | 0,0692 | 0,0695 | 0,0691 | 0,0692 | 0,0711 | 0,0699 | 0,0701 | 0,0695 | 0,0722 | 0,0718 | 0,0705 |
| H5 | 0,0604 | 0,0634 | 0,0674 | 0,0696 | 0,0736 | 0,0792 | 0,0758 | 0,0791 | 0,08 | 0,0759 | 0,0743 | 0,0679 | 0,0697 | 0,0643 | 0,069 | 0,067 | 0,0644 | 0,0664 | 0,0646 | 0,0646 | 0,0651 | 0,0648 | 0,0658 | 0,0657 | 0,0658 | 0,0661 | 0,067 | 0,0663 | 0,0667 | 0,067 | 0,0666 | 0,067 | 0,0683 | 0,068 | 0,0685 | 0,0675 | 0,0674 | 0,067 | 0,0677 | 0,0689 | 0,0685 | 0,069 | 0,0674 | 0,0707 | 0,0685 | 0,0685 |
| H6 | 0,0611 | 0,062 | 0,0631 | 0,0725 | 0,07 | 0,0837 | 0,0787 | 0,0782 | 0,0767 | 0,0804 | 0,0763 | 0,0691 | 0,0742 | 0,0645 | 0,0666 | 0,0655 | 0,0664 | 0,0663 | 0,0669 | 0,0655 | 0,0669 | 0,0668 | 0,0684 | 0,0686 | 0,069 | 0,0685 | 0,0705 | 0,068 | 0,0688 | 0,0681 | 0,0672 | 0,0696 | 0,0705 | 0,0702 | 0,0701 | 0,0702 | 0,0706 | 0,0692 | 0,0705 | 0,0722 | 0,0719 | 0,0719 | 0,0703 | 0,0723 | 0,072 | 0,0718 |
| H7 | 0,0801 | 0,0795 | 0,0829 | 0,0876 | 0,0875 | 0,0932 | 0,0912 | 0,0915 | 0,0897 | 0,0898 | 0,0838 | 0,0829 | 0,0843 | 0,0833 | 0,0837 | 0,0846 | 0,0852 | 0,0847 | 0,085 | 0,086 | 0,0856 | 0,0857 | 0,087 | 0,0875 | 0,0878 | 0,0872 | 0,0887 | 0,087 | 0,0891 | 0,0889 | 0,0883 | 0,0892 | 0,0899 | 0,0898 | 0,0902 | 0,0901 | 0,0902 | 0,0897 | 0,0902 | 0,0916 | 0,0911 | 0,0916 | 0,0906 | 0,0923 | 0,093 | 0,0923 |
| H8 | 0,0797 | 0,0786 | 0,0831 | 0,0835 | 0,0869 | 0,0907 | 0,0935 | 0,0889 | 0,0882 | 0,0844 | 0,0822 | 0,0828 | 0,0833 | 0,0836 | 0,0835 | 0,0846 | 0,0851 | 0,0846 | 0,0856 | 0,0856 | 0,0861 | 0,0859 | 0,0874 | 0,0886 | 0,0875 | 0,0881 | 0,0891 | 0,0875 | 0,0897 | 0,0892 | 0,0891 | 0,0892 | 0,0909 | 0,0906 | 0,0911 | 0,0909 | 0,0911 | 0,0904 | 0,0912 | 0,0923 | 0,0915 | 0,093 | 0,0912 | 0,093 | 0,0942 | 0,0931 |
| H9 | 0,0805 | 0,0807 | 0,082 | 0,0814 | 0,0847 | 0,0927 | 0,0897 | 0,0878 | 0,0884 | 0,0839 | 0,0843 | 0,0829 | 0,0836 | 0,0841 | 0,0839 | 0,0857 | 0,0861 | 0,0857 | 0,0862 | 0,0873 | 0,0873 | 0,0866 | 0,0884 | 0,0887 | 0,0888 | 0,0891 | 0,0894 | 0,0887 | 0,0894 | 0,0902 | 0,09 | 0,0905 | 0,0914 | 0,0915 | 0,0918 | 0,0909 | 0,0915 | 0,0909 | 0,0923 | 0,0926 | 0,0928 | 0,0942 | 0,0923 | 0,0945 | 0,0936 | 0,0944 |
| H10 | 0,0985 | 0,0959 | 0,0977 | 0,0993 | 0,0989 | 0,0992 | 0,099 | 0,0996 | 0,099 | 0,1001 | 0,1002 | 0,1011 | 0,1017 | 0,1026 | 0,1025 | 0,1036 | 0,1047 | 0,1039 | 0,1046 | 0,106 | 0,1055 | 0,1055 | 0,1076 | 0,1076 | 0,1075 | 0,1074 | 0,108 | 0,1075 | 0,1082 | 0,109 | 0,1087 | 0,1105 | 0,11 | 0,1109 | 0,1105 | 0,1109 | 0,1115 | 0,1106 | 0,112 | 0,1125 | 0,1133 | 0,1134 | 0,1128 | 0,1141 | 0,1145 | 0,1148 |
| H11 | 0,0966 | 0,0965 | 0,1035 | 0,1039 | 0,1003 | 0,0985 | 0,098 | 0,0986 | 0,0988 | 0,0999 | 0,1001 | 0,1019 | 0,1013 | 0,1025 | 0,103 | 0,1038 | 0,1041 | 0,1042 | 0,1046 | 0,1059 | 0,1053 | 0,1054 | 0,1062 | 0,1068 | 0,1069 | 0,1072 | 0,1081 | 0,1075 | 0,108 | 0,1085 | 0,1083 | 0,1098 | 0,1101 | 0,1107 | 0,1103 | 0,1108 | 0,1109 | 0,1111 | 0,1116 | 0,1123 | 0,1128 | 0,113 | 0,113 | 0,1135 | 0,1144 | 0,1142 |
| H12 | 0,0959 | 0,096 | 0,0966 | 0,0976 | 0,0989 | 0,0986 | 0,1002 | 0,1002 | 0,1013 | 0,1012 | 0,1019 | 0,1032 | 0,103 | 0,1044 | 0,1041 | 0,1061 | 0,1057 | 0,1062 | 0,1071 | 0,1075 | 0,1075 | 0,1076 | 0,1082 | 0,11 | 0,1092 | 0,1108 | 0,1107 | 0,1106 | 0,1114 | 0,1118 | 0,1127 | 0,1127 | 0,113 | 0,1131 | 0,113 | 0,1141 | 0,1138 | 0,1146 | 0,1147 | 0,1148 | 0,1154 | 0,1171 | 0,1161 | 0,1165 | 0,1176 | 0,1173 |

End Time: 2020 09-29 15:37:25

**Raw data(triplicates) 5000nM WT PTPN22**

**Raw data(triplicates) 5000nM C129S PTPN22**

**1.5**

**1.0**

**0.5**

**0.0**


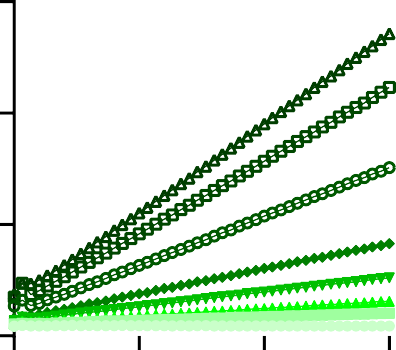


**0 5 10 15 20**

**Time(min)**

##### WT 0

WT 0.125mM WT 0.25mM WT 0.5mM WT 1mM

##### WT 2.5mM WT 5mM WT 7.5mM

**0.4**

**0.3**

**0.2**

**A405**

**0.1**

**0.0**


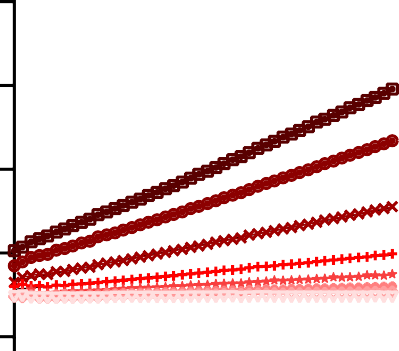


**0 5 10 15 20**

#### Time(min)

##### MUT 0


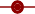

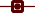
MUT 0.125mM MUT 0.25mM MUT 0.5mM MUT 1mM MUT 2.5mM MUT 5mM MUT 7.5mM

# wt

**0.10**


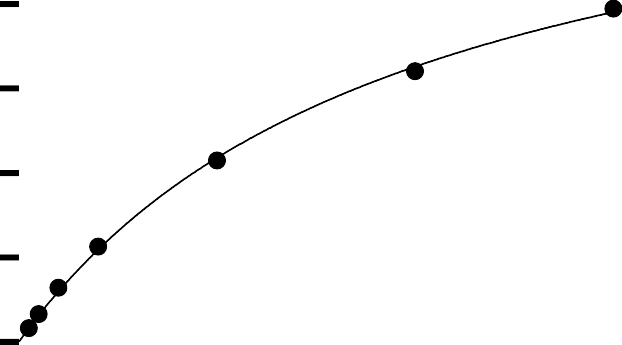


Km=4.91

**0.08**

**0.06**

**slop**

**0.015**

**0.010**


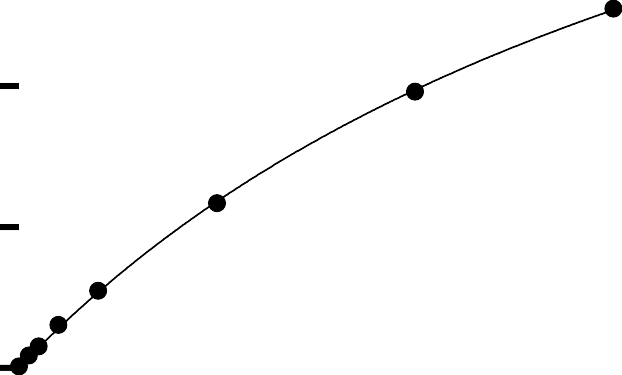


Km=10.14

**slop**

**mut**

**0.04**

**0.02**

**0.005**

**0.00**

**A405**

**0 2 4 6 8**

**mM(p-NPP)**

**0.000**

**0 2 4 6 8**

## mM(p-NPP)

List of actions in this measurement script: Kinetic

Shaking (Linear) Duration: Shaking (Linear) Amplitude: Absorbance

2 s

1 mm

Label: Label1

Kinetic Measurement

Kinetic duration 00:25:00

Interval Time 00:00:20

Measurement Wavelength 405 nm

Bandwidth 9 nm

Number of Flashes 5

Settle Time 0 ms

Part of Plate B1-E12

Start Time: 2020 09-30 13:57:22

Cycle Nr.

1

2

3

4

5

6

7

8

9

10 11 12 13 14 15 16 17 18 19 20 21 22 23 24 25 26 27 28 29 30 31 32 33 34 35 36 37 38 39 40 41 42 43 44 45 46

|  |  |  |  |  |  |  |  |  |  |  |  |  |  |  |  |  |  |  |  |  |  |  |  |  |  |  |  |  |  |  |  |  |  |  |  |  |  |  |  |  |  |  |  |  |  |  |
| --- | --- | --- | --- | --- | --- | --- | --- | --- | --- | --- | --- | --- | --- | --- | --- | --- | --- | --- | --- | --- | --- | --- | --- | --- | --- | --- | --- | --- | --- | --- | --- | --- | --- | --- | --- | --- | --- | --- | --- | --- | --- | --- | --- | --- | --- | --- |
| Time [s] | 0 | 20 | 40 | 60 | 80 | 100 | 120 | 140 | 160 | 180 | 200 | 220 | 240 | 260 | 280 | 300 | 320 | 340 | 360 | 380 | 400 | 420 | 440 | 460 | 480 | 500 | 520 | 540 | 560 | 580 | 600 | 620 | 640 | 660 | 680 | 700 | 720 | 740 | 760 | 780 | 800 | 820 | 840 | 860 | 880 | 900 |
| Temp. [°C] | 27,3 | 27,2 | 27,2 | 27,3 | 27,2 | 27,3 | 27,3 | 27,4 | 27,4 | 27,4 | 27,2 | 27,5 | 27,6 | 27,6 | 27,3 | 27,4 | 27,3 | 27,6 | 27,4 | 27,6 | 27,6 | 27,7 | 27,5 | 27,5 | 27,9 | 27,9 | 27,9 | 27,6 | 27,6 | 28 | 27,7 | 27,9 | 27,9 | 27,7 | 27,7 | 27,7 | 27,9 | 28 | 27,6 | 28 | 27,9 | 28 | 27,9 | 28 | 27,9 | 27,9 |
| B1 | 0,0441 | 0,0441 | 0,0441 | 0,044 | 0,0441 | 0,0439 | 0,0444 | 0,0443 | 0,0442 | 0,0443 | 0,0443 | 0,0447 | 0,0442 | 0,0442 | 0,0444 | 0,0444 | 0,0444 | 0,0443 | 0,0443 | 0,0443 | 0,0442 | 0,0444 | 0,0444 | 0,0444 | 0,0444 | 0,0444 | 0,0445 | 0,0444 | 0,0443 | 0,0442 | 0,0445 | 0,0442 | 0,0444 | 0,0445 | 0,0443 | 0,0441 | 0,0441 | 0,0441 | 0,0443 | 0,0442 | 0,0446 | 0,0443 | 0,0444 | 0,0444 | 0,0445 | 0,0446 |
| B2 | 0,0433 | 0,0452 | 0,0456 | 0,0462 | 0,0452 | 0,0479 | 0,0476 | 0,0474 | 0,0467 | 0,0452 | 0,0445 | 0,0449 | 0,0451 | 0,0451 | 0,0445 | 0,0445 | 0,0451 | 0,0446 | 0,0445 | 0,0445 | 0,0447 | 0,0445 | 0,0446 | 0,0445 | 0,0444 | 0,0447 | 0,0451 | 0,0445 | 0,0445 | 0,0444 | 0,0447 | 0,0446 | 0,0446 | 0,0448 | 0,0444 | 0,0445 | 0,0446 | 0,0445 | 0,0446 | 0,0445 | 0,0452 | 0,0449 | 0,045 | 0,0446 | 0,045 | 0,0452 |
| B3 | 0,0428 | 0,0501 | 0,0447 | 0,0442 | 0,0442 | 0,0449 | 0,0459 | 0,0478 | 0,0461 | 0,0497 | 0,0486 | 0,049 | 0,0475 | 0,0497 | 0,048 | 0,0464 | 0,0467 | 0,0487 | 0,048 | 0,0451 | 0,0468 | 0,0446 | 0,0484 | 0,0465 | 0,046 | 0,0449 | 0,0447 | 0,0448 | 0,045 | 0,0446 | 0,0448 | 0,0448 | 0,0448 | 0,0449 | 0,0446 | 0,0447 | 0,0446 | 0,045 | 0,0445 | 0,0443 | 0,0445 | 0,0448 | 0,0446 | 0,0445 | 0,0444 | 0,0447 |
| B4 | 0,0506 | 0,0577 | 0,0534 | 0,0542 | 0,0548 | 0,0563 | 0,0578 | 0,0598 | 0,0602 | 0,0629 | 0,0649 | 0,0669 | 0,0671 | 0,0688 | 0,0707 | 0,0712 | 0,0736 | 0,0737 | 0,0763 | 0,0738 | 0,077 | 0,0755 | 0,0774 | 0,079 | 0,0808 | 0,0803 | 0,0818 | 0,0843 | 0,0848 | 0,0847 | 0,0858 | 0,0868 | 0,0881 | 0,0893 | 0,0902 | 0,0913 | 0,0922 | 0,0935 | 0,0945 | 0,0962 | 0,0972 | 0,0979 | 0,099 | 0,0999 | 0,1013 | 0,1026 |
| B5 | 0,0588 | 0,0553 | 0,0532 | 0,0574 | 0,0553 | 0,0568 | 0,0588 | 0,0603 | 0,0618 | 0,0634 | 0,0639 | 0,0678 | 0,0664 | 0,0662 | 0,0671 | 0,0694 | 0,0691 | 0,0703 | 0,0717 | 0,0726 | 0,0737 | 0,0793 | 0,0763 | 0,0799 | 0,0782 | 0,0794 | 0,085 | 0,0831 | 0,083 | 0,0841 | 0,0854 | 0,0865 | 0,0877 | 0,0916 | 0,0902 | 0,0906 | 0,0917 | 0,0935 | 0,0952 | 0,0953 | 0,0965 | 0,1003 | 0,0987 | 0,1001 | 0,1023 | 0,1029 |
| B6 | 0,0482 | 0,052 | 0,0553 | 0,0536 | 0,0542 | 0,0557 | 0,0583 | 0,0623 | 0,0615 | 0,0625 | 0,0638 | 0,0652 | 0,0653 | 0,0664 | 0,0663 | 0,0673 | 0,0683 | 0,0706 | 0,0706 | 0,0714 | 0,0734 | 0,0739 | 0,0753 | 0,0766 | 0,0781 | 0,0787 | 0,0799 | 0,0808 | 0,0821 | 0,083 | 0,0846 | 0,0857 | 0,0865 | 0,0878 | 0,0889 | 0,0898 | 0,091 | 0,0921 | 0,0933 | 0,0945 | 0,0956 | 0,0968 | 0,098 | 0,0992 | 0,1001 | 0,1016 |
| B7 | 0,0541 | 0,067 | 0,0624 | 0,0631 | 0,0635 | 0,0662 | 0,0689 | 0,0714 | 0,0733 | 0,0758 | 0,0794 | 0,0807 | 0,0832 | 0,0869 | 0,0906 | 0,0912 | 0,093 | 0,0979 | 0,1011 | 0,0997 | 0,1035 | 0,104 | 0,1053 | 0,11 | 0,1133 | 0,113 | 0,1157 | 0,1165 | 0,1216 | 0,123 | 0,1235 | 0,126 | 0,1282 | 0,1299 | 0,133 | 0,1363 | 0,1376 | 0,1397 | 0,1414 | 0,1432 | 0,1452 | 0,1476 | 0,1502 | 0,1523 | 0,1541 | 0,1562 |
| B8 | 0,0575 | 0,0615 | 0,0609 | 0,0639 | 0,0637 | 0,0694 | 0,0711 | 0,0717 | 0,0739 | 0,0757 | 0,0772 | 0,0794 | 0,0837 | 0,0855 | 0,0859 | 0,0882 | 0,0906 | 0,0926 | 0,0954 | 0,0973 | 0,0995 | 0,1017 | 0,1041 | 0,1064 | 0,1088 | 0,1108 | 0,113 | 0,1154 | 0,1177 | 0,1204 | 0,1222 | 0,1241 | 0,1267 | 0,1292 | 0,1312 | 0,1331 | 0,1352 | 0,1377 | 0,1397 | 0,1418 | 0,1444 | 0,1465 | 0,1488 | 0,1507 | 0,1528 | 0,1552 |
| B9 | 0,0563 | 0,0657 | 0,0605 | 0,0628 | 0,0644 | 0,0671 | 0,0693 | 0,0716 | 0,0754 | 0,0775 | 0,08 | 0,0823 | 0,0867 | 0,087 | 0,0879 | 0,091 | 0,0941 | 0,0951 | 0,0973 | 0,0985 | 0,1014 | 0,1038 | 0,1061 | 0,1082 | 0,1102 | 0,1123 | 0,1146 | 0,1168 | 0,1191 | 0,1214 | 0,1238 | 0,126 | 0,1282 | 0,1306 | 0,1325 | 0,135 | 0,137 | 0,1392 | 0,1412 | 0,144 | 0,1459 | 0,1482 | 0,1502 | 0,1524 | 0,1547 | 0,1573 |
| B10 | 0,0682 | 0,0823 | 0,0786 | 0,0799 | 0,0832 | 0,0889 | 0,0941 | 0,0986 | 0,1032 | 0,1062 | 0,1093 | 0,1136 | 0,1183 | 0,1233 | 0,1258 | 0,1299 | 0,1339 | 0,1386 | 0,1423 | 0,1466 | 0,1512 | 0,1556 | 0,1603 | 0,1642 | 0,1687 | 0,1729 | 0,1769 | 0,1813 | 0,1856 | 0,1897 | 0,1943 | 0,1987 | 0,2021 | 0,2067 | 0,2107 | 0,215 | 0,2193 | 0,2232 | 0,228 | 0,2325 | 0,2362 | 0,2398 | 0,2444 | 0,2487 | 0,2529 | 0,2573 |
| B11 | 0,0659 | 0,0827 | 0,0774 | 0,0779 | 0,0814 | 0,0865 | 0,0916 | 0,0959 | 0,1004 | 0,1043 | 0,1081 | 0,113 | 0,118 | 0,1229 | 0,1268 | 0,1302 | 0,1351 | 0,1402 | 0,1446 | 0,1482 | 0,1541 | 0,1566 | 0,1623 | 0,17 | 0,1741 | 0,1751 | 0,1801 | 0,1818 | 0,1906 | 0,1928 | 0,1958 | 0,1998 | 0,2045 | 0,2086 | 0,2137 | 0,2179 | 0,2216 | 0,2267 | 0,2306 | 0,2342 | 0,2376 | 0,2421 | 0,2474 | 0,2518 | 0,2553 | 0,2585 |
| B12 | 0,0708 | 0,0872 | 0,0775 | 0,0813 | 0,0844 | 0,0901 | 0,0931 | 0,096 | 0,1008 | 0,1053 | 0,1098 | 0,1141 | 0,1189 | 0,1231 | 0,1277 | 0,1318 | 0,1365 | 0,1411 | 0,1455 | 0,1497 | 0,1543 | 0,159 | 0,1634 | 0,168 | 0,1728 | 0,1771 | 0,1817 | 0,186 | 0,191 | 0,1953 | 0,2002 | 0,2039 | 0,2094 | 0,2139 | 0,2179 | 0,222 | 0,2268 | 0,231 | 0,2353 | 0,2395 | 0,2441 | 0,2488 | 0,253 | 0,2573 | 0,2611 | 0,265 |
| C1 | 0,0739 | 0,0823 | 0,0927 | 0,1005 | 0,1085 | 0,1176 | 0,1262 | 0,1338 | 0,1445 | 0,1535 | 0,1575 | 0,1649 | 0,175 | 0,1836 | 0,1922 | 0,1987 | 0,2027 | 0,2149 | 0,2233 | 0,2257 | 0,2381 | 0,2413 | 0,2516 | 0,2571 | 0,2663 | 0,2723 | 0,2795 | 0,2878 | 0,2958 | 0,3032 | 0,3116 | 0,3184 | 0,3261 | 0,334 | 0,3427 | 0,3497 | 0,3584 | 0,3632 | 0,3725 | 0,3799 | 0,3867 | 0,3955 | 0,4029 | 0,4119 | 0,4162 | 0,4259 |
| C2 | 0,084 | 0,0845 | 0,0892 | 0,0942 | 0,1016 | 0,1088 | 0,1154 | 0,123 | 0,1308 | 0,1392 | 0,145 | 0,1528 | 0,1612 | 0,1682 | 0,1762 | 0,1834 | 0,1909 | 0,1989 | 0,2065 | 0,2135 | 0,2216 | 0,2289 | 0,238 | 0,244 | 0,2523 | 0,2596 | 0,2669 | 0,2752 | 0,2823 | 0,2897 | 0,2982 | 0,3051 | 0,3129 | 0,3206 | 0,3284 | 0,3354 | 0,3436 | 0,3499 | 0,3573 | 0,3649 | 0,3724 | 0,3799 | 0,3872 | 0,395 | 0,4015 | 0,4096 |
| C3 | 0,0817 | 0,0939 | 0,0922 | 0,0977 | 0,1049 | 0,112 | 0,1169 | 0,1242 | 0,1331 | 0,1412 | 0,1465 | 0,1542 | 0,1641 | 0,1704 | 0,1782 | 0,1848 | 0,1908 | 0,2006 | 0,2087 | 0,2137 | 0,2225 | 0,2291 | 0,2383 | 0,2436 | 0,2529 | 0,2593 | 0,2657 | 0,2755 | 0,2818 | 0,2895 | 0,2978 | 0,305 | 0,3131 | 0,3195 | 0,328 | 0,3347 | 0,3421 | 0,347 | 0,3554 | 0,3635 | 0,3719 | 0,3786 | 0,3836 | 0,3924 | 0,3983 | 0,4078 |
| C4 | 0,1496 | 0,1496 | 0,1349 | 0,1457 | 0,1613 | 0,176 | 0,1849 | 0,2 | 0,2144 | 0,2294 | 0,2422 | 0,2577 | 0,2764 | 0,2854 | 0,3005 | 0,3146 | 0,3291 | 0,3431 | 0,3601 | 0,3715 | 0,3864 | 0,4013 | 0,415 | 0,4292 | 0,4444 | 0,4604 | 0,4727 | 0,49 | 0,5048 | 0,518 | 0,5342 | 0,5477 | 0,5601 | 0,5767 | 0,592 | 0,606 | 0,6212 | 0,6319 | 0,6488 | 0,6643 | 0,6791 | 0,6924 | 0,7047 | 0,7222 | 0,7328 | 0,7513 |
| C5 | 0,1185 | 0,1628 | 0,1379 | 0,1454 | 0,1597 | 0,1724 | 0,1858 | 0,2003 | 0,2156 | 0,2301 | 0,2429 | 0,2577 | 0,2727 | 0,2874 | 0,3019 | 0,3165 | 0,3297 | 0,3452 | 0,3589 | 0,3736 | 0,387 | 0,4032 | 0,4192 | 0,4322 | 0,4487 | 0,4616 | 0,4759 | 0,4921 | 0,5065 | 0,5219 | 0,5365 | 0,5511 | 0,5655 | 0,5806 | 0,5953 | 0,6098 | 0,6245 | 0,6387 | 0,6528 | 0,6668 | 0,6821 | 0,6974 | 0,7108 | 0,7276 | 0,7398 | 0,7551 |
| C6 | 0,1362 | 0,1697 | 0,1391 | 0,1474 | 0,1631 | 0,1746 | 0,1882 | 0,2022 | 0,2167 | 0,2315 | 0,245 | 0,2593 | 0,275 | 0,2891 | 0,3047 | 0,3181 | 0,3327 | 0,3487 | 0,3622 | 0,3764 | 0,3914 | 0,4067 | 0,4216 | 0,4355 | 0,4518 | 0,466 | 0,4798 | 0,4973 | 0,5106 | 0,5253 | 0,5421 | 0,5562 | 0,5708 | 0,5864 | 0,6009 | 0,6166 | 0,6308 | 0,6452 | 0,6579 | 0,6721 | 0,688 | 0,703 | 0,7149 | 0,7308 | 0,7449 | 0,7616 |
| C7 | 0,1876 | 0,2476 | 0,191 | 0,2093 | 0,231 | 0,2514 | 0,2708 | 0,2906 | 0,3137 | 0,3344 | 0,3541 | 0,3748 | 0,3994 | 0,4195 | 0,4403 | 0,4601 | 0,481 | 0,5055 | 0,5285 | 0,5451 | 0,5692 | 0,5888 | 0,6105 | 0,6324 | 0,6541 | 0,6748 | 0,6972 | 0,7192 | 0,739 | 0,7629 | 0,785 | 0,8077 | 0,8306 | 0,85 | 0,8726 | 0,8931 | 0,9174 | 0,9361 | 0,9578 | 0,9825 | 1,0056 | 1,0263 | 1,0437 | 1,068 | 1,0904 | 1,115 |
| C8 | 0,1878 | 0,2253 | 0,1921 | 0,2026 | 0,2222 | 0,2429 | 0,2621 | 0,2836 | 0,3045 | 0,326 | 0,3471 | 0,3685 | 0,3901 | 0,4108 | 0,4324 | 0,4544 | 0,4756 | 0,4967 | 0,5195 | 0,5374 | 0,5607 | 0,5818 | 0,6058 | 0,6258 | 0,6474 | 0,6675 | 0,689 | 0,7119 | 0,7338 | 0,7555 | 0,7768 | 0,8 | 0,8213 | 0,8425 | 0,8651 | 0,8862 | 0,9077 | 0,9306 | 0,9521 | 0,9743 | 0,9962 | 1,0166 | 1,0387 | 1,0614 | 1,085 | 1,1078 |
| C9 | 0,1495 | 0,2388 | 0,175 | 0,1968 | 0,2217 | 0,2432 | 0,2639 | 0,2869 | 0,3086 | 0,3303 | 0,3521 | 0,3713 | 0,3945 | 0,4161 | 0,4385 | 0,4591 | 0,4804 | 0,5032 | 0,5248 | 0,5462 | 0,5698 | 0,5916 | 0,6134 | 0,6345 | 0,6571 | 0,6795 | 0,7005 | 0,7226 | 0,7455 | 0,7687 | 0,7904 | 0,8131 | 0,8352 | 0,8582 | 0,8805 | 0,9021 | 0,9231 | 0,9473 | 0,9698 | 0,9914 | 1,0115 | 1,0355 | 1,0559 | 1,0832 | 1,1068 | 1,1237 |
| C10 | 0,1882 | 0,2238 | 0,2392 | 0,2553 | 0,2795 | 0,2991 | 0,323 | 0,3473 | 0,3728 | 0,3984 | 0,4247 | 0,4477 | 0,4757 | 0,5011 | 0,528 | 0,5517 | 0,5782 | 0,6068 | 0,6308 | 0,6567 | 0,6848 | 0,7091 | 0,7351 | 0,7626 | 0,7876 | 0,8154 | 0,8429 | 0,8686 | 0,895 | 0,9212 | 0,949 | 0,9751 | 1,004 | 1,0326 | 1,0577 | 1,0837 | 1,1128 | 1,1379 | 1,1609 | 1,1891 | 1,2155 | 1,2442 | 1,2705 | 1,2928 | 1,3237 | 1,3508 |
| C11 | 0,186 | 0,2531 | 0,2354 | 0,2506 | 0,2683 | 0,2905 | 0,3157 | 0,3413 | 0,3678 | 0,3933 | 0,4194 | 0,4451 | 0,4722 | 0,4971 | 0,5227 | 0,5498 | 0,5762 | 0,6028 | 0,6279 | 0,6525 | 0,6789 | 0,7059 | 0,7338 | 0,7598 | 0,7859 | 0,8104 | 0,8405 | 0,867 | 0,893 | 0,9196 | 0,9481 | 0,9755 | 1,0032 | 1,0286 | 1,0531 | 1,0826 | 1,1103 | 1,1367 | 1,1616 | 1,1908 | 1,2192 | 1,247 | 1,271 | 1,2974 | 1,3251 | 1,3554 |
| C12 | 0,1834 | 0,237 | 0,228 | 0,2464 | 0,2679 | 0,2914 | 0,3173 | 0,3421 | 0,3678 | 0,3946 | 0,4211 | 0,4459 | 0,4735 | 0,5002 | 0,5266 | 0,5527 | 0,577 | 0,6053 | 0,6315 | 0,6563 | 0,6845 | 0,7097 | 0,74 | 0,7646 | 0,793 | 0,8218 | 0,8466 | 0,8733 | 0,9024 | 0,9306 | 0,9569 | 0,986 | 1,0128 | 1,0382 | 1,0675 | 1,0952 | 1,1239 | 1,1516 | 1,1789 | 1,2043 | 1,2345 | 1,262 | 1,2874 | 1,3157 | 1,3415 | 1,3653 |
| D1 | 0,0471 | 0,0493 | 0,0469 | 0,0467 | 0,0468 | 0,0463 | 0,0464 | 0,046 | 0,047 | 0,0467 | 0,0465 | 0,0463 | 0,0471 | 0,0465 | 0,0466 | 0,0465 | 0,0465 | 0,0467 | 0,0466 | 0,0469 | 0,0465 | 0,0471 | 0,0469 | 0,0469 | 0,0469 | 0,0471 | 0,0469 | 0,0465 | 0,047 | 0,0472 | 0,047 | 0,0469 | 0,0469 | 0,0469 | 0,0469 | 0,0469 | 0,0469 | 0,0467 | 0,0468 | 0,0466 | 0,0468 | 0,0471 | 0,0469 | 0,0471 | 0,0468 | 0,0468 |
| D2 | 0,0484 | 0,0505 | 0,0473 | 0,0474 | 0,0478 | 0,0475 | 0,0477 | 0,0477 | 0,0475 | 0,0476 | 0,0472 | 0,0478 | 0,048 | 0,0483 | 0,0489 | 0,0489 | 0,0489 | 0,0487 | 0,0487 | 0,0485 | 0,0488 | 0,0487 | 0,0489 | 0,0487 | 0,0487 | 0,0491 | 0,0492 | 0,0487 | 0,0491 | 0,0491 | 0,0492 | 0,0492 | 0,0489 | 0,0491 | 0,0489 | 0,0489 | 0,0491 | 0,0489 | 0,0489 | 0,0491 | 0,0492 | 0,049 | 0,0491 | 0,0493 | 0,0493 | 0,0493 |
| D3 | 0,0492 | 0,0469 | 0,0454 | 0,045 | 0,0448 | 0,0452 | 0,0454 | 0,0451 | 0,0451 | 0,0452 | 0,0458 | 0,0456 | 0,0461 | 0,0465 | 0,047 | 0,0464 | 0,0468 | 0,0465 | 0,0467 | 0,0465 | 0,0465 | 0,0466 | 0,0467 | 0,0465 | 0,0467 | 0,0468 | 0,047 | 0,0463 | 0,0468 | 0,0473 | 0,0469 | 0,0467 | 0,0466 | 0,0469 | 0,0465 | 0,0464 | 0,0467 | 0,0465 | 0,0467 | 0,0469 | 0,0464 | 0,0467 | 0,0469 | 0,047 | 0,0469 | 0,0469 |
| D4 | 0,0472 | 0,0469 | 0,0463 | 0,0462 | 0,0468 | 0,0469 | 0,047 | 0,047 | 0,0473 | 0,0474 | 0,0472 | 0,048 | 0,0485 | 0,0487 | 0,0492 | 0,0494 | 0,0497 | 0,0496 | 0,0501 | 0,0498 | 0,0499 | 0,05 | 0,0504 | 0,0504 | 0,0506 | 0,0507 | 0,051 | 0,0506 | 0,0511 | 0,0515 | 0,0513 | 0,0515 | 0,0516 | 0,052 | 0,0517 | 0,0519 | 0,0522 | 0,0519 | 0,0522 | 0,0525 | 0,0525 | 0,0526 | 0,0529 | 0,0531 | 0,053 | 0,0534 |
| D5 | 0,0505 | 0,047 | 0,0503 | 0,047 | 0,047 | 0,0469 | 0,0471 | 0,0474 | 0,0475 | 0,048 | 0,0475 | 0,0486 | 0,0492 | 0,0495 | 0,0497 | 0,0497 | 0,0502 | 0,0499 | 0,0502 | 0,05 | 0,0503 | 0,0506 | 0,0507 | 0,0509 | 0,0507 | 0,051 | 0,0515 | 0,0507 | 0,0515 | 0,0516 | 0,0518 | 0,0521 | 0,0518 | 0,0521 | 0,0519 | 0,0522 | 0,0525 | 0,0523 | 0,0525 | 0,053 | 0,0529 | 0,053 | 0,0534 | 0,0536 | 0,0535 | 0,0541 |
| D6 | 0,0464 | 0,0488 | 0,0485 | 0,0472 | 0,0472 | 0,0472 | 0,048 | 0,048 | 0,0479 | 0,0485 | 0,0478 | 0,049 | 0,0498 | 0,0497 | 0,0498 | 0,0499 | 0,0499 | 0,0501 | 0,0501 | 0,0502 | 0,0501 | 0,0506 | 0,0506 | 0,0508 | 0,0507 | 0,0511 | 0,0515 | 0,0508 | 0,0513 | 0,0515 | 0,0518 | 0,052 | 0,0516 | 0,052 | 0,0521 | 0,0522 | 0,0524 | 0,0522 | 0,0528 | 0,0529 | 0,0529 | 0,0529 | 0,0534 | 0,0535 | 0,0536 | 0,0537 |
| D7 | 0,0487 | 0,0513 | 0,0494 | 0,0484 | 0,0486 | 0,0493 | 0,0496 | 0,0497 | 0,0496 | 0,0504 | 0,0498 | 0,0511 | 0,0519 | 0,0525 | 0,0525 | 0,0526 | 0,0529 | 0,0531 | 0,0533 | 0,0534 | 0,0537 | 0,0541 | 0,0544 | 0,0548 | 0,0548 | 0,0552 | 0,0557 | 0,0547 | 0,0561 | 0,0563 | 0,0567 | 0,0568 | 0,0567 | 0,0574 | 0,0574 | 0,0575 | 0,0579 | 0,058 | 0,0584 | 0,0586 | 0,0587 | 0,0592 | 0,0598 | 0,06 | 0,0601 | 0,0604 |
| D8 | 0,0471 | 0,0483 | 0,0504 | 0,05 | 0,0499 | 0,0538 | 0,0543 | 0,052 | 0,0516 | 0,0522 | 0,0521 | 0,0553 | 0,0524 | 0,0524 | 0,0525 | 0,0532 | 0,053 | 0,0534 | 0,0538 | 0,0538 | 0,0538 | 0,0544 | 0,0548 | 0,0549 | 0,0552 | 0,0554 | 0,0557 | 0,0553 | 0,0562 | 0,0564 | 0,057 | 0,057 | 0,0573 | 0,058 | 0,0583 | 0,0579 | 0,0584 | 0,0584 | 0,0587 | 0,0588 | 0,0591 | 0,0595 | 0,0597 | 0,0601 | 0,06 | 0,0605 |
| D9 | 0,0476 | 0,0491 | 0,0532 | 0,0488 | 0,0491 | 0,049 | 0,0494 | 0,0498 | 0,0496 | 0,0504 | 0,0502 | 0,0513 | 0,0522 | 0,0524 | 0,0524 | 0,0527 | 0,053 | 0,0533 | 0,0534 | 0,0537 | 0,0538 | 0,0542 | 0,0545 | 0,0546 | 0,0549 | 0,0552 | 0,0556 | 0,0549 | 0,0559 | 0,0562 | 0,0566 | 0,057 | 0,057 | 0,0572 | 0,0573 | 0,0578 | 0,058 | 0,058 | 0,0585 | 0,0588 | 0,0593 | 0,0593 | 0,0594 | 0,0598 | 0,0601 | 0,0607 |
| D10 | 0,0502 | 0,052 | 0,0572 | 0,052 | 0,052 | 0,0525 | 0,0539 | 0,054 | 0,0541 | 0,055 | 0,0552 | 0,0563 | 0,0568 | 0,0572 | 0,0583 | 0,059 | 0,0591 | 0,0596 | 0,06 | 0,0604 | 0,0609 | 0,0615 | 0,0621 | 0,0626 | 0,0632 | 0,0636 | 0,0641 | 0,0638 | 0,0651 | 0,0658 | 0,0662 | 0,0667 | 0,0669 | 0,0675 | 0,0684 | 0,0687 | 0,0696 | 0,0693 | 0,0702 | 0,0715 | 0,0713 | 0,0721 | 0,0726 | 0,0728 | 0,0733 | 0,0739 |
| D11 | 0,0499 | 0,0512 | 0,056 | 0,0527 | 0,0536 | 0,054 | 0,055 | 0,0554 | 0,0553 | 0,0564 | 0,0566 | 0,056 | 0,057 | 0,058 | 0,0579 | 0,0581 | 0,0586 | 0,0591 | 0,0596 | 0,0615 | 0,0606 | 0,0618 | 0,0617 | 0,0624 | 0,0634 | 0,0635 | 0,0638 | 0,0635 | 0,065 | 0,0654 | 0,0661 | 0,0697 | 0,0669 | 0,0674 | 0,0679 | 0,0686 | 0,0693 | 0,0696 | 0,0726 | 0,071 | 0,0707 | 0,0712 | 0,0754 | 0,0745 | 0,0731 | 0,0762 |
| D12 | 0,0501 | 0,059 | 0,0566 | 0,0513 | 0,0518 | 0,0522 | 0,0533 | 0,0536 | 0,0536 | 0,0544 | 0,0547 | 0,0555 | 0,0564 | 0,0569 | 0,058 | 0,0584 | 0,0584 | 0,0589 | 0,0593 | 0,06 | 0,0602 | 0,0613 | 0,0617 | 0,062 | 0,0629 | 0,0633 | 0,0635 | 0,0643 | 0,065 | 0,0658 | 0,0662 | 0,0664 | 0,0675 | 0,0674 | 0,0681 | 0,0687 | 0,0691 | 0,0699 | 0,0703 | 0,0705 | 0,0715 | 0,0725 | 0,073 | 0,0737 | 0,0734 | 0,0742 |
| E1 | 0,0616 | 0,0677 | 0,0645 | 0,063 | 0,0611 | 0,0632 | 0,0617 | 0,0643 | 0,0639 | 0,0648 | 0,0654 | 0,0666 | 0,0672 | 0,0682 | 0,069 | 0,0699 | 0,0708 | 0,0717 | 0,0728 | 0,0733 | 0,0746 | 0,0759 | 0,0768 | 0,0779 | 0,0788 | 0,0804 | 0,0804 | 0,0813 | 0,0835 | 0,0846 | 0,085 | 0,0857 | 0,0868 | 0,0876 | 0,0884 | 0,0893 | 0,0907 | 0,0918 | 0,0927 | 0,0938 | 0,095 | 0,0957 | 0,0969 | 0,0985 | 0,0988 | 0,1002 |
| E2 | 0,0586 | 0,0618 | 0,0594 | 0,0608 | 0,0589 | 0,0616 | 0,061 | 0,0621 | 0,0631 | 0,0632 | 0,0645 | 0,0657 | 0,0665 | 0,0669 | 0,0681 | 0,069 | 0,0698 | 0,0708 | 0,0717 | 0,0725 | 0,0736 | 0,075 | 0,0759 | 0,0768 | 0,0779 | 0,0793 | 0,0795 | 0,0804 | 0,0821 | 0,0833 | 0,0836 | 0,0844 | 0,0858 | 0,0862 | 0,0872 | 0,0883 | 0,0897 | 0,0904 | 0,0914 | 0,0922 | 0,0934 | 0,0945 | 0,0952 | 0,0965 | 0,0971 | 0,0983 |
| E3 | 0,0603 | 0,0592 | 0,0588 | 0,0593 | 0,0584 | 0,0606 | 0,0604 | 0,0617 | 0,0626 | 0,0632 | 0,0646 | 0,0657 | 0,0662 | 0,0672 | 0,0681 | 0,0691 | 0,0701 | 0,0707 | 0,0719 | 0,0726 | 0,0739 | 0,0751 | 0,0758 | 0,0768 | 0,078 | 0,0791 | 0,0798 | 0,0804 | 0,0819 | 0,0834 | 0,0837 | 0,0848 | 0,0861 | 0,0864 | 0,0875 | 0,0882 | 0,0894 | 0,0904 | 0,0914 | 0,0923 | 0,0935 | 0,0941 | 0,0949 | 0,0963 | 0,0971 | 0,0985 |
| E4 | 0,0659 | 0,069 | 0,071 | 0,073 | 0,0728 | 0,0757 | 0,0767 | 0,079 | 0,0808 | 0,082 | 0,0851 | 0,0873 | 0,0887 | 0,0905 | 0,0924 | 0,0945 | 0,096 | 0,0978 | 0,0998 | 0,1017 | 0,1035 | 0,1058 | 0,1077 | 0,1096 | 0,1119 | 0,1139 | 0,1155 | 0,1173 | 0,1198 | 0,1225 | 0,1239 | 0,1257 | 0,1277 | 0,1296 | 0,1318 | 0,1333 | 0,1356 | 0,1377 | 0,1398 | 0,1419 | 0,144 | 0,1456 | 0,1478 | 0,1499 | 0,1518 | 0,1541 |
| E5 | 0,0649 | 0,0744 | 0,0734 | 0,0751 | 0,0744 | 0,0776 | 0,0777 | 0,0806 | 0,0821 | 0,0831 | 0,0854 | 0,0879 | 0,0893 | 0,0914 | 0,093 | 0,0951 | 0,0969 | 0,0989 | 0,101 | 0,1027 | 0,1045 | 0,107 | 0,1086 | 0,1107 | 0,1128 | 0,1149 | 0,1164 | 0,1182 | 0,1213 | 0,1233 | 0,1247 | 0,1266 | 0,129 | 0,1307 | 0,1327 | 0,1346 | 0,137 | 0,1391 | 0,1411 | 0,143 | 0,1451 | 0,1471 | 0,1491 | 0,1514 | 0,1531 | 0,1554 |
| E6 | 0,0647 | 0,0706 | 0,0754 | 0,077 | 0,0763 | 0,0799 | 0,0797 | 0,0819 | 0,0838 | 0,0848 | 0,0875 | 0,0893 | 0,0902 | 0,0917 | 0,0937 | 0,0957 | 0,0975 | 0,0995 | 0,1014 | 0,1043 | 0,1053 | 0,1075 | 0,1095 | 0,1113 | 0,1145 | 0,1156 | 0,1179 | 0,1194 | 0,1231 | 0,1242 | 0,1259 | 0,1277 | 0,1302 | 0,1315 | 0,1337 | 0,1359 | 0,138 | 0,1422 | 0,1423 | 0,1451 | 0,1462 | 0,1482 | 0,1502 | 0,1531 | 0,1543 | 0,1569 |
| E7 | 0,086 | 0,0883 | 0,0952 | 0,0971 | 0,0981 | 0,1032 | 0,1048 | 0,1081 | 0,1111 | 0,1134 | 0,1182 | 0,1211 | 0,123 | 0,1263 | 0,1293 | 0,1323 | 0,1358 | 0,1384 | 0,1416 | 0,1445 | 0,1479 | 0,1515 | 0,1542 | 0,1573 | 0,1605 | 0,1644 | 0,1671 | 0,17 | 0,1738 | 0,1775 | 0,1816 | 0,1841 | 0,1879 | 0,19 | 0,1938 | 0,1971 | 0,2004 | 0,2038 | 0,207 | 0,2107 | 0,214 | 0,2174 | 0,2201 | 0,2237 | 0,2269 | 0,231 |
| E8 | 0,0846 | 0,0896 | 0,0948 | 0,0978 | 0,0988 | 0,1032 | 0,1056 | 0,1086 | 0,1123 | 0,1144 | 0,1201 | 0,122 | 0,1244 | 0,1275 | 0,1307 | 0,1348 | 0,1372 | 0,1399 | 0,1434 | 0,1464 | 0,1494 | 0,1533 | 0,1565 | 0,1597 | 0,1631 | 0,1666 | 0,1698 | 0,1728 | 0,1765 | 0,1809 | 0,1837 | 0,1869 | 0,1905 | 0,1938 | 0,1972 | 0,2005 | 0,2039 | 0,2075 | 0,2109 | 0,2151 | 0,218 | 0,2211 | 0,2247 | 0,2284 | 0,2319 | 0,2355 |
| E9 | 0,0843 | 0,0908 | 0,0948 | 0,097 | 0,099 | 0,1037 | 0,1061 | 0,1092 | 0,113 | 0,1149 | 0,1201 | 0,1224 | 0,125 | 0,1284 | 0,1317 | 0,1347 | 0,1378 | 0,1412 | 0,1445 | 0,1477 | 0,1502 | 0,1543 | 0,1572 | 0,1603 | 0,1637 | 0,1671 | 0,1702 | 0,1733 | 0,1775 | 0,1813 | 0,184 | 0,1872 | 0,1911 | 0,1947 | 0,1979 | 0,2012 | 0,2048 | 0,2088 | 0,2118 | 0,2148 | 0,2182 | 0,2219 | 0,2259 | 0,2295 | 0,2328 | 0,2362 |
| E10 | 0,1028 | 0,1076 | 0,1149 | 0,1189 | 0,1208 | 0,1263 | 0,1296 | 0,133 | 0,1369 | 0,1405 | 0,1454 | 0,1483 | 0,1521 | 0,1564 | 0,1596 | 0,1631 | 0,167 | 0,1709 | 0,1749 | 0,1788 | 0,1833 | 0,1878 | 0,1923 | 0,1954 | 0,1999 | 0,2047 | 0,2084 | 0,2129 | 0,217 | 0,2225 | 0,2267 | 0,2303 | 0,2357 | 0,2393 | 0,2435 | 0,2479 | 0,2534 | 0,2567 | 0,2603 | 0,2648 | 0,2695 | 0,2746 | 0,2787 | 0,2824 | 0,2864 | 0,2911 |
| E11 | 0,1039 | 0,1083 | 0,1153 | 0,1183 | 0,1213 | 0,1264 | 0,1304 | 0,1339 | 0,1381 | 0,1418 | 0,1481 | 0,151 | 0,1545 | 0,1583 | 0,1622 | 0,1664 | 0,1709 | 0,1741 | 0,1781 | 0,182 | 0,1858 | 0,1906 | 0,1946 | 0,1983 | 0,2035 | 0,2072 | 0,2122 | 0,2165 | 0,2202 | 0,2254 | 0,2301 | 0,2341 | 0,2387 | 0,2426 | 0,2473 | 0,2515 | 0,256 | 0,2606 | 0,2644 | 0,2697 | 0,2742 | 0,2783 | 0,2827 | 0,2867 | 0,2909 | 0,2965 |
| E12 | 0,1014 | 0,1061 | 0,1106 | 0,1146 | 0,1191 | 0,1232 | 0,1269 | 0,1314 | 0,1358 | 0,1397 | 0,1439 | 0,148 | 0,1526 | 0,1563 | 0,1605 | 0,1643 | 0,1685 | 0,1728 | 0,1774 | 0,1812 | 0,1859 | 0,1904 | 0,1959 | 0,1998 | 0,2028 | 0,2089 | 0,2136 | 0,2165 | 0,2225 | 0,2275 | 0,2303 | 0,236 | 0,2407 | 0,2443 | 0,249 | 0,253 | 0,2598 | 0,2619 | 0,2685 | 0,2709 | 0,277 | 0,2801 | 0,2848 | 0,2888 | 0,2948 | 0,3003 |

End Time: 2020 09-30 14:22:40

**Raw data(triplicates) 5000nM WT PTPN22**

**Raw data(triplicates) 5000nM C129S PTPN22**

**1.5**

**1.0**

**0.5**

**0.0**


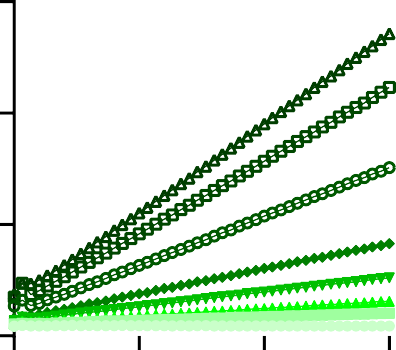


**0 5 10 15 20**

**Time(min)**

##### WT 0

WT 0.125mM WT 0.25mM WT 0.5mM WT 1mM

##### WT 2.5mM WT 5mM WT 7.5mM

**0.4**

**0.3**

**0.2**

**A405**

**0.1**

**0.0**


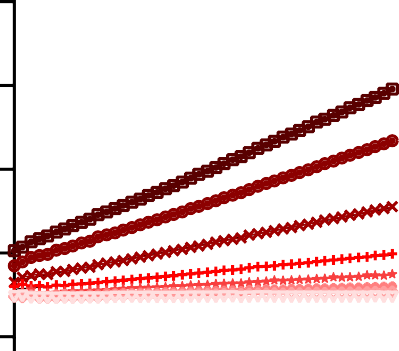


**0 5 10 15 20**

#### Time(min)

##### MUT 0


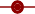

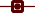
MUT 0.125mM MUT 0.25mM MUT 0.5mM MUT 1mM MUT 2.5mM MUT 5mM MUT 7.5mM

# wt

**0.10**


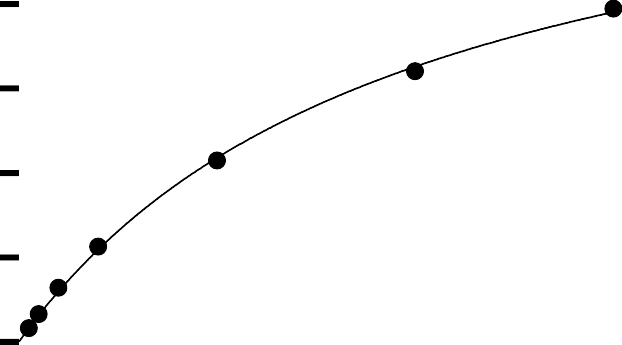


Km=4.91

**0.08**

**0.06**

**slop**

**0.015**

**0.010**


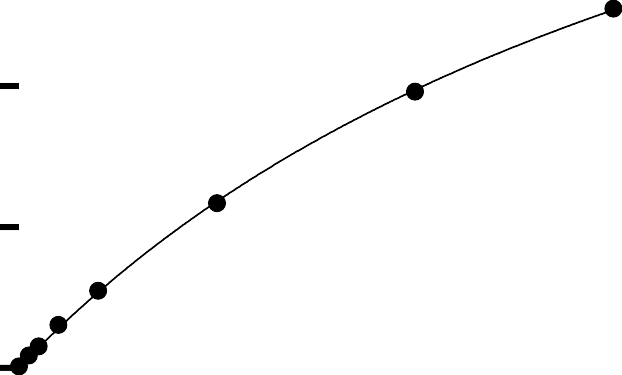


Km=10.14

**slop**

**mut**

**0.04**

**0.02**

**0.005**

**0.00**

**A405**

**0 2 4 6 8**

**mM(p-NPP)**

**0.000**

**0 2 4 6 8**

## mM(p-NPP)

| Application: Tecan i-control  Device: infinite 200Pro | | Tecan i-control , 2.0.10.0  Serial number: 1307001123 | Serial number of connected stacker: |
| --- | --- | --- | --- |
| Firmware: V_3.40_01/15_Infinite (Dec 23 201 MAI, V_3.40_01/15_Infinite (Dec 23 2014/12.45.11) | | | |
| Date: | ######### | | |
| Time: | 11:03:06 | | |

System MTC-MU059-S

User MTC-MU059-S\fretho

Plate Thermo Fisher Scientific-Nunclon 96 Flat Bottom Transparent Polystyrene Catalog No.: 269620/269787/439454/442404/475094 [NUN96ft.pdfx] Plate-ID (Stacker)

List of actions in this measurement script: Kinetic

Shaking (Linear) Duration: Shaking (Linear) Amplitude: Absorbance

2 s

1 mm

Label: Label1

Kinetic Measurement

Kinetic duration 00:15:00

Interval Time 00:00:20

Measurement Wavelength 405 nm

Bandwidth 10 nm

Number of Flashes 5

Settle Time 0 ms

Part of Plate A1-D12

Start Time: 2020 10-06 11:03:08

Cycle Nr.

1

2

3

4

5

6

7

8

9

10 11 12 13 14 15 16 17 18 19 20 21 22 23 24 25 26 27 28 29 30 31 32 33 34 35 36 37 38 39 40 41 42 43 44 45 46

|  |  |  |  |  |  |  |  |  |  |  |  |  |  |  |  |  |  |  |  |  |  |  |  |  |  |  |  |  |  |  |  |  |  |  |  |  |  |  |  |  |  |  |  |  |  |  |
| --- | --- | --- | --- | --- | --- | --- | --- | --- | --- | --- | --- | --- | --- | --- | --- | --- | --- | --- | --- | --- | --- | --- | --- | --- | --- | --- | --- | --- | --- | --- | --- | --- | --- | --- | --- | --- | --- | --- | --- | --- | --- | --- | --- | --- | --- | --- |
| Time [s] | 0 | 20 | 40 | 60 | 80 | 100 | 120 | 140 | 160 | 180 | 200 | 220 | 240 | 260 | 280 | 300 | 320 | 340 | 360 | 380 | 400 | 420 | 440 | 460 | 480 | 500 | 520 | 540 | 560 | 580 | 600 | 620 | 640 | 660 | 680 | 700 | 720 | 740 | 760 | 780 | 800 | 820 | 840 | 860 | 880 | 900 |
| Temp. [°C] | 23,5 | 23,6 | 23,5 | 23,5 | 23,8 | 23,9 | 23,9 | 23,8 | 23,6 | 23,5 | 24 | 23,7 | 23,7 | 23,7 | 23,8 | 24,1 | 24 | 24 | 24,2 | 24,2 | 24,2 | 23,9 | 24,3 | 24,1 | 24,3 | 24 | 24,1 | 24 | 24,3 | 24,3 | 24 | 24,3 | 24,3 | 24,4 | 24,2 | 24,3 | 24,2 | 24,2 | 24,5 | 24 | 24,4 | 24,2 | 24,3 | 24,3 | 24,5 | 24,4 |
| A1 | 0,1562 | 0,1882 | 0,2063 | 0,2195 | 0,2399 | 0,2551 | 0,2664 | 0,2809 | 0,2937 | 0,3063 | 0,3214 | 0,3349 | 0,3487 | 0,3631 | 0,3804 | 0,3914 | 0,4025 | 0,4191 | 0,4321 | 0,4434 | 0,4594 | 0,4719 | 0,4852 | 0,4983 | 0,5127 | 0,5273 | 0,5408 | 0,5528 | 0,5651 | 0,5789 | 0,5906 | 0,6064 | 0,6175 | 0,6343 | 0,6467 | 0,6594 | 0,6721 | 0,6871 | 0,7004 | 0,7142 | 0,7262 | 0,7391 | 0,7526 | 0,7658 | 0,779 | 0,7933 |
| A2 | 0,1546 | 0,1935 | 0,2114 | 0,2258 | 0,2408 | 0,2573 | 0,2721 | 0,2875 | 0,3022 | 0,3166 | 0,3343 | 0,3466 | 0,3644 | 0,3761 | 0,3914 | 0,4035 | 0,4184 | 0,4326 | 0,45 | 0,4619 | 0,4793 | 0,4914 | 0,5059 | 0,5199 | 0,5337 | 0,5488 | 0,563 | 0,5777 | 0,5893 | 0,603 | 0,6183 | 0,6332 | 0,6474 | 0,6601 | 0,6771 | 0,694 | 0,7012 | 0,7159 | 0,73 | 0,7445 | 0,7585 | 0,771 | 0,7848 | 0,7985 | 0,8128 | 0,8281 |
| A3 | 0,1579 | 0,1973 | 0,2191 | 0,2357 | 0,2532 | 0,2679 | 0,2852 | 0,3016 | 0,3182 | 0,3323 | 0,3496 | 0,3633 | 0,3809 | 0,3969 | 0,414 | 0,4295 | 0,4429 | 0,4599 | 0,4766 | 0,4887 | 0,5078 | 0,524 | 0,5376 | 0,5535 | 0,5685 | 0,5871 | 0,6007 | 0,617 | 0,6308 | 0,6462 | 0,6614 | 0,6784 | 0,6931 | 0,708 | 0,7241 | 0,7386 | 0,7528 | 0,77 | 0,7856 | 0,8028 | 0,816 | 0,8322 | 0,8468 | 0,8619 | 0,8767 | 0,8922 |
| A4 | 0,1351 | 0,1632 | 0,1774 | 0,1918 | 0,2046 | 0,218 | 0,2325 | 0,2448 | 0,2588 | 0,271 | 0,2853 | 0,2973 | 0,3116 | 0,3245 | 0,3374 | 0,3518 | 0,3646 | 0,3771 | 0,3903 | 0,4013 | 0,4177 | 0,4294 | 0,4432 | 0,4573 | 0,4683 | 0,4819 | 0,4971 | 0,5089 | 0,5197 | 0,5321 | 0,5458 | 0,5605 | 0,5727 | 0,5844 | 0,599 | 0,6118 | 0,6225 | 0,6376 | 0,649 | 0,6625 | 0,675 | 0,686 | 0,6999 | 0,7125 | 0,7263 | 0,7382 |
| A5 | 0,1348 | 0,1597 | 0,1756 | 0,1872 | 0,1999 | 0,214 | 0,2272 | 0,2389 | 0,2519 | 0,2632 | 0,2768 | 0,2873 | 0,3018 | 0,3137 | 0,3268 | 0,3387 | 0,3493 | 0,3631 | 0,378 | 0,3884 | 0,4022 | 0,4135 | 0,4257 | 0,4385 | 0,4505 | 0,4624 | 0,4771 | 0,4889 | 0,4979 | 0,5118 | 0,5257 | 0,5384 | 0,5491 | 0,5602 | 0,5744 | 0,5855 | 0,5972 | 0,6092 | 0,6213 | 0,6356 | 0,6459 | 0,6572 | 0,6703 | 0,683 | 0,6936 | 0,7062 |
| A6 | 0,1328 | 0,1545 | 0,1672 | 0,1768 | 0,1905 | 0,2027 | 0,2149 | 0,2266 | 0,2374 | 0,2498 | 0,2613 | 0,2727 | 0,2849 | 0,2962 | 0,308 | 0,3181 | 0,3299 | 0,3409 | 0,3537 | 0,363 | 0,3798 | 0,3872 | 0,3982 | 0,4086 | 0,421 | 0,4322 | 0,4447 | 0,4562 | 0,466 | 0,4784 | 0,4898 | 0,5033 | 0,5129 | 0,524 | 0,536 | 0,5471 | 0,5567 | 0,5686 | 0,5797 | 0,5923 | 0,6025 | 0,6129 | 0,6244 | 0,6356 | 0,6481 | 0,6589 |
| A7 | 0,1032 | 0,1172 | 0,1256 | 0,1348 | 0,1477 | 0,1529 | 0,1624 | 0,1713 | 0,1804 | 0,1877 | 0,197 | 0,2066 | 0,221 | 0,2251 | 0,2393 | 0,2413 | 0,2483 | 0,2581 | 0,2695 | 0,2753 | 0,2859 | 0,2922 | 0,3037 | 0,3105 | 0,3197 | 0,33 | 0,3383 | 0,3466 | 0,353 | 0,3609 | 0,3712 | 0,3828 | 0,3892 | 0,397 | 0,4061 | 0,4151 | 0,4228 | 0,4311 | 0,4401 | 0,4515 | 0,4569 | 0,4648 | 0,4724 | 0,4817 | 0,4894 | 0,4971 |
| A8 | 0,1044 | 0,1173 | 0,1271 | 0,1343 | 0,1443 | 0,1534 | 0,1626 | 0,1721 | 0,1814 | 0,1893 | 0,1981 | 0,207 | 0,2164 | 0,2263 | 0,2348 | 0,2429 | 0,2519 | 0,26 | 0,269 | 0,2756 | 0,2867 | 0,2947 | 0,3036 | 0,3116 | 0,3216 | 0,3309 | 0,3397 | 0,349 | 0,3552 | 0,3616 | 0,3697 | 0,3816 | 0,3902 | 0,3969 | 0,4078 | 0,4145 | 0,4217 | 0,4307 | 0,4403 | 0,4509 | 0,4567 | 0,4648 | 0,4733 | 0,4818 | 0,4909 | 0,4996 |
| A9 | 0,1038 | 0,1148 | 0,1226 | 0,129 | 0,1386 | 0,1472 | 0,1566 | 0,1652 | 0,1739 | 0,1813 | 0,1896 | 0,1974 | 0,2065 | 0,2137 | 0,2232 | 0,2322 | 0,2394 | 0,2492 | 0,2564 | 0,2618 | 0,2723 | 0,279 | 0,2881 | 0,2959 | 0,3057 | 0,3129 | 0,3215 | 0,3302 | 0,3357 | 0,3444 | 0,3524 | 0,3629 | 0,3697 | 0,3752 | 0,3867 | 0,3946 | 0,3998 | 0,4069 | 0,4166 | 0,4255 | 0,4321 | 0,439 | 0,4472 | 0,455 | 0,4623 | 0,4697 |
| A10 | 0,0698 | 0,0735 | 0,0769 | 0,0805 | 0,0876 | 0,092 | 0,096 | 0,1002 | 0,1057 | 0,1058 | 0,1118 | 0,1165 | 0,1205 | 0,1259 | 0,1298 | 0,133 | 0,1349 | 0,141 | 0,1447 | 0,1478 | 0,1543 | 0,1572 | 0,1608 | 0,1638 | 0,1686 | 0,1723 | 0,1769 | 0,1817 | 0,1846 | 0,1884 | 0,1921 | 0,1974 | 0,2015 | 0,2047 | 0,2092 | 0,213 | 0,2149 | 0,2198 | 0,2243 | 0,2291 | 0,2318 | 0,2353 | 0,2388 | 0,242 | 0,2469 | 0,2507 |
| A11 | 0,0694 | 0,0758 | 0,0782 | 0,0826 | 0,0872 | 0,0922 | 0,098 | 0,1009 | 0,1055 | 0,1078 | 0,1145 | 0,118 | 0,1227 | 0,1262 | 0,1317 | 0,1342 | 0,1375 | 0,1427 | 0,1487 | 0,1499 | 0,1576 | 0,1589 | 0,1647 | 0,1684 | 0,1728 | 0,1745 | 0,181 | 0,185 | 0,1879 | 0,1914 | 0,1945 | 0,2013 | 0,2056 | 0,2098 | 0,2142 | 0,217 | 0,2205 | 0,2235 | 0,2282 | 0,2332 | 0,2375 | 0,2415 | 0,2442 | 0,2481 | 0,2519 | 0,2556 |
| A12 | 0,0698 | 0,0742 | 0,0761 | 0,0803 | 0,0849 | 0,0903 | 0,0946 | 0,0986 | 0,1024 | 0,105 | 0,1105 | 0,1135 | 0,1188 | 0,1225 | 0,1276 | 0,1306 | 0,1329 | 0,1378 | 0,1436 | 0,1438 | 0,151 | 0,1543 | 0,1578 | 0,1614 | 0,1697 | 0,1698 | 0,1743 | 0,178 | 0,1791 | 0,1849 | 0,187 | 0,1947 | 0,1952 | 0,1999 | 0,2051 | 0,2088 | 0,2091 | 0,2152 | 0,219 | 0,2246 | 0,2276 | 0,2291 | 0,2338 | 0,2373 | 0,2423 | 0,2469 |
| B1 | 0,0561 | 0,0792 | 0,0787 | 0,0776 | 0,0742 | 0,0702 | 0,073 | 0,0726 | 0,0751 | 0,0757 | 0,08 | 0,0806 | 0,0831 | 0,0858 | 0,0891 | 0,0898 | 0,0929 | 0,0949 | 0,0993 | 0,1001 | 0,1048 | 0,1049 | 0,1078 | 0,1096 | 0,1124 | 0,1175 | 0,117 | 0,1198 | 0,1205 | 0,1228 | 0,124 | 0,1299 | 0,1313 | 0,1325 | 0,1366 | 0,1384 | 0,1391 | 0,1416 | 0,1443 | 0,1478 | 0,1488 | 0,1495 | 0,1531 | 0,1544 | 0,1569 | 0,1595 |
| B2 | 0,0676 | 0,0673 | 0,0697 | 0,0674 | 0,0695 | 0,0726 | 0,075 | 0,0747 | 0,0771 | 0,0766 | 0,0798 | 0,0811 | 0,0835 | 0,0854 | 0,0887 | 0,0922 | 0,0914 | 0,096 | 0,0999 | 0,0988 | 0,1034 | 0,1051 | 0,1065 | 0,109 | 0,1152 | 0,116 | 0,1201 | 0,12 | 0,1201 | 0,1223 | 0,1233 | 0,1306 | 0,1301 | 0,1333 | 0,1361 | 0,1383 | 0,1429 | 0,1433 | 0,1434 | 0,1474 | 0,1485 | 0,1508 | 0,1519 | 0,1535 | 0,1558 | 0,1589 |
| B3 | 0,056 | 0,058 | 0,0579 | 0,0618 | 0,0638 | 0,0671 | 0,0699 | 0,0732 | 0,0757 | 0,0745 | 0,0794 | 0,0805 | 0,083 | 0,0866 | 0,0896 | 0,0909 | 0,0902 | 0,095 | 0,0983 | 0,0969 | 0,1018 | 0,1033 | 0,1068 | 0,1082 | 0,1112 | 0,1124 | 0,1159 | 0,1169 | 0,1174 | 0,1195 | 0,1226 | 0,1269 | 0,1281 | 0,1307 | 0,134 | 0,1341 | 0,1339 | 0,1368 | 0,14 | 0,1447 | 0,146 | 0,1464 | 0,1495 | 0,1504 | 0,1547 | 0,1557 |
| B4 | 0,053 | 0,0559 | 0,0533 | 0,0528 | 0,0551 | 0,0567 | 0,0577 | 0,0577 | 0,0599 | 0,0603 | 0,0617 | 0,0616 | 0,0643 | 0,0658 | 0,0682 | 0,0682 | 0,068 | 0,0706 | 0,0735 | 0,0736 | 0,0747 | 0,0738 | 0,0758 | 0,0775 | 0,0772 | 0,0817 | 0,0811 | 0,0825 | 0,0827 | 0,083 | 0,0868 | 0,0879 | 0,0892 | 0,089 | 0,0912 | 0,0914 | 0,0899 | 0,0925 | 0,0942 | 0,0967 | 0,0977 | 0,0979 | 0,101 | 0,0985 | 0,1011 | 0,1035 |
| B5 | 0,0508 | 0,054 | 0,058 | 0,0576 | 0,0563 | 0,0566 | 0,0568 | 0,0597 | 0,0608 | 0,0605 | 0,0623 | 0,0623 | 0,0654 | 0,0661 | 0,0678 | 0,0665 | 0,0676 | 0,0694 | 0,0734 | 0,0719 | 0,0749 | 0,0747 | 0,0765 | 0,0755 | 0,0799 | 0,0812 | 0,0838 | 0,0833 | 0,0838 | 0,0849 | 0,0865 | 0,0899 | 0,0909 | 0,0891 | 0,0914 | 0,0925 | 0,0919 | 0,0945 | 0,095 | 0,0981 | 0,0995 | 0,0992 | 0,1023 | 0,1023 | 0,1021 | 0,104 |
| B6 | 0,0506 | 0,0493 | 0,0516 | 0,0547 | 0,0546 | 0,0566 | 0,0583 | 0,0588 | 0,0601 | 0,0593 | 0,0626 | 0,0619 | 0,0647 | 0,0677 | 0,0681 | 0,0692 | 0,0687 | 0,07 | 0,0724 | 0,0725 | 0,0746 | 0,0755 | 0,0763 | 0,0767 | 0,0786 | 0,0826 | 0,0801 | 0,0842 | 0,0833 | 0,0841 | 0,0865 | 0,0893 | 0,0902 | 0,0894 | 0,093 | 0,0917 | 0,0922 | 0,094 | 0,0956 | 0,0987 | 0,0989 | 0,099 | 0,1001 | 0,1011 | 0,102 | 0,1037 |
| B7 | 0,0471 | 0,046 | 0,0476 | 0,0459 | 0,0485 | 0,0486 | 0,0503 | 0,0506 | 0,0524 | 0,0513 | 0,0521 | 0,0519 | 0,0533 | 0,054 | 0,0557 | 0,0555 | 0,0527 | 0,0559 | 0,0578 | 0,0544 | 0,0582 | 0,0579 | 0,0601 | 0,0597 | 0,0608 | 0,0617 | 0,0628 | 0,064 | 0,0623 | 0,0599 | 0,0618 | 0,0642 | 0,0661 | 0,066 | 0,0687 | 0,0666 | 0,0673 | 0,068 | 0,0692 | 0,0712 | 0,0713 | 0,07 | 0,0704 | 0,071 | 0,0725 | 0,0736 |
| B8 | 0,0468 | 0,0459 | 0,0475 | 0,0469 | 0,0475 | 0,0502 | 0,0501 | 0,0513 | 0,0517 | 0,0499 | 0,0507 | 0,0521 | 0,0537 | 0,0541 | 0,0552 | 0,0545 | 0,0549 | 0,0546 | 0,0569 | 0,0564 | 0,0596 | 0,0595 | 0,059 | 0,0612 | 0,0591 | 0,0602 | 0,0629 | 0,0626 | 0,062 | 0,0627 | 0,0631 | 0,0664 | 0,064 | 0,0648 | 0,0673 | 0,0674 | 0,0662 | 0,0677 | 0,0681 | 0,0697 | 0,07 | 0,07 | 0,0708 | 0,0715 | 0,0721 | 0,0731 |
| B9 | 0,0463 | 0,0452 | 0,0451 | 0,0449 | 0,048 | 0,0481 | 0,0482 | 0,0491 | 0,0503 | 0,05 | 0,0526 | 0,0494 | 0,0531 | 0,0526 | 0,0541 | 0,0543 | 0,053 | 0,0559 | 0,0573 | 0,057 | 0,0577 | 0,0561 | 0,0582 | 0,0587 | 0,0587 | 0,0599 | 0,0606 | 0,0605 | 0,0599 | 0,0607 | 0,0608 | 0,0641 | 0,0656 | 0,0655 | 0,0653 | 0,0667 | 0,0651 | 0,0661 | 0,0664 | 0,0694 | 0,0698 | 0,0685 | 0,0699 | 0,07 | 0,0713 | 0,0703 |
| B10 | 0,0413 | 0,0397 | 0,0395 | 0,0394 | 0,0397 | 0,04 | 0,0401 | 0,0413 | 0,042 | 0,0409 | 0,0407 | 0,0412 | 0,0402 | 0,0404 | 0,0402 | 0,0417 | 0,0392 | 0,04 | 0,042 | 0,0399 | 0,0427 | 0,0394 | 0,0415 | 0,04 | 0,0413 | 0,0409 | 0,0415 | 0,0418 | 0,0408 | 0,0403 | 0,0408 | 0,0417 | 0,0406 | 0,0404 | 0,0401 | 0,0411 | 0,0404 | 0,0405 | 0,04 | 0,0422 | 0,0415 | 0,0408 | 0,0409 | 0,0399 | 0,0403 | 0,0413 |
| B11 | 0,0414 | 0,0402 | 0,0409 | 0,0389 | 0,0409 | 0,0405 | 0,042 | 0,0405 | 0,0413 | 0,0388 | 0,0422 | 0,0392 | 0,0407 | 0,0423 | 0,0399 | 0,0394 | 0,0414 | 0,0407 | 0,0412 | 0,0396 | 0,0427 | 0,0408 | 0,041 | 0,0411 | 0,0406 | 0,0415 | 0,0413 | 0,0406 | 0,0384 | 0,0397 | 0,0401 | 0,0423 | 0,0425 | 0,0398 | 0,0402 | 0,0414 | 0,0408 | 0,0401 | 0,0402 | 0,0414 | 0,0404 | 0,0395 | 0,0412 | 0,0408 | 0,0394 | 0,0411 |
| B12 | 0,0417 | 0,0393 | 0,0388 | 0,0385 | 0,0404 | 0,0416 | 0,041 | 0,0401 | 0,0408 | 0,0385 | 0,0416 | 0,0393 | 0,0401 | 0,0402 | 0,0411 | 0,0404 | 0,0384 | 0,0387 | 0,0416 | 0,0384 | 0,0407 | 0,0395 | 0,0401 | 0,0396 | 0,0396 | 0,0412 | 0,0413 | 0,0402 | 0,0389 | 0,0371 | 0,0375 | 0,0419 | 0,0419 | 0,0401 | 0,0401 | 0,04 | 0,0389 | 0,0401 | 0,0395 | 0,041 | 0,0401 | 0,0389 | 0,0414 | 0,0397 | 0,0399 | 0,0412 |
| C1 | 0,0969 | 0,1 | 0,0998 | 0,1034 | 0,1078 | 0,1111 | 0,1139 | 0,117 | 0,1194 | 0,1202 | 0,1251 | 0,1244 | 0,1283 | 0,1333 | 0,135 | 0,1368 | 0,1391 | 0,1413 | 0,1451 | 0,1467 | 0,151 | 0,1511 | 0,1541 | 0,1577 | 0,1604 | 0,1647 | 0,1667 | 0,1697 | 0,1696 | 0,1716 | 0,1752 | 0,1796 | 0,182 | 0,1829 | 0,1872 | 0,1906 | 0,1916 | 0,194 | 0,1975 | 0,2014 | 0,2036 | 0,2036 | 0,207 | 0,21 | 0,2123 | 0,2162 |
| C2 | 0,0963 | 0,0989 | 0,1014 | 0,1045 | 0,1072 | 0,1114 | 0,1153 | 0,1182 | 0,1216 | 0,1237 | 0,1256 | 0,1288 | 0,1334 | 0,1342 | 0,1371 | 0,1386 | 0,1431 | 0,1452 | 0,1475 | 0,1503 | 0,1541 | 0,1556 | 0,1573 | 0,161 | 0,1626 | 0,1661 | 0,1713 | 0,1714 | 0,1722 | 0,1756 | 0,1773 | 0,1831 | 0,1857 | 0,1878 | 0,1932 | 0,1942 | 0,1948 | 0,1994 | 0,2017 | 0,2062 | 0,209 | 0,2092 | 0,2134 | 0,2143 | 0,2172 | 0,2221 |
| C3 | 0,0962 | 0,0984 | 0,1019 | 0,1042 | 0,1098 | 0,1123 | 0,1151 | 0,1194 | 0,1232 | 0,1231 | 0,1271 | 0,1286 | 0,1334 | 0,1345 | 0,1386 | 0,1383 | 0,1403 | 0,143 | 0,1465 | 0,1472 | 0,1545 | 0,1536 | 0,157 | 0,1605 | 0,1618 | 0,1665 | 0,1686 | 0,1722 | 0,1731 | 0,1762 | 0,1792 | 0,1836 | 0,1849 | 0,187 | 0,1923 | 0,1932 | 0,1952 | 0,1986 | 0,2017 | 0,2065 | 0,2069 | 0,21 | 0,2136 | 0,2146 | 0,218 | 0,2217 |
| C4 | 0,078 | 0,0793 | 0,0814 | 0,0833 | 0,0864 | 0,0894 | 0,0927 | 0,0956 | 0,0969 | 0,1013 | 0,1027 | 0,1029 | 0,1075 | 0,1087 | 0,1106 | 0,1125 | 0,1152 | 0,1156 | 0,1204 | 0,1204 | 0,1235 | 0,1271 | 0,1284 | 0,128 | 0,1323 | 0,1322 | 0,1368 | 0,1389 | 0,1401 | 0,1388 | 0,1419 | 0,1477 | 0,148 | 0,1492 | 0,153 | 0,1546 | 0,1548 | 0,1587 | 0,1608 | 0,1643 | 0,1659 | 0,167 | 0,17 | 0,1723 | 0,1746 | 0,177 |
| C5 | 0,078 | 0,0798 | 0,0831 | 0,0834 | 0,0881 | 0,0911 | 0,0937 | 0,0953 | 0,0987 | 0,1001 | 0,1026 | 0,1024 | 0,1061 | 0,1073 | 0,1113 | 0,1099 | 0,1122 | 0,1139 | 0,1174 | 0,1172 | 0,1227 | 0,1253 | 0,1265 | 0,1277 | 0,1303 | 0,132 | 0,1337 | 0,1353 | 0,1372 | 0,1388 | 0,1432 | 0,1467 | 0,1464 | 0,1486 | 0,1516 | 0,1534 | 0,1538 | 0,1564 | 0,1597 | 0,1626 | 0,1649 | 0,1649 | 0,1668 | 0,1695 | 0,1723 | 0,176 |
| C6 | 0,0786 | 0,0802 | 0,0817 | 0,0847 | 0,088 | 0,0891 | 0,0939 | 0,0946 | 0,0975 | 0,0991 | 0,1008 | 0,1 | 0,1034 | 0,1054 | 0,1065 | 0,1078 | 0,1105 | 0,1113 | 0,115 | 0,1152 | 0,1198 | 0,1192 | 0,1225 | 0,1239 | 0,1253 | 0,129 | 0,1305 | 0,1332 | 0,1343 | 0,1345 | 0,1536 | 0,142 | 0,1433 | 0,1449 | 0,1483 | 0,1492 | 0,1496 | 0,1528 | 0,1556 | 0,1602 | 0,1617 | 0,1616 | 0,1644 | 0,1794 | 0,1691 | 0,1701 |
| C7 | 0,0629 | 0,0618 | 0,0636 | 0,0656 | 0,0659 | 0,0687 | 0,0701 | 0,0725 | 0,0738 | 0,0749 | 0,0761 | 0,0756 | 0,0798 | 0,0798 | 0,0817 | 0,0814 | 0,0835 | 0,0828 | 0,0875 | 0,0854 | 0,0879 | 0,089 | 0,0916 | 0,0907 | 0,0914 | 0,095 | 0,0956 | 0,0967 | 0,0958 | 0,0967 | 0,0982 | 0,1009 | 0,1014 | 0,1038 | 0,105 | 0,1053 | 0,1068 | 0,1077 | 0,1094 | 0,1123 | 0,1137 | 0,1122 | 0,1154 | 0,1149 | 0,1172 | 0,1188 |
| C8 | 0,0639 | 0,0615 | 0,0622 | 0,0642 | 0,0669 | 0,072 | 0,0698 | 0,0721 | 0,0737 | 0,0739 | 0,0746 | 0,0767 | 0,0787 | 0,0784 | 0,082 | 0,0808 | 0,0823 | 0,0882 | 0,0863 | 0,085 | 0,0889 | 0,0882 | 0,0899 | 0,0901 | 0,0934 | 0,095 | 0,0961 | 0,097 | 0,097 | 0,0982 | 0,1 | 0,1024 | 0,1012 | 0,103 | 0,1061 | 0,1062 | 0,1052 | 0,1088 | 0,1095 | 0,1124 | 0,1126 | 0,1139 | 0,1157 | 0,1159 | 0,1182 | 0,1202 |
| C9 | 0,0615 | 0,0617 | 0,061 | 0,0627 | 0,0656 | 0,0672 | 0,068 | 0,0701 | 0,0713 | 0,0722 | 0,0729 | 0,0728 | 0,0762 | 0,0773 | 0,0779 | 0,0793 | 0,0771 | 0,0805 | 0,0836 | 0,0813 | 0,0849 | 0,0856 | 0,0862 | 0,0878 | 0,0879 | 0,0891 | 0,0912 | 0,0935 | 0,0921 | 0,094 | 0,0938 | 0,099 | 0,0988 | 0,0992 | 0,1012 | 0,1032 | 0,1019 | 0,1035 | 0,1057 | 0,108 | 0,1087 | 0,1086 | 0,1107 | 0,1104 | 0,114 | 0,1145 |
| C10 | 0,0508 | 0,0493 | 0,0506 | 0,0504 | 0,0506 | 0,0519 | 0,0538 | 0,0539 | 0,054 | 0,0549 | 0,056 | 0,0565 | 0,058 | 0,057 | 0,0577 | 0,0581 | 0,0575 | 0,0584 | 0,0602 | 0,0583 | 0,0631 | 0,0614 | 0,0626 | 0,0633 | 0,0645 | 0,0647 | 0,0656 | 0,0634 | 0,0632 | 0,0638 | 0,0669 | 0,0693 | 0,0684 | 0,0679 | 0,0706 | 0,0704 | 0,0691 | 0,0711 | 0,0715 | 0,0725 | 0,0722 | 0,072 | 0,0734 | 0,0745 | 0,0746 | 0,0755 |
| C11 | 0,0503 | 0,0498 | 0,0493 | 0,0482 | 0,0487 | 0,0511 | 0,0524 | 0,0536 | 0,0528 | 0,0539 | 0,0542 | 0,0534 | 0,0559 | 0,0546 | 0,0556 | 0,056 | 0,0539 | 0,0564 | 0,058 | 0,0549 | 0,0589 | 0,0606 | 0,0595 | 0,0588 | 0,059 | 0,0614 | 0,0612 | 0,0613 | 0,061 | 0,0607 | 0,0608 | 0,0641 | 0,0651 | 0,0629 | 0,0643 | 0,0636 | 0,0629 | 0,0644 | 0,0649 | 0,0677 | 0,0662 | 0,0667 | 0,0682 | 0,0679 | 0,069 | 0,0704 |
| C12 | 0,0499 | 0,0493 | 0,0504 | 0,0499 | 0,0509 | 0,0508 | 0,0544 | 0,0536 | 0,0526 | 0,0545 | 0,0546 | 0,0535 | 0,0553 | 0,0574 | 0,0571 | 0,0569 | 0,0563 | 0,058 | 0,0588 | 0,0588 | 0,0606 | 0,0596 | 0,062 | 0,0597 | 0,0604 | 0,0629 | 0,0633 | 0,0629 | 0,0627 | 0,0626 | 0,0626 | 0,0653 | 0,0649 | 0,0651 | 0,0667 | 0,0646 | 0,0658 | 0,0663 | 0,0673 | 0,0707 | 0,0683 | 0,0673 | 0,0691 | 0,0691 | 0,0716 | 0,0709 |
| D1 | 0,0469 | 0,049 | 0,0451 | 0,0446 | 0,0498 | 0,0515 | 0,0548 | 0,0498 | 0,0531 | 0,053 | 0,0487 | 0,0485 | 0,0492 | 0,0518 | 0,052 | 0,0515 | 0,0512 | 0,0564 | 0,0515 | 0,0497 | 0,0527 | 0,0522 | 0,0537 | 0,0555 | 0,0535 | 0,0546 | 0,0548 | 0,0548 | 0,0565 | 0,0551 | 0,0528 | 0,0587 | 0,0589 | 0,0559 | 0,0693 | 0,0571 | 0,0564 | 0,0574 | 0,0574 | 0,0636 | 0,0689 | 0,06 | 0,0714 | 0,0595 | 0,0616 | 0,0609 |
| D2 | 0,0475 | 0,0459 | 0,049 | 0,0461 | 0,0465 | 0,048 | 0,0488 | 0,0512 | 0,0504 | 0,0522 | 0,0522 | 0,0502 | 0,0529 | 0,053 | 0,0524 | 0,053 | 0,0529 | 0,0534 | 0,0549 | 0,0528 | 0,0555 | 0,0535 | 0,0543 | 0,0564 | 0,0556 | 0,0564 | 0,0552 | 0,0561 | 0,0549 | 0,0548 | 0,0541 | 0,0589 | 0,0575 | 0,0557 | 0,0578 | 0,058 | 0,0558 | 0,0582 | 0,0583 | 0,0609 | 0,06 | 0,0585 | 0,0601 | 0,0594 | 0,0615 | 0,0619 |
| D3 | 0,0482 | 0,046 | 0,0474 | 0,0454 | 0,047 | 0,0478 | 0,047 | 0,0488 | 0,0477 | 0,0476 | 0,0492 | 0,0482 | 0,0509 | 0,0499 | 0,0502 | 0,0514 | 0,0496 | 0,0496 | 0,0515 | 0,0491 | 0,0536 | 0,0521 | 0,0533 | 0,0524 | 0,053 | 0,0583 | 0,0552 | 0,0549 | 0,0549 | 0,0528 | 0,0538 | 0,057 | 0,0579 | 0,0566 | 0,0581 | 0,0597 | 0,0555 | 0,0568 | 0,0577 | 0,059 | 0,0594 | 0,058 | 0,0598 | 0,0582 | 0,0606 | 0,061 |
| D4 | 0,0439 | 0,0422 | 0,0431 | 0,042 | 0,0428 | 0,0437 | 0,0439 | 0,0448 | 0,0447 | 0,044 | 0,044 | 0,0438 | 0,0463 | 0,0459 | 0,0465 | 0,0459 | 0,045 | 0,0467 | 0,0481 | 0,0465 | 0,0478 | 0,046 | 0,0472 | 0,0477 | 0,0468 | 0,049 | 0,0487 | 0,0481 | 0,0479 | 0,0474 | 0,0469 | 0,0476 | 0,0488 | 0,0482 | 0,0503 | 0,0485 | 0,0483 | 0,0485 | 0,0495 | 0,0516 | 0,0511 | 0,05 | 0,0517 | 0,0505 | 0,0517 | 0,0513 |
| D5 | 0,0462 | 0,0436 | 0,0454 | 0,044 | 0,0439 | 0,0454 | 0,0465 | 0,0445 | 0,0454 | 0,0449 | 0,0466 | 0,0458 | 0,0477 | 0,0457 | 0,0468 | 0,0463 | 0,0465 | 0,0469 | 0,0487 | 0,0451 | 0,05 | 0,0476 | 0,0491 | 0,048 | 0,0478 | 0,0489 | 0,0493 | 0,0501 | 0,0476 | 0,0483 | 0,0478 | 0,0513 | 0,0491 | 0,0485 | 0,0511 | 0,0513 | 0,0504 | 0,0499 | 0,0506 | 0,0517 | 0,0516 | 0,051 | 0,0515 | 0,0516 | 0,0509 | 0,054 |
| D6 | 0,0455 | 0,0433 | 0,046 | 0,0433 | 0,0453 | 0,0453 | 0,0452 | 0,0472 | 0,046 | 0,0456 | 0,0448 | 0,0447 | 0,0461 | 0,0464 | 0,0471 | 0,0463 | 0,0553 | 0,0468 | 0,0472 | 0,0442 | 0,0473 | 0,048 | 0,0471 | 0,0484 | 0,0485 | 0,0474 | 0,0493 | 0,0486 | 0,0477 | 0,048 | 0,0497 | 0,0495 | 0,05 | 0,0504 | 0,0511 | 0,0502 | 0,0475 | 0,0496 | 0,0503 | 0,0514 | 0,0505 | 0,0503 | 0,0565 | 0,0509 | 0,0513 | 0,0522 |
| D7 | 0,0449 | 0,0434 | 0,0436 | 0,0423 | 0,0449 | 0,0463 | 0,0447 | 0,0437 | 0,0452 | 0,0449 | 0,0436 | 0,0437 | 0,0453 | 0,0449 | 0,0464 | 0,0465 | 0,0465 | 0,0475 | 0,0513 | 0,0506 | 0,0449 | 0,0445 | 0,0456 | 0,0442 | 0,0431 | 0,045 | 0,0492 | 0,0471 | 0,0444 | 0,0502 | 0,0443 | 0,0473 | 0,0464 | 0,0445 | 0,0484 | 0,0455 | 0,0444 | 0,0465 | 0,046 | 0,0457 | 0,0476 | 0,0465 | 0,0458 | 0,0462 | 0,0472 | 0,0468 |
| D8 | 0,0429 | 0,0424 | 0,0428 | 0,0426 | 0,0413 | 0,0433 | 0,0443 | 0,0436 | 0,0439 | 0,0462 | 0,045 | 0,0432 | 0,0459 | 0,0434 | 0,0456 | 0,0446 | 0,0432 | 0,0438 | 0,0452 | 0,0438 | 0,0451 | 0,0443 | 0,0446 | 0,047 | 0,0445 | 0,0449 | 0,0465 | 0,0454 | 0,0439 | 0,0434 | 0,0448 | 0,0467 | 0,0461 | 0,0453 | 0,0472 | 0,0463 | 0,0445 | 0,0454 | 0,0456 | 0,0466 | 0,0468 | 0,046 | 0,0456 | 0,0482 | 0,0488 | 0,0468 |
| D9 | 0,0441 | 0,0428 | 0,0423 | 0,0414 | 0,0426 | 0,0427 | 0,0427 | 0,0436 | 0,044 | 0,0438 | 0,0442 | 0,0419 | 0,0428 | 0,0438 | 0,0445 | 0,0432 | 0,0435 | 0,0418 | 0,0441 | 0,0431 | 0,0458 | 0,0459 | 0,0445 | 0,044 | 0,0451 | 0,0454 | 0,045 | 0,0444 | 0,0449 | 0,0438 | 0,0424 | 0,0463 | 0,0437 | 0,0434 | 0,0453 | 0,0445 | 0,0441 | 0,0451 | 0,0452 | 0,0464 | 0,0457 | 0,0451 | 0,0464 | 0,0448 | 0,0452 | 0,0454 |
| D10 | 0,0432 | 0,0397 | 0,0402 | 0,0393 | 0,0405 | 0,0419 | 0,0415 | 0,0421 | 0,0415 | 0,0422 | 0,0417 | 0,0403 | 0,0415 | 0,0414 | 0,0419 | 0,0418 | 0,0397 | 0,0406 | 0,0417 | 0,0412 | 0,0417 | 0,0398 | 0,0404 | 0,0406 | 0,0403 | 0,041 | 0,0411 | 0,0412 | 0,0398 | 0,0406 | 0,0407 | 0,0416 | 0,0422 | 0,0413 | 0,0412 | 0,0415 | 0,0403 | 0,0397 | 0,04 | 0,0414 | 0,0413 | 0,0404 | 0,0411 | 0,0411 | 0,0407 | 0,0403 |
| D11 | 0,0402 | 0,0397 | 0,0406 | 0,0401 | 0,0393 | 0,042 | 0,0401 | 0,0421 | 0,0411 | 0,0409 | 0,0414 | 0,0386 | 0,0416 | 0,0404 | 0,0412 | 0,0411 | 0,0399 | 0,0396 | 0,0429 | 0,0397 | 0,0424 | 0,0417 | 0,0422 | 0,0411 | 0,0423 | 0,0418 | 0,0419 | 0,0423 | 0,0385 | 0,0384 | 0,0407 | 0,0427 | 0,0422 | 0,0421 | 0,0416 | 0,0413 | 0,0395 | 0,0418 | 0,0409 | 0,0422 | 0,0413 | 0,0409 | 0,0412 | 0,0412 | 0,042 | 0,0412 |
| D12 | 0,0422 | 0,0411 | 0,0412 | 0,0395 | 0,0408 | 0,0407 | 0,0402 | 0,0425 | 0,0418 | 0,0409 | 0,0413 | 0,0393 | 0,0407 | 0,0403 | 0,0402 | 0,0402 | 0,0395 | 0,0413 | 0,0415 | 0,0392 | 0,0416 | 0,0391 | 0,0406 | 0,0402 | 0,0409 | 0,0421 | 0,0417 | 0,0407 | 0,0398 | 0,0373 | 0,0399 | 0,0419 | 0,0408 | 0,0406 | 0,0412 | 0,0411 | 0,0397 | 0,0405 | 0,0396 | 0,0407 | 0,0407 | 0,0395 | 0,0394 | 0,0393 | 0,0408 | 0,0417 |

**150**

**100**

**50**

**0**

**1**


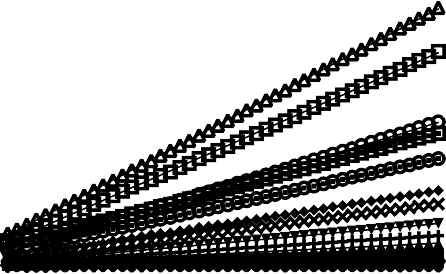


**0 5 10 15 20**

**Time(min)**

### WT 0

WT 0.125mM WT 0.25mM WT 0.5mM WT 1mM

###
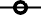
WT 2.5mM WT 5mM WT 7.5mM MUT 0


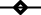

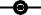
MUT 0.125mM MUT 0.25mM MUT 0.5mM MUT 1mM MUT 2.5mM MUT 5mM

### MUT 7.5mM

**150**

**100**

**50**

**0**

**2**


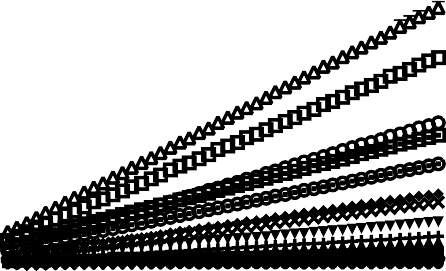


**0 5 10 15 20**

**Time(min)**

### WT 0

WT 0.125mM WT 0.25mM WT 0.5mM WT 1mM

###
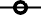
WT 2.5mM WT 5mM WT 7.5mM MUT 0


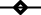

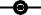
MUT 0.125mM MUT 0.25mM MUT 0.5mM MUT 1mM MUT 2.5mM MUT 5mM

### MUT 7.5mM

**WT**

**slop**

**8**


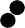

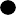

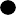


### **6** Km=4.570

**4**

**2**

**0**

**0 2 4 6 8**

**mM(p-NPP)**

Date: #########

Time: 10:33:44

System MTC-MU063-S

User MTC-MU063-S\TECAN

Plate Thermo Fisher Scientific-Nunclon 96 Flat Bottom Transparent Polystyrene Catalog No.: 269620/269787/439454/442404/475094 [NUN96ft.pdfx] Plate-ID (Stacker)

List of actions in this measurement script: Kinetic

Shaking (Linear) Duration: Shaking (Linear) Amplitude: Absorbance

2 s

1 mm

Label: Label1

Kinetic Measurement

Kinetic duration 00:15:00

Interval Time 00:00:20

Measurement Wavelength 405 nm

Bandwidth 9 nm

Number of Flashes 5

Settle Time 0 ms

Part of Plate A1-D12

Start Time: 2020 10-08 10:33:45

Cycle Nr.

1

2

3

4

5

6

7

8

9

10 11 12 13 14 15 16 17 18 19 20 21 22 23 24 25 26 27 28 29 30 31 32 33 34 35 36 37 38 39 40 41 42 43 44 45 46

|  |  |  |  |  |  |  |  |  |  |  |  |  |  |  |  |  |  |  |  |  |  |  |  |  |  |  |  |  |  |  |  |  |  |  |  |  |  |  |  |  |  |  |  |  |  |  |
| --- | --- | --- | --- | --- | --- | --- | --- | --- | --- | --- | --- | --- | --- | --- | --- | --- | --- | --- | --- | --- | --- | --- | --- | --- | --- | --- | --- | --- | --- | --- | --- | --- | --- | --- | --- | --- | --- | --- | --- | --- | --- | --- | --- | --- | --- | --- |
| Time [s] | 0 | 20 | 40 | 60 | 80 | 100 | 120 | 140 | 160 | 180 | 200 | 220 | 240 | 260 | 280 | 300 | 320 | 340 | 360 | 380 | 400 | 420 | 440 | 460 | 480 | 500 | 520 | 540 | 560 | 580 | 600 | 620 | 640 | 660 | 680 | 700 | 720 | 740 | 760 | 780 | 800 | 820 | 840 | 860 | 880 | 900 |
| Temp. [°C] | 26,4 | 26,5 | 26,3 | 26,5 | 26,8 | 26,7 | 26,4 | 26,4 | 26,4 | 26,5 | 26,7 | 26,5 | 26,4 | 26,4 | 26,4 | 26,8 | 26,4 | 26,6 | 26,8 | 26,5 | 26,7 | 26,6 | 26,4 | 26,8 | 26,5 | 26,6 | 26,5 | 26,5 | 26,5 | 26,5 | 26,7 | 27 | 26,5 | 26,8 | 26,5 | 26,7 | 26,5 | 26,8 | 26,7 | 27 | 26,8 | 27 | 27,2 | 26,9 | 27,1 | 26,9 |
| A1 | 0,0432 | 0,0433 | 0,0434 | 0,0436 | 0,0436 | 0,0436 | 0,0436 | 0,0436 | 0,0438 | 0,0436 | 0,0436 | 0,0437 | 0,0439 | 0,0436 | 0,0439 | 0,044 | 0,0441 | 0,0441 | 0,044 | 0,044 | 0,044 | 0,0442 | 0,0441 | 0,0441 | 0,044 | 0,0442 | 0,0442 | 0,0442 | 0,0443 | 0,0445 | 0,0446 | 0,0447 | 0,0445 | 0,0448 | 0,0448 | 0,0448 | 0,0449 | 0,0449 | 0,045 | 0,0452 | 0,0451 | 0,045 | 0,0452 | 0,0452 | 0,0452 | 0,0452 |
| A2 | 0,0481 | 0,0438 | 0,0439 | 0,0436 | 0,0432 | 0,0439 | 0,044 | 0,0447 | 0,0455 | 0,0469 | 0,0457 | 0,0465 | 0,046 | 0,0445 | 0,0465 | 0,0459 | 0,0464 | 0,0457 | 0,0451 | 0,045 | 0,0455 | 0,0444 | 0,045 | 0,0441 | 0,0447 | 0,0443 | 0,0443 | 0,0442 | 0,0443 | 0,0447 | 0,0442 | 0,0443 | 0,0442 | 0,0441 | 0,0443 | 0,0443 | 0,0446 | 0,0445 | 0,0447 | 0,0443 | 0,0445 | 0,0442 | 0,0444 | 0,0444 | 0,0445 | 0,0445 |
| A3 | 0,0447 | 0,0466 | 0,0451 | 0,0443 | 0,0446 | 0,0448 | 0,0449 | 0,0464 | 0,048 | 0,0473 | 0,0469 | 0,0475 | 0,0468 | 0,0457 | 0,0462 | 0,0477 | 0,0456 | 0,046 | 0,0461 | 0,0455 | 0,0461 | 0,0454 | 0,0459 | 0,0452 | 0,0453 | 0,0453 | 0,0453 | 0,0454 | 0,0453 | 0,0451 | 0,0452 | 0,0452 | 0,0452 | 0,0452 | 0,0451 | 0,0453 | 0,0454 | 0,0452 | 0,0453 | 0,0452 | 0,0452 | 0,0453 | 0,0453 | 0,0452 | 0,0453 | 0,0453 |
| A4 | 0,0466 | 0,0473 | 0,0486 | 0,0487 | 0,0495 | 0,0505 | 0,0511 | 0,0522 | 0,0544 | 0,0555 | 0,0549 | 0,0559 | 0,0568 | 0,0562 | 0,0572 | 0,0578 | 0,0588 | 0,0596 | 0,0592 | 0,0597 | 0,0602 | 0,0605 | 0,0614 | 0,0618 | 0,0623 | 0,0629 | 0,0636 | 0,0644 | 0,0649 | 0,0655 | 0,0661 | 0,067 | 0,0673 | 0,068 | 0,0686 | 0,0692 | 0,0701 | 0,0707 | 0,0713 | 0,0719 | 0,0724 | 0,073 | 0,0736 | 0,0744 | 0,0749 | 0,0756 |
| A5 | 0,048 | 0,0485 | 0,0502 | 0,0494 | 0,0508 | 0,0513 | 0,0524 | 0,0532 | 0,0552 | 0,0549 | 0,0551 | 0,0562 | 0,0564 | 0,0563 | 0,0572 | 0,0583 | 0,0589 | 0,0598 | 0,0603 | 0,0605 | 0,0613 | 0,0615 | 0,0622 | 0,0628 | 0,0632 | 0,0643 | 0,0641 | 0,0648 | 0,0656 | 0,0661 | 0,0667 | 0,0674 | 0,0679 | 0,0687 | 0,0694 | 0,0703 | 0,0707 | 0,072 | 0,0719 | 0,0733 | 0,0737 | 0,0737 | 0,0743 | 0,075 | 0,0757 | 0,0762 |
| A6 | 0,0475 | 0,0482 | 0,0488 | 0,0494 | 0,0503 | 0,0508 | 0,0516 | 0,052 | 0,0526 | 0,0532 | 0,0537 | 0,0544 | 0,055 | 0,0554 | 0,0567 | 0,0574 | 0,058 | 0,0584 | 0,0592 | 0,0598 | 0,0607 | 0,0614 | 0,0622 | 0,0626 | 0,0635 | 0,0641 | 0,0644 | 0,0651 | 0,0657 | 0,0665 | 0,0671 | 0,0678 | 0,0683 | 0,0689 | 0,0696 | 0,0701 | 0,071 | 0,0717 | 0,072 | 0,0725 | 0,0733 | 0,0736 | 0,0745 | 0,0753 | 0,0758 | 0,0765 |
| A7 | 0,0503 | 0,0515 | 0,0528 | 0,0541 | 0,0554 | 0,0567 | 0,0577 | 0,0593 | 0,0605 | 0,0615 | 0,0628 | 0,0641 | 0,0652 | 0,0661 | 0,0676 | 0,069 | 0,0702 | 0,0715 | 0,0725 | 0,0736 | 0,0751 | 0,0763 | 0,078 | 0,0786 | 0,0799 | 0,0814 | 0,0824 | 0,0838 | 0,0846 | 0,0857 | 0,0872 | 0,0887 | 0,0895 | 0,0908 | 0,0921 | 0,0935 | 0,0946 | 0,0957 | 0,0969 | 0,0984 | 0,0994 | 0,1006 | 0,102 | 0,1031 | 0,1045 | 0,1054 |
| A8 | 0,0502 | 0,0515 | 0,0526 | 0,0537 | 0,055 | 0,0565 | 0,0576 | 0,059 | 0,0602 | 0,0612 | 0,0624 | 0,0637 | 0,0652 | 0,066 | 0,0672 | 0,0684 | 0,0698 | 0,0708 | 0,072 | 0,0733 | 0,0744 | 0,0757 | 0,0768 | 0,0781 | 0,0793 | 0,0804 | 0,0818 | 0,083 | 0,0842 | 0,0855 | 0,0866 | 0,0878 | 0,0891 | 0,0903 | 0,0918 | 0,0929 | 0,0942 | 0,0954 | 0,0965 | 0,0978 | 0,0989 | 0,1001 | 0,1014 | 0,1027 | 0,104 | 0,1051 |
| A9 | 0,0511 | 0,0523 | 0,0537 | 0,0548 | 0,056 | 0,0572 | 0,0586 | 0,0595 | 0,0603 | 0,0615 | 0,0627 | 0,0639 | 0,0653 | 0,0662 | 0,0675 | 0,0684 | 0,0699 | 0,071 | 0,0722 | 0,0734 | 0,0746 | 0,0759 | 0,0771 | 0,0784 | 0,0796 | 0,0807 | 0,082 | 0,083 | 0,0845 | 0,0856 | 0,0868 | 0,0882 | 0,0894 | 0,0906 | 0,0918 | 0,0931 | 0,0945 | 0,0955 | 0,0968 | 0,0982 | 0,0994 | 0,1005 | 0,1018 | 0,1029 | 0,104 | 0,1052 |
| A10 | 0,0554 | 0,0577 | 0,0597 | 0,0617 | 0,0637 | 0,0662 | 0,0683 | 0,0706 | 0,0725 | 0,075 | 0,0771 | 0,0798 | 0,082 | 0,0839 | 0,0862 | 0,0884 | 0,0909 | 0,0927 | 0,0951 | 0,0972 | 0,0997 | 0,1021 | 0,1041 | 0,1064 | 0,1089 | 0,1108 | 0,113 | 0,1152 | 0,1176 | 0,1196 | 0,122 | 0,1245 | 0,1266 | 0,1289 | 0,131 | 0,1337 | 0,1359 | 0,1381 | 0,1404 | 0,1428 | 0,1449 | 0,1471 | 0,1494 | 0,1517 | 0,154 | 0,1564 |
| A11 | 0,0568 | 0,0585 | 0,0605 | 0,0632 | 0,0657 | 0,0677 | 0,0697 | 0,0727 | 0,0743 | 0,0764 | 0,0781 | 0,0808 | 0,0839 | 0,085 | 0,0872 | 0,0896 | 0,092 | 0,0943 | 0,0963 | 0,0992 | 0,1012 | 0,1039 | 0,1059 | 0,108 | 0,1104 | 0,1129 | 0,1149 | 0,1177 | 0,1194 | 0,1216 | 0,1245 | 0,127 | 0,1291 | 0,1309 | 0,1332 | 0,1357 | 0,1381 | 0,1404 | 0,1437 | 0,1453 | 0,1473 | 0,1494 | 0,152 | 0,1545 | 0,157 | 0,1589 |
| A12 | 0,054 | 0,0562 | 0,0589 | 0,0614 | 0,0633 | 0,0657 | 0,0682 | 0,0701 | 0,0726 | 0,0748 | 0,0768 | 0,0794 | 0,0815 | 0,0834 | 0,0857 | 0,0877 | 0,0899 | 0,092 | 0,0943 | 0,0966 | 0,0987 | 0,1012 | 0,1034 | 0,1058 | 0,1081 | 0,1102 | 0,1125 | 0,1147 | 0,1171 | 0,1194 | 0,1217 | 0,1241 | 0,1262 | 0,1287 | 0,1311 | 0,1333 | 0,1357 | 0,1379 | 0,14 | 0,1426 | 0,1447 | 0,1468 | 0,1495 | 0,1516 | 0,1538 | 0,1561 |
| B1 | 0,0755 | 0,0669 | 0,0699 | 0,0738 | 0,0795 | 0,0842 | 0,0866 | 0,0926 | 0,0977 | 0,0999 | 0,1041 | 0,1113 | 0,1142 | 0,1175 | 0,1229 | 0,1301 | 0,1324 | 0,1346 | 0,1376 | 0,1416 | 0,1475 | 0,1509 | 0,1551 | 0,157 | 0,1606 | 0,1649 | 0,1681 | 0,1728 | 0,1766 | 0,1802 | 0,1847 | 0,1892 | 0,1928 | 0,1965 | 0,2013 | 0,2049 | 0,2088 | 0,2129 | 0,2168 | 0,2213 | 0,2248 | 0,2291 | 0,2327 | 0,2365 | 0,2414 | 0,2451 |
| B2 | 0,074 | 0,0676 | 0,0724 | 0,0761 | 0,0807 | 0,0857 | 0,0881 | 0,0939 | 0,0973 | 0,1018 | 0,1059 | 0,1122 | 0,1163 | 0,1191 | 0,1256 | 0,1286 | 0,1316 | 0,1328 | 0,1377 | 0,1405 | 0,1479 | 0,1496 | 0,1535 | 0,1567 | 0,1607 | 0,1645 | 0,1692 | 0,1725 | 0,1764 | 0,1801 | 0,1842 | 0,189 | 0,1926 | 0,1964 | 0,2009 | 0,2043 | 0,2088 | 0,2128 | 0,2164 | 0,2193 | 0,2223 | 0,2266 | 0,2305 | 0,236 | 0,2386 | 0,2432 |
| B3 | 0,0695 | 0,0666 | 0,071 | 0,0743 | 0,0783 | 0,0843 | 0,0857 | 0,0914 | 0,0963 | 0,099 | 0,1031 | 0,1086 | 0,1151 | 0,1178 | 0,1211 | 0,1274 | 0,1297 | 0,1317 | 0,1376 | 0,1402 | 0,1449 | 0,1484 | 0,1515 | 0,1544 | 0,1588 | 0,1627 | 0,1669 | 0,1708 | 0,1753 | 0,1779 | 0,1818 | 0,1854 | 0,1906 | 0,1947 | 0,1988 | 0,2019 | 0,2058 | 0,2097 | 0,2133 | 0,2175 | 0,2215 | 0,2255 | 0,2296 | 0,2333 | 0,2375 | 0,2417 |
| B4 | 0,0886 | 0,0894 | 0,0967 | 0,1032 | 0,1114 | 0,1218 | 0,1257 | 0,1369 | 0,1435 | 0,1502 | 0,1568 | 0,1665 | 0,1757 | 0,1813 | 0,1888 | 0,1948 | 0,2012 | 0,2068 | 0,2148 | 0,2214 | 0,2299 | 0,2366 | 0,2438 | 0,2507 | 0,2592 | 0,266 | 0,2728 | 0,2808 | 0,2885 | 0,2951 | 0,3034 | 0,3119 | 0,3204 | 0,3257 | 0,3342 | 0,3415 | 0,3492 | 0,3567 | 0,3649 | 0,372 | 0,3803 | 0,3871 | 0,3941 | 0,4024 | 0,4106 | 0,4179 |
| B5 | 0,0876 | 0,0891 | 0,097 | 0,1048 | 0,1125 | 0,1202 | 0,1264 | 0,1353 | 0,1436 | 0,1497 | 0,1565 | 0,1668 | 0,1734 | 0,1795 | 0,1884 | 0,1962 | 0,2034 | 0,2084 | 0,2159 | 0,2234 | 0,2328 | 0,2387 | 0,2459 | 0,2526 | 0,2606 | 0,2681 | 0,2747 | 0,2823 | 0,2899 | 0,2972 | 0,3054 | 0,3134 | 0,3209 | 0,3275 | 0,3361 | 0,3433 | 0,3501 | 0,3587 | 0,366 | 0,3744 | 0,3808 | 0,3879 | 0,3966 | 0,4057 | 0,4137 | 0,4193 |
| B6 | 0,088 | 0,0903 | 0,0978 | 0,1053 | 0,1131 | 0,1212 | 0,1274 | 0,1355 | 0,1438 | 0,1515 | 0,1584 | 0,1679 | 0,1757 | 0,1828 | 0,1917 | 0,199 | 0,2073 | 0,2123 | 0,2211 | 0,2283 | 0,2382 | 0,2442 | 0,2518 | 0,2585 | 0,2667 | 0,2746 | 0,2824 | 0,2902 | 0,2981 | 0,3058 | 0,3134 | 0,3212 | 0,3291 | 0,3372 | 0,3454 | 0,3531 | 0,3607 | 0,3695 | 0,3777 | 0,3845 | 0,3923 | 0,3999 | 0,4092 | 0,4177 | 0,4248 | 0,4327 |
| B7 | 0,1133 | 0,1191 | 0,1307 | 0,1419 | 0,1527 | 0,1635 | 0,1747 | 0,1852 | 0,1964 | 0,2087 | 0,2197 | 0,2328 | 0,2438 | 0,2549 | 0,2648 | 0,2731 | 0,2845 | 0,2936 | 0,3058 | 0,3161 | 0,3294 | 0,3387 | 0,3495 | 0,3611 | 0,3721 | 0,3844 | 0,3949 | 0,4067 | 0,4173 | 0,4289 | 0,4404 | 0,4513 | 0,4637 | 0,4766 | 0,4865 | 0,4965 | 0,5073 | 0,5197 | 0,5309 | 0,542 | 0,554 | 0,5652 | 0,5788 | 0,5883 | 0,5993 | 0,6111 |
| B8 | 0,1109 | 0,1193 | 0,131 | 0,1422 | 0,1536 | 0,1632 | 0,1764 | 0,1848 | 0,1955 | 0,2084 | 0,2197 | 0,2299 | 0,2418 | 0,2527 | 0,2625 | 0,2712 | 0,2825 | 0,2923 | 0,3035 | 0,3143 | 0,3259 | 0,3374 | 0,3481 | 0,3588 | 0,3714 | 0,3818 | 0,3919 | 0,404 | 0,4154 | 0,4256 | 0,4364 | 0,4479 | 0,4614 | 0,4711 | 0,4822 | 0,4938 | 0,5058 | 0,5159 | 0,5283 | 0,5397 | 0,5521 | 0,5612 | 0,5722 | 0,5831 | 0,5943 | 0,608 |
| B9 | 0,1112 | 0,1187 | 0,1292 | 0,1405 | 0,1509 | 0,1608 | 0,1726 | 0,1825 | 0,1937 | 0,2046 | 0,2159 | 0,2275 | 0,2389 | 0,2497 | 0,262 | 0,2701 | 0,2821 | 0,2907 | 0,3026 | 0,3127 | 0,3265 | 0,336 | 0,3466 | 0,3563 | 0,3681 | 0,3791 | 0,3897 | 0,4014 | 0,4118 | 0,4231 | 0,4346 | 0,4457 | 0,4574 | 0,4676 | 0,4788 | 0,4909 | 0,5016 | 0,5128 | 0,5243 | 0,5354 | 0,546 | 0,5566 | 0,5684 | 0,5802 | 0,5901 | 0,6032 |
| B10 | 0,1336 | 0,1427 | 0,1556 | 0,1692 | 0,1813 | 0,1935 | 0,2072 | 0,2179 | 0,2312 | 0,2442 | 0,2579 | 0,271 | 0,2833 | 0,2962 | 0,3098 | 0,3215 | 0,3359 | 0,3471 | 0,3612 | 0,3744 | 0,3896 | 0,4006 | 0,4138 | 0,4256 | 0,4394 | 0,453 | 0,4663 | 0,4795 | 0,4929 | 0,5057 | 0,5186 | 0,5321 | 0,5454 | 0,5602 | 0,5729 | 0,5858 | 0,5987 | 0,6125 | 0,6266 | 0,6393 | 0,6522 | 0,6658 | 0,6795 | 0,6938 | 0,7045 | 0,7201 |
| B11 | 0,1309 | 0,1423 | 0,155 | 0,1674 | 0,1795 | 0,192 | 0,2055 | 0,217 | 0,23 | 0,2432 | 0,2567 | 0,2698 | 0,2835 | 0,2973 | 0,309 | 0,32 | 0,3337 | 0,3451 | 0,3593 | 0,3719 | 0,3851 | 0,3987 | 0,4112 | 0,4247 | 0,4374 | 0,4511 | 0,4645 | 0,4771 | 0,4901 | 0,5031 | 0,5163 | 0,5282 | 0,5434 | 0,5586 | 0,5692 | 0,5842 | 0,5972 | 0,6078 | 0,6222 | 0,6363 | 0,6493 | 0,665 | 0,6766 | 0,6875 | 0,7003 | 0,7159 |
| B12 | 0,1282 | 0,1415 | 0,1537 | 0,167 | 0,179 | 0,192 | 0,2045 | 0,2176 | 0,2314 | 0,2443 | 0,2572 | 0,2699 | 0,2836 | 0,2965 | 0,3099 | 0,3231 | 0,3369 | 0,3509 | 0,3629 | 0,3767 | 0,3898 | 0,4037 | 0,4171 | 0,4303 | 0,4447 | 0,4589 | 0,4708 | 0,4838 | 0,4977 | 0,5105 | 0,5257 | 0,5398 | 0,5542 | 0,5664 | 0,5818 | 0,5947 | 0,6079 | 0,6205 | 0,6355 | 0,6485 | 0,6615 | 0,6756 | 0,6876 | 0,7029 | 0,7166 | 0,7298 |
| C1 | 0,0448 | 0,0449 | 0,0458 | 0,0459 | 0,0457 | 0,0456 | 0,0458 | 0,0458 | 0,0465 | 0,0458 | 0,046 | 0,0462 | 0,0463 | 0,046 | 0,0459 | 0,0464 | 0,0459 | 0,046 | 0,0463 | 0,0462 | 0,0465 | 0,0462 | 0,0462 | 0,0462 | 0,0458 | 0,0461 | 0,0462 | 0,0464 | 0,0458 | 0,046 | 0,046 | 0,0463 | 0,046 | 0,0461 | 0,0463 | 0,046 | 0,0459 | 0,0463 | 0,046 | 0,0461 | 0,0462 | 0,0459 | 0,0461 | 0,0461 | 0,0461 | 0,0459 |
| C2 | 0,0454 | 0,0482 | 0,045 | 0,0457 | 0,0451 | 0,0452 | 0,0468 | 0,0478 | 0,0469 | 0,0463 | 0,0465 | 0,0461 | 0,0461 | 0,0462 | 0,0461 | 0,0461 | 0,0462 | 0,0464 | 0,0462 | 0,0463 | 0,0467 | 0,0465 | 0,0464 | 0,0465 | 0,0463 | 0,0462 | 0,0466 | 0,0463 | 0,0471 | 0,0461 | 0,0464 | 0,0464 | 0,0471 | 0,0463 | 0,0464 | 0,0464 | 0,0467 | 0,0472 | 0,0466 | 0,0462 | 0,0468 | 0,0462 | 0,0474 | 0,0463 | 0,0463 | 0,0463 |
| C3 | 0,0452 | 0,0459 | 0,0447 | 0,0444 | 0,0445 | 0,0452 | 0,0456 | 0,0458 | 0,0457 | 0,0458 | 0,0458 | 0,0459 | 0,0458 | 0,0458 | 0,0458 | 0,0459 | 0,046 | 0,0461 | 0,0458 | 0,0459 | 0,0461 | 0,046 | 0,0459 | 0,0459 | 0,0461 | 0,046 | 0,0459 | 0,0462 | 0,046 | 0,0458 | 0,0461 | 0,0459 | 0,0461 | 0,0461 | 0,0461 | 0,0461 | 0,0464 | 0,0464 | 0,0458 | 0,0462 | 0,046 | 0,046 | 0,046 | 0,046 | 0,046 | 0,0459 |
| C4 | 0,0455 | 0,0479 | 0,0496 | 0,0466 | 0,0467 | 0,0472 | 0,0473 | 0,0482 | 0,0484 | 0,049 | 0,0491 | 0,0493 | 0,0497 | 0,0499 | 0,05 | 0,0502 | 0,0503 | 0,0506 | 0,0509 | 0,051 | 0,0515 | 0,0516 | 0,0517 | 0,052 | 0,052 | 0,0524 | 0,0525 | 0,0529 | 0,0531 | 0,0531 | 0,0535 | 0,0537 | 0,0541 | 0,0541 | 0,0545 | 0,0546 | 0,0551 | 0,0551 | 0,0552 | 0,0556 | 0,0558 | 0,0557 | 0,0563 | 0,0563 | 0,0566 | 0,0566 |
| C5 | 0,0466 | 0,0483 | 0,0493 | 0,048 | 0,0481 | 0,0487 | 0,0486 | 0,0495 | 0,0499 | 0,0502 | 0,0504 | 0,0506 | 0,0511 | 0,0511 | 0,0513 | 0,0516 | 0,0515 | 0,0518 | 0,0521 | 0,0523 | 0,0526 | 0,0528 | 0,0532 | 0,0534 | 0,0534 | 0,0536 | 0,0539 | 0,0539 | 0,0542 | 0,0543 | 0,0548 | 0,055 | 0,0555 | 0,0554 | 0,0559 | 0,0557 | 0,0565 | 0,0565 | 0,0567 | 0,0569 | 0,0569 | 0,0569 | 0,0574 | 0,0576 | 0,0579 | 0,0581 |
| C6 | 0,0493 | 0,0493 | 0,0479 | 0,0483 | 0,0485 | 0,0496 | 0,0498 | 0,0501 | 0,0506 | 0,0509 | 0,051 | 0,0515 | 0,0517 | 0,0517 | 0,0518 | 0,0521 | 0,0524 | 0,0526 | 0,0529 | 0,053 | 0,0535 | 0,0537 | 0,0538 | 0,0541 | 0,0544 | 0,0545 | 0,0548 | 0,0548 | 0,0551 | 0,0555 | 0,0555 | 0,0561 | 0,0565 | 0,0565 | 0,0567 | 0,0571 | 0,0575 | 0,0574 | 0,0575 | 0,0583 | 0,0581 | 0,058 | 0,0585 | 0,0586 | 0,059 | 0,0592 |
| C7 | 0,0481 | 0,0537 | 0,0497 | 0,0488 | 0,0497 | 0,0512 | 0,0516 | 0,0518 | 0,0525 | 0,053 | 0,0534 | 0,0538 | 0,0541 | 0,0548 | 0,055 | 0,0557 | 0,0563 | 0,0565 | 0,057 | 0,0575 | 0,0583 | 0,0585 | 0,0591 | 0,0594 | 0,0601 | 0,0604 | 0,061 | 0,0612 | 0,0618 | 0,0624 | 0,0629 | 0,0639 | 0,064 | 0,0643 | 0,0651 | 0,0655 | 0,0665 | 0,0666 | 0,0668 | 0,0677 | 0,0679 | 0,0684 | 0,069 | 0,0694 | 0,0702 | 0,0706 |
| C8 | 0,0496 | 0,0531 | 0,0491 | 0,0497 | 0,0503 | 0,0513 | 0,0517 | 0,0518 | 0,053 | 0,053 | 0,0532 | 0,0539 | 0,0543 | 0,0547 | 0,0551 | 0,0556 | 0,0562 | 0,0568 | 0,0573 | 0,0578 | 0,0585 | 0,0586 | 0,059 | 0,0596 | 0,06 | 0,0605 | 0,0611 | 0,0613 | 0,0618 | 0,0627 | 0,0631 | 0,0637 | 0,0641 | 0,0647 | 0,0653 | 0,0657 | 0,0665 | 0,0665 | 0,0669 | 0,0675 | 0,068 | 0,068 | 0,0692 | 0,0692 | 0,0701 | 0,0704 |
| C9 | 0,0484 | 0,0585 | 0,0487 | 0,0487 | 0,049 | 0,0498 | 0,0506 | 0,0511 | 0,0517 | 0,052 | 0,0524 | 0,0531 | 0,0534 | 0,0538 | 0,0543 | 0,0548 | 0,055 | 0,0555 | 0,056 | 0,0564 | 0,0568 | 0,0575 | 0,0579 | 0,0583 | 0,0586 | 0,0591 | 0,0596 | 0,0597 | 0,0604 | 0,0611 | 0,0611 | 0,062 | 0,0625 | 0,0629 | 0,0632 | 0,0636 | 0,0646 | 0,0648 | 0,065 | 0,0654 | 0,0661 | 0,066 | 0,067 | 0,0672 | 0,0676 | 0,0685 |
| C10 | 0,0521 | 0,0605 | 0,0517 | 0,0524 | 0,0538 | 0,0547 | 0,0556 | 0,056 | 0,0571 | 0,0577 | 0,0585 | 0,0591 | 0,06 | 0,0608 | 0,0613 | 0,0624 | 0,0633 | 0,0655 | 0,0645 | 0,0655 | 0,0667 | 0,0673 | 0,0678 | 0,0688 | 0,0698 | 0,0704 | 0,071 | 0,0717 | 0,0726 | 0,0735 | 0,0744 | 0,0756 | 0,0763 | 0,0776 | 0,0777 | 0,0787 | 0,0799 | 0,0806 | 0,0811 | 0,0848 | 0,0833 | 0,0832 | 0,0847 | 0,0852 | 0,0861 | 0,0887 |
| C11 | 0,0512 | 0,0546 | 0,0543 | 0,0542 | 0,0552 | 0,0559 | 0,055 | 0,0555 | 0,0567 | 0,0591 | 0,058 | 0,0612 | 0,0595 | 0,062 | 0,0609 | 0,0618 | 0,0627 | 0,0654 | 0,0641 | 0,0667 | 0,0682 | 0,0667 | 0,0679 | 0,0683 | 0,0711 | 0,0698 | 0,0722 | 0,0713 | 0,0721 | 0,0754 | 0,0759 | 0,075 | 0,0766 | 0,0783 | 0,0774 | 0,0784 | 0,0794 | 0,0817 | 0,0819 | 0,0832 | 0,0837 | 0,0826 | 0,0838 | 0,0847 | 0,0871 | 0,0886 |
| C12 | 0,05 | 0,0523 | 0,0549 | 0,0524 | 0,0538 | 0,0538 | 0,0551 | 0,0556 | 0,0565 | 0,0573 | 0,0581 | 0,0589 | 0,0597 | 0,0609 | 0,0613 | 0,0624 | 0,0628 | 0,0636 | 0,0644 | 0,0654 | 0,0661 | 0,067 | 0,0679 | 0,069 | 0,0695 | 0,0705 | 0,0712 | 0,0719 | 0,0727 | 0,0738 | 0,0745 | 0,0754 | 0,0762 | 0,0774 | 0,0782 | 0,0788 | 0,0799 | 0,081 | 0,0814 | 0,0823 | 0,0831 | 0,0836 | 0,0849 | 0,0858 | 0,0865 | 0,0871 |
| D1 | 0,062 | 0,0615 | 0,0592 | 0,061 | 0,0618 | 0,0632 | 0,0646 | 0,0662 | 0,0677 | 0,069 | 0,0706 | 0,0722 | 0,0735 | 0,0751 | 0,0765 | 0,0781 | 0,0798 | 0,0814 | 0,083 | 0,0846 | 0,0862 | 0,088 | 0,0895 | 0,0914 | 0,0929 | 0,0944 | 0,0961 | 0,0976 | 0,0994 | 0,1011 | 0,1029 | 0,1046 | 0,1061 | 0,1078 | 0,1095 | 0,1112 | 0,1134 | 0,1148 | 0,1162 | 0,118 | 0,1193 | 0,1208 | 0,1227 | 0,1245 | 0,1262 | 0,1278 |
| D2 | 0,0603 | 0,0591 | 0,0581 | 0,0598 | 0,0607 | 0,0622 | 0,0638 | 0,0654 | 0,0668 | 0,0681 | 0,0697 | 0,0711 | 0,0726 | 0,074 | 0,0755 | 0,0771 | 0,0785 | 0,0802 | 0,0818 | 0,0836 | 0,0848 | 0,0866 | 0,0881 | 0,0897 | 0,0912 | 0,0928 | 0,0944 | 0,0958 | 0,0974 | 0,0991 | 0,101 | 0,1023 | 0,104 | 0,1057 | 0,1071 | 0,1088 | 0,1111 | 0,112 | 0,1138 | 0,1152 | 0,1169 | 0,1182 | 0,1201 | 0,1217 | 0,1233 | 0,125 |
| D3 | 0,0599 | 0,0642 | 0,0588 | 0,0604 | 0,0615 | 0,0623 | 0,0636 | 0,065 | 0,0663 | 0,0678 | 0,0692 | 0,0708 | 0,0722 | 0,0737 | 0,0749 | 0,0766 | 0,0779 | 0,0795 | 0,081 | 0,0825 | 0,084 | 0,0857 | 0,0872 | 0,0888 | 0,0904 | 0,0918 | 0,0932 | 0,0948 | 0,0964 | 0,0982 | 0,0998 | 0,1013 | 0,1029 | 0,1044 | 0,106 | 0,1076 | 0,1097 | 0,1105 | 0,1122 | 0,1136 | 0,1152 | 0,1168 | 0,1184 | 0,1199 | 0,1216 | 0,1231 |
| D4 | 0,071 | 0,0741 | 0,074 | 0,0765 | 0,0795 | 0,0818 | 0,0846 | 0,0876 | 0,0903 | 0,093 | 0,096 | 0,0987 | 0,1017 | 0,1044 | 0,1073 | 0,1104 | 0,1134 | 0,1162 | 0,1194 | 0,1226 | 0,1257 | 0,1289 | 0,1322 | 0,1352 | 0,1381 | 0,1412 | 0,1445 | 0,1476 | 0,1507 | 0,1537 | 0,157 | 0,1602 | 0,1636 | 0,1665 | 0,1699 | 0,1729 | 0,177 | 0,1793 | 0,1824 | 0,1857 | 0,1891 | 0,1917 | 0,1945 | 0,1984 | 0,2014 | 0,2047 |
| D5 | 0,0747 | 0,0777 | 0,0744 | 0,0787 | 0,0807 | 0,0831 | 0,0853 | 0,0884 | 0,0912 | 0,0938 | 0,0968 | 0,1 | 0,1027 | 0,1057 | 0,109 | 0,1119 | 0,1148 | 0,1176 | 0,1211 | 0,1243 | 0,1273 | 0,1308 | 0,1339 | 0,1371 | 0,1402 | 0,1432 | 0,1465 | 0,1495 | 0,1529 | 0,1561 | 0,1596 | 0,1628 | 0,1655 | 0,169 | 0,1723 | 0,1755 | 0,1793 | 0,1825 | 0,1858 | 0,1891 | 0,1916 | 0,1948 | 0,1979 | 0,202 | 0,2048 | 0,2076 |
| D6 | 0,0695 | 0,0792 | 0,0748 | 0,0783 | 0,0809 | 0,0828 | 0,0856 | 0,0885 | 0,0912 | 0,0939 | 0,0971 | 0,1005 | 0,1031 | 0,106 | 0,1094 | 0,1125 | 0,1156 | 0,1191 | 0,1225 | 0,1258 | 0,1288 | 0,1322 | 0,1355 | 0,1386 | 0,1419 | 0,1452 | 0,1483 | 0,1515 | 0,1552 | 0,1583 | 0,1619 | 0,1653 | 0,1685 | 0,172 | 0,175 | 0,1784 | 0,1825 | 0,1856 | 0,1886 | 0,1918 | 0,1951 | 0,1986 | 0,202 | 0,2055 | 0,2086 | 0,2119 |
| D7 | 0,0912 | 0,1019 | 0,0999 | 0,1042 | 0,1086 | 0,1132 | 0,1174 | 0,1222 | 0,1271 | 0,1319 | 0,1366 | 0,1417 | 0,1466 | 0,1517 | 0,1568 | 0,1623 | 0,1677 | 0,173 | 0,1783 | 0,1839 | 0,189 | 0,1944 | 0,1997 | 0,2053 | 0,2107 | 0,2163 | 0,2213 | 0,2273 | 0,2325 | 0,2379 | 0,2435 | 0,2489 | 0,2543 | 0,2602 | 0,2655 | 0,2714 | 0,2772 | 0,2818 | 0,2873 | 0,2929 | 0,2988 | 0,3042 | 0,3094 | 0,315 | 0,321 | 0,3262 |
| D8 | 0,0919 | 0,0981 | 0,0982 | 0,1041 | 0,1084 | 0,1129 | 0,1179 | 0,1229 | 0,1279 | 0,1328 | 0,1377 | 0,1432 | 0,1486 | 0,1536 | 0,159 | 0,1647 | 0,1705 | 0,1754 | 0,1812 | 0,1869 | 0,1924 | 0,1976 | 0,2032 | 0,2089 | 0,2144 | 0,2201 | 0,2252 | 0,2309 | 0,2368 | 0,2424 | 0,2482 | 0,254 | 0,2597 | 0,2654 | 0,2709 | 0,2769 | 0,2833 | 0,2883 | 0,294 | 0,2998 | 0,3057 | 0,3104 | 0,3158 | 0,3222 | 0,3282 | 0,3331 |
| D9 | 0,0881 | 0,0997 | 0,0981 | 0,103 | 0,1069 | 0,1116 | 0,1163 | 0,1217 | 0,1259 | 0,1303 | 0,1354 | 0,1406 | 0,1452 | 0,1502 | 0,1554 | 0,1611 | 0,1658 | 0,171 | 0,1765 | 0,1823 | 0,187 | 0,1927 | 0,1984 | 0,2036 | 0,2089 | 0,2142 | 0,2195 | 0,2246 | 0,2301 | 0,2355 | 0,2413 | 0,2474 | 0,2518 | 0,2574 | 0,2631 | 0,2687 | 0,275 | 0,2797 | 0,2852 | 0,291 | 0,2956 | 0,3013 | 0,3061 | 0,3131 | 0,3177 | 0,323 |
| D10 | 0,1093 | 0,1174 | 0,1176 | 0,1249 | 0,1291 | 0,1349 | 0,1409 | 0,1458 | 0,1525 | 0,1577 | 0,1632 | 0,1694 | 0,1749 | 0,181 | 0,1876 | 0,1938 | 0,2 | 0,2073 | 0,2135 | 0,2199 | 0,2258 | 0,2323 | 0,2389 | 0,2455 | 0,2527 | 0,2588 | 0,2649 | 0,2716 | 0,2794 | 0,285 | 0,2917 | 0,2979 | 0,3048 | 0,3117 | 0,3181 | 0,3243 | 0,3316 | 0,3384 | 0,3445 | 0,3504 | 0,3562 | 0,3634 | 0,3707 | 0,3764 | 0,3831 | 0,3894 |
| D11 | 0,106 | 0,1157 | 0,1178 | 0,1237 | 0,1291 | 0,1348 | 0,1404 | 0,1461 | 0,1519 | 0,1577 | 0,1641 | 0,17 | 0,1763 | 0,1826 | 0,1886 | 0,1953 | 0,2021 | 0,2081 | 0,2157 | 0,2217 | 0,2279 | 0,2353 | 0,2414 | 0,2483 | 0,2548 | 0,2612 | 0,2684 | 0,2752 | 0,2817 | 0,2885 | 0,2947 | 0,3016 | 0,3088 | 0,3161 | 0,3224 | 0,3291 | 0,3368 | 0,3429 | 0,3492 | 0,3556 | 0,3627 | 0,37 | 0,3753 | 0,3824 | 0,3889 | 0,3961 |
| D12 | 0,1022 | 0,1096 | 0,1146 | 0,1223 | 0,1284 | 0,1341 | 0,14 | 0,1459 | 0,1516 | 0,1574 | 0,1636 | 0,1695 | 0,1759 | 0,1821 | 0,1883 | 0,1951 | 0,2015 | 0,2076 | 0,2153 | 0,222 | 0,2284 | 0,2356 | 0,2426 | 0,2497 | 0,255 | 0,2611 | 0,2688 | 0,2744 | 0,2815 | 0,2893 | 0,296 | 0,3018 | 0,3086 | 0,3154 | 0,3222 | 0,328 | 0,3382 | 0,3418 | 0,3493 | 0,3555 | 0,3617 | 0,3715 | 0,3759 | 0,3823 | 0,3876 | 0,3953 |
