# Supplementary material for: Redox regulation of PTPN22 affects the severity of T-cell-dependent autoimmune inflammation
Source: eLife. 2022 May 19;11:e74549. doi: 10.7554/eLife.74549 (PMC9119677; doi:10.7554/eLife.74549)
Supplement: Figure 1—source data 2. [file elife-74549-fig1-data2.docx]

Activity of WT PTPN22

treated with 0,3.1,6.2,12.5,25,50µM H_2_O_2_ and 1mM NaN_3_ was measured at 5,15,30 minutes

**30**


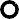

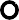

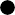


PTPN22 reduced control

**PTPN22 activity min-1**

PTPN22 3.4uM H2O2

**20** PTPN22 6.3uM H2O2

PTPN22 12.5uM H2O2 PTPN22 25uM H2O2

**10**

PTPN22 50uM H2O2

**0**

**0 10 20 30 40**

**min**

min

PTPN22 reduced control PTPN22 3.4uM H2O2 PTPN22 6.3uM H2O2 PTPN22 12.5uM H2O2 PTPN22 25uM H2O2

PTPN22 50uM H2O2

1 2 3 mean sd sem 1 2 3 mean sd sem 1 2 3 mean sd sem 1 2 3 mean sd sem 1 2 3 mean sd sem 1 2 3 mean sd sem

5 36,29645 40,28773 42,29972 39,62797 3,055531 2,841557 38,83254 35,51168 33,24784 35,86402 2,808972 2,003608 34,31835 39,67431 31,98404 35,32557 3,942833 0,757188 36,95069 34,9052 31,2601 34,372 2,88252 1,560271 31,57471 36,20905 33,80081 33,86152 2,317765 0,856997 34,54708 33,41882 32,14231 33,3694 1,203146 4,539089

| 15 36,75739 49,86215 35,89829 40,83928 7,825833 0,87178 30,04482 48,44701 33,55667 | 37,3495 9,769814 1,545244 33,18315 38,19794 | 30,29743 33,89284 3,997781 1,134803 35,15279 37,91222 29,92507 34,33003 4,056644 1,517674 37,26607 36,35807 31,25318 34,9591 3,241376 0,24037 28,74251 25,73202 33,66411 29,37955 4,004233 0,409607 |
| --- | --- | --- |
| 30 38,93189 37,76897 34,30526 37,00204 2,406774 2,386071 37,68618 38,78133 33,29829 | 36,5886 2,901625 0,360555 32,27901 38,47789 | 33,378 34,71163 3,307638 2,305308 33,53703 39,1406 27,85723 33,51162 5,641727 0,87178 28,52302 30,50383 25,39354 28,14013 2,576569 1,507021 22,16009 28,04707 18,37908 22,86208 4,872072 1,171893 |

| 54 | 49 | 51,5 | 49,1 | 49,5 | 57,8 | 49,2 | 49,7 | 61,8 | 55 | 50,8 | 54,5 | 55,8 | 44,1 | 53,4 | 44,7 | 43,8 | 45,2 |
| --- | --- | --- | --- | --- | --- | --- | --- | --- | --- | --- | --- | --- | --- | --- | --- | --- | --- |
| 52,2 | 51,2 | 51,2 | 49,3 | 49,5 | 51,7 | 52,1 | 50,5 | 48,9 | 38 | 46,6 | 46,8 | 33,3 | 38 | 38,9 | 24,7 | 31,2 | 28,6 |
| 49,2 | 51,4 | 45,2 | 40,5 | 48,4 | 44,4 | 33,8 | 48,5 | 42,1 | 32,7 | 41,1 | 37,1 | 24 | 35,5 | 27 | 16,6 | 17,5 | 16,2 |

7,714286 7 7,357143 7,014286 7,071429 8,257143 7,028571 7,1 8,828571 7,857143 7,257143 7,785714 7,971429 6,3 7,628571 6,385714 6,257143 6,457143

7,457143 7,314286 7,314286 7,042857 7,071429 7,385714 7,442857 7,214286 6,985714 5,428571 6,657143 6,685714 4,757143 5,428571 5,557143 3,528571 4,457143 4,085714

7,028571 7,342857 6,457143 5,785714 6,914286 6,342857 4,828571 6,928571 6,014286 4,671429 5,871429 5,3 3,428571 5,071429 3,857143 2,371429 2,5 2,314286

23,14286 21 22,07143 21,04286 21,21429 24,77143 21,08571 21,3 26,48571 23,57143 21,77143 23,35714 23,91429 18,9 22,88571 19,15714 18,77143 19,37143

22,37143 21,94286 21,94286 21,12857 21,21429 22,15714 22,32857 21,64286 20,95714 16,28571 19,97143 20,05714 14,27143 16,28571 16,67143 10,58571 13,37143 12,25714

21,08571 22,02857 19,37143 17,35714 20,74286 19,02857 14,48571 20,78571 18,04286 14,01429 17,61429 15,9 10,28571 15,21429 11,57143 7,114286 7,5 6,942857

| 20 | 0,251300007 | 0,251899987 | 0,251599997 | | 0,259200007 | 0,262100011 | 0,256199986 | 0,260399997 | 0,255600005 | 0,2456 | 0,252099991 | 0,249599993 | 0,256900012 | 0,254200011 | 0,250499994 |
| --- | --- | --- | --- | --- | --- | --- | --- | --- | --- | --- | --- | --- | --- | --- | --- |
| 40 | 0,252499998 | 0,253600001 | 0,25850001 | | 0,2588 | 0,2588 | 0,259499997 | 0,268000007 | 0,263200015 | 0,248300001 | 0,259299994 | 0,256799996 | 0,263500005 | 0,262699991 | 0,257600009 |
| 60 | 0,251100004 | 0,252499998 | 0,264400005 | | 0,263300002 | 0,263999999 | 0,265300006 | 0,273699999 | 0,270599991 | 0,248999998 | 0,264999986 | 0,259299994 | 0,268900007 | 0,26699999 | 0,261400014 |
| 80 | 0,251100004 | 0,251199991 | 0,273099989 | | 0,269499987 | 0,270300001 | 0,271499991 | 0,281699985 | 0,276899993 | 0,251899987 | 0,270300001 | 0,263300002 | 0,275999993 | 0,272000015 | 0,266099989 |
| 100 | 0,250699997 | 0,252400011 | 0,280200005 | | 0,27700001 | 0,27700001 | 0,278200001 | 0,291200012 | 0,283300012 | 0,252900004 | 0,27669999 | 0,2685 | 0,283300012 | 0,280000001 | 0,272100002 |
| 120 | 0,250499994 | 0,252200007 | 0,287999988 | | 0,284299999 | 0,282499999 | 0,283699989 | 0,298099995 | 0,293099999 | 0,254799992 | 0,282099992 | 0,273000002 | 0,289499998 | 0,286300004 | 0,27759999 |
| 140 | 0,248899996 | 0,25029999 | 0,293500006 | | 0,288700014 | 0,289099991 | 0,289499998 | 0,307500005 | 0,299299985 | 0,256900012 | 0,287099987 | 0,278400004 | 0,296799988 | 0,292899996 | 0,282900006 |
| 160 | 0,249300003 | 0,251100004 | 0,301499993 | | 0,2958 | 0,296099991 | 0,296099991 | 0,315899998 | 0,305700004 | 0,258599997 | 0,293199986 | 0,283399999 | 0,30430001 | 0,300599992 | 0,289000005 |
| 180 | 0,2491 | 0,250200003 | 0,308699995 | | 0,303200006 | 0,302100003 | 0,301800013 | 0,322699994 | 0,31400001 | 0,261000007 | 0,299100012 | 0,288700014 | 0,310600013 | 0,306600004 | 0,29519999 |
| 200 | 0,250200003 | 0,250099987 | 0,316399992 | | 0,308499992 | 0,308899999 | 0,307999998 | 0,330900013 | 0,321399987 | 0,262300014 | 0,305099994 | 0,292600006 | 0,317499995 | 0,31279999 | 0,299699992 |
| 220 | 0,249300003 | 0,250699997 | 0,323399991 | | 0,315200001 | 0,315299988 | 0,314599991 | 0,339100003 | 0,328799993 | 0,264699996 | 0,310000002 | 0,298000008 | 0,324699998 | 0,319999993 | 0,305700004 |
| 240 | 0,250400007 | 0,251199991 | 0,330799997 | | 0,320300013 | 0,32100001 | 0,318699986 | 0,346799999 | 0,335700005 | 0,268000007 | 0,315600008 | 0,302100003 | 0,3301 | 0,327300012 | 0,3125 |
| 260 | 0,248999998 | 0,250999987 | 0,337700009 | | 0,325399995 | 0,327100009 | 0,323799998 | 0,353399992 | 0,342400014 | 0,270300001 | 0,32159999 | 0,306499988 | 0,337799996 | 0,332700014 | 0,316900015 |
| 280 | 0,248899996 | 0,25 | 0,345899999 | | 0,332100004 | 0,332899988 | 0,330500007 | 0,362300009 | 0,349900007 | 0,273600012 | 0,326299995 | 0,312999994 | 0,344199985 | 0,339100003 | 0,32159999 |
| 300 | 0,249699995 | 0,2509 | 0,354400009 | | 0,336699992 | 0,339599997 | 0,336400002 | 0,370499998 | 0,356700003 | 0,275099993 | 0,332500011 | 0,315400004 | 0,350699991 | 0,346300006 | 0,326200008 |
| 320 | 0,248600006 | 0,249899998 | 0,361900002 | | 0,341800004 | 0,345099986 | 0,342200011 | 0,379700005 | 0,364399999 | 0,2773 | 0,337900013 | 0,320300013 | 0,357300013 | 0,351999998 | 0,331099987 |
| 340 | 0,248099998 | 0,250400007 | 0,368999988 | | 0,34889999 | 0,351799995 | 0,348199993 | 0,388700008 | 0,373299986 | 0,280499995 | 0,343199998 | 0,326099992 | 0,365399987 | 0,357300013 | 0,336699992 |
| 360 | 0,247199997 | 0,249500006 | 0,378800005 | | 0,354499996 | 0,35800001 | 0,353500009 | 0,396100014 | 0,379400015 | 0,281199992 | 0,34799999 | 0,32980001 | 0,370099992 | 0,364600003 | 0,341199994 |
| slope | -1,04076E-05 | -7,87924E-06 | 0,000370526 | | 0,000295996 | 0,000303462 | 0,000291914 | 0,000401109 | 0,000364112 | 0,000107389 | 0,000280831 | 0,00023582 | 0,000337699 | 0,000325655 | 0,000269288 |
| slope w/o |  |  | 0,00037967 | | 0,000305139 | 0,000312606 | 0,000301058 | 0,000410253 | 0,000373256 | 0,000116533 | 0,000289974 | 0,000244964 | 0,000346842 | 0,000334799 | 0,000278431 |
| blank | |  |  |  | 0,000342405 |  | 0,000306832 |  | 0,000391754 |  | 0,000203253 |  | 0,000295903 |  | 0,000306615 |
|  | |  |  |  | 0,020544272 |  | 0,018409907 |  | 0,023505264 |  | 0,012195202 |  | 0,017754179 |  | 0,018396904 |
|  | |  |  |  | 3,066309222 |  | 2,747747356 |  | 3,508248337 |  | 1,820179415 |  | 2,649877416 |  | 2,745806549 |
|  | |  |  |  | 21,90220873 |  | 19,62676683 |  | 25,0589167 |  | 13,00128154 |  | 18,92769583 |  | 19,61290392 |
|  | |  | **min -1** |  | 21,90220873 |  | 19,62676683 |  | 25,0589167 |  | 13,00128154 |  | 18,92769583 |  | 19,61290392 |

**5 min**

**PTPN22 reduced control** 21,90220873 **PTPN22 3.4uM H2O2** 19,62676683 **PTPN22 6.3uM H2O2** 25,0589167 **PTPN22 12.5uM H2O2** 13,00128154 **PTPN22 25uM H2O2** 18,92769583 **PTPN22 50uM H2O2** 19,61290392

#DIV/0!

# Device: infinite 200Pro Serial number: 1307001123 Serial number of connected stacker: Firmware: V_3.40_01/15_Infinite (Dec 23 201 MAI, V_3.40_01/15_Infinite (Dec 23 2014/12.45.11)

Date: #########

# Time: 12:51:24

System MTC-MU059-S

# User MTC-MU059-S\fretho

Plate Greiner 96 Flat Bottom Transparent Polystyrene Cat. No.: 655101/655161/655192 [GRE96ft.pdfx] Plate-ID (Stacker)

List of actions in this measurement script: Kinetic

Absorbance

# Label: Label1

Kinetic Measurement

# Kinetic duration 00:06:00

Interval Time 00:00:20

# Measurement Wavelength 405 nm

Bandwidth 10 nm

# Number of Flashes 5

Settle Time 0 ms

# Part of Plate C1-C12; D1-D2

Start Time: 2020 05-18 12:51:26

| Cycle Nr. | 1 | 2 | 3 | 4 | 5 | 6 | 7 | 8 | 9 | 10 | 11 | 12 | 13 | 14 | 15 | 16 | 17 | 18 | 19 |
| --- | --- | --- | --- | --- | --- | --- | --- | --- | --- | --- | --- | --- | --- | --- | --- | --- | --- | --- | --- |
| Time [s] | 0 | 20 | 40 | 60 | 80 | 100 | 120 | 140 | 160 | 180 | 200 | 220 | 240 | 260 | 280 | 300 | 320 | 340 | 360 |
| Temp. [°C] | 24,1 | 24,4 | 24,5 | 24,2 | 24,4 | 24,2 | 24,4 | 24,3 | 24,3 | 24,5 | 24,3 | 24,2 | 24,2 | 24,3 | 24,3 | 24,3 | 24,2 | 24,5 | 24,2 |
| C1 | 0,2506 | 0,2516 | 0,2585 | 0,2644 | 0,2731 | 0,2802 | 0,288 | 0,2935 | 0,3015 | 0,3087 | 0,3164 | 0,3234 | 0,3308 | 0,3377 | 0,3459 | 0,3544 | 0,3619 | 0,369 | 0,3788 |
| C2 | 0,2494 | 0,2592 | 0,2588 | 0,2633 | 0,2695 | 0,277 | 0,2843 | 0,2887 | 0,2958 | 0,3032 | 0,3085 | 0,3152 | 0,3203 | 0,3254 | 0,3321 | 0,3367 | 0,3418 | 0,3489 | 0,3545 |
| C3 | 0,2471 | 0,2621 | 0,2588 | 0,264 | 0,2703 | 0,277 | 0,2825 | 0,2891 | 0,2961 | 0,3021 | 0,3089 | 0,3153 | 0,321 | 0,3271 | 0,3329 | 0,3396 | 0,3451 | 0,3518 | 0,358 |
| C4 | 0,253 | 0,2562 | 0,2595 | 0,2653 | 0,2715 | 0,2782 | 0,2837 | 0,2895 | 0,2961 | 0,3018 | 0,308 | 0,3146 | 0,3187 | 0,3238 | 0,3305 | 0,3364 | 0,3422 | 0,3482 | 0,3535 |
| C5 | 0,2518 | 0,2604 | 0,268 | 0,2737 | 0,2817 | 0,2912 | 0,2981 | 0,3075 | 0,3159 | 0,3227 | 0,3309 | 0,3391 | 0,3468 | 0,3534 | 0,3623 | 0,3705 | 0,3797 | 0,3887 | 0,3961 |
| C6 | 0,2519 | 0,2556 | 0,2632 | 0,2706 | 0,2769 | 0,2833 | 0,2931 | 0,2993 | 0,3057 | 0,314 | 0,3214 | 0,3288 | 0,3357 | 0,3424 | 0,3499 | 0,3567 | 0,3644 | 0,3733 | 0,3794 |
| C7 | 0,2466 | 0,2456 | 0,2483 | 0,249 | 0,2519 | 0,2529 | 0,2548 | 0,2569 | 0,2586 | 0,261 | 0,2623 | 0,2647 | 0,268 | 0,2703 | 0,2736 | 0,2751 | 0,2773 | 0,2805 | 0,2812 |
| C8 | 0,2515 | 0,2521 | 0,2593 | 0,265 | 0,2703 | 0,2767 | 0,2821 | 0,2871 | 0,2932 | 0,2991 | 0,3051 | 0,31 | 0,3156 | 0,3216 | 0,3263 | 0,3325 | 0,3379 | 0,3432 | 0,348 |
| C9 | 0,248 | 0,2496 | 0,2568 | 0,2593 | 0,2633 | 0,2685 | 0,273 | 0,2784 | 0,2834 | 0,2887 | 0,2926 | 0,298 | 0,3021 | 0,3065 | 0,313 | 0,3154 | 0,3203 | 0,3261 | 0,3298 |
| C10 | 0,2542 | 0,2569 | 0,2635 | 0,2689 | 0,276 | 0,2833 | 0,2895 | 0,2968 | 0,3043 | 0,3106 | 0,3175 | 0,3247 | 0,3301 | 0,3378 | 0,3442 | 0,3507 | 0,3573 | 0,3654 | 0,3701 |
| C11 | 0,2526 | 0,2542 | 0,2627 | 0,267 | 0,272 | 0,28 | 0,2863 | 0,2929 | 0,3006 | 0,3066 | 0,3128 | 0,32 | 0,3273 | 0,3327 | 0,3391 | 0,3463 | 0,352 | 0,3573 | 0,3646 |
| C12 | 0,2489 | 0,2505 | 0,2576 | 0,2614 | 0,2661 | 0,2721 | 0,2776 | 0,2829 | 0,289 | 0,2952 | 0,2997 | 0,3057 | 0,3125 | 0,3169 | 0,3216 | 0,3262 | 0,3311 | 0,3367 | 0,3412 |
| D1 | 0,2516 | 0,2513 | 0,2525 | 0,2511 | 0,2511 | 0,2507 | 0,2505 | 0,2489 | 0,2493 | 0,2491 | 0,2502 | 0,2493 | 0,2504 | 0,249 | 0,2489 | 0,2497 | 0,2486 | 0,2481 | 0,2472 |
| D2 | 0,2532 | 0,2519 | 0,2536 | 0,2525 | 0,2512 | 0,2524 | 0,2522 | 0,2503 | 0,2511 | 0,2502 | 0,2501 | 0,2507 | 0,2512 | 0,251 | 0,25 | 0,2509 | 0,2499 | 0,2504 | 0,2495 |

blank

| 20 | 0,250800014 | 0,249799997 | 0,2509 | 0,252700001 | 0,248899996 | 0,251899987 | 0,252900004 | 0,249300003 | 0,246800005 | 0,256199986 | 0,248300001 | 0,251899987 | 0,245900005 | 0,248199999 |
| --- | --- | --- | --- | --- | --- | --- | --- | --- | --- | --- | --- | --- | --- | --- |
| 40 | 0,249899998 | 0,251899987 | 0,255299985 | 0,258599997 | 0,254099995 | 0,25940001 | 0,257999986 | 0,254599988 | 0,250800014 | 0,266299993 | 0,252099991 | 0,259499997 | 0,246800005 | 0,253199995 |
| 60 | 0,249799997 | 0,251100004 | 0,260600001 | 0,261900008 | 0,258700013 | 0,263599992 | 0,263300002 | 0,259299994 | 0,252700001 | 0,276300013 | 0,254999995 | 0,267399997 | 0,249400005 | 0,256900012 |
| 80 | 0,250200003 | 0,250499994 | 0,268599987 | 0,267500013 | 0,262899995 | 0,26789999 | 0,270200014 | 0,264999986 | 0,257099986 | 0,28670001 | 0,258899987 | 0,27669999 | 0,251399994 | 0,265500009 |
| 100 | 0,250800014 | 0,251399994 | 0,278299987 | 0,275099993 | 0,271400005 | 0,275999993 | 0,279100001 | 0,272899985 | 0,263000011 | 0,299100012 | 0,266099989 | 0,285699993 | 0,256999999 | 0,271299988 |
| 120 | 0,250099987 | 0,251399994 | 0,28459999 | 0,282900006 | 0,27759999 | 0,282900006 | 0,286199987 | 0,278800011 | 0,2685 | 0,309799999 | 0,270999998 | 0,293199986 | 0,258300006 | 0,277399987 |
| 140 | 0,251100004 | 0,25 | 0,292299986 | 0,288300008 | 0,282700002 | 0,289200008 | 0,292400002 | 0,284500003 | 0,272599995 | 0,319099993 | 0,27610001 | 0,300300002 | 0,263399988 | 0,284299999 |
| 160 | 0,249799997 | 0,249799997 | 0,299800009 | 0,294499993 | 0,288599998 | 0,296099991 | 0,299199998 | 0,291000009 | 0,277099997 | 0,328399986 | 0,280000001 | 0,308899999 | 0,266200006 | 0,289099991 |
| 180 | 0,248600006 | 0,25 | 0,307099998 | 0,300500005 | 0,294499993 | 0,30250001 | 0,307799995 | 0,296999991 | 0,280800015 | 0,339599997 | 0,284999996 | 0,318100005 | 0,269499987 | 0,297100008 |
| 200 | 0,249500006 | 0,248600006 | 0,3125 | 0,307200015 | 0,299400002 | 0,307300001 | 0,31189999 | 0,302700013 | 0,2852 | 0,3486 | 0,289499998 | 0,324600011 | 0,272599995 | 0,301200002 |
| 220 | 0,25 | 0,250099987 | 0,321799994 | 0,313800007 | 0,307300001 | 0,315200001 | 0,320899993 | 0,309100002 | 0,289999992 | 0,360799998 | 0,295700014 | 0,334600002 | 0,277099997 | 0,308699995 |
| 240 | 0,2491 | 0,250400007 | 0,328000009 | 0,319900006 | 0,312400013 | 0,321500003 | 0,327800006 | 0,315400004 | 0,295599997 | 0,371399999 | 0,299899995 | 0,341800004 | 0,280200005 | 0,313800007 |
| 260 | 0,248999998 | 0,249699995 | 0,335099995 | 0,326999992 | 0,317099988 | 0,328099996 | 0,334500015 | 0,319799989 | 0,299499989 | 0,381000012 | 0,303900003 | 0,350100011 | 0,283499986 | 0,320899993 |
| 280 | 0,251100004 | 0,251300007 | 0,342599988 | 0,333499998 | 0,324900001 | 0,336499989 | 0,342200011 | 0,327800006 | 0,305400014 | 0,3926 | 0,311399996 | 0,359600008 | 0,287299991 | 0,327800006 |
| 300 | 0,2509 | 0,252200007 | 0,349799991 | 0,339700013 | 0,3301 | 0,341699988 | 0,34799999 | 0,333600014 | 0,309199989 | 0,40259999 | 0,314999998 | 0,367199987 | 0,291399986 | 0,332599998 |
| 320 | 0,251599997 | 0,251100004 | 0,35679999 | 0,347000003 | 0,336499989 | 0,347900003 | 0,354699999 | 0,339899987 | 0,315499991 | 0,412200004 | 0,320800006 | 0,37560001 | 0,293900013 | 0,3389 |
| 340 | 0,249500006 | 0,249799997 | 0,362599999 | 0,351799995 | 0,341800004 | 0,352699995 | 0,360700011 | 0,344199985 | 0,318100005 | 0,422100008 | 0,323199987 | 0,382400006 | 0,296700001 | 0,342099994 |
| 360 | 0,250200003 | 0,251300007 | 0,370599985 | 0,359800011 | 0,34799999 | 0,362899989 | 0,367700011 | 0,350800008 | 0,324299991 | 0,431800008 | 0,329299986 | 0,392699987 | 0,300099999 | 0,350300014 |
| slope | 7,22345E-08 | 7,22706E-08 | 0,000359665 | 0,000318684 | 0,000295062 | 0,000323978 | 0,000345114 | 0,000302761 | 0,000230815 | 0,00051968 | 0,000243787 | 0,000412472 | 0,000168075 | 0,000304267 |
| slope w/o |  |  | 0,000359592 | 0,000318612 | 0,00029499 | 0,000323906 | 0,000345041 | 0,000302688 | 0,000230743 | 0,000519608 | 0,000243715 | 0,000412399 | 0,000168003 | 0,000304195 |

0,000339102 0,000309448 0,000323865 0,000375175 0,000328057 0,000236099

0,020346129 0,018566872 0,019431889 0,022510525 0,019683437 0,014165943

3,036735716 2,771174879 2,900281932 3,359779877 2,937826375 2,114319895

21,6909694 19,79410628 20,71629951 23,99842769 20,98447411 15,10228497

**min -1** 21,6909694 19,79410628 20,71629951 23,99842769 20,98447411 15,10228497

**15 min** 30

**PTPN22 reduced control** 21,6909694

**PTPN22 3.4uM H2O2** 19,79410628

**PTPN22 6.3uM H2O2** 20,71629951 **PTPN22 12.5uM H2O2** 23,99842769 **PTPN22 25uM H2O2** 20,98447411

**PTPN22 50uM H2O2** 15,10228497 #DIV/0!

Date: #########

Time: 13:01:28

System MTC-MU059-S

User MTC-MU059-S\fretho

Plate Greiner 96 Flat Bottom Transparent Polystyrene Cat. No.: 655101/655161/655192 [GRE96ft.pdfx] Plate-ID (Stacker)

List of actions in this measurement script: Kinetic

Absorbance

Label: Label1

Kinetic Measurement

Kinetic duration 00:06:00

Interval Time 00:00:20

Measurement Wavelength 405 nm

Bandwidth 10 nm

Number of Flashes 5

Settle Time 0 ms

Part of Plate C1-C12; D1-D2

Start Time: 2020 05-18 13:01:30

| Cycle Nr. | 1 | 2 | 3 | 4 | 5 | 6 | 7 | 8 | 9 | 10 | 11 | 12 | 13 | 14 | 15 | 16 | 17 | 18 | 19 |
| --- | --- | --- | --- | --- | --- | --- | --- | --- | --- | --- | --- | --- | --- | --- | --- | --- | --- | --- | --- |
| Time [s] | 0 | 20 | 40 | 60 | 80 | 100 | 120 | 140 | 160 | 180 | 200 | 220 | 240 | 260 | 280 | 300 | 320 | 340 | 360 |
| Temp. [°C] | 24,4 | 24,4 | 24,3 | 24,3 | 24,4 | 24,3 | 24,5 | 24,4 | 24,3 | 24,4 | 24,4 | 24,7 | 24,3 | 24,4 | 24,4 | 24,3 | 24,4 | 24,3 | 24,3 |
| C1 | 0,2437 | 0,2509 | 0,2553 | 0,2606 | 0,2686 | 0,2783 | 0,2846 | 0,2923 | 0,2998 | 0,3071 | 0,3125 | 0,3218 | 0,328 | 0,3351 | 0,3426 | 0,3498 | 0,3568 | 0,3626 | 0,3706 |
| C2 | 0,2431 | 0,2527 | 0,2586 | 0,2619 | 0,2675 | 0,2751 | 0,2829 | 0,2883 | 0,2945 | 0,3005 | 0,3072 | 0,3138 | 0,3199 | 0,327 | 0,3335 | 0,3397 | 0,347 | 0,3518 | 0,3598 |
| C3 | 0,2427 | 0,2489 | 0,2541 | 0,2587 | 0,2629 | 0,2714 | 0,2776 | 0,2827 | 0,2886 | 0,2945 | 0,2994 | 0,3073 | 0,3124 | 0,3171 | 0,3249 | 0,3301 | 0,3365 | 0,3418 | 0,348 |
| C4 | 0,2453 | 0,2519 | 0,2594 | 0,2636 | 0,2679 | 0,276 | 0,2829 | 0,2892 | 0,2961 | 0,3025 | 0,3073 | 0,3152 | 0,3215 | 0,3281 | 0,3365 | 0,3417 | 0,3479 | 0,3527 | 0,3629 |
| C5 | 0,2478 | 0,2529 | 0,258 | 0,2633 | 0,2702 | 0,2791 | 0,2862 | 0,2924 | 0,2992 | 0,3078 | 0,3119 | 0,3209 | 0,3278 | 0,3345 | 0,3422 | 0,348 | 0,3547 | 0,3607 | 0,3677 |
| C6 | 0,2449 | 0,2493 | 0,2546 | 0,2593 | 0,265 | 0,2729 | 0,2788 | 0,2845 | 0,291 | 0,297 | 0,3027 | 0,3091 | 0,3154 | 0,3198 | 0,3278 | 0,3336 | 0,3399 | 0,3442 | 0,3508 |
| C7 | 0,2421 | 0,2468 | 0,2508 | 0,2527 | 0,2571 | 0,263 | 0,2685 | 0,2726 | 0,2771 | 0,2808 | 0,2852 | 0,29 | 0,2956 | 0,2995 | 0,3054 | 0,3092 | 0,3155 | 0,3181 | 0,3243 |
| C8 | 0,2472 | 0,2562 | 0,2663 | 0,2763 | 0,2867 | 0,2991 | 0,3098 | 0,3191 | 0,3284 | 0,3396 | 0,3486 | 0,3608 | 0,3714 | 0,381 | 0,3926 | 0,4026 | 0,4122 | 0,4221 | 0,4318 |
| C9 | 0,243 | 0,2483 | 0,2521 | 0,255 | 0,2589 | 0,2661 | 0,271 | 0,2761 | 0,28 | 0,285 | 0,2895 | 0,2957 | 0,2999 | 0,3039 | 0,3114 | 0,315 | 0,3208 | 0,3232 | 0,3293 |
| C10 | 0,2438 | 0,2519 | 0,2595 | 0,2674 | 0,2767 | 0,2857 | 0,2932 | 0,3003 | 0,3089 | 0,3181 | 0,3246 | 0,3346 | 0,3418 | 0,3501 | 0,3596 | 0,3672 | 0,3756 | 0,3824 | 0,3927 |
| C11 | 0,2409 | 0,2459 | 0,2468 | 0,2494 | 0,2514 | 0,257 | 0,2583 | 0,2634 | 0,2662 | 0,2695 | 0,2726 | 0,2771 | 0,2802 | 0,2835 | 0,2873 | 0,2914 | 0,2939 | 0,2967 | 0,3001 |
| C12 | 0,2435 | 0,2482 | 0,2532 | 0,2569 | 0,2655 | 0,2713 | 0,2774 | 0,2843 | 0,2891 | 0,2971 | 0,3012 | 0,3087 | 0,3138 | 0,3209 | 0,3278 | 0,3326 | 0,3389 | 0,3421 | 0,3503 |
| D1 | 0,2488 | 0,2508 | 0,2499 | 0,2498 | 0,2502 | 0,2508 | 0,2501 | 0,2511 | 0,2498 | 0,2486 | 0,2495 | 0,25 | 0,2491 | 0,249 | 0,2511 | 0,2509 | 0,2516 | 0,2495 | 0,2502 |
| D2 | 0,2494 | 0,2498 | 0,2519 | 0,2511 | 0,2505 | 0,2514 | 0,2514 | 0,25 | 0,2498 | 0,25 | 0,2486 | 0,2501 | 0,2504 | 0,2497 | 0,2513 | 0,2522 | 0,2511 | 0,2498 | 0,2513 |

End Time: 2020 05-18 13:07:38

blank

| 20 | 0,25150001 | 0,252700001 | 0,2465 | 0,255400002 | 0,257499993 | 0,260199994 | 0,251599997 | 0,247999996 | 0,250800014 | 0,252000004 | 0,243300006 | 0,247400001 | 0,241600007 | 0,239999995 |
| --- | --- | --- | --- | --- | --- | --- | --- | --- | --- | --- | --- | --- | --- | --- |
| 40 | 0,252600014 | 0,253899992 | 0,253100008 | 0,258700013 | 0,25850001 | 0,260600001 | 0,254200011 | 0,254099995 | 0,256000012 | 0,256500006 | 0,248799995 | 0,252799988 | 0,242899999 | 0,242899999 |
| 60 | 0,252400011 | 0,254099995 | 0,2588 | 0,265700012 | 0,264499992 | 0,267399997 | 0,259000003 | 0,257800013 | 0,261000007 | 0,262600005 | 0,250200003 | 0,254799992 | 0,244399995 | 0,244399995 |
| 80 | 0,25150001 | 0,254000008 | 0,265599996 | 0,271200001 | 0,270700008 | 0,273900002 | 0,263200015 | 0,261500001 | 0,265100002 | 0,267100006 | 0,253699988 | 0,257299989 | 0,245100006 | 0,245000005 |
| 100 | 0,253600001 | 0,254400015 | 0,272000015 | 0,277399987 | 0,278899997 | 0,283100009 | 0,269300014 | 0,268400013 | 0,273699999 | 0,273499995 | 0,257600009 | 0,263200015 | 0,246000007 | 0,247299999 |
| 120 | 0,253100008 | 0,254299998 | 0,277700007 | 0,284299999 | 0,284299999 | 0,288300008 | 0,274300009 | 0,273799986 | 0,27790001 | 0,279199988 | 0,262199998 | 0,2667 | 0,246900007 | 0,247799993 |
| 140 | 0,252299994 | 0,253600001 | 0,282799989 | 0,289400011 | 0,290499985 | 0,294499993 | 0,278899997 | 0,278100014 | 0,282599986 | 0,282799989 | 0,264299989 | 0,269800007 | 0,249699995 | 0,25 |
| 160 | 0,253600001 | 0,254299998 | 0,291099995 | 0,296900004 | 0,297100008 | 0,3028 | 0,284799993 | 0,283399999 | 0,289600015 | 0,288800001 | 0,26910001 | 0,272899985 | 0,252200007 | 0,251399994 |
| 180 | 0,252999991 | 0,253899992 | 0,295899987 | 0,303299993 | 0,304199994 | 0,309899986 | 0,289000005 | 0,287800014 | 0,295300007 | 0,294499993 | 0,271600008 | 0,276300013 | 0,254299998 | 0,253699988 |
| 200 | 0,252299994 | 0,254999995 | 0,302599996 | 0,307700008 | 0,310499996 | 0,316399992 | 0,294800013 | 0,292899996 | 0,299800009 | 0,299499989 | 0,275599986 | 0,280900002 | 0,254999995 | 0,256500006 |
| 220 | 0,253100008 | 0,254500002 | 0,308999985 | 0,314999998 | 0,315800011 | 0,322299987 | 0,299299985 | 0,297600001 | 0,306400001 | 0,3046 | 0,278600007 | 0,282900006 | 0,256599993 | 0,257299989 |
| 240 | 0,252299994 | 0,253399998 | 0,314200014 | 0,320899993 | 0,322699994 | 0,328299999 | 0,303600013 | 0,301899999 | 0,311699986 | 0,309300005 | 0,282299995 | 0,28670001 | 0,257600009 | 0,258700013 |
| 260 | 0,252600014 | 0,253600001 | 0,321200013 | 0,324699998 | 0,328200012 | 0,335500002 | 0,307799995 | 0,306699991 | 0,315899998 | 0,314799994 | 0,285100013 | 0,289299995 | 0,258899987 | 0,259000003 |
| 280 | 0,254599988 | 0,256000012 | 0,328299999 | 0,332899988 | 0,337599993 | 0,344000012 | 0,315200001 | 0,312999994 | 0,323199987 | 0,321099997 | 0,289900005 | 0,294999987 | 0,261599988 | 0,262699991 |
| 300 | 0,250999987 | 0,254099995 | 0,332599998 | 0,337900013 | 0,340499997 | 0,348699987 | 0,318599999 | 0,316399992 | 0,326000005 | 0,324900001 | 0,292299986 | 0,296099991 | 0,261400014 | 0,262199998 |
| 320 | 0,253399998 | 0,255299985 | 0,33950001 | 0,344999999 | 0,3477 | 0,355300009 | 0,324400008 | 0,322299987 | 0,334100008 | 0,330599993 | 0,29550001 | 0,302300006 | 0,263099998 | 0,263900012 |
| 340 | 0,254000008 | 0,254599988 | 0,346700013 | 0,352499992 | 0,354499996 | 0,363200009 | 0,3292 | 0,327699989 | 0,34009999 | 0,337000012 | 0,298999995 | 0,304800004 | 0,266600013 | 0,266499996 |
| 360 | 0,252900004 | 0,255899996 | 0,351799995 | 0,357499987 | 0,360300004 | 0,369300008 | 0,334199995 | 0,330900013 | 0,345200002 | 0,341500014 | 0,301200002 | 0,307500005 | 0,2676 | 0,2676 |
| slope | 2,8689E-06 | 4,73682E-06 | 0,000310052 | 0,000304737 | 0,000314551 | 0,000333937 | 0,000248153 | 0,00024531 | 0,000278555 | 0,000263741 | 0,000171533 | 0,000175929 | 7,72807E-05 | 7,98194E-05 |
| slope w/o |  |  | 0,000306249 | 0,000300934 | 0,000310748 | 0,000330134 | 0,00024435 | 0,000241507 | 0,000274752 | 0,000259938 | 0,00016773 | 0,000172126 | 7,34778E-05 | 7,60166E-05 |

0,000303591 0,000320441 0,000242928 0,000267345 0,000169928 7,47472E-05

0,018215481 0,019226473 0,014575697 0,016040713 0,010195668 0,004484832

2,718728452 2,869622822 2,175477136 2,394136263 1,521741474 0,669377867

19,41948894 20,49730587 15,5391224 17,10097331 10,86958196 4,78127048

**min -1** 19,41948894 20,49730587 15,5391224 17,10097331 10,86958196 4,78127048

**30 min PTPN22 reduced control** 19,41948894 **PTPN22 3.4uM H2O2** 20,49730587

**PTPN22 6.3uM H2O2** 15,5391224

**PTPN22 12.5uM H2O2** 17,10097331

**PTPN22 25uM H2O2** 10,86958196

**PTPN22 50uM H2O2** 4,78127048

Date: #########

Time: 13:16:27

System MTC-MU059-S

User MTC-MU059-S\fretho

Plate Greiner 96 Flat Bottom Transparent Polystyrene Cat. No.: 655101/655161/655192 [GRE96ft.pdfx] Plate-ID (Stacker)

List of actions in this measurement script: Kinetic

Absorbance

Label: Label1

Kinetic Measurement

Kinetic duration 00:06:00

Interval Time 00:00:20

Measurement Wavelength 405 nm

Bandwidth 10 nm

Number of Flashes 5

Settle Time 0 ms

Part of Plate C1-C12; D1-D2

Start Time: 2020 05-18 13:16:29

| Cycle Nr. | 1 | 2 | 3 | 4 | 5 | 6 | 7 | 8 | 9 | 10 | 11 | 12 | 13 | 14 | 15 | 16 | 17 | 18 | 19 |
| --- | --- | --- | --- | --- | --- | --- | --- | --- | --- | --- | --- | --- | --- | --- | --- | --- | --- | --- | --- |
| Time [s] | 0 | 20 | 40 | 60 | 80 | 100 | 120 | 140 | 160 | 180 | 200 | 220 | 240 | 260 | 280 | 300 | 320 | 340 | 360 |
| Temp. [°C] | 24,4 | 24,4 | 24,3 | 24,3 | 24,7 | 24,4 | 24,5 | 24,3 | 24,8 | 24,6 | 24,4 | 24,4 | 24,5 | 24,4 | 24,5 | 24,6 | 24,4 | 25 | 24,5 |
| C1 | 0,245 | 0,2465 | 0,2531 | 0,2588 | 0,2656 | 0,272 | 0,2777 | 0,2828 | 0,2911 | 0,2959 | 0,3026 | 0,309 | 0,3142 | 0,3212 | 0,3283 | 0,3326 | 0,3395 | 0,3467 | 0,3518 |
| C2 | 0,2496 | 0,2554 | 0,2587 | 0,2657 | 0,2712 | 0,2774 | 0,2843 | 0,2894 | 0,2969 | 0,3033 | 0,3077 | 0,315 | 0,3209 | 0,3247 | 0,3329 | 0,3379 | 0,345 | 0,3525 | 0,3575 |
| C3 | 0,2461 | 0,2575 | 0,2585 | 0,2645 | 0,2707 | 0,2789 | 0,2843 | 0,2905 | 0,2971 | 0,3042 | 0,3105 | 0,3158 | 0,3227 | 0,3282 | 0,3376 | 0,3405 | 0,3477 | 0,3545 | 0,3603 |
| C4 | 0,2492 | 0,2602 | 0,2606 | 0,2674 | 0,2739 | 0,2831 | 0,2883 | 0,2945 | 0,3028 | 0,3099 | 0,3164 | 0,3223 | 0,3283 | 0,3355 | 0,344 | 0,3487 | 0,3553 | 0,3632 | 0,3693 |
| C5 | 0,2459 | 0,2516 | 0,2542 | 0,259 | 0,2632 | 0,2693 | 0,2743 | 0,2789 | 0,2848 | 0,289 | 0,2948 | 0,2993 | 0,3036 | 0,3078 | 0,3152 | 0,3186 | 0,3244 | 0,3292 | 0,3342 |
| C6 | 0,2465 | 0,248 | 0,2541 | 0,2578 | 0,2615 | 0,2684 | 0,2738 | 0,2781 | 0,2834 | 0,2878 | 0,2929 | 0,2976 | 0,3019 | 0,3067 | 0,313 | 0,3164 | 0,3223 | 0,3277 | 0,3309 |
| C7 | 0,2474 | 0,2508 | 0,256 | 0,261 | 0,2651 | 0,2737 | 0,2779 | 0,2826 | 0,2896 | 0,2953 | 0,2998 | 0,3064 | 0,3117 | 0,3159 | 0,3232 | 0,326 | 0,3341 | 0,3401 | 0,3452 |
| C8 | 0,248 | 0,252 | 0,2565 | 0,2626 | 0,2671 | 0,2735 | 0,2792 | 0,2828 | 0,2888 | 0,2945 | 0,2995 | 0,3046 | 0,3093 | 0,3148 | 0,3211 | 0,3249 | 0,3306 | 0,337 | 0,3415 |
| C9 | 0,2424 | 0,2433 | 0,2488 | 0,2502 | 0,2537 | 0,2576 | 0,2622 | 0,2643 | 0,2691 | 0,2716 | 0,2756 | 0,2786 | 0,2823 | 0,2851 | 0,2899 | 0,2923 | 0,2955 | 0,299 | 0,3012 |
| C10 | 0,2475 | 0,2474 | 0,2528 | 0,2548 | 0,2573 | 0,2632 | 0,2667 | 0,2698 | 0,2729 | 0,2763 | 0,2809 | 0,2829 | 0,2867 | 0,2893 | 0,295 | 0,2961 | 0,3023 | 0,3048 | 0,3075 |
| C11 | 0,2411 | 0,2416 | 0,2429 | 0,2444 | 0,2451 | 0,246 | 0,2469 | 0,2497 | 0,2522 | 0,2543 | 0,255 | 0,2566 | 0,2576 | 0,2589 | 0,2616 | 0,2614 | 0,2631 | 0,2666 | 0,2676 |
| C12 | 0,2442 | 0,24 | 0,2429 | 0,2444 | 0,245 | 0,2473 | 0,2478 | 0,25 | 0,2514 | 0,2537 | 0,2565 | 0,2573 | 0,2587 | 0,259 | 0,2627 | 0,2622 | 0,2639 | 0,2665 | 0,2676 |
| D1 | 0,2531 | 0,2515 | 0,2526 | 0,2524 | 0,2515 | 0,2536 | 0,2531 | 0,2523 | 0,2536 | 0,253 | 0,2523 | 0,2531 | 0,2523 | 0,2526 | 0,2546 | 0,251 | 0,2534 | 0,254 | 0,2529 |
| D2 | 0,2542 | 0,2527 | 0,2539 | 0,2541 | 0,254 | 0,2544 | 0,2543 | 0,2536 | 0,2543 | 0,2539 | 0,255 | 0,2545 | 0,2534 | 0,2536 | 0,256 | 0,2541 | 0,2553 | 0,2546 | 0,2559 |

End Time: 2020 05-18 13:22:37

| 20 | 0,255499989 | 0,2563 | 0,261599988 | | 0,270799994 | 0,262800008 | 0,262199998 | 0,262899995 | 0,264899999 | 0,263200015 | 0,265399992 | 0,261799991 | 0,260399997 | 0,260399997 | 0,259600013 |
| --- | --- | --- | --- | --- | --- | --- | --- | --- | --- | --- | --- | --- | --- | --- | --- |
| 40 | 0,254500002 | 0,2561 | 0,267100006 | | 0,267699987 | 0,270999998 | 0,268400013 | 0,269899994 | 0,271100014 | 0,2685 | 0,270900011 | 0,266799986 | 0,266499996 | 0,265300006 | 0,264899999 |
| 60 | 0,256099999 | 0,25470001 | 0,272899985 | | 0,275000006 | 0,276300013 | 0,273900002 | 0,2755 | 0,277999997 | 0,275200009 | 0,277099997 | 0,271899998 | 0,271400005 | 0,269300014 | 0,269499987 |
| 80 | 0,254599988 | 0,25619999 | 0,280900002 | | 0,281100005 | 0,283499986 | 0,280299991 | 0,281500012 | 0,285699993 | 0,282799989 | 0,284799993 | 0,27790001 | 0,277200013 | 0,275099993 | 0,27669999 |
| 100 | 0,254900008 | 0,25470001 | 0,287099987 | | 0,286799997 | 0,289799988 | 0,287400007 | 0,288100004 | 0,292199999 | 0,289000005 | 0,291399986 | 0,2852 | 0,283199996 | 0,282400012 | 0,282200009 |
| 120 | 0,255100012 | 0,25510001 | 0,29519999 | | 0,294400007 | 0,297800004 | 0,294 | 0,294200003 | 0,300300002 | 0,295899987 | 0,298599988 | 0,290399998 | 0,290600002 | 0,288399994 | 0,289900005 |
| 140 | 0,254900008 | 0,25420001 | 0,301899999 | | 0,301099986 | 0,3037 | 0,300000012 | 0,300199986 | 0,305799991 | 0,303900003 | 0,305299997 | 0,296499997 | 0,29519999 | 0,292199999 | 0,29519999 |
| 160 | 0,255699992 | 0,2563 | 0,308999985 | | 0,306699991 | 0,310699999 | 0,307000011 | 0,307700008 | 0,313300014 | 0,310400009 | 0,312000006 | 0,303000003 | 0,302100003 | 0,300099999 | 0,3028 |
| 180 | 0,254700005 | 0,25459999 | 0,314500004 | | 0,314200014 | 0,316799998 | 0,312599987 | 0,315400004 | 0,320199996 | 0,316700011 | 0,318699986 | 0,308200002 | 0,307500005 | 0,304100007 | 0,307099998 |
| 200 | 0,254299998 | 0,2538 | 0,323000014 | | 0,320100009 | 0,324000001 | 0,319400012 | 0,3213 | 0,326299995 | 0,32280001 | 0,325399995 | 0,313300014 | 0,312299997 | 0,309899986 | 0,313600004 |
| 220 | 0,255199999 | 0,25529999 | 0,329699993 | | 0,326700002 | 0,330799997 | 0,327399999 | 0,327199996 | 0,333900005 | 0,330199987 | 0,333099991 | 0,320300013 | 0,318800002 | 0,316000015 | 0,319999993 |
| 240 | 0,254799992 | 0,255 | 0,335299999 | | 0,333200008 | 0,338200003 | 0,333999991 | 0,333299994 | 0,340000004 | 0,337599993 | 0,33919999 | 0,326299995 | 0,325199991 | 0,32159999 | 0,326299995 |
| 260 | 0,254900008 | 0,2543 | 0,342000008 | | 0,33919999 | 0,344700009 | 0,33919999 | 0,339399993 | 0,348699987 | 0,342700005 | 0,346300006 | 0,331800014 | 0,331099987 | 0,326000005 | 0,332100004 |
| 280 | 0,252400011 | 0,2543 | 0,34799999 | | 0,344900012 | 0,350300014 | 0,344300002 | 0,344599992 | 0,354200006 | 0,34889999 | 0,351500005 | 0,336299986 | 0,333700001 | 0,330799997 | 0,336600006 |
| 300 | 0,253399998 | 0,25369999 | 0,354799986 | | 0,350600004 | 0,356000006 | 0,350199997 | 0,351099998 | 0,358500004 | 0,355300009 | 0,35769999 | 0,342799991 | 0,340299994 | 0,337399989 | 0,342000008 |
| 320 | 0,253600001 | 0,25420001 | 0,360500008 | | 0,356599987 | 0,363000005 | 0,356999993 | 0,358399987 | 0,36590001 | 0,362599999 | 0,365399987 | 0,348199993 | 0,346700013 | 0,342599988 | 0,347200006 |
| 340 | 0,253100008 | 0,25440001 | 0,367799997 | | 0,362899989 | 0,369500011 | 0,363499999 | 0,363000005 | 0,372000009 | 0,369100004 | 0,371800005 | 0,354200006 | 0,352800012 | 0,348699987 | 0,354200006 |
| 360 | 0,253199995 | 0,252 | 0,372999996 | | 0,369100004 | 0,37560001 | 0,368200004 | 0,368800014 | 0,378800005 | 0,374799997 | 0,376800001 | 0,358900011 | 0,357300013 | 0,352299988 | 0,358500004 |
| slope | -6,81629E-06 | -7,3065E-06 | 0,000333813 | | 0,000308942 | 0,000331914 | 0,000317038 | 0,000314494 | 0,000336894 | 0,000332332 | 0,000333922 | 0,000290155 | 0,000286775 | 0,000276099 | 0,000296584 |
| slope w/o |  |  | 0,000340875 | | 0,000316004 | 0,000338976 | 0,0003241 | 0,000321556 | 0,000343955 | 0,000339394 | 0,000340983 | 0,000297216 | 0,000293836 | 0,00028316 | 0,000303646 |
| blank | |  |  |  | 0,000328439 |  | 0,000331538 |  | 0,000332755 |  | 0,000340188 |  | 0,000295526 |  | 0,000293403 |
|  | |  |  |  | 0,019706347 |  | 0,01989226 |  | 0,019965325 |  | 0,020411299 |  | 0,017731579 |  | 0,017604178 |
|  | |  |  |  | 2,941245792 |  | 2,968994012 |  | 2,97989925 |  | 3,046462609 |  | 2,646504398 |  | 2,627489281 |
|  | |  |  |  | 21,00889852 |  | 21,20710008 |  | 21,28499465 |  | 21,7604472 |  | 18,90360284 |  | 18,76778058 |
|  | |  | **min -1** |  | 21,00889852 |  | 21,20710008 |  | 21,28499465 |  | 21,7604472 |  | 18,90360284 |  | 18,76778058 |

**5 min PTPN22 reduced control** 21,0088985 **PTPN22 3.4uM H2O2** 21,2071001 **PTPN22 6.3uM H2O2** 21,2849946 **PTPN22 12.5uM H2O2** 21,7604472 **PTPN22 25uM H2O2** 18,9036028 **PTPN22 50uM H2O2** 18,7677806

Date: #########

Time: 13:29:27

System MTC-MU059-S

User MTC-MU059-S\fretho

Plate Greiner 96 Flat Bottom Transparent Polystyrene Cat. No.: 655101/655161/655192 [GRE96ft.pdfx] Plate-ID (Stacker)

List of actions in this measurement script: Kinetic

Absorbance

Label: Label1

Kinetic Measurement

Kinetic duration 00:06:00

Interval Time 00:00:20

Measurement Wavelength 405 nm

Bandwidth 10 nm

Number of Flashes 5

Settle Time 0 ms

Part of Plate E1-E12; F1-F2

Start Time: 2020 05-18 13:29:30

| Cycle Nr. | 1 | 2 | 3 | 4 | 5 | 6 | 7 | 8 | 9 | 10 | 11 | 12 | 13 | 14 | 15 | 16 | 17 | 18 | 19 |
| --- | --- | --- | --- | --- | --- | --- | --- | --- | --- | --- | --- | --- | --- | --- | --- | --- | --- | --- | --- |
| Time [s] | 0 | 20 | 40 | 60 | 80 | 100 | 120 | 140 | 160 | 180 | 200 | 220 | 240 | 260 | 280 | 300 | 320 | 340 | 360 |
| Temp. [°C] | 24,5 | 24,6 | 24,7 | 24,8 | 24,5 | 24,6 | 24,6 | 25 | 24,5 | 24,5 | 24,7 | 24,7 | 24,7 | 24,7 | 24,6 | 24,8 | 25,2 | 24,8 | 24,8 |
| E1 | 0,2602 | 0,2616 | 0,2671 | 0,2729 | 0,2809 | 0,2871 | 0,2952 | 0,3019 | 0,309 | 0,3145 | 0,323 | 0,3297 | 0,3353 | 0,342 | 0,348 | 0,3548 | 0,3605 | 0,3678 | 0,373 |
| E2 | 0,2596 | 0,2708 | 0,2677 | 0,275 | 0,2811 | 0,2868 | 0,2944 | 0,3011 | 0,3067 | 0,3142 | 0,3201 | 0,3267 | 0,3332 | 0,3392 | 0,3449 | 0,3506 | 0,3566 | 0,3629 | 0,3691 |
| E3 | 0,2594 | 0,2628 | 0,271 | 0,2763 | 0,2835 | 0,2898 | 0,2978 | 0,3037 | 0,3107 | 0,3168 | 0,324 | 0,3308 | 0,3382 | 0,3447 | 0,3503 | 0,356 | 0,363 | 0,3695 | 0,3756 |
| E4 | 0,2695 | 0,2622 | 0,2684 | 0,2739 | 0,2803 | 0,2874 | 0,294 | 0,3 | 0,307 | 0,3126 | 0,3194 | 0,3274 | 0,334 | 0,3392 | 0,3443 | 0,3502 | 0,357 | 0,3635 | 0,3682 |
| E5 | 0,261 | 0,2629 | 0,2699 | 0,2755 | 0,2815 | 0,2881 | 0,2942 | 0,3002 | 0,3077 | 0,3154 | 0,3213 | 0,3272 | 0,3333 | 0,3394 | 0,3446 | 0,3511 | 0,3584 | 0,363 | 0,3688 |
| E6 | 0,26 | 0,2649 | 0,2711 | 0,278 | 0,2857 | 0,2922 | 0,3003 | 0,3058 | 0,3133 | 0,3202 | 0,3263 | 0,3339 | 0,34 | 0,3487 | 0,3542 | 0,3585 | 0,3659 | 0,372 | 0,3788 |
| E7 | 0,2678 | 0,2632 | 0,2685 | 0,2752 | 0,2828 | 0,289 | 0,2959 | 0,3039 | 0,3104 | 0,3167 | 0,3228 | 0,3302 | 0,3376 | 0,3427 | 0,3489 | 0,3553 | 0,3626 | 0,3691 | 0,3748 |
| E8 | 0,2599 | 0,2654 | 0,2709 | 0,2771 | 0,2848 | 0,2914 | 0,2986 | 0,3053 | 0,312 | 0,3187 | 0,3254 | 0,3331 | 0,3392 | 0,3463 | 0,3515 | 0,3577 | 0,3654 | 0,3718 | 0,3768 |
| E9 | 0,2574 | 0,2618 | 0,2668 | 0,2719 | 0,2779 | 0,2852 | 0,2904 | 0,2965 | 0,303 | 0,3082 | 0,3133 | 0,3203 | 0,3263 | 0,3318 | 0,3363 | 0,3428 | 0,3482 | 0,3542 | 0,3589 |
| E10 | 0,2551 | 0,2604 | 0,2665 | 0,2714 | 0,2772 | 0,2832 | 0,2906 | 0,2952 | 0,3021 | 0,3075 | 0,3123 | 0,3188 | 0,3252 | 0,3311 | 0,3337 | 0,3403 | 0,3467 | 0,3528 | 0,3573 |
| E11 | 0,2564 | 0,2604 | 0,2653 | 0,2693 | 0,2751 | 0,2824 | 0,2884 | 0,2922 | 0,3001 | 0,3041 | 0,3099 | 0,316 | 0,3216 | 0,326 | 0,3308 | 0,3374 | 0,3426 | 0,3487 | 0,3523 |
| E12 | 0,2529 | 0,2596 | 0,2649 | 0,2695 | 0,2767 | 0,2822 | 0,2899 | 0,2952 | 0,3028 | 0,3071 | 0,3136 | 0,32 | 0,3263 | 0,3321 | 0,3366 | 0,342 | 0,3472 | 0,3542 | 0,3585 |
| F1 | 0,2554 | 0,2555 | 0,2545 | 0,2561 | 0,2546 | 0,2549 | 0,2551 | 0,2549 | 0,2557 | 0,2547 | 0,2543 | 0,2552 | 0,2548 | 0,2549 | 0,2524 | 0,2534 | 0,2536 | 0,2531 | 0,2532 |
| F2 | 0,2568 | 0,2563 | 0,2561 | 0,2547 | 0,2562 | 0,2547 | 0,2551 | 0,2542 | 0,2563 | 0,2546 | 0,2538 | 0,2553 | 0,255 | 0,2543 | 0,2543 | 0,2537 | 0,2542 | 0,2544 | 0,252 |

End Time: 2020 05-18 13:35:38

blank

| 20 | 0,254599988 | 0,255699992 | 0,254099995 | 0,256700009 | 0,259600013 | 0,254700005 | 0,258100003 | 0,254200011 | 0,252400011 | 0,253699988 | 0,252600014 | 0,252299994 | 0,250499994 | 0,248400003 |
| --- | --- | --- | --- | --- | --- | --- | --- | --- | --- | --- | --- | --- | --- | --- |
| 40 | 0,253100008 | 0,254500002 | 0,257999986 | 0,258100003 | 0,256999999 | 0,259799987 | 0,262699991 | 0,259299994 | 0,257800013 | 0,257999986 | 0,254000008 | 0,255699992 | 0,253399998 | 0,250999987 |
| 60 | 0,255299985 | 0,256700009 | 0,266299993 | 0,26820001 | 0,265399992 | 0,26789999 | 0,269300014 | 0,269199997 | 0,264200002 | 0,266499996 | 0,259900004 | 0,261299998 | 0,258700013 | 0,256999999 |
| 80 | 0,254200011 | 0,255100012 | 0,271800011 | 0,273400009 | 0,270700008 | 0,274199992 | 0,273900002 | 0,275299996 | 0,270599991 | 0,271100014 | 0,264800012 | 0,265199989 | 0,262499988 | 0,262400001 |
| 100 | 0,255400002 | 0,255800009 | 0,281399995 | 0,279399991 | 0,2764 | 0,281500012 | 0,281399995 | 0,280600011 | 0,277700007 | 0,278200001 | 0,270900011 | 0,272799999 | 0,267699987 | 0,265199989 |
| 120 | 0,255400002 | 0,256300002 | 0,289299995 | 0,28670001 | 0,283300012 | 0,288899988 | 0,287999988 | 0,287900001 | 0,283699989 | 0,286199987 | 0,27669999 | 0,27759999 | 0,273200005 | 0,271100014 |
| 140 | 0,255299985 | 0,257299989 | 0,294800013 | 0,292899996 | 0,289499998 | 0,29460001 | 0,295599997 | 0,294699997 | 0,290100008 | 0,290300012 | 0,280099988 | 0,282200009 | 0,27759999 | 0,273499995 |
| 160 | 0,254999995 | 0,255400002 | 0,300900012 | 0,299699992 | 0,294699997 | 0,301400006 | 0,300900012 | 0,302399993 | 0,295700014 | 0,296400011 | 0,284700006 | 0,288300008 | 0,281100005 | 0,27759999 |
| 180 | 0,254999995 | 0,255499989 | 0,308200002 | 0,305700004 | 0,302399993 | 0,308299989 | 0,307999998 | 0,308600008 | 0,303000003 | 0,30250001 | 0,289299995 | 0,293000013 | 0,285600007 | 0,281199992 |
| 200 | 0,255400002 | 0,255199999 | 0,314500004 | 0,3125 | 0,309599996 | 0,315800011 | 0,314799994 | 0,316399992 | 0,307900012 | 0,308699995 | 0,294499993 | 0,297800004 | 0,287900001 | 0,28490001 |
| 220 | 0,255199999 | 0,256000012 | 0,322100013 | 0,319900006 | 0,315200001 | 0,322100013 | 0,32100001 | 0,32159999 | 0,314599991 | 0,314500004 | 0,298900008 | 0,303799987 | 0,293099999 | 0,289900005 |
| 240 | 0,254700005 | 0,255699992 | 0,328500003 | 0,326099992 | 0,321099997 | 0,329899997 | 0,328399986 | 0,327899992 | 0,321399987 | 0,320600003 | 0,303900003 | 0,308299989 | 0,297399998 | 0,293500006 |
| 260 | 0,254700005 | 0,253899992 | 0,335900009 | 0,333099991 | 0,327600002 | 0,335700005 | 0,333400011 | 0,334699988 | 0,326799989 | 0,326599985 | 0,308699995 | 0,313499987 | 0,300199986 | 0,297100008 |
| 280 | 0,253500015 | 0,254700005 | 0,342400014 | 0,339899987 | 0,333900005 | 0,342900008 | 0,341199994 | 0,341600001 | 0,332599998 | 0,331499994 | 0,31279999 | 0,317499995 | 0,305200011 | 0,300700009 |
| 300 | 0,255699992 | 0,254299998 | 0,350199997 | 0,346599996 | 0,33950001 | 0,350499988 | 0,347799987 | 0,349400014 | 0,339399993 | 0,33919999 | 0,319499999 | 0,325300008 | 0,309599996 | 0,304899991 |
| 320 | 0,254500002 | 0,254000008 | 0,3574 | 0,353399992 | 0,346500009 | 0,3583 | 0,355800003 | 0,356999993 | 0,34709999 | 0,344199985 | 0,324400008 | 0,329699993 | 0,314399987 | 0,309799999 |
| 340 | 0,254700005 | 0,253800005 | 0,36500001 | 0,359899998 | 0,353799999 | 0,365500003 | 0,363400012 | 0,362599999 | 0,352600008 | 0,351500005 | 0,328700006 | 0,334600002 | 0,318699986 | 0,312299997 |
| 360 | 0,255199999 | 0,254099995 | 0,372200012 | 0,367500007 | 0,360700011 | 0,371800005 | 0,370099992 | 0,370299995 | 0,359800011 | 0,357899994 | 0,333999991 | 0,340000004 | 0,3222 | 0,317400008 |
| slope | 8,41093E-07 | -5,92364E-06 | 0,000349484 | 0,000331584 | 0,00031146 | 0,000346594 | 0,00033225 | 0,000339923 | 0,000314561 | 0,00030483 | 0,000243158 | 0,000261099 | 0,000211785 | 0,000200645 |
| slope w/o |  |  | 0,000352025 | 0,000334125 | 0,000314002 | 0,000349136 | 0,000334791 | 0,000342464 | 0,000317103 | 0,000307371 | 0,000245699 | 0,00026364 | 0,000214327 | 0,000203186 |

0,000343075 0,000331569 0,000338627 0,000312237 0,00025467 0,000208756

0,02058452 0,019894118 0,020317646 0,018734209 0,015280185 0,012525387

3,072316416 2,969271364 3,032484485 2,796150616 2,2806246 1,869460718

21,94511726 21,20908117 21,66060346 19,9725044 16,29017572 13,35329084

**min -1** 21,94511726 21,20908117 21,66060346 19,9725044 16,29017572 13,35329084

**15 min PTPN22 reduced control** 21,94511726 **PTPN22 3.4uM H2O2** 21,20908117 **PTPN22 6.3uM H2O2** 21,66060346 **PTPN22 12.5uM H2O2** 19,9725044 **PTPN22 25uM H2O2** 16,29017572

**PTPN22 50uM H2O2** 13,35329084

Date: #########

Time: 13:39:23

System MTC-MU059-S

User MTC-MU059-S\fretho

Plate Greiner 96 Flat Bottom Transparent Polystyrene Cat. No.: 655101/655161/655192 [GRE96ft.pdfx] Plate-ID (Stacker)

List of actions in this measurement script: Kinetic

Absorbance

Label: Label1

Kinetic Measurement

Kinetic duration 00:06:00

Interval Time 00:00:20

Measurement Wavelength 405 nm

Bandwidth 10 nm

Number of Flashes 5

Settle Time 0 ms

Part of Plate E1-E12; F1-F2

Start Time: 2020 05-18 13:39:25

| Cycle Nr. | 1 | 2 | 3 | 4 | 5 | 6 | 7 | 8 | 9 | 10 | 11 | 12 | 13 | 14 | 15 | 16 | 17 | 18 | 19 |
| --- | --- | --- | --- | --- | --- | --- | --- | --- | --- | --- | --- | --- | --- | --- | --- | --- | --- | --- | --- |
| Time [s] | 0 | 20 | 40 | 60 | 80 | 100 | 120 | 140 | 160 | 180 | 200 | 220 | 240 | 260 | 280 | 300 | 320 | 340 | 360 |
| Temp. [°C] | 24,7 | 24,8 | 24,8 | 24,8 | 25,2 | 25,1 | 25,1 | 25,2 | 25,2 | 25,2 | 25,3 | 25,2 | 25,4 | 25,1 | 25,1 | 25,1 | 25,3 | 25,4 | 25,4 |
| E1 | 0,2517 | 0,2541 | 0,258 | 0,2663 | 0,2718 | 0,2814 | 0,2893 | 0,2948 | 0,3009 | 0,3082 | 0,3145 | 0,3221 | 0,3285 | 0,3359 | 0,3424 | 0,3502 | 0,3574 | 0,365 | 0,3722 |
| E2 | 0,253 | 0,2567 | 0,2581 | 0,2682 | 0,2734 | 0,2794 | 0,2867 | 0,2929 | 0,2997 | 0,3057 | 0,3125 | 0,3199 | 0,3261 | 0,3331 | 0,3399 | 0,3466 | 0,3534 | 0,3599 | 0,3675 |
| E3 | 0,2517 | 0,2596 | 0,257 | 0,2654 | 0,2707 | 0,2764 | 0,2833 | 0,2895 | 0,2947 | 0,3024 | 0,3096 | 0,3152 | 0,3211 | 0,3276 | 0,3339 | 0,3395 | 0,3465 | 0,3538 | 0,3607 |
| E4 | 0,2502 | 0,2547 | 0,2598 | 0,2679 | 0,2742 | 0,2815 | 0,2889 | 0,2946 | 0,3014 | 0,3083 | 0,3158 | 0,3221 | 0,3299 | 0,3357 | 0,3429 | 0,3505 | 0,3583 | 0,3655 | 0,3718 |
| E5 | 0,2556 | 0,2581 | 0,2627 | 0,2693 | 0,2739 | 0,2814 | 0,288 | 0,2956 | 0,3009 | 0,308 | 0,3148 | 0,321 | 0,3284 | 0,3334 | 0,3412 | 0,3478 | 0,3558 | 0,3634 | 0,3701 |
| E6 | 0,2495 | 0,2542 | 0,2593 | 0,2692 | 0,2753 | 0,2806 | 0,2879 | 0,2947 | 0,3024 | 0,3086 | 0,3164 | 0,3216 | 0,3279 | 0,3347 | 0,3416 | 0,3494 | 0,357 | 0,3626 | 0,3703 |
| E7 | 0,2489 | 0,2524 | 0,2578 | 0,2642 | 0,2706 | 0,2777 | 0,2837 | 0,2901 | 0,2957 | 0,303 | 0,3079 | 0,3146 | 0,3214 | 0,3268 | 0,3326 | 0,3394 | 0,3471 | 0,3526 | 0,3598 |
| E8 | 0,2505 | 0,2537 | 0,258 | 0,2665 | 0,2711 | 0,2782 | 0,2862 | 0,2903 | 0,2964 | 0,3025 | 0,3087 | 0,3145 | 0,3206 | 0,3266 | 0,3315 | 0,3392 | 0,3442 | 0,3515 | 0,3579 |
| E9 | 0,25 | 0,2526 | 0,254 | 0,2599 | 0,2648 | 0,2709 | 0,2767 | 0,2801 | 0,2847 | 0,2893 | 0,2945 | 0,2989 | 0,3039 | 0,3087 | 0,3128 | 0,3195 | 0,3244 | 0,3287 | 0,334 |
| E10 | 0,248 | 0,2523 | 0,2557 | 0,2613 | 0,2652 | 0,2728 | 0,2776 | 0,2822 | 0,2883 | 0,293 | 0,2978 | 0,3038 | 0,3083 | 0,3135 | 0,3175 | 0,3253 | 0,3297 | 0,3346 | 0,34 |
| E11 | 0,2499 | 0,2505 | 0,2534 | 0,2587 | 0,2625 | 0,2677 | 0,2732 | 0,2776 | 0,2811 | 0,2856 | 0,2879 | 0,2931 | 0,2974 | 0,3002 | 0,3052 | 0,3096 | 0,3144 | 0,3187 | 0,3222 |
| E12 | 0,2459 | 0,2484 | 0,251 | 0,257 | 0,2624 | 0,2652 | 0,2711 | 0,2735 | 0,2776 | 0,2812 | 0,2849 | 0,2899 | 0,2935 | 0,2971 | 0,3007 | 0,3049 | 0,3098 | 0,3123 | 0,3174 |
| F1 | 0,2542 | 0,2546 | 0,2531 | 0,2553 | 0,2542 | 0,2554 | 0,2554 | 0,2553 | 0,255 | 0,255 | 0,2554 | 0,2552 | 0,2547 | 0,2547 | 0,2535 | 0,2557 | 0,2545 | 0,2547 | 0,2552 |
| F2 | 0,2545 | 0,2557 | 0,2545 | 0,2567 | 0,2551 | 0,2558 | 0,2563 | 0,2573 | 0,2554 | 0,2555 | 0,2552 | 0,256 | 0,2557 | 0,2539 | 0,2547 | 0,2543 | 0,254 | 0,2538 | 0,2541 |

End Time: 2020 05-18 13:45:34

blank

| 20 | 0,253699988 | 0,255199999 | 0,255600005 | 0,262100011 | 0,256599993 | 0,255100012 | 0,253500015 | 0,254099995 | 0,253500015 | 0,250999987 | 0,251599997 | 0,253800005 | 0,248500004 | 0,247099996 |
| --- | --- | --- | --- | --- | --- | --- | --- | --- | --- | --- | --- | --- | --- | --- |
| 40 | 0,253500015 | 0,256000012 | 0,261900008 | 0,265100002 | 0,279300004 | 0,261299998 | 0,25999999 | 0,25909999 | 0,2588 | 0,256099999 | 0,255600005 | 0,257400006 | 0,250099987 | 0,248600006 |
| 60 | 0,254099995 | 0,256099999 | 0,268999994 | 0,270599991 | 0,282499999 | 0,266400009 | 0,266000003 | 0,266099989 | 0,262899995 | 0,260699987 | 0,2588 | 0,260899991 | 0,25 | 0,247999996 |
| 80 | 0,253899992 | 0,254900008 | 0,274800003 | 0,27790001 | 0,27610001 | 0,273699999 | 0,272199988 | 0,271800011 | 0,268599987 | 0,2667 | 0,263300002 | 0,264999986 | 0,253600001 | 0,251899987 |
| 100 | 0,255100012 | 0,256000012 | 0,283800006 | 0,283800006 | 0,281100005 | 0,281100005 | 0,279700011 | 0,27790001 | 0,275099993 | 0,272799999 | 0,269699991 | 0,270999998 | 0,255499989 | 0,255499989 |
| 120 | 0,254900008 | 0,255899996 | 0,291099995 | 0,291799992 | 0,289299995 | 0,288199991 | 0,287099987 | 0,2852 | 0,280400008 | 0,279000014 | 0,275200009 | 0,275700003 | 0,25940001 | 0,25940001 |
| 140 | 0,253199995 | 0,254999995 | 0,296400011 | 0,297399998 | 0,293500006 | 0,293599993 | 0,290899992 | 0,289000005 | 0,283800006 | 0,282999992 | 0,27790001 | 0,280699998 | 0,25999999 | 0,260100007 |
| 160 | 0,254400015 | 0,255400002 | 0,304399997 | 0,304699987 | 0,301400006 | 0,301699996 | 0,298599988 | 0,297600001 | 0,290899992 | 0,289999992 | 0,283800006 | 0,28580001 | 0,262899995 | 0,262499988 |
| 180 | 0,254700005 | 0,255600005 | 0,312599987 | 0,311399996 | 0,307300001 | 0,307700008 | 0,305400014 | 0,304399997 | 0,296700001 | 0,294200003 | 0,289400011 | 0,289799988 | 0,264999986 | 0,266000003 |
| 200 | 0,255600005 | 0,256099999 | 0,318399996 | 0,318899989 | 0,314999998 | 0,314599991 | 0,312099993 | 0,310499996 | 0,302700013 | 0,300700009 | 0,295100003 | 0,295899987 | 0,26789999 | 0,268400013 |
| 220 | 0,255899996 | 0,256599993 | 0,326099992 | 0,325500011 | 0,32159999 | 0,32249999 | 0,319499999 | 0,317699999 | 0,308099985 | 0,307000011 | 0,299299985 | 0,301200002 | 0,270999998 | 0,270500004 |
| 240 | 0,255100012 | 0,255499989 | 0,333400011 | 0,331800014 | 0,32949999 | 0,328099996 | 0,325199991 | 0,323799998 | 0,313699991 | 0,311300009 | 0,303799987 | 0,305000007 | 0,272700012 | 0,272100002 |
| 260 | 0,256500006 | 0,255499989 | 0,340499997 | 0,339100003 | 0,334899992 | 0,336299986 | 0,332100004 | 0,331 | 0,3204 | 0,317600012 | 0,308999985 | 0,311399996 | 0,276199996 | 0,275099993 |
| 280 | 0,254999995 | 0,255400002 | 0,347299993 | 0,345200002 | 0,341500014 | 0,342500001 | 0,338 | 0,337500006 | 0,325300008 | 0,321099997 | 0,31279999 | 0,313800007 | 0,276800007 | 0,276199996 |
| 300 | 0,255899996 | 0,255299985 | 0,354299992 | 0,351500005 | 0,348800004 | 0,350199997 | 0,345499992 | 0,343100011 | 0,330900013 | 0,327499986 | 0,317400008 | 0,318100005 | 0,279500008 | 0,280299991 |
| 320 | 0,254500002 | 0,254099995 | 0,360500008 | 0,358099997 | 0,355300009 | 0,354799986 | 0,351599991 | 0,348500013 | 0,335099995 | 0,332700014 | 0,321999997 | 0,323000014 | 0,280099988 | 0,280999988 |
| 340 | 0,252799988 | 0,253699988 | 0,368400007 | 0,36469999 | 0,361499995 | 0,361900002 | 0,356900007 | 0,354699999 | 0,340000004 | 0,336699992 | 0,326099992 | 0,325800002 | 0,282599986 | 0,283600003 |
| 360 | 0,254599988 | 0,254999995 | 0,377400011 | 0,372200012 | 0,368800014 | 0,369700015 | 0,365999997 | 0,363000005 | 0,34740001 | 0,344000012 | 0,332100004 | 0,332700014 | 0,287 | 0,286599994 |
| slope | 2,95148E-06 | -3,15278E-06 | 0,000356311 | 0,000332219 | 0,000309474 | 0,000339474 | 0,000328467 | 0,000321889 | 0,000277456 | 0,000273411 | 0,000239881 | 0,000235686 | 0,000114458 | 0,000119737 |
| slope w/o |  |  | 0,000356411 | 0,000332319 | 0,000309574 | 0,000339574 | 0,000328568 | 0,000321989 | 0,000277557 | 0,000273511 | 0,000239982 | 0,000235787 | 0,000114559 | 0,000119837 |

0,000344365 0,000324574 0,000325279 0,000275534 0,000237884 0,000117198

0,020661922 0,01947446 0,019516721 0,016532047 0,014273067 0,00703189

3,083868909 2,906635844 2,912943426 2,467469669 2,130308577 1,049535877

22,02763507 20,7616846 20,80673876 17,62478335 15,21648984 7,496684836

**min -1** 22,02763507 20,7616846 20,80673876 17,62478335 15,21648984 7,496684836

**30 min** 30

**PTPN22 reduced control** 22,02763507 76,05790021

**PTPN22 3.4uM H2O2** 20,7616846 4,469147798

**PTPN22 6.3uM H2O2** 20,80673876 97,26724967

**PTPN22 12.5uM H2O2** 17,62478335 31,22145889

**PTPN22 25uM H2O2** 15,21648984

**PTPN22 50uM H2O2** 7,496684836

Date: #########

Time: 13:54:27

System MTC-MU059-S

User MTC-MU059-S\fretho

Plate Greiner 96 Flat Bottom Transparent Polystyrene Cat. No.: 655101/655161/655192 [GRE96ft.pdfx] Plate-ID (Stacker)

List of actions in this measurement script: Kinetic

Absorbance

Label: Label1

Kinetic Measurement

Kinetic duration 00:06:00

Interval Time 00:00:20

Measurement Wavelength 405 nm

Bandwidth 10 nm

Number of Flashes 5

Settle Time 0 ms

Part of Plate E1-E12; F1-F2

Start Time: 2020 05-18 13:54:29

| Cycle Nr. | 1 | 2 | 3 | 4 | 5 | 6 | 7 | 8 | 9 | 10 | 11 | 12 | 13 | 14 | 15 | 16 | 17 | 18 | 19 |
| --- | --- | --- | --- | --- | --- | --- | --- | --- | --- | --- | --- | --- | --- | --- | --- | --- | --- | --- | --- |
| Time [s] | 0 | 20 | 40 | 60 | 80 | 100 | 120 | 140 | 160 | 180 | 200 | 220 | 240 | 260 | 280 | 300 | 320 | 340 | 360 |
| Temp. [°C] | 25,2 | 25,3 | 25,3 | 25,3 | 25,2 | 25,3 | 25,3 | 25,1 | 25,3 | 25,2 | 25,2 | 25,3 | 25,3 | 25,3 | 25,2 | 25,5 | 25,4 | 25,4 | 25,5 |
| E1 | 0,2511 | 0,2556 | 0,2619 | 0,269 | 0,2748 | 0,2838 | 0,2911 | 0,2964 | 0,3044 | 0,3126 | 0,3184 | 0,3261 | 0,3334 | 0,3405 | 0,3473 | 0,3543 | 0,3605 | 0,3684 | 0,3774 |
| E2 | 0,2554 | 0,2621 | 0,2651 | 0,2706 | 0,2779 | 0,2838 | 0,2918 | 0,2974 | 0,3047 | 0,3114 | 0,3189 | 0,3255 | 0,3318 | 0,3391 | 0,3452 | 0,3515 | 0,3581 | 0,3647 | 0,3722 |
| E3 | 0,251 | 0,2566 | 0,2793 | 0,2825 | 0,2761 | 0,2811 | 0,2893 | 0,2935 | 0,3014 | 0,3073 | 0,315 | 0,3216 | 0,3295 | 0,3349 | 0,3415 | 0,3488 | 0,3553 | 0,3615 | 0,3688 |
| E4 | 0,2509 | 0,2551 | 0,2613 | 0,2664 | 0,2737 | 0,2811 | 0,2882 | 0,2936 | 0,3017 | 0,3077 | 0,3146 | 0,3225 | 0,3281 | 0,3363 | 0,3425 | 0,3502 | 0,3548 | 0,3619 | 0,3697 |
| E5 | 0,2506 | 0,2535 | 0,26 | 0,266 | 0,2722 | 0,2797 | 0,2871 | 0,2909 | 0,2986 | 0,3054 | 0,3121 | 0,3195 | 0,3252 | 0,3321 | 0,338 | 0,3455 | 0,3516 | 0,3569 | 0,366 |
| E6 | 0,2489 | 0,2541 | 0,2591 | 0,2661 | 0,2718 | 0,2779 | 0,2852 | 0,289 | 0,2976 | 0,3044 | 0,3105 | 0,3177 | 0,3238 | 0,331 | 0,3375 | 0,3431 | 0,3485 | 0,3547 | 0,363 |
| E7 | 0,25 | 0,2535 | 0,2588 | 0,2629 | 0,2686 | 0,2751 | 0,2804 | 0,2838 | 0,2909 | 0,2967 | 0,3027 | 0,3081 | 0,3137 | 0,3204 | 0,3253 | 0,3309 | 0,3351 | 0,34 | 0,3474 |
| E8 | 0,2474 | 0,251 | 0,2561 | 0,2607 | 0,2667 | 0,2728 | 0,279 | 0,283 | 0,29 | 0,2942 | 0,3007 | 0,307 | 0,3113 | 0,3176 | 0,3211 | 0,3275 | 0,3327 | 0,3367 | 0,344 |
| E9 | 0,2463 | 0,2516 | 0,2556 | 0,2588 | 0,2633 | 0,2697 | 0,2752 | 0,2779 | 0,2838 | 0,2894 | 0,2951 | 0,2993 | 0,3038 | 0,309 | 0,3128 | 0,3174 | 0,322 | 0,3261 | 0,3321 |
| E10 | 0,2491 | 0,2538 | 0,2574 | 0,2609 | 0,265 | 0,271 | 0,2757 | 0,2807 | 0,2858 | 0,2898 | 0,2959 | 0,3012 | 0,305 | 0,3114 | 0,3138 | 0,3181 | 0,323 | 0,3258 | 0,3327 |
| E11 | 0,2472 | 0,2485 | 0,2501 | 0,25 | 0,2536 | 0,2555 | 0,2594 | 0,26 | 0,2629 | 0,265 | 0,2679 | 0,271 | 0,2727 | 0,2762 | 0,2768 | 0,2795 | 0,2801 | 0,2826 | 0,287 |
| E12 | 0,245 | 0,2471 | 0,2486 | 0,248 | 0,2519 | 0,2555 | 0,2594 | 0,2601 | 0,2625 | 0,266 | 0,2684 | 0,2705 | 0,2721 | 0,2751 | 0,2762 | 0,2803 | 0,281 | 0,2836 | 0,2866 |
| F1 | 0,2544 | 0,2537 | 0,2535 | 0,2541 | 0,2539 | 0,2551 | 0,2549 | 0,2532 | 0,2544 | 0,2547 | 0,2556 | 0,2559 | 0,2551 | 0,2565 | 0,255 | 0,2559 | 0,2545 | 0,2528 | 0,2546 |
| F2 | 0,2555 | 0,2552 | 0,256 | 0,2561 | 0,2549 | 0,256 | 0,2559 | 0,255 | 0,2554 | 0,2556 | 0,2561 | 0,2566 | 0,2555 | 0,2555 | 0,2554 | 0,2553 | 0,2541 | 0,2537 | 0,255 |

End Time: 2020 05-18 14:00:38

| 20 | 0,257600009 | 0,256500006 | 0,251599997 | | 0,259200007 | 0,261500001 | 0,270200014 | 0,263599992 | 0,264699996 | 0,262300014 | 0,259799987 | 0,263700008 | 0,257600009 | 0,257999986 | 0,258599997 |
| --- | --- | --- | --- | --- | --- | --- | --- | --- | --- | --- | --- | --- | --- | --- | --- |
| 40 | 0,2579 | 0,257699996 | 0,25850001 | | 0,2588 | 0,268099993 | 0,270200014 | 0,274699986 | 0,272399992 | 0,270099998 | 0,26910001 | 0,271499991 | 0,264899999 | 0,265599996 | 0,263999999 |
| 60 | 0,257699996 | 0,257200003 | 0,264400005 | | 0,263300002 | 0,274100006 | 0,276899993 | 0,282999992 | 0,279599994 | 0,278299987 | 0,275700003 | 0,278600007 | 0,271400005 | 0,271400005 | 0,270700008 |
| 80 | 0,258399993 | 0,256399989 | 0,273099989 | | 0,269499987 | 0,284799993 | 0,283600003 | 0,289999992 | 0,287099987 | 0,284500003 | 0,282200009 | 0,286199987 | 0,278200001 | 0,276300013 | 0,275400013 |
| 100 | 0,256599993 | 0,254299998 | 0,280200005 | | 0,27700001 | 0,291299999 | 0,289200008 | 0,297500014 | 0,293000013 | 0,290300012 | 0,287499994 | 0,292899996 | 0,282799989 | 0,280900002 | 0,280999988 |
| 120 | 0,256099999 | 0,257200003 | 0,287999988 | | 0,284299999 | 0,300300002 | 0,297899991 | 0,308099985 | 0,30219999 | 0,300300002 | 0,295599997 | 0,301999986 | 0,290399998 | 0,288100004 | 0,289200008 |
| 140 | 0,255299985 | 0,255600005 | 0,293500006 | | 0,288700014 | 0,307399988 | 0,305000007 | 0,314900011 | 0,309700012 | 0,305900007 | 0,300799996 | 0,309100002 | 0,297100008 | 0,293000013 | 0,293599993 |
| 160 | 0,256199986 | 0,256399989 | 0,301499993 | | 0,2958 | 0,315899998 | 0,313199997 | 0,323599994 | 0,318399996 | 0,315499991 | 0,309399992 | 0,317999989 | 0,30309999 | 0,301299989 | 0,300999999 |
| 180 | 0,254500002 | 0,255299985 | 0,308699995 | | 0,303200006 | 0,323700011 | 0,320899993 | 0,333200008 | 0,324499995 | 0,321500003 | 0,315600008 | 0,324400008 | 0,308600008 | 0,305599988 | 0,305400014 |
| 200 | 0,255299985 | 0,255199999 | 0,316399992 | | 0,308499992 | 0,332800001 | 0,328200012 | 0,341800004 | 0,333900005 | 0,330900013 | 0,323399991 | 0,333099991 | 0,316500008 | 0,311399996 | 0,312299997 |
| 220 | 0,254700005 | 0,256399989 | 0,323399991 | | 0,315200001 | 0,341899991 | 0,335700005 | 0,350400001 | 0,342700005 | 0,338699996 | 0,33039999 | 0,341500014 | 0,323599994 | 0,318699986 | 0,319000006 |
| 240 | 0,253300011 | 0,253600001 | 0,330799997 | | 0,320300013 | 0,3486 | 0,342099994 | 0,3583 | 0,349299997 | 0,344799995 | 0,336600006 | 0,34799999 | 0,328200012 | 0,322400004 | 0,323799998 |
| 260 | 0,254900008 | 0,255899996 | 0,337700009 | | 0,325399995 | 0,355399996 | 0,351000011 | 0,367700011 | 0,357899994 | 0,352800012 | 0,342400014 | 0,355599999 | 0,333900005 | 0,328999996 | 0,330900013 |
| 280 | 0,254299998 | 0,255400002 | 0,345899999 | | 0,332100004 | 0,363499999 | 0,357300013 | 0,375699997 | 0,365399987 | 0,359100014 | 0,349900007 | 0,363799989 | 0,340700001 | 0,334899992 | 0,335000008 |
| 300 | 0,254000008 | 0,254999995 | 0,354400009 | | 0,336699992 | 0,370200008 | 0,364100009 | 0,383100003 | 0,371499985 | 0,367599994 | 0,355699986 | 0,369800001 | 0,345400006 | 0,339700013 | 0,341699988 |
| 320 | 0,254200011 | 0,254999995 | 0,361900002 | | 0,341800004 | 0,377900004 | 0,371100008 | 0,391499996 | 0,379000008 | 0,373100013 | 0,361600012 | 0,377600014 | 0,352400005 | 0,345800012 | 0,346500009 |
| 340 | 0,252999991 | 0,254099995 | 0,368999988 | | 0,34889999 | 0,385600001 | 0,378399998 | 0,398799986 | 0,386799991 | 0,380899996 | 0,368600011 | 0,384900004 | 0,358599991 | 0,350499988 | 0,351300001 |
| 360 | 0,253199995 | 0,253399998 | 0,378800005 | | 0,354499996 | 0,393599987 | 0,385100007 | 0,407200009 | 0,3935 | 0,387499988 | 0,375099987 | 0,391499996 | 0,364300013 | 0,356900007 | 0,358099997 |
| slope | -1,47059E-05 | -8,25594E-06 | 0,000370526 | | 0,000295996 | 0,000393478 | 0,000357652 | 0,000421275 | 0,000384314 | 0,000371367 | 0,000336264 | 0,000380144 | 0,000312802 | 0,000288787 | 0,000294401 |
| slope w/o |  |  | 0,000382007 | | 0,000307477 | 0,000404959 | 0,000369133 | 0,000432755 | 0,000395795 | 0,000382848 | 0,000347745 | 0,000391625 | 0,000324283 | 0,000300268 | 0,000305882 |
| blank | |  |  |  | 0,000344742 |  | 0,000387046 |  | 0,000414275 |  | 0,000365297 |  | 0,000357954 |  | 0,000303075 |
|  | |  |  |  | 0,02068452 |  | 0,023222755 |  | 0,024856502 |  | 0,021917802 |  | 0,021477245 |  | 0,01818452 |
|  | |  |  |  | 3,087241718 |  | 3,466082902 |  | 3,709925687 |  | 3,271313681 |  | 3,205558976 |  | 2,714107505 |
|  | |  |  |  | 22,05172656 |  | 24,75773502 |  | 26,49946919 |  | 23,36652629 |  | 22,89684983 |  | 19,38648218 |
|  | |  | **min -1** |  | 22,05172656 |  | 24,75773502 |  | 26,49946919 |  | 23,36652629 |  | 22,89684983 |  | 19,38648218 |

**5 min**

**PTPN22 reduced control** 22,05172656 **PTPN22 3.4uM H2O2** 24,75773502 **PTPN22 6.3uM H2O2** 26,49946919 **PTPN22 12.5uM H2O2** 23,36652629 **PTPN22 25uM H2O2** 22,89684983 **PTPN22 50uM H2O2** 19,38648218

Date: #########

Time: 14:08:11

System MTC-MU059-S

User MTC-MU059-S\fretho

Plate Greiner 96 Flat Bottom Transparent Polystyrene Cat. No.: 655101/655161/655192 [GRE96ft.pdfx] Plate-ID (Stacker)

List of actions in this measurement script: Kinetic

Absorbance

Label: Label1

Kinetic Measurement

Kinetic duration 00:06:00

Interval Time 00:00:20

Measurement Wavelength 405 nm

Bandwidth 10 nm

Number of Flashes 5

Settle Time 0 ms

Part of Plate G1-G12; H1-H2

Start Time: 2020 05-18 14:08:13

| Cycle Nr. | 1 | 2 | 3 | 4 | 5 | 6 | 7 | 8 | 9 | 10 | 11 | 12 | 13 | 14 | 15 | 16 | 17 | 18 | 19 |
| --- | --- | --- | --- | --- | --- | --- | --- | --- | --- | --- | --- | --- | --- | --- | --- | --- | --- | --- | --- |
| Time [s] | 0 | 20 | 40 | 60 | 80 | 100 | 120 | 140 | 160 | 180 | 200 | 220,1 | 240 | 260 | 280 | 300 | 320 | 340 | 360 |
| Temp. [°C] | 25 | 25,1 | 25,1 | 25,1 | 25,1 | 25,4 | 25,2 | 25,1 | 25,3 | 25,4 | 25,4 | 25,3 | 25,4 | 25,5 | 25,4 | 25,4 | 25,4 | 25,4 | 25,5 |
| G1 | 0,2599 | 0,2615 | 0,2681 | 0,2741 | 0,2848 | 0,2913 | 0,3003 | 0,3074 | 0,3159 | 0,3237 | 0,3328 | 0,3419 | 0,3486 | 0,3554 | 0,3635 | 0,3702 | 0,3779 | 0,3856 | 0,3936 |
| G2 | 0,2625 | 0,2702 | 0,2702 | 0,2769 | 0,2836 | 0,2892 | 0,2979 | 0,305 | 0,3132 | 0,3209 | 0,3282 | 0,3357 | 0,3421 | 0,351 | 0,3573 | 0,3641 | 0,3711 | 0,3784 | 0,3851 |
| G3 | 0,2604 | 0,2636 | 0,2747 | 0,283 | 0,29 | 0,2975 | 0,3081 | 0,3149 | 0,3236 | 0,3332 | 0,3418 | 0,3504 | 0,3583 | 0,3677 | 0,3757 | 0,3831 | 0,3915 | 0,3988 | 0,4072 |
| G4 | 0,2658 | 0,2647 | 0,2724 | 0,2796 | 0,2871 | 0,293 | 0,3022 | 0,3097 | 0,3184 | 0,3245 | 0,3339 | 0,3427 | 0,3493 | 0,3579 | 0,3654 | 0,3715 | 0,379 | 0,3868 | 0,3935 |
| G5 | 0,2564 | 0,2623 | 0,2701 | 0,2783 | 0,2845 | 0,2903 | 0,3003 | 0,3059 | 0,3155 | 0,3215 | 0,3309 | 0,3387 | 0,3448 | 0,3528 | 0,3591 | 0,3676 | 0,3731 | 0,3809 | 0,3875 |
| G6 | 0,2602 | 0,2598 | 0,2691 | 0,2757 | 0,2822 | 0,2875 | 0,2956 | 0,3008 | 0,3094 | 0,3156 | 0,3234 | 0,3304 | 0,3366 | 0,3424 | 0,3499 | 0,3557 | 0,3616 | 0,3686 | 0,3751 |
| G7 | 0,2614 | 0,2637 | 0,2715 | 0,2786 | 0,2862 | 0,2929 | 0,302 | 0,3091 | 0,318 | 0,3244 | 0,3331 | 0,3415 | 0,348 | 0,3556 | 0,3638 | 0,3698 | 0,3776 | 0,3849 | 0,3915 |
| G8 | 0,2529 | 0,2576 | 0,2649 | 0,2714 | 0,2782 | 0,2828 | 0,2904 | 0,2971 | 0,3031 | 0,3086 | 0,3165 | 0,3236 | 0,3282 | 0,3339 | 0,3407 | 0,3454 | 0,3524 | 0,3586 | 0,3643 |
| G9 | 0,2563 | 0,258 | 0,2656 | 0,2714 | 0,2763 | 0,2809 | 0,2881 | 0,293 | 0,3013 | 0,3056 | 0,3114 | 0,3187 | 0,3224 | 0,329 | 0,3349 | 0,3397 | 0,3458 | 0,3505 | 0,3569 |
| G10 | 0,2574 | 0,2586 | 0,264 | 0,2707 | 0,2754 | 0,281 | 0,2892 | 0,2936 | 0,301 | 0,3054 | 0,3123 | 0,319 | 0,3238 | 0,3309 | 0,335 | 0,3417 | 0,3465 | 0,3513 | 0,3581 |
| G11 | 0,2591 | 0,2572 | 0,2614 | 0,2653 | 0,2712 | 0,2747 | 0,2814 | 0,2854 | 0,2908 | 0,2954 | 0,3003 | 0,3072 | 0,3098 | 0,3134 | 0,3199 | 0,3251 | 0,3292 | 0,334 | 0,339 |
| G12 | 0,2574 | 0,2551 | 0,2609 | 0,2661 | 0,2714 | 0,2759 | 0,2808 | 0,2848 | 0,2911 | 0,2937 | 0,3012 | 0,3075 | 0,3101 | 0,3159 | 0,3214 | 0,3253 | 0,3298 | 0,3357 | 0,3379 |
| H1 | 0,2569 | 0,2576 | 0,2579 | 0,2577 | 0,2584 | 0,2566 | 0,2561 | 0,2553 | 0,2562 | 0,2545 | 0,2553 | 0,2547 | 0,2533 | 0,2549 | 0,2543 | 0,254 | 0,2542 | 0,253 | 0,2532 |
| H2 | 0,2581 | 0,2565 | 0,2577 | 0,2572 | 0,2564 | 0,2543 | 0,2572 | 0,2556 | 0,2564 | 0,2553 | 0,2552 | 0,2564 | 0,2536 | 0,2559 | 0,2554 | 0,255 | 0,255 | 0,2541 | 0,2534 |

End Time: 2020 05-18 14:14:22

blank

| 20 | 0,257800013 | 0,255600005 | 0,2509 | 0,252700001 | 0,258899987 | 0,259299994 | 0,280099988 | 0,255100012 | 0,255299985 | 0,254500002 | 0,252900004 | 0,251599997 | 0,248899996 | 0,248400003 |
| --- | --- | --- | --- | --- | --- | --- | --- | --- | --- | --- | --- | --- | --- | --- |
| 40 | 0,257400006 | 0,256900012 | 0,255299985 | 0,258599997 | 0,263500005 | 0,260800004 | 0,261999995 | 0,261999995 | 0,260500014 | 0,25999999 | 0,257200003 | 0,256799996 | 0,25150001 | 0,253500015 |
| 60 | 0,257299989 | 0,256300002 | 0,260600001 | 0,261900008 | 0,269899994 | 0,26789999 | 0,268299997 | 0,26910001 | 0,267399997 | 0,265399992 | 0,263399988 | 0,261200011 | 0,254099995 | 0,254700005 |
| 80 | 0,256000012 | 0,255199999 | 0,268599987 | 0,267500013 | 0,275200009 | 0,270599991 | 0,271499991 | 0,273299992 | 0,271299988 | 0,270799994 | 0,265199989 | 0,264999986 | 0,257099986 | 0,2588 |
| 100 | 0,256500006 | 0,255100012 | 0,278299987 | 0,275099993 | 0,282599986 | 0,277700007 | 0,279300004 | 0,279500008 | 0,277099997 | 0,2764 | 0,270799994 | 0,269800007 | 0,260800004 | 0,261700004 |
| 120 | 0,257400006 | 0,256399989 | 0,28459999 | 0,282900006 | 0,290800005 | 0,284700006 | 0,28459999 | 0,287600011 | 0,284500003 | 0,282700002 | 0,27669999 | 0,275400013 | 0,265899986 | 0,2667 |
| 140 | 0,257800013 | 0,256199986 | 0,292299986 | 0,288300008 | 0,300099999 | 0,291099995 | 0,294699997 | 0,294400007 | 0,291799992 | 0,289600015 | 0,282799989 | 0,281500012 | 0,270099998 | 0,271400005 |
| 160 | 0,256799996 | 0,256199986 | 0,299800009 | 0,294499993 | 0,307099998 | 0,298299998 | 0,300300002 | 0,301099986 | 0,297300011 | 0,296000004 | 0,288199991 | 0,286599994 | 0,273699999 | 0,274699986 |
| 180 | 0,255299985 | 0,254400015 | 0,307099998 | 0,300500005 | 0,314200014 | 0,302700013 | 0,305900007 | 0,305700004 | 0,3028 | 0,300599992 | 0,292400002 | 0,291000009 | 0,276499987 | 0,278299987 |
| 200 | 0,256799996 | 0,255499989 | 0,3125 | 0,307200015 | 0,320800006 | 0,309500009 | 0,311800003 | 0,313699991 | 0,310400009 | 0,307000011 | 0,298299998 | 0,296099991 | 0,280800015 | 0,281399995 |
| 220 | 0,256000012 | 0,254700005 | 0,321799994 | 0,313800007 | 0,327300012 | 0,317699999 | 0,320100009 | 0,321399987 | 0,316300005 | 0,314700007 | 0,304800004 | 0,301899999 | 0,28549999 | 0,2861 |
| 240 | 0,256000012 | 0,255199999 | 0,328000009 | 0,319900006 | 0,336299986 | 0,323300004 | 0,327899992 | 0,326700002 | 0,322899997 | 0,321399987 | 0,309599996 | 0,306300014 | 0,287999988 | 0,288399994 |
| 260 | 0,256099999 | 0,256300002 | 0,335099995 | 0,326999992 | 0,344500005 | 0,330199987 | 0,332899988 | 0,334399998 | 0,328599989 | 0,326400012 | 0,314599991 | 0,311399996 | 0,291999996 | 0,292400002 |
| 280 | 0,255699992 | 0,253600001 | 0,342599988 | 0,333499998 | 0,351999998 | 0,336800009 | 0,340000004 | 0,340000004 | 0,334699988 | 0,333900005 | 0,319000006 | 0,316199988 | 0,296200007 | 0,297100008 |
| 300 | 0,256700009 | 0,255100012 | 0,349799991 | 0,339700013 | 0,359600008 | 0,343199998 | 0,347900003 | 0,347600013 | 0,340499997 | 0,340799987 | 0,324900001 | 0,32159999 | 0,299699992 | 0,300799996 |
| 320 | 0,256399989 | 0,255899996 | 0,35679999 | 0,347000003 | 0,367300004 | 0,350199997 | 0,354499996 | 0,354499996 | 0,34799999 | 0,346199989 | 0,331099987 | 0,328000009 | 0,305000007 | 0,306199998 |
| 340 | 0,256399989 | 0,254000008 | 0,362599999 | 0,351799995 | 0,3741 | 0,355500013 | 0,363299996 | 0,360199988 | 0,354000002 | 0,351999998 | 0,334699988 | 0,33160001 | 0,307300001 | 0,306899995 |
| 360 | 0,256700009 | 0,255400002 | 0,370599985 | 0,359800011 | 0,380899996 | 0,362199992 | 0,371499985 | 0,366899997 | 0,359100014 | 0,357199997 | 0,33919999 | 0,337199986 | 0,311399996 | 0,311500013 |
| slope | -3,29724E-06 | -3,91125E-06 | 0,000359665 | 0,000318684 | 0,000370294 | 0,000315516 | 0,000317337 | 0,000330423 | 0,0003108 | 0,000309143 | 0,000260846 | 0,000253498 | 0,00018887 | 0,0001871 |
| slope w/o |  |  | 0,000363269 | 0,000322288 | 0,000373898 | 0,00031912 | 0,000320942 | 0,000334027 | 0,000314404 | 0,000312748 | 0,00026445 | 0,000257103 | 0,000192474 | 0,000190704 |

0,000342779 0,000346509 0,000327485 0,000313576 0,000260777 0,000191589

0,020566719 0,020790559 0,019649071 0,018814552 0,015646595 0,011495357

3,069659553 3,103068458 2,932697204 2,808142074 2,335312613 1,715724864

21,92613966 22,1647747 20,94783717 20,05815767 16,68080438 12,2551776

**min -1** 21,92613966 22,1647747 20,94783717 20,05815767 16,68080438 12,2551776

**15 min** 30

**PTPN22 reduced control** 21,92613966 76,05790021

**PTPN22 3.4uM H2O2** 22,1647747 4,469147798

**PTPN22 6.3uM H2O2** 20,94783717 97,26724967

**PTPN22 12.5uM H2O2** 20,05815767 31,22145889

**PTPN22 25uM H2O2** 16,68080438

**PTPN22 50uM H2O2** 12,2551776

Date: #########

Time: 14:18:07

System MTC-MU059-S

User MTC-MU059-S\fretho

Plate Greiner 96 Flat Bottom Transparent Polystyrene Cat. No.: 655101/655161/655192 [GRE96ft.pdfx] Plate-ID (Stacker)

List of actions in this measurement script: Kinetic

Absorbance

Label: Label1

Kinetic Measurement

Kinetic duration 00:06:00

Interval Time 00:00:20

Measurement Wavelength 405 nm

Bandwidth 10 nm

Number of Flashes 5

Settle Time 0 ms

Part of Plate G1-G12; H1-H2

Start Time: 2020 05-18 14:18:10

| Cycle Nr. | 1 | 2 | 3 | 4 | 5 | 6 | 7 | 8 | 9 | 10 | 11 | 12 | 13 | 14 | 15 | 16 | 17 | 18 | 19 |
| --- | --- | --- | --- | --- | --- | --- | --- | --- | --- | --- | --- | --- | --- | --- | --- | --- | --- | --- | --- |
| Time [s] | 0 | 20 | 40 | 60 | 80 | 100 | 120 | 140 | 160 | 180 | 200 | 220 | 240 | 260 | 280 | 300 | 320 | 340 | 360 |
| Temp. [°C] | 25,4 | 25,3 | 25,5 | 25,3 | 25,4 | 25,4 | 25,6 | 25,6 | 25,5 | 25,4 | 25,5 | 25,4 | 25,3 | 25,6 | 25,4 | 25,3 | 25,8 | 25,6 | 25,8 |
| G1 | 0,2574 | 0,2589 | 0,2635 | 0,2699 | 0,2752 | 0,2826 | 0,2908 | 0,3001 | 0,3071 | 0,3142 | 0,3208 | 0,3273 | 0,3363 | 0,3445 | 0,352 | 0,3596 | 0,3673 | 0,3741 | 0,3809 |
| G2 | 0,2556 | 0,2593 | 0,2608 | 0,2679 | 0,2706 | 0,2777 | 0,2847 | 0,2911 | 0,2983 | 0,3027 | 0,3095 | 0,3177 | 0,3233 | 0,3302 | 0,3368 | 0,3432 | 0,3502 | 0,3555 | 0,3622 |
| G3 | 0,252 | 0,2801 | 0,262 | 0,2683 | 0,2715 | 0,2793 | 0,2846 | 0,2947 | 0,3003 | 0,3059 | 0,3118 | 0,3201 | 0,3279 | 0,3329 | 0,34 | 0,3479 | 0,3545 | 0,3633 | 0,3715 |
| G4 | 0,253 | 0,2551 | 0,262 | 0,2691 | 0,2733 | 0,2795 | 0,2876 | 0,2944 | 0,3011 | 0,3057 | 0,3137 | 0,3214 | 0,3267 | 0,3344 | 0,34 | 0,3476 | 0,3545 | 0,3602 | 0,3669 |
| G5 | 0,2564 | 0,2553 | 0,2605 | 0,2674 | 0,2713 | 0,2771 | 0,2845 | 0,2918 | 0,2973 | 0,3028 | 0,3104 | 0,3163 | 0,3229 | 0,3286 | 0,3347 | 0,3405 | 0,348 | 0,354 | 0,3591 |
| G6 | 0,2522 | 0,2545 | 0,26 | 0,2654 | 0,2708 | 0,2764 | 0,2827 | 0,2896 | 0,296 | 0,3006 | 0,307 | 0,3147 | 0,3214 | 0,3264 | 0,3339 | 0,3408 | 0,3462 | 0,352 | 0,3572 |
| G7 | 0,2536 | 0,2529 | 0,2572 | 0,2634 | 0,2652 | 0,2708 | 0,2767 | 0,2828 | 0,2882 | 0,2924 | 0,2983 | 0,3048 | 0,3096 | 0,3146 | 0,319 | 0,3249 | 0,3311 | 0,3347 | 0,3392 |
| G8 | 0,251 | 0,2516 | 0,2568 | 0,2612 | 0,265 | 0,2698 | 0,2754 | 0,2815 | 0,2866 | 0,291 | 0,2961 | 0,3019 | 0,3063 | 0,3114 | 0,3162 | 0,3216 | 0,328 | 0,3316 | 0,3372 |
| G9 | 0,2485 | 0,2489 | 0,2515 | 0,2541 | 0,2571 | 0,2608 | 0,2659 | 0,2701 | 0,2737 | 0,2765 | 0,2808 | 0,2855 | 0,288 | 0,292 | 0,2962 | 0,2997 | 0,305 | 0,3073 | 0,3114 |
| G10 | 0,2487 | 0,2484 | 0,2535 | 0,2547 | 0,2588 | 0,2617 | 0,2667 | 0,2714 | 0,2747 | 0,2783 | 0,2814 | 0,2861 | 0,2884 | 0,2924 | 0,2971 | 0,3008 | 0,3062 | 0,3069 | 0,3115 |
| G11 | 0,2588 | 0,2595 | 0,2604 | 0,2646 | 0,2699 | 0,2758 | 0,2795 | 0,2861 | 0,2898 | 0,2931 | 0,3001 | 0,3036 | 0,3076 | 0,313 | 0,319 | 0,3227 | 0,3288 | 0,3324 | 0,3377 |
| G12 | 0,256 | 0,2582 | 0,2591 | 0,2637 | 0,2696 | 0,2737 | 0,2807 | 0,2856 | 0,2894 | 0,2935 | 0,2993 | 0,304 | 0,31 | 0,3149 | 0,3184 | 0,3248 | 0,3304 | 0,3337 | 0,3386 |
| H1 | 0,2559 | 0,2578 | 0,2574 | 0,2573 | 0,256 | 0,2565 | 0,2574 | 0,2578 | 0,2568 | 0,2553 | 0,2568 | 0,256 | 0,256 | 0,2561 | 0,2557 | 0,2567 | 0,2564 | 0,2564 | 0,2567 |
| H2 | 0,2544 | 0,2556 | 0,2569 | 0,2563 | 0,2552 | 0,2551 | 0,2564 | 0,2562 | 0,2562 | 0,2544 | 0,2555 | 0,2547 | 0,2552 | 0,2563 | 0,2536 | 0,2551 | 0,2559 | 0,254 | 0,2554 |

End Time: 2020 05-18 14:24:18

| 20 | 0,253699988 0,253699988 | 0,2465 | | 0,255400002 | 0,256000012 | 0,254000008 | 0,252400011 | 0,255100012 | 0,251300007 | 0,254700005 | 0,250999987 | 0,249799997 | 0,246299997 | 0,243100002 |
| --- | --- | --- | --- | --- | --- | --- | --- | --- | --- | --- | --- | --- | --- | --- |
| 40 | 0,255699992 0,256500006 | 0,253100008 | | 0,258700013 | 0,262199998 | 0,261799991 | 0,260100007 | 0,262600005 | 0,257499993 | 0,25999999 | 0,255699992 | 0,254400015 | 0,2491 | 0,246399999 |
| 60 | 0,256099999 0,255499989 | 0,2588 | | 0,265700012 | 0,267199993 | 0,267399997 | 0,266499996 | 0,267100006 | 0,263599992 | 0,265199989 | 0,259799987 | 0,257099986 | 0,252499998 | 0,249200001 |
| 80 | 0,256900012 0,255699992 | 0,265599996 | | 0,271200001 | 0,274500012 | 0,274199992 | 0,271800011 | 0,273000002 | 0,268099993 | 0,269899994 | 0,265100002 | 0,261700004 | 0,254000008 | 0,251800001 |
| 100 | 0,256000012 0,254700005 | 0,272000015 | | 0,277399987 | 0,280000001 | 0,278299987 | 0,274599999 | 0,278100014 | 0,272399992 | 0,272899985 | 0,2667 | 0,264800012 | 0,256500006 | 0,253699988 |
| 120 | 0,254999995 0,254500002 | 0,277700007 | | 0,284299999 | 0,2852 | 0,284000009 | 0,281699985 | 0,283800006 | 0,277200013 | 0,278299987 | 0,271200001 | 0,268900007 | 0,25819999 | 0,255400002 |
| 140 | 0,256700009 0,255100012 | 0,282799989 | | 0,289400011 | 0,291999996 | 0,290300012 | 0,286799997 | 0,290199995 | 0,283199996 | 0,282999992 | 0,274399996 | 0,272899985 | 0,261500001 | 0,259200007 |
| 160 | 0,256199986 0,256000012 | 0,291099995 | | 0,296900004 | 0,298799992 | 0,297399998 | 0,293199986 | 0,295700014 | 0,288599998 | 0,289400011 | 0,278299987 | 0,27700001 | 0,263799995 | 0,262100011 |
| 180 | 0,257099986 0,257200003 | 0,295899987 | | 0,303299993 | 0,306499988 | 0,3037 | 0,299199998 | 0,303299993 | 0,294200003 | 0,29519999 | 0,284500003 | 0,281500012 | 0,267199993 | 0,264200002 |
| 200 | 0,256799996 0,256099999 | 0,302599996 | | 0,307700008 | 0,3116 | 0,310099989 | 0,30430001 | 0,308699995 | 0,298400015 | 0,299100012 | 0,285600007 | 0,28490001 | 0,268599987 | 0,265300006 |
| 220 | 0,257499993 0,255899996 | 0,308999985 | | 0,314999998 | 0,318899989 | 0,315600008 | 0,310699999 | 0,315800011 | 0,3037 | 0,3046 | 0,290800005 | 0,289600015 | 0,270999998 | 0,26820001 |
| 240 | 0,256900012 0,255899996 | 0,314200014 | | 0,320899993 | 0,324900001 | 0,321099997 | 0,316500008 | 0,321799994 | 0,310099989 | 0,309500009 | 0,294699997 | 0,293500006 | 0,273799986 | 0,271499991 |
| 260 | 0,256999999 0,256500006 | 0,321200013 | | 0,324699998 | 0,330199987 | 0,326599985 | 0,321799994 | 0,327600002 | 0,31400001 | 0,314700007 | 0,297800004 | 0,296299994 | 0,275200009 | 0,272700012 |
| 280 | 0,255699992 0,255499989 | 0,328299999 | | 0,332899988 | 0,336400002 | 0,332700014 | 0,326299995 | 0,333000004 | 0,319599986 | 0,319400012 | 0,299699992 | 0,299400002 | 0,27700001 | 0,273699999 |
| 300 | 0,256500006 0,256599993 | 0,332599998 | | 0,337900013 | 0,341600001 | 0,337900013 | 0,33129999 | 0,337199986 | 0,324900001 | 0,324600011 | 0,30399999 | 0,3028 | 0,279599994 | 0,275700003 |
| 320 | 0,256399989 0,256000012 | 0,33950001 | | 0,344999999 | 0,347900003 | 0,343300015 | 0,337599993 | 0,343499988 | 0,328700006 | 0,327899992 | 0,305999994 | 0,305099994 | 0,280400008 | 0,277200013 |
| 340 | 0,257800013 0,257200003 | 0,346700013 | | 0,352499992 | 0,354699999 | 0,350400001 | 0,343300015 | 0,350100011 | 0,336800009 | 0,334500015 | 0,311399996 | 0,311399996 | 0,28490001 | 0,280699998 |
| 360 | 0,256399989 0,256399989 | 0,351799995 | | 0,357499987 | 0,360100001 | 0,354900002 | 0,347200006 | 0,354900002 | 0,339100003 | 0,338099986 | 0,314300001 | 0,31220001 | 0,285299987 | 0,280800015 |
| slope | 4,78844E-06 4,98452E-06 | 0,000310052 | | 0,000304737 | 0,000309438 | 0,000295898 | 0,000278148 | 0,000295815 | 0,000259009 | 0,00024806 | 0,00018387 | 0,000187786 | 0,000115186 | 0,000111677 |
| slope w/o |  | 0,000305165 | | 0,00029985 | 0,000304551 | 0,000291011 | 0,000273261 | 0,000290929 | 0,000254123 | 0,000243173 | 0,000178984 | 0,0001829 | 0,000110299 | 0,000106791 |
| blank | | |  | 0,000302508 |  | 0,000297781 |  | 0,000282095 |  | 0,000248648 |  | 0,000180942 |  | 0,000108545 |
|  | | |  | 0,018150463 |  | 0,017866873 |  | 0,016925695 |  | 0,014918887 |  | 0,010856502 |  | 0,006512694 |
|  | | |  | 2,709024388 |  | 2,666697533 |  | 2,526223197 |  | 2,226699563 |  | 1,6203734 |  | 0,972043917 |
|  | | |  | 19,3501742 |  | 19,04783952 |  | 18,04445141 |  | 15,90499688 |  | 11,57409572 |  | 6,943170837 |
| **min -1** | | |  | 19,3501742 |  | 19,04783952 |  | 18,04445141 |  | 15,90499688 |  | 11,57409572 |  | 6,943170837 |

|  |  | **30 min** |  | | | | | | | | | | | | |
| --- | --- | --- | --- | --- | --- | --- | --- | --- | --- | --- | --- | --- | --- | --- | --- |
|  | **PTPN22 reduced control** | 19,3501742 |  |  |  |  |  |  |  |  |  |  |  |  |  |
|  | **PTPN22 3.4uM H2O2** | 19,04783952 |  |  |  |  |  |  |  |  |  |  |  |  |  |
|  | **PTPN22 6.3uM H2O2** | 18,04445141 |  |  |  |  |  |  |  |  |  |  |  |  |  |
|  | **PTPN22 12.5uM H2O2** | 15,90499688 |  |  |  |  |  |  |  |  |  |  |  |  |  |
|  | **PTPN22 25uM H2O2** | 11,57409572 |  |  |  |  |  |  |  |  |  |  |  |  |  |
|  | **PTPN22 50uM H2O2** | 6,943170837 |  |  |  |  |  |  |  |  |  |  |  |  |  |

Date: #########

Time: 14:33:21

System MTC-MU059-S

User MTC-MU059-S\fretho

Plate Greiner 96 Flat Bottom Transparent Polystyrene Cat. No.: 655101/655161/655192 [GRE96ft.pdfx] Plate-ID (Stacker)

List of actions in this measurement script: Kinetic

Absorbance

Label: Label1

Kinetic Measurement

Kinetic duration 00:06:00

Interval Time 00:00:20

Measurement Wavelength 405 nm

Bandwidth 10 nm

Number of Flashes 5

Settle Time 0 ms

Part of Plate G1-G12; H1-H2

Start Time: 2020 05-18 14:33:23

| Cycle Nr. | 1 | 2 | 3 | 4 | 5 | 6 | 7 | 8 | 9 | 10 | 11 | 12 | 13 | 14 | 15 | 16 | 17 | 18 | 19 |
| --- | --- | --- | --- | --- | --- | --- | --- | --- | --- | --- | --- | --- | --- | --- | --- | --- | --- | --- | --- |
| Time [s] | 0 | 20 | 40 | 60 | 80 | 100 | 120 | 140 | 160 | 180 | 200 | 220 | 240 | 260 | 280 | 300 | 320 | 340 | 360 |
| Temp. [°C] | 25,3 | 25,7 | 25,5 | 25,5 | 25,3 | 25,6 | 25,5 | 25,5 | 25,7 | 25,5 | 25,7 | 25,6 | 25,5 | 25,5 | 25,4 | 25,5 | 25,6 | 25,4 | 25,7 |
| G1 | 0,2561 | 0,256 | 0,2622 | 0,2672 | 0,2745 | 0,28 | 0,2852 | 0,292 | 0,2988 | 0,3065 | 0,3116 | 0,3189 | 0,3249 | 0,3302 | 0,3364 | 0,3416 | 0,3479 | 0,3547 | 0,3601 |
| G2 | 0,2542 | 0,254 | 0,2618 | 0,2674 | 0,2742 | 0,2783 | 0,284 | 0,2903 | 0,2974 | 0,3037 | 0,3101 | 0,3156 | 0,3211 | 0,3266 | 0,3327 | 0,3379 | 0,3433 | 0,3504 | 0,3549 |
| G3 | 0,2531 | 0,2524 | 0,2601 | 0,2665 | 0,2718 | 0,2746 | 0,2817 | 0,2868 | 0,2932 | 0,2992 | 0,3043 | 0,3107 | 0,3165 | 0,3218 | 0,3263 | 0,3313 | 0,3376 | 0,3433 | 0,3472 |
| G4 | 0,2792 | 0,2551 | 0,2626 | 0,2671 | 0,273 | 0,2781 | 0,2838 | 0,2902 | 0,2957 | 0,3033 | 0,3087 | 0,3158 | 0,3218 | 0,3276 | 0,333 | 0,3372 | 0,3435 | 0,3501 | 0,3549 |
| G5 | 0,2635 | 0,2513 | 0,2575 | 0,2636 | 0,2681 | 0,2724 | 0,2772 | 0,2832 | 0,2886 | 0,2942 | 0,2984 | 0,3037 | 0,3101 | 0,314 | 0,3196 | 0,3249 | 0,3287 | 0,3368 | 0,3391 |
| G6 | 0,2507 | 0,2547 | 0,26 | 0,2652 | 0,2699 | 0,2729 | 0,2783 | 0,283 | 0,2894 | 0,2952 | 0,2991 | 0,3046 | 0,3095 | 0,3147 | 0,3194 | 0,3246 | 0,3279 | 0,3345 | 0,3381 |
| G7 | 0,2491 | 0,251 | 0,2557 | 0,2598 | 0,2651 | 0,2667 | 0,2712 | 0,2744 | 0,2783 | 0,2845 | 0,2856 | 0,2908 | 0,2947 | 0,2978 | 0,2997 | 0,304 | 0,306 | 0,3114 | 0,3143 |
| G8 | 0,2472 | 0,2498 | 0,2544 | 0,2571 | 0,2617 | 0,2648 | 0,2689 | 0,2729 | 0,277 | 0,2815 | 0,2849 | 0,2896 | 0,2935 | 0,2963 | 0,2994 | 0,3028 | 0,3051 | 0,3114 | 0,3122 |
| G9 | 0,2454 | 0,2463 | 0,2491 | 0,2525 | 0,254 | 0,2565 | 0,2582 | 0,2615 | 0,2638 | 0,2672 | 0,2686 | 0,271 | 0,2738 | 0,2752 | 0,277 | 0,2796 | 0,2804 | 0,2849 | 0,2853 |
| G10 | 0,2441 | 0,2431 | 0,2464 | 0,2492 | 0,2518 | 0,2537 | 0,2554 | 0,2592 | 0,2621 | 0,2642 | 0,2653 | 0,2682 | 0,2715 | 0,2727 | 0,2737 | 0,2757 | 0,2772 | 0,2807 | 0,2808 |
| G11 | 0,2578 | 0,256 | 0,2622 | 0,2669 | 0,2713 | 0,2759 | 0,2798 | 0,2852 | 0,2896 | 0,2949 | 0,2996 | 0,3056 | 0,3087 | 0,3126 | 0,3174 | 0,3215 | 0,3259 | 0,3319 | 0,3349 |
| G12 | 0,257 | 0,2534 | 0,2539 | 0,2578 | 0,2605 | 0,2626 | 0,266 | 0,2706 | 0,2725 | 0,2766 | 0,2778 | 0,2819 | 0,2849 | 0,2885 | 0,292 | 0,2933 | 0,2954 | 0,3004 | 0,3011 |
| H1 | 0,2536 | 0,2537 | 0,2557 | 0,2561 | 0,2569 | 0,256 | 0,255 | 0,2567 | 0,2562 | 0,2571 | 0,2568 | 0,2575 | 0,2569 | 0,257 | 0,2557 | 0,2565 | 0,2564 | 0,2578 | 0,2564 |
| H2 | 0,2547 | 0,2537 | 0,2565 | 0,2555 | 0,2557 | 0,2547 | 0,2545 | 0,2551 | 0,256 | 0,2572 | 0,2561 | 0,2559 | 0,2559 | 0,2565 | 0,2555 | 0,2566 | 0,256 | 0,2572 | 0,2564 |

End Time: 2020 05-18 14:39:32
