# Supplementary material for: Redox regulation of PTPN22 affects the severity of T-cell-dependent autoimmune inflammation
Source: eLife. 2022 May 19;11:e74549. doi: 10.7554/eLife.74549 (PMC9119677; doi:10.7554/eLife.74549)
Supplement: Figure 1—source data 3. [file elife-74549-fig1-data3.docx]

**PTPN22 WT control PTPN22 WT control PTPN22 WT control+bicobnaPTPN22 WT control+bicobna PTPN22 WT 30uM H2O2 PTPN22 WT 30uM H2O2 PTPN22 WT 30uM H2O2+bicobnate PTPN22 WT 30uM H2O2+bicobnate**

| blank |  | 0,000439814 | 0,000367758 | 0,00031832 | 5,15274E-05 |
| --- | --- | --- | --- | --- | --- |
|  |  | 0,026388856 | 0,022065481 | 0,019099226 | 0,003091641 |
|  |  | 3,938635238 | 3,293355363 | 2,850630792 | 0,461439007 |
|  |  | 28,13310884 | 23,52396688 | 20,36164852 | 3,295992907 |
|  | **min -1** | 28,13310884 | 23,52396688 | 20,36164852 | 3,295992907 |

**Time (sec) Blank Blank**

|  | 20 | 0,258100003 | 0,26409999 | 0,257299989 | 0,273499995 | 0,282799989 | 0,256999999 | 0,253500015 | 0,253800005 | 0,241899997 | 0,240400001 |
| --- | --- | --- | --- | --- | --- | --- | --- | --- | --- | --- | --- |
|  | 40 | 0,254799992 | 0,25600001 | 0,263300002 | 0,266799986 | 0,262699991 | 0,261500001 | 0,259200007 | 0,25819999 | 0,238800004 | 0,237299994 |
|  | 60 | 0,258599997 | 0,25870001 | 0,272399992 | 0,276899993 | 0,272899985 | 0,272399992 | 0,2676 | 0,265500009 | 0,243200004 | 0,241699994 |
|  | 80 | 0,25819999 | 0,2588 | 0,283399999 | 0,283600003 | 0,279700011 | 0,279599994 | 0,275400013 | 0,270999998 | 0,243699998 | 0,2421 |
|  | 100 | 0,256500006 | 0,26010001 | 0,293799996 | 0,293199986 | 0,287699997 | 0,287999988 | 0,282099992 | 0,275599986 | 0,243699998 | 0,242699996 |
|  | 120 | 0,256099999 | 0,2579 | 0,300700009 | 0,302399993 | 0,294400007 | 0,29339999 | 0,288700014 | 0,280999988 | 0,246399999 | 0,244299993 |
|  | 140 | 0,256599993 | 0,25889999 | 0,31099999 | 0,3116 | 0,30399999 | 0,301699996 | 0,295100003 | 0,290499985 | 0,249400005 | 0,246199995 |
|  | 160 | 0,254999995 | 0,2581 | 0,319700003 | 0,322299987 | 0,309399992 | 0,306600004 | 0,30250001 | 0,295700014 | 0,251599997 | 0,246099994 |
|  | 180 | 0,255400002 | 0,25709999 | 0,328599989 | 0,330000013 | 0,316399992 | 0,315699995 | 0,310000002 | 0,301499993 | 0,253100008 | 0,246900007 |
|  | 200 | 0,255400002 | 0,25549999 | 0,336400002 | 0,338999987 | 0,326299995 | 0,325300008 | 0,316199988 | 0,307900012 | 0,252600014 | 0,249500006 |
|  | 220 | 0,256799996 | 0,25889999 | 0,345800012 | 0,348500013 | 0,332300007 | 0,331499994 | 0,324099988 | 0,3134 | 0,254700005 | 0,248099998 |
|  | 240 | 0,255299985 | 0,25569999 | 0,354499996 | 0,3565 | 0,339399993 | 0,33829999 | 0,3292 | 0,319400012 | 0,254799992 | 0,247899994 |
|  | 260 | 0,255299985 | 0,25839999 | 0,364399999 | 0,363900006 | 0,345699996 | 0,34709999 | 0,336400002 | 0,325100005 | 0,253100008 | 0,249799997 |
|  | 280 | 0,256300002 | 0,25619999 | 0,372700006 | 0,373699993 | 0,354999989 | 0,35589999 | 0,342299998 | 0,330199987 | 0,256000012 | 0,250400007 |
|  | 300 | 0,257200003 | 0,25659999 | 0,381300002 | 0,381399989 | 0,361600012 | 0,362399995 | 0,3486 | 0,334500015 | 0,256300002 | 0,251800001 |
|  | 320 | 0,256900012 | 0,257 | 0,390500009 | 0,390399992 | 0,367799997 | 0,369100004 | 0,353199989 | 0,340600014 | 0,255199999 | 0,251199991 |
|  | 340 | 0,256099999 | 0,25709999 | 0,396299988 | 0,397500008 | 0,375999987 | 0,375800014 | 0,358999997 | 0,345899999 | 0,256700009 | 0,252700001 |
|  | 360 | 0,256700009 | 0,25920001 | 0,407400012 | 0,405499995 | 0,382499993 | 0,384200007 | 0,36680001 | 0,352600008 | 0,256799996 | 0,251899987 |
| slope |  | -2,3684E-06 | -8,2508E-06 | 0,000444711 | 0,000424298 | 0,000348266 | 0,000376631 | 0,000333246 | 0,000292776 | 5,21053E-05 | 4,03302E-05 |
| slope w/o | | 0,000450021 | | | 0,000429608 | 0,000353576 | 0,00038194 | 0,000338555 | 0,000298086 | 5,74149E-05 | 4,56398E-05 |

|  | **5 min** | 30 |
| --- | --- | --- |
| **PTPN22 WT control** | 28,1331088 | 76,05790021 |
| **PTPN22 WT control+bicobnate** | 23,5239669 | 4,469147798 |
| **PTPN22 WT 30uM H2O2** | 20,3616485 | 97,26724967 |
| **PTPN22 WT 30uM H2O2+bicobnate** | 3,29599291 | 31,22145889 |

| Application: Tecan i-control  Device: infinite 200Pro | | Tecan i-control , 2.0.10.0  Serial number: 1307001123 | Serial number of connected stacker: |
| --- | --- | --- | --- |
| Firmware: V_3.40_01/15_Infinite (Dec 23 201 MAI, V_3.40_01/15_Infinite (Dec 23 2014/12.45.11) | | | |
| Date: | ######### | | |
| Time: | 11:39:06 | | |

System MTC-MU059-S

User MTC-MU059-S\fretho

Plate Greiner 96 Flat Bottom Transparent Polystyrene Cat. No.: 655101/655161/655192 [GRE96ft.pdfx] Plate-ID (Stacker)

List of actions in this measurement script: Kinetic

Absorbance

Shaking (Linear) Duration: Shaking (Linear) Amplitude:

3 s

1 mm

Label: Label1

Kinetic Measurement

Kinetic duration 00:06:00

Interval Time 00:00:20

Measurement Wavelength 405 nm

Bandwidth 10 nm

Number of Flashes 5

Settle Time 0 ms

Part of Plate G1-G10

Start Time: 2020 08-05 11:39:08

|  |  |  |  |  |  |  |  |  |  |  |  |  |  |  |  |  |  |  |  |
| --- | --- | --- | --- | --- | --- | --- | --- | --- | --- | --- | --- | --- | --- | --- | --- | --- | --- | --- | --- |
|  |  |  |  |  |  |  |  |  |  |  |  |  |  |  |  |  |  |  |  |
| Cycle Nr. | 1 | 2 | 3 | 4 | 5 | 6 | 7 | 8 | 9 | 10 | 11 | 12 | 13 | 14 | 15 | 16 | 17 | 18 | 19 |
| Time [s] | 0 | 20 | 40 | 60 | 80 | 100 | 120 | 140 | 160 | 180 | 200 | 220 | 240 | 260 | 280 | 300 | 320 | 340 | 360 |
| Temp. [°C] | 25,3 | 25,4 | 25,3 | 25,4 | 25,3 | 25,2 | 25,3 | 25,4 | 25,3 | 25,5 | 25,3 | 25,3 | 25,3 | 25,4 | 25,2 | 25,5 | 25,6 | 25,3 | 25,3 |
| G1 | 0,2533 | 0,2573 | 0,2633 | 0,2724 | 0,2834 | 0,2938 | 0,3007 | 0,311 | 0,3197 | 0,3286 | 0,3364 | 0,3458 | 0,3545 | 0,3644 | 0,3727 | 0,3813 | 0,3905 | 0,3963 | 0,4074 |
| G2 | 0,2551 | 0,2735 | 0,2668 | 0,2769 | 0,2836 | 0,2932 | 0,3024 | 0,3116 | 0,3223 | 0,33 | 0,339 | 0,3485 | 0,3565 | 0,3639 | 0,3737 | 0,3814 | 0,3904 | 0,3975 | 0,4055 |
| G3 | 0,253 | 0,2828 | 0,2627 | 0,2729 | 0,2797 | 0,2877 | 0,2944 | 0,304 | 0,3094 | 0,3164 | 0,3263 | 0,3323 | 0,3394 | 0,3457 | 0,355 | 0,3616 | 0,3678 | 0,376 | 0,3825 |
| G4 | 0,2533 | 0,257 | 0,2615 | 0,2724 | 0,2796 | 0,288 | 0,2934 | 0,3017 | 0,3066 | 0,3157 | 0,3253 | 0,3315 | 0,3383 | 0,3471 | 0,3559 | 0,3624 | 0,3691 | 0,3758 | 0,3842 |
| G5 | 0,2525 | 0,2535 | 0,2592 | 0,2676 | 0,2754 | 0,2821 | 0,2887 | 0,2951 | 0,3025 | 0,31 | 0,3162 | 0,3241 | 0,3292 | 0,3364 | 0,3423 | 0,3486 | 0,3532 | 0,359 | 0,3668 |
| G6 | 0,2535 | 0,2538 | 0,2582 | 0,2655 | 0,271 | 0,2756 | 0,281 | 0,2905 | 0,2957 | 0,3015 | 0,3079 | 0,3134 | 0,3194 | 0,3251 | 0,3302 | 0,3345 | 0,3406 | 0,3459 | 0,3526 |
| G7 | 0,2447 | 0,2419 | 0,2388 | 0,2432 | 0,2437 | 0,2437 | 0,2464 | 0,2494 | 0,2516 | 0,2531 | 0,2526 | 0,2547 | 0,2548 | 0,2531 | 0,256 | 0,2563 | 0,2552 | 0,2567 | 0,2568 |
| G8 | 0,2398 | 0,2404 | 0,2373 | 0,2417 | 0,2421 | 0,2427 | 0,2443 | 0,2462 | 0,2461 | 0,2469 | 0,2495 | 0,2481 | 0,2479 | 0,2498 | 0,2504 | 0,2518 | 0,2512 | 0,2527 | 0,2519 |
| G9 | 0,2615 | 0,2581 | 0,2548 | 0,2586 | 0,2582 | 0,2565 | 0,2561 | 0,2566 | 0,255 | 0,2554 | 0,2554 | 0,2568 | 0,2553 | 0,2553 | 0,2563 | 0,2572 | 0,2569 | 0,2561 | 0,2567 |
| G10 | 0,2616 | 0,2641 | 0,256 | 0,2587 | 0,2588 | 0,2601 | 0,2579 | 0,2589 | 0,2581 | 0,2571 | 0,2555 | 0,2589 | 0,2557 | 0,2584 | 0,2562 | 0,2566 | 0,257 | 0,2571 | 0,2592 |

End Time: 2020 08-05 11:45:19

**PTPN22 WT control PTPN22 WT control PTPN22 WT control+bicobnate PTPN22 WT control+bicobnate PTPN22 WT 30uM H2O2 PTPN22 WT 30uM H2O2 PTPN22 WT 30uM H2O2+bicobnate PTPN22 WT 30uM H2O2+bicobnate**

| blank |  | 0,000420263 | 0,000339466 | 0,00021781 | 7,69352E-06 |
| --- | --- | --- | --- | --- | --- |
|  |  | 0,025215791 | 0,020367958 | 0,013068577 | 0,000461611 |
|  |  | 3,763550842 | 3,039993717 | 1,950533838 | 0,0688972 |
|  |  | 26,88250602 | 21,71424084 | 13,93238456 | 0,492122856 |
|  | **min -1** | 26,88250602 | 21,71424084 | 13,93238456 | 0,492122856 |

**Time (sec) Blank Blank**

|  | 20 | 0,254700005 | 0,254400015 | 0,256199986 | 0,2861 | 0,263200015 | 0,250800014 | 0,244499996 | 0,247899994 | 0,235300004 | 0,235599995 |
| --- | --- | --- | --- | --- | --- | --- | --- | --- | --- | --- | --- |
|  | 40 | 0,256399989 | 0,256500006 | 0,265100002 | 0,264200002 | 0,260100007 | 0,260199994 | 0,2491 | 0,250999987 | 0,234799996 | 0,238600001 |
|  | 60 | 0,257299989 | 0,255800009 | 0,272599995 | 0,269899994 | 0,265799999 | 0,267399997 | 0,252700001 | 0,255400002 | 0,236200005 | 0,2368 |
|  | 80 | 0,25940001 | 0,259499997 | 0,282599986 | 0,280999988 | 0,275599986 | 0,27669999 | 0,256700009 | 0,260100007 | 0,237399995 | 0,239399999 |
|  | 100 | 0,257099986 | 0,257499993 | 0,291700006 | 0,288700014 | 0,281699985 | 0,282400012 | 0,263399988 | 0,265599996 | 0,239700004 | 0,2403 |
|  | 120 | 0,2597 | 0,25819999 | 0,300900012 | 0,297899991 | 0,287499994 | 0,287800014 | 0,265899986 | 0,268299997 | 0,240999997 | 0,241099998 |
|  | 140 | 0,257600009 | 0,256900012 | 0,311500013 | 0,305999994 | 0,294800013 | 0,297100008 | 0,274599999 | 0,275200009 | 0,241600007 | 0,241699994 |
|  | 160 | 0,256700009 | 0,255899996 | 0,3222 | 0,314399987 | 0,301999986 | 0,301899999 | 0,278600007 | 0,279500008 | 0,239800006 | 0,2403 |
|  | 180 | 0,256700009 | 0,256799996 | 0,329899997 | 0,322699994 | 0,308600008 | 0,310299993 | 0,282900006 | 0,284399986 | 0,238900006 | 0,240899995 |
|  | 200 | 0,256799996 | 0,256799996 | 0,337799996 | 0,330500007 | 0,314799994 | 0,316799998 | 0,284500003 | 0,287400007 | 0,238600001 | 0,240500003 |
|  | 220 | 0,254799992 | 0,256700009 | 0,347499996 | 0,337199986 | 0,320899993 | 0,3222 | 0,288199991 | 0,291000009 | 0,239500001 | 0,239500001 |
|  | 240 | 0,256099999 | 0,257099986 | 0,355599999 | 0,346199989 | 0,328200012 | 0,329899997 | 0,292699993 | 0,295300007 | 0,2377 | 0,238700002 |
|  | 260 | 0,256599993 | 0,257699996 | 0,364399999 | 0,356000006 | 0,336600006 | 0,336600006 | 0,298099995 | 0,301099986 | 0,238499999 | 0,238900006 |
|  | 280 | 0,255600005 | 0,256199986 | 0,372500002 | 0,362399995 | 0,341800004 | 0,345400006 | 0,300599992 | 0,303499997 | 0,238399997 | 0,240099996 |
|  | 300 | 0,255600005 | 0,254999995 | 0,38319999 | 0,370599985 | 0,348199993 | 0,350699991 | 0,3046 | 0,31099999 | 0,240199998 | 0,240199998 |
|  | 320 | 0,256500006 | 0,256399989 | 0,391499996 | 0,379700005 | 0,354499996 | 0,358200014 | 0,307500005 | 0,313199997 | 0,238499999 | 0,238499999 |
|  | 340 | 0,254599988 | 0,255299985 | 0,398699999 | 0,385899991 | 0,359899998 | 0,362399995 | 0,311199993 | 0,316300005 | 0,238600001 | 0,238700002 |
|  | 360 | 0,255499989 | 0,253500015 | 0,406100005 | 0,393299997 | 0,365700006 | 0,367500007 | 0,314300001 | 0,318699986 | 0,2359 | 0,236900002 |
| slope |  | -5,8772E-06 | -4,47888E-06 | 0,000447915 | 0,000382255 | 0,000325372 | 0,000343204 | 0,000208385 | 0,000216878 | 4,02476E-06 | 1,0062E-06 |
| slope w/o | | 0,000453093 | | | 0,000387433 | 0,00033055 | 0,000348382 | 0,000213563 | 0,000222056 | 9,2028E-06 | 6,18424E-06 |

|  | **15 min** | 30 |
| --- | --- | --- |
| **PTPN22 WT control** | 26,88250602 | 76,05790021 |
| **PTPN22 WT control+bicobnate** | 21,71424084 | 4,469147798 |
| **PTPN22 WT 30uM H2O2** | 13,93238456 | 97,26724967 |
| **PTPN22 WT 30uM H2O2+bicobnate** | 0,492122856 | 31,22145889 |

| Application: Tecan i-control  Device: infinite 200Pro | | Tecan i-control , 2.0.10.0  Serial number: 1307001123 | Serial number of connected stacker: |
| --- | --- | --- | --- |
| Firmware: V_3.40_01/15_Infinite (Dec 23 201 MAI, V_3.40_01/15_Infinite (Dec 23 2014/12.45.11) | | | |
| Date: | ######### | | |
| Time: | 11:49:19 | | |

System MTC-MU059-S

User MTC-MU059-S\fretho

Plate Greiner 96 Flat Bottom Transparent Polystyrene Cat. No.: 655101/655161/655192 [GRE96ft.pdfx] Plate-ID (Stacker)

List of actions in this measurement script: Kinetic

Absorbance

Shaking (Linear) Duration: Shaking (Linear) Amplitude:

3 s

1 mm

Label: Label1

Kinetic Measurement

Kinetic duration 00:06:00

Interval Time 00:00:20

Measurement Wavelength 405 nm

Bandwidth 10 nm

Number of Flashes 5

Settle Time 0 ms

Part of Plate G1-G10

Start Time: 2020 08-05 11:49:21

|  |  |  |  |  |  |  |  |  |  |  |  |  |  |  |  |  |  |  |  |
| --- | --- | --- | --- | --- | --- | --- | --- | --- | --- | --- | --- | --- | --- | --- | --- | --- | --- | --- | --- |
|  |  |  |  |  |  |  |  |  |  |  |  |  |  |  |  |  |  |  |  |
| Cycle Nr. | 1 | 2 | 3 | 4 | 5 | 6 | 7 | 8 | 9 | 10 | 11 | 12 | 13 | 14 | 15 | 16 | 17 | 18 | 19 |
| Time [s] | 0 | 20 | 40 | 60 | 80 | 100 | 120 | 140 | 160 | 180 | 200 | 220 | 240 | 260 | 280 | 300 | 320 | 340 | 360 |
| Temp. [°C] | 25,3 | 25,4 | 25,6 | 25,4 | 25,4 | 25,2 | 25,6 | 25,5 | 25,5 | 25,3 | 25,7 | 25,6 | 25,5 | 25,3 | 25,5 | 25,5 | 25,6 | 25,4 | 25,4 |
| G1 | 0,2532 | 0,2562 | 0,2651 | 0,2726 | 0,2826 | 0,2917 | 0,3009 | 0,3115 | 0,3222 | 0,3299 | 0,3378 | 0,3475 | 0,3556 | 0,3644 | 0,3725 | 0,3832 | 0,3915 | 0,3987 | 0,4061 |
| G2 | 0,2541 | 0,2861 | 0,2642 | 0,2699 | 0,281 | 0,2887 | 0,2979 | 0,306 | 0,3144 | 0,3227 | 0,3305 | 0,3372 | 0,3462 | 0,356 | 0,3624 | 0,3706 | 0,3797 | 0,3859 | 0,3933 |
| G3 | 0,2491 | 0,2632 | 0,2601 | 0,2658 | 0,2756 | 0,2817 | 0,2875 | 0,2948 | 0,302 | 0,3086 | 0,3148 | 0,3209 | 0,3282 | 0,3366 | 0,3418 | 0,3482 | 0,3545 | 0,3599 | 0,3657 |
| G4 | 0,2522 | 0,2508 | 0,2602 | 0,2674 | 0,2767 | 0,2824 | 0,2878 | 0,2971 | 0,3019 | 0,3103 | 0,3168 | 0,3222 | 0,3299 | 0,3366 | 0,3454 | 0,3507 | 0,3582 | 0,3624 | 0,3675 |
| G5 | 0,2462 | 0,2445 | 0,2491 | 0,2527 | 0,2567 | 0,2634 | 0,2659 | 0,2746 | 0,2786 | 0,2829 | 0,2845 | 0,2882 | 0,2927 | 0,2981 | 0,3006 | 0,3046 | 0,3075 | 0,3112 | 0,3143 |
| G6 | 0,2488 | 0,2479 | 0,251 | 0,2554 | 0,2601 | 0,2656 | 0,2683 | 0,2752 | 0,2795 | 0,2844 | 0,2874 | 0,291 | 0,2953 | 0,3011 | 0,3035 | 0,311 | 0,3132 | 0,3163 | 0,3187 |
| G7 | 0,2395 | 0,2353 | 0,2348 | 0,2362 | 0,2374 | 0,2397 | 0,241 | 0,2416 | 0,2398 | 0,2389 | 0,2386 | 0,2395 | 0,2377 | 0,2385 | 0,2384 | 0,2402 | 0,2385 | 0,2386 | 0,2359 |
| G8 | 0,2428 | 0,2356 | 0,2386 | 0,2368 | 0,2394 | 0,2403 | 0,2411 | 0,2417 | 0,2403 | 0,2409 | 0,2405 | 0,2395 | 0,2387 | 0,2389 | 0,2401 | 0,2402 | 0,2385 | 0,2387 | 0,2369 |
| G9 | 0,2598 | 0,2547 | 0,2564 | 0,2573 | 0,2594 | 0,2571 | 0,2597 | 0,2576 | 0,2567 | 0,2567 | 0,2568 | 0,2548 | 0,2561 | 0,2566 | 0,2556 | 0,2556 | 0,2565 | 0,2546 | 0,2555 |
| G10 | 0,2591 | 0,2544 | 0,2565 | 0,2558 | 0,2595 | 0,2575 | 0,2582 | 0,2569 | 0,2559 | 0,2568 | 0,2568 | 0,2567 | 0,2571 | 0,2577 | 0,2562 | 0,255 | 0,2564 | 0,2553 | 0,2535 |

End Time: 2020 08-05 11:55:31

| Application: Tecan i-control  Device: infinite 200Pro | | Tecan i-control , 2.0.10.0  Serial number: 1307001123 | Serial number of connected stacker: |
| --- | --- | --- | --- |
| Firmware: V_3.40_01/15_Infinite (Dec 23 201 MAI, V_3.40_01/15_Infinite (Dec 23 2014/12.45.11) | | | |
| Date: | ######### | | |
| Time: | 12:04:23 | | |

System MTC-MU059-S

User MTC-MU059-S\fretho

Plate Greiner 96 Flat Bottom Transparent Polystyrene Cat. No.: 655101/655161/655192 [GRE96ft.pdfx] Plate-ID (Stacker)

List of actions in this measurement script: Kinetic

Absorbance

Shaking (Linear) Duration: Shaking (Linear) Amplitude:

3 s

1 mm

Label: Label1

Kinetic Measurement

Kinetic duration 00:06:00

Interval Time 00:00:20

Measurement Wavelength 405 nm

Bandwidth 10 nm

Number of Flashes 5

Settle Time 0 ms

Part of Plate G1-G10

Start Time: 2020 08-05 12:04:25

|  |  |  |  |  |  |  |  |  |  |  |  |  |  |  |  |  |  |  |  |
| --- | --- | --- | --- | --- | --- | --- | --- | --- | --- | --- | --- | --- | --- | --- | --- | --- | --- | --- | --- |
|  |  |  |  |  |  |  |  |  |  |  |  |  |  |  |  |  |  |  |  |
| Cycle Nr. | 1 | 2 | 3 | 4 | 5 | 6 | 7 | 8 | 9 | 10 | 11 | 12 | 13 | 14 | 15 | 16 | 17 | 18 | 19 |
| Time [s] | 0 | 20 | 40 | 60 | 80 | 100 | 120 | 140 | 160 | 180 | 200 | 220 | 240 | 260 | 280 | 300 | 320 | 340 | 360 |
| Temp. [°C] | 25,6 | 25,6 | 25,6 | 25,7 | 25,7 | 25,7 | 25,7 | 25,6 | 25,6 | 25,6 | 25,8 | 25,8 | 25,6 | 25,7 | 25,7 | 25,9 | 25,7 | 25,7 | 25,6 |
| G1 | 0,2529 | 0,2606 | 0,2655 | 0,2728 | 0,2826 | 0,2896 | 0,2979 | 0,3073 | 0,3164 | 0,3243 | 0,3328 | 0,3419 | 0,3486 | 0,3592 | 0,3671 | 0,3749 | 0,3805 | 0,3891 | 0,397 |
| G2 | 0,2546 | 0,2643 | 0,2775 | 0,2789 | 0,2888 | 0,2983 | 0,3093 | 0,3194 | 0,33 | 0,3381 | 0,3477 | 0,358 | 0,3675 | 0,3778 | 0,3855 | 0,3944 | 0,4028 | 0,4122 | 0,4216 |
| G3 | 0,249 | 0,2688 | 0,2704 | 0,2654 | 0,2759 | 0,2787 | 0,2857 | 0,2923 | 0,2984 | 0,3058 | 0,3104 | 0,3183 | 0,3235 | 0,3308 | 0,3361 | 0,3423 | 0,3476 | 0,3526 | 0,3591 |
| G4 | 0,251 | 0,2692 | 0,2626 | 0,2704 | 0,2739 | 0,2805 | 0,2876 | 0,295 | 0,3018 | 0,3091 | 0,3161 | 0,3223 | 0,3291 | 0,3371 | 0,3434 | 0,3477 | 0,3534 | 0,3594 | 0,3659 |
| G5 | 0,2454 | 0,245 | 0,2483 | 0,2509 | 0,2529 | 0,2562 | 0,26 | 0,2647 | 0,2669 | 0,2708 | 0,2736 | 0,2768 | 0,2795 | 0,2836 | 0,2857 | 0,2875 | 0,2907 | 0,2932 | 0,2962 |
| G6 | 0,2466 | 0,248 | 0,2513 | 0,2541 | 0,2566 | 0,2619 | 0,2662 | 0,2707 | 0,2736 | 0,2774 | 0,2819 | 0,2862 | 0,2887 | 0,2931 | 0,295 | 0,2984 | 0,3045 | 0,3052 | 0,3089 |
| G7 | 0,2418 | 0,2406 | 0,2395 | 0,2402 | 0,2393 | 0,2419 | 0,2418 | 0,2429 | 0,2417 | 0,2418 | 0,2411 | 0,242 | 0,2412 | 0,2425 | 0,241 | 0,2419 | 0,2401 | 0,2406 | 0,2391 |
| G8 | 0,2436 | 0,2415 | 0,2408 | 0,2403 | 0,242 | 0,2409 | 0,2433 | 0,2426 | 0,242 | 0,2425 | 0,2425 | 0,2429 | 0,2409 | 0,2437 | 0,2441 | 0,2415 | 0,2409 | 0,2421 | 0,2407 |
| G9 | 0,258 | 0,2584 | 0,2586 | 0,2594 | 0,2586 | 0,2594 | 0,2588 | 0,2575 | 0,2584 | 0,2591 | 0,2569 | 0,2586 | 0,2587 | 0,2607 | 0,2598 | 0,2582 | 0,2576 | 0,2568 | 0,2597 |
| G10 | 0,2618 | 0,2593 | 0,2595 | 0,2597 | 0,259 | 0,2584 | 0,2604 | 0,2597 | 0,2589 | 0,2613 | 0,2619 | 0,2597 | 0,2618 | 0,2621 | 0,2615 | 0,261 | 0,2605 | 0,263 | 0,26 |

End Time: 2020 08-05 12:10:35

|  | | | **PTPN22 WT control** | **PTPN22 WT control** | **PTPN22 WT control+bicobnate** | **PTPN22 WT control+bicobnate** | **PTPN22 WT 30uM H2O2** | **PTPN22 WT 30uM H2O2** | **PTPN22 WT 30uM H2O2+bicobnate PTPN22 WT 30uM** | **H2O2+bicobnate** |
| --- | --- | --- | --- | --- | --- | --- | --- | --- | --- | --- |
| **Time (sec) Blank** |  | **Blank** |  |  |  |  |  |  |  |  |
| 20 | 0,258399993 | 0,259299994 | 0,260600001 | 0,264299989 | 0,26879999 | 0,269199997 | 0,245000005 | 0,247999996 | 0,240600005 | 0,241500005 |
| 40 | 0,258599997 | 0,259499997 | 0,265500009 | 0,277500004 | 0,270399988 | 0,262600005 | 0,248300001 | 0,251300007 | 0,239500001 | 0,240799993 |
| 60 | 0,25940001 | 0,2597 | 0,272799999 | 0,278899997 | 0,265399992 | 0,270399988 | 0,2509 | 0,254099995 | 0,240199998 | 0,2403 |
| 80 | 0,258599997 | 0,259000003 | 0,282599986 | 0,288800001 | 0,275900006 | 0,273900002 | 0,252900004 | 0,256599993 | 0,239299998 | 0,241999999 |
| 100 | 0,25940001 | 0,258399993 | 0,289600015 | 0,298299998 | 0,278699994 | 0,280499995 | 0,256199986 | 0,261900008 | 0,241899997 | 0,240899995 |
| 120 | 0,2588 | 0,260399997 | 0,297899991 | 0,309300005 | 0,285699993 | 0,287600011 | 0,25999999 | 0,266200006 | 0,241799995 | 0,243300006 |
| 140 | 0,257499993 | 0,2597 | 0,307300001 | 0,319400012 | 0,292299986 | 0,294999987 | 0,264699996 | 0,270700008 | 0,242899999 | 0,242599994 |
| 160 | 0,258399993 | 0,258899987 | 0,316399992 | 0,330000013 | 0,298400015 | 0,301800013 | 0,266900003 | 0,273600012 | 0,241699994 | 0,241999999 |
| 180 | 0,25909999 | 0,261299998 | 0,324299991 | 0,338099986 | 0,305799991 | 0,309100002 | 0,270799994 | 0,277399987 | 0,241799995 | 0,242500007 |
| 200 | 0,256900012 | 0,261900008 | 0,332800001 | 0,3477 | 0,310400009 | 0,316100001 | 0,273600012 | 0,281899989 | 0,241099998 | 0,242500007 |
| 220 | 0,258599997 | 0,2597 | 0,341899991 | 0,35800001 | 0,318300009 | 0,322299987 | 0,276800007 | 0,286199987 | 0,241999999 | 0,242899999 |
| 240 | 0,258700013 | 0,261799991 | 0,3486 | 0,367500007 | 0,323500007 | 0,329100013 | 0,279500008 | 0,288700014 | 0,2412 | 0,240899995 |
| 260 | 0,260699987 | 0,262100011 | 0,359200001 | 0,377799988 | 0,330799997 | 0,337099999 | 0,283600003 | 0,293099999 | 0,242500007 | 0,243699998 |
| 280 | 0,259799987 | 0,261500001 | 0,3671 | 0,385500014 | 0,336100012 | 0,343400002 | 0,285699993 | 0,294999987 | 0,240999997 | 0,244100004 |
| 300 | 0,25819999 | 0,261000007 | 0,374900013 | 0,394400001 | 0,342299998 | 0,3477 | 0,287499994 | 0,298400015 | 0,241899997 | 0,241500005 |
| 320 | 0,257600009 | 0,260500014 | 0,380499989 | 0,402799994 | 0,347600013 | 0,353399992 | 0,290699989 | 0,304500014 | 0,240099996 | 0,240899995 |
| 340 | 0,256799996 | 0,263000011 | 0,389099985 | 0,412200004 | 0,352600008 | 0,359400004 | 0,293199986 | 0,305200011 | 0,240600005 | 0,2421 |
| 360 | 0,2597 | 0,25999999 | 0,397000015 | 0,421600014 | 0,359100014 | 0,36590001 | 0,296200007 | 0,308899999 | 0,239099994 | 0,240700006 |
| slope | -5,88242E-07 | 7,74513E-06 | 0,000413122 | 0,000467766 | 0,000289974 | 0,000316734 | 0,00015372 | 0,000184587 | 3,50875E-07 | 1,75439E-06 |
| slope w/o |  |  | 0,000409543 | 0,000464187 | 0,000286396 | 0,000313155 | 0,000150142 | 0,000181009 | -3,22757E-06 | -1,82405E-06 |

| blank |  | 0,000436865 | 0,000299776 | 0,000165575 | -2,52581E-06 |
| --- | --- | --- | --- | --- | --- |
|  |  | 0,026211919 | 0,017986534 | 0,00993452 | -0,000151548 |
|  |  | 3,912226781 | 2,684557327 | 1,482764124 | -0,022619172 |
|  |  | 27,944477 | 19,17540948 | 10,59117231 | -0,161565516 |
|  | **min -1** | 27,944477 | 19,17540948 | 10,59117231 | -0,161565516 |

|  | **30 min** | 30 |
| --- | --- | --- |
| **PTPN22 WT control** | 27,944477 | 76,05790021 |
| **PTPN22 WT control+bicobnate** | 19,17540948 | 4,469147798 |
| **PTPN22 WT 30uM H2O2** | 10,59117231 | 97,26724967 |
| **PTPN22 WT 30uM H2O2+bicobnate** | -0,16156552 | 31,22145889 |

**PTPN22 WT control PTPN22 WT control PTPN22 WT control+bicobnaPTPN22 WT control+bicobna PTPN22 WT 30uM H2O2 PTPN22 WT 30uM H2O2 PTPN22 WT 30uM H2O2+bicobnate PTPN22 WT 30uM H2O2+bicobnate**

| blank |  | 0,00049137 | 0,000318395 | 0,000309368 | 4,08385E-05 |
| --- | --- | --- | --- | --- | --- |
|  |  | 0,029482198 | 0,019103714 | 0,018562075 | 0,002450309 |
|  |  | 4,400328021 | 2,851300652 | 2,770458989 | 0,36571782 |
|  |  | 31,43091444 | 20,36643323 | 19,78899278 | 2,612270143 |
|  | **min -1** | 31,43091444 | 20,36643323 | 19,78899278 | 2,612270143 |

**Time (sec) Blank Blank**

|  | 20 | 0,250400007 | 0,245800003 | 0,256700009 | 0,265300006 | 0,277799994 | 0,252299994 | 0,248500004 | 0,250400007 | 0,233600006 | 0,234799996 |
| --- | --- | --- | --- | --- | --- | --- | --- | --- | --- | --- | --- |
|  | 40 | 0,252000004 | 0,246199995 | 0,264299989 | 0,266600013 | 0,256000012 | 0,258399993 | 0,254500002 | 0,256799996 | 0,234300002 | 0,237100005 |
|  | 60 | 0,250800014 | 0,247099996 | 0,272599995 | 0,275200009 | 0,261400014 | 0,265100002 | 0,260500014 | 0,260699987 | 0,233600006 | 0,235799998 |
|  | 80 | 0,249500006 | 0,248699993 | 0,2852 | 0,285100013 | 0,269699991 | 0,271699995 | 0,269199997 | 0,267399997 | 0,234999999 | 0,239099994 |
|  | 100 | 0,250499994 | 0,246900007 | 0,293599993 | 0,294499993 | 0,275099993 | 0,279100001 | 0,274100006 | 0,275299996 | 0,236499995 | 0,241600007 |
|  | 120 | 0,25029999 | 0,246199995 | 0,305000007 | 0,304899991 | 0,282099992 | 0,286500007 | 0,279399991 | 0,282099992 | 0,237100005 | 0,239600003 |
|  | 140 | 0,249400005 | 0,247799993 | 0,314700007 | 0,314799994 | 0,288399994 | 0,29460001 | 0,288100004 | 0,289600015 | 0,2377 | 0,240400001 |
|  | 160 | 0,250999987 | 0,248199999 | 0,326200008 | 0,324699998 | 0,296700001 | 0,301699996 | 0,294200003 | 0,294400007 | 0,239600003 | 0,240600005 |
|  | 180 | 0,250200003 | 0,248600006 | 0,338099986 | 0,335099995 | 0,304100007 | 0,309599996 | 0,299699992 | 0,300799996 | 0,240199998 | 0,242899999 |
|  | 200 | 0,247600004 | 0,251599997 | 0,345699996 | 0,344399989 | 0,309399992 | 0,315100014 | 0,307000011 | 0,310000002 | 0,240700006 | 0,243599996 |
|  | 220 | 0,247600004 | 0,248300001 | 0,355800003 | 0,353599995 | 0,314599991 | 0,32069999 | 0,31099999 | 0,3134 | 0,240199998 | 0,245000005 |
|  | 240 | 0,255199999 | 0,248300001 | 0,366100013 | 0,362899989 | 0,321099997 | 0,328599989 | 0,319200009 | 0,319299996 | 0,241099998 | 0,244299993 |
|  | 260 | 0,248099998 | 0,249200001 | 0,376899987 | 0,372200012 | 0,326499999 | 0,333900005 | 0,323399991 | 0,323300004 | 0,242799997 | 0,244399995 |
|  | 280 | 0,248300001 | 0,245499998 | 0,387400001 | 0,381599993 | 0,335299999 | 0,342599988 | 0,3301 | 0,330500007 | 0,244399995 | 0,246099994 |
|  | 300 | 0,247799993 | 0,245499998 | 0,396800011 | 0,389899999 | 0,337799996 | 0,346899986 | 0,333900005 | 0,337500006 | 0,244000003 | 0,246199995 |
|  | 320 | 0,248500004 | 0,2456 | 0,405800015 | 0,398600012 | 0,343899995 | 0,353799999 | 0,339700013 | 0,342299998 | 0,243699998 | 0,246700004 |
|  | 340 | 0,247400001 | 0,251199991 | 0,416399986 | 0,406899989 | 0,350699991 | 0,359699994 | 0,344999999 | 0,347000003 | 0,246600002 | 0,247400001 |
|  | 360 | 0,246900007 | 0,246600002 | 0,428200006 | 0,417499989 | 0,354999989 | 0,365700006 | 0,353300005 | 0,353500009 | 0,245000005 | 0,247400001 |
| slope |  | -1,01187E-05 | 2,48194E-06 | 0,000508622 | 0,000466481 | 0,000290485 | 0,000338669 | 0,000304727 | 0,000306373 | 3,81269E-05 | 3,59133E-05 |
| slope w/o | | 0,000512441 | | | 0,000470299 | 0,000294303 | 0,000342487 | 0,000308545 | 0,000310191 | 4,19453E-05 | 3,97317E-05 |

|  | **5 min** | 30 |
| --- | --- | --- |
| **PTPN22 WT control** | 31,43091444 | 76,05790021 |
| **PTPN22 WT control+bicobnate** | 20,36643323 | 4,469147798 |
| **PTPN22 WT 30uM H2O2** | 19,78899278 | 97,26724967 |
| **PTPN22 WT 30uM H2O2+bicobnate** | 2,612270143 | 31,22145889 |

| Application: Tecan i-control  Device: infinite 200Pro | | Tecan i-control , 2.0.10.0  Serial number: 1307001123 | Serial number of connected stacker: |
| --- | --- | --- | --- |
| Firmware: V_3.40_01/15_Infinite (Dec 23 201 MAI, V_3.40_01/15_Infinite (Dec 23 2014/12.45.11) | | | |
| Date: | ######### | | |
| Time: | 12:59:59 | | |

System MTC-MU059-S

User MTC-MU059-S\fretho

Plate Greiner 96 Flat Bottom Transparent Polystyrene Cat. No.: 655101/655161/655192 [GRE96ft.pdfx] Plate-ID (Stacker)

List of actions in this measurement script: Kinetic

Absorbance

Shaking (Linear) Duration: Shaking (Linear) Amplitude:

3 s

1 mm

Label: Label1

Kinetic Measurement

Kinetic duration 00:06:00

Interval Time 00:00:20

Measurement Wavelength 405 nm

Bandwidth 10 nm

Number of Flashes 5

Settle Time 0 ms

Part of Plate H1-H10

Start Time: 2020 08-05 13:00:01

|  |  |  |  |  |  |  |  |  |  |  |  |  |  |  |  |  |  |  |  |
| --- | --- | --- | --- | --- | --- | --- | --- | --- | --- | --- | --- | --- | --- | --- | --- | --- | --- | --- | --- |
|  |  |  |  |  |  |  |  |  |  |  |  |  |  |  |  |  |  |  |  |
| Cycle Nr. | 1 | 2 | 3 | 4 | 5 | 6 | 7 | 8 | 9 | 10 | 11 | 12 | 13 | 14 | 15 | 16 | 17 | 18 | 19 |
| Time [s] | 0 | 20 | 40 | 60 | 80 | 100 | 120 | 140 | 160 | 180 | 200 | 220 | 240 | 260 | 280 | 300 | 320 | 340 | 360 |
| Temp. [°C] | 25,5 | 25,4 | 25,6 | 25,4 | 25,8 | 25,2 | 25,5 | 25,6 | 25,4 | 25,6 | 25,7 | 25,3 | 25,5 | 25,3 | 25,4 | 25,7 | 25,4 | 25,6 | 25,6 |
| H1 | 0,2463 | 0,2567 | 0,2643 | 0,2726 | 0,2852 | 0,2936 | 0,305 | 0,3147 | 0,3262 | 0,3381 | 0,3457 | 0,3558 | 0,3661 | 0,3769 | 0,3874 | 0,3968 | 0,4058 | 0,4164 | 0,4282 |
| H2 | 0,2495 | 0,2653 | 0,2666 | 0,2752 | 0,2851 | 0,2945 | 0,3049 | 0,3148 | 0,3247 | 0,3351 | 0,3444 | 0,3536 | 0,3629 | 0,3722 | 0,3816 | 0,3899 | 0,3986 | 0,4069 | 0,4175 |
| H3 | 0,2488 | 0,2778 | 0,256 | 0,2614 | 0,2697 | 0,2751 | 0,2821 | 0,2884 | 0,2967 | 0,3041 | 0,3094 | 0,3146 | 0,3211 | 0,3265 | 0,3353 | 0,3378 | 0,3439 | 0,3507 | 0,355 |
| H4 | 0,2518 | 0,2523 | 0,2584 | 0,2651 | 0,2717 | 0,2791 | 0,2865 | 0,2946 | 0,3017 | 0,3096 | 0,3151 | 0,3207 | 0,3286 | 0,3339 | 0,3426 | 0,3469 | 0,3538 | 0,3597 | 0,3657 |
| H5 | 0,2498 | 0,2485 | 0,2545 | 0,2605 | 0,2692 | 0,2741 | 0,2794 | 0,2881 | 0,2942 | 0,2997 | 0,307 | 0,311 | 0,3192 | 0,3234 | 0,3301 | 0,3339 | 0,3397 | 0,345 | 0,3533 |
| H6 | 0,247 | 0,2504 | 0,2568 | 0,2607 | 0,2674 | 0,2753 | 0,2821 | 0,2896 | 0,2944 | 0,3008 | 0,31 | 0,3134 | 0,3193 | 0,3233 | 0,3305 | 0,3375 | 0,3423 | 0,347 | 0,3535 |
| H7 | 0,2381 | 0,2336 | 0,2343 | 0,2336 | 0,235 | 0,2365 | 0,2371 | 0,2377 | 0,2396 | 0,2402 | 0,2407 | 0,2402 | 0,2411 | 0,2428 | 0,2444 | 0,244 | 0,2437 | 0,2466 | 0,245 |
| H8 | 0,2351 | 0,2348 | 0,2371 | 0,2358 | 0,2391 | 0,2416 | 0,2396 | 0,2404 | 0,2406 | 0,2429 | 0,2436 | 0,245 | 0,2443 | 0,2444 | 0,2461 | 0,2462 | 0,2467 | 0,2474 | 0,2474 |
| H9 | 0,2512 | 0,2504 | 0,252 | 0,2508 | 0,2495 | 0,2505 | 0,2503 | 0,2494 | 0,251 | 0,2502 | 0,2476 | 0,2476 | 0,2552 | 0,2481 | 0,2483 | 0,2478 | 0,2485 | 0,2474 | 0,2469 |
| H10 | 0,2472 | 0,2458 | 0,2462 | 0,2471 | 0,2487 | 0,2469 | 0,2462 | 0,2478 | 0,2482 | 0,2486 | 0,2516 | 0,2483 | 0,2483 | 0,2492 | 0,2455 | 0,2455 | 0,2456 | 0,2512 | 0,2466 |

End Time: 2020 08-05 13:06:12

**PTPN22 WT control PTPN22 WT control PTPN22 WT control+bicobnate PTPN22 WT control+bicobnate PTPN22 WT 30uM H2O2 PTPN22 WT 30uM H2O2 PTPN22 WT 30uM H2O2+bicobnate PTPN22 WT 30uM H2O2+bicobnate**

| blank |  | 0,000478875 | 0,000354662 | 0,000227116 | 5,83849E-06 |
| --- | --- | --- | --- | --- | --- |
|  |  | 0,028732509 | 0,021279722 | 0,013626936 | 0,000350309 |
|  |  | 4,288434113 | 3,176077937 | 2,033870994 | 0,052284994 |
|  |  | 30,63167224 | 22,68627098 | 14,52764996 | 0,373464244 |
|  | **min -1** | 30,63167224 | 22,68627098 | 14,52764996 | 0,373464244 |

**Time (sec) Blank Blank**

|  | 20 | 0,260800004 | 0,245900005 | 0,25909999 | 0,256700009 | 0,275400013 | 0,251199991 | 0,242400005 | 0,244299993 | 0,228400007 | 0,228100002 |
| --- | --- | --- | --- | --- | --- | --- | --- | --- | --- | --- | --- |
|  | 40 | 0,26030001 | 0,245900005 | 0,268999994 | 0,272199988 | 0,263099998 | 0,260600001 | 0,246800005 | 0,248099998 | 0,228400007 | 0,230299994 |
|  | 60 | 0,256799996 | 0,2456 | 0,278299987 | 0,270300001 | 0,270399988 | 0,26730001 | 0,252200007 | 0,252799988 | 0,228599995 | 0,230100006 |
|  | 80 | 0,256799996 | 0,245800003 | 0,288599998 | 0,286300004 | 0,277799994 | 0,273999989 | 0,25819999 | 0,257299989 | 0,2324 | 0,231399998 |
|  | 100 | 0,256700009 | 0,246099994 | 0,298799992 | 0,293500006 | 0,282499999 | 0,28459999 | 0,260399997 | 0,262699991 | 0,231199995 | 0,2324 |
|  | 120 | 0,255699992 | 0,245499998 | 0,309300005 | 0,310000002 | 0,290300012 | 0,290100008 | 0,266299993 | 0,267699987 | 0,231900007 | 0,231999993 |
|  | 140 | 0,254999995 | 0,244499996 | 0,319000006 | 0,315299988 | 0,2958 | 0,298400015 | 0,272000015 | 0,271499991 | 0,230399996 | 0,233199999 |
|  | 160 | 0,255299985 | 0,245100006 | 0,332100004 | 0,323599994 | 0,303299993 | 0,306699991 | 0,27579999 | 0,27669999 | 0,231900007 | 0,232299998 |
|  | 180 | 0,255600005 | 0,2456 | 0,343899995 | 0,332199991 | 0,312000006 | 0,315200001 | 0,278400004 | 0,283199996 | 0,232800007 | 0,231800005 |
|  | 200 | 0,254599988 | 0,2447 | 0,351799995 | 0,338800013 | 0,317200005 | 0,32280001 | 0,281800002 | 0,287 | 0,231000006 | 0,230900005 |
|  | 220 | 0,255299985 | 0,245700002 | 0,362100005 | 0,344599992 | 0,323799998 | 0,328099996 | 0,285699993 | 0,291000009 | 0,229800001 | 0,230499998 |
|  | 240 | 0,255800009 | 0,246199995 | 0,372799993 | 0,3574 | 0,332100004 | 0,336899996 | 0,290800005 | 0,297199994 | 0,229699999 | 0,231000006 |
|  | 260 | 0,255100012 | 0,244499996 | 0,382800013 | 0,364300013 | 0,337900013 | 0,345299989 | 0,294099987 | 0,300300002 | 0,229499996 | 0,230199993 |
|  | 280 | 0,254299998 | 0,244800001 | 0,394199997 | 0,370200008 | 0,344000012 | 0,350899994 | 0,298099995 | 0,304399997 | 0,229699999 | 0,229499996 |
|  | 300 | 0,254900008 | 0,244800001 | 0,403800011 | 0,377799988 | 0,350800008 | 0,358700007 | 0,3028 | 0,310699999 | 0,230299994 | 0,230000004 |
|  | 320 | 0,257200003 | 0,247299999 | 0,416900009 | 0,388099998 | 0,358799994 | 0,365500003 | 0,306300014 | 0,314500004 | 0,230900005 | 0,229900002 |
|  | 340 | 0,255800009 | 0,2447 | 0,425199986 | 0,394499987 | 0,363799989 | 0,371800005 | 0,310200006 | 0,318300009 | 0,229800001 | 0,229399994 |
|  | 360 | 0,254599988 | 0,244200006 | 0,433800012 | 0,4014 | 0,369500011 | 0,378800005 | 0,313499987 | 0,321399987 | 0,230900005 | 0,229800001 |
| slope |  | -1,09082E-05 | -2,32714E-06 | 0,000522286 | 0,000422229 | 0,000319778 | 0,000376311 | 0,000207152 | 0,000233844 | 1,46542E-06 | -3,02374E-06 |
| slope w/o | | 0,000528904 | | | 0,000428847 | 0,000326396 | 0,000382928 | 0,000213769 | 0,000240462 | 8,08307E-06 | 3,59391E-06 |

|  | **15 min** | 30 |
| --- | --- | --- |
| **PTPN22 WT control** | 30,63167224 | 76,05790021 |
| **PTPN22 WT control+bicobnate** | 22,68627098 | 4,469147798 |
| **PTPN22 WT 30uM H2O2** | 14,52764996 | 97,26724967 |
| **PTPN22 WT 30uM H2O2+bicobnate** | 0,373464244 | 31,22145889 |

| Application: Tecan i-control  Device: infinite 200Pro | | Tecan i-control , 2.0.10.0  Serial number: 1307001123 | Serial number of connected stacker: |
| --- | --- | --- | --- |
| Firmware: V_3.40_01/15_Infinite (Dec 23 201 MAI, V_3.40_01/15_Infinite (Dec 23 2014/12.45.11) | | | |
| Date: | ######### | | |
| Time: | 13:10:00 | | |

System MTC-MU059-S

User MTC-MU059-S\fretho

Plate Greiner 96 Flat Bottom Transparent Polystyrene Cat. No.: 655101/655161/655192 [GRE96ft.pdfx] Plate-ID (Stacker)

List of actions in this measurement script: Kinetic

Absorbance

Shaking (Linear) Duration: Shaking (Linear) Amplitude:

3 s

1 mm

Label: Label1

Kinetic Measurement

Kinetic duration 00:06:00

Interval Time 00:00:20

Measurement Wavelength 405 nm

Bandwidth 10 nm

Number of Flashes 5

Settle Time 0 ms

Part of Plate H1-H10

Start Time: 2020 08-05 13:10:02

|  |  |  |  |  |  |  |  |  |  |  |  |  |  |  |  |  |  |  |  |
| --- | --- | --- | --- | --- | --- | --- | --- | --- | --- | --- | --- | --- | --- | --- | --- | --- | --- | --- | --- |
|  |  |  |  |  |  |  |  |  |  |  |  |  |  |  |  |  |  |  |  |
| Cycle Nr. | 1 | 2 | 3 | 4 | 5 | 6 | 7 | 8 | 9 | 10 | 11 | 12 | 13 | 14 | 15 | 16 | 17 | 18 | 19 |
| Time [s] | 0 | 20 | 40 | 60 | 80 | 100 | 120 | 140 | 160 | 180 | 200 | 220 | 240 | 260 | 280 | 300 | 320 | 340 | 360 |
| Temp. [°C] | 25,5 | 25,7 | 25,7 | 25,7 | 25,4 | 25,7 | 25,8 | 25,6 | 25,7 | 25,6 | 25,8 | 25,6 | 25,5 | 25,7 | 25,7 | 25,7 | 25,6 | 25,7 | 25,9 |
| H1 | 0,2525 | 0,2591 | 0,269 | 0,2783 | 0,2886 | 0,2988 | 0,3093 | 0,319 | 0,3321 | 0,3439 | 0,3518 | 0,3621 | 0,3728 | 0,3828 | 0,3942 | 0,4038 | 0,4169 | 0,4252 | 0,4338 |
| H2 | 0,2492 | 0,2567 | 0,2722 | 0,2703 | 0,2863 | 0,2935 | 0,31 | 0,3153 | 0,3236 | 0,3322 | 0,3388 | 0,3446 | 0,3574 | 0,3643 | 0,3702 | 0,3778 | 0,3881 | 0,3945 | 0,4014 |
| H3 | 0,2541 | 0,2754 | 0,2631 | 0,2704 | 0,2778 | 0,2825 | 0,2903 | 0,2958 | 0,3033 | 0,312 | 0,3172 | 0,3238 | 0,3321 | 0,3379 | 0,344 | 0,3508 | 0,3588 | 0,3638 | 0,3695 |
| H4 | 0,2501 | 0,2512 | 0,2606 | 0,2673 | 0,274 | 0,2846 | 0,2901 | 0,2984 | 0,3067 | 0,3152 | 0,3228 | 0,3281 | 0,3369 | 0,3453 | 0,3509 | 0,3587 | 0,3655 | 0,3718 | 0,3788 |
| H5 | 0,243 | 0,2424 | 0,2468 | 0,2522 | 0,2582 | 0,2604 | 0,2663 | 0,272 | 0,2758 | 0,2784 | 0,2818 | 0,2857 | 0,2908 | 0,2941 | 0,2981 | 0,3028 | 0,3063 | 0,3102 | 0,3135 |
| H6 | 0,2439 | 0,2443 | 0,2481 | 0,2528 | 0,2573 | 0,2627 | 0,2677 | 0,2715 | 0,2767 | 0,2832 | 0,287 | 0,291 | 0,2972 | 0,3003 | 0,3044 | 0,3107 | 0,3145 | 0,3183 | 0,3214 |
| H7 | 0,2334 | 0,2284 | 0,2284 | 0,2286 | 0,2324 | 0,2312 | 0,2319 | 0,2304 | 0,2319 | 0,2328 | 0,231 | 0,2298 | 0,2297 | 0,2295 | 0,2297 | 0,2303 | 0,2309 | 0,2298 | 0,2309 |
| H8 | 0,2307 | 0,2281 | 0,2303 | 0,2301 | 0,2314 | 0,2324 | 0,232 | 0,2332 | 0,2323 | 0,2318 | 0,2309 | 0,2305 | 0,231 | 0,2302 | 0,2295 | 0,23 | 0,2299 | 0,2294 | 0,2298 |
| H9 | 0,2618 | 0,2608 | 0,2603 | 0,2568 | 0,2568 | 0,2567 | 0,2557 | 0,255 | 0,2553 | 0,2556 | 0,2546 | 0,2553 | 0,2558 | 0,2551 | 0,2543 | 0,2549 | 0,2572 | 0,2558 | 0,2546 |
| H10 | 0,2477 | 0,2459 | 0,2459 | 0,2456 | 0,2458 | 0,2461 | 0,2455 | 0,2445 | 0,2451 | 0,2456 | 0,2447 | 0,2457 | 0,2462 | 0,2445 | 0,2448 | 0,2448 | 0,2473 | 0,2447 | 0,2442 |

End Time: 2020 08-05 13:16:12

|  | | **PTPN22 WT control** | **PTPN22 WT control** | **PTPN22 WT control+bicobnate** | **PTPN22 WT control+bicobnate** | **PTPN22 WT 30uM H2O2** | **PTPN22 WT 30uM H2O2** | **PTPN22 WT 30uM H2O2+bicobnate PTPN22 WT 30uM** | **H2O2+bicobnate** |
| --- | --- | --- | --- | --- | --- | --- | --- | --- | --- |
| **Time (sec) Blank** | **Blank** |  |  |  |  |  |  |  |  |
| 20 | 0,251100004 0,248600006 | 0,256399989 | 0,254799992 | 0,248699993 | 0,250400007 | 0,239800006 | 0,245299995 | 0,229499996 | 0,231199995 |
| 40 | 0,25029999 0,248600006 | 0,261099994 | 0,262800008 | 0,254599988 | 0,257400006 | 0,242899999 | 0,246299997 | 0,230100006 | 0,2315 |
| 60 | 0,250400007 0,251300007 | 0,268900007 | 0,272000015 | 0,262100011 | 0,264200002 | 0,245700002 | 0,250400007 | 0,232099995 | 0,232600003 |
| 80 | 0,250400007 0,248999998 | 0,275700003 | 0,280600011 | 0,268000007 | 0,270399988 | 0,248300001 | 0,252900004 | 0,231800005 | 0,230900005 |
| 100 | 0,250400007 0,249300003 | 0,285299987 | 0,290399998 | 0,276899993 | 0,277799994 | 0,253899992 | 0,258100003 | 0,231199995 | 0,233199999 |
| 120 | 0,247899994 0,247199997 | 0,292899996 | 0,300300002 | 0,282200009 | 0,28459999 | 0,256099999 | 0,257699996 | 0,230700001 | 0,231600001 |
| 140 | 0,251199991 0,249200001 | 0,304800004 | 0,308999985 | 0,290499985 | 0,292699993 | 0,260500014 | 0,262400001 | 0,231000006 | 0,231700003 |
| 160 | 0,249599993 0,248699993 | 0,311199993 | 0,317499995 | 0,296700001 | 0,299400002 | 0,262699991 | 0,264600009 | 0,2315 | 0,231800005 |
| 180 | 0,249799997 0,249200001 | 0,319599986 | 0,326799989 | 0,30399999 | 0,305799991 | 0,266400009 | 0,26730001 | 0,2315 | 0,231600001 |
| 200 | 0,249300003 0,251599997 | 0,326900005 | 0,335999995 | 0,308299989 | 0,3116 | 0,269499987 | 0,270099998 | 0,230199993 | 0,231099993 |
| 220 | 0,251599997 0,249699995 | 0,336699992 | 0,345999986 | 0,317299992 | 0,320800006 | 0,274599999 | 0,275099993 | 0,230800003 | 0,232600003 |
| 240 | 0,251899987 0,248999998 | 0,344700009 | 0,353700012 | 0,323399991 | 0,325800002 | 0,276899993 | 0,278600007 | 0,231399998 | 0,232999995 |
| 260 | 0,251100004 0,25150001 | 0,354099989 | 0,361900002 | 0,329299986 | 0,333000004 | 0,280099988 | 0,280200005 | 0,231700003 | 0,232299998 |
| 280 | 0,250499994 0,249300003 | 0,361200005 | 0,369500011 | 0,335500002 | 0,339899987 | 0,283100009 | 0,283399999 | 0,230700001 | 0,231299996 |
| 300 | 0,250800014 0,25060001 | 0,369300008 | 0,378199995 | 0,340999991 | 0,345400006 | 0,286799997 | 0,2861 | 0,229200006 | 0,230299994 |
| 320 | 0,251199991 0,249799997 | 0,378399998 | 0,386999995 | 0,346700013 | 0,352200001 | 0,290300012 | 0,289499998 | 0,230399996 | 0,231099993 |
| 340 | 0,251599997 0,249699995 | 0,386900008 | 0,395500004 | 0,352899998 | 0,359100014 | 0,293099999 | 0,292199999 | 0,230800003 | 0,231600001 |
| 360 | 0,251599997 0,249699995 | 0,395999998 | 0,403499991 | 0,359400004 | 0,366400003 | 0,297300011 | 0,296400011 | 0,230199993 | 0,233099997 |
| slope | 3,87512E-06 3,49844E-06 | 0,000418375 | 0,000441089 | 0,000327497 | 0,000340542 | 0,000169525 | 0,000151249 | -1,40351E-06 | 2,01224E-07 |
| slope w/o |  | 0,000414688 | 0,000437402 | 0,000323811 | 0,000336855 | 0,000165839 | 0,000147562 | -5,09029E-06 | -3,48555E-06 |

|  | **30 min** | 30 |
| --- | --- | --- |
| **PTPN22 WT control** | 27,2523395 | 76,05790021 |
| **PTPN22 WT control+bicobnate** | 21,13003251 | 4,469147798 |
| **PTPN22 WT 30uM H2O2** | 10,02346861 | 97,26724967 |
| **PTPN22 WT 30uM H2O2+bicobnate** | -0,27428071 | 31,22145889 |

#DIV/0!

| blank |  | 0,000426045 | 0,000330333 | 0,0001567 | -4,28792E-06 |
| --- | --- | --- | --- | --- | --- |
|  |  | 0,025562694 | 0,01981997 | 0,009402014 | -0,000257275 |
|  |  | 3,81532753 | 2,958204551 | 1,403285605 | -0,038399299 |
|  |  | 27,2523395 | 21,13003251 | 10,02346861 | -0,274280711 |
|  | **min -1** | 27,2523395 | 21,13003251 | 10,02346861 | -0,274280711 |

| Application: Tecan i-control  Device: infinite 200Pro | | Tecan i-control , 2.0.10.0  Serial number: 1307001123 | Serial number of connected stacker: |
| --- | --- | --- | --- |
| Firmware: V_3.40_01/15_Infinite (Dec 23 201 MAI, V_3.40_01/15_Infinite (Dec 23 2014/12.45.11) | | | |
| Date: | ######### | | |
| Time: | 13:25:05 | | |

System MTC-MU059-S

User MTC-MU059-S\fretho

Plate Greiner 96 Flat Bottom Transparent Polystyrene Cat. No.: 655101/655161/655192 [GRE96ft.pdfx] Plate-ID (Stacker)

List of actions in this measurement script: Kinetic

Absorbance

Shaking (Linear) Duration: Shaking (Linear) Amplitude:

3 s

1 mm

Label: Label1

Kinetic Measurement

Kinetic duration 00:06:00

Interval Time 00:00:20

Measurement Wavelength 405 nm

Bandwidth 10 nm

Number of Flashes 5

Settle Time 0 ms

Part of Plate H1-H10

Start Time: 2020 08-05 13:25:07

|  |  |  |  |  |  |  |  |  |  |  |  |  |  |  |  |  |  |  |  |
| --- | --- | --- | --- | --- | --- | --- | --- | --- | --- | --- | --- | --- | --- | --- | --- | --- | --- | --- | --- |
|  |  |  |  |  |  |  |  |  |  |  |  |  |  |  |  |  |  |  |  |
| Cycle Nr. | 1 | 2 | 3 | 4 | 5 | 6 | 7 | 8 | 9 | 10 | 11 | 12 | 13 | 14 | 15 | 16 | 17 | 18 | 19 |
| Time [s] | 0 | 20 | 40 | 60 | 80 | 100 | 120 | 140 | 160 | 180 | 200 | 220 | 240 | 260 | 280 | 300 | 320 | 340 | 360 |
| Temp. [°C] | 25,6 | 25,7 | 26 | 25,6 | 25,7 | 26 | 25,7 | 26,1 | 25,8 | 25,9 | 25,9 | 26 | 25,9 | 25,8 | 25,6 | 25,6 | 25,8 | 25,6 | 25,6 |
| H1 | 0,2495 | 0,2564 | 0,2611 | 0,2689 | 0,2757 | 0,2853 | 0,2929 | 0,3048 | 0,3112 | 0,3196 | 0,3269 | 0,3367 | 0,3447 | 0,3541 | 0,3612 | 0,3693 | 0,3784 | 0,3869 | 0,396 |
| H2 | 0,2495 | 0,2548 | 0,2628 | 0,272 | 0,2806 | 0,2904 | 0,3003 | 0,309 | 0,3175 | 0,3268 | 0,336 | 0,346 | 0,3537 | 0,3619 | 0,3695 | 0,3782 | 0,387 | 0,3955 | 0,4035 |
| H3 | 0,2631 | 0,2487 | 0,2546 | 0,2621 | 0,268 | 0,2769 | 0,2822 | 0,2905 | 0,2967 | 0,304 | 0,3083 | 0,3173 | 0,3234 | 0,3293 | 0,3355 | 0,341 | 0,3467 | 0,3529 | 0,3594 |
| H4 | 0,2463 | 0,2504 | 0,2574 | 0,2642 | 0,2704 | 0,2778 | 0,2846 | 0,2927 | 0,2994 | 0,3058 | 0,3116 | 0,3208 | 0,3258 | 0,333 | 0,3399 | 0,3454 | 0,3522 | 0,3591 | 0,3664 |
| H5 | 0,2374 | 0,2398 | 0,2429 | 0,2457 | 0,2483 | 0,2539 | 0,2561 | 0,2605 | 0,2627 | 0,2664 | 0,2695 | 0,2746 | 0,2769 | 0,2801 | 0,2831 | 0,2868 | 0,2903 | 0,2931 | 0,2973 |
| H6 | 0,2396 | 0,2453 | 0,2463 | 0,2504 | 0,2529 | 0,2581 | 0,2577 | 0,2624 | 0,2646 | 0,2673 | 0,2701 | 0,2751 | 0,2786 | 0,2802 | 0,2834 | 0,2861 | 0,2895 | 0,2922 | 0,2964 |
| H7 | 0,2296 | 0,2295 | 0,2301 | 0,2321 | 0,2318 | 0,2312 | 0,2307 | 0,231 | 0,2315 | 0,2315 | 0,2302 | 0,2308 | 0,2314 | 0,2317 | 0,2307 | 0,2292 | 0,2304 | 0,2308 | 0,2302 |
| H8 | 0,2312 | 0,2312 | 0,2315 | 0,2326 | 0,2309 | 0,2332 | 0,2316 | 0,2317 | 0,2318 | 0,2316 | 0,2311 | 0,2326 | 0,233 | 0,2323 | 0,2313 | 0,2303 | 0,2311 | 0,2316 | 0,2331 |
| H9 | 0,2504 | 0,2511 | 0,2503 | 0,2504 | 0,2504 | 0,2504 | 0,2479 | 0,2512 | 0,2496 | 0,2498 | 0,2493 | 0,2516 | 0,2519 | 0,2511 | 0,2505 | 0,2508 | 0,2512 | 0,2516 | 0,2516 |
| H10 | 0,2465 | 0,2486 | 0,2486 | 0,2513 | 0,249 | 0,2493 | 0,2472 | 0,2492 | 0,2487 | 0,2492 | 0,2516 | 0,2497 | 0,249 | 0,2515 | 0,2493 | 0,2506 | 0,2498 | 0,2497 | 0,2497 |

End Time: 2020 08-05 13:31:18

| Application: Tecan i-control  Device: infinite 200Pro | | Tecan i-control , 2.0.10.0  Serial number: 1307001123 | Serial number of connected stacker: |
| --- | --- | --- | --- |
| Firmware: V_3.40_01/15_Infinite (Dec 23 201 MAI, V_3.40_01/15_Infinite (Dec 23 2014/12.45.11) | | | |
| Date: | ######### | | |
| Time: | 13:35:58 | | |

System MTC-MU059-S

User MTC-MU059-S\fretho

Plate Greiner 96 Flat Bottom Transparent Polystyrene Cat. No.: 655101/655161/655192 [GRE96ft.pdfx] Plate-ID (Stacker)

List of actions in this measurement script: Kinetic

Absorbance

Shaking (Linear) Duration: Shaking (Linear) Amplitude:

3 s

1 mm

Label: Label1

Kinetic Measurement

Kinetic duration 00:06:00

Interval Time 00:00:20

Measurement Wavelength 405 nm

Bandwidth 10 nm

Number of Flashes 5

Settle Time 0 ms

Part of Plate B1-B10

Start Time: 2020 08-05 13:36:00

|  |  |  |  |  |  |  |  |  |  |  |  |  |  |  |  |  |  |  |  |
| --- | --- | --- | --- | --- | --- | --- | --- | --- | --- | --- | --- | --- | --- | --- | --- | --- | --- | --- | --- |
|  |  |  |  |  |  |  |  |  |  |  |  |  |  |  |  |  |  |  |  |
| Cycle Nr. | 1 | 2 | 3 | 4 | 5 | 6 | 7 | 8 | 9 | 10 | 11 | 12 | 13 | 14 | 15 | 16 | 17 | 18 | 19 |
| Time [s] | 0 | 20 | 40 | 60 | 80 | 100 | 120 | 140 | 160 | 180 | 200 | 220 | 240 | 260 | 280 | 300 | 320 | 340 | 360 |
| Temp. [°C] | 25,6 | 25,7 | 25,7 | 25,8 | 25,8 | 25,7 | 25,8 | 26 | 25,8 | 25,8 | 25,8 | 25,8 | 25,9 | 25,6 | 25,8 | 26 | 25,6 | 26 | 25,6 |
| B1 | 0,2557 | 0,2591 | 0,2643 | 0,2717 | 0,2811 | 0,2888 | 0,2975 | 0,3052 | 0,3133 | 0,3215 | 0,3282 | 0,3361 | 0,3444 | 0,3514 | 0,3598 | 0,368 | 0,3756 | 0,3825 | 0,3916 |
| B2 | 0,2557 | 0,2614 | 0,268 | 0,2766 | 0,2882 | 0,2981 | 0,3056 | 0,3132 | 0,3215 | 0,3311 | 0,3401 | 0,3468 | 0,3551 | 0,3629 | 0,3707 | 0,3792 | 0,387 | 0,3949 | 0,4038 |
| B3 | 0,2562 | 0,2549 | 0,256 | 0,258 | 0,2579 | 0,2632 | 0,2661 | 0,2656 | 0,2678 | 0,2721 | 0,2745 | 0,2763 | 0,2795 | 0,2828 | 0,285 | 0,2867 | 0,2909 | 0,2924 | 0,2962 |
| B4 | 0,252 | 0,2551 | 0,2587 | 0,2639 | 0,2691 | 0,2756 | 0,2827 | 0,2875 | 0,2919 | 0,297 | 0,302 | 0,3066 | 0,3106 | 0,3165 | 0,3195 | 0,3237 | 0,3293 | 0,3333 | 0,34 |
| B5 | 0,2543 | 0,2581 | 0,2669 | 0,2756 | 0,2838 | 0,2921 | 0,3021 | 0,3085 | 0,316 | 0,3237 | 0,3299 | 0,3383 | 0,347 | 0,3545 | 0,3619 | 0,3703 | 0,3784 | 0,3864 | 0,3947 |
| B6 | 0,2526 | 0,2553 | 0,2625 | 0,2699 | 0,2772 | 0,2866 | 0,2928 | 0,2997 | 0,3059 | 0,3144 | 0,3208 | 0,3268 | 0,3341 | 0,3404 | 0,3457 | 0,3525 | 0,3589 | 0,3656 | 0,373 |
| B7 | 0,2495 | 0,2455 | 0,2456 | 0,2435 | 0,2412 | 0,242 | 0,2402 | 0,241 | 0,2406 | 0,2414 | 0,2407 | 0,2408 | 0,2418 | 0,2415 | 0,2413 | 0,2413 | 0,2434 | 0,2432 | 0,2436 |
| B8 | 0,2464 | 0,2427 | 0,2429 | 0,2395 | 0,2391 | 0,2406 | 0,24 | 0,2421 | 0,2405 | 0,2448 | 0,243 | 0,2424 | 0,243 | 0,2426 | 0,2438 | 0,2453 | 0,2449 | 0,2448 | 0,2461 |
| B9 | 0,2535 | 0,2545 | 0,253 | 0,2541 | 0,2517 | 0,2513 | 0,2515 | 0,252 | 0,2513 | 0,2523 | 0,2515 | 0,2533 | 0,2519 | 0,2516 | 0,2515 | 0,2515 | 0,2529 | 0,2512 | 0,2528 |
| B10 | 0,2552 | 0,2543 | 0,255 | 0,2542 | 0,253 | 0,2543 | 0,2534 | 0,2549 | 0,2538 | 0,2543 | 0,2536 | 0,2534 | 0,2552 | 0,2535 | 0,2529 | 0,2541 | 0,2543 | 0,2539 | 0,2541 |

End Time: 2020 08-05 13:42:10

**PTPN22 WT control PTPN22 WT control PTPN22 WT control+bicobnaPTPN22 WT control+bicobna PTPN22 WT 30uM H2O2 PTPN22 WT 30uM H2O2 PTPN22 WT 30uM H2O2+bicobnate PTPN22 WT 30uM H2O2+bicobnate**

| blank |  | 0,000408003 | 0,000188282 | 0,000371192 | 7,83283E-06 |
| --- | --- | --- | --- | --- | --- |
|  |  | 0,024480187 | 0,011296904 | 0,022271518 | 0,00046997 |
|  |  | 3,653759251 | 1,686105045 | 3,32410713 | 0,070144739 |
|  |  | 26,09828036 | 12,04360746 | 23,74362235 | 0,501033852 |
|  | **min -1** | 26,09828036 | 12,04360746 | 23,74362235 | 0,501033852 |

| **Time (sec) Blank** |  | **Blank** |  | | | | | | | |
| --- | --- | --- | --- | --- | --- | --- | --- | --- | --- | --- |
| 20 | 0,254500002 | 0,254299998 | 0,25909999 | 0,261400014 | 0,254900008 | 0,255100012 | 0,258100003 | 0,255299985 | 0,245499998 | 0,242699996 |
| 40 | 0,252999991 | 0,254999995 | 0,264299989 | 0,268000007 | 0,256000012 | 0,258700013 | 0,266900003 | 0,262499988 | 0,2456 | 0,242899999 |
| 60 | 0,254099995 | 0,254200011 | 0,271699995 | 0,276600003 | 0,257999986 | 0,263900012 | 0,275599986 | 0,269899994 | 0,243499994 | 0,239500001 |
| 80 | 0,251700014 | 0,252999991 | 0,281100005 | 0,288199991 | 0,2579 | 0,26910001 | 0,283800006 | 0,277200013 | 0,2412 | 0,239099994 |
| 100 | 0,251300007 | 0,254299998 | 0,288800001 | 0,298099995 | 0,263200015 | 0,275599986 | 0,292100012 | 0,286599994 | 0,241999999 | 0,240600005 |
| 120 | 0,25150001 | 0,253399998 | 0,297500014 | 0,305599988 | 0,266099989 | 0,282700002 | 0,302100003 | 0,292800009 | 0,240199998 | 0,239999995 |
| 140 | 0,252000004 | 0,254900008 | 0,305200011 | 0,313199997 | 0,265599996 | 0,287499994 | 0,308499992 | 0,299699992 | 0,240999997 | 0,2421 |
| 160 | 0,251300007 | 0,253800005 | 0,313300014 | 0,321500003 | 0,267800003 | 0,291900009 | 0,316000015 | 0,305900007 | 0,240600005 | 0,240500003 |
| 180 | 0,252299994 | 0,254299998 | 0,321500003 | 0,331099987 | 0,272100002 | 0,296999991 | 0,323700011 | 0,314399987 | 0,241400003 | 0,244800001 |
| 200 | 0,25150001 | 0,253600001 | 0,328200012 | 0,34009999 | 0,274500012 | 0,301999986 | 0,329899997 | 0,320800006 | 0,240700006 | 0,243000001 |
| 220 | 0,253300011 | 0,253399998 | 0,336100012 | 0,346799999 | 0,276300013 | 0,306600004 | 0,33829999 | 0,326799989 | 0,240799993 | 0,242400005 |
| 240 | 0,251899987 | 0,255199999 | 0,344399989 | 0,355100006 | 0,279500008 | 0,310600013 | 0,347000003 | 0,334100008 | 0,241799995 | 0,243000001 |
| 260 | 0,251599997 | 0,253500015 | 0,351399988 | 0,362899989 | 0,282799989 | 0,316500008 | 0,354499996 | 0,34040001 | 0,241500005 | 0,242599994 |
| 280 | 0,25150001 | 0,252900004 | 0,359800011 | 0,370700002 | 0,284999996 | 0,319499999 | 0,361900002 | 0,345699996 | 0,241300002 | 0,243799999 |
| 300 | 0,25150001 | 0,254099995 | 0,368000001 | 0,379200011 | 0,28670001 | 0,323700011 | 0,370299995 | 0,352499992 | 0,241300002 | 0,245299995 |
| 320 | 0,252900004 | 0,254299998 | 0,37560001 | 0,386999995 | 0,290899992 | 0,329299986 | 0,378399998 | 0,358900011 | 0,243399993 | 0,244900003 |
| 340 | 0,251199991 | 0,253899992 | 0,382499993 | 0,394899994 | 0,292400002 | 0,333299994 | 0,386400014 | 0,36559999 | 0,243200004 | 0,244800001 |
| 360 | 0,252799988 | 0,254099995 | 0,391600013 | 0,403800011 | 0,296200007 | 0,340000004 | 0,394699991 | 0,372999996 | 0,243599996 | 0,246099994 |
| slope | -3,43139E-06 | -9,70079E-07 | 0,000393163 | 0,000418442 | 0,000123999 | 0,000248163 | 0,000395418 | 0,000342565 | -3,18885E-06 | 1,4453E-05 |
| slope w/o |  |  | 0,000395364 | 0,000420642 | 0,0001262 | 0,000250364 | 0,000397619 | 0,000344765 | -9,88118E-07 | 1,66538E-05 |

|  | **5 min** | 30 |
| --- | --- | --- |
| **PTPN22 WT control** | 26,09828036 | 76,05790021 |
| **PTPN22 WT control+bicobnate** | 12,04360746 | 4,469147798 |
| **PTPN22 WT 30uM H2O2** | 23,74362235 | 97,26724967 |
| **PTPN22 WT 30uM H2O2+bicobnate** | 0,501033852 | 31,22145889 |

|  | | | **PTPN22 WT control** | **PTPN22 WT control** | **PTPN22 WT control+bicobnate** | **PTPN22 WT control+bicobnate** | **PTPN22 WT 30uM H2O2** | **PTPN22 WT 30uM H2O2** | **PTPN22 WT 30uM H2O2+bicobnate PTPN22 WT 30uM** | **H2O2+bicobnate** |
| --- | --- | --- | --- | --- | --- | --- | --- | --- | --- | --- |
| **Time (sec) Blank** |  | **Blank** |  |  |  |  |  |  |  |  |
| 20 | 0,254400015 | 0,256900012 | 0,257699996 | 0,255299985 | 0,252999991 | 0,256099999 | 0,249400005 | 0,246000007 | 0,238700002 | 0,241400003 |
| 40 | 0,254599988 | 0,257400006 | 0,266900003 | 0,266200006 | 0,261900008 | 0,264800012 | 0,254700005 | 0,251700014 | 0,239600003 | 0,243000001 |
| 60 | 0,253899992 | 0,257099986 | 0,275400013 | 0,273299992 | 0,269499987 | 0,270799994 | 0,257299989 | 0,253300011 | 0,241300002 | 0,245199993 |
| 80 | 0,254400015 | 0,257400006 | 0,283800006 | 0,279799998 | 0,273200005 | 0,279500008 | 0,260899991 | 0,257400006 | 0,240999997 | 0,244900003 |
| 100 | 0,255199999 | 0,256900012 | 0,293099999 | 0,288899988 | 0,279399991 | 0,28639999 | 0,265199989 | 0,261200011 | 0,241300002 | 0,243900001 |
| 120 | 0,254700005 | 0,257800013 | 0,30250001 | 0,296700001 | 0,286199987 | 0,29370001 | 0,270399988 | 0,264999986 | 0,241899997 | 0,244200006 |
| 140 | 0,253199995 | 0,256999999 | 0,311699986 | 0,309399992 | 0,290800005 | 0,298599988 | 0,273499995 | 0,268000007 | 0,240799993 | 0,243100002 |
| 160 | 0,253600001 | 0,259000003 | 0,32069999 | 0,317299992 | 0,298000008 | 0,306100011 | 0,277500004 | 0,272799999 | 0,241600007 | 0,241999999 |
| 180 | 0,255199999 | 0,2588 | 0,331800014 | 0,321500003 | 0,304399997 | 0,312999994 | 0,282099992 | 0,277799994 | 0,2421 | 0,241699994 |
| 200 | 0,255299985 | 0,258599997 | 0,340999991 | 0,329100013 | 0,3116 | 0,32159999 | 0,287800014 | 0,282000005 | 0,241899997 | 0,243300006 |
| 220 | 0,256000012 | 0,258399993 | 0,350600004 | 0,337300003 | 0,317799985 | 0,329400003 | 0,290499985 | 0,287200004 | 0,242500007 | 0,244399995 |
| 240 | 0,254200011 | 0,258899987 | 0,358700007 | 0,343899995 | 0,324800014 | 0,337099999 | 0,29519999 | 0,291799992 | 0,241400003 | 0,243499994 |
| 260 | 0,253699988 | 0,25819999 | 0,36680001 | 0,350699991 | 0,329699993 | 0,342900008 | 0,297899991 | 0,294699997 | 0,240799993 | 0,241300002 |
| 280 | 0,254599988 | 0,260199994 | 0,377499998 | 0,359499991 | 0,337500006 | 0,351000011 | 0,30340001 | 0,298999995 | 0,242200002 | 0,243399993 |
| 300 | 0,254999995 | 0,25940001 | 0,389099985 | 0,368000001 | 0,344799995 | 0,359200001 | 0,307300001 | 0,302899987 | 0,242200002 | 0,243900001 |
| 320 | 0,254700005 | 0,25850001 | 0,395300001 | 0,374799997 | 0,349099994 | 0,365399987 | 0,310799986 | 0,306800008 | 0,241799995 | 0,242599994 |
| 340 | 0,254200011 | 0,259799987 | 0,404900014 | 0,383300006 | 0,35679999 | 0,372200012 | 0,317200005 | 0,31099999 | 0,242400005 | 0,244299993 |
| 360 | 0,256000012 | 0,259600013 | 0,414700001 | 0,391000003 | 0,363400012 | 0,379700005 | 0,320499986 | 0,315299988 | 0,243399993 | 0,2447 |
| slope | 1,96595E-06 | 8,36428E-06 | 0,000464376 | 0,000392497 | 0,000319231 | 0,000361997 | 0,000208545 | 0,000205268 | 7,61093E-06 | 8,66855E-07 |
| slope w/o |  |  | 0,000459211 | 0,000387332 | 0,000314066 | 0,000356832 | 0,00020338 | 0,000200103 | 2,44582E-06 | -4,29826E-06 |

| blank |  | 0,000423271 | 0,000335449 | 0,000201741 | -9,2622E-07 |
| --- | --- | --- | --- | --- | --- |
|  |  | 0,025396286 | 0,020126936 | 0,012104488 | -5,55732E-05 |
|  |  | 3,790490408 | 3,004020272 | 1,806639953 | -0,008294506 |
|  |  | 27,07493149 | 21,45728766 | 12,90457109 | -0,05924647 |
|  | **min -1** | 27,07493149 | 21,45728766 | 12,90457109 | -0,05924647 |

|  | **15 min** | 30 |
| --- | --- | --- |
| **PTPN22 WT control** | 27,07493149 | 76,05790021 |
| **PTPN22 WT control+bicobnate** | 21,45728766 | 4,469147798 |
| **PTPN22 WT 30uM H2O2** | 12,90457109 | 97,26724967 |
| **PTPN22 WT 30uM H2O2+bicobnate** | -0,05924647 | 31,22145889 |

| Application: Tecan i-control  Device: infinite 200Pro | | Tecan i-control , 2.0.10.0  Serial number: 1307001123 | Serial number of connected stacker: |
| --- | --- | --- | --- |
| Firmware: V_3.40_01/15_Infinite (Dec 23 201 MAI, V_3.40_01/15_Infinite (Dec 23 2014/12.45.11) | | | |
| Date: | ######### | | |
| Time: | 13:46:55 | | |

System MTC-MU059-S

User MTC-MU059-S\fretho

Plate Greiner 96 Flat Bottom Transparent Polystyrene Cat. No.: 655101/655161/655192 [GRE96ft.pdfx] Plate-ID (Stacker)

List of actions in this measurement script: Kinetic

Absorbance

Shaking (Linear) Duration: Shaking (Linear) Amplitude:

3 s

1 mm

Label: Label1

Kinetic Measurement

Kinetic duration 00:06:00

Interval Time 00:00:20

Measurement Wavelength 405 nm

Bandwidth 10 nm

Number of Flashes 5

Settle Time 0 ms

Part of Plate C1-C10

Start Time: 2020 08-05 13:46:57

|  |  |  |  |  |  |  |  |  |  |  |  |  |  |  |  |  |  |  |  |
| --- | --- | --- | --- | --- | --- | --- | --- | --- | --- | --- | --- | --- | --- | --- | --- | --- | --- | --- | --- |
|  |  |  |  |  |  |  |  |  |  |  |  |  |  |  |  |  |  |  |  |
| Cycle Nr. | 1 | 2 | 3 | 4 | 5 | 6 | 7 | 8 | 9 | 10 | 11 | 12 | 13 | 14 | 15 | 16 | 17 | 18 | 19 |
| Time [s] | 0 | 20 | 40 | 60 | 80 | 100 | 120 | 140 | 160 | 180 | 200 | 220 | 240 | 260 | 280 | 300 | 320 | 340 | 360 |
| Temp. [°C] | 26 | 25,8 | 26,1 | 25,9 | 26,1 | 26 | 26 | 26 | 26,3 | 26 | 26,1 | 26,1 | 25,9 | 25,9 | 26 | 26 | 26 | 26,1 | 26 |
| C1 | 0,2528 | 0,2577 | 0,2669 | 0,2754 | 0,2838 | 0,2931 | 0,3025 | 0,3117 | 0,3207 | 0,3318 | 0,341 | 0,3506 | 0,3587 | 0,3668 | 0,3775 | 0,3891 | 0,3953 | 0,4049 | 0,4147 |
| C2 | 0,2511 | 0,2553 | 0,2662 | 0,2733 | 0,2798 | 0,2889 | 0,2967 | 0,3094 | 0,3173 | 0,3215 | 0,3291 | 0,3373 | 0,3439 | 0,3507 | 0,3595 | 0,368 | 0,3748 | 0,3833 | 0,391 |
| C3 | 0,2516 | 0,253 | 0,2619 | 0,2695 | 0,2732 | 0,2794 | 0,2862 | 0,2908 | 0,298 | 0,3044 | 0,3116 | 0,3178 | 0,3248 | 0,3297 | 0,3375 | 0,3448 | 0,3491 | 0,3568 | 0,3634 |
| C4 | 0,2515 | 0,2561 | 0,2648 | 0,2708 | 0,2795 | 0,2864 | 0,2937 | 0,2986 | 0,3061 | 0,313 | 0,3216 | 0,3294 | 0,3371 | 0,3429 | 0,351 | 0,3592 | 0,3654 | 0,3722 | 0,3797 |
| C5 | 0,247 | 0,2494 | 0,2547 | 0,2573 | 0,2609 | 0,2652 | 0,2704 | 0,2735 | 0,2775 | 0,2821 | 0,2878 | 0,2905 | 0,2952 | 0,2979 | 0,3034 | 0,3073 | 0,3108 | 0,3172 | 0,3205 |
| C6 | 0,2462 | 0,246 | 0,2517 | 0,2533 | 0,2574 | 0,2612 | 0,265 | 0,268 | 0,2728 | 0,2778 | 0,282 | 0,2872 | 0,2918 | 0,2947 | 0,299 | 0,3029 | 0,3068 | 0,311 | 0,3153 |
| C7 | 0,2414 | 0,2387 | 0,2396 | 0,2413 | 0,241 | 0,2413 | 0,2419 | 0,2408 | 0,2416 | 0,2421 | 0,2419 | 0,2425 | 0,2414 | 0,2408 | 0,2422 | 0,2422 | 0,2418 | 0,2424 | 0,2434 |
| C8 | 0,2408 | 0,2414 | 0,243 | 0,2452 | 0,2449 | 0,2439 | 0,2442 | 0,2431 | 0,242 | 0,2417 | 0,2433 | 0,2444 | 0,2435 | 0,2413 | 0,2434 | 0,2439 | 0,2426 | 0,2443 | 0,2447 |
| C9 | 0,2547 | 0,2544 | 0,2546 | 0,2539 | 0,2544 | 0,2552 | 0,2547 | 0,2532 | 0,2536 | 0,2552 | 0,2553 | 0,256 | 0,2542 | 0,2537 | 0,2546 | 0,255 | 0,2547 | 0,2542 | 0,256 |
| C10 | 0,2554 | 0,2569 | 0,2574 | 0,2571 | 0,2574 | 0,2569 | 0,2578 | 0,257 | 0,259 | 0,2588 | 0,2586 | 0,2584 | 0,2589 | 0,2582 | 0,2602 | 0,2594 | 0,2585 | 0,2598 | 0,2596 |

End Time: 2020 08-05 13:53:07

**PTPN22 WT control PTPN22 WT control PTPN22 WT control+bicobnate PTPN22 WT control+bicobnate PTPN22 WT 30uM H2O2 PTPN22 WT 30uM H2O2 PTPN22 WT 30uM H2O2+bicobnate PTPN22 WT 30uM H2O2+bicobnate**

| blank |  | 0,000317237 | 0,000220312 | 0,000146244 | -3,94481E-06 |
| --- | --- | --- | --- | --- | --- |
|  |  | 0,019034207 | 0,013218732 | 0,008774612 | -0,000236689 |
|  |  | 2,840926426 | 1,972945072 | 1,309643585 | -0,035326655 |
|  |  | 20,29233161 | 14,0924648 | 9,354597034 | -0,252333253 |
|  | **min -1** | 20,29233161 | 14,0924648 | 9,354597034 | -0,252333253 |

**Time (sec) Blank Blank**

|  | 20 | 0,252900004 | 0,258100003 | 0,637399971 | 0,583100021 | 0,50999999 | 0,541700006 | 0,42719999 | 0,4111 | 0,242400005 | 0,246600002 |
| --- | --- | --- | --- | --- | --- | --- | --- | --- | --- | --- | --- |
|  | 40 | 0,252799988 | 0,258300006 | 0,644699991 | 0,587100029 | 0,515799999 | 0,546299994 | 0,428600013 | 0,414999992 | 0,242400005 | 0,245199993 |
|  | 60 | 0,253199995 | 0,258300006 | 0,652700007 | 0,593800008 | 0,519500017 | 0,552699983 | 0,431400001 | 0,416700006 | 0,242300004 | 0,244499996 |
|  | 80 | 0,252799988 | 0,259900004 | 0,659200013 | 0,599799991 | 0,525200009 | 0,558099985 | 0,435400009 | 0,420899987 | 0,242699996 | 0,2447 |
|  | 100 | 0,252499998 | 0,257999986 | 0,665600002 | 0,605199993 | 0,528400004 | 0,561699986 | 0,437700003 | 0,423500001 | 0,243900001 | 0,245000005 |
|  | 120 | 0,252900004 | 0,258300006 | 0,673099995 | 0,610800028 | 0,533100009 | 0,566799998 | 0,442299992 | 0,425300002 | 0,2421 | 0,245000005 |
|  | 140 | 0,252900004 | 0,259499997 | 0,680000007 | 0,616900027 | 0,536599994 | 0,570900023 | 0,443300009 | 0,428600013 | 0,241400003 | 0,2447 |
|  | 160 | 0,252200007 | 0,257499993 | 0,68629998 | 0,622200012 | 0,541899979 | 0,573700011 | 0,4463 | 0,430900007 | 0,241099998 | 0,244900003 |
|  | 180 | 0,252499998 | 0,25819999 | 0,694199979 | 0,628000021 | 0,545700014 | 0,578299999 | 0,450700015 | 0,433899999 | 0,241699994 | 0,245000005 |
|  | 200 | 0,252000004 | 0,257499993 | 0,699999988 | 0,631699979 | 0,547999978 | 0,580900013 | 0,452199996 | 0,435200006 | 0,2403 | 0,242899999 |
|  | 220 | 0,252799988 | 0,259000003 | 0,709599972 | 0,641900003 | 0,554400027 | 0,58859998 | 0,457399994 | 0,440600008 | 0,241999999 | 0,244800001 |
|  | 240 | 0,251800001 | 0,257400006 | 0,714600027 | 0,6426 | 0,555499971 | 0,589600027 | 0,458499998 | 0,439599991 | 0,240799993 | 0,242599994 |
|  | 260 | 0,251700014 | 0,257400006 | 0,721499979 | 0,650399983 | 0,561600029 | 0,595499992 | 0,462900013 | 0,444099993 | 0,241300002 | 0,242899999 |
|  | 280 | 0,252799988 | 0,257800013 | 0,729300022 | 0,657000005 | 0,567799985 | 0,602999985 | 0,467400014 | 0,450199991 | 0,242699996 | 0,244499996 |
|  | 300 | 0,252299994 | 0,258399993 | 0,735199988 | 0,66049999 | 0,571399987 | 0,604900002 | 0,468199998 | 0,449900001 | 0,240999997 | 0,243599996 |
|  | 320 | 0,253399998 | 0,25850001 | 0,744700015 | 0,666499972 | 0,575399995 | 0,610000014 | 0,472299993 | 0,453399986 | 0,241899997 | 0,243599996 |
|  | 340 | 0,250699997 | 0,257099986 | 0,748399973 | 0,669700027 | 0,578100026 | 0,614400029 | 0,47240001 | 0,4551 | 0,240199998 | 0,242200002 |
|  | 360 | 0,251700014 | 0,258700013 | 0,757399976 | 0,67839998 | 0,583800018 | 0,620000005 | 0,476599991 | 0,458200008 | 0,241600007 | 0,242799997 |
| slope |  | -3,33848E-06 | -1,88338E-06 | 0,000350273 | 0,000278978 | 0,000212198 | 0,000223204 | 0,000150237 | 0,000137028 | -4,57174E-06 | -8,53974E-06 |
| slope w/o | | 0,000352884 | | | 0,000281589 | 0,000214809 | 0,000225815 | 0,000152848 | 0,000139639 | -1,96081E-06 | -5,92881E-06 |

|  | **30 min** | 30 |
| --- | --- | --- |
| **PTPN22 WT control** | 20,29233161 | 76,05790021 |
| **PTPN22 WT control+bicobnate** | 14,0924648 | 4,469147798 |
| **PTPN22 WT 30uM H2O2** | 9,354597034 | 97,26724967 |
| **PTPN22 WT 30uM H2O2+bicobnate** | -0,252333253 | 31,22145889 |

| Application: Tecan i-control  Device: infinite 200Pro | | Tecan i-control , 2.0.10.0  Serial number: 1307001123 | Serial number of connected stacker: |
| --- | --- | --- | --- |
| Firmware: V_3.40_01/15_Infinite (Dec 23 201 MAI, V_3.40_01/15_Infinite (Dec 23 2014/12.45.11) | | | |
| Date: | ######### | | |
| Time: | 14:01:34 | | |

System MTC-MU059-S

User MTC-MU059-S\fretho

Plate Greiner 96 Flat Bottom Transparent Polystyrene Cat. No.: 655101/655161/655192 [GRE96ft.pdfx] Plate-ID (Stacker)

List of actions in this measurement script: Kinetic

Absorbance

Shaking (Linear) Duration: Shaking (Linear) Amplitude:

3 s

1 mm

Label: Label1

Kinetic Measurement

Kinetic duration 00:06:00

Interval Time 00:00:20

Measurement Wavelength 405 nm

Bandwidth 10 nm

Number of Flashes 5

Settle Time 0 ms

Part of Plate C1-C10

Start Time: 2020 08-05 14:01:37

|  |  |  |  |  |  |  |  |  |  |  |  |  |  |  |  |  |  |  |  |
| --- | --- | --- | --- | --- | --- | --- | --- | --- | --- | --- | --- | --- | --- | --- | --- | --- | --- | --- | --- |
|  |  |  |  |  |  |  |  |  |  |  |  |  |  |  |  |  |  |  |  |
| Cycle Nr. | 1 | 2 | 3 | 4 | 5 | 6 | 7 | 8 | 9 | 10 | 11 | 12 | 13 | 14 | 15 | 16 | 17 | 18 | 19 |
| Time [s] | 0 | 20 | 40 | 60 | 80 | 100 | 120 | 140 | 160 | 180 | 200 | 220 | 240 | 260 | 280 | 300 | 320 | 340 | 360 |
| Temp. [°C] | 25,6 | 25,8 | 25,9 | 25,6 | 25,7 | 25,6 | 25,7 | 25,7 | 25,7 | 25,6 | 25,6 | 25,6 | 25,7 | 25,8 | 25,8 | 25,7 | 26 | 26,1 | 25,7 |
| C1 | 0,6291 | 0,6374 | 0,6447 | 0,6527 | 0,6592 | 0,6656 | 0,6731 | 0,68 | 0,6863 | 0,6942 | 0,7 | 0,7096 | 0,7146 | 0,7215 | 0,7293 | 0,7352 | 0,7447 | 0,7484 | 0,7574 |
| C2 | 0,5747 | 0,5831 | 0,5871 | 0,5938 | 0,5998 | 0,6052 | 0,6108 | 0,6169 | 0,6222 | 0,628 | 0,6317 | 0,6419 | 0,6426 | 0,6504 | 0,657 | 0,6605 | 0,6665 | 0,6697 | 0,6784 |
| C3 | 0,5053 | 0,51 | 0,5158 | 0,5195 | 0,5252 | 0,5284 | 0,5331 | 0,5366 | 0,5419 | 0,5457 | 0,548 | 0,5544 | 0,5555 | 0,5616 | 0,5678 | 0,5714 | 0,5754 | 0,5781 | 0,5838 |
| C4 | 0,5354 | 0,5417 | 0,5463 | 0,5527 | 0,5581 | 0,5617 | 0,5668 | 0,5709 | 0,5737 | 0,5783 | 0,5809 | 0,5886 | 0,5896 | 0,5955 | 0,603 | 0,6049 | 0,61 | 0,6144 | 0,62 |
| C5 | 0,4212 | 0,4272 | 0,4286 | 0,4314 | 0,4354 | 0,4377 | 0,4423 | 0,4433 | 0,4463 | 0,4507 | 0,4522 | 0,4574 | 0,4585 | 0,4629 | 0,4674 | 0,4682 | 0,4723 | 0,4724 | 0,4766 |
| C6 | 0,4071 | 0,4111 | 0,415 | 0,4167 | 0,4209 | 0,4235 | 0,4253 | 0,4286 | 0,4309 | 0,4339 | 0,4352 | 0,4406 | 0,4396 | 0,4441 | 0,4502 | 0,4499 | 0,4534 | 0,4551 | 0,4582 |
| C7 | 0,2424 | 0,2424 | 0,2424 | 0,2423 | 0,2427 | 0,2439 | 0,2421 | 0,2414 | 0,2411 | 0,2417 | 0,2403 | 0,242 | 0,2408 | 0,2413 | 0,2427 | 0,241 | 0,2419 | 0,2402 | 0,2416 |
| C8 | 0,2452 | 0,2466 | 0,2452 | 0,2445 | 0,2447 | 0,245 | 0,245 | 0,2447 | 0,2449 | 0,245 | 0,2429 | 0,2448 | 0,2426 | 0,2429 | 0,2445 | 0,2436 | 0,2436 | 0,2422 | 0,2428 |
| C9 | 0,2519 | 0,2529 | 0,2528 | 0,2532 | 0,2528 | 0,2525 | 0,2529 | 0,2529 | 0,2522 | 0,2525 | 0,252 | 0,2528 | 0,2518 | 0,2517 | 0,2528 | 0,2523 | 0,2534 | 0,2507 | 0,2517 |
| C10 | 0,2587 | 0,2581 | 0,2583 | 0,2583 | 0,2599 | 0,258 | 0,2583 | 0,2595 | 0,2575 | 0,2582 | 0,2575 | 0,259 | 0,2574 | 0,2574 | 0,2578 | 0,2584 | 0,2585 | 0,2571 | 0,2587 |

End Time: 2020 08-05 14:07:46
