# Supplementary material for: Redox regulation of PTPN22 affects the severity of T-cell-dependent autoimmune inflammation
Source: eLife. 2022 May 19;11:e74549. doi: 10.7554/eLife.74549 (PMC9119677; doi:10.7554/eLife.74549)
Supplement: Figure 1—source data 4. [file elife-74549-fig1-data4.docx]

Activity of WT and C129S mutant PTPN22 added to 0,25,50µM H_2_O_2_ with 1mM NaN_3_ was measured at 5,15,30 minutes

**150**


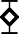

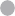

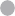

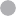

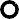

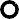

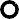


**PTPN22 activity% of control**

**100**

**50**

PTPN22 wt control PTPN22 mut control PTPN22 wt 25uM H2O2 PTPN22 mut 25uM H2O2 PTPN22 wt 50uM H2O2 PTPN22 mut 50uM H2O2

**0**

**0 10 20 30 40**

**min**

**170330**

**Time (sec) Blank Blank**

**PTPN22 wt control PTPN22 wt control PTPN22 mut control PTPN22 mut control PTPN22 wt 25uM H2O2 PTPN22 wt 25uM H2O2 PTPN22 mut 25uM H2O2 PTPN22 mut 25uM H2O2 PTPN22 wt 50uM H2O PTPN22 wt 50uM H2O PTPN22 mut 50uM H2O2 PTPN22 mut 50uM H2O2**

| 20 | 0,253399998 | 0,255100012 | 0,251599997 | | 0,259200007 | 0,251399994 | 0,25150001 | 0,250699997 | 0,257299989 | 0,247400001 | 0,247799993 | 0,255299985 | 0,250800014 | 0,245399997 | 0,246600002 |
| --- | --- | --- | --- | --- | --- | --- | --- | --- | --- | --- | --- | --- | --- | --- | --- |
| 40 | 0,254599988 | 0,255899996 | 0,256799996 | | 0,263500005 | 0,254000008 | 0,252799988 | 0,256099999 | 0,25850001 | 0,251300007 | 0,249899998 | 0,260500014 | 0,254799992 | 0,247999996 | 0,247400001 |
| 60 | 0,254700005 | 0,254700005 | 0,259799987 | | 0,267699987 | 0,256900012 | 0,254000008 | 0,259299994 | 0,263500005 | 0,252099991 | 0,251700014 | 0,265500009 | 0,257499993 | 0,249699995 | 0,249300003 |
| 80 | 0,254900008 | 0,255699992 | 0,265100002 | | 0,276199996 | 0,260399997 | 0,257600009 | 0,264400005 | 0,268900007 | 0,255400002 | 0,254799992 | 0,272399992 | 0,261999995 | 0,252000004 | 0,251899987 |
| 100 | 0,253500015 | 0,254700005 | 0,268700004 | | 0,279599994 | 0,261799991 | 0,259000003 | 0,26730001 | 0,272000015 | 0,255299985 | 0,256500006 | 0,277099997 | 0,264800012 | 0,253899992 | 0,251199991 |
| 120 | 0,253600001 | 0,254099995 | 0,273600012 | | 0,286799997 | 0,265399992 | 0,261200011 | 0,272700012 | 0,277399987 | 0,257299989 | 0,25999999 | 0,284000009 | 0,269400001 | 0,256799996 | 0,255100012 |
| 140 | 0,253800005 | 0,255199999 | 0,278600007 | | 0,292600006 | 0,2685 | 0,265100002 | 0,277500004 | 0,282999992 | 0,260500014 | 0,262699991 | 0,289700001 | 0,273499995 | 0,258599997 | 0,257299989 |
| 160 | 0,252900004 | 0,254200011 | 0,282799989 | | 0,299600005 | 0,272300005 | 0,269800007 | 0,281800002 | 0,286799997 | 0,264299989 | 0,267199993 | 0,295300007 | 0,275700003 | 0,260800004 | 0,25999999 |
| 180 | 0,254400015 | 0,255299985 | 0,287699997 | | 0,305900007 | 0,2773 | 0,273499995 | 0,286900014 | 0,291399986 | 0,268000007 | 0,269300014 | 0,30309999 | 0,280900002 | 0,265599996 | 0,261500001 |
| 200 | 0,253600001 | 0,256000012 | 0,292899996 | | 0,3125 | 0,279500008 | 0,27610001 | 0,291900009 | 0,297699988 | 0,271200001 | 0,273099989 | 0,309399992 | 0,286199987 | 0,268900007 | 0,264699996 |
| 220 | 0,251599997 | 0,254000008 | 0,296600014 | | 0,317099988 | 0,282000005 | 0,277999997 | 0,296099991 | 0,301699996 | 0,271899998 | 0,274599999 | 0,313499987 | 0,288399994 | 0,268299997 | 0,265799999 |
| 240 | 0,252799988 | 0,254400015 | 0,301299989 | | 0,324400008 | 0,287 | 0,282499999 | 0,301400006 | 0,306699991 | 0,275299996 | 0,278299987 | 0,320300013 | 0,29339999 | 0,270900011 | 0,268599987 |
| 260 | 0,252700001 | 0,254099995 | 0,305999994 | | 0,330599993 | 0,289900005 | 0,286599994 | 0,306100011 | 0,311500013 | 0,277500004 | 0,281399995 | 0,326900005 | 0,297100008 | 0,273699999 | 0,270300001 |
| 280 | 0,251899987 | 0,253100008 | 0,310200006 | | 0,335599989 | 0,293099999 | 0,288599998 | 0,309899986 | 0,316300005 | 0,281599998 | 0,284000009 | 0,333099991 | 0,300500005 | 0,2755 | 0,271899998 |
| 300 | 0,252499998 | 0,253100008 | 0,314700007 | | 0,342500001 | 0,296000004 | 0,290300012 | 0,314500004 | 0,320899993 | 0,282599986 | 0,2861 | 0,337500006 | 0,303299993 | 0,278200001 | 0,274500012 |
| 320 | 0,251399994 | 0,254299998 | 0,320300013 | | 0,349799991 | 0,301600009 | 0,295300007 | 0,320100009 | 0,326599985 | 0,286300004 | 0,290199995 | 0,344599992 | 0,308800012 | 0,282200009 | 0,27759999 |
| 340 | 0,252900004 | 0,253100008 | 0,325899988 | | 0,355699986 | 0,305599988 | 0,299299985 | 0,325899988 | 0,332399994 | 0,289900005 | 0,294099987 | 0,351700008 | 0,314099997 | 0,284200013 | 0,279199988 |
| 360 | 0,251399994 | 0,250999987 | 0,328099996 | | 0,359600008 | 0,306699991 | 0,300599992 | 0,328000009 | 0,335200012 | 0,291299999 | 0,294999987 | 0,35589999 | 0,316199988 | 0,285299987 | 0,280499995 |
| slope | -8,21468E-06 | -8,45201E-06 | 0,00022856 | | 0,000305217 | 0,000168411 | 0,000154138 | 0,000231538 | 0,000239009 | 0,000131434 | 0,000145072 | 0,000301383 | 0,000195077 | 0,00012033 | 0,000105243 |
| slope w/o |  |  | 0,000236894 | | 0,00031355 | 0,000176744 | 0,000162472 | 0,000239871 | 0,000247343 | 0,000139768 | 0,000153406 | 0,000309716 | 0,000203411 | 0,000128664 | 0,000113576 |
| blank |  |  |  |  | 0,000275222 |  | 0,000169608 |  | 0,000243607 |  | 0,000146587 |  | 0,000256563 |  | 0,00012112 |
|  |  |  |  |  | 0,016513313 |  | 0,01017647 |  | 0,014616409 |  | 0,008795202 |  | 0,015393808 |  | 0,007267184 |
|  |  |  |  |  | 2,464673613 |  | 1,518876159 |  | 2,181553603 |  | 1,312716642 |  | 2,297583321 |  | 1,08465428 |
|  |  |  |  |  | 41,07789356 |  | 25,31460265 |  | 36,35922671 |  | 21,8786107 |  | 38,29305536 |  | 18,07757134 |
|  |  |  | **min -1** |  | 41,07789356 |  | 25,31460265 |  | 36,35922671 |  | 21,8786107 |  | 38,29305536 |  | 18,07757134 |
|  |  | **5 min** |  |  |  |  |  | 30 |  |  |  |  |  |  |  |
|  | **PTPN22 wt control** | 41,07789356 |  | 1 | 100 |  |  | 76,05790021 |  | 36,29645354 | 40,2877268 | 42,29972198 |  |  |  |
|  | **PTPN22 mut control** | 25,31460265 |  | 1 | 100 |  |  | 4,469147798 |  | 38,83254163 | 35,511682 | 33,24784231 |  |  |  |
|  | **PTPN22 wt 25uM H2O2** | 36,35922671 |  | 0,885128802 | 88,5128802 |  |  | 97,26724967 |  | 34,31834659 | 39,67431092 | **31,9840417** |  |  |  |
|  | **PTPN22 mut 25uM H2O2** | 21,8786107 |  | 0,864268383 | 86,4268383 |  |  | 31,22145889 |  | 36,95069113 | 34,9051965 | 31,260104 |  |  |  |
|  | **PTPN22 wt 50uM H2O2** | 38,29305536 |  | 0,932205915 | 93,22059152 |  |  |  |  | 31,57471215 | 36,20904822 | **33,80080718** |  |  |  |
|  | **PTPN22 mut 50uM H2O2** | 18,07757134  #DIV/0! #DIV/0! |  | 0,714116338 | 71,41163378 |  |  |  |  | 34,54707642 | 33,41881996 | **32,142307** |  |  |  |

**PTPN22 reduced control PTPN22 3.4uM H2O2 PTPN22 6.3uM H2O2 PTPN22 12.5uM H2O2 PTPN22 25uM H2O2 PTPN22 50uM H2O2**

Protocol Layout:

- 20mM Hepes Buffer 0,1mM DTPA, pH 7.4, 10min
- Wash 1ml spinn collumn slurry 5x with Hepes Buffer
- Spinn 1000 rpm for 2 min, apply 50μl PTP1B from Freezer (recombinant HishPTP1B in 20mM Hepes, 1mM TCEP), ( desalt 1x)
- Measure PTP1B (reduced PTP) and protein concentration with Bradford -> (0,585/1,16) X5 -> - 0,5 μg/μl
- Prepare 16.7mM (15mM end concentration) pNPP (Sigma) substrate sollution Mw 371

**PTP1B activity catalytic domain exposed to 50μM H2O2 and 25mM Bicarbonat and TrxR1, Trx, Prx2**

- prepare 20μl PTP1B, 600nM
- add 20μl from each condition in duplicates to 96-well plate
- Add 180μl substrate solution


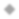

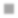

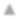

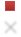

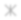

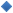

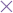

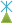

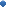

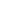

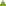

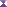

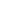


45

40

35

30

25

20

15

PTPN22 wt control #REF!

PTPN22 mut control PTPN22 wt 25uM H2O2

PTPN22 mut 25uM H2O2

10

5

0

0

0,2 0,4 0,6 0,8 1

1,2

Application: Tecan i-control Tecan i-control , 2.0.10.0

Device: infinite 200Pro Serial number: 1307001123 Serial number of connected stacker: Firmware: V_3.40_01/15_Infinite (Dec 23 201 MAI, V_3.40_01/15_Infinite (Dec 23 2014/12.45.11)

Date: #########

Time: 13:25:44

System MTC-MU059-S

User MTC-MU059-S\fretho

Plate Greiner 96 Flat Bottom Transparent Polystyrene Cat. No.: 655101/655161/655192 [GRE96ft.pdfx] Plate-ID (Stacker)

List of actions in this measurement script: Kinetic

Absorbance

Label: Label1

Kinetic Measurement

Kinetic duration 00:06:00

Interval Time 00:00:20

Measurement Wavelength 405 nm

Bandwidth 10 nm

Number of Flashes 5

Settle Time 0 ms

Part of Plate A1-A12; B1-B2

Start Time: 2020 05-19 13:25:46

| Cycle Nr. | 1 | 2 | 3 | 4 | 5 | 6 | 7 | 8 | 9 | 10 | 11 | 12 | 13 | 14 | 15 | 16 | 17 | 18 | 19 |
| --- | --- | --- | --- | --- | --- | --- | --- | --- | --- | --- | --- | --- | --- | --- | --- | --- | --- | --- | --- |
| Time [s] | 0 | 20 | 40 | 60 | 80 | 100 | 120 | 140 | 160 | 180 | 200 | 220 | 240 | 260 | 280 | 300 | 320 | 340 | 360 |
| Temp. [°C] | 24,3 | 24,2 | 24,5 | 24,3 | 24,4 | 24,5 | 24,4 | 24,4 | 24,6 | 24,5 | 24,7 | 24,5 | 24,3 | 24,7 | 24,7 | 24,6 | 24,6 | 24,3 | 24,4 |
| A1 | 0,2476 | 0,248 | 0,2534 | 0,259 | 0,2657 | 0,2709 | 0,2745 | 0,283 | 0,2884 | 0,2951 | 0,301 | 0,3074 | 0,3136 | 0,3202 | 0,3249 | 0,3303 | 0,3359 | 0,3421 | 0,3479 |
| A2 | 0,2479 | 0,2478 | 0,2539 | 0,2578 | 0,2618 | 0,2677 | 0,272 | 0,2778 | 0,2836 | 0,2894 | 0,2936 | 0,2999 | 0,3043 | 0,3109 | 0,3147 | 0,3208 | 0,325 | 0,3308 | 0,3354 |
| A3 | 0,2409 | 0,2435 | 0,2462 | 0,2499 | 0,2525 | 0,2539 | 0,2566 | 0,2609 | 0,2649 | 0,2678 | 0,2723 | 0,2746 | 0,2779 | 0,2838 | 0,2846 | 0,288 | 0,2904 | 0,2945 | 0,2967 |
| A4 | 0,2429 | 0,2449 | 0,2469 | 0,2499 | 0,2535 | 0,2562 | 0,2574 | 0,2622 | 0,266 | 0,2704 | 0,2744 | 0,278 | 0,2793 | 0,2852 | 0,2866 | 0,2916 | 0,2941 | 0,2977 | 0,3 |
| A5 | 0,2454 | 0,2464 | 0,2481 | 0,2512 | 0,2542 | 0,2567 | 0,259 | 0,2653 | 0,2677 | 0,272 | 0,2773 | 0,2813 | 0,2825 | 0,2878 | 0,2903 | 0,2948 | 0,2993 | 0,302 | 0,3049 |
| A6 | 0,2497 | 0,2489 | 0,2533 | 0,2553 | 0,2594 | 0,2623 | 0,2657 | 0,2727 | 0,2779 | 0,2825 | 0,2863 | 0,2905 | 0,2955 | 0,2988 | 0,3037 | 0,3076 | 0,3126 | 0,3162 | 0,3203 |
| A7 | 0,2422 | 0,2416 | 0,2438 | 0,2447 | 0,2475 | 0,2476 | 0,2493 | 0,2528 | 0,2557 | 0,257 | 0,2607 | 0,2623 | 0,2636 | 0,2667 | 0,2679 | 0,271 | 0,2739 | 0,2746 | 0,2755 |
| A8 | 0,2428 | 0,2409 | 0,2431 | 0,2467 | 0,2463 | 0,2492 | 0,2499 | 0,254 | 0,2558 | 0,2582 | 0,2605 | 0,2683 | 0,2631 | 0,2674 | 0,2684 | 0,2715 | 0,2735 | 0,2748 | 0,2771 |
| A9 | 0,2421 | 0,2412 | 0,2431 | 0,2455 | 0,2471 | 0,2494 | 0,2491 | 0,2535 | 0,2555 | 0,2592 | 0,2609 | 0,264 | 0,2646 | 0,2683 | 0,2698 | 0,2728 | 0,2742 | 0,2767 | 0,28 |
| A10 | 0,242 | 0,2429 | 0,2479 | 0,2513 | 0,2532 | 0,2572 | 0,2574 | 0,2642 | 0,269 | 0,2716 | 0,276 | 0,2805 | 0,2825 | 0,2872 | 0,2894 | 0,2943 | 0,2976 | 0,301 | 0,3056 |
| A11 | 0,2409 | 0,2403 | 0,2419 | 0,2435 | 0,244 | 0,2452 | 0,2452 | 0,2495 | 0,2514 | 0,2531 | 0,2541 | 0,2549 | 0,2557 | 0,2583 | 0,259 | 0,2615 | 0,2625 | 0,2625 | 0,2646 |
| A12 | 0,2406 | 0,2409 | 0,2432 | 0,2432 | 0,2425 | 0,2443 | 0,2444 | 0,2478 | 0,2495 | 0,2507 | 0,252 | 0,254 | 0,2534 | 0,2575 | 0,2579 | 0,2594 | 0,2606 | 0,2612 | 0,2624 |
| B1 | 0,2497 | 0,2493 | 0,2506 | 0,2498 | 0,2497 | 0,2487 | 0,2478 | 0,2496 | 0,2505 | 0,2509 | 0,2506 | 0,2518 | 0,2506 | 0,2516 | 0,2505 | 0,2515 | 0,2501 | 0,2499 | 0,25 |
| B2 | 0,252 | 0,2515 | 0,2503 | 0,2522 | 0,251 | 0,2509 | 0,2488 | 0,2508 | 0,2512 | 0,2505 | 0,2502 | 0,2509 | 0,2501 | 0,2511 | 0,2504 | 0,2519 | 0,2509 | 0,2496 | 0,2514 |

**170330**

**Time (sec) Blank Blank**

**PTPN22 wt control PTPN22 wt control PTPN22 mut control PTPN22 mut control PTPN22 wt 25uM H2O2 PTPN22 wt 25uM H2O2 PTPN22 mut 25uM H2O2 PTPN22 mut 25uM H2O2 PTPN22 wt 50uM H2O PTPN22 wt 50uM H2O PTPN22 mut 50uM H2O2 PTPN22 mut 50uM H2O2**

| 20 | 0,249300003 | 0,25150001 | 0,247999996 | | 0,247799993 | 0,243499994 | 0,244900003 | 0,246399999 | 0,248899996 | 0,241600007 | 0,240899995 | 0,2412 | 0,242899999 | 0,2403 | 0,240899995 |
| --- | --- | --- | --- | --- | --- | --- | --- | --- | --- | --- | --- | --- | --- | --- | --- |
| 40 | 0,25060001 | 0,25029999 | 0,253399998 | | 0,253899992 | 0,246199995 | 0,246900007 | 0,248099998 | 0,253300011 | 0,243799999 | 0,243100002 | 0,243100002 | 0,247899994 | 0,241899997 | 0,243200004 |
| 60 | 0,249799997 | 0,252200007 | 0,259000003 | | 0,257800013 | 0,249899998 | 0,249899998 | 0,251199991 | 0,255299985 | 0,2447 | 0,246700004 | 0,245499998 | 0,251300007 | 0,243499994 | 0,243200004 |
| 80 | 0,249699995 | 0,250999987 | 0,265700012 | | 0,261799991 | 0,252499998 | 0,253500015 | 0,254200011 | 0,25940001 | 0,247500002 | 0,246299997 | 0,247099996 | 0,253199995 | 0,244000003 | 0,242500007 |
| 100 | 0,248699993 | 0,2509 | 0,270900011 | | 0,267699987 | 0,253899992 | 0,256199986 | 0,256700009 | 0,262300014 | 0,247600004 | 0,249200001 | 0,249400005 | 0,257200003 | 0,245199993 | 0,244299993 |
| 120 | 0,247799993 | 0,248799995 | 0,274500012 | | 0,272000015 | 0,256599993 | 0,257400006 | 0,259000003 | 0,265700012 | 0,249300003 | 0,249899998 | 0,2491 | 0,257400006 | 0,245199993 | 0,244399995 |
| 140 | 0,249599993 | 0,250800014 | 0,282999992 | | 0,277799994 | 0,260899991 | 0,262199998 | 0,265300006 | 0,272700012 | 0,252799988 | 0,254000008 | 0,253500015 | 0,264200002 | 0,249500006 | 0,247799993 |
| 160 | 0,250499994 | 0,251199991 | 0,288399994 | | 0,283600003 | 0,264899999 | 0,266000003 | 0,267699987 | 0,27790001 | 0,255699992 | 0,255800009 | 0,255499989 | 0,268999994 | 0,251399994 | 0,249500006 |
| 180 | 0,2509 | 0,250499994 | 0,295100003 | | 0,289400011 | 0,267800003 | 0,270399988 | 0,272000015 | 0,282499999 | 0,256999999 | 0,25819999 | 0,259200007 | 0,271600008 | 0,253100008 | 0,250699997 |
| 200 | 0,25060001 | 0,250200003 | 0,300999999 | | 0,293599993 | 0,272300005 | 0,274399996 | 0,2773 | 0,286300004 | 0,260699987 | 0,260500014 | 0,260899991 | 0,275999993 | 0,254099995 | 0,252000004 |
| 220 | 0,251800001 | 0,2509 | 0,307399988 | | 0,299899995 | 0,274599999 | 0,277999997 | 0,281300008 | 0,290499985 | 0,262300014 | 0,268299997 | 0,263999999 | 0,280499995 | 0,254900008 | 0,254000008 |
| 240 | 0,25060001 | 0,250099987 | 0,313600004 | | 0,30430001 | 0,27790001 | 0,279300004 | 0,282499999 | 0,29550001 | 0,263599992 | 0,263099998 | 0,264600009 | 0,282499999 | 0,255699992 | 0,253399998 |
| 260 | 0,251599997 | 0,251100004 | 0,320199996 | | 0,310900003 | 0,283800006 | 0,2852 | 0,287800014 | 0,298799992 | 0,2667 | 0,267399997 | 0,268299997 | 0,287200004 | 0,258300006 | 0,257499993 |
| 280 | 0,250499994 | 0,250400007 | 0,324900001 | | 0,314700007 | 0,28459999 | 0,286599994 | 0,290300012 | 0,3037 | 0,26789999 | 0,268400013 | 0,269800007 | 0,289400011 | 0,259000003 | 0,2579 |
| 300 | 0,25150001 | 0,251899987 | 0,330300003 | | 0,320800006 | 0,287999988 | 0,291599989 | 0,294800013 | 0,307599992 | 0,270999998 | 0,271499991 | 0,272799999 | 0,29429999 | 0,261500001 | 0,25940001 |
| 320 | 0,250099987 | 0,2509 | 0,335900009 | | 0,324999988 | 0,290399998 | 0,294099987 | 0,299299985 | 0,312599987 | 0,273900002 | 0,273499995 | 0,274199992 | 0,297600001 | 0,262499988 | 0,260600001 |
| 340 | 0,249899998 | 0,249599993 | 0,342099994 | | 0,330799997 | 0,294499993 | 0,297699988 | 0,301999986 | 0,316199988 | 0,274599999 | 0,274800003 | 0,27669999 | 0,300999999 | 0,262499988 | 0,261200011 |
| 360 | 0,25 | 0,251399994 | 0,347900003 | | 0,335399985 | 0,296700001 | 0,300000012 | 0,304899991 | 0,320300013 | 0,2755 | 0,277099997 | 0,280000001 | 0,305599988 | 0,264600009 | 0,262400001 |
| slope | 3,94737E-06 | -6,3469E-07 | 0,00029726 | | 0,000260382 | 0,000161569 | 0,000169561 | 0,000181166 | 0,000216321 | 0,000106213 | 0,000108209 | 0,000114556 | 0,000182931 | 7,32714E-05 | 6,73117E-05 |
| slope w/o |  |  | 0,000295604 | | 0,000258725 | 0,000159912 | 0,000167905 | 0,00017951 | 0,000214665 | 0,000104556 | 0,000106553 | 0,0001129 | 0,000181275 | 7,16151E-05 | 6,56553E-05 |
| blank |  | |  |  | 0,000277165 |  | 0,000163909 |  | 0,000197087 |  | 0,000105555 |  | 0,000147087 |  | 6,86352E-05 |
|  |  | |  |  | 0,016629876 |  | 0,00983452 |  | 0,011825231 |  | 0,006333282 |  | 0,008825232 |  | 0,004118112 |
|  |  | |  |  | 2,482071103 |  | 1,467838815 |  | 1,764959909 |  | 0,945265933 |  | 1,31719881 |  | 0,614643656 |
|  |  | |  |  | 41,36785172 |  | 24,46398024 |  | 29,41599849 |  | 15,75443221 |  | 21,95331349 |  | 10,24406093 |
|  |  | | **min -1** |  | 41,36785172 |  | 24,46398024 |  | 29,41599849 |  | 15,75443221 |  | 21,95331349 |  | 10,24406093 |
|  | **15 min PTPN22 wt control** 41,36785172 | |  | 1 | 100 |  |  |  |  |  |  |  |  |  |  |
|  | **PTPN22 mut control** 24,46398024 | |  | 1 | 100 |  |  |  |  |  |  |  |  |  |  |
|  | **PTPN22 wt 25uM H2O2** 29,41599849 | |  | 0,711083541 | 71,10835411 |  |  |  |  |  |  |  |  |  |  |
|  | **PTPN22 mut 25uM H2O2** 15,75443221 | |  | 0,643984832 | 64,39848323 |  |  |  |  |  |  |  |  |  |  |
|  | **PTPN22 wt 50uM H2O2** 21,95331349 | |  | 0,530685365 | 53,06853651 |  |  |  |  |  |  |  |  |  |  |
|  | **PTPN22 mut 50uM H2O2** 10,24406093 | |  | 0,418740566 | 41,8740566 |  |  |  |  |  |  |  |  |  |  |

#DIV/0! #DIV/0!

Protocol Layout:

- 20mM Hepes Buffer 0,1mM DTPA, pH 7.4, 10min
- Wash 1ml spinn collumn slurry 5x with Hepes Buffer
- Spinn 1000 rpm for 2 min, apply 50μl PTP1B from Freezer (recombinant HishPTP1B in 20mM Hepes, 1mM TCEP), ( desalt 1x)
- Measure PTP1B (reduced PTP) and protein concentration with Bradford -> (0,585/1,16) X5 -> - 0,5 μg/μl
- Prepare 16.7mM (15mM end concentration) pNPP (Sigma) substrate sollution Mw 371

**PTP1B activity catalytic domain exposed to 50μM H2O2 and 25mM Bicarbonat and TrxR1, Trx, Prx2**

- prepare 20μl PTP1B, 600nM
- add 20μl from each condition in duplicates to 96-well plate
- Add 180μl substrate solution


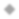

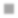

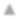

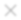

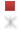

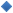

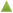

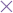

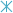

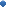

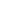

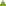

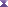

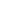


45

40

35

30

25

20

15

PTPN22 wt control #REF!

PTPN22 mut control PTPN22 wt 25uM H2O2

PTPN22 mut 25uM H2O2

10

5

0

0

0,2 0,4 0,6 0,8 1

1,2

Application: Tecan i-control Tecan i-control , 2.0.10.0

Device: infinite 200Pro Serial number: 1307001123 Serial number of connected stacker: Firmware: V_3.40_01/15_Infinite (Dec 23 201 MAI, V_3.40_01/15_Infinite (Dec 23 2014/12.45.11)

Date: #########

Time: 13:25:44

System MTC-MU059-S

User MTC-MU059-S\fretho

Plate Greiner 96 Flat Bottom Transparent Polystyrene Cat. No.: 655101/655161/655192 [GRE96ft.pdfx] Plate-ID (Stacker)

List of actions in this measurement script: Kinetic

Absorbance

Label: Label1

Kinetic Measurement

Kinetic duration 00:06:00

Interval Time 00:00:20

Measurement Wavelength 405 nm

Bandwidth 10 nm

Number of Flashes 5

Settle Time 0 ms

Part of Plate A1-A12; B1-B2

Start Time: 2020 05-19 13:25:46

| Cycle Nr. | 1 | 2 | 3 | 4 | 5 | 6 | 7 | 8 | 9 | 10 | 11 | 12 | 13 | 14 | 15 | 16 | 17 | 18 | 19 |
| --- | --- | --- | --- | --- | --- | --- | --- | --- | --- | --- | --- | --- | --- | --- | --- | --- | --- | --- | --- |
| Time [s] | 0 | 20 | 40 | 60 | 80 | 100 | 120 | 140 | 160 | 180 | 200 | 220 | 240 | 260 | 280 | 300 | 320 | 340 | 360 |
| Temp. [°C] | 24,3 | 24,2 | 24,5 | 24,3 | 24,4 | 24,5 | 24,4 | 24,4 | 24,6 | 24,5 | 24,7 | 24,5 | 24,3 | 24,7 | 24,7 | 24,6 | 24,6 | 24,3 | 24,4 |
| A1 | 0,2476 | 0,248 | 0,2534 | 0,259 | 0,2657 | 0,2709 | 0,2745 | 0,283 | 0,2884 | 0,2951 | 0,301 | 0,3074 | 0,3136 | 0,3202 | 0,3249 | 0,3303 | 0,3359 | 0,3421 | 0,3479 |
| A2 | 0,2479 | 0,2478 | 0,2539 | 0,2578 | 0,2618 | 0,2677 | 0,272 | 0,2778 | 0,2836 | 0,2894 | 0,2936 | 0,2999 | 0,3043 | 0,3109 | 0,3147 | 0,3208 | 0,325 | 0,3308 | 0,3354 |
| A3 | 0,2409 | 0,2435 | 0,2462 | 0,2499 | 0,2525 | 0,2539 | 0,2566 | 0,2609 | 0,2649 | 0,2678 | 0,2723 | 0,2746 | 0,2779 | 0,2838 | 0,2846 | 0,288 | 0,2904 | 0,2945 | 0,2967 |
| A4 | 0,2429 | 0,2449 | 0,2469 | 0,2499 | 0,2535 | 0,2562 | 0,2574 | 0,2622 | 0,266 | 0,2704 | 0,2744 | 0,278 | 0,2793 | 0,2852 | 0,2866 | 0,2916 | 0,2941 | 0,2977 | 0,3 |
| A5 | 0,2454 | 0,2464 | 0,2481 | 0,2512 | 0,2542 | 0,2567 | 0,259 | 0,2653 | 0,2677 | 0,272 | 0,2773 | 0,2813 | 0,2825 | 0,2878 | 0,2903 | 0,2948 | 0,2993 | 0,302 | 0,3049 |
| A6 | 0,2497 | 0,2489 | 0,2533 | 0,2553 | 0,2594 | 0,2623 | 0,2657 | 0,2727 | 0,2779 | 0,2825 | 0,2863 | 0,2905 | 0,2955 | 0,2988 | 0,3037 | 0,3076 | 0,3126 | 0,3162 | 0,3203 |
| A7 | 0,2422 | 0,2416 | 0,2438 | 0,2447 | 0,2475 | 0,2476 | 0,2493 | 0,2528 | 0,2557 | 0,257 | 0,2607 | 0,2623 | 0,2636 | 0,2667 | 0,2679 | 0,271 | 0,2739 | 0,2746 | 0,2755 |
| A8 | 0,2428 | 0,2409 | 0,2431 | 0,2467 | 0,2463 | 0,2492 | 0,2499 | 0,254 | 0,2558 | 0,2582 | 0,2605 | 0,2683 | 0,2631 | 0,2674 | 0,2684 | 0,2715 | 0,2735 | 0,2748 | 0,2771 |
| A9 | 0,2421 | 0,2412 | 0,2431 | 0,2455 | 0,2471 | 0,2494 | 0,2491 | 0,2535 | 0,2555 | 0,2592 | 0,2609 | 0,264 | 0,2646 | 0,2683 | 0,2698 | 0,2728 | 0,2742 | 0,2767 | 0,28 |
| A10 | 0,242 | 0,2429 | 0,2479 | 0,2513 | 0,2532 | 0,2572 | 0,2574 | 0,2642 | 0,269 | 0,2716 | 0,276 | 0,2805 | 0,2825 | 0,2872 | 0,2894 | 0,2943 | 0,2976 | 0,301 | 0,3056 |
| A11 | 0,2409 | 0,2403 | 0,2419 | 0,2435 | 0,244 | 0,2452 | 0,2452 | 0,2495 | 0,2514 | 0,2531 | 0,2541 | 0,2549 | 0,2557 | 0,2583 | 0,259 | 0,2615 | 0,2625 | 0,2625 | 0,2646 |
| A12 | 0,2406 | 0,2409 | 0,2432 | 0,2432 | 0,2425 | 0,2443 | 0,2444 | 0,2478 | 0,2495 | 0,2507 | 0,252 | 0,254 | 0,2534 | 0,2575 | 0,2579 | 0,2594 | 0,2606 | 0,2612 | 0,2624 |
| B1 | 0,2497 | 0,2493 | 0,2506 | 0,2498 | 0,2497 | 0,2487 | 0,2478 | 0,2496 | 0,2505 | 0,2509 | 0,2506 | 0,2518 | 0,2506 | 0,2516 | 0,2505 | 0,2515 | 0,2501 | 0,2499 | 0,25 |
| B2 | 0,252 | 0,2515 | 0,2503 | 0,2522 | 0,251 | 0,2509 | 0,2488 | 0,2508 | 0,2512 | 0,2505 | 0,2502 | 0,2509 | 0,2501 | 0,2511 | 0,2504 | 0,2519 | 0,2509 | 0,2496 | 0,2514 |

**170330**

**Time (sec) Blank Blank**

**PTPN22 wt control PTPN22 wt control PTPN22 mut control PTPN22 mut control PTPN22 wt 25uM H2O2 PTPN22 wt 25uM H2O2 PTPN22 mut 25uM H2O2 PTPN22 mut 25uM H2O2 PTPN22 wt 50uM H2O PTPN22 wt 50uM H2O PTPN22 mut 50uM H2O2 PTPN22 mut 50uM H2O2**

| 20 | 0,252600014 0,256000012 | 0,251700014 | | 0,246800005 | 0,243100002 | 0,2447 | 0,245199993 | 0,248600006 | 0,239999995 | 0,241600007 | 0,242599994 | 0,243100002 | 0,238000005 | 0,241899997 |
| --- | --- | --- | --- | --- | --- | --- | --- | --- | --- | --- | --- | --- | --- | --- |
| 40 | 0,252400011 0,254000008 | 0,256000012 | | 0,251700014 | 0,244800001 | 0,246299997 | 0,245399997 | 0,249300003 | 0,240199998 | 0,244200006 | 0,243000001 | 0,245100006 | 0,239399999 | 0,242799997 |
| 60 | 0,251800001 0,254400015 | 0,262600005 | | 0,258300006 | 0,249300003 | 0,249699995 | 0,248300001 | 0,254000008 | 0,242899999 | 0,247199997 | 0,243599996 | 0,246399999 | 0,240700006 | 0,241899997 |
| 80 | 0,251399994 0,253600001 | 0,2685 | | 0,261799991 | 0,251599997 | 0,250999987 | 0,249799997 | 0,255600005 | 0,241500005 | 0,245100006 | 0,245100006 | 0,247799993 | 0,239500001 | 0,241600007 |
| 100 | 0,252700001 0,254099995 | 0,273699999 | | 0,2676 | 0,254799992 | 0,254700005 | 0,252999991 | 0,259499997 | 0,243300006 | 0,247999996 | 0,2465 | 0,2491 | 0,240799993 | 0,242599994 |
| 120 | 0,253199995 0,253300011 | 0,279100001 | | 0,272799999 | 0,258599997 | 0,257999986 | 0,256599993 | 0,264099985 | 0,244100004 | 0,247999996 | 0,247899994 | 0,251100004 | 0,241699994 | 0,243000001 |
| 140 | 0,252799988 0,254900008 | 0,286500007 | | 0,277999997 | 0,263399988 | 0,261500001 | 0,261299998 | 0,267199993 | 0,247799993 | 0,2491 | 0,251300007 | 0,254200011 | 0,242500007 | 0,2447 |
| 160 | 0,251700014 0,253399998 | 0,291500002 | | 0,283899993 | 0,264299989 | 0,264800012 | 0,263000011 | 0,269899994 | 0,246399999 | 0,248799995 | 0,251399994 | 0,254000008 | 0,2421 | 0,244499996 |
| 180 | 0,253899992 0,253899992 | 0,297199994 | | 0,287999988 | 0,268599987 | 0,26789999 | 0,2667 | 0,271899998 | 0,248600006 | 0,249899998 | 0,252900004 | 0,256500006 | 0,242799997 | 0,245900005 |
| 200 | 0,253699988 0,254599988 | 0,3046 | | 0,293199986 | 0,271499991 | 0,271400005 | 0,269800007 | 0,276199996 | 0,251199991 | 0,251700014 | 0,254200011 | 0,259799987 | 0,243399993 | 0,246600002 |
| 220 | 0,252200007 0,254599988 | 0,310000002 | | 0,298099995 | 0,273900002 | 0,275200009 | 0,272799999 | 0,278699994 | 0,251399994 | 0,253399998 | 0,256199986 | 0,25999999 | 0,244599998 | 0,246800005 |
| 240 | 0,253699988 0,254299998 | 0,316799998 | | 0,304500014 | 0,278899997 | 0,278600007 | 0,27669999 | 0,284299999 | 0,254599988 | 0,254500002 | 0,259000003 | 0,263099998 | 0,245900005 | 0,248699993 |
| 260 | 0,25150001 0,253500015 | 0,321999997 | | 0,308499992 | 0,279900014 | 0,280200005 | 0,279599994 | 0,28490001 | 0,253300011 | 0,254200011 | 0,257499993 | 0,262600005 | 0,244900003 | 0,247199997 |
| 280 | 0,253100008 0,253399998 | 0,327199996 | | 0,313499987 | 0,282799989 | 0,283499986 | 0,282200009 | 0,289900005 | 0,254000008 | 0,255600005 | 0,260100007 | 0,2667 | 0,245000005 | 0,247600004 |
| 300 | 0,253199995 0,254999995 | 0,334800005 | | 0,319599986 | 0,287400007 | 0,287699997 | 0,286900014 | 0,293799996 | 0,257999986 | 0,257499993 | 0,262699991 | 0,26879999 | 0,246600002 | 0,248899996 |
| 320 | 0,253600001 0,254099995 | 0,341300011 | | 0,323300004 | 0,290100008 | 0,290600002 | 0,288500011 | 0,296499997 | 0,2588 | 0,258700013 | 0,264899999 | 0,270300001 | 0,246099994 | 0,251100004 |
| 340 | 0,253300011 0,254500002 | 0,345400006 | | 0,328700006 | 0,293199986 | 0,29339999 | 0,291500002 | 0,299899995 | 0,2588 | 0,259000003 | 0,265199989 | 0,272100002 | 0,246999994 | 0,249699995 |
| 360 | 0,252999991 0,255100012 | 0,352299988 | | 0,334300011 | 0,295100003 | 0,295399994 | 0,29519999 | 0,301600009 | 0,260399997 | 0,260699987 | 0,266499996 | 0,272799999 | 0,247600004 | 0,250200003 |
| slope | 3,14756E-06 5,13621E-09 | 0,000299267 | | 0,000256275 | 0,000157095 | 0,000156791 | 0,000155083 | 0,000163473 | 6,32766E-05 | 5,09288E-05 | 7,50051E-05 | 9,06243E-05 | 2,63571E-05 | 2,81992E-05 |
| slope w/o |  | 0,000297691 | | 0,000254698 | 0,000155519 | 0,000155214 | 0,000153506 | 0,000161896 | 6,17002E-05 | 4,93524E-05 | 7,34288E-05 | 8,9048E-05 | 2,47807E-05 | 2,66228E-05 |
| blank |  |  |  | 0,000276195 |  | 0,000155366 |  | 0,000157701 |  | 5,55263E-05 |  | 8,12384E-05 |  | 2,57018E-05 |
|  |  |  |  | 0,016571672 |  | 0,009321983 |  | 0,009462076 |  | 0,00333158 |  | 0,004874304 |  | 0,001542106 |
|  |  |  |  | 2,473383855 |  | 1,3913407 |  | 1,41225015 |  | 0,497250702 |  | 0,727508009 |  | 0,230165118 |
|  |  |  |  | 41,22306426 |  | 23,18901167 |  | 23,5375025 |  | 8,287511707 |  | 12,12513348 |  | 3,836085308 |
|  |  | **min -1** |  | 41,22306426 |  | 23,18901167 |  | 23,5375025 |  | 8,287511707 |  | 12,12513348 |  | 3,836085308 |
|  | **30 min PTPN22 wt control** 41,22306426 |  | 1 | 100 |  |  | 30 |  |  |  |  |  |  |  |
|  | **PTPN22 mut control** 23,18901167 |  | 1 | 100 |  |  |  |  |  |  |  |  |  |  |
|  | **PTPN22 wt 25uM H2O2** 23,5375025 |  | 0,570978963 | 57,09789635 |  |  |  |  |  |  |  |  |  |  |
|  | **PTPN22 mut 25uM H2O2** 8,287511707 |  | 0,357389604 | 35,73896043 |  |  |  |  |  |  |  |  |  |  |
|  | **PTPN22 wt 50uM H2O2** 12,12513348 |  | 0,294134696 | 29,41346962 |  |  |  |  |  |  |  |  |  |  |
|  | **PTPN22 mut 50uM H2O2** 3,836085308 |  | 0,165426857 | 16,54268566 |  |  |  |  |  |  |  |  |  |  |

#DIV/0! #DIV/0!

Protocol Layout:

- 20mM Hepes Buffer 0,1mM DTPA, pH 7.4, 10min
- Wash 1ml spinn collumn slurry 5x with Hepes Buffer
- Spinn 1000 rpm for 2 min, apply 50μl PTP1B from Freezer (recombinant HishPTP1B in 20mM Hepes, 1mM TCEP), ( desalt 1x)
- Measure PTP1B (reduced PTP) and protein concentration with Bradford -> (0,585/1,16) X5 -> - 0,5 μg/μl
- Prepare 16.7mM (15mM end concentration) pNPP (Sigma) substrate sollution Mw 371

**PTP1B activity catalytic domain exposed to 50μM H2O2 and 25mM Bicarbonat and TrxR1, Trx, Prx2**

- prepare 20μl PTP1B, 600nM
- add 20μl from each condition in duplicates to 96-well plate
- Add 180μl substrate solution


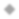

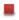

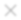

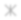

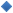

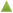

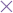

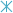

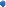

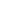

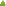

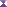

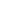


45

40

35

30

25

20

15

PTPN22 wt control #REF!

PTPN22 mut control PTPN22 wt 25uM H2O2

PTPN22 mut 25uM H2O2

10

5

0

0

0,2 0,4 0,6 0,8 1

1,2

Application: Tecan i-control Tecan i-control , 2.0.10.0

Device: infinite 200Pro Serial number: 1307001123 Serial number of connected stacker: Firmware: V_3.40_01/15_Infinite (Dec 23 201 MAI, V_3.40_01/15_Infinite (Dec 23 2014/12.45.11)

Date: #########

Time: 13:40:50

System MTC-MU059-S

User MTC-MU059-S\fretho

Plate Greiner 96 Flat Bottom Transparent Polystyrene Cat. No.: 655101/655161/655192 [GRE96ft.pdfx] Plate-ID (Stacker)

List of actions in this measurement script: Kinetic

Absorbance

Label: Label1

Kinetic Measurement

Kinetic duration 00:06:00

Interval Time 00:00:20

Measurement Wavelength 405 nm

Bandwidth 10 nm

Number of Flashes 5

Settle Time 0 ms

Part of Plate A1-A12; B1-B2

Start Time: 2020 05-19 13:40:53

| Cycle Nr. | 1 | 2 | 3 | 4 | 5 | 6 | 7 | 8 | 9 | 10 | 11 | 12 | 13 | 14 | 15 | 16 | 17 | 18 | 19 |
| --- | --- | --- | --- | --- | --- | --- | --- | --- | --- | --- | --- | --- | --- | --- | --- | --- | --- | --- | --- |
| Time [s] | 0 | 20 | 40 | 60 | 80 | 100 | 120 | 140 | 160 | 180 | 200 | 220 | 240 | 260 | 280 | 300 | 320 | 340 | 360 |
| Temp. [°C] | 24,6 | 24,4 | 24,4 | 24,5 | 24,6 | 24,5 | 24,6 | 25 | 25 | 24,7 | 25 | 24,6 | 24,5 | 24,4 | 24,5 | 24,6 | 24,8 | 24,6 | 24,8 |
| A1 | 0,2491 | 0,2517 | 0,256 | 0,2626 | 0,2685 | 0,2737 | 0,2791 | 0,2865 | 0,2915 | 0,2972 | 0,3046 | 0,31 | 0,3168 | 0,322 | 0,3272 | 0,3348 | 0,3413 | 0,3454 | 0,3523 |
| A2 | 0,2437 | 0,2468 | 0,2517 | 0,2583 | 0,2618 | 0,2676 | 0,2728 | 0,278 | 0,2839 | 0,288 | 0,2932 | 0,2981 | 0,3045 | 0,3085 | 0,3135 | 0,3196 | 0,3233 | 0,3287 | 0,3343 |
| A3 | 0,2407 | 0,2431 | 0,2448 | 0,2493 | 0,2516 | 0,2548 | 0,2586 | 0,2634 | 0,2643 | 0,2686 | 0,2715 | 0,2739 | 0,2789 | 0,2799 | 0,2828 | 0,2874 | 0,2901 | 0,2932 | 0,2951 |
| A4 | 0,2419 | 0,2447 | 0,2463 | 0,2497 | 0,251 | 0,2547 | 0,258 | 0,2615 | 0,2648 | 0,2679 | 0,2714 | 0,2752 | 0,2786 | 0,2802 | 0,2835 | 0,2877 | 0,2906 | 0,2934 | 0,2954 |
| A5 | 0,2426 | 0,2452 | 0,2454 | 0,2483 | 0,2498 | 0,253 | 0,2566 | 0,2613 | 0,263 | 0,2667 | 0,2698 | 0,2728 | 0,2767 | 0,2796 | 0,2822 | 0,2869 | 0,2885 | 0,2915 | 0,2952 |
| A6 | 0,2468 | 0,2486 | 0,2493 | 0,254 | 0,2556 | 0,2595 | 0,2641 | 0,2672 | 0,2699 | 0,2719 | 0,2762 | 0,2787 | 0,2843 | 0,2849 | 0,2899 | 0,2938 | 0,2965 | 0,2999 | 0,3016 |
| A7 | 0,2396 | 0,24 | 0,2402 | 0,2429 | 0,2415 | 0,2433 | 0,2441 | 0,2478 | 0,2464 | 0,2486 | 0,2512 | 0,2514 | 0,2546 | 0,2533 | 0,254 | 0,258 | 0,2588 | 0,2588 | 0,2604 |
| A8 | 0,2401 | 0,2416 | 0,2442 | 0,2472 | 0,2451 | 0,248 | 0,248 | 0,2491 | 0,2488 | 0,2499 | 0,2517 | 0,2534 | 0,2545 | 0,2542 | 0,2556 | 0,2575 | 0,2587 | 0,259 | 0,2607 |
| A9 | 0,2433 | 0,2426 | 0,243 | 0,2436 | 0,2451 | 0,2465 | 0,2479 | 0,2513 | 0,2514 | 0,2529 | 0,2542 | 0,2562 | 0,259 | 0,2575 | 0,2601 | 0,2627 | 0,2649 | 0,2652 | 0,2665 |
| A10 | 0,2442 | 0,2431 | 0,2451 | 0,2464 | 0,2478 | 0,2491 | 0,2511 | 0,2542 | 0,254 | 0,2565 | 0,2598 | 0,26 | 0,2631 | 0,2626 | 0,2667 | 0,2688 | 0,2703 | 0,2721 | 0,2728 |
| A11 | 0,2394 | 0,238 | 0,2394 | 0,2407 | 0,2395 | 0,2408 | 0,2417 | 0,2425 | 0,2421 | 0,2428 | 0,2434 | 0,2446 | 0,2459 | 0,2449 | 0,245 | 0,2466 | 0,2461 | 0,247 | 0,2476 |
| A12 | 0,2431 | 0,2419 | 0,2428 | 0,2419 | 0,2416 | 0,2426 | 0,243 | 0,2447 | 0,2445 | 0,2459 | 0,2466 | 0,2468 | 0,2487 | 0,2472 | 0,2476 | 0,2489 | 0,2511 | 0,2497 | 0,2502 |
| B1 | 0,2521 | 0,2526 | 0,2524 | 0,2518 | 0,2514 | 0,2527 | 0,2532 | 0,2528 | 0,2517 | 0,2539 | 0,2537 | 0,2522 | 0,2537 | 0,2515 | 0,2531 | 0,2532 | 0,2536 | 0,2533 | 0,253 |
| B2 | 0,2553 | 0,256 | 0,254 | 0,2544 | 0,2536 | 0,2541 | 0,2533 | 0,2549 | 0,2534 | 0,2539 | 0,2546 | 0,2546 | 0,2543 | 0,2535 | 0,2534 | 0,255 | 0,2541 | 0,2545 | 0,2551 |

**PTPN22 wt control PTPN22 wt control PTPN22 mut control PTPN22 mut control PTPN22 wt 25uM H2O2 PTPN22 wt 25uM H2O2 PTPN22 mut 25uM H2O2 PTPN22 mut 25uM H2O2 PTPN22 wt 50uM H2O PTPN22 wt 50uM H2O PTPN22 mut 50uM H2O2 PTPN22 mut 50uM H2O**

**Time (sec) Blank Blank**

| 20 | 0,254299998 | 0,256700009 | 0,257099986 | | 0,254999995 | 0,251100004 | 0,261799991 | 0,255499989 | 0,255800009 | 0,25029999 | 0,247400001 | 0,257200003 | 0,255100012 | 0,247199997 | 0,245199993 |
| --- | --- | --- | --- | --- | --- | --- | --- | --- | --- | --- | --- | --- | --- | --- | --- |
| 40 | 0,256300002 | 0,256900012 | 0,264800012 | | 0,267399997 | 0,259900004 | 0,257800013 | 0,267500013 | 0,262400001 | 0,253800005 | 0,252900004 | 0,261500001 | 0,25999999 | 0,251599997 | 0,249300003 |
| 60 | 0,255499989 | 0,256999999 | 0,268599987 | | 0,272300005 | 0,257099986 | 0,260600001 | 0,265899986 | 0,265799999 | 0,255600005 | 0,254700005 | 0,266000003 | 0,264099985 | 0,253600001 | 0,251800001 |
| 80 | 0,254299998 | 0,256300002 | 0,275099993 | | 0,270700008 | 0,2597 | 0,265399992 | 0,268700004 | 0,267800003 | 0,257699996 | 0,255499989 | 0,269600004 | 0,2667 | 0,256999999 | 0,253399998 |
| 100 | 0,255100012 | 0,255899996 | 0,281599998 | | 0,276300013 | 0,263200015 | 0,268099993 | 0,274699986 | 0,272000015 | 0,261099994 | 0,257999986 | 0,274899989 | 0,270999998 | 0,2579 | 0,256199986 |
| 120 | 0,257400006 | 0,257099986 | 0,289600015 | | 0,282400012 | 0,26699999 | 0,272599995 | 0,280099988 | 0,278899997 | 0,263999999 | 0,261700004 | 0,280600011 | 0,27700001 | 0,262899995 | 0,260699987 |
| 140 | 0,254700005 | 0,254999995 | 0,294099987 | | 0,286199987 | 0,268700004 | 0,275700003 | 0,282700002 | 0,282000005 | 0,266799986 | 0,263099998 | 0,282599986 | 0,280400008 | 0,264999986 | 0,263000011 |
| 160 | 0,254200011 | 0,256399989 | 0,300999999 | | 0,291900009 | 0,273200005 | 0,279599994 | 0,288300008 | 0,287299991 | 0,269499987 | 0,26699999 | 0,288599998 | 0,286199987 | 0,268299997 | 0,264699996 |
| 180 | 0,253699988 | 0,255499989 | 0,307799995 | | 0,296299994 | 0,2764 | 0,283699989 | 0,293000013 | 0,291700006 | 0,272500008 | 0,26910001 | 0,293300003 | 0,291399986 | 0,271499991 | 0,2676 |
| 200 | 0,254799992 | 0,253500015 | 0,313899994 | | 0,30219999 | 0,279300004 | 0,287699997 | 0,296900004 | 0,295399994 | 0,274899989 | 0,271699995 | 0,297600001 | 0,295899987 | 0,274500012 | 0,270799994 |
| 220 | 0,254299998 | 0,253899992 | 0,321099997 | | 0,306899995 | 0,282900006 | 0,291999996 | 0,3028 | 0,301200002 | 0,277500004 | 0,274899989 | 0,300999999 | 0,300500005 | 0,277799994 | 0,272899985 |
| 240 | 0,253600001 | 0,252900004 | 0,325899988 | | 0,312900007 | 0,28580001 | 0,29550001 | 0,307000011 | 0,304399997 | 0,280800015 | 0,27669999 | 0,306600004 | 0,30430001 | 0,279500008 | 0,275200009 |
| 260 | 0,253500015 | 0,253399998 | 0,332100004 | | 0,317200005 | 0,290699989 | 0,298200011 | 0,312099993 | 0,308999985 | 0,282999992 | 0,277500004 | 0,311100006 | 0,308999985 | 0,280600011 | 0,277799994 |
| 280 | 0,254500002 | 0,253699988 | 0,339300007 | | 0,323599994 | 0,292499989 | 0,302599996 | 0,316500008 | 0,313699991 | 0,28580001 | 0,280299991 | 0,317000002 | 0,31279999 | 0,285699993 | 0,279799998 |
| 300 | 0,254000008 | 0,254000008 | 0,345400006 | | 0,329100013 | 0,296499997 | 0,307000011 | 0,32190001 | 0,319700003 | 0,287900001 | 0,284999996 | 0,321500003 | 0,319499999 | 0,287499994 | 0,283499986 |
| 320 | 0,253300011 | 0,253899992 | 0,352899998 | | 0,333900005 | 0,301299989 | 0,311500013 | 0,327899992 | 0,323900014 | 0,291599989 | 0,28580001 | 0,326799989 | 0,323700011 | 0,290499985 | 0,285699993 |
| 340 | 0,253600001 | 0,255199999 | 0,359100014 | | 0,339899987 | 0,303499997 | 0,315499991 | 0,332500011 | 0,327100009 | 0,294 | 0,289299995 | 0,33129999 | 0,328000009 | 0,293500006 | 0,287800014 |
| 360 | 0,254000008 | 0,253699988 | 0,364600003 | | 0,345899999 | 0,307900012 | 0,318699986 | 0,336600006 | 0,333000004 | 0,297300011 | 0,291500002 | 0,334500015 | 0,332300007 | 0,296299994 | 0,289999992 |
| slope | -5,89781E-06 | -1,07224E-05 | 0,000317513 | | 0,00025437 | 0,000162337 | 0,00018483 | 0,000232921 | 0,000224587 | 0,00013694 | 0,000125392 | 0,000230975 | 0,000229881 | 0,000142234 | 0,00013064 |
| slope w/o |  |  | 0,000325823 | | 0,000262681 | 0,000170648 | 0,00019314 | 0,000241231 | 0,000232897 | 0,00014525 | 0,000133702 | 0,000239285 | 0,000238191 | 0,000150544 | 0,00013895 |
| blank | |  |  |  | 0,000294252 |  | 0,000181894 |  | 0,000237064 |  | 0,000139476 |  | 0,000238738 |  | 0,000144747 |
|  | |  |  |  | 0,017655109 |  | 0,010913622 |  | 0,01422384 |  | 0,008368577 |  | 0,014324304 |  | 0,00868483 |
|  | |  |  |  | 2,635090904 |  | 1,628898853 |  | 2,122961185 |  | 1,249041273 |  | 2,137955832 |  | 1,296243254 |
|  | |  |  |  | 43,91818174 |  | 27,14831422 |  | 35,38268641 |  | 20,81735455 |  | 35,6325972 |  | 21,60405423 |
|  | |  | **min -1** |  | 43,91818174 |  | 27,14831422 |  | 35,38268641 |  | 20,81735455 |  | 35,6325972 |  | 21,60405423 |
|  | | **5 min** |  |  |  |  |  | 30 |  |  |  |  |  |  |  |
| **PTPN22 wt control** | | 43,91818174 |  | 1 | 100 |  |  | 76,05790021 |  | 36,29645354 | 40,2877268 | 42,29972198 |  |  |  |
| **PTPN22 mut control** | | 27,14831422 |  | 1 | 100 |  |  | 4,469147798 |  | 38,83254163 | 35,511682 | 33,24784231 |  |  |  |
| **PTPN22 wt 25uM H2O2** | | 35,38268641 |  | 0,805650075 | 80,5650075 |  |  | 97,26724967 |  | 34,31834659 | 39,67431092 | **31,9840417** |  |  |  |
| **PTPN22 mut 25uM H2O2** | | 20,81735455 |  | 0,766801002 | 76,68010021 |  |  | 31,22145889 |  | 36,95069113 | 34,9051965 | 31,260104 |  |  |  |
| **PTPN22 wt 50uM H2O2** | | 35,6325972 |  | 0,811340447 | 81,13404469 |  |  |  |  | 31,57471215 | 36,20904822 | **33,80080718** |  |  |  |
| **PTPN22 mut 50uM H2O2** | | 21,60405423 #DIV/0!  #DIV/0! |  | 0,795778849 | 79,57788486 |  |  |  |  | 34,54707642 | 33,41881996 | **32,142307** |  |  |  |

**PTPN22 reduced control PTPN22 3.4uM H2O2 PTPN22 6.3uM H2O2 PTPN22 12.5uM H2O2 PTPN22 25uM H2O2 PTPN22 50uM H2O2**

Protocol Layout:

- 20mM Hepes Buffer 0,1mM DTPA, pH 7.4, 10min
- Wash 1ml spinn collumn slurry 5x with Hepes Buffer
- Spinn 1000 rpm for 2 min, apply 50μl PTP1B from Freezer (recombinant HishPTP1B in 20mM Hepes, 1mM TCEP), ( desalt 1x)
- Measure PTP1B (reduced PTP) and protein concentration with Bradford -> (0,585/1,16) X5 -> - 0,5 μg/μl
- Prepare 16.7mM (15mM end concentration) pNPP (Sigma) substrate sollution Mw 371

**PTP1B activity catalytic domain exposed to 50μM H2O2 and 25mM Bicarbonat and TrxR1, Trx, Prx2**

- prepare 20μl PTP1B, 600nM
- add 20μl from each condition in duplicates to 96-well plate
- Add 180μl substrate solution


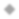

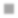

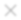

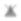

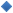

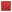

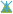

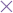

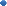

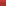

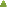

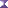

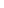


50

45

40

35

30

25

20

15

PTPN22 wt control #REF!

PTPN22 mut control

PTPN22 wt 25uM H2O2 PTPN22 mut 25uM H2O2

10

5

0

0

0,2 0,4 0,6 0,8

1

1,2

Application: Tecan i-control Tecan i-control , 2.0.10.0

Device: infinite 200Pro Serial number: 1307001123 Serial number of connected stacker: Firmware: V_3.40_01/15_Infinite (Dec 23 201 MAI, V_3.40_01/15_Infinite (Dec 23 2014/12.45.11)

Date: #########

Time: 13:55:24

System MTC-MU059-S

User MTC-MU059-S\fretho

Plate Greiner 96 Flat Bottom Transparent Polystyrene Cat. No.: 655101/655161/655192 [GRE96ft.pdfx] Plate-ID (Stacker)

List of actions in this measurement script: Kinetic

Absorbance

Label: Label1

Kinetic Measurement

Kinetic duration 00:06:00

Interval Time 00:00:20

Measurement Wavelength 405 nm

Bandwidth 10 nm

Number of Flashes 5

Settle Time 0 ms

Part of Plate C1-C12; D1-D2

Start Time: 2020 05-19 13:55:26

| Cycle Nr. | 1 | 2 | 3 | 4 | 5 | 6 | 7 | 8 | 9 | 10 | 11 | 12 | 13 | 14 | 15 | 16 | 17 | 18 | 19 |
| --- | --- | --- | --- | --- | --- | --- | --- | --- | --- | --- | --- | --- | --- | --- | --- | --- | --- | --- | --- |
| Time [s] | 0 | 20 | 40 | 60 | 80 | 100 | 120 | 140 | 160 | 180 | 200 | 220 | 240 | 260 | 280 | 300 | 320 | 340 | 360 |
| Temp. [°C] | 24,6 | 24,6 | 24,8 | 24,6 | 24,8 | 24,6 | 24,8 | 24,6 | 24,8 | 24,6 | 24,7 | 24,7 | 25,1 | 24,8 | 25 | 24,9 | 24,9 | 24,7 | 25,2 |
| C1 | 0,2549 | 0,2571 | 0,2648 | 0,2686 | 0,2751 | 0,2816 | 0,2896 | 0,2941 | 0,301 | 0,3078 | 0,3139 | 0,3211 | 0,3259 | 0,3321 | 0,3393 | 0,3454 | 0,3529 | 0,3591 | 0,3646 |
| C2 | 0,258 | 0,255 | 0,2674 | 0,2723 | 0,2707 | 0,2763 | 0,2824 | 0,2862 | 0,2919 | 0,2963 | 0,3022 | 0,3069 | 0,3129 | 0,3172 | 0,3236 | 0,3291 | 0,3339 | 0,3399 | 0,3459 |
| C3 | 0,2524 | 0,2511 | 0,2599 | 0,2571 | 0,2597 | 0,2632 | 0,267 | 0,2687 | 0,2732 | 0,2764 | 0,2793 | 0,2829 | 0,2858 | 0,2907 | 0,2925 | 0,2965 | 0,3013 | 0,3035 | 0,3079 |
| C4 | 0,2507 | 0,2618 | 0,2578 | 0,2606 | 0,2654 | 0,2681 | 0,2726 | 0,2757 | 0,2796 | 0,2837 | 0,2877 | 0,292 | 0,2955 | 0,2982 | 0,3026 | 0,307 | 0,3115 | 0,3155 | 0,3187 |
| C5 | 0,2576 | 0,2555 | 0,2675 | 0,2659 | 0,2687 | 0,2747 | 0,2801 | 0,2827 | 0,2883 | 0,293 | 0,2969 | 0,3028 | 0,307 | 0,3121 | 0,3165 | 0,3219 | 0,3279 | 0,3325 | 0,3366 |
| C6 | 0,2557 | 0,2558 | 0,2624 | 0,2658 | 0,2678 | 0,272 | 0,2789 | 0,282 | 0,2873 | 0,2917 | 0,2954 | 0,3012 | 0,3044 | 0,309 | 0,3137 | 0,3197 | 0,3239 | 0,3271 | 0,333 |
| C7 | 0,2515 | 0,2503 | 0,2538 | 0,2556 | 0,2577 | 0,2611 | 0,264 | 0,2668 | 0,2695 | 0,2725 | 0,2749 | 0,2775 | 0,2808 | 0,283 | 0,2858 | 0,2879 | 0,2916 | 0,294 | 0,2973 |
| C8 | 0,25 | 0,2474 | 0,2529 | 0,2547 | 0,2555 | 0,258 | 0,2617 | 0,2631 | 0,267 | 0,2691 | 0,2717 | 0,2749 | 0,2767 | 0,2775 | 0,2803 | 0,285 | 0,2858 | 0,2893 | 0,2915 |
| C9 | 0,2572 | 0,2572 | 0,2615 | 0,266 | 0,2696 | 0,2749 | 0,2806 | 0,2826 | 0,2886 | 0,2933 | 0,2976 | 0,301 | 0,3066 | 0,3111 | 0,317 | 0,3215 | 0,3268 | 0,3313 | 0,3345 |
| C10 | 0,2549 | 0,2551 | 0,26 | 0,2641 | 0,2667 | 0,271 | 0,277 | 0,2804 | 0,2862 | 0,2914 | 0,2959 | 0,3005 | 0,3043 | 0,309 | 0,3128 | 0,3195 | 0,3237 | 0,328 | 0,3323 |
| C11 | 0,2461 | 0,2472 | 0,2516 | 0,2536 | 0,257 | 0,2579 | 0,2629 | 0,265 | 0,2683 | 0,2715 | 0,2745 | 0,2778 | 0,2795 | 0,2806 | 0,2857 | 0,2875 | 0,2905 | 0,2935 | 0,2963 |
| C12 | 0,2436 | 0,2452 | 0,2493 | 0,2518 | 0,2534 | 0,2562 | 0,2607 | 0,263 | 0,2647 | 0,2676 | 0,2708 | 0,2729 | 0,2752 | 0,2778 | 0,2798 | 0,2835 | 0,2857 | 0,2878 | 0,29 |
| D1 | 0,2561 | 0,2543 | 0,2563 | 0,2555 | 0,2543 | 0,2551 | 0,2574 | 0,2547 | 0,2542 | 0,2537 | 0,2548 | 0,2543 | 0,2536 | 0,2535 | 0,2545 | 0,254 | 0,2533 | 0,2536 | 0,254 |
| D2 | 0,2576 | 0,2567 | 0,2569 | 0,257 | 0,2563 | 0,2559 | 0,2571 | 0,255 | 0,2564 | 0,2555 | 0,2535 | 0,2539 | 0,2529 | 0,2534 | 0,2537 | 0,254 | 0,2539 | 0,2552 | 0,2537 |

**170330**

**Time (sec) Blank Blank**

**PTPN22 wt control PTPN22 wt control PTPN22 mut control PTPN22 mut control PTPN22 wt 25uM H2O2 PTPN22 wt 25uM H2O2 PTPN22 mut 25uM H2O2 PTPN22 mut 25uM H2O2 PTPN22 wt 50uM H2O PTPN22 wt 50uM H2O PTPN22 mut 50uM H2O2 PTPN22 mut 50uM H2O**

| 20 | 0,2542 | 0,253999996 | 0,259399992 | | 0,252700013 | 0,247099996 | 0,249200001 | 0,253199995 | 0,252700001 | 0,244800001 | 0,248699993 | 0,246999994 | 0,25 | 0,244100004 | 0,244000003 |
| --- | --- | --- | --- | --- | --- | --- | --- | --- | --- | --- | --- | --- | --- | --- | --- |
| 40 | 0,252900007 | 0,254400003 | 0,262299997 | | 0,258999985 | 0,249200001 | 0,251800001 | 0,254299998 | 0,255299985 | 0,247700006 | 0,2491 | 0,247899994 | 0,252900004 | 0,245700002 | 0,245800003 |
| 60 | 0,255300003 | 0,254600006 | 0,269200009 | | 0,271500003 | 0,253399998 | 0,254200011 | 0,260500014 | 0,259499997 | 0,250200003 | 0,252099991 | 0,251899987 | 0,256300002 | 0,246800005 | 0,248300001 |
| 80 | 0,253099996 | 0,2551 | 0,275500011 | | 0,265699995 | 0,255499989 | 0,257200003 | 0,264600009 | 0,263900012 | 0,250999987 | 0,252900004 | 0,254599988 | 0,2597 | 0,246900007 | 0,248799995 |
| 100 | 0,255500007 | 0,254899997 | 0,282899988 | | 0,272299987 | 0,259499997 | 0,260399997 | 0,269300014 | 0,268700004 | 0,254299998 | 0,256000012 | 0,2579 | 0,261799991 | 0,248600006 | 0,25060001 |
| 120 | 0,253799993 | 0,255300003 | 0,288200003 | | 0,277899992 | 0,262400001 | 0,263799995 | 0,273299992 | 0,272500008 | 0,256199986 | 0,258300006 | 0,260600001 | 0,264899999 | 0,2491 | 0,251199991 |
| 140 | 0,254300001 | 0,254799995 | 0,294999999 | | 0,281800014 | 0,266200006 | 0,267100006 | 0,277200013 | 0,277999997 | 0,25909999 | 0,260500014 | 0,264699996 | 0,269300014 | 0,2509 | 0,253100008 |
| 160 | 0,255300003 | 0,256100002 | **0,302999985** | | 0,287500006 | 0,272399992 | 0,271800011 | 0,283600003 | 0,283300012 | 0,262100011 | 0,264299989 | 0,267500013 | 0,272599995 | 0,253699988 | 0,255299985 |
| 180 | 0,253400001 | 0,254699993 | 0,3083 | | 0,29040001 | 0,273299992 | 0,275299996 | 0,287299991 | 0,285899997 | 0,264699996 | 0,266000003 | 0,270599991 | 0,275299996 | 0,254000008 | 0,256500006 |
| 200 | 0,255500007 | 0,255599993 | 0,316600007 | | 0,297799987 | 0,279700011 | 0,279500008 | 0,292400002 | 0,293300003 | 0,267199993 | 0,270599991 | 0,275400013 | 0,279300004 | 0,255899996 | 0,259200007 |
| 220 | 0,253199998 | 0,2551 | 0,321800005 | | 0,301000011 | 0,281300008 | 0,282599986 | 0,29519999 | 0,296200007 | 0,269800007 | 0,271699995 | 0,2773 | 0,282499999 | 0,257299989 | 0,258599997 |
| 240 | 0,254300001 | 0,256300005 | 0,329700005 | | 0,3083 | 0,286599994 | 0,286500007 | 0,30219999 | 0,30250001 | 0,273999989 | 0,274800003 | 0,281800002 | 0,287999988 | 0,2588 | 0,262199998 |
| 260 | 0,2542 | 0,255899999 | 0,336700004 | | 0,313799989 | 0,289600015 | 0,288800001 | 0,305799991 | 0,307200015 | 0,274699986 | 0,276600003 | 0,28490001 | 0,290199995 | 0,259499997 | 0,262600005 |
| 280 | 0,253999996 | 0,254999998 | 0,34320001 | | 0,317500007 | 0,292199999 | 0,292600006 | 0,310600013 | 0,310400009 | 0,27700001 | 0,278699994 | 0,287299991 | 0,292100012 | 0,261599988 | 0,264899999 |
| 300 | 0,253899994 | 0,254099998 | 0,350099993 | | 0,323600006 | 0,296000004 | 0,297100008 | 0,316599995 | 0,317600012 | 0,280200005 | 0,282400012 | 0,290300012 | 0,29550001 | 0,262899995 | 0,266200006 |
| 320 | 0,256400007 | 0,256100002 | 0,357700002 | | 0,329200011 | 0,299299985 | 0,300300002 | 0,321500003 | 0,32100001 | 0,282499999 | 0,284700006 | 0,29429999 | 0,300099999 | 0,265100002 | 0,2676 |
| 340 | 0,254500005 | 0,254500005 | 0,363599998 | | 0,333599997 | 0,301699996 | 0,303299993 | 0,324800014 | 0,324600011 | 0,284099996 | 0,284799993 | 0,296999991 | 0,302100003 | 0,265199989 | 0,26730001 |
| 360 | 0,254799995 | 0,254899997 | 0,36929999 | | 0,338900012 | 0,305299997 | 0,306600004 | 0,32949999 | 0,329899997 | 0,286599994 | 0,288100004 | 0,299100012 | 0,305700004 | 0,266400009 | 0,270200014 |
| slope | 2,18781E-06 | 1,84726E-06 | 0,000334329 | | 0,000248189 | 0,000177446 | 0,000174659 | 0,000231357 | 0,000233689 | 0,00012547 | 0,000123029 | 0,000161017 | 0,000166414 | 6,84984E-05 | 7,62023E-05 |
| slope w/o |  |  | 0,000332312 | | 0,000246171 | 0,000175428 | 0,000172642 | 0,00022934 | 0,000231672 | 0,000123452 | 0,000121011 | 0,000158999 | 0,000164396 | 6,64809E-05 | 7,41848E-05 |
| blank |  | |  |  | 0,000289242 |  | 0,000174035 |  | 0,000230506 |  | 0,000122232 |  | 0,000161698 |  | 7,03328E-05 |
|  |  | |  |  | 0,01735449 |  | 0,010442106 |  | 0,013830341 |  | 0,007333901 |  | 0,009701859 |  | 0,004219969 |
|  |  | |  |  | 2,590222407 |  | 1,558523211 |  | 2,064230049 |  | 1,094612138 |  | 1,448038644 |  | 0,629846142 |
|  |  | |  |  | 43,17037345 |  | 25,97538686 |  | 34,40383415 |  | 18,24353563 |  | 24,13397741 |  | 10,4974357 |
|  |  | | **min -1** |  | 43,17037345 |  | 25,97538686 |  | 34,40383415 |  | 18,24353563 |  | 24,13397741 |  | 10,4974357 |
|  | **15 min PTPN22 wt control** 43,17037345 | |  | 1 | 100 |  |  |  |  |  |  |  |  |  |  |
|  | **PTPN22 mut control** 25,97538686 | |  | 1 | 100 |  |  |  |  |  |  |  |  |  |  |
|  | **PTPN22 wt 25uM H2O2** 34,40383415 | |  | 0,796931585 | 79,69315854 |  |  |  |  |  |  |  |  |  |  |
|  | **PTPN22 mut 25uM H2O2** 18,24353563 | |  | 0,702339323 | 70,23393232 |  |  |  |  |  |  |  |  |  |  |
|  | **PTPN22 wt 50uM H2O2** 24,13397741 | |  | 0,559040274 | 55,90402741 |  |  |  |  |  |  |  |  |  |  |
|  | **PTPN22 mut 50uM H2O2** 10,4974357 | |  | 0,404130101 | 40,41301005 |  |  |  |  |  |  |  |  |  |  |

#DIV/0! #DIV/0!

Protocol Layout:

- 20mM Hepes Buffer 0,1mM DTPA, pH 7.4, 10min
- Wash 1ml spinn collumn slurry 5x with Hepes Buffer
- Spinn 1000 rpm for 2 min, apply 50μl PTP1B from Freezer (recombinant HishPTP1B in 20mM Hepes, 1mM TCEP), ( desalt 1x)
- Measure PTP1B (reduced PTP) and protein concentration with Bradford -> (0,585/1,16) X5 -> - 0,5 μg/μl
- Prepare 16.7mM (15mM end concentration) pNPP (Sigma) substrate sollution Mw 371

**PTP1B activity catalytic domain exposed to 50μM H2O2 and 25mM Bicarbonat and TrxR1, Trx, Prx2**

- prepare 20μl PTP1B, 600nM
- add 20μl from each condition in duplicates to 96-well plate
- Add 180μl substrate solution


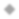

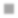

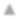

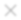

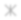

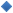

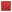

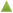

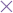

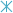

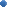

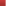

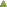

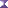

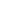


50

45

40

35

30

25

20

15

PTPN22 wt control #REF!

PTPN22 mut control PTPN22 wt 25uM H2O2

PTPN22 mut 25uM H2O2

10

5

0

0

0,2 0,4 0,6 0,8

1

1,2

Application: Tecan i-control Tecan i-control , 2.0.10.0

Device: infinite 200Pro Serial number: 1307001123 Serial number of connected stacker: Firmware: V_3.40_01/15_Infinite (Dec 23 201 MAI, V_3.40_01/15_Infinite (Dec 23 2014/12.45.11)

Date: #########

Time: 14:04:58

System MTC-MU059-S

User MTC-MU059-S\fretho

Plate Greiner 96 Flat Bottom Transparent Polystyrene Cat. No.: 655101/655161/655192 [GRE96ft.pdfx] Plate-ID (Stacker)

List of actions in this measurement script: Kinetic

Absorbance

Label: Label1

Kinetic Measurement

Kinetic duration 00:06:00

Interval Time 00:00:20

Measurement Wavelength 405 nm

Bandwidth 10 nm

Number of Flashes 5

Settle Time 0 ms

Part of Plate C1-C12; D1-D2

Start Time: 2020 05-19 14:05:01

| 0,2594 | 0,2594 | 0,2623 | 0,2692 | 0,2755 | 0,2829 | 0,2882 | 0,295 | 0,303 | 0,3083 | 0,3166 | 0,3218 | 0,3297 | 0,3367 | 0,3432 | 0,3501 | 0,3577 | 0,3636 | 0,3693 |
| --- | --- | --- | --- | --- | --- | --- | --- | --- | --- | --- | --- | --- | --- | --- | --- | --- | --- | --- |
| 0,254 | 0,2527 | 0,259 | 0,2715 | 0,2657 | 0,2723 | 0,2779 | 0,2818 | 0,2875 | 0,2904 | 0,2978 | 0,301 | 0,3083 | 0,3138 | 0,3175 | 0,3236 | 0,3292 | 0,3336 | 0,3389 |

| Cycle Nr. | 1 | 2 | 3 | 4 | 5 | 6 | 7 | 8 | 9 | 10 | 11 | 12 | 13 | 14 | 15 | 16 | 17 | 18 | 19 |
| --- | --- | --- | --- | --- | --- | --- | --- | --- | --- | --- | --- | --- | --- | --- | --- | --- | --- | --- | --- |
| Time [s] | 0 | 20 | 40 | 60 | 80 | 100 | 120 | 140 | 160 | 180 | 200 | 220 | 240 | 260 | 280 | 300 | 320 | 340 | 360 |
| Temp. [°C] | 25,1 | 25 | 25,1 | 25,1 | 25,1 | 25,1 | 25,1 | 25,3 | 25,3 | 25 | 25,3 | 25,2 | 25,1 | 25,2 | 25,2 | 25,2 | 25,2 | 25,3 | 25,2 |
| C1 | 0,3094 | 0,3094 | 0,3123 | 0,3192 | 0,3255 | 0,3329 | 0,3382 | 0,345 | 0,353 | 0,3583 | 0,3666 | 0,3718 | 0,3797 | 0,3867 | 0,3932 | 0,4001 | 0,4077 | 0,4136 | 0,4193 |
| C2 | 0,304 | 0,3027 | 0,309 | 0,3215 | 0,3157 | 0,3223 | 0,3279 | 0,3318 | 0,3375 | 0,3404 | 0,3478 | 0,351 | 0,3583 | 0,3638 | 0,3675 | 0,3736 | 0,3792 | 0,3836 | 0,3889 |
| C3 | 0,2482 | 0,2471 | 0,2492 | 0,2534 | 0,2555 | 0,2595 | 0,2624 | 0,2662 | 0,2724 | 0,2733 | 0,2797 | 0,2813 | 0,2866 | 0,2896 | 0,2922 | 0,296 | 0,2993 | 0,3017 | 0,3053 |
| C4 | 0,25 | 0,2492 | 0,2518 | 0,2542 | 0,2572 | 0,2604 | 0,2638 | 0,2671 | 0,2718 | 0,2753 | 0,2795 | 0,2826 | 0,2865 | 0,2888 | 0,2926 | 0,2971 | 0,3003 | 0,3033 | 0,3066 |
| C5 | 0,2525 | 0,2532 | 0,2543 | 0,2605 | 0,2646 | 0,2693 | 0,2733 | 0,2772 | 0,2836 | 0,2873 | 0,2924 | 0,2952 | 0,3022 | 0,3058 | 0,3106 | 0,3166 | 0,3215 | 0,3248 | 0,3295 |
| C6 | 0,2539 | 0,2527 | 0,2553 | 0,2595 | 0,2639 | 0,2687 | 0,2725 | 0,278 | 0,2833 | 0,2859 | 0,2933 | 0,2962 | 0,3025 | 0,3072 | 0,3104 | 0,3176 | 0,321 | 0,3246 | 0,3299 |
| C7 | 0,2461 | 0,2448 | 0,2477 | 0,2502 | 0,251 | 0,2543 | 0,2562 | 0,2591 | 0,2621 | 0,2647 | 0,2672 | 0,2698 | 0,274 | 0,2747 | 0,277 | 0,2802 | 0,2825 | 0,2841 | 0,2866 |
| C8 | 0,2487 | 0,2487 | 0,2491 | 0,2521 | 0,2529 | 0,256 | 0,2583 | 0,2605 | 0,2643 | 0,266 | 0,2706 | 0,2717 | 0,2748 | 0,2766 | 0,2787 | 0,2824 | 0,2847 | 0,2848 | 0,2881 |
| C9 | 0,247 | 0,247 | 0,2479 | 0,2519 | 0,2546 | 0,2579 | 0,2606 | 0,2647 | 0,2675 | 0,2706 | 0,2754 | 0,2773 | 0,2818 | 0,2849 | 0,2873 | 0,2903 | 0,2943 | 0,297 | 0,2991 |
| C10 | 0,2519 | 0,25 | 0,2529 | 0,2563 | 0,2597 | 0,2618 | 0,2649 | 0,2693 | 0,2726 | 0,2753 | 0,2793 | 0,2825 | 0,288 | 0,2902 | 0,2921 | 0,2955 | 0,3001 | 0,3021 | 0,3057 |
| C11 | 0,2495 | 0,2441 | 0,2457 | 0,2468 | 0,2469 | 0,2486 | 0,2491 | 0,2509 | 0,2537 | 0,254 | 0,2559 | 0,2573 | 0,2588 | 0,2595 | 0,2616 | 0,2629 | 0,2651 | 0,2652 | 0,2664 |
| C12 | 0,2489 | 0,244 | 0,2458 | 0,2483 | 0,2488 | 0,2506 | 0,2512 | 0,2531 | 0,2553 | 0,2565 | 0,2592 | 0,2586 | 0,2622 | 0,2626 | 0,2649 | 0,2662 | 0,2676 | 0,2673 | 0,2702 |
| D1 | 0,2043 | 0,2042 | 0,2029 | 0,2053 | 0,2031 | 0,2055 | 0,2038 | 0,2043 | 0,2053 | 0,2034 | 0,2055 | 0,2032 | 0,2043 | 0,2042 | 0,204 | 0,2039 | 0,2064 | 0,2045 | 0,2048 |
| D2 | 0,2051 | 0,204 | 0,2044 | 0,2046 | 0,2051 | 0,2049 | 0,2053 | 0,2048 | 0,2061 | 0,2047 | 0,2056 | 0,2051 | 0,2063 | 0,2059 | 0,205 | 0,2041 | 0,2061 | 0,2045 | 0,2049 |

| 0,2543 | 0,2542 | 0,2529 | 0,2553 | 0,2531 | 0,2555 | 0,2538 | 0,2543 | 0,2553 | 0,2534 | 0,2555 | 0,2532 | 0,2543 | 0,2542 | 0,254 | 0,2539 | 0,2564 | 0,2545 | 0,2548 |
| --- | --- | --- | --- | --- | --- | --- | --- | --- | --- | --- | --- | --- | --- | --- | --- | --- | --- | --- |
| 0,2551 | 0,254 | 0,2544 | 0,2546 | 0,2551 | 0,2549 | 0,2553 | 0,2548 | 0,2561 | 0,2547 | 0,2556 | 0,2551 | 0,2563 | 0,2559 | 0,255 | 0,2541 | 0,2561 | 0,2545 | 0,2549 |

Application: Tecan i-control Tecan i-control , 2.0.10.0

Device: infinite 200Pro Serial number: 1307001123 Serial number of connected stacker: Firmware: V_3.40_01/15_Infinite (Dec 23 201 MAI, V_3.40_01/15_Infinite (Dec 23 2014/12.45.11)

Date: #########

Time: 14:20:08

System MTC-MU059-S

User MTC-MU059-S\fretho

Plate Greiner 96 Flat Bottom Transparent Polystyrene Cat. No.: 655101/655161/655192 [GRE96ft.pdfx] Plate-ID (Stacker)

List of actions in this measurement script: Kinetic

Absorbance

Label: Label1

Kinetic Measurement

Kinetic duration 00:06:00

Interval Time 00:00:20

Measurement Wavelength 405 nm

Bandwidth 10 nm

Number of Flashes 5

Settle Time 0 ms

Part of Plate C1-C12; D1-D2

Start Time: 2020 05-19 14:20:11

| Cycle Nr. | 1 | 2 | 3 | 4 | 5 | 6 | 7 | 8 | 9 | 10 | 11 | 12 | 13 | 14 | 15 | 16 | 17 | 18 | 19 |
| --- | --- | --- | --- | --- | --- | --- | --- | --- | --- | --- | --- | --- | --- | --- | --- | --- | --- | --- | --- |
| Time [s] | 0 | 20 | 40 | 60 | 80 | 100 | 120 | 140 | 160 | 180 | 200 | 220 | 240 | 260 | 280 | 300 | 320 | 340 | 360 |
| Temp. [°C] | 25,2 | 25,3 | 25,4 | 25,2 | 25,3 | 25,3 | 25,3 | 25,5 | 25,3 | 25,4 | 25,3 | 25,3 | 25,4 | 25,4 | 25,5 | 25,4 | 25,4 | 25,3 | 25,5 |
| C1 | 0,2566 | 0,2598 | 0,2692 | 0,2715 | 0,2794 | 0,2852 | 0,2922 | 0,2972 | 0,3036 | 0,3091 | 0,3169 | 0,3222 | 0,3286 | 0,3324 | 0,3391 | 0,3447 | 0,3512 | 0,3577 | 0,3637 |
| C2 | 0,251 | 0,2567 | 0,2612 | 0,264 | 0,2693 | 0,2741 | 0,2801 | 0,2849 | 0,2892 | 0,2945 | 0,3015 | 0,3071 | 0,313 | 0,3173 | 0,3224 | 0,3289 | 0,3346 | 0,3374 | 0,3453 |
| C3 | 0,2435 | 0,2486 | 0,2508 | 0,2528 | 0,2559 | 0,2596 | 0,2632 | 0,266 | 0,2689 | 0,2717 | 0,2771 | 0,2794 | 0,2843 | 0,2869 | 0,2887 | 0,2922 | 0,2961 | 0,2984 | 0,3029 |
| C4 | 0,2482 | 0,2516 | 0,2535 | 0,2584 | 0,2604 | 0,2644 | 0,2682 | 0,272 | 0,275 | 0,2781 | 0,2822 | 0,2856 | 0,289 | 0,291 | 0,2946 | 0,2982 | 0,3014 | 0,3035 | 0,3059 |
| C5 | 0,2463 | 0,2465 | 0,2512 | 0,2535 | 0,258 | 0,2614 | 0,2647 | 0,2686 | 0,2714 | 0,2741 | 0,2792 | 0,2828 | 0,2866 | 0,2901 | 0,2938 | 0,2969 | 0,3005 | 0,3036 | 0,3073 |
| C6 | 0,2447 | 0,2476 | 0,2494 | 0,2528 | 0,2565 | 0,2582 | 0,2632 | 0,2659 | 0,2685 | 0,2714 | 0,2755 | 0,2794 | 0,2822 | 0,2848 | 0,2876 | 0,2904 | 0,2948 | 0,2968 | 0,3002 |
| C7 | 0,2431 | 0,2433 | 0,2443 | 0,2462 | 0,2483 | 0,2501 | 0,2523 | 0,2544 | 0,2536 | 0,2543 | 0,2572 | 0,2589 | 0,2609 | 0,2621 | 0,2635 | 0,2642 | 0,2652 | 0,2663 | 0,2668 |
| C8 | 0,2416 | 0,2437 | 0,2455 | 0,2461 | 0,2485 | 0,2492 | 0,2506 | 0,2515 | 0,253 | 0,2541 | 0,257 | 0,2585 | 0,2594 | 0,2596 | 0,2604 | 0,262 | 0,2636 | 0,2644 | 0,2643 |
| C9 | 0,2429 | 0,2436 | 0,2464 | 0,2463 | 0,2488 | 0,252 | 0,2534 | 0,2556 | 0,2564 | 0,2583 | 0,2626 | 0,2623 | 0,2667 | 0,2684 | 0,2689 | 0,2715 | 0,2731 | 0,2745 | 0,2773 |
| C10 | 0,2428 | 0,2485 | 0,2461 | 0,2478 | 0,2491 | 0,2516 | 0,2557 | 0,2627 | 0,2603 | 0,2583 | 0,2608 | 0,2619 | 0,2645 | 0,2659 | 0,2671 | 0,2688 | 0,272 | 0,273 | 0,2749 |
| C11 | 0,2429 | 0,2419 | 0,2438 | 0,2459 | 0,2447 | 0,245 | 0,2446 | 0,2474 | 0,2446 | 0,2454 | 0,2477 | 0,2469 | 0,2485 | 0,2484 | 0,2483 | 0,2478 | 0,2494 | 0,2504 | 0,249 |
| C12 | 0,2412 | 0,2414 | 0,2435 | 0,2431 | 0,2442 | 0,2453 | 0,2462 | 0,2467 | 0,2469 | 0,246 | 0,2489 | 0,2503 | 0,2501 | 0,25 | 0,2499 | 0,2514 | 0,2518 | 0,2507 | 0,252 |
| D1 | 0,2588 | 0,2575 | 0,2577 | 0,258 | 0,2573 | 0,2585 | 0,2588 | 0,2583 | 0,2572 | 0,2562 | 0,2584 | 0,259 | 0,2586 | 0,2573 | 0,2568 | 0,2583 | 0,256 | 0,2547 | 0,2566 |
| D2 | 0,258 | 0,2582 | 0,2575 | 0,2581 | 0,2577 | 0,258 | 0,2581 | 0,2593 | 0,2562 | 0,2551 | 0,2562 | 0,257 | 0,2562 | 0,2555 | 0,2558 | 0,2547 | 0,2557 | 0,256 | 0,2554 |

End Time: 2020 05-19 14:26:19

**170330**

**Time (sec) Blank Blank**

**PTPN22 wt control PTPN22 wt control PTPN22 mut control PTPN22 mut control PTPN22 wt 25uM H2O2 PTPN22 wt 25uM H2O2 PTPN22 mut 25uM H2O2 PTPN22 mut 25uM H2O2 PTPN22 wt 50uM H2O PTPN22 wt 50uM H2O PTPN22 mut 50uM H2O2 PTPN22 mut 50uM H2O**

| 20 | 0,257499993 | 0,25819999 | 0,259799987 | | 0,256700009 | 0,248600006 | 0,251599997 | 0,2465 | 0,247600004 | 0,243300006 | 0,243699998 | 0,243599996 | 0,248500004 | 0,241899997 | 0,241400003 |
| --- | --- | --- | --- | --- | --- | --- | --- | --- | --- | --- | --- | --- | --- | --- | --- |
| 40 | 0,257699996 | 0,257499993 | 0,269199997 | | 0,261200011 | 0,250800014 | 0,253500015 | 0,251199991 | 0,249400005 | 0,244299993 | 0,245499998 | 0,246399999 | 0,246099994 | 0,243799999 | 0,243499994 |
| 60 | 0,257999986 | 0,258100003 | 0,271499991 | | 0,263999999 | 0,252799988 | 0,258399993 | 0,253500015 | 0,252799988 | 0,246199995 | 0,246099994 | 0,246299997 | 0,247799993 | 0,245900005 | 0,243100002 |
| 80 | 0,257299989 | 0,257699996 | 0,279399991 | | 0,269300014 | 0,255899996 | 0,260399997 | 0,257999986 | 0,256500006 | 0,248300001 | 0,248500004 | 0,248799995 | 0,2491 | 0,2447 | 0,244200006 |
| 100 | 0,25850001 | 0,257999986 | 0,2852 | | 0,274100006 | 0,259600013 | 0,264400005 | 0,261400014 | 0,25819999 | 0,250099987 | 0,249200001 | 0,252000004 | 0,251599997 | 0,245000005 | 0,245299995 |
| 120 | 0,2588 | 0,258100003 | 0,292199999 | | 0,280099988 | 0,263200015 | 0,26820001 | 0,264699996 | 0,263200015 | 0,252299994 | 0,25060001 | 0,253399998 | 0,255699992 | 0,244599998 | 0,246199995 |
| 140 | 0,258300006 | 0,259299994 | 0,297199994 | | 0,28490001 | 0,266000003 | 0,272000015 | 0,268599987 | 0,265899986 | 0,254400015 | 0,25150001 | 0,255600005 | 0,262699991 | 0,247400001 | 0,246700004 |
| 160 | 0,257200003 | 0,256199986 | 0,303600013 | | 0,289200008 | 0,268900007 | 0,275000006 | 0,271400005 | 0,2685 | 0,253600001 | 0,252999991 | 0,256399989 | 0,26030001 | 0,244599998 | 0,246900007 |
| 180 | 0,256199986 | 0,255100012 | 0,309100002 | | 0,294499993 | 0,271699995 | 0,278100014 | 0,274100006 | 0,271400005 | 0,254299998 | 0,254099995 | 0,258300006 | 0,258300006 | 0,245399997 | 0,246000007 |
| 200 | 0,258399993 | 0,256199986 | 0,316900015 | | 0,301499993 | 0,277099997 | 0,282200009 | 0,279199988 | 0,2755 | 0,257200003 | 0,256999999 | 0,262600005 | 0,260800004 | 0,247700006 | 0,248899996 |
| 220 | 0,259000003 | 0,256999999 | 0,3222 | | 0,307099998 | 0,279399991 | 0,285600007 | 0,282799989 | 0,279399991 | 0,258899987 | 0,25850001 | 0,262300014 | 0,261900008 | 0,246900007 | 0,25029999 |
| 240 | 0,258599997 | 0,256199986 | 0,328599989 | | 0,312999994 | 0,284299999 | 0,289000005 | 0,286599994 | 0,282200009 | 0,260899991 | 0,25940001 | 0,2667 | 0,264499992 | 0,248500004 | 0,250099987 |
| 260 | 0,257299989 | 0,255499989 | 0,332399994 | | 0,317299992 | 0,286900014 | 0,291000009 | 0,290100008 | 0,284799993 | 0,262100011 | 0,259600013 | 0,268400013 | 0,265899986 | 0,248400003 | 0,25 |
| 280 | 0,256799996 | 0,255800009 | 0,339100003 | | 0,322400004 | 0,288700014 | 0,29460001 | 0,293799996 | 0,287600011 | 0,263500005 | 0,260399997 | 0,268900007 | 0,267100006 | 0,248300001 | 0,249899998 |
| 300 | 0,258300006 | 0,254700005 | 0,344700009 | | 0,328900009 | 0,292199999 | 0,298200011 | 0,296900004 | 0,290399998 | 0,264200002 | 0,261999995 | 0,271499991 | 0,26879999 | 0,247799993 | 0,251399994 |
| 320 | 0,256000012 | 0,255699992 | 0,351200014 | | 0,334600002 | 0,296099991 | 0,301400006 | 0,300500005 | 0,294800013 | 0,265199989 | 0,263599992 | 0,273099989 | 0,272000015 | 0,249400005 | 0,251800001 |
| 340 | 0,254700005 | 0,256000012 | 0,35769999 | | 0,337399989 | 0,298400015 | 0,303499997 | 0,303600013 | 0,296799988 | 0,266299993 | 0,264400005 | 0,274500012 | 0,273000002 | 0,250400007 | 0,250699997 |
| 360 | 0,256599993 | 0,255400002 | 0,363700002 | | 0,345299989 | 0,302899987 | 0,305900007 | 0,307300001 | 0,300199986 | 0,266799986 | 0,264299989 | 0,2773 | 0,274899989 | 0,248999998 | 0,252000004 |
| slope | -4,74714E-06 | -9,50978E-06 | 0,000301099 | | 0,000264133 | 0,000162822 | 0,000165129 | 0,00017839 | 0,000157802 | 7,18215E-05 | 6,35913E-05 | 9,94376E-05 | 8,31166E-05 | 1,91073E-05 | 2,98555E-05 |
| slope w/o |  |  | 0,000308228 | | 0,000271262 | 0,000169951 | 0,000172257 | 0,000185519 | 0,00016493 | 7,89499E-05 | 7,07198E-05 | 0,000106566 | 9,02451E-05 | 2,62358E-05 | 3,6984E-05 |
| blank | |  |  |  | 0,000289745 |  | 0,000171104 |  | 0,000175224 |  | 7,48348E-05 |  | 9,84056E-05 |  | 3,16099E-05 |
|  | |  |  |  | 0,017384673 |  | 0,010266252 |  | 0,010513466 |  | 0,004490091 |  | 0,005904334 |  | 0,001896593 |
|  | |  |  |  | 2,594727342 |  | 1,532276456 |  | 1,569174064 |  | 0,670162803 |  | 0,881243811 |  | 0,283073557 |
|  | |  |  |  | 43,24545569 |  | 25,53794093 |  | 26,15290107 |  | 11,16938005 |  | 14,68739685 |  | 4,717892616 |
|  | |  | **min -1** |  | 43,24545569 |  | 25,53794093 |  | 26,15290107 |  | 11,16938005 |  | 14,68739685 |  | 4,717892616 |

| **30 min** |  |  | 30 |
| --- | --- | --- | --- |
| **PTPN22 wt control** 43,24545569 | 1 | 100 |  |
| **PTPN22 mut control** 25,53794093 | 1 | 100 |  |
| **PTPN22 wt 25uM H2O2** 26,15290107 | 0,604754896 | 60,47548963 |  |
| **PTPN22 mut 25uM H2O2** 11,16938005 | 0,437364159 | 43,73641589 |  |
| **PTPN22 wt 50uM H2O2** 14,68739685 | 0,339628676 | 33,96286757 |  |
| **PTPN22 mut 50uM H2O2** 4,717892616 | 0,184740525 | 18,4740525 |  |

#DIV/0! #DIV/0!

Protocol Layout:

- 20mM Hepes Buffer 0,1mM DTPA, pH 7.4, 10min
- Wash 1ml spinn collumn slurry 5x with Hepes Buffer
- Spinn 1000 rpm for 2 min, apply 50μl PTP1B from Freezer (recombinant HishPTP1B in 20mM Hepes, 1mM TCEP), ( desalt 1x)
- Measure PTP1B (reduced PTP) and protein concentration with Bradford -> (0,585/1,16) X5 -> - 0,5 μg/μl
- Prepare 16.7mM (15mM end concentration) pNPP (Sigma) substrate sollution Mw 371

**PTP1B activity catalytic domain exposed to 50μM H2O2 and 25mM Bicarbonat and TrxR1, Trx, Prx2**

- prepare 20μl PTP1B, 600nM
- add 20μl from each condition in duplicates to 96-well plate
- Add 180μl substrate solution


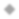

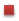

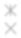

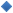

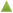

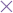

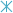

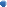

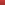

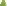

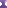

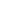


50

45

40

35

30

25

20

15

PTPN22 wt control #REF!

PTPN22 mut control PTPN22 wt 25uM H2O2

PTPN22 mut 25uM H2O2

10

5

0

0

0,2 0,4 0,6 0,8

1

1,2

**PTPN22 wt control PTPN22 wt control PTPN22 mut control PTPN22 mut control PTPN22 wt 25uM H2O2 PTPN22 wt 25uM H2O2 PTPN22 mut 25uM H2O2 PTPN22 mut 25uM H2O2 PTPN22 wt 50uM H2O PTPN22 wt 50uM H2O PTPN22 mut 50uM H2O2 PTPN22 mut 50uM H2O2**

**Time (sec) Blank Blank**

| 20 | 0,259600013 | 0,259600013 | 0,258899987 | | 0,265799999 | 0,252099991 | 0,256500006 | 0,273600012 | 0,256900012 | 0,257499993 | 0,253800005 | 0,260100007 | 0,257999986 | 0,251599997 | 0,252000004 |
| --- | --- | --- | --- | --- | --- | --- | --- | --- | --- | --- | --- | --- | --- | --- | --- |
| 40 | 0,258399993 | 0,258399993 | 0,263300002 | | 0,266099989 | 0,251800001 | 0,254999995 | 0,263000011 | 0,262300014 | 0,256199986 | 0,256199986 | 0,264299989 | 0,263799995 | 0,255100012 | 0,253600001 |
| 60 | 0,258700013 | 0,258700013 | 0,270999998 | | 0,273299992 | 0,256799996 | 0,2597 | 0,268999994 | 0,268000007 | 0,25940001 | 0,25940001 | 0,271400005 | 0,269300014 | 0,257299989 | 0,256900012 |
| 80 | 0,258300006 | 0,258300006 | 0,27610001 | | 0,277700007 | 0,259799987 | 0,261999995 | 0,273900002 | 0,274899989 | 0,262899995 | 0,262400001 | 0,277099997 | 0,273499995 | 0,259499997 | 0,260800004 |
| 100 | 0,2579 | 0,2579 | 0,28459999 | | 0,284399986 | 0,261700004 | 0,264899999 | 0,280099988 | 0,279799998 | 0,266400009 | 0,265599996 | 0,281800002 | 0,278600007 | 0,262899995 | 0,263799995 |
| 120 | 0,25850001 | 0,25850001 | 0,290899992 | | 0,292600006 | 0,2667 | 0,271299988 | 0,287900001 | 0,285100013 | 0,270900011 | 0,269300014 | 0,289200008 | 0,28459999 | 0,265799999 | 0,266799986 |
| 140 | 0,258100003 | 0,258100003 | 0,298400015 | | 0,300500005 | 0,270900011 | 0,274199992 | 0,292400002 | 0,291799992 | 0,274100006 | 0,272500008 | 0,294999987 | 0,290300012 | 0,26879999 | 0,269600004 |
| 160 | 0,258399993 | 0,258399993 | 0,30399999 | | 0,306600004 | 0,273900002 | 0,279199988 | 0,299600005 | 0,296799988 | 0,278600007 | 0,27579999 | 0,299600005 | 0,296299994 | 0,271100014 | 0,273299992 |
| 180 | 0,2579 | 0,2579 | 0,31099999 | | 0,314200014 | 0,278699994 | 0,283199996 | 0,3046 | 0,30309999 | 0,282099992 | 0,279599994 | 0,307399988 | 0,301499993 | 0,274500012 | 0,276800007 |
| 200 | 0,258300006 | 0,258300006 | 0,317600012 | | 0,319299996 | 0,281599998 | 0,28639999 | 0,31099999 | 0,310200006 | 0,283800006 | 0,282599986 | 0,312700003 | 0,307200015 | 0,277999997 | 0,278600007 |
| 220 | 0,259000003 | 0,259000003 | 0,321500003 | | 0,327600002 | 0,285899997 | 0,290499985 | 0,316799998 | 0,316700011 | 0,288700014 | 0,286300004 | 0,318399996 | 0,3125 | 0,280499995 | 0,281699985 |
| 240 | 0,258599997 | 0,258599997 | 0,3301 | | 0,333900005 | 0,289600015 | 0,294999987 | 0,323300004 | 0,32190001 | 0,291799992 | 0,290100008 | 0,324999988 | 0,318300009 | 0,283600003 | 0,283499986 |
| 260 | 0,255699992 | 0,255699992 | 0,334399998 | | 0,339899987 | 0,291399986 | 0,297100008 | 0,327399999 | 0,326499999 | 0,294099987 | 0,291099995 | 0,328500003 | 0,32190001 | 0,285299987 | 0,28549999 |
| 280 | 0,257699996 | 0,257699996 | 0,342799991 | | 0,346799999 | 0,295599997 | 0,301400006 | 0,334600002 | 0,333400011 | 0,299699992 | 0,294200003 | 0,336699992 | 0,328700006 | 0,287999988 | 0,289000005 |
| 300 | 0,256199986 | 0,256199986 | 0,347799987 | | 0,352499992 | 0,298000008 | 0,30340001 | 0,340799987 | 0,337300003 | 0,300000012 | 0,297199994 | 0,340700001 | 0,33160001 | 0,289600015 | 0,290600002 |
| 320 | 0,257600009 | 0,257600009 | 0,354000002 | | 0,360100001 | 0,301400006 | 0,308699995 | 0,345699996 | 0,343600005 | 0,303299993 | 0,300199986 | 0,345499992 | 0,335900009 | 0,292299986 | 0,293000013 |
| 340 | 0,257299989 | 0,257299989 | 0,361499995 | | 0,367199987 | 0,3046 | 0,311199993 | 0,351000011 | 0,348800004 | 0,306800008 | 0,303900003 | 0,351200014 | 0,342299998 | 0,294999987 | 0,295100003 |
| 360 | 0,255899996 | 0,255899996 | 0,368000001 | | 0,371199995 | 0,307500005 | 0,3134 | 0,355699986 | 0,353500009 | 0,309100002 | 0,304800004 | 0,356599987 | 0,346599996 | 0,296200007 | 0,298200011 |
| slope | -6,88859E-06 | -6,88859E-06 | 0,000322049 | | 0,000329582 | 0,000172559 | 0,000183328 | 0,000280093 | 0,000288875 | 0,000164995 | 0,000156254 | 0,000288328 | 0,000262895 | 0,000134721 | 0,000137214 |
| slope w/o |  |  | 0,000328937 | | 0,000336471 | 0,000179448 | 0,000190217 | 0,000286981 | 0,000295764 | 0,000171883 | 0,000163142 | 0,000295217 | 0,000269783 | 0,00014161 | 0,000144102 |
| blank | |  |  |  | 0,000332704 |  | 0,000184832 |  | 0,000291373 |  | 0,000167513 |  | 0,0002825 |  | 0,000142856 |
|  | |  |  |  | 0,019962231 |  | 0,011089941 |  | 0,017482355 |  | 0,010050776 |  | 0,016950003 |  | 0,008571365 |
|  | |  |  |  | 2,979437494 |  | 1,655215133 |  | 2,609306676 |  | 1,500115865 |  | 2,529851166 |  | 1,279308137 |
|  | |  |  |  | 49,65729157 |  | 27,58691888 |  | 43,4884446 |  | 25,00193109 |  | 42,1641861 |  | 21,32180228 |
|  | |  | **min -1** |  | 49,65729157 |  | 27,58691888 |  | 43,4884446 |  | 25,00193109 |  | 42,1641861 |  | 21,32180228 |
|  | | **5 min** |  |  |  |  |  | 30 |  |  |  |  |  |  |  |
| **PTPN22 wt control** | | 49,65729157 |  | 1 | 100 |  |  | 76,05790021 |  | 36,29645354 | 40,2877268 | 42,29972198 |  |  |  |
| **PTPN22 mut control** | | 27,58691888 |  | 1 | 100 |  |  | 4,469147798 |  | 38,83254163 | 35,511682 | 33,24784231 |  |  |  |
| **PTPN22 wt 25uM H2O2** | | 43,4884446 |  | 0,875771578 | 87,57715781 |  |  | 97,26724967 |  | 34,31834659 | 39,67431092 | **31,9840417** |  |  |  |
| **PTPN22 mut 25uM H2O2** | | 25,00193109 |  | 0,906296611 | 90,6296611 |  |  | 31,22145889 |  | 36,95069113 | 34,9051965 | 31,260104 |  |  |  |
| **PTPN22 wt 50uM H2O2** | | 42,1641861 |  | 0,849103621 | 84,91036213 |  |  |  |  | 31,57471215 | 36,20904822 | **33,80080718** |  |  |  |
| **PTPN22 mut 50uM H2O2** | | 21,32180228 #DIV/0!  #DIV/0! |  | 0,772895385 | 77,28953847 |  |  |  |  | 34,54707642 | 33,41881996 | **32,142307** |  |  |  |

**PTPN22 reduced control PTPN22 3.4uM H2O2 PTPN22 6.3uM H2O2 PTPN22 12.5uM H2O2 PTPN22 25uM H2O2 PTPN22 50uM H2O2**

Protocol Layout:

- 20mM Hepes Buffer 0,1mM DTPA, pH 7.4, 10min
- Wash 1ml spinn collumn slurry 5x with Hepes Buffer
- Spinn 1000 rpm for 2 min, apply 50μl PTP1B from Freezer (recombinant HishPTP1B in 20mM Hepes, 1mM TCEP), ( desalt 1x)
- Measure PTP1B (reduced PTP) and protein concentration with Bradford -> (0,585/1,16) X5 -> - 0,5 μg/μl
- Prepare 16.7mM (15mM end concentration) pNPP (Sigma) substrate sollution Mw 371

**PTP1B activity catalytic domain exposed to 50μM H2O2 and 25mM Bicarbonat and TrxR1, Trx, Prx2**

- prepare 20μl PTP1B, 600nM
- add 20μl from each condition in duplicates to 96-well plate
- Add 180μl substrate solution


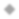

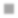

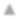

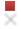

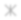

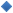

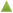

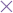

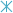

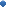

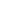

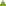

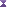


60

50

40

30

20

PTPN22 wt control #REF!

PTPN22 mut control PTPN22 wt 25uM H2O2

PTPN22 mut 25uM H2O2

10

0

0

0,2 0,4 0,6 0,8 1

1,2

Application: Tecan i-control Tecan i-control , 2.0.10.0

Device: infinite 200Pro Serial number: 1307001123 Serial number of connected stacker: Firmware: V_3.40_01/15_Infinite (Dec 23 201 MAI, V_3.40_01/15_Infinite (Dec 23 2014/12.45.11)

Date: #########

Time: 14:34:04

System MTC-MU059-S

User MTC-MU059-S\fretho

Plate Greiner 96 Flat Bottom Transparent Polystyrene Cat. No.: 655101/655161/655192 [GRE96ft.pdfx] Plate-ID (Stacker)

List of actions in this measurement script: Kinetic

Absorbance

Label: Label1

Kinetic Measurement

Kinetic duration 00:06:00

Interval Time 00:00:20

Measurement Wavelength 405 nm

Bandwidth 10 nm

Number of Flashes 5

Settle Time 0 ms

Part of Plate E1-F2; F3-F12

Start Time: 2020 05-19 14:34:06

| Cycle Nr. | 1 | 2 | 3 | 4 | 5 | 6 | 7 | 8 | 9 | 10 | 11 | 12 | 13 | 14 | 15 | 16 | 17 | 18 | 19 |
| --- | --- | --- | --- | --- | --- | --- | --- | --- | --- | --- | --- | --- | --- | --- | --- | --- | --- | --- | --- |
| Time [s] | 0 | 20 | 40 | 60 | 80 | 100 | 120 | 140 | 160 | 180 | 200 | 220 | 240 | 260 | 280 | 300 | 320 | 340 | 360 |
| Temp. [°C] | 25 | 25,2 | 25,4 | 25,2 | 25 | 25,1 | 25,3 | 25,2 | 25,4 | 25,4 | 25,4 | 25,2 | 25,2 | 25,4 | 25,2 | 25,4 | 25,3 | 25,4 | 25,3 |
| E1 | 0,2581 | 0,2589 | 0,2633 | 0,271 | 0,2761 | 0,2846 | 0,2909 | 0,2984 | 0,304 | 0,311 | 0,3176 | 0,3215 | 0,3301 | 0,3344 | 0,3428 | 0,3478 | 0,354 | 0,3615 | 0,368 |
| E2 | 0,2624 | 0,2658 | 0,2661 | 0,2733 | 0,2777 | 0,2844 | 0,2926 | 0,3005 | 0,3066 | 0,3142 | 0,3193 | 0,3276 | 0,3339 | 0,3399 | 0,3468 | 0,3525 | 0,3601 | 0,3672 | 0,3712 |
| F1 | 0,2626 | 0,263 | 0,2718 | 0,2695 | 0,2648 | 0,2704 | 0,2696 | 0,2797 | 0,2668 | 0,2715 | 0,2742 | 0,2768 | 0,2746 | 0,2778 | 0,2844 | 0,278 | 0,2806 | 0,2858 | 0,2812 |
| F2 | 0,2596 | 0,2596 | 0,2584 | 0,2587 | 0,2583 | 0,2579 | 0,2585 | 0,2581 | 0,2584 | 0,2579 | 0,2583 | 0,259 | 0,2586 | 0,2557 | 0,2577 | 0,2562 | 0,2576 | 0,2573 | 0,2559 |
| F3 | 0,2514 | 0,2521 | 0,2518 | 0,2568 | 0,2598 | 0,2617 | 0,2667 | 0,2709 | 0,2739 | 0,2787 | 0,2816 | 0,2859 | 0,2896 | 0,2914 | 0,2956 | 0,298 | 0,3014 | 0,3046 | 0,3075 |
| F4 | 0,2513 | 0,2565 | 0,255 | 0,2597 | 0,262 | 0,2649 | 0,2713 | 0,2742 | 0,2792 | 0,2832 | 0,2864 | 0,2905 | 0,295 | 0,2971 | 0,3014 | 0,3034 | 0,3087 | 0,3112 | 0,3134 |
| F5 | 0,2573 | 0,2736 | 0,263 | 0,269 | 0,2739 | 0,2801 | 0,2879 | 0,2924 | 0,2996 | 0,3046 | 0,311 | 0,3168 | 0,3233 | 0,3274 | 0,3346 | 0,3408 | 0,3457 | 0,351 | 0,3557 |
| F6 | 0,2557 | 0,2569 | 0,2623 | 0,268 | 0,2749 | 0,2798 | 0,2851 | 0,2918 | 0,2968 | 0,3031 | 0,3102 | 0,3167 | 0,3219 | 0,3265 | 0,3334 | 0,3373 | 0,3436 | 0,3488 | 0,3535 |
| F7 | 0,2578 | 0,2575 | 0,2562 | 0,2594 | 0,2629 | 0,2664 | 0,2709 | 0,2741 | 0,2786 | 0,2821 | 0,2838 | 0,2887 | 0,2918 | 0,2941 | 0,2997 | 0,3 | 0,3033 | 0,3068 | 0,3091 |
| F8 | 0,2536 | 0,2538 | 0,2562 | 0,2594 | 0,2624 | 0,2656 | 0,2693 | 0,2725 | 0,2758 | 0,2796 | 0,2826 | 0,2863 | 0,2901 | 0,2911 | 0,2942 | 0,2972 | 0,3002 | 0,3039 | 0,3048 |
| F9 | 0,2588 | 0,2601 | 0,2643 | 0,2714 | 0,2771 | 0,2818 | 0,2892 | 0,295 | 0,2996 | 0,3074 | 0,3127 | 0,3184 | 0,325 | 0,3285 | 0,3367 | 0,3407 | 0,3455 | 0,3512 | 0,3566 |
| F10 | 0,2566 | 0,258 | 0,2638 | 0,2693 | 0,2735 | 0,2786 | 0,2846 | 0,2903 | 0,2963 | 0,3015 | 0,3072 | 0,3125 | 0,3183 | 0,3219 | 0,3287 | 0,3316 | 0,3359 | 0,3423 | 0,3466 |
| F11 | 0,2497 | 0,2516 | 0,2551 | 0,2573 | 0,2595 | 0,2629 | 0,2658 | 0,2688 | 0,2711 | 0,2745 | 0,278 | 0,2805 | 0,2836 | 0,2853 | 0,288 | 0,2896 | 0,2923 | 0,295 | 0,2962 |
| F12 | 0,2499 | 0,252 | 0,2536 | 0,2569 | 0,2608 | 0,2638 | 0,2668 | 0,2696 | 0,2733 | 0,2768 | 0,2786 | 0,2817 | 0,2835 | 0,2855 | 0,289 | 0,2906 | 0,293 | 0,2951 | 0,2982 |

**PTPN22 wt control PTPN22 wt control PTPN22 mut control PTPN22 mut control PTPN22 wt 25uM H2O2 PTPN22 wt 25uM H2O2 PTPN22 mut 25uM H2O2 PTPN22 mut 25uM H2O2 PTPN22 wt 50uM H2O PTPN22 wt 50uM H2O PTPN22 mut 50uM H2O2 PTPN22 mut 50uM H2O2**

**Time (sec) Blank Blank**

| 20 | 0,256099999 0,255600005 | 0,25060001 | | 0,255499989 | 0,252600014 | 0,253399998 | 0,257400006 | 0,251399994 | 0,248500004 | 0,246800005 | 0,251300007 | 0,249799997 | 0,244100004 | 0,243599996 |
| --- | --- | --- | --- | --- | --- | --- | --- | --- | --- | --- | --- | --- | --- | --- |
| 40 | 0,257200003 0,256099999 | 0,255199999 | | 0,261200011 | 0,252400011 | 0,251599997 | 0,259799987 | 0,256500006 | 0,2509 | 0,248999998 | 0,256900012 | 0,254200011 | 0,246600002 | 0,247099996 |
| 60 | 0,256599993 0,256799996 | 0,261099994 | | 0,26730001 | 0,256099999 | 0,255100012 | 0,263200015 | 0,260800004 | 0,252000004 | 0,251300007 | 0,259799987 | 0,256900012 | 0,248099998 | 0,246999994 |
| 80 | 0,257699996 0,256799996 | 0,269600004 | | 0,273200005 | 0,259799987 | 0,259299994 | 0,276499987 | 0,266099989 | 0,255600005 | 0,253899992 | 0,266099989 | 0,261500001 | 0,249500006 | 0,249200001 |
| 100 | 0,256399989 0,256099999 | 0,274300009 | | 0,279799998 | 0,263599992 | 0,261099994 | 0,284999996 | 0,270799994 | 0,256999999 | 0,254700005 | 0,268000007 | 0,263300002 | 0,251199991 | 0,251300007 |
| 120 | 0,255699992 0,255199999 | 0,281300008 | | 0,285600007 | 0,265599996 | 0,265100002 | 0,279000014 | 0,273499995 | 0,2597 | 0,255899996 | 0,271800011 | 0,26730001 | 0,25150001 | 0,253100008 |
| 140 | 0,2579 0,257800013 | 0,289600015 | | 0,294800013 | 0,272199988 | 0,270599991 | 0,287600011 | 0,280699998 | 0,262699991 | 0,261900008 | 0,279399991 | 0,271699995 | 0,255299985 | 0,255600005 |
| 160 | 0,257200003 0,257099986 | 0,2949 | | 0,301200002 | 0,275400013 | 0,274300009 | 0,292400002 | 0,284000009 | 0,264999986 | 0,263200015 | 0,282999992 | 0,274699986 | 0,256599993 | 0,256199986 |
| 180 | 0,256900012 0,257099986 | 0,302599996 | | 0,308999985 | 0,280499995 | 0,278100014 | 0,297899991 | 0,289400011 | 0,268099993 | 0,265399992 | 0,28639999 | 0,278699994 | 0,256199986 | 0,256500006 |
| 200 | 0,256199986 0,257200003 | 0,309700012 | | 0,314599991 | 0,284099996 | 0,282099992 | 0,30399999 | 0,294999987 | 0,270399988 | 0,2667 | 0,292100012 | 0,283199996 | 0,258100003 | 0,258899987 |
| 220 | 0,256999999 0,256700009 | 0,315299988 | | 0,321799994 | 0,287999988 | 0,2852 | 0,308699995 | 0,299400002 | 0,273299992 | 0,26910001 | 0,296700001 | 0,28549999 | 0,259499997 | 0,260399997 |
| 240 | 0,255499989 0,256599993 | 0,322100013 | | 0,328500003 | 0,290699989 | 0,289200008 | 0,314599991 | 0,30430001 | 0,275200009 | 0,271100014 | 0,298400015 | 0,288899988 | 0,260899991 | 0,260500014 |
| 260 | 0,256000012 0,256599993 | 0,3292 | | 0,336899996 | 0,296400011 | 0,29460001 | 0,320499986 | 0,309100002 | 0,279300004 | 0,273400009 | 0,304199994 | 0,292400002 | 0,262400001 | 0,263300002 |
| 280 | 0,255299985 0,256900012 | 0,335299999 | | 0,343499988 | 0,300599992 | 0,297800004 | 0,325899988 | 0,314999998 | 0,280299991 | 0,2764 | 0,308299989 | 0,296799988 | 0,263500005 | 0,264699996 |
| 300 | 0,256099999 0,257800013 | 0,342700005 | | 0,349599987 | 0,30340001 | 0,299600005 | 0,331400007 | 0,320100009 | 0,282599986 | 0,277200013 | 0,312700003 | 0,300500005 | 0,265399992 | 0,265399992 |
| 320 | 0,257499993 0,257099986 | 0,350800008 | | 0,3565 | 0,307099998 | 0,30430001 | 0,337900013 | 0,324000001 | 0,285600007 | 0,280099988 | 0,317600012 | 0,305099994 | 0,268000007 | 0,267399997 |
| 340 | 0,255100012 0,255400002 | 0,355100006 | | 0,362300009 | 0,309799999 | 0,306699991 | 0,342000008 | 0,328500003 | 0,287299991 | 0,281800002 | 0,321700007 | 0,305500001 | 0,267100006 | 0,266900003 |
| 360 | 0,256799996 0,255400002 | 0,362899989 | | 0,370499998 | 0,313699991 | 0,310400009 | 0,349599987 | 0,333600014 | 0,289900005 | 0,284200013 | 0,326000005 | 0,310499996 | 0,269199997 | 0,269499987 |
| slope | -2,40453E-06 5,62441E-07 | 0,000335031 | | 0,000342611 | 0,000192198 | 0,000182915 | 0,000269556 | 0,000243024 | 0,000124881 | 0,00011049 | 0,000218638 | 0,000178385 | 7,18989E-05 | 7,28689E-05 |
| slope w/o |  | 0,000335952 | | 0,000343532 | 0,000193119 | 0,000183836 | 0,000270477 | 0,000243945 | 0,000125802 | 0,000111411 | 0,000219559 | 0,000179306 | 7,28199E-05 | 7,379E-05 |
| blank | |  |  | 0,000339742 |  | 0,000188478 |  | 0,000257211 |  | 0,000118607 |  | 0,000199432 |  | 7,33049E-05 |
|  | |  |  | 0,020384519 |  | 0,011308668 |  | 0,015432663 |  | 0,007116408 |  | 0,011965943 |  | 0,004398297 |
|  | |  |  | 3,042465474 |  | 1,687860966 |  | 2,303382497 |  | 1,062150487 |  | 1,785961698 |  | 0,656462177 |
|  | |  |  | 50,7077579 |  | 28,13101611 |  | 38,38970829 |  | 17,70250812 |  | 29,76602831 |  | 10,94103628 |
|  | | **min -1** |  | 50,7077579 |  | 28,13101611 |  | 38,38970829 |  | 17,70250812 |  | 29,76602831 |  | 10,94103628 |

**15 min**

| **PTPN22 wt control** 50,7077579 | 1 | 100 |
| --- | --- | --- |
| **PTPN22 mut control** 28,13101611 | 1 | 100 |
| **PTPN22 wt 25uM H2O2** 38,38970829 | 0,757077612 | 75,70776124 |
| **PTPN22 mut 25uM H2O2** 17,70250812 | 0,629287902 | 62,92879023 |
| **PTPN22 wt 50uM H2O2** 29,76602831 | 0,587011328 | 58,7011328 |
| **PTPN22 mut 50uM H2O2** 10,94103628 | 0,388931428 | 38,89314285 |

#DIV/0! #DIV/0!

Protocol Layout:

- 20mM Hepes Buffer 0,1mM DTPA, pH 7.4, 10min
- Wash 1ml spinn collumn slurry 5x with Hepes Buffer
- Spinn 1000 rpm for 2 min, apply 50μl PTP1B from Freezer (recombinant HishPTP1B in 20mM Hepes, 1mM TCEP), ( desalt 1x)
- Measure PTP1B (reduced PTP) and protein concentration with Bradford -> (0,585/1,16) X5 -> - 0,5 μg/μl
- Prepare 16.7mM (15mM end concentration) pNPP (Sigma) substrate sollution Mw 371

**PTP1B activity catalytic domain exposed to 50μM H2O2 and 25mM Bicarbonat and TrxR1, Trx, Prx2**

- prepare 20μl PTP1B, 600nM
- add 20μl from each condition in duplicates to 96-well plate
- Add 180μl substrate solution

60

50

40

30

20

PTPN22 wt control #REF!

PTPN22 mut control PTPN22 wt 25uM H2O2

PTPN22 mut 25uM H2O2

10

0

0

0,2 0,4 0,6 0,8 1

1,2

Application: Tecan i-control Tecan i-control , 2.0.10.0

Device: infinite 200Pro Serial number: 1307001123 Serial number of connected stacker: Firmware: V_3.40_01/15_Infinite (Dec 23 201 MAI, V_3.40_01/15_Infinite (Dec 23 2014/12.45.11)

Date: #########

Time: 14:44:08

System MTC-MU059-S

User MTC-MU059-S\fretho

Plate Greiner 96 Flat Bottom Transparent Polystyrene Cat. No.: 655101/655161/655192 [GRE96ft.pdfx] Plate-ID (Stacker)

List of actions in this measurement script: Kinetic

Absorbance

Label: Label1

Kinetic Measurement

Kinetic duration 00:06:00

Interval Time 00:00:20

Measurement Wavelength 405 nm

Bandwidth 10 nm

Number of Flashes 5

Settle Time 0 ms

Part of Plate E1-E12; F1-F2

Start Time: 2020 05-19 14:44:10

| Cycle Nr. | 1 | 2 | 3 | 4 | 5 | 6 | 7 | 8 | 9 | 10 | 11 | 12 | 13 | 14 | 15 | 16 | 17 | 18 | 19 |
| --- | --- | --- | --- | --- | --- | --- | --- | --- | --- | --- | --- | --- | --- | --- | --- | --- | --- | --- | --- |
| Time [s] | 0 | 20 | 40 | 60 | 80 | 100 | 120 | 140 | 160 | 180 | 200 | 220 | 240 | 260 | 280 | 300 | 320 | 340 | 360 |
| Temp. [°C] | 25,4 | 25,6 | 25,3 | 25,4 | 25,6 | 25,3 | 25,3 | 25,8 | 25,3 | 25,5 | 25,3 | 25,4 | 25,5 | 25,6 | 25,5 | 25,4 | 25,5 | 25,3 | 25,7 |
| E1 | 0,248 | 0,2506 | 0,2552 | 0,2611 | 0,2696 | 0,2743 | 0,2813 | 0,2896 | 0,2949 | 0,3026 | 0,3097 | 0,3153 | 0,3221 | 0,3292 | 0,3353 | 0,3427 | 0,3508 | 0,3551 | 0,3629 |
| E2 | 0,252 | 0,2555 | 0,2612 | 0,2673 | 0,2732 | 0,2798 | 0,2856 | 0,2948 | 0,3012 | 0,309 | 0,3146 | 0,3218 | 0,3285 | 0,3369 | 0,3435 | 0,3496 | 0,3565 | 0,3623 | 0,3705 |
| E3 | 0,2505 | 0,2526 | 0,2524 | 0,2561 | 0,2598 | 0,2636 | 0,2656 | 0,2722 | 0,2754 | 0,2805 | 0,2841 | 0,288 | 0,2907 | 0,2964 | 0,3006 | 0,3034 | 0,3071 | 0,3098 | 0,3137 |
| E4 | 0,2495 | 0,2534 | 0,2516 | 0,2551 | 0,2593 | 0,2611 | 0,2651 | 0,2706 | 0,2743 | 0,2781 | 0,2821 | 0,2852 | 0,2892 | 0,2946 | 0,2978 | 0,2996 | 0,3043 | 0,3067 | 0,3104 |
| E5 | 0,2525 | 0,2574 | 0,2598 | 0,2632 | 0,2765 | 0,285 | 0,279 | 0,2876 | 0,2924 | 0,2979 | 0,304 | 0,3087 | 0,3146 | 0,3205 | 0,3259 | 0,3314 | 0,3379 | 0,342 | 0,3496 |
| E6 | 0,2537 | 0,2514 | 0,2565 | 0,2608 | 0,2661 | 0,2708 | 0,2735 | 0,2807 | 0,284 | 0,2894 | 0,295 | 0,2994 | 0,3043 | 0,3091 | 0,315 | 0,3201 | 0,324 | 0,3285 | 0,3336 |
| E7 | 0,2495 | 0,2485 | 0,2509 | 0,252 | 0,2556 | 0,257 | 0,2597 | 0,2627 | 0,265 | 0,2681 | 0,2704 | 0,2733 | 0,2752 | 0,2793 | 0,2803 | 0,2826 | 0,2856 | 0,2873 | 0,2899 |
| E8 | 0,248 | 0,2468 | 0,249 | 0,2513 | 0,2539 | 0,2547 | 0,2559 | 0,2619 | 0,2632 | 0,2654 | 0,2667 | 0,2691 | 0,2711 | 0,2734 | 0,2764 | 0,2772 | 0,2801 | 0,2818 | 0,2842 |
| E9 | 0,2535 | 0,2513 | 0,2569 | 0,2598 | 0,2661 | 0,268 | 0,2718 | 0,2794 | 0,283 | 0,2864 | 0,2921 | 0,2967 | 0,2984 | 0,3042 | 0,3083 | 0,3127 | 0,3176 | 0,3217 | 0,326 |
| E10 | 0,2531 | 0,2498 | 0,2542 | 0,2569 | 0,2615 | 0,2633 | 0,2673 | 0,2717 | 0,2747 | 0,2787 | 0,2832 | 0,2855 | 0,2889 | 0,2924 | 0,2968 | 0,3005 | 0,3051 | 0,3055 | 0,3105 |
| E11 | 0,2454 | 0,2441 | 0,2466 | 0,2481 | 0,2495 | 0,2512 | 0,2515 | 0,2553 | 0,2566 | 0,2562 | 0,2581 | 0,2595 | 0,2609 | 0,2624 | 0,2635 | 0,2654 | 0,268 | 0,2671 | 0,2692 |
| E12 | 0,2462 | 0,2436 | 0,2471 | 0,247 | 0,2492 | 0,2513 | 0,2531 | 0,2556 | 0,2562 | 0,2565 | 0,2589 | 0,2604 | 0,2605 | 0,2633 | 0,2647 | 0,2654 | 0,2674 | 0,2669 | 0,2695 |
| F1 | 0,2554 | 0,2561 | 0,2572 | 0,2566 | 0,2577 | 0,2564 | 0,2557 | 0,2579 | 0,2572 | 0,2569 | 0,2562 | 0,257 | 0,2555 | 0,256 | 0,2553 | 0,2561 | 0,2575 | 0,2551 | 0,2568 |
| F2 | 0,2556 | 0,2556 | 0,2561 | 0,2568 | 0,2568 | 0,2561 | 0,2552 | 0,2578 | 0,2571 | 0,2571 | 0,2572 | 0,2567 | 0,2566 | 0,2566 | 0,2569 | 0,2578 | 0,2571 | 0,2554 | 0,2554 |

**PTPN22 wt control PTPN22 wt control PTPN22 mut control PTPN22 mut control PTPN22 wt 25uM H2O2 PTPN22 wt 25uM H2O2 PTPN22 mut 25uM H2O2 PTPN22 mut 25uM H2O2 PTPN22 wt 50uM H2O PTPN22 wt 50uM H2O PTPN22 mut 50uM H2O2 PTPN22 mut 50uM H2O**

**Time (sec) Blank Blank**

| 20 | 0,25940001 0,258399993 | 0,254299998 | | 0,255600005 | 0,253500015 | 0,25029999 | 0,248699993 | 0,2491 | 0,245399997 | 0,2447 | 0,247099996 | 0,246299997 | 0,243100002 | 0,240899995 |
| --- | --- | --- | --- | --- | --- | --- | --- | --- | --- | --- | --- | --- | --- | --- |
| 40 | 0,257499993 0,257400006 | 0,2579 | | 0,266600013 | 0,250699997 | 0,254500002 | 0,251300007 | 0,252600014 | 0,245399997 | 0,245199993 | 0,247199997 | 0,247799993 | 0,241799995 | 0,2421 |
| 60 | 0,257999986 0,257299989 | 0,261299998 | | 0,264499992 | 0,254400015 | 0,256900012 | 0,253800005 | 0,254400015 | 0,246399999 | 0,245399997 | 0,248799995 | 0,246700004 | 0,241500005 | 0,241799995 |
| 80 | 0,258899987 0,256599993 | 0,267699987 | | 0,268700004 | 0,256500006 | 0,264699996 | 0,256300002 | 0,258399993 | 0,247600004 | 0,247600004 | 0,250400007 | 0,248500004 | 0,2421 | 0,242500007 |
| 100 | 0,257999986 0,256500006 | 0,272599995 | | 0,272300005 | 0,259799987 | 0,26820001 | 0,258100003 | 0,259600013 | 0,248199999 | 0,248199999 | 0,250499994 | 0,254999995 | 0,250400007 | 0,242599994 |
| 120 | 0,2579 0,255199999 | 0,277500004 | | 0,27669999 | 0,263300002 | 0,271699995 | 0,260899991 | 0,261099994 | 0,250099987 | 0,249699995 | 0,253100008 | 0,257999986 | 0,251399994 | 0,244800001 |
| 140 | 0,257600009 0,256900012 | 0,284000009 | | 0,282499999 | 0,26730001 | 0,275599986 | 0,265899986 | 0,266600013 | 0,252499998 | 0,252299994 | 0,256999999 | 0,261599988 | 0,252299994 | 0,245199993 |
| 160 | 0,258599997 0,257299989 | 0,289600015 | | 0,287499994 | 0,270900011 | 0,282400012 | 0,2685 | 0,269400001 | 0,253199995 | 0,253100008 | 0,256999999 | 0,259299994 | 0,252999991 | 0,2465 |
| 180 | 0,259299994 0,256599993 | 0,295899987 | | 0,294200003 | 0,276300013 | 0,284299999 | 0,272899985 | 0,273099989 | 0,256199986 | 0,256399989 | 0,261299998 | 0,258100003 | 0,254400015 | 0,247700006 |
| 200 | 0,256599993 0,255699992 | 0,300199986 | | 0,296499997 | 0,276199996 | 0,287999988 | 0,274500012 | 0,273799986 | 0,255800009 | 0,256399989 | 0,260899991 | 0,257699996 | 0,251800001 | 0,246900007 |
| 220 | 0,2579 0,256500006 | 0,306100011 | | 0,302700013 | 0,281699985 | 0,29370001 | 0,278100014 | 0,277399987 | 0,258100003 | 0,256999999 | 0,263300002 | 0,25999999 | 0,252700001 | 0,247799993 |
| 240 | 0,257499993 0,256700009 | 0,311399996 | | 0,309399992 | 0,28459999 | 0,296600014 | 0,281500012 | 0,281300008 | 0,259799987 | 0,25850001 | 0,264800012 | 0,261200011 | 0,253500015 | 0,248099998 |
| 260 | 0,25819999 0,257200003 | 0,319099993 | | 0,316100001 | 0,289400011 | 0,30250001 | 0,28549999 | 0,28580001 | 0,25999999 | 0,261799991 | 0,26820001 | 0,263300002 | 0,255400002 | 0,248999998 |
| 280 | 0,257299989 0,257099986 | 0,325599998 | | 0,320199996 | 0,292800009 | 0,3046 | 0,289499998 | 0,288399994 | 0,263200015 | 0,262199998 | 0,269199997 | 0,265799999 | 0,255100012 | 0,2509 |
| 300 | 0,257999986 0,258399993 | 0,331200004 | | 0,326299995 | 0,296499997 | 0,308699995 | 0,294 | 0,290899992 | 0,263200015 | 0,263599992 | 0,273600012 | 0,26699999 | 0,255800009 | 0,251300007 |
| 320 | 0,257400006 0,258300006 | 0,336600006 | | 0,330300003 | 0,300500005 | 0,31220001 | 0,296099991 | 0,29429999 | 0,266400009 | 0,264200002 | 0,274399996 | 0,268299997 | 0,257099986 | 0,252099991 |
| 340 | 0,257299989 0,257800013 | 0,341699988 | | 0,336100012 | 0,302899987 | 0,315600008 | 0,298900008 | 0,298099995 | 0,266600013 | 0,264999986 | 0,275299996 | 0,26910001 | 0,257699996 | 0,252900004 |
| 360 | 0,257699996 0,257999986 | 0,348100007 | | 0,342999995 | 0,306600004 | 0,318800002 | 0,30340001 | 0,299800009 | 0,26820001 | 0,26730001 | 0,278800011 | 0,273000002 | 0,25819999 | 0,253300011 |
| slope | -3,04954E-06 2,43035E-06 | 0,000283179 | | 0,000253179 | 0,000171491 | 0,000204835 | 0,000163658 | 0,000152265 | 7,18422E-05 | 6,99174E-05 | 9,70021E-05 | 7,18112E-05 | 4,7549E-05 | 3,742E-05 |
| slope w/o |  | 0,000283488 | | 0,000253488 | 0,000171801 | 0,000205145 | 0,000163968 | 0,000152575 | 7,21518E-05 | 7,0227E-05 | 9,73117E-05 | 7,21208E-05 | 4,78586E-05 | 3,77296E-05 |
| blank |  |  |  | 0,000268488 |  | 0,000188473 |  | 0,000158271 |  | 7,11894E-05 |  | 8,47162E-05 |  | 4,27941E-05 |
|  |  |  |  | 0,016109288 |  | 0,011308359 |  | 0,009496284 |  | 0,004271364 |  | 0,005082973 |  | 0,002567647 |
|  |  |  |  | 2,404371346 |  | 1,687814792 |  | 1,417355872 |  | 0,637516969 |  | 0,758652748 |  | 0,383230957 |
|  |  |  |  | 40,07285576 |  | 28,13024653 |  | 23,62259787 |  | 10,62528282 |  | 12,64421246 |  | 6,387182615 |
|  |  | **min -1** |  | 40,07285576 |  | 28,13024653 |  | 23,62259787 |  | 10,62528282 |  | 12,64421246 |  | 6,387182615 |
|  | **30 min PTPN22 wt control** 40,07285576 |  | 1 | 100 |  |  | 30 |  |  |  |  |  |  |  |
|  | **PTPN22 mut control** 28,13024653 |  | 1 | 100 |  |  |  |  |  |  |  |  |  |  |
|  | **PTPN22 wt 25uM H2O2** 23,62259787 |  | 0,589491251 | 58,94912509 |  |  |  |  |  |  |  |  |  |  |
|  | **PTPN22 mut 25uM H2O2** 10,62528282 |  | 0,377717373 | 37,77173732 |  |  |  |  |  |  |  |  |  |  |
|  | **PTPN22 wt 50uM H2O2** 12,64421246 |  | 0,315530606 | 31,55306059 |  |  |  |  |  |  |  |  |  |  |
|  | **PTPN22 mut 50uM H2O2** 6,387182615 |  | 0,22705747 | 22,70574703 |  |  |  |  |  |  |  |  |  |  |

#DIV/0! #DIV/0!

Protocol Layout:

- 20mM Hepes Buffer 0,1mM DTPA, pH 7.4, 10min
- Wash 1ml spinn collumn slurry 5x with Hepes Buffer
- Spinn 1000 rpm for 2 min, apply 50μl PTP1B from Freezer (recombinant HishPTP1B in 20mM Hepes, 1mM TCEP), ( desalt 1x)
- Measure PTP1B (reduced PTP) and protein concentration with Bradford -> (0,585/1,16) X5 -> - 0,5 μg/μl
- Prepare 16.7mM (15mM end concentration) pNPP (Sigma) substrate sollution Mw 371

**PTP1B activity catalytic domain exposed to 50μM H2O2 and 25mM Bicarbonat and TrxR1, Trx, Prx2**

- prepare 20μl PTP1B, 600nM
- add 20μl from each condition in duplicates to 96-well plate
- Add 180μl substrate solution

45

40

35

30

25

20

15

PTPN22 wt control #REF!

PTPN22 mut control

PTPN22 wt 25uM H2O2 PTPN22 mut 25uM H2O2

10

5

0

0

0,2 0,4 0,6 0,8

1

1,2

Application: Tecan i-control Tecan i-control , 2.0.10.0

Device: infinite 200Pro Serial number: 1307001123 Serial number of connected stacker: Firmware: V_3.40_01/15_Infinite (Dec 23 201 MAI, V_3.40_01/15_Infinite (Dec 23 2014/12.45.11)

Date: #########

Time: 14:59:18

System MTC-MU059-S

User MTC-MU059-S\fretho

Plate Greiner 96 Flat Bottom Transparent Polystyrene Cat. No.: 655101/655161/655192 [GRE96ft.pdfx] Plate-ID (Stacker)

List of actions in this measurement script: Kinetic

Absorbance

Label: Label1

Kinetic Measurement

Kinetic duration 00:06:00

Interval Time 00:00:20

Measurement Wavelength 405 nm

Bandwidth 10 nm

Number of Flashes 5

Settle Time 0 ms

Part of Plate E1-E12; F1-F2

Start Time: 2020 05-19 14:59:20

| Cycle Nr. | 1 | 2 | 3 | 4 | 5 | 6 | 7 | 8 | 9 | 10 | 11 | 12 | 13 | 14 | 15 | 16 | 17 | 18 | 19 |
| --- | --- | --- | --- | --- | --- | --- | --- | --- | --- | --- | --- | --- | --- | --- | --- | --- | --- | --- | --- |
| Time [s] | 0 | 20 | 40 | 60 | 80 | 100 | 120 | 140 | 160 | 180 | 200 | 220 | 240 | 260 | 280 | 300 | 320 | 340 | 360 |
| Temp. [°C] | 25,7 | 25,8 | 25,9 | 25,6 | 25,8 | 25,6 | 25,6 | 25,6 | 25,6 | 25,7 | 25,6 | 25,6 | 25,7 | 25,9 | 25,7 | 25,6 | 25,8 | 25,7 | 25,8 |
| E1 | 0,2515 | 0,2543 | 0,2579 | 0,2613 | 0,2677 | 0,2726 | 0,2775 | 0,284 | 0,2896 | 0,2959 | 0,3002 | 0,3061 | 0,3114 | 0,3191 | 0,3256 | 0,3312 | 0,3366 | 0,3417 | 0,3481 |
| E2 | 0,2543 | 0,2556 | 0,2666 | 0,2645 | 0,2687 | 0,2723 | 0,2767 | 0,2825 | 0,2875 | 0,2942 | 0,2965 | 0,3027 | 0,3094 | 0,3161 | 0,3202 | 0,3263 | 0,3303 | 0,3361 | 0,343 |
| E3 | 0,2488 | 0,2535 | 0,2507 | 0,2544 | 0,2565 | 0,2598 | 0,2633 | 0,2673 | 0,2709 | 0,2763 | 0,2762 | 0,2817 | 0,2846 | 0,2894 | 0,2928 | 0,2965 | 0,3005 | 0,3029 | 0,3066 |
| E4 | 0,2485 | 0,2503 | 0,2545 | 0,2569 | 0,2647 | 0,2682 | 0,2717 | 0,2756 | 0,2824 | 0,2843 | 0,288 | 0,2937 | 0,2966 | 0,3025 | 0,3046 | 0,3087 | 0,3122 | 0,3156 | 0,3188 |
| E5 | 0,2492 | 0,2487 | 0,2513 | 0,2538 | 0,2563 | 0,2581 | 0,2609 | 0,2659 | 0,2685 | 0,2729 | 0,2745 | 0,2781 | 0,2815 | 0,2855 | 0,2895 | 0,294 | 0,2961 | 0,2989 | 0,3034 |
| E6 | 0,2524 | 0,2491 | 0,2526 | 0,2544 | 0,2584 | 0,2596 | 0,2611 | 0,2666 | 0,2694 | 0,2731 | 0,2738 | 0,2774 | 0,2813 | 0,2858 | 0,2884 | 0,2909 | 0,2943 | 0,2981 | 0,2998 |
| E7 | 0,2454 | 0,2454 | 0,2454 | 0,2464 | 0,2476 | 0,2482 | 0,2501 | 0,2525 | 0,2532 | 0,2562 | 0,2558 | 0,2581 | 0,2598 | 0,26 | 0,2632 | 0,2632 | 0,2664 | 0,2666 | 0,2682 |
| E8 | 0,249 | 0,2447 | 0,2452 | 0,2454 | 0,2476 | 0,2482 | 0,2497 | 0,2523 | 0,2531 | 0,2564 | 0,2564 | 0,257 | 0,2585 | 0,2618 | 0,2622 | 0,2636 | 0,2642 | 0,265 | 0,2673 |
| E9 | 0,2518 | 0,2471 | 0,2472 | 0,2488 | 0,2504 | 0,2505 | 0,2531 | 0,257 | 0,257 | 0,2613 | 0,2609 | 0,2633 | 0,2648 | 0,2682 | 0,2692 | 0,2736 | 0,2744 | 0,2753 | 0,2788 |
| E10 | 0,2506 | 0,2463 | 0,2478 | 0,2467 | 0,2485 | 0,255 | 0,258 | 0,2616 | 0,2593 | 0,2581 | 0,2577 | 0,26 | 0,2612 | 0,2633 | 0,2658 | 0,267 | 0,2683 | 0,2691 | 0,273 |
| E11 | 0,247 | 0,2431 | 0,2418 | 0,2415 | 0,2421 | 0,2504 | 0,2514 | 0,2523 | 0,253 | 0,2544 | 0,2518 | 0,2527 | 0,2535 | 0,2554 | 0,2551 | 0,2558 | 0,2571 | 0,2577 | 0,2582 |
| E12 | 0,2455 | 0,2409 | 0,2421 | 0,2418 | 0,2425 | 0,2426 | 0,2448 | 0,2452 | 0,2465 | 0,2477 | 0,2469 | 0,2478 | 0,2481 | 0,249 | 0,2509 | 0,2513 | 0,2521 | 0,2529 | 0,2533 |
| F1 | 0,2591 | 0,2594 | 0,2575 | 0,258 | 0,2589 | 0,258 | 0,2579 | 0,2576 | 0,2586 | 0,2593 | 0,2566 | 0,2579 | 0,2575 | 0,2582 | 0,2573 | 0,258 | 0,2574 | 0,2573 | 0,2577 |
| F2 | 0,2581 | 0,2584 | 0,2574 | 0,2573 | 0,2566 | 0,2565 | 0,2552 | 0,2569 | 0,2573 | 0,2566 | 0,2557 | 0,2565 | 0,2567 | 0,2572 | 0,2571 | 0,2584 | 0,2583 | 0,2578 | 0,258 |
