# Supplementary material for: Redox regulation of PTPN22 affects the severity of T-cell-dependent autoimmune inflammation
Source: eLife. 2022 May 19;11:e74549. doi: 10.7554/eLife.74549 (PMC9119677; doi:10.7554/eLife.74549)
Supplement: Figure 1—source data 5. [file elife-74549-fig1-data5.docx]

**170330**

**WTPTPN22 ctr WTPTPN22 ctr WTPTPN22reduced WTPTPN22reduced WTPTPN22+TrxR1+ WTPTPN22+TrxR WTPTPN22+TrxR1+NAD WTPTPN22+TrxR1+NA WTPTPN22+TrxR1 WTPTPN22+TrxR1+ mut PTPN22 ctr mut PTPN22 ctr mut PTPN22reduced mut PTPN22reduced mut PTPN22+TrxR1+NADPH mut PTPN22+TrxR1+NADPH mut PTPN22+TrxR1+Nmut PTPN22+TrxR1+Nmut PTPN22+TrxR1+NA mut PTPN22+TrxR1+NADPH+Trp14**

| **Time (sec) Blank** | **Blank** |  | | | | | | | | | | | | | | | | | | | | | |
| --- | --- | --- | --- | --- | --- | --- | --- | --- | --- | --- | --- | --- | --- | --- | --- | --- | --- | --- | --- | --- | --- | --- | --- |
| 20 | 0,261799991 0,2631 |  | 0,273999989 | 0,276800007 | 0,266099989 | 0,267500013 | 0,281399995 0,273699999 | 0,275200009 | 0,272300005 | 0,282999992 | 0,281599998 | 0,256999999 | 0,2579 | 0,251300007 | 0,253100008 | 0,265399992 | 0,260399997 | 0,254200011 | 0,258700013 | 0,260899991 | 0,261200011 |  |  |
| 40 | 0,26030001 0,26199999 |  | 0,278499991 | 0,278699994 | 0,271699995 | 0,269300014 | 0,291900009 0,278100014 | 0,278200001 | 0,2764 | 0,289600015 | 0,286500007 | 0,258700013 | 0,258300006 | 0,250099987 | 0,250999987 | 0,266000003 | 0,261700004 | 0,251199991 | 0,255400002 | 0,262499988 | 0,263000011 |  |  |
| 60 | 0,25909999 0,26210001 |  | 0,284700006 | 0,285899997 | 0,274100006 | 0,273699999 | 0,301400006 0,285899997 | 0,282799989 | 0,280400008 | 0,297199994 | 0,294699997 | 0,261000007 | 0,260100007 | 0,248799995 | 0,248300001 | 0,269699991 | 0,264699996 | 0,252000004 | 0,256399989 | 0,265500009 | 0,266799986 |  |  |
| 80 | 0,258899987 0,26100001 |  | 0,293900013 | 0,295599997 | 0,280600011 | 0,280000001 | 0,313199997 0,29550001 | 0,288399994 | 0,285400003 | 0,308999985 | 0,301899999 | 0,262699991 | 0,263900012 | 0,249400005 | 0,249200001 | 0,2764 | 0,271499991 | 0,252600014 | 0,257699996 | 0,26820001 | 0,269899994 |  |  |
| 100 | 0,259799987 0,2622 |  | 0,30430001 | 0,306100011 | 0,285299987 | 0,287200004 | 0,325199991 0,304800004 | 0,294099987 | 0,291900009 | 0,318899989 | 0,31099999 | 0,265700012 | 0,26820001 | 0,252799988 | 0,250200003 | 0,279500008 | 0,275900006 | 0,256399989 | 0,259200007 | 0,271899998 | 0,272300005 |  |  |
| 120 | 0,260899991 0,264 |  | 0,316700011 | 0,317799985 | 0,292100012 | 0,293900013 | 0,336400002 0,314099997 | 0,300799996 | 0,299299985 | 0,326999992 | 0,320300013 | 0,271100014 | 0,273299992 | 0,254099995 | 0,253100008 | 0,287 | 0,281300008 | 0,256500006 | 0,262199998 | 0,27669999 | 0,27790001 |  |  |
| 140 | 0,259799987 0,2626 |  | 0,322299987 | 0,327800006 | 0,296799988 | 0,299299985 | 0,344399989 0,320899993 | 0,306199998 | 0,303600013 | 0,335700005 | 0,328500003 | 0,273799986 | 0,274500012 | 0,253399998 | 0,253800005 | 0,290499985 | 0,284299999 | 0,257099986 | 0,263000011 | 0,278200001 | 0,280099988 |  |  |
| 160 | 0,259799987 0,26249999 |  | 0,331900001 | 0,338400006 | 0,301800013 | 0,305400014 | 0,354999989 0,327899992 | 0,312099993 | 0,308999985 | 0,345299989 | 0,335399985 | 0,277399987 | 0,277999997 | 0,255199999 | 0,254999995 | 0,293900013 | 0,290499985 | 0,259499997 | 0,265300006 | 0,281800002 | 0,283699989 |  |  |
| 180 | 0,25819999 0,26109999 |  | 0,340799987 | 0,346500009 | 0,307399988 | 0,310400009 | 0,363900006 0,335900009 | 0,315800011 | 0,314500004 | 0,353199989 | 0,343600005 | 0,280999988 | 0,281699985 | 0,256700009 | 0,256300002 | 0,297899991 | 0,291999996 | 0,259799987 | 0,266799986 | 0,283800006 | 0,288199991 |  |  |
| 200 | 0,259799987 0,26069999 |  | 0,349099994 | 0,355100006 | 0,312299997 | 0,315200001 | 0,373299986 0,344500005 | 0,322899997 | 0,321500003 | 0,361999989 | 0,353799999 | 0,285600007 | 0,285100013 | 0,2579 | 0,258100003 | 0,304100007 | 0,297300011 | 0,262899995 | 0,268700004 | 0,289499998 | 0,291900009 |  |  |
| 220 | 0,257800013 0,26179999 |  | 0,355699986 | 0,363299996 | 0,317699999 | 0,321200013 | 0,382299989 0,350600004 | 0,328399986 | 0,326400012 | 0,371300012 | 0,361400008 | 0,287600011 | 0,288700014 | 0,261099994 | 0,259799987 | 0,308699995 | 0,301699996 | 0,264600009 | 0,269899994 | 0,291599989 | 0,29460001 |  |  |
| 240 | 0,258599997 0,26030001 |  | 0,363999993 | 0,370599985 | 0,32280001 | 0,325899988 | 0,390599996 0,356999993 | 0,332399994 | 0,332199991 | 0,380100012 | 0,369399995 | 0,290300012 | 0,290300012 | 0,261700004 | 0,260699987 | 0,312000006 | 0,305599988 | 0,266200006 | 0,270399988 | 0,2949 | 0,299600005 |  |  |
| 260 | 0,258100003 0,2613 |  | 0,372200012 | 0,378399998 | 0,327399999 | 0,33129999 | 0,401199996 0,36500001 | 0,33919999 | 0,338999987 | 0,390500009 | 0,376300007 | 0,293500006 | 0,29460001 | 0,263900012 | 0,262600005 | 0,319200009 | 0,310299993 | 0,267800003 | 0,274899989 | 0,299100012 | 0,304100007 |  |  |
| 280 | 0,258700013 0,26010001 |  | 0,379599988 | 0,38530001 | 0,331499994 | 0,336400002 | 0,410299987 0,371499985 | 0,343800008 | 0,344399989 | 0,397700012 | 0,385500014 | 0,295100003 | 0,298999995 | 0,265500009 | 0,262600005 | 0,323199987 | 0,313899994 | 0,269899994 | 0,2764 | 0,3037 | 0,306400001 |  |  |
| 300 | 0,258100003 0,25979999 |  | 0,387800008 | 0,39230001 | 0,336499989 | 0,341300011 | 0,416900009 0,377700001 | 0,348500013 | 0,34889999 | 0,406300008 | 0,39289999 | 0,297500014 | 0,300399989 | 0,265399992 | 0,262800008 | 0,326999992 | 0,316799998 | 0,268999994 | 0,277500004 | 0,305700004 | 0,309500009 |  |  |
| 320 | 0,258100003 0,2604 |  | 0,395000011 | 0,399599999 | 0,342299998 | 0,346300006 | 0,426299989 0,384600013 | 0,355199993 | 0,355800003 | 0,414400011 | 0,400099993 | 0,299800009 | 0,303900003 | 0,2676 | 0,264699996 | 0,333200008 | 0,319999993 | 0,268599987 | 0,279199988 | 0,309899986 | 0,313600004 |  |  |
| 340 | 0,256900012 0,25920001 |  | 0,40200001 | 0,407599986 | 0,348500013 | 0,351399988 | 0,43689999 0,393400013 | 0,359699994 | 0,362100005 | 0,423999995 | 0,410100013 | 0,302599996 | 0,307200015 | 0,268999994 | 0,266099989 | 0,338200003 | 0,324999988 | 0,270599991 | 0,281199992 | 0,313300014 | 0,317999989 |  |  |
| 360 | 0,256099999 0,26050001 |  | 0,408300012 | 0,414600015 | 0,352400005 | 0,355599999 | 0,445300013 0,400000006 | 0,36469999 | 0,3662 | 0,431199998 | 0,416200012 | 0,305000007 | 0,309500009 | 0,269600004 | 0,266299993 | 0,341600001 | 0,327699989 | 0,272300005 | 0,281500012 | 0,316300005 | 0,3213 |  |  |
| slope | -1,08152E-05 -8,9422E-06 |  | 0,000410568 | 0,00042581 | 0,000256249 | 0,000270232 | 0,000478199 0,000375526 | 0,000270867 | 0,000284474 | 0,000443333 | 0,000406017 | 0,00014968 | 0,000161115 | 6,49278E-05 | 5,37926E-05 | 0,000234123 | 0,00020646 | 6,49845E-05 | 8,17956E-05 | 0,000167962 | 0,000181367 | #DIV/0! | #DIV/0! |
| slope w/o blank |  |  | 0,000420446 | 0,000435689 | 0,000266127 | 0,000280111 | 0,000488078 0,000385405 | 0,000280746 | 0,000294352 | 0,000453212 | 0,000415895 | 0,000159559 | 0,000170993 | 7,48065E-05 | 6,36713E-05 | 0,000244002 | 0,000216339 | 7,48632E-05 | 9,16744E-05 | 0,000177841 | 0,000191246 | #DIV/0! | #DIV/0! |
|  |  |  |  | 0,000428068 |  | 0,000273119 | 0,000436741 |  | 0,000287549 |  | 0,000434554 |  | 0,000165276 |  | 6,92389E-05 |  | 0,00023017 |  | 8,32688E-05 |  | 0,000184543 | #DIV/0! | #DIV/0! |
|  |  |  |  | 0,025684055 |  | 0,016387149 | 0,026204488 |  | 0,017252939 |  | 0,02607322 |  | 0,009916562 |  | 0,004154333 |  | 0,013810214 |  | 0,004996127 |  | 0,011072599 | #DIV/0! | #DIV/0! |
|  |  |  |  | 3,833441041 |  | 2,445843203 | 3,911117542 |  | 2,575065479 |  | 3,891525335 |  | 1,480083908 |  | 0,620049643 |  | 2,061226041 |  | 0,745690621 |  | 1,652626781 | #DIV/0! | #DIV/0! |
|  |  |  |  | 27,38172172 |  | 17,47030859 | 27,93655387 |  | 18,39332485 |  | 27,79660954 |  | 10,57202791 |  | 4,428926018 |  | 14,72304315 |  | 5,326361575 |  | 11,80447701 | #DIV/0! | #DIV/0! |
|  |  | **min -1** |  | 27,38172172 |  | 17,47030859 | 27,93655387 |  | 18,39332485 |  | 27,79660954 |  | 10,57202791 |  | 4,428926018 |  | 14,72304315 |  | 5,326361575 |  | 11,80447701 | #DIV/0! | #DIV/0! |

|  | **5 min** | 30 |
| --- | --- | --- |
| **WTPTPN22 ctr** | 27,3817217 | 76,05790021 |
| **WTPTPN22reduced** | 17,4703086 | 4,469147798 |
| **WTPTPN22+TrxR1+NADPH** | 27,9365539 | 97,26724967 |
| **WTPTPN22+TrxR1+NADPH+Trx1** | 18,3933249 | 31,22145889 |
| **WTPTPN22+TrxR1+NADPH+Trp14** | 27,7966095 |  |
| **mut PTPN22 ctr** | 10,5720279 |  |
| **mut PTPN22reduced** | 4,42892602 |  |
| **mut PTPN22+TrxR1+NADPH** | 14,7230431 |  |
| **mut PTPN22+TrxR1+NADPH+Trx1** | 5,32636158 |  |
| **mut PTPN22+TrxR1+NADPH+Trp14** | 11,804477 |  |

Protocol Layout:

- 20mM Hepes Buffer 0,1mM DTPA, pH 7.4, 10min
- Wash 1ml spinn collumn slurry 5x with Hepes Buffer
- Spinn 1000 rpm for 2 min, apply 50μl PTP1B from Freezer (recombinant HishPTP1B in 20mM Hepes, 1mM TCEP), ( desalt 1x)
- Measure PTP1B (reduced PTP) and protein concentration with Bradford -> (0,585/1,16) X5 -> - 0,5 μg/μl
- Prepare 16.7mM (15mM end concentration) pNPP (Sigma) substrate sollution Mw 371

**PTP1B activity catalytic domain exposed to 50μM H2O2 and 25mM Bicarbonat and TrxR1, Trx, Prx2**

- prepare 20μl PTP1B, 600nM
- add 20μl from each condition in duplicates to 96-well plate
- Add 180μl substrate solution


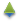

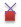

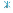

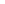


30

25

WTPTPN22 ctr

20

#REF!

15

WTPTPN22reduced

10

WTPTPN22+TrxR1+NADP H

WTPTPN22+TrxR1+NADP

H+Trx1

5

0

0

0,5

1

1,5

| Application: Tecan i-control  Device: infinite 200Pro | | Tecan i-control , 2.0.10.0  Serial number: 1307001123 | Serial number of connected stacker: |
| --- | --- | --- | --- |
| Firmware: V_3.40_01/15_Infinite (Dec 23 201 MAI, V_3.40_01/15_Infinite (Dec 23 2014/12.45.11) | | | |
| Date: | ######### | | |
| Time: | 14:41:20 | | |

System MTC-MU059-S

User MTC-MU059-S\fretho

Plate Greiner 96 Flat Bottom Transparent Polystyrene Cat. No.: 655101/655161/655192 [GRE96ft.pdfx] Plate-ID (Stacker)

List of actions in this measurement script: Kinetic

Absorbance

Shaking (Linear) Duration: Shaking (Linear) Amplitude:

3 s

1 mm

Label: Label1

Kinetic Measurement

Kinetic duration 00:06:00

Interval Time 00:00:20

Measurement Wavelength 405 nm

Bandwidth 10 nm

Number of Flashes 5

Settle Time 0 ms

Part of Plate C1-C12; D1-D10

Start Time: 2020 08-04 14:41:23

| Cycle Nr. | 1 | 2 | 3 | 4 | 5 | 6 | 7 | 8 | 9 | 10 | 11 | 12 | 13 | 14 | 15 | 16 | 17 | 18 | 19 |
| --- | --- | --- | --- | --- | --- | --- | --- | --- | --- | --- | --- | --- | --- | --- | --- | --- | --- | --- | --- |
| Time [s] | 0 | 20 | 40 | 60 | 80 | 100 | 120 | 140 | 160 | 180 | 200 | 220 | 240 | 260 | 280 | 300 | 320 | 340 | 360 |
| Temp. [°C] | 25,5 | 25,3 | 25,5 | 25,5 | 25,7 | 25,4 | 25,2 | 25,5 | 25,4 | 25,6 | 25,4 | 25,3 | 25,5 | 25,5 | 25,4 | 25,5 | 25,2 | 25,4 | 25,3 |
| C1 | 0,2714 | 0,274 | 0,2785 | 0,2847 | 0,2939 | 0,3043 | 0,3167 | 0,3223 | 0,3319 | 0,3408 | 0,3491 | 0,3557 | 0,364 | 0,3722 | 0,3796 | 0,3878 | 0,395 | 0,402 | 0,4083 |
| C2 | 0,2677 | 0,2768 | 0,2787 | 0,2859 | 0,2956 | 0,3061 | 0,3178 | 0,3278 | 0,3384 | 0,3465 | 0,3551 | 0,3633 | 0,3706 | 0,3784 | 0,3853 | 0,3923 | 0,3996 | 0,4076 | 0,4146 |
| C3 | 0,2588 | 0,2661 | 0,2717 | 0,2741 | 0,2806 | 0,2853 | 0,2921 | 0,2968 | 0,3018 | 0,3074 | 0,3123 | 0,3177 | 0,3228 | 0,3274 | 0,3315 | 0,3365 | 0,3423 | 0,3485 | 0,3524 |
| C4 | 0,2632 | 0,2675 | 0,2693 | 0,2737 | 0,28 | 0,2872 | 0,2939 | 0,2993 | 0,3054 | 0,3104 | 0,3152 | 0,3212 | 0,3259 | 0,3313 | 0,3364 | 0,3413 | 0,3463 | 0,3514 | 0,3556 |
| C5 | 0,2727 | 0,2814 | 0,2919 | 0,3014 | 0,3132 | 0,3252 | 0,3364 | 0,3444 | 0,355 | 0,3639 | 0,3733 | 0,3823 | 0,3906 | 0,4012 | 0,4103 | 0,4169 | 0,4263 | 0,4369 | 0,4453 |
| C6 | 0,2666 | 0,2737 | 0,2781 | 0,2859 | 0,2955 | 0,3048 | 0,3141 | 0,3209 | 0,3279 | 0,3359 | 0,3445 | 0,3506 | 0,357 | 0,365 | 0,3715 | 0,3777 | 0,3846 | 0,3934 | 0,4 |
| C7 | 0,2685 | 0,2752 | 0,2782 | 0,2828 | 0,2884 | 0,2941 | 0,3008 | 0,3062 | 0,3121 | 0,3158 | 0,3229 | 0,3284 | 0,3324 | 0,3392 | 0,3438 | 0,3485 | 0,3552 | 0,3597 | 0,3647 |
| C8 | 0,2677 | 0,2723 | 0,2764 | 0,2804 | 0,2854 | 0,2919 | 0,2993 | 0,3036 | 0,309 | 0,3145 | 0,3215 | 0,3264 | 0,3322 | 0,339 | 0,3444 | 0,3489 | 0,3558 | 0,3621 | 0,3662 |
| C9 | 0,2716 | 0,283 | 0,2896 | 0,2972 | 0,309 | 0,3189 | 0,327 | 0,3357 | 0,3453 | 0,3532 | 0,362 | 0,3713 | 0,3801 | 0,3905 | 0,3977 | 0,4063 | 0,4144 | 0,424 | 0,4312 |
| C10 | 0,2689 | 0,2816 | 0,2865 | 0,2947 | 0,3019 | 0,311 | 0,3203 | 0,3285 | 0,3354 | 0,3436 | 0,3538 | 0,3614 | 0,3694 | 0,3763 | 0,3855 | 0,3929 | 0,4001 | 0,4101 | 0,4162 |
| C11 | 0,2554 | 0,257 | 0,2587 | 0,261 | 0,2627 | 0,2657 | 0,2711 | 0,2738 | 0,2774 | 0,281 | 0,2856 | 0,2876 | 0,2903 | 0,2935 | 0,2951 | 0,2975 | 0,2998 | 0,3026 | 0,305 |
| C12 | 0,2551 | 0,2579 | 0,2583 | 0,2601 | 0,2639 | 0,2682 | 0,2733 | 0,2745 | 0,278 | 0,2817 | 0,2851 | 0,2887 | 0,2903 | 0,2946 | 0,299 | 0,3004 | 0,3039 | 0,3072 | 0,3095 |
| D1 | 0,2501 | 0,2513 | 0,2501 | 0,2488 | 0,2494 | 0,2528 | 0,2541 | 0,2534 | 0,2552 | 0,2567 | 0,2579 | 0,2611 | 0,2617 | 0,2639 | 0,2655 | 0,2654 | 0,2676 | 0,269 | 0,2696 |
| D2 | 0,2519 | 0,2531 | 0,251 | 0,2483 | 0,2492 | 0,2502 | 0,2531 | 0,2538 | 0,255 | 0,2563 | 0,2581 | 0,2598 | 0,2607 | 0,2626 | 0,2626 | 0,2628 | 0,2647 | 0,2661 | 0,2663 |
| D3 | 0,2566 | 0,2654 | 0,266 | 0,2697 | 0,2764 | 0,2795 | 0,287 | 0,2905 | 0,2939 | 0,2979 | 0,3041 | 0,3087 | 0,312 | 0,3192 | 0,3232 | 0,327 | 0,3332 | 0,3382 | 0,3416 |
| D4 | 0,2573 | 0,2604 | 0,2617 | 0,2647 | 0,2715 | 0,2759 | 0,2813 | 0,2843 | 0,2905 | 0,292 | 0,2973 | 0,3017 | 0,3056 | 0,3103 | 0,3139 | 0,3168 | 0,32 | 0,325 | 0,3277 |
| D5 | 0,2514 | 0,2542 | 0,2512 | 0,252 | 0,2526 | 0,2564 | 0,2565 | 0,2571 | 0,2595 | 0,2598 | 0,2629 | 0,2646 | 0,2662 | 0,2678 | 0,2699 | 0,269 | 0,2686 | 0,2706 | 0,2723 |
| D6 | 0,256 | 0,2587 | 0,2554 | 0,2564 | 0,2577 | 0,2592 | 0,2622 | 0,263 | 0,2653 | 0,2668 | 0,2687 | 0,2699 | 0,2704 | 0,2749 | 0,2764 | 0,2775 | 0,2792 | 0,2812 | 0,2815 |
| D7 | 0,2553 | 0,2609 | 0,2625 | 0,2655 | 0,2682 | 0,2719 | 0,2767 | 0,2782 | 0,2818 | 0,2838 | 0,2895 | 0,2916 | 0,2949 | 0,2991 | 0,3037 | 0,3057 | 0,3099 | 0,3133 | 0,3163 |
| D8 | 0,2557 | 0,2612 | 0,263 | 0,2668 | 0,2699 | 0,2723 | 0,2779 | 0,2801 | 0,2837 | 0,2882 | 0,2919 | 0,2946 | 0,2996 | 0,3041 | 0,3064 | 0,3095 | 0,3136 | 0,318 | 0,3213 |
| D9 | 0,2613 | 0,2618 | 0,2603 | 0,2591 | 0,2589 | 0,2598 | 0,2609 | 0,2598 | 0,2598 | 0,2582 | 0,2598 | 0,2578 | 0,2586 | 0,2581 | 0,2587 | 0,2581 | 0,2581 | 0,2569 | 0,2561 |
| D10 | 0,2635 | 0,2631 | 0,262 | 0,2621 | 0,261 | 0,2622 | 0,264 | 0,2626 | 0,2625 | 0,2611 | 0,2607 | 0,2618 | 0,2603 | 0,2613 | 0,2601 | 0,2598 | 0,2604 | 0,2592 | 0,2605 |

**WTPTPN22 ctr WTPTPN22 ctr WTPTPN22reduced WTPTPN22reduced WTPTPN22+TrxR1+NADPH WTPTPN22+TrxR1+NADPH WTPTPN22+TrxR1+NADPH+Trx WTPTPN22+TrxR1+NADPH+Trx1 WTPTPN22+TrxR1+NADPH+Trp14 WTPTPN22+TrxR1+NADPH+Trp14 mut PTPN22 ctr mut PTPN22 ctr mut PTPN22reduced mut PTPN22reduced mut PTPN22+TrxR1+NADPH mut PTPN22+TrxR1+NADPH mut PTPN22+TrxR1+Nmut PTPN22+TrxR1+Nmut PTPN22+TrxR1+NA mut PTPN22+TrxR1+NADPH+Trp14**

**Time (sec) Blank Blank**

20 0,258700013 0,2608 0,271600008 0,268700004 0,245700002 0,247899994 0,261700004 0,25909999 0,255199999 0,256900012 0,260100007 0,257400006 0,251800001 0,252999991 0,242799997 0,242300004 0,245900005 0,246700004 0,252400011 0,249899998 0,248999998 0,247500002

40 0,2579 0,26199999 0,280400008 0,273099989 0,244000003 0,248699993 0,266900003 0,262600005 0,258399993 0,260100007 0,266200006 0,262100011 0,256099999 0,254900008 0,242599994 0,240500003 0,250999987 0,25060001 0,260399997 0,253899992 0,250999987 0,248899996

60 0,256000012 0,2617 0,289499998 0,279000014 0,2447 0,2491 0,271800011 0,26730001 0,25909999 0,261099994 0,26910001 0,267100006 0,256799996 0,257499993 0,241899997 0,240400001 0,253800005 0,253399998 0,250699997 0,251100004 0,251700014 0,251700014

80 0,258100003 0,2604 0,305799991 0,289499998 0,246600002 0,252200007 0,281100005 0,274500012 0,261200011 0,264200002 0,274300009 0,271800011 0,260899991 0,261400014 0,240700006 0,241999999 0,258899987 0,257600009 0,252400011 0,254500002 0,254999995 0,256099999

100 0,257200003 0,25819999 0,312700003 0,296600014 0,249200001 0,255299985 0,284799993 0,280400008 0,268400013 0,26789999 0,278699994 0,27669999 0,263999999 0,263799995 0,240899995 0,241699994 0,262800008 0,260500014 0,252200007 0,252400011 0,259000003 0,2588

120 0,257200003 0,2579 0,323700011 0,304199994 0,251800001 0,257999986 0,293099999 0,28670001 0,266499996 0,267500013 0,281300008 0,280299991 0,26699999 0,266900003 0,241300002 0,240700006 0,2676 0,264600009 0,252999991 0,252999991 0,258899987 0,260100007

140 0,257999986 0,2613 0,335799992 0,31400001 0,254599988 0,262199998 0,299299985 0,294800013 0,269600004 0,273099989 0,287099987 0,287 0,271899998 0,271699995 0,243599996 0,243200004 0,271699995 0,270099998 0,254700005 0,255800009 0,262100011 0,263799995

160 0,256799996 0,25749999 0,344399989 0,321500003 0,255899996 0,262699991 0,306100011 0,299499989 0,270200014 0,273900002 0,290100008 0,288500011 0,274899989 0,2755 0,241999999 0,243799999 0,274199992 0,273999989 0,254700005 0,256000012 0,263900012 0,264999986

180 0,25819999 0,26010001 0,354499996 0,328999996 0,257999986 0,265300006 0,310699999 0,304800004 0,273699999 0,275099993 0,294400007 0,291799992 0,278400004 0,27790001 0,243900001 0,244299993 0,277099997 0,274899989 0,255699992 0,257099986 0,266600013 0,266299993

200 0,256300002 0,25709999 0,364100009 0,335700005 0,258599997 0,267399997 0,315600008 0,310200006 0,27610001 0,278200001 0,297199994 0,297199994 0,279300004 0,280699998 0,2421 0,244800001 0,281100005 0,277099997 0,256099999 0,256700009 0,266600013 0,26699999

220 0,257099986 0,2588 0,374000013 0,344199985 0,259799987 0,26789999 0,320800006 0,316199988 0,278899997 0,281300008 0,302700013 0,30219999 0,284299999 0,284000009 0,242400005 0,244000003 0,284999996 0,280499995 0,257600009 0,25819999 0,270900011 0,271499991

240 0,257800013 0,2579 0,384499997 0,353199989 0,262899995 0,271699995 0,328500003 0,322100013 0,282099992 0,284299999 0,306600004 0,307099998 0,286000013 0,287 0,242699996 0,243100002 0,288599998 0,285299987 0,257999986 0,260600001 0,271400005 0,272700012

260 0,256799996 0,25909999 0,394199997 0,361499995 0,264499992 0,274500012 0,334300011 0,327600002 0,284200013 0,287400007 0,312000006 0,311399996 0,289799988 0,289999992 0,243499994 0,244100004 0,292499989 0,287 0,259799987 0,261999995 0,276300013 0,275999993

280 0,2579 0,259 0,403299987 0,367399991 0,266200006 0,2755 0,338800013 0,332500011 0,28670001 0,287800014 0,313699991 0,315400004 0,292899996 0,292899996 0,243499994 0,244100004 0,2949 0,291200012 0,261099994 0,261900008 0,277500004 0,277500004

300 0,258300006 0,25940001 0,413800001 0,377099991 0,266900003 0,279700011 0,345400006 0,338400006 0,288899988 0,290800005 0,318500012 0,319000006 0,2949 0,295100003 0,244100004 0,245100006 0,299699992 0,293099999 0,262699991 0,263799995 0,279900014 0,278600007

320 0,256500006 0,2588 0,422600001 0,38319999 0,26910001 0,279799998 0,351700008 0,343899995 0,292499989 0,293500006 0,322100013 0,324200004 0,297500014 0,297699988 0,244200006 0,245499998 0,302700013 0,29550001 0,260600001 0,263700008 0,279799998 0,280499995

340 0,254999995 0,25909999 0,431199998 0,391099989 0,270200014 0,281300008 0,356400013 0,349599987 0,295700014 0,294999987 0,324400008 0,328200012 0,299400002 0,299299985 0,243300006 0,243799999 0,305099994 0,299199998 0,263099998 0,265899986 0,282799989 0,282299995

360 0,257499993 0,2581 0,442499995 0,398099989 0,270700008 0,283199996 0,363200009 0,353799999 0,298500001 0,298200011 0,331400007 0,333999991 0,304100007 0,303600013 0,244200006 0,245800003 0,308699995 0,303799987 0,265100002 0,266099989 0,2852 0,286300004

slope -2,77092E-06 -7,1517E-06 0,000502394 0,000392007 8,53561E-05 0,000111104 0,000298421 0,000286935 0,000124567 0,000120697 0,000202699 0,000220237 0,000152786 0,00015211 6,98144E-06 1,31785E-05 0,00018306 0,000163483 3,36274E-05 4,64499E-05 0,000107709 0,000109742

slope w/o blank

0,000507356 0,000396968 9,03174E-05 0,000116066 0,000303382 0,000291896 0,000129528 0,000125658 0,00020766 0,000225199 0,000157748 0,000157072 1,19428E-05 1,81398E-05 0,000188021 0,000168444 3,85887E-05 5,14112E-05 0,00011267 0,000114703

0,000452162 0,000103191 0,000297639 0,000127593 0,000216429 0,00015741 1,50413E-05 0,000178233 4,5E-05 0,000113687

0,02712972 0,006191488 0,01785836 0,007655574 0,012985759 0,009444583 0,000902478 0,010693963 0,002699999 0,006821208

4,049211914 0,924102703 2,665426848 1,142622927 1,938172973 1,40963928 0,134698214 1,596113816 0,402984959 1,018090764

28,92294224 6,600733596 19,0387632 8,161592336 13,84409267 10,068852 0,962130099 11,40081297 2,878463993 7,272076884

**min -1** 28,92294224 6,600733596 19,0387632 8,161592336 13,84409267 10,068852 0,962130099 11,40081297 2,878463993 7,272076884

**5 min** 30

**WTPTPN22 ctr** 28,9229422 76,05790021

**WTPTPN22reduced** 6,6007336 4,469147798

**WTPTPN22+TrxR1+NADPH** 19,0387632 97,26724967

**WTPTPN22+TrxR1+NADPH+Trx1** 8,16159234 31,22145889

**WTPTPN22+TrxR1+NADPH+Trp14** 13,8440927

**mut PTPN22 ctr** 10,068852

**mut PTPN22reduced** 0,9621301

**mut PTPN22+TrxR1+NADPH** 11,400813

**mut PTPN22+TrxR1+NADPH+Trx1** 2,87846399

**mut PTPN22+TrxR1+NADPH+Trp14** 7,27207688

| Application: Tecan i-control  Device: infinite 200Pro | | Tecan i-control , 2.0.10.0  Serial number: 1307001123 | Serial number of connected stacker: |
| --- | --- | --- | --- |
| Firmware: V_3.40_01/15_Infinite (Dec 23 201 MAI, V_3.40_01/15_Infinite (Dec 23 2014/12.45.11) | | | |
| Date: | ######### | | |
| Time: | 14:51:44 | | |

System MTC-MU059-S

User MTC-MU059-S\fretho

Plate Greiner 96 Flat Bottom Transparent Polystyrene Cat. No.: 655101/655161/655192 [GRE96ft.pdfx] Plate-ID (Stacker)

List of actions in this measurement script: Kinetic

Absorbance

Shaking (Linear) Duration: Shaking (Linear) Amplitude:

3 s

1 mm

Label: Label1

Kinetic Measurement

Kinetic duration 00:06:00

Interval Time 00:00:20

Measurement Wavelength 405 nm

Bandwidth 10 nm

Number of Flashes 5

Settle Time 0 ms

Part of Plate C1-C12; D1-D10

Start Time: 2020 08-04 14:51:46

| Cycle Nr. | 1 | 2 | 3 | 4 | 5 | 6 | 7 | 8 | 9 | 10 | 11 | 12 | 13 | 14 | 15 | 16 | 17 | 18 | 19 |
| --- | --- | --- | --- | --- | --- | --- | --- | --- | --- | --- | --- | --- | --- | --- | --- | --- | --- | --- | --- |
| Time [s] | 0 | 20 | 40 | 60 | 80 | 100 | 120 | 140 | 160 | 180 | 200 | 220 | 240 | 260 | 280 | 300 | 320 | 340 | 360 |
| Temp. [°C] | 25,6 | 25,7 | 25,6 | 25,8 | 25,9 | 25,6 | 25,6 | 25,7 | 25,9 | 25,9 | 25,7 | 25,7 | 25,8 | 25,8 | 25,8 | 25,9 | 25,8 | 25,9 | 25,9 |
| C1 | 0,266 | 0,2716 | 0,2804 | 0,2895 | 0,3058 | 0,3127 | 0,3237 | 0,3358 | 0,3444 | 0,3545 | 0,3641 | 0,374 | 0,3845 | 0,3942 | 0,4033 | 0,4138 | 0,4226 | 0,4312 | 0,4425 |
| C2 | 0,2619 | 0,2687 | 0,2731 | 0,279 | 0,2895 | 0,2966 | 0,3042 | 0,314 | 0,3215 | 0,329 | 0,3357 | 0,3442 | 0,3532 | 0,3615 | 0,3674 | 0,3771 | 0,3832 | 0,3911 | 0,3981 |
| C3 | 0,2436 | 0,2457 | 0,244 | 0,2447 | 0,2466 | 0,2492 | 0,2518 | 0,2546 | 0,2559 | 0,258 | 0,2586 | 0,2598 | 0,2629 | 0,2645 | 0,2662 | 0,2669 | 0,2691 | 0,2702 | 0,2707 |
| C4 | 0,2468 | 0,2479 | 0,2487 | 0,2491 | 0,2522 | 0,2553 | 0,258 | 0,2622 | 0,2627 | 0,2653 | 0,2674 | 0,2679 | 0,2717 | 0,2745 | 0,2755 | 0,2797 | 0,2798 | 0,2813 | 0,2832 |
| C5 | 0,2572 | 0,2617 | 0,2669 | 0,2718 | 0,2811 | 0,2848 | 0,2931 | 0,2993 | 0,3061 | 0,3107 | 0,3156 | 0,3208 | 0,3285 | 0,3343 | 0,3388 | 0,3454 | 0,3517 | 0,3564 | 0,3632 |
| C6 | 0,2549 | 0,2591 | 0,2626 | 0,2673 | 0,2745 | 0,2804 | 0,2867 | 0,2948 | 0,2995 | 0,3048 | 0,3102 | 0,3162 | 0,3221 | 0,3276 | 0,3325 | 0,3384 | 0,3439 | 0,3496 | 0,3538 |
| C7 | 0,2519 | 0,2552 | 0,2584 | 0,2591 | 0,2612 | 0,2684 | 0,2665 | 0,2696 | 0,2702 | 0,2737 | 0,2761 | 0,2789 | 0,2821 | 0,2842 | 0,2867 | 0,2889 | 0,2925 | 0,2957 | 0,2985 |
| C8 | 0,2533 | 0,2569 | 0,2601 | 0,2611 | 0,2642 | 0,2679 | 0,2675 | 0,2731 | 0,2739 | 0,2751 | 0,2782 | 0,2813 | 0,2843 | 0,2874 | 0,2878 | 0,2908 | 0,2935 | 0,295 | 0,2982 |
| C9 | 0,2531 | 0,2601 | 0,2662 | 0,2691 | 0,2743 | 0,2787 | 0,2813 | 0,2871 | 0,2901 | 0,2944 | 0,2972 | 0,3027 | 0,3066 | 0,312 | 0,3137 | 0,3185 | 0,3221 | 0,3244 | 0,3314 |
| C10 | 0,2544 | 0,2574 | 0,2621 | 0,2671 | 0,2718 | 0,2767 | 0,2803 | 0,287 | 0,2885 | 0,2918 | 0,2972 | 0,3022 | 0,3071 | 0,3114 | 0,3154 | 0,319 | 0,3242 | 0,3282 | 0,334 |
| C11 | 0,2503 | 0,2518 | 0,2561 | 0,2568 | 0,2609 | 0,264 | 0,267 | 0,2719 | 0,2749 | 0,2784 | 0,2793 | 0,2843 | 0,286 | 0,2898 | 0,2929 | 0,2949 | 0,2975 | 0,2994 | 0,3041 |
| C12 | 0,2502 | 0,253 | 0,2549 | 0,2575 | 0,2614 | 0,2638 | 0,2669 | 0,2717 | 0,2755 | 0,2779 | 0,2807 | 0,284 | 0,287 | 0,29 | 0,2929 | 0,2951 | 0,2977 | 0,2993 | 0,3036 |
| D1 | 0,2484 | 0,2428 | 0,2426 | 0,2419 | 0,2407 | 0,2409 | 0,2413 | 0,2436 | 0,242 | 0,2439 | 0,2421 | 0,2424 | 0,2427 | 0,2435 | 0,2435 | 0,2441 | 0,2442 | 0,2433 | 0,2442 |
| D2 | 0,2446 | 0,2423 | 0,2405 | 0,2404 | 0,242 | 0,2417 | 0,2407 | 0,2432 | 0,2438 | 0,2443 | 0,2448 | 0,244 | 0,2431 | 0,2441 | 0,2441 | 0,2451 | 0,2455 | 0,2438 | 0,2458 |
| D3 | 0,2448 | 0,2459 | 0,251 | 0,2538 | 0,2589 | 0,2628 | 0,2676 | 0,2717 | 0,2742 | 0,2771 | 0,2811 | 0,285 | 0,2886 | 0,2925 | 0,2949 | 0,2997 | 0,3027 | 0,3051 | 0,3087 |
| D4 | 0,2446 | 0,2467 | 0,2506 | 0,2534 | 0,2576 | 0,2605 | 0,2646 | 0,2701 | 0,274 | 0,2749 | 0,2771 | 0,2805 | 0,2853 | 0,287 | 0,2912 | 0,2931 | 0,2955 | 0,2992 | 0,3038 |
| D5 | 0,2493 | 0,2524 | 0,2604 | 0,2507 | 0,2524 | 0,2522 | 0,253 | 0,2547 | 0,2547 | 0,2557 | 0,2561 | 0,2576 | 0,258 | 0,2598 | 0,2611 | 0,2627 | 0,2606 | 0,2631 | 0,2651 |
| D6 | 0,2483 | 0,2499 | 0,2539 | 0,2511 | 0,2545 | 0,2524 | 0,253 | 0,2558 | 0,256 | 0,2571 | 0,2567 | 0,2582 | 0,2606 | 0,262 | 0,2619 | 0,2638 | 0,2637 | 0,2659 | 0,2661 |
| D7 | 0,2464 | 0,249 | 0,251 | 0,2517 | 0,255 | 0,259 | 0,2589 | 0,2621 | 0,2639 | 0,2666 | 0,2666 | 0,2709 | 0,2714 | 0,2763 | 0,2775 | 0,2799 | 0,2798 | 0,2828 | 0,2852 |
| D8 | 0,2441 | 0,2475 | 0,2489 | 0,2517 | 0,2561 | 0,2588 | 0,2601 | 0,2638 | 0,265 | 0,2663 | 0,267 | 0,2715 | 0,2727 | 0,276 | 0,2775 | 0,2786 | 0,2805 | 0,2823 | 0,2863 |
| D9 | 0,2576 | 0,2587 | 0,2579 | 0,256 | 0,2581 | 0,2572 | 0,2572 | 0,258 | 0,2568 | 0,2582 | 0,2563 | 0,2571 | 0,2578 | 0,2568 | 0,2579 | 0,2583 | 0,2565 | 0,255 | 0,2575 |
| D10 | 0,2609 | 0,2608 | 0,262 | 0,2617 | 0,2604 | 0,2582 | 0,2579 | 0,2613 | 0,2575 | 0,2601 | 0,2571 | 0,2588 | 0,2579 | 0,2591 | 0,259 | 0,2594 | 0,2588 | 0,2591 | 0,2581 |

End Time: 2020 08-04 14:57:59

**WTPTPN22 ctr WTPTPN22 ctr WTPTPN22reduced WTPTPN22reduced WTPTPN22+TrxR1+NADPH WTPTPN22+TrxR1+NADPH WTPTPN22+TrxR1+NADPH+Tr WTPTPN22+TrxR1+NADPH+ WTPTPN22+TrxR1+NADPH+Trp14 WTPTPN22+TrxR1+NADPH+Trp14 mut PTPN22 ctr mut PTPN22 ctr mut PTPN22reduced mut PTPN22reduced mut PTPN22+TrxR1+NADPH mut PTPN22+TrxR1+NADPH mut PTPN22+TrxR1+Nmut PTPN22+TrxR1+Nmut PTPN22+TrxR1+NA mut PTPN22+TrxR1+NADPH+Trp14**

**Time (sec) Blank Blank**

20 0,051100001 0,0529 0,329100013 0,321399987 0,248999998 0,251599997 0,291700006 0,290600002 0,2588 0,258899987 0,273200005 0,268700004 0,281100005 0,280000001 0,244399995 0,244399995 0,261299998 0,261299998 0,260399997 0,256900012 0,237200007 0,201100007

40 0,051800001 0,0516 0,332800001 0,325800002 0,249599993 0,251300007 0,29429999 0,293500006 0,25940001 0,257400006 0,274699986 0,272000015 0,283300012 0,282799989 0,243300006 0,244399995 0,261900008 0,260600001 0,257099986 0,258599997 0,235799998 0,204500005

60 0,050799999 0,0515 0,342500001 0,334199995 0,251100004 0,252400011 0,298400015 0,2958 0,25850001 0,258300006 0,277500004 0,273600012 0,288399994 0,28580001 0,243200004 0,245399997 0,26820001 0,262499988 0,257299989 0,251599997 0,2368 0,207100004

80 0,050799999 0,0531 0,351000011 0,342999995 0,253100008 0,253199995 0,302100003 0,300799996 0,259200007 0,257999986 0,279799998 0,276199996 0,291500002 0,288800001 0,242599994 0,246199995 0,271699995 0,265700012 0,257200003 0,252400011 0,238399997 0,206

100 0,051199999 0,0544 0,359499991 0,350699991 0,253199995 0,256500006 0,307599992 0,305599988 0,261500001 0,258700013 0,282700002 0,278100014 0,29429999 0,290800005 0,241699994 0,246199995 0,274500012 0,26789999 0,256900012 0,252099991 0,240099996 0,208000004

120 0,0515 0,0577 0,368400007 0,358099997 0,253699988 0,255800009 0,311100006 0,308800012 0,260699987 0,259600013 0,2861 0,279399991 0,298099995 0,29460001 0,243100002 0,245700002 0,27669999 0,269899994 0,256300002 0,252600014 0,240899995 0,209999993

140 0,0528 0,0565 0,377600014 0,366600007 0,256700009 0,255400002 0,314300001 0,313800007 0,261299998 0,259900004 0,28670001 0,282099992 0,301299989 0,298000008 0,242599994 0,2447 0,278699994 0,272899985 0,255299985 0,251199991 0,241300002 0,208700001

160 0,052299999 0,0571 0,387100011 0,374500006 0,258100003 0,260699987 0,319099993 0,316799998 0,264899999 0,261400014 0,290699989 0,285299987 0,305700004 0,301999986 0,241999999 0,246000007 0,281599998 0,274300009 0,25909999 0,255100012 0,246199995 0,211700007

180 0,050500002 0,0562 0,394400001 0,381700009 0,257800013 0,259200007 0,323199987 0,321399987 0,263000011 0,263200015 0,292299986 0,28639999 0,307000011 0,304199994 0,242699996 0,246199995 0,283899993 0,27700001 0,257800013 0,254299998 0,243599996 0,211700007

200 0,052000001 0,0563 0,401300013 0,390100002 0,256599993 0,260399997 0,325700015 0,325100005 0,264699996 0,262899995 0,29550001 0,288800001 0,310699999 0,308600008 0,243499994 0,245199993 0,286500007 0,279000014 0,257999986 0,252499998 0,245499998 0,214499995

220 0,049400002 0,0564 0,408699989 0,397199988 0,257600009 0,260899991 0,330500007 0,328900009 0,263999999 0,263099998 0,297800004 0,289799988 0,31220001 0,309500009 0,240899995 0,244599998 0,287299991 0,280800015 0,256099999 0,252900004 0,243900001 0,214599997

240 0,052700002 0,0565 0,418099999 0,405499995 0,259200007 0,261599988 0,335299999 0,333400011 0,266499996 0,263999999 0,301400006 0,292400002 0,316700011 0,313699991 0,241799995 0,245199993 0,291700006 0,284399986 0,257600009 0,253800005 0,247500002 0,215000004

260 0,0517 0,0562 0,426499993 0,411799997 0,25850001 0,262400001 0,3398 0,337000012 0,266900003 0,264999986 0,30250001 0,29460001 0,317200005 0,315100014 0,242699996 0,243799999 0,29370001 0,284099996 0,258899987 0,254799992 0,248500004 0,216000006

280 0,0517 0,056 0,434199989 0,419699997 0,26030001 0,261900008 0,344199985 0,341800004 0,26910001 0,265500009 0,306100011 0,2958 0,322400004 0,3204 0,241699994 0,244200006 0,298599988 0,287099987 0,257600009 0,254200011 0,248799995 0,218600005

300 0,050999999 0,0577 0,442699999 0,428000003 0,260600001 0,264099985 0,34889999 0,346300006 0,269699991 0,266200006 0,307500005 0,299600005 0,325899988 0,326999992 0,242899999 0,246700004 0,301999986 0,289900005 0,25909999 0,255299985 0,251599997 0,219600007

320 0,0517 0,0579 0,450700015 0,434700012 0,260199994 0,264499992 0,350800008 0,350199997 0,270300001 0,26820001 0,310499996 0,301499993 0,327600002 0,325800002 0,241600007 0,245100006 0,301899999 0,291599989 0,259499997 0,254700005 0,250999987 0,216299996

340 0,0517 0,0557 0,458900005 0,442200005 0,262300014 0,265100002 0,354799986 0,355199993 0,271100014 0,26910001 0,313199997 0,304100007 0,330799997 0,3301 0,2412 0,243699998 0,304699987 0,290600002 0,2588 0,255800009 0,251700014 0,217299998

360 0,053800002 0,0584 0,467900008 0,451499999 0,264200002 0,267199993 0,361999989 0,360399991 0,274599999 0,271899998 0,317499995 0,306100011 0,336800009 0,334600002 0,242799997 0,246199995 0,308800012 0,296299994 0,261200011 0,256999999 0,253100008 0,220300004

slope 2,91538E-06 1,5E-05 0,000413808 0,000385299 3,90609E-05 4,52528E-05 0,000204881 0,000205418 4,38493E-05 3,81682E-05 0,000128364 0,000106837 0,000156563 0,000159608 -4,41176E-06 -6,3466E-07 0,0001363 0,000105299 5,97525E-06 3,65324E-06 5,13984E-05 4,99484E-05

slope w/o blank

0,00040485 0,000376342 3,01032E-05 3,62951E-05 0,000195924 0,00019646 3,48916E-05 2,92105E-05 0,000119407 9,78793E-05 0,000147606 0,00015065 -1,33695E-05 -9,59235E-06 0,000127343 9,63416E-05 -2,98244E-06 -5,30445E-06 4,24407E-05 4,09907E-05

0,000390596 3,31992E-05 0,000196192 3,20511E-05 0,000108643 0,000149128 -1,14809E-05 0,000111842 -4,14344E-06 4,17157E-05

0,023435759 0,00199195 0,011771516 0,001923065 0,006518576 0,008947678 -0,000688854 0,006710526 -0,000248606 0,002502941

3,497874465 0,297306008 1,756942677 0,287024658 0,972921788 1,335474339 -0,102814036 1,001570991 -0,037105441 0,373573316

24,98481761 2,123614341 12,54959055 2,050176126 6,949441346 9,539102422 -0,73438597 7,154078504 -0,265038862 2,668380829

**min -1** 24,98481761 2,123614341 12,54959055 2,050176126 6,949441346 9,539102422 -0,73438597 7,154078504 -0,265038862 2,668380829

**5 min** 30

**WTPTPN22 ctr** 24,98482 76,05790021

**WTPTPN22reduced** 2,123614 4,469147798

**WTPTPN22+TrxR1+NADPH** 12,54959 97,26724967

**WTPTPN22+TrxR1+NADPH+Trx1** 2,050176 31,22145889

**WTPTPN22+TrxR1+NADPH+Trp14** 6,949441

**mut PTPN22 ctr** 9,539102

**mut PTPN22reduced** -0,73439

**mut PTPN22+TrxR1+NADPH** 7,154079

**mut PTPN22+TrxR1+NADPH+Trx1** -0,26504

**mut PTPN22+TrxR1+NADPH+Trp14** 2,668381

| Application: Tecan i-control  Device: infinite 200Pro | | Tecan i-control , 2.0.10.0  Serial number: 1307001123 | Serial number of connected stacker: |
| --- | --- | --- | --- |
| Firmware: V_3.40_01/15_Infinite (Dec 23 201 MAI, V_3.40_01/15_Infinite (Dec 23 2014/12.45.11) | | | |
| Date: | ######### | | |
| Time: | 15:08:27 | | |

System MTC-MU059-S

User MTC-MU059-S\fretho

Plate Greiner 96 Flat Bottom Transparent Polystyrene Cat. No.: 655101/655161/655192 [GRE96ft.pdfx] Plate-ID (Stacker)

List of actions in this measurement script: Kinetic

Absorbance

Shaking (Linear) Duration: Shaking (Linear) Amplitude:

3 s

1 mm

Label: Label1

Kinetic Measurement

Kinetic duration 00:06:00

Interval Time 00:00:20

Measurement Wavelength 405 nm

Bandwidth 10 nm

Number of Flashes 5

Settle Time 0 ms

Part of Plate C1-C12; D1-D10

Start Time: 2020 08-04 15:08:29

| Cycle Nr. | 1 | 2 | 3 | 4 | 5 | 6 | 7 | 8 | 9 | 10 | 11 | 12 | 13 | 14 | 15 | 16 | 17 | 18 | 19 |
| --- | --- | --- | --- | --- | --- | --- | --- | --- | --- | --- | --- | --- | --- | --- | --- | --- | --- | --- | --- |
| Time [s] | 0 | 20 | 40 | 60 | 80 | 100 | 120 | 140 | 160 | 180 | 200 | 220 | 240 | 260 | 280 | 300 | 320 | 340 | 360 |
| Temp. [°C] | 26 | 26,1 | 25,9 | 26,1 | 26 | 26,1 | 25,9 | 26,1 | 26 | 26,1 | 26 | 25,8 | 26 | 25,8 | 26 | 26,1 | 26,3 | 25,9 | 26,1 |
| C1 | 0,3246 | 0,3291 | 0,3328 | 0,3425 | 0,351 | 0,3595 | 0,3684 | 0,3776 | 0,3871 | 0,3944 | 0,4013 | 0,4087 | 0,4181 | 0,4265 | 0,4342 | 0,4427 | 0,4507 | 0,4589 | 0,4679 |
| C2 | 0,3172 | 0,3214 | 0,3258 | 0,3342 | 0,343 | 0,3507 | 0,3581 | 0,3666 | 0,3745 | 0,3817 | 0,3901 | 0,3972 | 0,4055 | 0,4118 | 0,4197 | 0,428 | 0,4347 | 0,4422 | 0,4515 |
| C3 | 0,251 | 0,249 | 0,2496 | 0,2511 | 0,2531 | 0,2532 | 0,2537 | 0,2567 | 0,2581 | 0,2578 | 0,2566 | 0,2576 | 0,2592 | 0,2585 | 0,2603 | 0,2606 | 0,2602 | 0,2623 | 0,2642 |
| C4 | 0,2534 | 0,2516 | 0,2513 | 0,2524 | 0,2532 | 0,2565 | 0,2558 | 0,2554 | 0,2607 | 0,2592 | 0,2604 | 0,2609 | 0,2616 | 0,2624 | 0,2619 | 0,2641 | 0,2645 | 0,2651 | 0,2672 |
| C5 | 0,286 | 0,2917 | 0,2943 | 0,2984 | 0,3021 | 0,3076 | 0,3111 | 0,3143 | 0,3191 | 0,3232 | 0,3257 | 0,3305 | 0,3353 | 0,3398 | 0,3442 | 0,3489 | 0,3508 | 0,3548 | 0,362 |
| C6 | 0,2839 | 0,2906 | 0,2935 | 0,2958 | 0,3008 | 0,3056 | 0,3088 | 0,3138 | 0,3168 | 0,3214 | 0,3251 | 0,3289 | 0,3334 | 0,337 | 0,3418 | 0,3463 | 0,3502 | 0,3552 | 0,3604 |
| C7 | 0,26 | 0,2588 | 0,2594 | 0,2585 | 0,2592 | 0,2615 | 0,2607 | 0,2613 | 0,2649 | 0,263 | 0,2647 | 0,264 | 0,2665 | 0,2669 | 0,2691 | 0,2697 | 0,2703 | 0,2711 | 0,2746 |
| C8 | 0,2565 | 0,2589 | 0,2574 | 0,2583 | 0,258 | 0,2587 | 0,2596 | 0,2599 | 0,2614 | 0,2632 | 0,2629 | 0,2631 | 0,264 | 0,265 | 0,2655 | 0,2662 | 0,2682 | 0,2691 | 0,2719 |
| C9 | 0,2709 | 0,2732 | 0,2747 | 0,2775 | 0,2798 | 0,2827 | 0,2861 | 0,2867 | 0,2907 | 0,2923 | 0,2955 | 0,2978 | 0,3014 | 0,3025 | 0,3061 | 0,3075 | 0,3105 | 0,3132 | 0,3175 |
| C10 | 0,2681 | 0,2687 | 0,272 | 0,2736 | 0,2762 | 0,2781 | 0,2794 | 0,2821 | 0,2853 | 0,2864 | 0,2888 | 0,2898 | 0,2924 | 0,2946 | 0,2958 | 0,2996 | 0,3015 | 0,3041 | 0,3061 |
| C11 | 0,2795 | 0,2811 | 0,2833 | 0,2884 | 0,2915 | 0,2943 | 0,2981 | 0,3013 | 0,3057 | 0,307 | 0,3107 | 0,3122 | 0,3167 | 0,3172 | 0,3224 | 0,3259 | 0,3276 | 0,3308 | 0,3368 |
| C12 | 0,278 | 0,28 | 0,2828 | 0,2858 | 0,2888 | 0,2908 | 0,2946 | 0,298 | 0,302 | 0,3042 | 0,3086 | 0,3095 | 0,3137 | 0,3151 | 0,3204 | 0,327 | 0,3258 | 0,3301 | 0,3346 |
| D1 | 0,2456 | 0,2444 | 0,2433 | 0,2432 | 0,2426 | 0,2417 | 0,2431 | 0,2426 | 0,242 | 0,2427 | 0,2435 | 0,2409 | 0,2418 | 0,2427 | 0,2417 | 0,2429 | 0,2416 | 0,2412 | 0,2428 |
| D2 | 0,2455 | 0,2444 | 0,2444 | 0,2454 | 0,2462 | 0,2462 | 0,2457 | 0,2447 | 0,246 | 0,2462 | 0,2452 | 0,2446 | 0,2452 | 0,2438 | 0,2442 | 0,2467 | 0,2451 | 0,2437 | 0,2462 |
| D3 | 0,2584 | 0,2613 | 0,2619 | 0,2682 | 0,2717 | 0,2745 | 0,2767 | 0,2787 | 0,2816 | 0,2839 | 0,2865 | 0,2873 | 0,2917 | 0,2937 | 0,2986 | 0,302 | 0,3019 | 0,3047 | 0,3088 |
| D4 | 0,2619 | 0,2613 | 0,2606 | 0,2625 | 0,2657 | 0,2679 | 0,2699 | 0,2729 | 0,2743 | 0,277 | 0,279 | 0,2808 | 0,2844 | 0,2841 | 0,2871 | 0,2899 | 0,2916 | 0,2906 | 0,2963 |
| D5 | 0,2606 | 0,2604 | 0,2571 | 0,2573 | 0,2572 | 0,2569 | 0,2563 | 0,2553 | 0,2591 | 0,2578 | 0,258 | 0,2561 | 0,2576 | 0,2589 | 0,2576 | 0,2591 | 0,2595 | 0,2588 | 0,2612 |
| D6 | 0,2551 | 0,2569 | 0,2586 | 0,2516 | 0,2524 | 0,2521 | 0,2526 | 0,2512 | 0,2551 | 0,2543 | 0,2525 | 0,2529 | 0,2538 | 0,2548 | 0,2542 | 0,2553 | 0,2547 | 0,2558 | 0,257 |
| D7 | 0,237 | 0,2372 | 0,2358 | 0,2368 | 0,2384 | 0,2401 | 0,2409 | 0,2413 | 0,2462 | 0,2436 | 0,2455 | 0,2439 | 0,2475 | 0,2485 | 0,2488 | 0,2516 | 0,251 | 0,2517 | 0,2531 |
| D8 | 0,2027 | 0,2011 | 0,2045 | 0,2071 | 0,206 | 0,208 | 0,21 | 0,2087 | 0,2117 | 0,2117 | 0,2145 | 0,2146 | 0,215 | 0,216 | 0,2186 | 0,2196 | 0,2163 | 0,2173 | 0,2203 |
| D9 | 0,0524 | 0,0511 | 0,0518 | 0,0508 | 0,0508 | 0,0512 | 0,0515 | 0,0528 | 0,0523 | 0,0505 | 0,052 | 0,0494 | 0,0527 | 0,0517 | 0,0517 | 0,051 | 0,0517 | 0,0517 | 0,0538 |
| D10 | 0,0533 | 0,0529 | 0,0516 | 0,0515 | 0,0531 | 0,0544 | 0,0577 | 0,0565 | 0,0571 | 0,0562 | 0,0563 | 0,0564 | 0,0565 | 0,0562 | 0,056 | 0,0577 | 0,0579 | 0,0557 | 0,0584 |

End Time: 2020 08-04 15:14:42

**WTPTPN22 ctr WTPTPN22 ctr WTPTPN22reduced WTPTPN22reduced WTPTPN22+TrxR1+ WTPTPN22+TrxR WTPTPN22+TrxR1+NAD WTPTPN22+TrxR1+NA WTPTPN22+TrxR1 WTPTPN22+TrxR1+ mut PTPN22 ctr mut PTPN22 ctr mut PTPN22reduced mut PTPN22reduced mut PTPN22+TrxR1+NADPH mut PTPN22+TrxR1+NADPH mut PTPN22+TrxR1+Nmut PTPN22+TrxR1+Nmut PTPN22+TrxR1+NA mut PTPN22+TrxR1+NADPH+Trp14**

| **Time (sec) Blank** | **Blank** |  | | | | | | | | | | | | | | | | | | | |
| --- | --- | --- | --- | --- | --- | --- | --- | --- | --- | --- | --- | --- | --- | --- | --- | --- | --- | --- | --- | --- | --- |
| 20 | 0,270000011 0,273 |  | 0,291999996 | 0,297399998 | 0,282999992 | 0,277500004 | 0,283100009 0,288399994 | 0,278800011 | 0,283600003 | 0,291900009 | 0,291399986 | 0,274399996 | 0,275900006 | 0,257699996 | 0,257699996 | 0,261599988 | 0,261799991 | 0,260600001 | 0,259000003 | 0,268599987 | 0,263200015 |
| 40 | 0,269899994 0,2721 |  | 0,299400002 | 0,30340001 | 0,281199992 | 0,279700011 | 0,287900001 0,294999987 | 0,282999992 | 0,287800014 | 0,300999999 | 0,298099995 | 0,273900002 | 0,277500004 | 0,257200003 | 0,257200003 | 0,264200002 | 0,262100011 | 0,261299998 | 0,261200011 | 0,26820001 | 0,2667 |
| 60 | 0,270700008 0,27360001 |  | 0,312299997 | 0,314700007 | 0,288700014 | 0,287400007 | 0,299199998 0,305799991 | 0,290699989 | 0,294800013 | 0,308999985 | 0,307099998 | 0,27759999 | 0,282099992 | 0,261200011 | 0,261200011 | 0,270900011 | 0,268400013 | 0,263999999 | 0,264600009 | 0,273000002 | 0,272899985 |
| 80 | 0,268299997 0,2714 |  | 0,324200004 | 0,324200004 | 0,292800009 | 0,291399986 | 0,306899995 0,314999998 | 0,295599997 | 0,296600014 | 0,315400004 | 0,313899994 | 0,278800011 | 0,287099987 | 0,261599988 | 0,261599988 | 0,274699986 | 0,271600008 | 0,26789999 | 0,266200006 | 0,275200009 | 0,276600003 |
| 100 | 0,266900003 0,27180001 |  | 0,333799988 | 0,335099995 | 0,299600005 | 0,298799992 | 0,315200001 0,323500007 | 0,300700009 | 0,30219999 | 0,323500007 | 0,323700011 | 0,282599986 | 0,292499989 | 0,262800008 | 0,262800008 | 0,277999997 | 0,274100006 | 0,26879999 | 0,268700004 | 0,280000001 | 0,279799998 |
| 120 | 0,268999994 0,27090001 |  | 0,345699996 | 0,346599996 | 0,305299997 | 0,306699991 | 0,324400008 0,334300011 | 0,305000007 | 0,307399988 | 0,333900005 | 0,334500015 | 0,287 | 0,298000008 | 0,266499996 | 0,266499996 | 0,280800015 | 0,279199988 | 0,272000015 | 0,270900011 | 0,285699993 | 0,28490001 |
| 140 | 0,269499987 0,27079999 |  | 0,356000006 | 0,358399987 | 0,313300014 | 0,311500013 | 0,330000013 0,341199994 | 0,311100006 | 0,3134 | 0,343199998 | 0,342599988 | 0,289600015 | 0,299299985 | 0,268099993 | 0,268099993 | 0,283499986 | 0,281800002 | 0,274199992 | 0,271299988 | 0,288500011 | 0,287299991 |
| 160 | 0,268400013 0,27090001 |  | 0,366299987 | 0,367399991 | 0,318100005 | 0,317000002 | 0,336699992 0,349999994 | 0,314900011 | 0,316799998 | 0,351000011 | 0,351500005 | 0,291700006 | 0,304699987 | 0,267800003 | 0,267800003 | 0,287299991 | 0,284500003 | 0,275599986 | 0,273900002 | 0,293000013 | 0,291399986 |
| 180 | 0,26730001 0,27000001 |  | 0,375800014 | 0,377400011 | 0,3204 | 0,320800006 | 0,343400002 0,35620001 | 0,319900006 | 0,321700007 | 0,359600008 | 0,359800011 | 0,294400007 | 0,307399988 | 0,270300001 | 0,270300001 | 0,289299995 | 0,286000013 | 0,279700011 | 0,276800007 | 0,2949 | 0,294400007 |
| 200 | 0,268299997 0,26989999 |  | 0,385699987 | 0,387699991 | 0,326900005 | 0,325599998 | 0,349999994 0,364300013 | 0,325300008 | 0,327199996 | 0,36590001 | 0,3671 | 0,297500014 | 0,310600013 | 0,272599995 | 0,272599995 | 0,292499989 | 0,289000005 | 0,281199992 | 0,278899997 | 0,298999995 | 0,298299998 |
| 220 | 0,26879999 0,2712 |  | 0,396400005 | 0,399100006 | 0,334600002 | 0,332300007 | 0,357800007 0,372700006 | 0,331900001 | 0,334699988 | 0,375999987 | 0,378199995 | 0,300199986 | 0,314599991 | 0,274800003 | 0,274800003 | 0,296299994 | 0,294200003 | 0,284299999 | 0,282299995 | 0,30340001 | 0,300900012 |
| 240 | 0,269300014 0,27070001 |  | 0,405600011 | 0,408800006 | 0,338699996 | 0,336600006 | 0,363799989 0,381999999 | 0,336199999 | 0,3389 | 0,38499999 | 0,386900008 | 0,303799987 | 0,317600012 | 0,277399987 | 0,277399987 | 0,301699996 | 0,296900004 | 0,287699997 | 0,286000013 | 0,306100011 | 0,304899991 |
| 260 | 0,268000007 0,26899999 |  | 0,415199995 | 0,417800009 | 0,344999999 | 0,339899987 | 0,370900005 0,388000011 | 0,341800004 | 0,343199998 | 0,392199993 | 0,393700004 | 0,305599988 | 0,320300013 | 0,2773 | 0,2773 | 0,302899987 | 0,298900008 | 0,288100004 | 0,286599994 | 0,310400009 | 0,305700004 |
| 280 | 0,268599987 0,26949999 |  | 0,42719999 | 0,427599996 | 0,348399997 | 0,345999986 | 0,378399998 0,398200005 | 0,348199993 | 0,349099994 | 0,400599986 | 0,404199988 | 0,308600008 | 0,324999988 | 0,280900002 | 0,280900002 | 0,307999998 | 0,301499993 | 0,29339999 | 0,290499985 | 0,316000015 | 0,31189999 |
| 300 | 0,26879999 0,271 |  | 0,435699999 | 0,438199997 | 0,355500013 | 0,351500005 | 0,3847 0,403800011 | 0,352800012 | 0,352999985 | 0,407400012 | 0,413500011 | 0,31189999 | 0,328999996 | 0,282200009 | 0,282200009 | 0,310900003 | 0,305599988 | 0,294999987 | 0,292100012 | 0,317400008 | 0,313699991 |
| 320 | 0,267199993 0,2714 |  | 0,445800006 | 0,448000014 | 0,361699998 | 0,3574 | 0,393099993 0,414499998 | 0,35800001 | 0,361299992 | 0,418799996 | 0,422300011 | 0,315400004 | 0,333200008 | 0,28459999 | 0,28459999 | 0,314999998 | 0,309300005 | 0,300799996 | 0,29550001 | 0,3213 | 0,319000006 |
| 340 | 0,270000011 0,2705 |  | 0,45570001 | 0,457800001 | 0,36559999 | 0,360399991 | 0,398799986 0,421099991 | 0,364899993 | 0,365999997 | 0,426899999 | 0,4296 | 0,316700011 | 0,337099999 | 0,285699993 | 0,285699993 | 0,317600012 | 0,313699991 | 0,300999999 | 0,296799988 | 0,322600007 | 0,32280001 |
| 360 | 0,266900003 0,26859999 |  | 0,465799987 | 0,467700005 | 0,371600002 | 0,36590001 | 0,405600011 0,42930001 | 0,367599994 | 0,369899988 | 0,435799986 | 0,43900001 | 0,319400012 | 0,339100003 | 0,287400007 | 0,287400007 | 0,32069999 | 0,314099997 | 0,30250001 | 0,298400015 | 0,324800014 | 0,324999988 |
| slope | -4,21569E-06 -8,5398E-06 |  | 0,000512714 | 0,000510129 | 0,000274045 | 0,000264076 | 0,000358911 0,00041306 | 0,000263633 | 0,000257193 | 0,000421388 | 0,000439768 | 0,000140676 | 0,000189195 | 9,14087E-05 | 9,14087E-05 | 0,000171471 | 0,000157941 | 0,000129696 | 0,00011854 | 0,000179758 | 0,000178246 |
| slope w/o blank |  |  | 0,000519092 | 0,000516507 | 0,000280423 | 0,000270454 | 0,000365289 0,000419438 | 0,00027001 | 0,000263571 | 0,000427766 | 0,000446146 | 0,000147054 | 0,000195573 | 9,77864E-05 | 9,77864E-05 | 0,000177848 | 0,000164319 | 0,000136073 | 0,000124917 | 0,000186135 | 0,000184623 |
|  |  |  |  | 0,000517799 |  | 0,000275439 | 0,000392363 |  | 0,00026679 |  | 0,000436956 |  | 0,000171313 |  | 9,77864E-05 |  | 0,000171084 |  | 0,000130495 |  | 0,000185379 |
|  |  |  |  | 0,031067958 |  | 0,016526317 | 0,023541797 |  | 0,01600743 |  | 0,026217339 |  | 0,010278795 |  | 0,005867184 |  | 0,010265016 |  | 0,007829722 |  | 0,011122757 |
|  |  |  |  | 4,637008726 |  | 2,466614407 | 3,513701108 |  | 2,38916865 |  | 3,913035661 |  | 1,534148496 |  | 0,875699069 |  | 1,532091956 |  | 1,16861528 |  | 1,660113036 |
|  |  |  |  | 33,1214909 |  | 17,61867433 | 25,09786506 |  | 17,06549035 |  | 27,95025472 |  | 10,95820355 |  | 6,25499335 |  | 10,94351397 |  | 8,347251998 |  | 11,85795026 |
|  |  | **min -1** |  | 33,1214909 |  | 17,61867433 | 25,09786506 |  | 17,06549035 |  | 27,95025472 |  | 10,95820355 |  | 6,25499335 |  | 10,94351397 |  | 8,347251998 |  | 11,85795026 |

|  | **5 min** | 30 |
| --- | --- | --- |
| **WTPTPN22 ctr** | 33,1214909 | 76,05790021 |
| **WTPTPN22reduced** | 17,6186743 | 4,469147798 |
| **WTPTPN22+TrxR1+NADPH** | 25,0978651 | 97,26724967 |
| **WTPTPN22+TrxR1+NADPH+Trx1** | 17,0654904 | 31,22145889 |
| **WTPTPN22+TrxR1+NADPH+Trp14** | 27,9502547 |  |
| **mut PTPN22 ctr** | 10,9582035 |  |
| **mut PTPN22reduced** | 6,25499335 |  |
| **mut PTPN22+TrxR1+NADPH** | 10,943514 |  |
| **mut PTPN22+TrxR1+NADPH+Trx1** | 8,347252 |  |
| **mut PTPN22+TrxR1+NADPH+Trp14** | 11,8579503 |  |

| Application: Tecan i-control  Device: infinite 200Pro | | Tecan i-control , 2.0.10.0  Serial number: 1307001123 | Serial number of connected stacker: |
| --- | --- | --- | --- |
| Firmware: V_3.40_01/15_Infinite (Dec 23 201 MAI, V_3.40_01/15_Infinite (Dec 23 2014/12.45.11) | | | |
| Date: | ######### | | |
| Time: | 15:20:24 | | |

System MTC-MU059-S

User MTC-MU059-S\fretho

Plate Greiner 96 Flat Bottom Transparent Polystyrene Cat. No.: 655101/655161/655192 [GRE96ft.pdfx] Plate-ID (Stacker)

List of actions in this measurement script: Kinetic

Absorbance

Shaking (Linear) Duration: Shaking (Linear) Amplitude:

3 s

1 mm

Label: Label1

Kinetic Measurement

Kinetic duration 00:06:00

Interval Time 00:00:20

Measurement Wavelength 405 nm

Bandwidth 10 nm

Number of Flashes 5

Settle Time 0 ms

Part of Plate E1-E12; F1-F10

Start Time: 2020 08-04 15:20:26

| Cycle Nr. | 1 | 2 | 3 | 4 | 5 | 6 | 7 | 8 | 9 | 10 | 11 | 12 | 13 | 14 | 15 | 16 | 17 | 18 | 19 |
| --- | --- | --- | --- | --- | --- | --- | --- | --- | --- | --- | --- | --- | --- | --- | --- | --- | --- | --- | --- |
| Time [s] | 0 | 20 | 40 | 60 | 80 | 100 | 120 | 140 | 160 | 180 | 200 | 220 | 240 | 260 | 280 | 300 | 320 | 340 | 360 |
| Temp. [°C] | 25,9 | 26,1 | 26,4 | 26,1 | 26 | 26,1 | 26,1 | 26,1 | 26,1 | 26 | 26,1 | 26,2 | 26,1 | 26,1 | 26,2 | 26 | 26,4 | 26,1 | 26,1 |
| E1 | 0,2869 | 0,292 | 0,2994 | 0,3123 | 0,3242 | 0,3338 | 0,3457 | 0,356 | 0,3663 | 0,3758 | 0,3857 | 0,3964 | 0,4056 | 0,4152 | 0,4272 | 0,4357 | 0,4458 | 0,4557 | 0,4658 |
| E2 | 0,2885 | 0,2974 | 0,3034 | 0,3147 | 0,3242 | 0,3351 | 0,3466 | 0,3584 | 0,3674 | 0,3774 | 0,3877 | 0,3991 | 0,4088 | 0,4178 | 0,4276 | 0,4382 | 0,448 | 0,4578 | 0,4677 |
| E3 | 0,2816 | 0,283 | 0,2812 | 0,2887 | 0,2928 | 0,2996 | 0,3053 | 0,3133 | 0,3181 | 0,3204 | 0,3269 | 0,3346 | 0,3387 | 0,345 | 0,3484 | 0,3555 | 0,3617 | 0,3656 | 0,3716 |
| E4 | 0,2747 | 0,2775 | 0,2797 | 0,2874 | 0,2914 | 0,2988 | 0,3067 | 0,3115 | 0,317 | 0,3208 | 0,3256 | 0,3323 | 0,3366 | 0,3399 | 0,346 | 0,3515 | 0,3574 | 0,3604 | 0,3659 |
| E5 | 0,2785 | 0,2831 | 0,2879 | 0,2992 | 0,3069 | 0,3152 | 0,3244 | 0,33 | 0,3367 | 0,3434 | 0,35 | 0,3578 | 0,3638 | 0,3709 | 0,3784 | 0,3847 | 0,3931 | 0,3988 | 0,4056 |
| E6 | 0,2831 | 0,2884 | 0,295 | 0,3058 | 0,315 | 0,3235 | 0,3343 | 0,3412 | 0,35 | 0,3562 | 0,3643 | 0,3727 | 0,382 | 0,388 | 0,3982 | 0,4038 | 0,4145 | 0,4211 | 0,4293 |
| E7 | 0,2766 | 0,2788 | 0,283 | 0,2907 | 0,2956 | 0,3007 | 0,305 | 0,3111 | 0,3149 | 0,3199 | 0,3253 | 0,3319 | 0,3362 | 0,3418 | 0,3482 | 0,3528 | 0,358 | 0,3649 | 0,3676 |
| E8 | 0,2794 | 0,2836 | 0,2878 | 0,2948 | 0,2966 | 0,3022 | 0,3074 | 0,3134 | 0,3168 | 0,3217 | 0,3272 | 0,3347 | 0,3389 | 0,3432 | 0,3491 | 0,353 | 0,3613 | 0,366 | 0,3699 |
| E9 | 0,2804 | 0,2919 | 0,301 | 0,309 | 0,3154 | 0,3235 | 0,3339 | 0,3432 | 0,351 | 0,3596 | 0,3659 | 0,376 | 0,385 | 0,3922 | 0,4006 | 0,4074 | 0,4188 | 0,4269 | 0,4358 |
| E10 | 0,2799 | 0,2914 | 0,2981 | 0,3071 | 0,3139 | 0,3237 | 0,3345 | 0,3426 | 0,3515 | 0,3598 | 0,3671 | 0,3782 | 0,3869 | 0,3937 | 0,4042 | 0,4135 | 0,4223 | 0,4296 | 0,439 |
| E11 | 0,273 | 0,2744 | 0,2739 | 0,2776 | 0,2788 | 0,2826 | 0,287 | 0,2896 | 0,2917 | 0,2944 | 0,2975 | 0,3002 | 0,3038 | 0,3056 | 0,3086 | 0,3119 | 0,3154 | 0,3167 | 0,3194 |
| E12 | 0,2728 | 0,2759 | 0,2775 | 0,2821 | 0,2871 | 0,2925 | 0,298 | 0,2993 | 0,3047 | 0,3074 | 0,3106 | 0,3146 | 0,3176 | 0,3203 | 0,325 | 0,329 | 0,3332 | 0,3371 | 0,3391 |
| F1 | 0,2601 | 0,2577 | 0,2572 | 0,2612 | 0,2616 | 0,2628 | 0,2665 | 0,2681 | 0,2678 | 0,2703 | 0,2726 | 0,2748 | 0,2774 | 0,2773 | 0,2809 | 0,2822 | 0,2846 | 0,2857 | 0,2874 |
| F2 | 0,2601 | 0,2577 | 0,2572 | 0,2612 | 0,2616 | 0,2628 | 0,2665 | 0,2681 | 0,2678 | 0,2703 | 0,2726 | 0,2748 | 0,2774 | 0,2773 | 0,2809 | 0,2822 | 0,2846 | 0,2857 | 0,2874 |
| F3 | 0,2562 | 0,2616 | 0,2642 | 0,2709 | 0,2747 | 0,278 | 0,2808 | 0,2835 | 0,2873 | 0,2893 | 0,2925 | 0,2963 | 0,3017 | 0,3029 | 0,308 | 0,3109 | 0,315 | 0,3176 | 0,3207 |
| F4 | 0,2566 | 0,2618 | 0,2621 | 0,2684 | 0,2716 | 0,2741 | 0,2792 | 0,2818 | 0,2845 | 0,286 | 0,289 | 0,2942 | 0,2969 | 0,2989 | 0,3015 | 0,3056 | 0,3093 | 0,3137 | 0,3141 |
| F5 | 0,2559 | 0,2606 | 0,2613 | 0,264 | 0,2679 | 0,2688 | 0,272 | 0,2742 | 0,2756 | 0,2797 | 0,2812 | 0,2843 | 0,2877 | 0,2881 | 0,2934 | 0,295 | 0,3008 | 0,301 | 0,3025 |
| F6 | 0,2588 | 0,259 | 0,2612 | 0,2646 | 0,2662 | 0,2687 | 0,2709 | 0,2713 | 0,2739 | 0,2768 | 0,2789 | 0,2823 | 0,286 | 0,2866 | 0,2905 | 0,2921 | 0,2955 | 0,2968 | 0,2984 |
| F7 | 0,2627 | 0,2686 | 0,2682 | 0,273 | 0,2752 | 0,28 | 0,2857 | 0,2885 | 0,293 | 0,2949 | 0,299 | 0,3034 | 0,3061 | 0,3104 | 0,316 | 0,3174 | 0,3213 | 0,3226 | 0,3248 |
| F8 | 0,2564 | 0,2632 | 0,2667 | 0,2729 | 0,2766 | 0,2798 | 0,2849 | 0,2873 | 0,2914 | 0,2944 | 0,2983 | 0,3009 | 0,3049 | 0,3057 | 0,3119 | 0,3137 | 0,319 | 0,3228 | 0,325 |
| F9 | 0,2698 | 0,27 | 0,2699 | 0,2707 | 0,2683 | 0,2669 | 0,269 | 0,2695 | 0,2684 | 0,2673 | 0,2683 | 0,2688 | 0,2693 | 0,268 | 0,2686 | 0,2688 | 0,2672 | 0,27 | 0,2669 |
| F10 | 0,2725 | 0,273 | 0,2721 | 0,2736 | 0,2714 | 0,2718 | 0,2709 | 0,2708 | 0,2709 | 0,27 | 0,2699 | 0,2712 | 0,2707 | 0,269 | 0,2695 | 0,271 | 0,2714 | 0,2705 | 0,2686 |

**WTPTPN22 ctr WTPTPN22 ctr WTPTPN22reduced WTPTPN22reduced WTPTPN22+TrxR1+NADPH WTPTPN22+TrxR1+NADPH WTPTPN22+TrxR1+NADPH+Trx WTPTPN22+TrxR1+NADPH+Trx1 WTPTPN22+TrxR1+NADPH+Trp14 WTPTPN22+TrxR1+NADPH+Trp14 mut PTPN22 ctr mut PTPN22 ctr mut PTPN22reduced mut PTPN22reduced mut PTPN22+TrxR1+NADPH mut PTPN22+TrxR1+NADPH mut PTPN22+TrxR1+Nmut PTPN22+TrxR1+Nmut PTPN22+TrxR1+NA mut PTPN22+TrxR1+NADPH+Trp14**

**Time (sec) Blank Blank**

20 0,25819999 0,2653 0,283300012 0,280099988 0,252799988 0,251199991 0,263799995 0,262300014 0,253800005 0,250999987 0,255199999 0,256999999 0,257800013 0,261500001 0,250999987 0,243699998 0,249599993 0,242500007 0,245700002 0,243699998 0,245700002 0,243399993

40 0,257800013 0,2644 0,289200008 0,281699985 0,252200007 0,252400011 0,268299997 0,266400009 0,253100008 0,251700014 0,25819999 0,263500005 0,256599993 0,261400014 0,250499994 0,243799999 0,251700014 0,244399995 0,244000003 0,242699996 0,248199999 0,245100006

60 0,256399989 0,265 0,300700009 0,288899988 0,252799988 0,254599988 0,275700003 0,274399996 0,254299998 0,255600005 0,262400001 0,266000003 0,257800013 0,266499996 0,250400007 0,243599996 0,255600005 0,248199999 0,243599996 0,244900003 0,250699997 0,2509

80 0,256300002 0,2657 0,312099993 0,296999991 0,254299998 0,258300006 0,282499999 0,280900002 0,256599993 0,256900012 0,263200015 0,270399988 0,261000007 0,268700004 0,249899998 0,243300006 0,259600013 0,252999991 0,241999999 0,245299995 0,252600014 0,253300011

100 0,256599993 0,2663 0,322600007 0,308299989 0,255100012 0,262300014 0,289099991 0,289200008 0,259000003 0,258300006 0,26789999 0,27579999 0,263599992 0,273499995 0,251199991 0,245199993 0,261299998 0,255600005 0,2447 0,246299997 0,255499989 0,255600005

120 0,255199999 0,2645 0,332100004 0,315600008 0,256000012 0,262400001 0,295700014 0,293799996 0,261500001 0,259000003 0,271899998 0,279300004 0,266000003 0,275700003 0,25150001 0,243499994 0,265199989 0,256599993 0,244200006 0,244200006 0,257699996 0,255699992

140 0,257299989 0,2641 0,342200011 0,324800014 0,258899987 0,266499996 0,305000007 0,301699996 0,263700008 0,262199998 0,2755 0,284999996 0,26910001 0,279700011 0,252700001 0,242799997 0,26879999 0,261000007 0,245700002 0,247400001 0,260399997 0,258599997

160 0,257099986 0,2648 0,351099998 0,333200008 0,26030001 0,267699987 0,311300009 0,309599996 0,266499996 0,264499992 0,280900002 0,287499994 0,273200005 0,283899993 0,253100008 0,243900001 0,271699995 0,263999999 0,247600004 0,247400001 0,262400001 0,262600005

180 0,256799996 0,2641 0,359499991 0,341399997 0,261200011 0,270399988 0,316900015 0,3134 0,2676 0,266400009 0,283899993 0,29460001 0,275200009 0,285899997 0,253199995 0,245499998 0,274800003 0,267699987 0,247199997 0,248300001 0,263599992 0,264899999

200 0,255800009 0,2651 0,369100004 0,346599996 0,261700004 0,270399988 0,323500007 0,319400012 0,268599987 0,268099993 0,28580001 0,297399998 0,277099997 0,289299995 0,252299994 0,246399999 0,27759999 0,26910001 0,247999996 0,2509 0,264999986 0,264699996

220 0,255499989 0,264 0,377999991 0,354600012 0,263000011 0,272599995 0,328999996 0,325500011 0,270099998 0,271200001 0,291299999 0,301299989 0,278800011 0,290699989 0,251300007 0,247400001 0,281699985 0,273299992 0,248099998 0,246999994 0,26820001 0,271499991

240 0,256399989 0,2652 0,386900008 0,362100005 0,262899995 0,273999989 0,333799988 0,332500011 0,272700012 0,273799986 0,294699997 0,306100011 0,281599998 0,29460001 0,252400011 0,244599998 0,28580001 0,2764 0,248699993 0,250499994 0,268700004 0,268099993

260 0,255800009 0,2636 0,395700008 0,368400007 0,265399992 0,276199996 0,34009999 0,33860001 0,273999989 0,275200009 0,297699988 0,310499996 0,281100005 0,296799988 0,252200007 0,244800001 0,28580001 0,274699986 0,249500006 0,250099987 0,270900011 0,26879999

280 0,256799996 0,2643 0,404399991 0,375699997 0,264899999 0,278299987 0,344799995 0,343400002 0,278899997 0,279799998 0,300999999 0,316399992 0,284999996 0,299600005 0,250999987 0,246099994 0,290100008 0,279900014 0,250099987 0,249899998 0,273000002 0,269400001

300 0,252799988 0,2623 0,413500011 0,381500006 0,266799986 0,278200001 0,351900011 0,3477 0,278699994 0,280800015 0,301800013 0,320300013 0,2852 0,300700009 0,25029999 0,243599996 0,291500002 0,282000005 0,249699995 0,249500006 0,272799999 0,273000002

320 0,255699992 0,2641 0,421499997 0,390500009 0,270000011 0,283899993 0,358099997 0,3565 0,281899989 0,283499986 0,309300005 0,324200004 0,289200008 0,305099994 0,251899987 0,245100006 0,297399998 0,286500007 0,253500015 0,252900004 0,27669999 0,2755

340 0,256000012 0,2643 0,429699987 0,398299992 0,269400001 0,282499999 0,364899993 0,363099992 0,283699989 0,285699993 0,312999994 0,330900013 0,291299999 0,307300001 0,254000008 0,246700004 0,298900008 0,290100008 0,252700001 0,254500002 0,280299991 0,277399987

360 0,256099999 0,2671 0,440499991 0,405400008 0,270500004 0,2861 0,371899992 0,367900014 0,287600011 0,289400011 0,317299992 0,336199999 0,296600014 0,3116 0,255400002 0,246800005 0,303299993 0,292199999 0,251899987 0,256999999 0,283100009 0,280200005

slope -5,91331E-06 -2,1E-06 0,000462379 0,00037985 5,68937E-05 9,9969E-05 0,000316672 0,000312534 0,000101078 0,000112234 0,00018161 0,000226852 0,000115273 0,000148344 7,55935E-06 8,08051E-06 0,000156677 0,000144427 2,84984E-05 3,3225E-05 0,000102565 9,99226E-05

slope w/o blank

0,000466373 0,000383844 6,08875E-05 0,000103963 0,000320666 0,000316527 0,000105072 0,000116228 0,000185604 0,000230846 0,000119267 0,000152337 1,15532E-05 1,20743E-05 0,000160671 0,000148421 3,24923E-05 3,72188E-05 0,000106558 0,000103916

0,000425108 8,24252E-05 0,000318596 0,00011065 0,000208225 0,000135802 1,18137E-05 0,000154546 3,48555E-05 0,000105237

0,025506502 0,004945511 0,019115789 0,006639009 0,012493499 0,008148142 0,000708824 0,009272756 0,002091331 0,006314241

3,806940592 0,738135929 2,853102845 0,990896792 1,86470138 1,216140626 0,105794678 1,383993445 0,312138984 0,94242406

27,1924328 5,272399496 20,37930604 7,077834228 13,31929557 8,68671876 0,755676269 9,885667467 2,229564173 6,73160043

**min -1** 27,1924328 5,272399496 20,37930604 7,077834228 13,31929557 8,68671876 0,755676269 9,885667467 2,229564173 6,73160043

**5 min** 30

**WTPTPN22 ctr** 27,19243 76,05790021

**WTPTPN22reduced** 5,272399 4,469147798

**WTPTPN22+TrxR1+NADPH** 20,37931 97,26724967

**WTPTPN22+TrxR1+NADPH+Trx1** 7,077834 31,22145889

**WTPTPN22+TrxR1+NADPH+Trp14** 13,3193

**mut PTPN22 ctr** 8,686719

**mut PTPN22reduced** 0,755676

**mut PTPN22+TrxR1+NADPH** 9,885667

**mut PTPN22+TrxR1+NADPH+Trx1** 2,229564

**mut PTPN22+TrxR1+NADPH+Trp14** 6,7316

| Application: Tecan i-control  Device: infinite 200Pro | | Tecan i-control , 2.0.10.0  Serial number: 1307001123 | Serial number of connected stacker: |
| --- | --- | --- | --- |
| Firmware: V_3.40_01/15_Infinite (Dec 23 201 MAI, V_3.40_01/15_Infinite (Dec 23 2014/12.45.11) | | | |
| Date: | ######### | | |
| Time: | 15:30:42 | | |

System MTC-MU059-S

User MTC-MU059-S\fretho

Plate Greiner 96 Flat Bottom Transparent Polystyrene Cat. No.: 655101/655161/655192 [GRE96ft.pdfx] Plate-ID (Stacker)

List of actions in this measurement script: Kinetic

Absorbance

Shaking (Linear) Duration: Shaking (Linear) Amplitude:

3 s

1 mm

Label: Label1

Kinetic Measurement

Kinetic duration 00:06:00

Interval Time 00:00:20

Measurement Wavelength 405 nm

Bandwidth 10 nm

Number of Flashes 5

Settle Time 0 ms

Part of Plate E1-E12; F1-F10

Start Time: 2020 08-04 15:30:44

| Cycle Nr. | 1 | 2 | 3 | 4 | 5 | 6 | 7 | 8 | 9 | 10 | 11 | 12 | 13 | 14 | 15 | 16 | 17 | 18 | 19 |
| --- | --- | --- | --- | --- | --- | --- | --- | --- | --- | --- | --- | --- | --- | --- | --- | --- | --- | --- | --- |
| Time [s] | 0 | 20 | 40 | 60 | 80 | 100 | 120 | 140 | 160 | 180 | 200 | 220 | 240 | 260 | 280 | 300 | 320 | 340 | 360 |
| Temp. [°C] | 26,1 | 26 | 26,1 | 26,1 | 26 | 26,3 | 26,1 | 26,1 | 26 | 26,4 | 26,4 | 26,1 | 26,5 | 26,5 | 26,5 | 26,1 | 26,1 | 26,3 | 26,5 |
| E1 | 0,2761 | 0,2833 | 0,2892 | 0,3007 | 0,3121 | 0,3226 | 0,3321 | 0,3422 | 0,3511 | 0,3595 | 0,3691 | 0,378 | 0,3869 | 0,3957 | 0,4044 | 0,4135 | 0,4215 | 0,4297 | 0,4405 |
| E2 | 0,2719 | 0,2801 | 0,2817 | 0,2889 | 0,297 | 0,3083 | 0,3156 | 0,3248 | 0,3332 | 0,3414 | 0,3466 | 0,3546 | 0,3621 | 0,3684 | 0,3757 | 0,3815 | 0,3905 | 0,3983 | 0,4054 |
| E3 | 0,2534 | 0,2528 | 0,2522 | 0,2528 | 0,2543 | 0,2551 | 0,256 | 0,2589 | 0,2603 | 0,2612 | 0,2617 | 0,263 | 0,2629 | 0,2654 | 0,2649 | 0,2668 | 0,27 | 0,2694 | 0,2705 |
| E4 | 0,2519 | 0,2512 | 0,2524 | 0,2546 | 0,2583 | 0,2623 | 0,2624 | 0,2665 | 0,2677 | 0,2704 | 0,2704 | 0,2726 | 0,274 | 0,2762 | 0,2783 | 0,2782 | 0,2839 | 0,2825 | 0,2861 |
| E5 | 0,2592 | 0,2638 | 0,2683 | 0,2757 | 0,2825 | 0,2891 | 0,2957 | 0,305 | 0,3113 | 0,3169 | 0,3235 | 0,329 | 0,3338 | 0,3401 | 0,3448 | 0,3519 | 0,3581 | 0,3649 | 0,3719 |
| E6 | 0,2579 | 0,2623 | 0,2664 | 0,2744 | 0,2809 | 0,2892 | 0,2938 | 0,3017 | 0,3096 | 0,3134 | 0,3194 | 0,3255 | 0,3325 | 0,3386 | 0,3434 | 0,3477 | 0,3565 | 0,3631 | 0,3679 |
| E7 | 0,2504 | 0,2538 | 0,2531 | 0,2543 | 0,2566 | 0,259 | 0,2615 | 0,2637 | 0,2665 | 0,2676 | 0,2686 | 0,2701 | 0,2727 | 0,274 | 0,2789 | 0,2787 | 0,2819 | 0,2837 | 0,2876 |
| E8 | 0,2479 | 0,251 | 0,2517 | 0,2556 | 0,2569 | 0,2583 | 0,259 | 0,2622 | 0,2645 | 0,2664 | 0,2681 | 0,2712 | 0,2738 | 0,2752 | 0,2798 | 0,2808 | 0,2835 | 0,2857 | 0,2894 |
| E9 | 0,2506 | 0,2552 | 0,2582 | 0,2624 | 0,2632 | 0,2679 | 0,2719 | 0,2755 | 0,2809 | 0,2839 | 0,2858 | 0,2913 | 0,2947 | 0,2977 | 0,301 | 0,3018 | 0,3093 | 0,313 | 0,3173 |
| E10 | 0,2535 | 0,257 | 0,2635 | 0,266 | 0,2704 | 0,2758 | 0,2793 | 0,285 | 0,2875 | 0,2946 | 0,2974 | 0,3013 | 0,3061 | 0,3105 | 0,3164 | 0,3203 | 0,3242 | 0,3309 | 0,3362 |
| E11 | 0,2556 | 0,2578 | 0,2566 | 0,2578 | 0,261 | 0,2636 | 0,266 | 0,2691 | 0,2732 | 0,2752 | 0,2771 | 0,2788 | 0,2816 | 0,2811 | 0,285 | 0,2852 | 0,2892 | 0,2913 | 0,2966 |
| E12 | 0,2614 | 0,2615 | 0,2614 | 0,2665 | 0,2687 | 0,2735 | 0,2757 | 0,2797 | 0,2839 | 0,2859 | 0,2893 | 0,2907 | 0,2946 | 0,2968 | 0,2996 | 0,3007 | 0,3051 | 0,3073 | 0,3116 |
| F1 | 0,2557 | 0,251 | 0,2505 | 0,2504 | 0,2499 | 0,2512 | 0,2515 | 0,2527 | 0,2531 | 0,2532 | 0,2523 | 0,2513 | 0,2524 | 0,2522 | 0,251 | 0,2503 | 0,2519 | 0,254 | 0,2554 |
| F2 | 0,2464 | 0,2437 | 0,2438 | 0,2436 | 0,2433 | 0,2452 | 0,2435 | 0,2428 | 0,2439 | 0,2455 | 0,2464 | 0,2474 | 0,2446 | 0,2448 | 0,2461 | 0,2436 | 0,2451 | 0,2467 | 0,2468 |
| F3 | 0,2438 | 0,2496 | 0,2517 | 0,2556 | 0,2596 | 0,2613 | 0,2652 | 0,2688 | 0,2717 | 0,2748 | 0,2776 | 0,2817 | 0,2858 | 0,2858 | 0,2901 | 0,2915 | 0,2974 | 0,2989 | 0,3033 |
| F4 | 0,2378 | 0,2425 | 0,2444 | 0,2482 | 0,253 | 0,2556 | 0,2566 | 0,261 | 0,264 | 0,2677 | 0,2691 | 0,2733 | 0,2764 | 0,2747 | 0,2799 | 0,282 | 0,2865 | 0,2901 | 0,2922 |
| F5 | 0,2442 | 0,2457 | 0,244 | 0,2436 | 0,242 | 0,2447 | 0,2442 | 0,2457 | 0,2476 | 0,2472 | 0,248 | 0,2481 | 0,2487 | 0,2495 | 0,2501 | 0,2497 | 0,2535 | 0,2527 | 0,2519 |
| F6 | 0,2431 | 0,2437 | 0,2427 | 0,2449 | 0,2453 | 0,2463 | 0,2442 | 0,2474 | 0,2474 | 0,2483 | 0,2509 | 0,247 | 0,2505 | 0,2501 | 0,2499 | 0,2495 | 0,2529 | 0,2545 | 0,257 |
| F7 | 0,2416 | 0,2457 | 0,2482 | 0,2507 | 0,2526 | 0,2555 | 0,2577 | 0,2604 | 0,2624 | 0,2636 | 0,265 | 0,2682 | 0,2687 | 0,2709 | 0,273 | 0,2728 | 0,2767 | 0,2803 | 0,2831 |
| F8 | 0,2472 | 0,2434 | 0,2451 | 0,2509 | 0,2533 | 0,2556 | 0,2557 | 0,2586 | 0,2626 | 0,2649 | 0,2647 | 0,2715 | 0,2681 | 0,2688 | 0,2694 | 0,273 | 0,2755 | 0,2774 | 0,2802 |
| F9 | 0,2581 | 0,2582 | 0,2578 | 0,2564 | 0,2563 | 0,2566 | 0,2552 | 0,2573 | 0,2571 | 0,2568 | 0,2558 | 0,2555 | 0,2564 | 0,2558 | 0,2568 | 0,2528 | 0,2557 | 0,256 | 0,2561 |
| F10 | 0,266 | 0,2653 | 0,2644 | 0,265 | 0,2657 | 0,2663 | 0,2645 | 0,2641 | 0,2648 | 0,2641 | 0,2651 | 0,264 | 0,2652 | 0,2636 | 0,2643 | 0,2623 | 0,2641 | 0,2643 | 0,2671 |

**WTPTPN22 ctr WTPTPN22 ctr WTPTPN22reduced WTPTPN22reduced WTPTPN22+TrxR1+NADPH WTPTPN22+TrxR1+NADPH WTPTPN22+TrxR1+NADPH+Tr WTPTPN22+TrxR1+NADPH+ WTPTPN22+TrxR1+NADPH+Trp14 WTPTPN22+TrxR1+NADPH+Trp14 mut PTPN22 ctr mut PTPN22 ctr mut PTPN22reduced mut PTPN22reduced mut PTPN22+TrxR1+NADPH mut PTPN22+TrxR1+NADPH mut PTPN22+TrxR1+Nmut PTPN22+TrxR1+Nmut PTPN22+TrxR1+NA mut PTPN22+TrxR1+NADPH+Trp14**

**Time (sec) Blank Blank**

20 0,245199993 0,246000007 0,255199999 0,264400005 0,231099993 0,228799999 0,241099998 0,242500007 0,233700007 0,240600005 0,244100004 0,238900006 0,249699995 0,253300011 0,229599997 0,228599995 0,233099997 0,231399998 0,228100002 0,231099993 0,235699996 0,239899993

40 0,242200002 0,244200006 0,258899987 0,260500014 0,226699993 0,229900002 0,244499996 0,245800003 0,230100006 0,240400001 0,244599998 0,241099998 0,25029999 0,253600001 0,224700004 0,224800006 0,230800003 0,228699997 0,225899994 0,226600006 0,234599993 0,238999993

60 0,244200006 0,242599994 0,265700012 0,267800003 0,228 0,229000002 0,250400007 0,251100004 0,237200007 0,2412 0,25029999 0,243300006 0,255899996 0,259900004 0,226600006 0,228300005 0,234400004 0,230000004 0,227799997 0,228499994 0,235200003 0,238299996

80 0,246900007 0,2456 0,271299988 0,27669999 0,232099995 0,230900005 0,254400015 0,257099986 0,2377 0,241799995 0,251599997 0,247799993 0,261700004 0,264200002 0,225999996 0,226600006 0,237599999 0,230000004 0,228499994 0,230199993 0,240099996 0,240099996

100 0,243399993 0,243799999 0,277999997 0,284000009 0,231600001 0,231299996 0,258599997 0,261000007 0,238499999 0,238999993 0,253600001 0,248500004 0,266900003 0,270000011 0,226400003 0,227699995 0,242300004 0,231399998 0,2289 0,230599999 0,238499999 0,240799993

120 0,2456 0,246000007 0,289299995 0,292499989 0,232999995 0,234300002 0,263399988 0,26699999 0,239899993 0,241600007 0,256199986 0,2509 0,271800011 0,273900002 0,226699993 0,2271 0,244399995 0,234099999 0,2315 0,230000004 0,239800006 0,245100006

140 0,244100004 0,246000007 0,294 0,298500001 0,234799996 0,233400002 0,26789999 0,269899994 0,2403 0,242400005 0,255800009 0,253899992 0,273799986 0,279300004 0,230000004 0,228699997 0,245499998 0,233500004 0,230800003 0,230499998 0,245299995 0,248600006

160 0,244200006 0,246600002 0,301899999 0,306400001 0,236499995 0,234099999 0,271200001 0,274100006 0,241600007 0,243399993 0,261200011 0,254000008 0,279900014 0,282799989 0,228100002 0,229100004 0,249300003 0,2359 0,229800001 0,230299994 0,243300006 0,247700006

180 0,246199995 0,247299999 0,307399988 0,314799994 0,236000001 0,234999999 0,275299996 0,279300004 0,242599994 0,244000003 0,264299989 0,25909999 0,28459999 0,288300008 0,229599997 0,229800001 0,248799995 0,235200003 0,231199995 0,231600001 0,246199995 0,25029999

200 0,243599996 0,2447 0,313300014 0,319799989 0,235599995 0,234699994 0,279300004 0,281699985 0,242500007 0,243200004 0,264099985 0,258300006 0,286300004 0,290800005 0,2271 0,2271 0,25150001 0,235799998 0,228699997 0,230900005 0,244599998 0,2491

220 0,245700002 0,244599998 0,32190001 0,325800002 0,2359 0,2359 0,284099996 0,287699997 0,244100004 0,246199995 0,26879999 0,260600001 0,291599989 0,295300007 0,229200006 0,229599997 0,252200007 0,238399997 0,229800001 0,231000006 0,246600002 0,253100008

240 0,246900007 0,246999994 0,329100013 0,335799992 0,238399997 0,237599999 0,288399994 0,291599989 0,245000005 0,247400001 0,273000002 0,264899999 0,296499997 0,300999999 0,230199993 0,228499994 0,254900008 0,238600001 0,230599999 0,232299998 0,248799995 0,255299985

260 0,243200004 0,245499998 0,335599989 0,340700001 0,238700002 0,237900004 0,292299986 0,294999987 0,245199993 0,247899994 0,273699999 0,266099989 0,298900008 0,301800013 0,228799999 0,228599995 0,256599993 0,239800006 0,230599999 0,231299996 0,247899994 0,256399989

280 0,244900003 0,245900005 0,342099994 0,346799999 0,238299996 0,237499997 0,295399994 0,297100008 0,246999994 0,248400003 0,276199996 0,268400013 0,30340001 0,307900012 0,228799999 0,226999998 0,2579 0,240999997 0,231299996 0,232299998 0,249500006 0,257499993

300 0,244900003 0,245900005 0,349000007 0,353799999 0,2403 0,239099994 0,300500005 0,301800013 0,248400003 0,248699993 0,278600007 0,271600008 0,307599992 0,310200006 0,228599995 0,226600006 0,258300006 0,241300002 0,2315 0,231399998 0,251300007 0,256099999

320 0,245700002 0,246099994 0,355699986 0,361900002 0,239700004 0,239299998 0,3046 0,306499988 0,250400007 0,251399994 0,282900006 0,273900002 0,310600013 0,315100014 0,228699997 0,227799997 0,262199998 0,240799993 0,231099993 0,232299998 0,252400011 0,256799996

340 0,247799993 0,247400001 0,362199992 0,369899988 0,240899995 0,239700004 0,308200002 0,309799999 0,251800001 0,252999991 0,286799997 0,276600003 0,315699995 0,319900006 0,230700001 0,227300003 0,263799995 0,2447 0,233600006 0,232999995 0,253300011 0,2597

360 0,244900003 0,247400001 0,369500011 0,374900013 0,240700006 0,240799993 0,311399996 0,311399996 0,252000004 0,25029999 0,287200004 0,27759999 0,3204 0,321799994 0,227699995 0,226999998 0,264699996 0,243399993 0,230900005 0,232800007 0,254900008 0,25909999

slope 4,59237E-06 6,83177E-06 0,000343638 0,000347946 3,78999E-05 3,52012E-05 0,000208514 0,000207214 5,52322E-05 3,73426E-05 0,000130083 0,000114009 0,000209876 0,000209881 7,82764E-06 2,9927E-07 9,8323E-05 4,48194E-05 1,29412E-05 1,14706E-05 5,85759E-05 6,86945E-05

slope w/o blank

0,000337926 0,000342234 3,21878E-05 2,94892E-05 0,000202802 0,000201502 4,95201E-05 3,16305E-05 0,000124371 0,000108297 0,000204164 0,000204169 2,11557E-06 -5,4128E-06 9,26109E-05 3,91073E-05 7,22912E-06 5,75853E-06 5,28638E-05 6,29825E-05

0,00034008 3,08385E-05 0,000202152 4,05753E-05 0,000116334 0,000204167 -1,64861E-06 6,58591E-05 6,49382E-06 5,79231E-05

0,020404799 0,00185031 0,012129102 0,002434519 0,006980032 0,012250001 -9,89167E-05 0,003951548 0,000389629 0,003475388

3,045492388 0,276165683 1,810313698 0,363361104 1,041795753 1,828358322 -0,014763693 0,589783247 0,058153625 0,518714647

21,75351705 1,972612024 12,93081213 2,59543646 7,441398235 13,0597023 -0,105454952 4,212737477 0,415383037 3,705104624

**min -1** 21,75351705 1,972612024 12,93081213 2,59543646 7,441398235 13,0597023 -0,105454952 4,212737477 0,415383037 3,705104624

**5 min** 30

**WTPTPN22 ctr** 21,75351705 76,05790021

**WTPTPN22reduced** 1,972612024 4,469147798

**WTPTPN22+TrxR1+NADPH** 12,93081213 97,26724967

**WTPTPN22+TrxR1+NADPH+Trx1** 2,59543646 31,22145889

**WTPTPN22+TrxR1+NADPH+Trp14** 7,441398235

**mut PTPN22 ctr** 13,0597023

**mut PTPN22reduced** -0,10545495

**mut PTPN22+TrxR1+NADPH** 4,212737477

**mut PTPN22+TrxR1+NADPH+Trx1** 0,415383037

**mut PTPN22+TrxR1+NADPH+Trp14** 3,705104624

| Application: Tecan i-control  Device: infinite 200Pro | | Tecan i-control , 2.0.10.0  Serial number: 1307001123 | Serial number of connected stacker: |
| --- | --- | --- | --- |
| Firmware: V_3.40_01/15_Infinite (Dec 23 201 MAI, V_3.40_01/15_Infinite (Dec 23 2014/12.45.11) | | | |
| Date: | ######### | | |
| Time: | 15:45:01 | | |

System MTC-MU059-S

User MTC-MU059-S\fretho

Plate Greiner 96 Flat Bottom Transparent Polystyrene Cat. No.: 655101/655161/655192 [GRE96ft.pdfx] Plate-ID (Stacker)

List of actions in this measurement script: Kinetic

Absorbance

Shaking (Linear) Duration: Shaking (Linear) Amplitude:

3 s

1 mm

Label: Label1

Kinetic Measurement

Kinetic duration 00:06:00

Interval Time 00:00:20

Measurement Wavelength 405 nm

Bandwidth 10 nm

Number of Flashes 5

Settle Time 0 ms

Part of Plate E1-E12; F1-F10

Start Time: 2020 08-04 15:45:04

| Cycle Nr. | 1 | 2 | 3 | 4 | 5 | 6 | 7 | 8 | 9 | 10 | 11 | 12 | 13 | 14 | 15 | 16 | 17 | 18 | 19 |
| --- | --- | --- | --- | --- | --- | --- | --- | --- | --- | --- | --- | --- | --- | --- | --- | --- | --- | --- | --- |
| Time [s] | 0 | 20 | 40 | 60 | 80 | 100 | 120 | 140 | 160 | 180 | 200 | 220 | 240 | 260 | 280 | 300 | 320 | 340 | 360 |
| Temp. [°C] | 26,6 | 26,3 | 26,5 | 26,1 | 26,4 | 26,3 | 26,4 | 26,7 | 26,3 | 26,2 | 26,6 | 26,3 | 26,4 | 26,3 | 26,4 | 26,6 | 26,6 | 26,4 | 26,4 |
| E1 | 0,251 | 0,2552 | 0,2589 | 0,2657 | 0,2713 | 0,278 | 0,2893 | 0,294 | 0,3019 | 0,3074 | 0,3133 | 0,3219 | 0,3291 | 0,3356 | 0,3421 | 0,349 | 0,3557 | 0,3622 | 0,3695 |
| E2 | 0,2537 | 0,2644 | 0,2605 | 0,2678 | 0,2767 | 0,284 | 0,2925 | 0,2985 | 0,3064 | 0,3148 | 0,3198 | 0,3258 | 0,3358 | 0,3407 | 0,3468 | 0,3538 | 0,3619 | 0,3699 | 0,3749 |
| E3 | 0,2329 | 0,2311 | 0,2267 | 0,228 | 0,2321 | 0,2316 | 0,233 | 0,2348 | 0,2365 | 0,236 | 0,2356 | 0,2359 | 0,2384 | 0,2387 | 0,2383 | 0,2403 | 0,2397 | 0,2409 | 0,2407 |
| E4 | 0,2315 | 0,2288 | 0,2299 | 0,229 | 0,2309 | 0,2313 | 0,2343 | 0,2334 | 0,2341 | 0,235 | 0,2347 | 0,2359 | 0,2376 | 0,2379 | 0,2375 | 0,2391 | 0,2393 | 0,2397 | 0,2408 |
| E5 | 0,24 | 0,2411 | 0,2445 | 0,2504 | 0,2544 | 0,2586 | 0,2634 | 0,2679 | 0,2712 | 0,2753 | 0,2793 | 0,2841 | 0,2884 | 0,2923 | 0,2954 | 0,3005 | 0,3046 | 0,3082 | 0,3114 |
| E6 | 0,2426 | 0,2425 | 0,2458 | 0,2511 | 0,2571 | 0,261 | 0,267 | 0,2699 | 0,2741 | 0,2793 | 0,2817 | 0,2877 | 0,2916 | 0,295 | 0,2971 | 0,3018 | 0,3065 | 0,3098 | 0,3114 |
| E7 | 0,2335 | 0,2337 | 0,2301 | 0,2372 | 0,2377 | 0,2385 | 0,2399 | 0,2403 | 0,2416 | 0,2426 | 0,2425 | 0,2441 | 0,245 | 0,2452 | 0,247 | 0,2484 | 0,2504 | 0,2518 | 0,252 |
| E8 | 0,2391 | 0,2406 | 0,2404 | 0,2412 | 0,2418 | 0,239 | 0,2416 | 0,2424 | 0,2434 | 0,244 | 0,2432 | 0,2462 | 0,2474 | 0,2479 | 0,2484 | 0,2487 | 0,2514 | 0,253 | 0,2503 |
| E9 | 0,2407 | 0,2441 | 0,2446 | 0,2503 | 0,2516 | 0,2536 | 0,2562 | 0,2558 | 0,2612 | 0,2643 | 0,2641 | 0,2688 | 0,273 | 0,2737 | 0,2762 | 0,2786 | 0,2829 | 0,2868 | 0,2872 |
| E10 | 0,2376 | 0,2389 | 0,2411 | 0,2433 | 0,2478 | 0,2485 | 0,2509 | 0,2539 | 0,254 | 0,2591 | 0,2583 | 0,2606 | 0,2649 | 0,2661 | 0,2684 | 0,2716 | 0,2739 | 0,2766 | 0,2776 |
| E11 | 0,2472 | 0,2497 | 0,2503 | 0,2559 | 0,2617 | 0,2669 | 0,2718 | 0,2738 | 0,2799 | 0,2846 | 0,2863 | 0,2916 | 0,2965 | 0,2989 | 0,3034 | 0,3076 | 0,3106 | 0,3157 | 0,3204 |
| E12 | 0,2489 | 0,2533 | 0,2536 | 0,2599 | 0,2642 | 0,27 | 0,2739 | 0,2793 | 0,2828 | 0,2883 | 0,2908 | 0,2953 | 0,301 | 0,3018 | 0,3079 | 0,3102 | 0,3151 | 0,3199 | 0,3218 |
| F1 | 0,2338 | 0,2296 | 0,2247 | 0,2266 | 0,226 | 0,2264 | 0,2267 | 0,23 | 0,2281 | 0,2296 | 0,2271 | 0,2292 | 0,2302 | 0,2288 | 0,2288 | 0,2286 | 0,2287 | 0,2307 | 0,2277 |
| F2 | 0,2313 | 0,2286 | 0,2248 | 0,2283 | 0,2266 | 0,2277 | 0,2271 | 0,2287 | 0,2291 | 0,2298 | 0,2271 | 0,2296 | 0,2285 | 0,2286 | 0,227 | 0,2266 | 0,2278 | 0,2273 | 0,227 |
| F3 | 0,2342 | 0,2331 | 0,2308 | 0,2344 | 0,2376 | 0,2423 | 0,2444 | 0,2455 | 0,2493 | 0,2488 | 0,2515 | 0,2522 | 0,2549 | 0,2566 | 0,2579 | 0,2583 | 0,2622 | 0,2638 | 0,2647 |
| F4 | 0,233 | 0,2314 | 0,2287 | 0,23 | 0,23 | 0,2314 | 0,2341 | 0,2335 | 0,2359 | 0,2352 | 0,2358 | 0,2384 | 0,2386 | 0,2398 | 0,241 | 0,2413 | 0,2408 | 0,2447 | 0,2434 |
| F5 | 0,2295 | 0,2281 | 0,2259 | 0,2278 | 0,2285 | 0,2289 | 0,2315 | 0,2308 | 0,2298 | 0,2312 | 0,2287 | 0,2298 | 0,2306 | 0,2306 | 0,2313 | 0,2315 | 0,2311 | 0,2336 | 0,2309 |
| F6 | 0,2321 | 0,2311 | 0,2266 | 0,2285 | 0,2302 | 0,2306 | 0,23 | 0,2305 | 0,2303 | 0,2316 | 0,2309 | 0,231 | 0,2323 | 0,2313 | 0,2323 | 0,2314 | 0,2323 | 0,233 | 0,2328 |
| F7 | 0,2347 | 0,2357 | 0,2346 | 0,2352 | 0,2401 | 0,2385 | 0,2398 | 0,2453 | 0,2433 | 0,2462 | 0,2446 | 0,2466 | 0,2488 | 0,2479 | 0,2495 | 0,2513 | 0,2524 | 0,2533 | 0,2549 |
| F8 | 0,2389 | 0,2399 | 0,239 | 0,2383 | 0,2401 | 0,2408 | 0,2451 | 0,2486 | 0,2477 | 0,2503 | 0,2491 | 0,2531 | 0,2553 | 0,2564 | 0,2575 | 0,2561 | 0,2568 | 0,2597 | 0,2591 |
| F9 | 0,2463 | 0,2452 | 0,2422 | 0,2442 | 0,2469 | 0,2434 | 0,2456 | 0,2441 | 0,2442 | 0,2462 | 0,2436 | 0,2457 | 0,2469 | 0,2432 | 0,2449 | 0,2449 | 0,2457 | 0,2478 | 0,2449 |
| F10 | 0,2463 | 0,246 | 0,2442 | 0,2426 | 0,2456 | 0,2438 | 0,246 | 0,246 | 0,2466 | 0,2473 | 0,2447 | 0,2446 | 0,247 | 0,2455 | 0,2459 | 0,2459 | 0,2461 | 0,2474 | 0,2474 |

WTPTPN22 ctr WTPTPN22reduced WTPTPN22+TrxR1+NADPH WTPTPN22+TrxR1+NADPH+Trx1 WTPTPN22+TrxR1+NADPH+Trp14 mut PTPN22 ctr mut PTPN22reduced mut PTPN22+TrxR1+NADPH mut PTPN22+TrxR1+NADPH+Trx1 mut PTPN22+TrxR1+NADPH+Trp14

| 27 | 27 | 27 | 27 | 27 | 27 | 27 | 27 | 27 | 27 | 27 | 27 | 27 | 27 | 27 | 12 | 12 | 12 | 12 | 12 | 12 | 12 | 12 | 12 12 | 12 | 12 | 12 | 12 | 12 |
| --- | --- | --- | --- | --- | --- | --- | --- | --- | --- | --- | --- | --- | --- | --- | --- | --- | --- | --- | --- | --- | --- | --- | --- | --- | --- | --- | --- | --- |
| 25,88571 | 27 | 27,4 9,428571 | | 17,6 | 17,5 24,25714 | | 25,1 | 27,9 19,07143 | | 17,1 | 18,4 22,07143 | | 28 | 27,8 12,94286 | | 11 | 10,6 | 3,9 | 6,3 | 4,4 15,06416 10,94351 14,72304 11,4188 8,347252 5,326362 8,787056 11,85795 | | | | | | | | 11,80448 |
| 25,24286 | 27,2 | 28,9 4,285714 | | 5,3 | 6,6 19,58571 | | 20,4 | 19 9,514286 | | 7,1 | 8,2 14,82857 | | 13,3 | 13,8 11,87143 | | 8,7 | 10,1 | 0,257143 | 0,8 | 1 10,7293 9,885667 11,40081 3,535617 2,229564 2,878464 6,047217 6,7316 | | | | | | | | 7,272076884 |

24,412 21,75352 24,98482 3,464984 1,972612 2,123614 11,58103 12,93081 12,54959 4,01404 2,595436 2,050176 6,45963 7,441398 6,949441 12,58062 13,0597 9,539102 -0,08571 -0,1 -0,7 7,696699 4,212737 7,154079 1,383452 0,415383 -0,26504 7,353766 3,705105 2,66838083
