# Supplementary figures and images for: Redox regulation of PTPN22 affects the severity of T-cell-dependent autoimmune inflammation
Source: eLife. 2022 May 19;11:e74549. doi: 10.7554/eLife.74549 (PMC9119677; doi:10.7554/eLife.74549)

## Slide 1
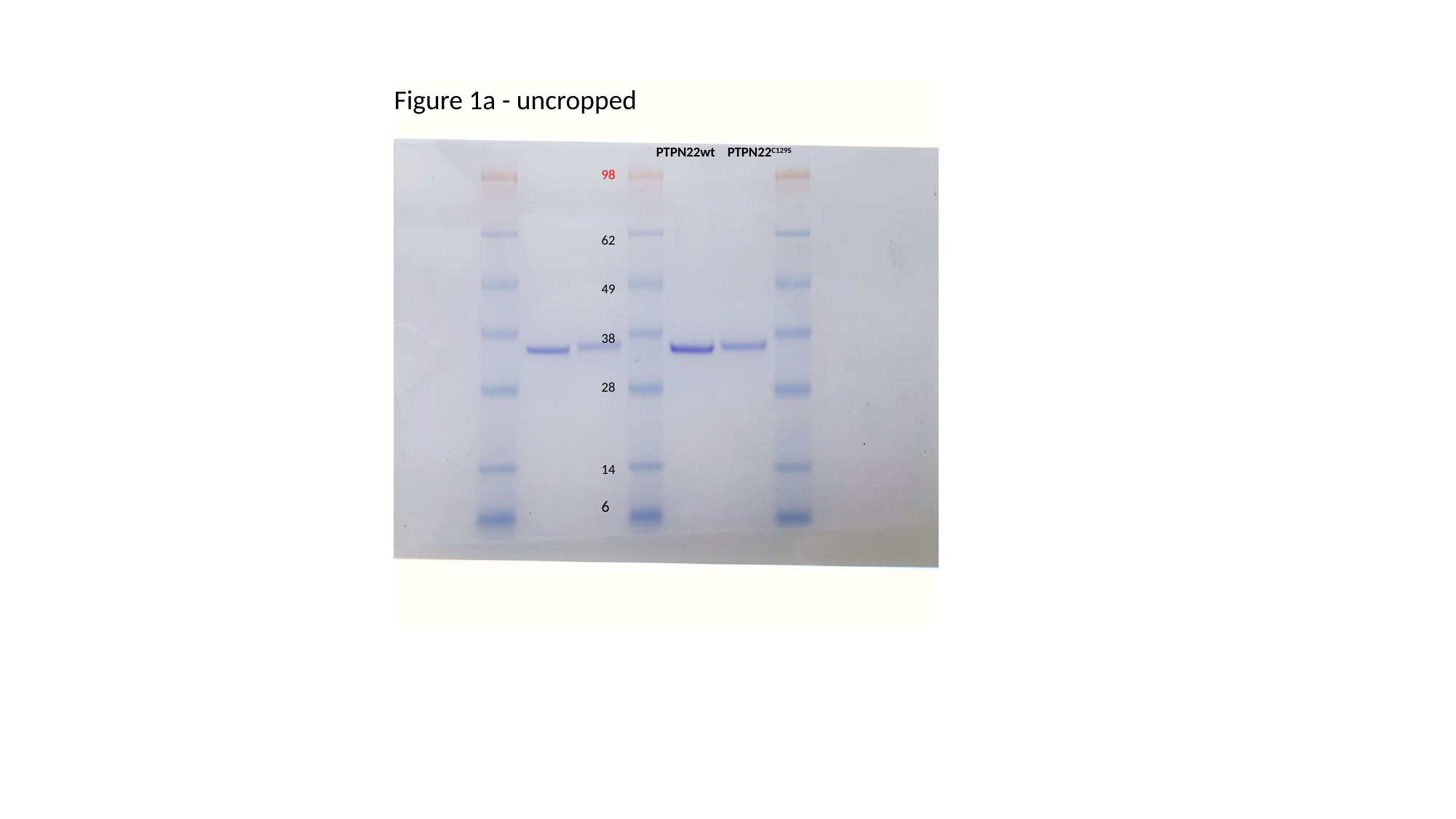

PTPN22wt PTPN22C129S
98
62
49
38
28
14
6
Figure 1a - uncropped

Supplement: Figure 1—source data 6. [file elife-74549-fig1-data6.pptx]

## Slide 1
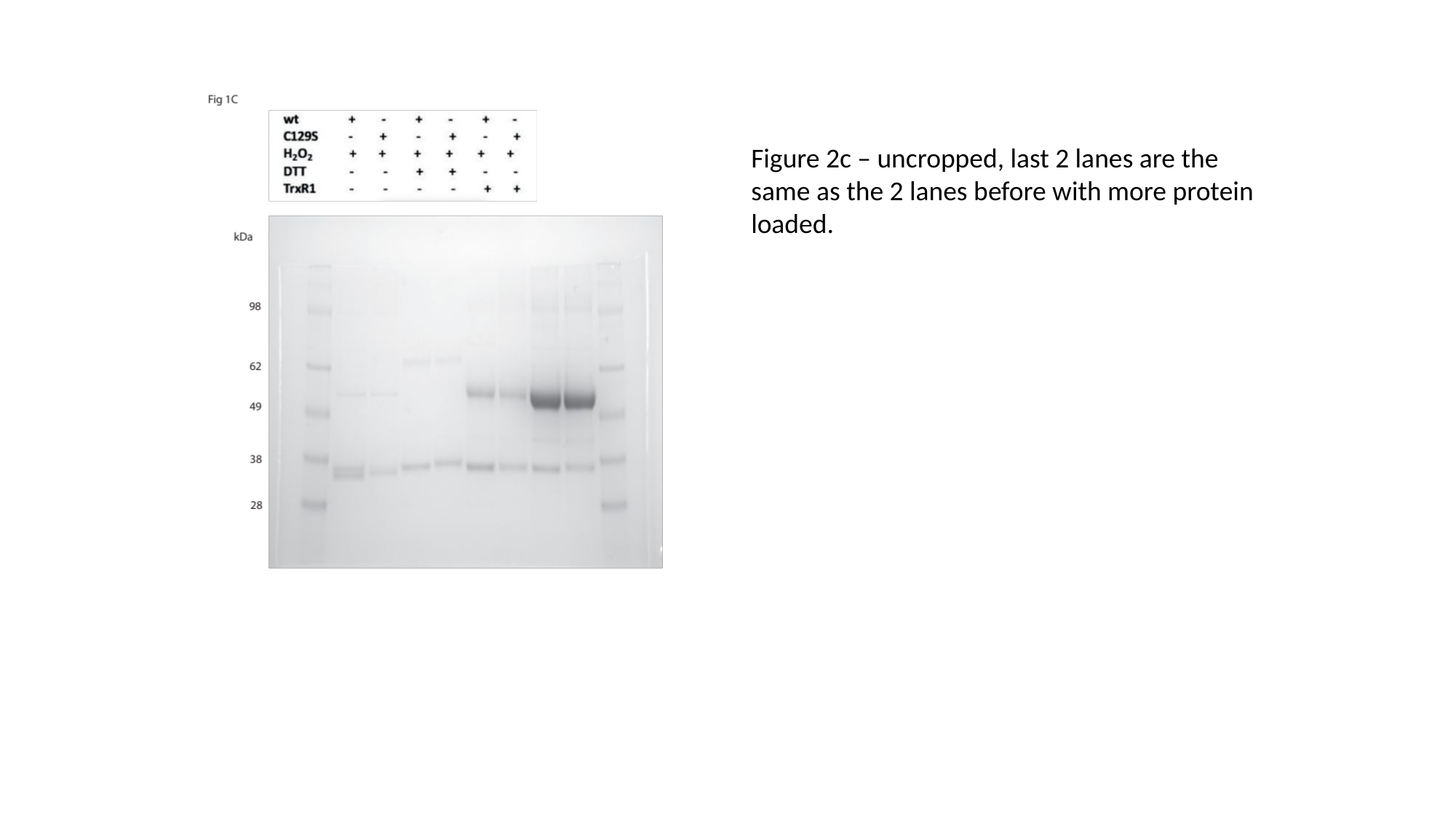

Figure 2c – uncropped, last 2 lanes are the same as the 2 lanes before with more protein loaded.

Supplement: Figure 2—source data 3. [file elife-74549-fig2-data3.pptx]

## Slide 1
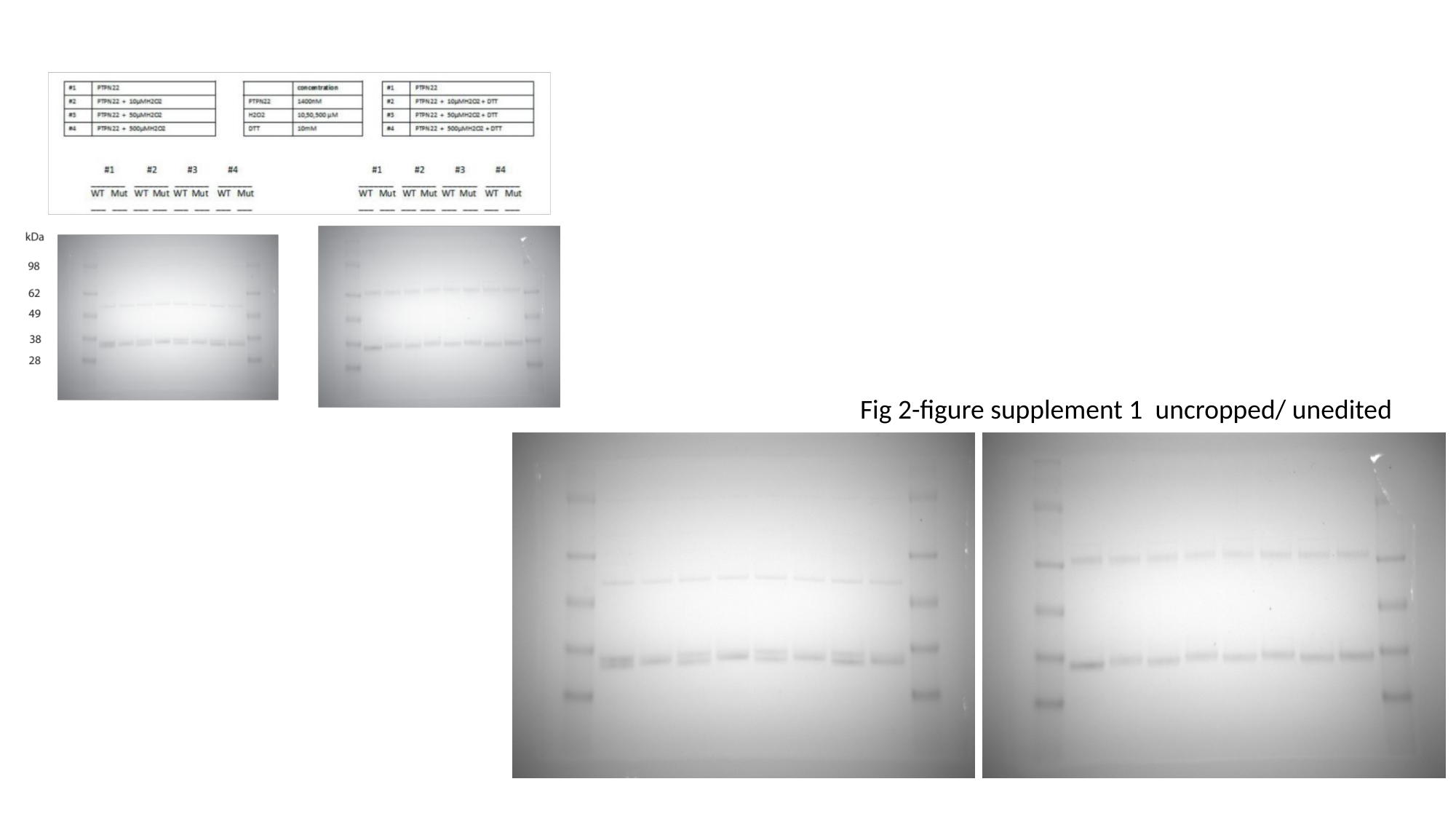

Fig 2-figure supplement 1 uncropped/ unedited

Supplement: Figure 2—figure supplement 1—source data 1. [file elife-74549-fig2-figsupp1-data1.pptx]

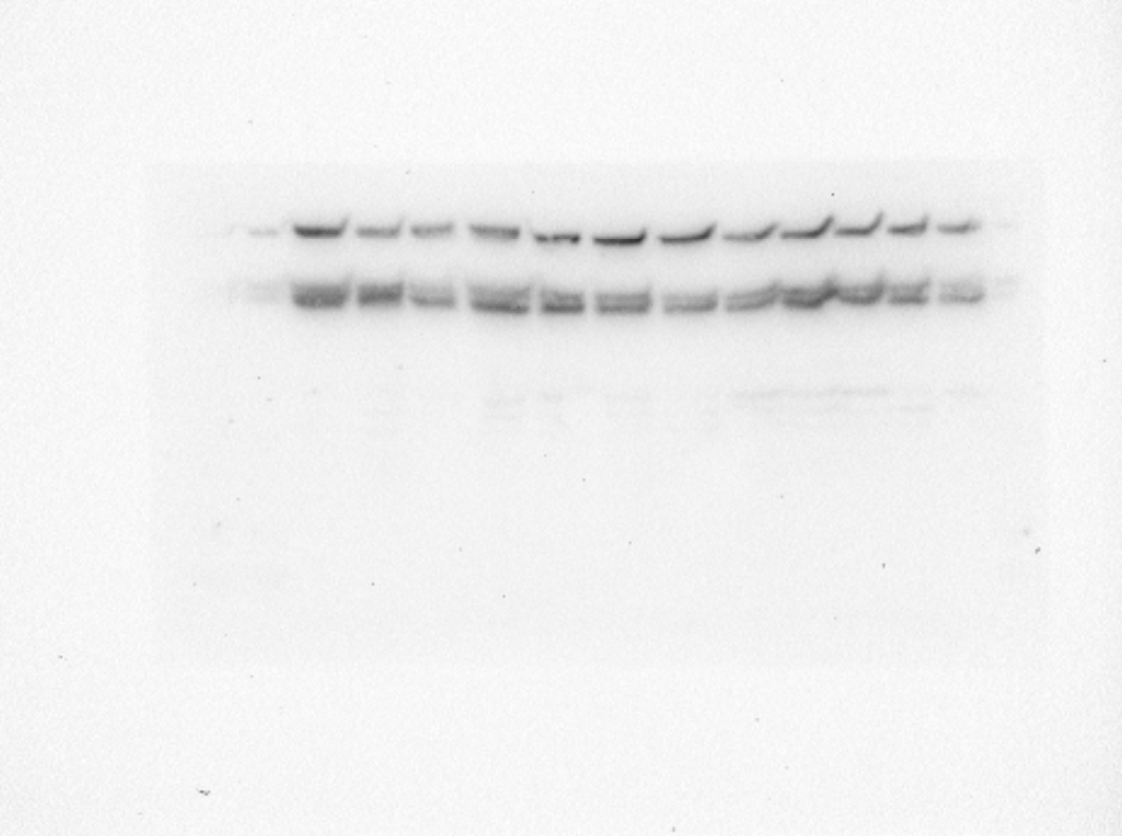

Supplement: Figure 6—source data 2. [file elife-74549-fig6-data2.zip › Source 6a-b, ERK, Zap70, FYN, LCK/Inflam 2020-04-20_11h24m58s_Exposure_269.5sec_to quantify_3mins.tif]

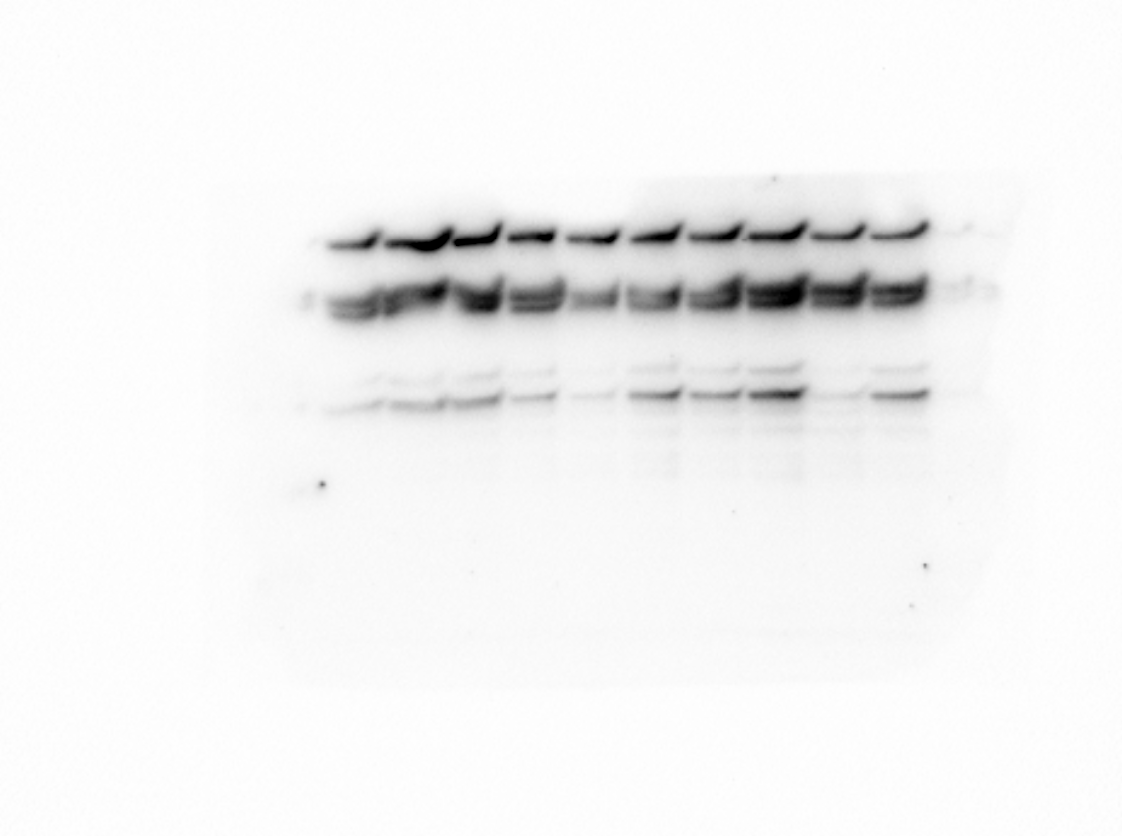

Supplement: Figure 6—source data 2. [file elife-74549-fig6-data2.zip › Source 6a-b, ERK, Zap70, FYN, LCK/Inflam 2020-04-20_11h49m42s_Exposure_15.2sec_pE.tif]

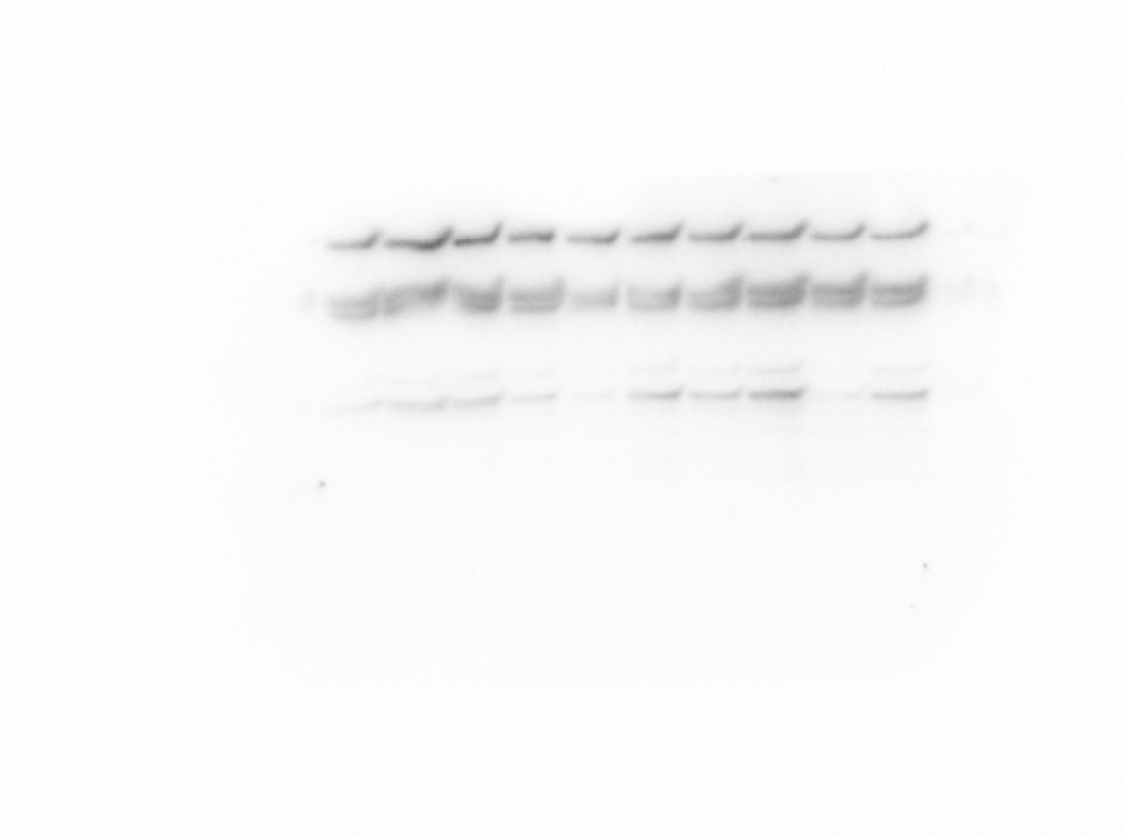

Supplement: Figure 6—source data 3. [file elife-74549-fig6-data3.zip › Source 6a-b, pZap70 Y493, pSrc/Fyn, LCK 6mins to quantify.tif]

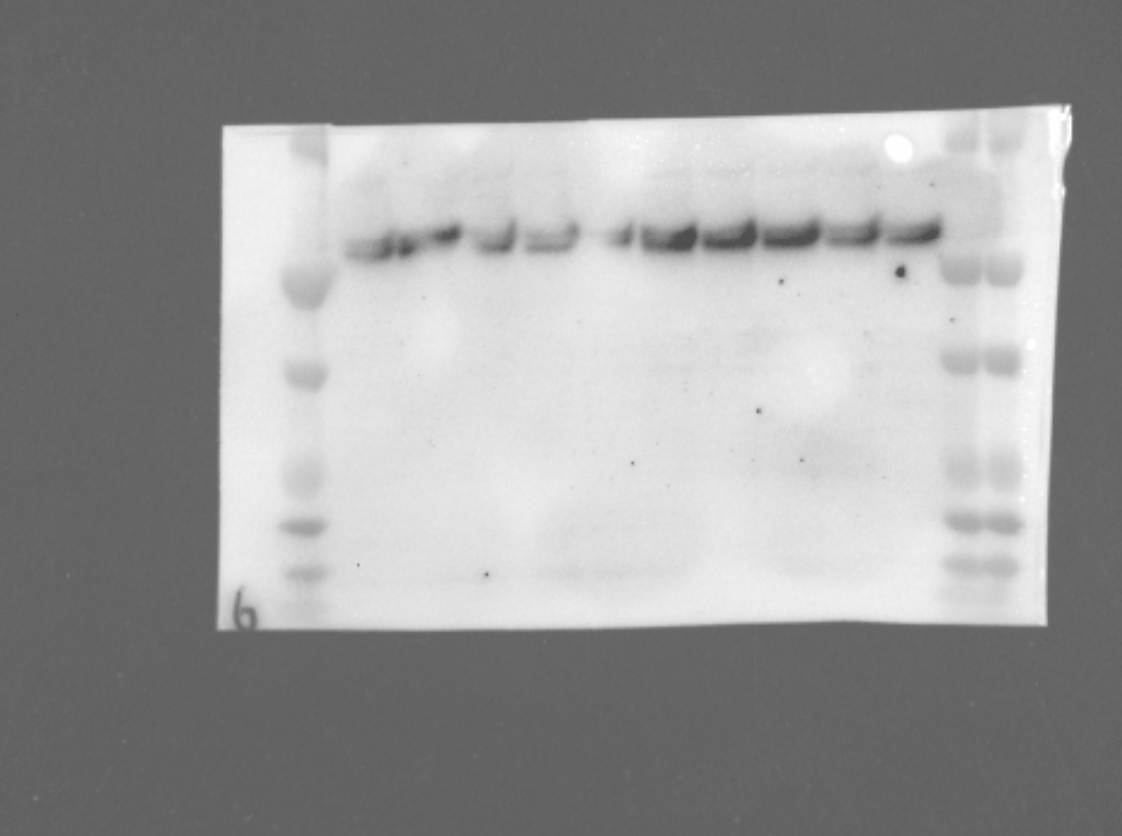

Supplement: Figure 6—source data 3. [file elife-74549-fig6-data3.zip › Source 6a-b, pZap70 Y493, pSrc/Inflam 2020-04-18_11h05m04s_Exposure_15.2sec_pSrc6'_to quantify.tif]

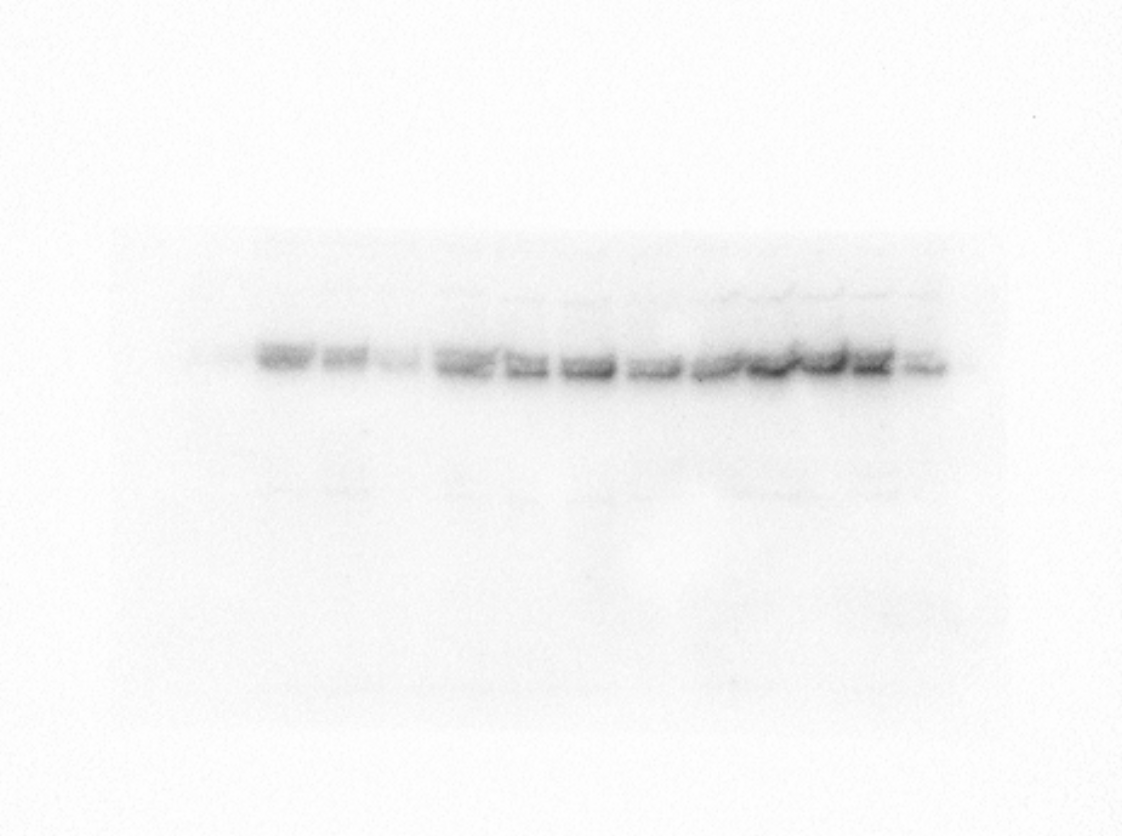

Supplement: Figure 6—source data 3. [file elife-74549-fig6-data3.zip › Source 6a-b, pZap70 Y493, pSrc/Inflam 2020-04-18_11h30m51s_Exposure_5.0sec_for quantification.tif]

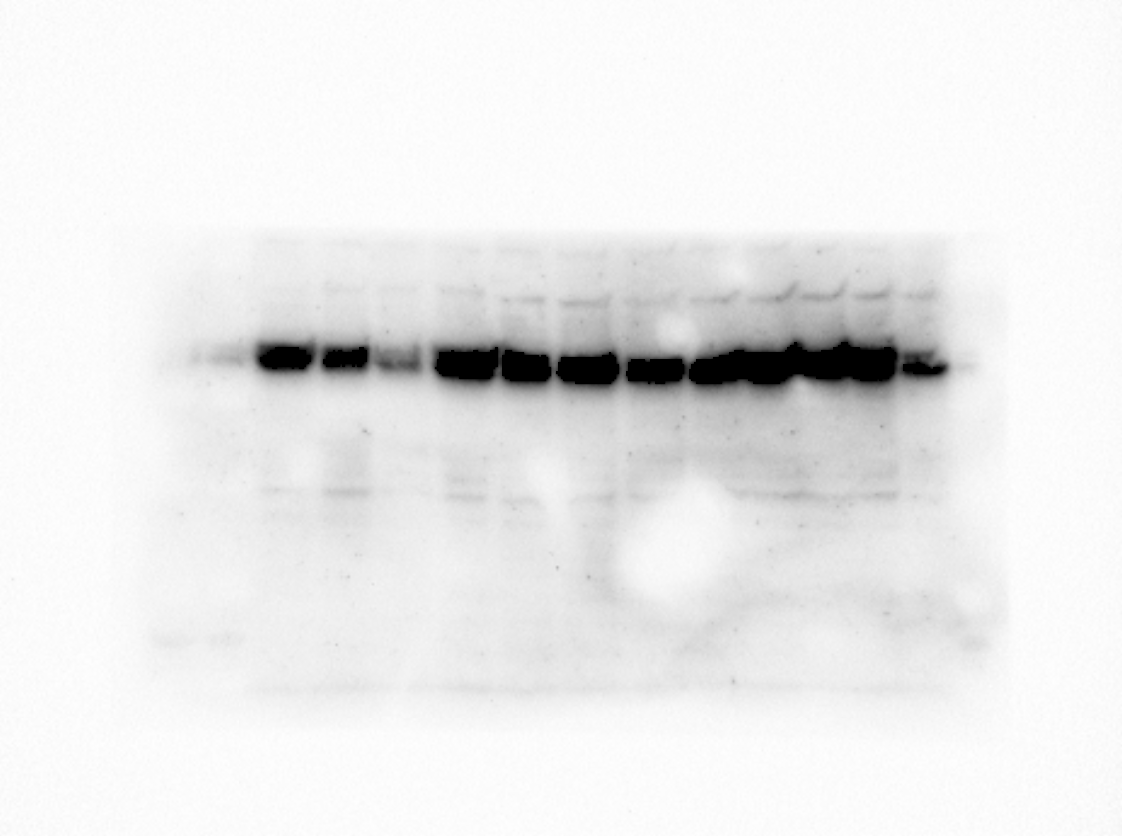

Supplement: Figure 6—source data 3. [file elife-74549-fig6-data3.zip › Source 6a-b, pZap70 Y493, pSrc/Inflam 2020-04-18_11h30m51s_Exposure_76.2sec_pZap70_for quantification.tif]

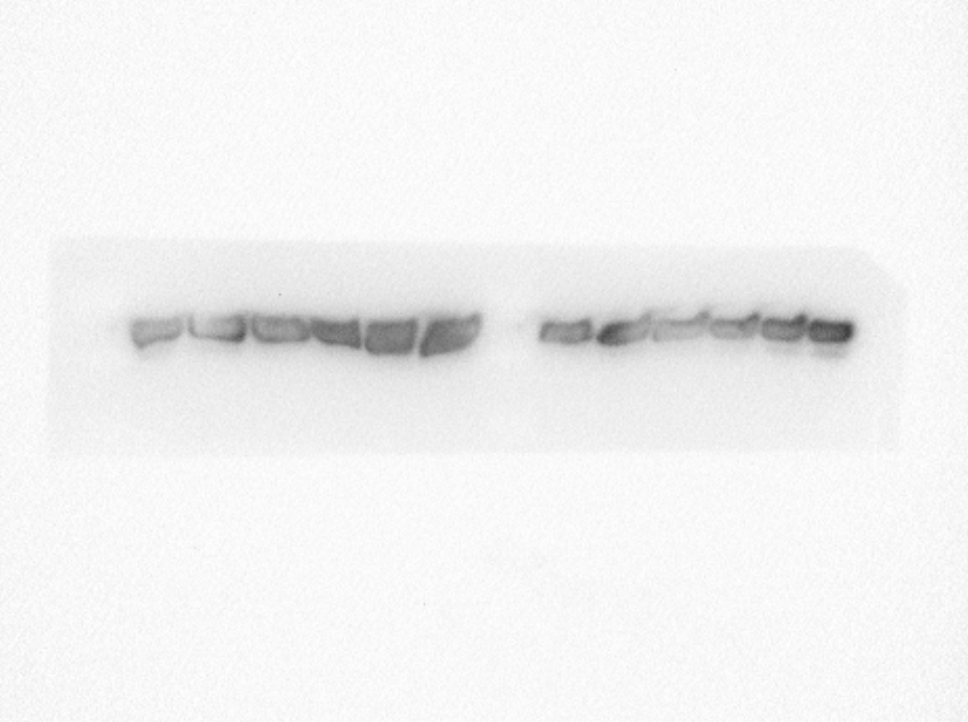

Supplement: Figure 6—source data 4. [file elife-74549-fig6-data4.zip › Soure 6c/gel 1/actin figure.tif]

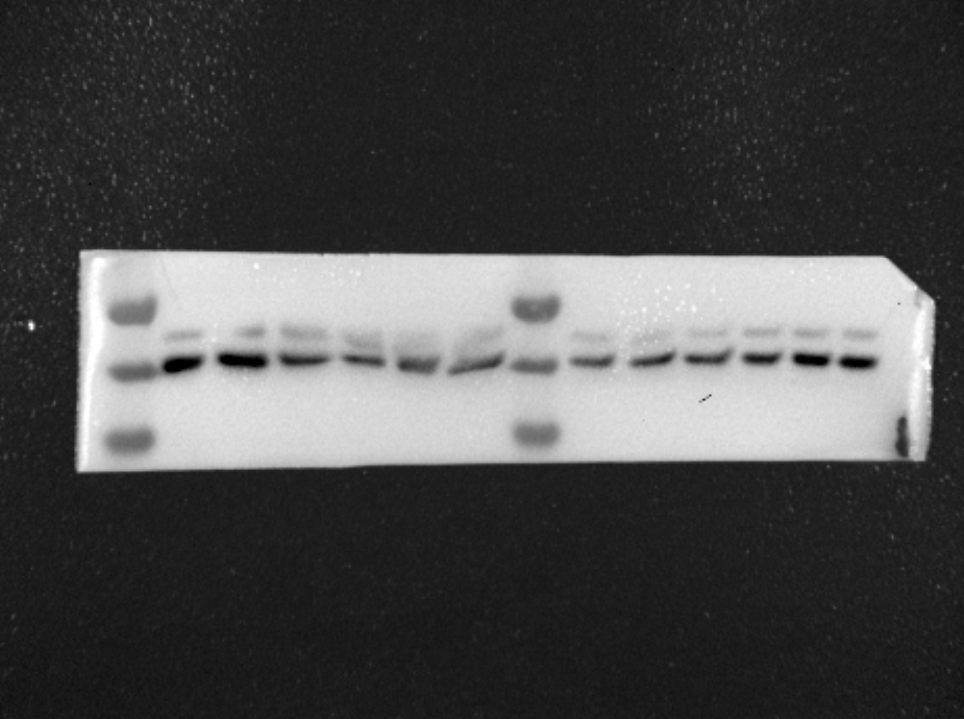

Supplement: Figure 6—source data 4. [file elife-74549-fig6-data4.zip › Soure 6c/gel 1/ERK figure.tif]

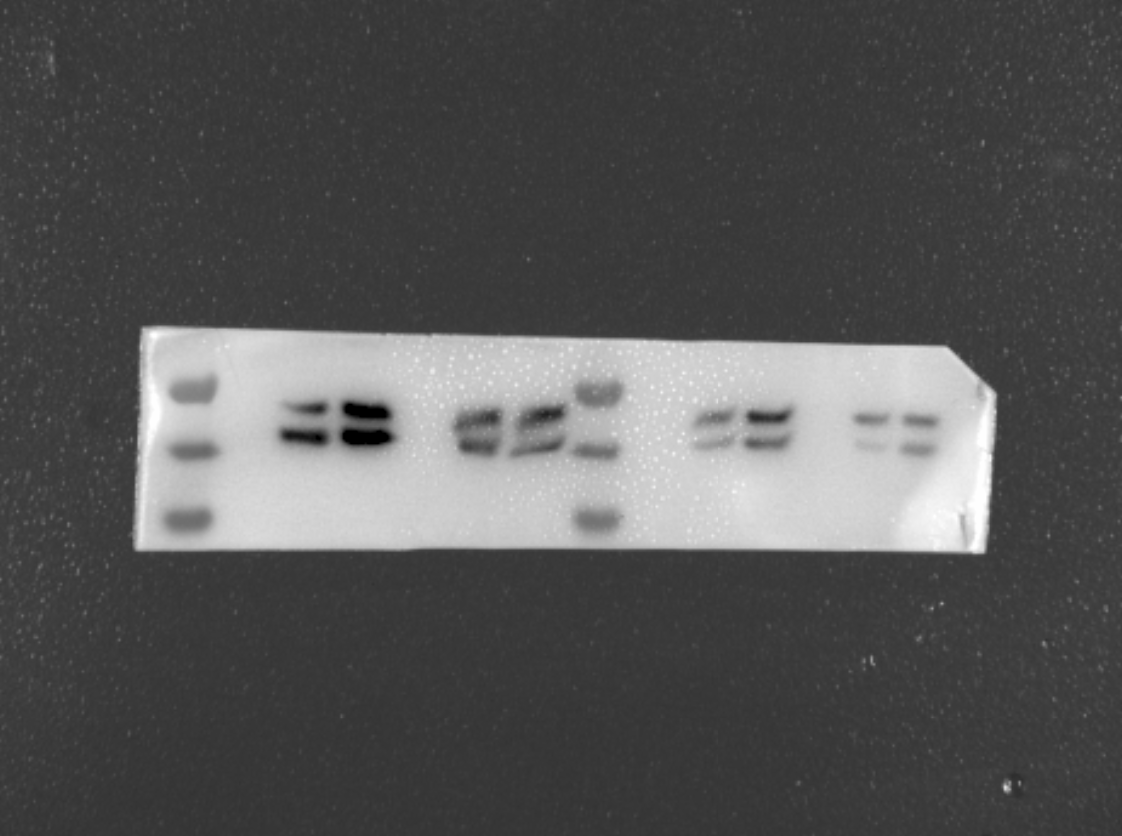

Supplement: Figure 6—source data 4. [file elife-74549-fig6-data4.zip › Soure 6c/gel 1/pERK figure.tif]

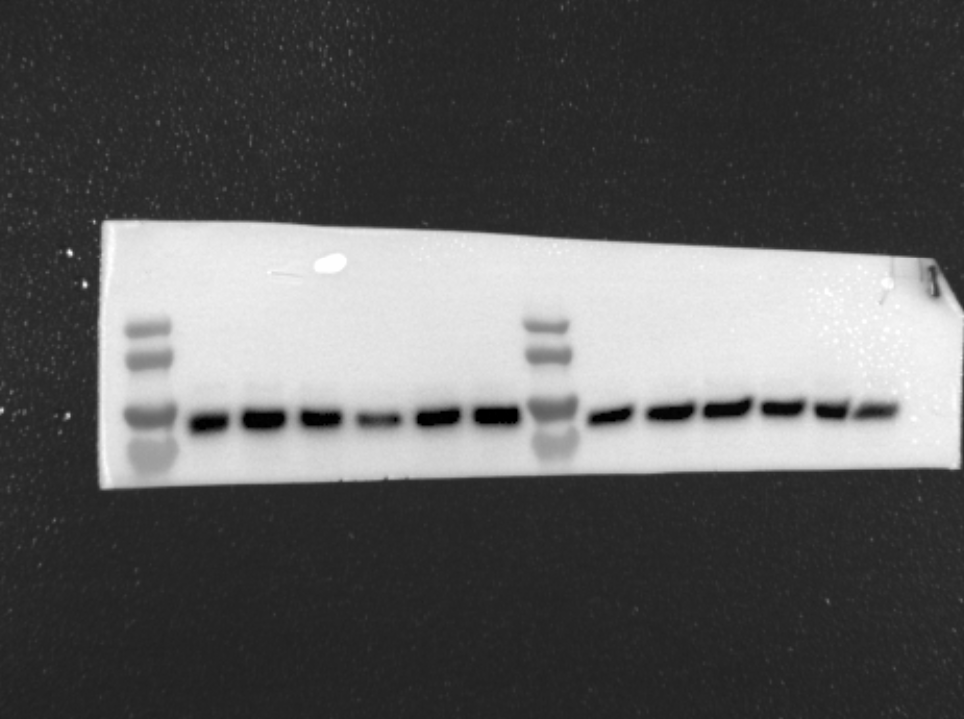

Supplement: Figure 6—source data 4. [file elife-74549-fig6-data4.zip › Soure 6c/gel 1/pkc figure.tif]

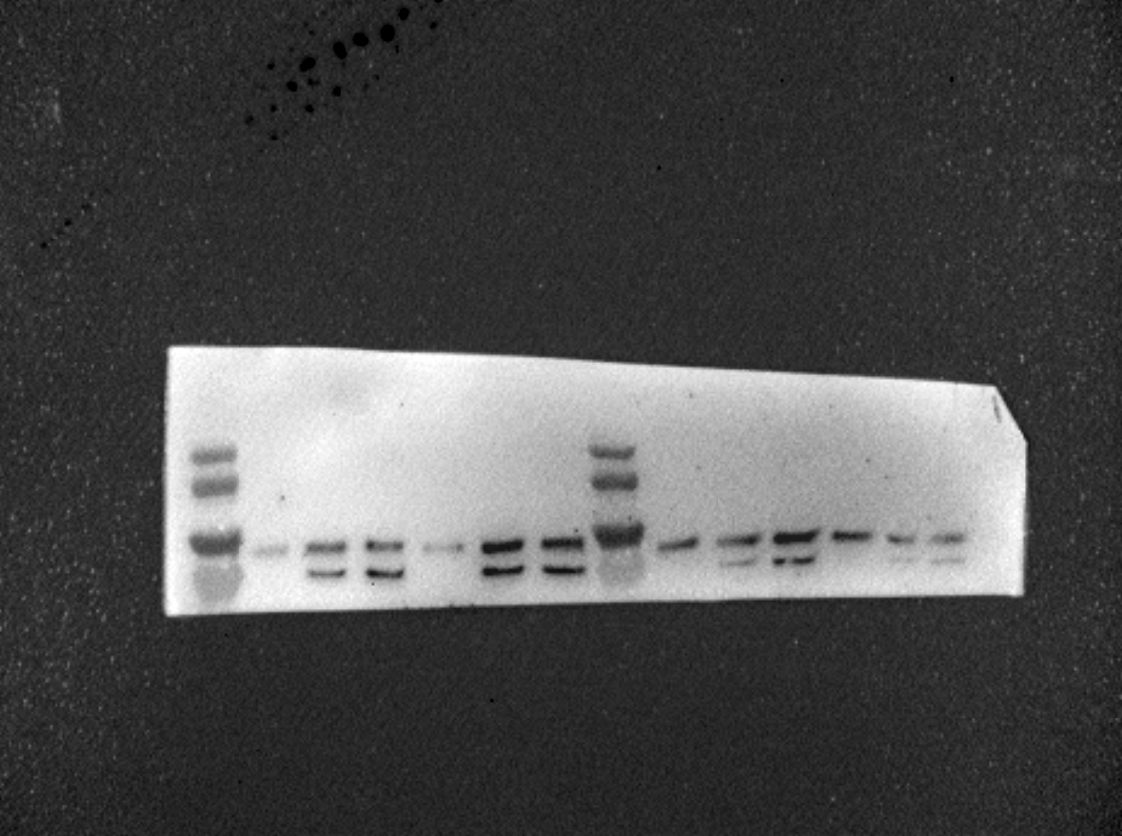

Supplement: Figure 6—source data 4. [file elife-74549-fig6-data4.zip › Soure 6c/gel 1/ppkc figure.tif]

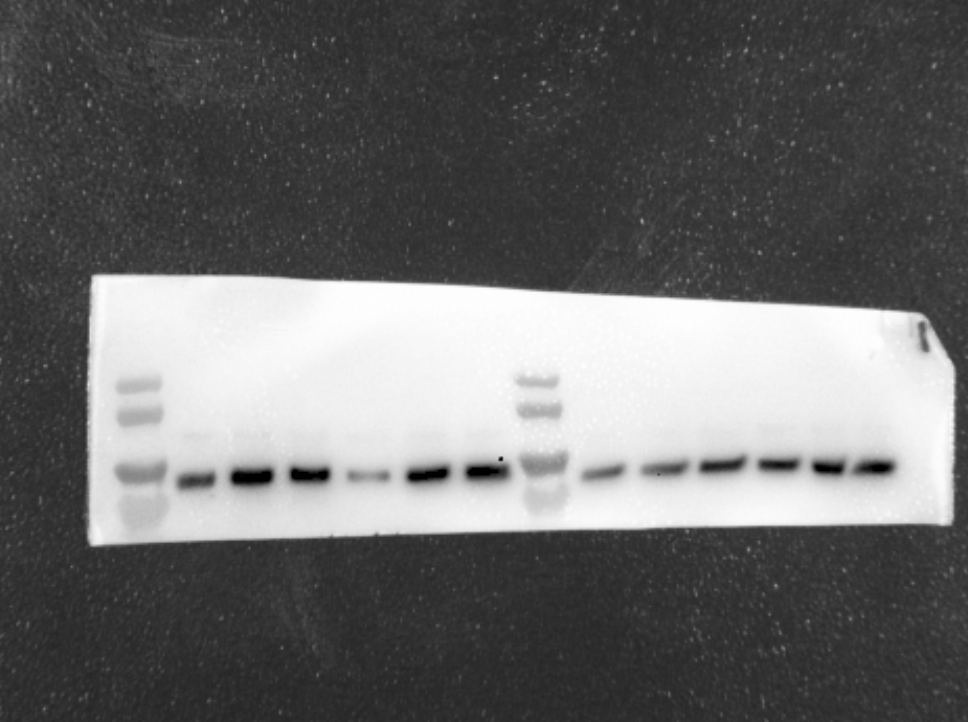

Supplement: Figure 6—source data 4. [file elife-74549-fig6-data4.zip › Soure 6c/gel 1/pzap70.tif]

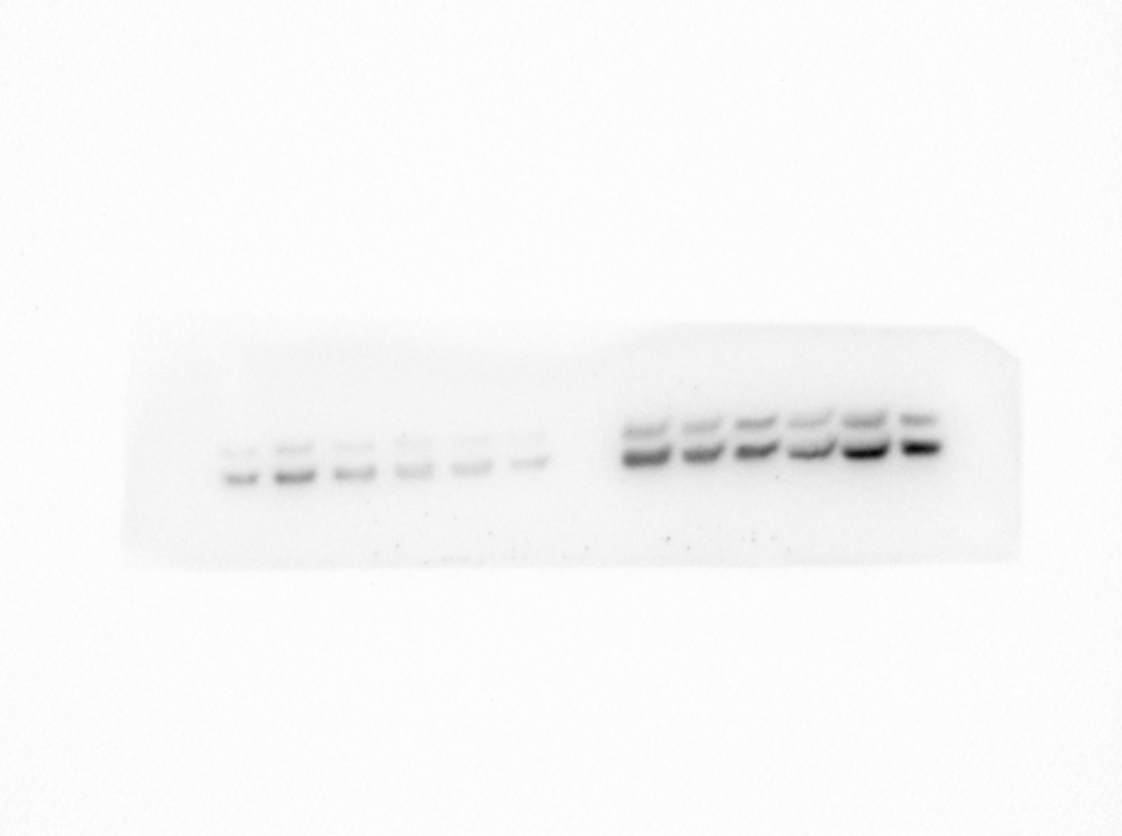

Supplement: Figure 6—source data 4. [file elife-74549-fig6-data4.zip › Soure 6c/gel 2/ERK figure.tif]

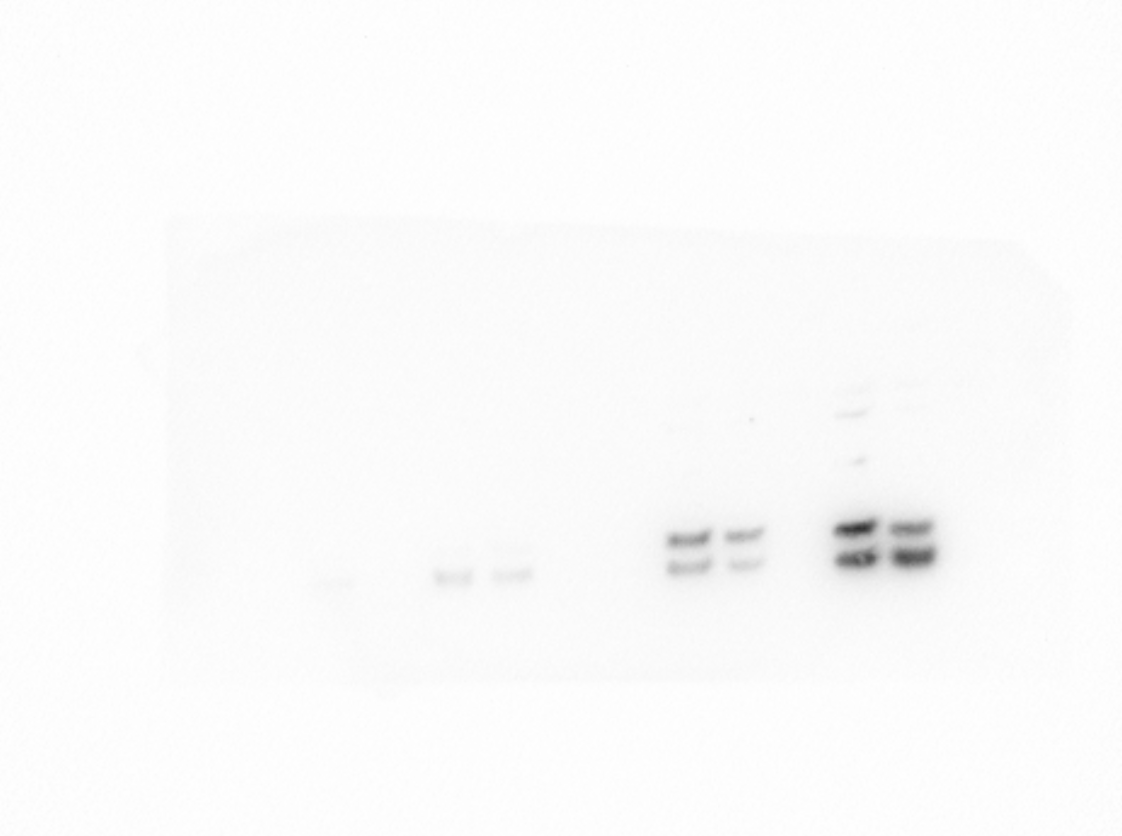

Supplement: Figure 6—source data 4. [file elife-74549-fig6-data4.zip › Soure 6c/gel 2/pERK figure.tif]

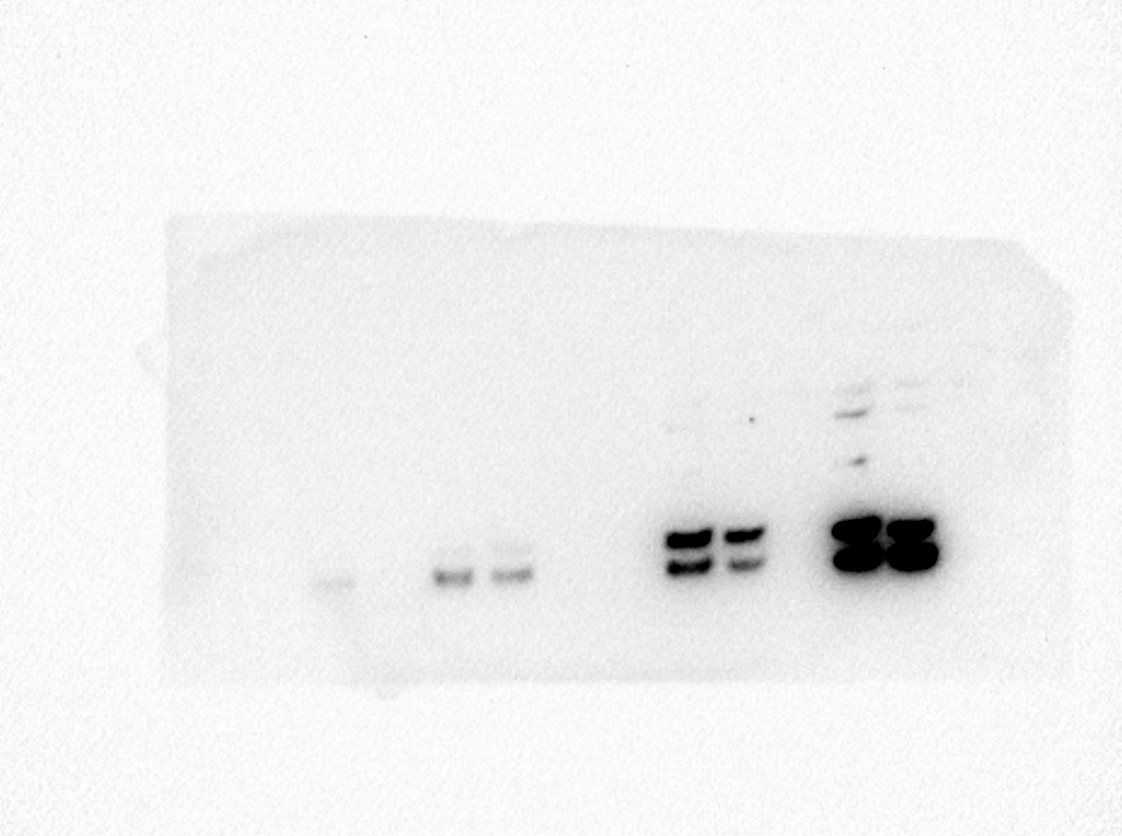

Supplement: Figure 6—source data 4. [file elife-74549-fig6-data4.zip › Soure 6c/gel 2/pERK figure_2.tif]

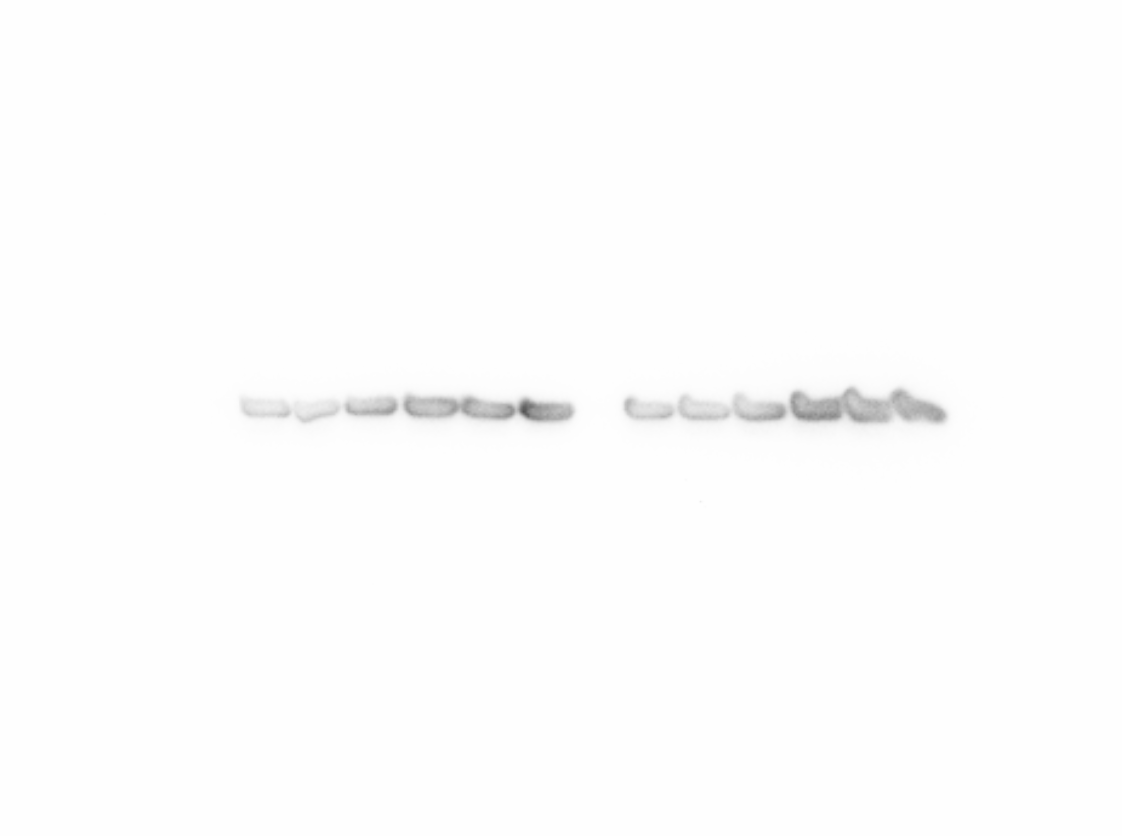

Supplement: Figure 6—source data 4. [file elife-74549-fig6-data4.zip › Soure 6c/gel 3/actin figure.tif]

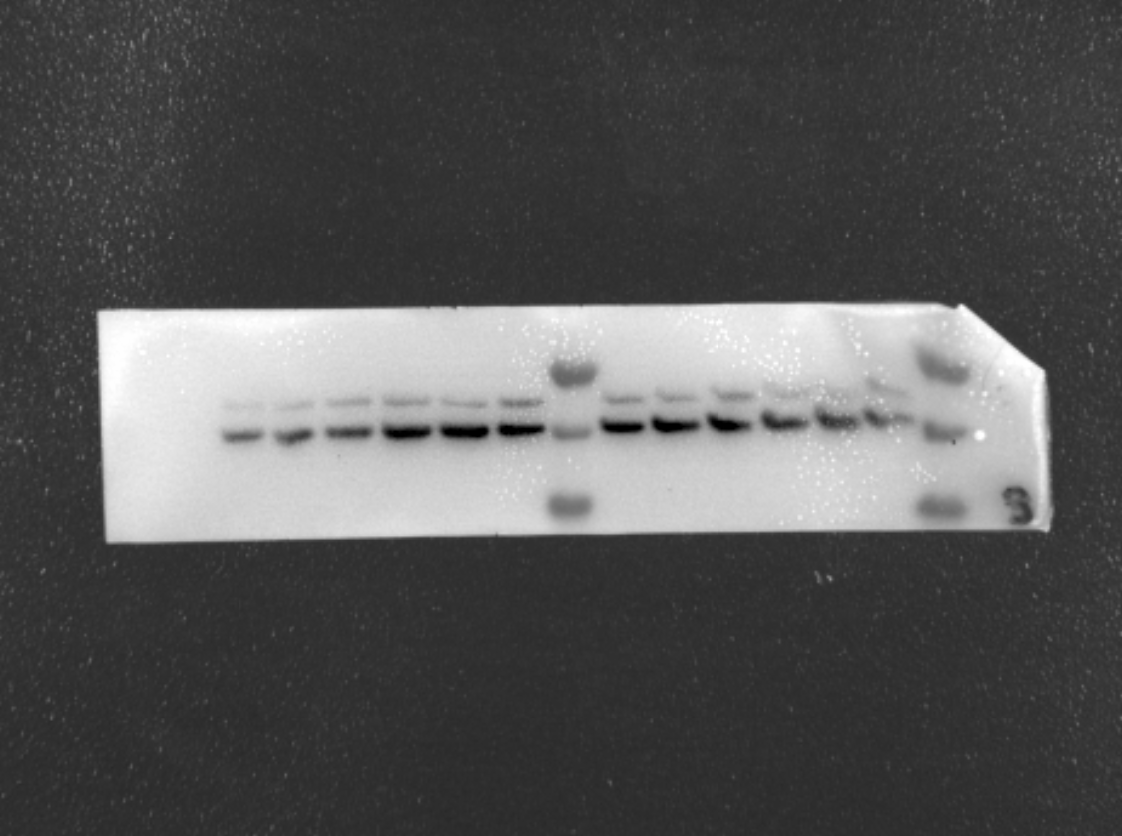

Supplement: Figure 6—source data 4. [file elife-74549-fig6-data4.zip › Soure 6c/gel 3/ERK figure.tif]
