# Supplementary material for: Redox regulation of PTPN22 affects the severity of T-cell-dependent autoimmune inflammation
Source: eLife. 2022 May 19;11:e74549. doi: 10.7554/eLife.74549 (PMC9119677; doi:10.7554/eLife.74549)
Supplement: Figure 2—source data 1. [file elife-74549-fig2-data1.docx]

Reduced PTPN22(black) and PTP1B(gray) was treated with 1mM H_2_O_2_ for 5 min and then with catalase to remove residual H_2_O_2_ and reactivated with 10mM DTT,TrxR1 (2.5µM) with or without NADPH (300µM) and Trx1 (10µM). After 60 min at 37 °C.

# 150

**PTP activity% of control**

**100**

# 50

**0**

| **Time (sec) Blank** | **Blank** | **no H2O2** |  | **no H2O2** |  | **+ H2O2** |  | **+ H2O2** |  | **+ DTT** |  | **+ DTT** |  | **TrxR1+NADPH** |  | **TrxR1+NADPH** |  | **TrxR1+Trx1+NADPH** |  | **TrxR1+Trx1+NADPH** |  | **TrxR1+Trx1** |  | **TrxR1+Trx1** |  |
| --- | --- | --- | --- | --- | --- | --- | --- | --- | --- | --- | --- | --- | --- | --- | --- | --- | --- | --- | --- | --- | --- | --- | --- | --- | --- |
| 20 | 0,235200003 0,232299998 |  | 0,254299998 |  | 0,250400007 |  | 0,238600001 |  | 0,2368 |  | 0,252400011 |  | 0,253899992 |  | 0,252499998 |  | 0,247500002 |  | 0,245299995 |  | 0,246700004 |  | 0,238600001 |  | 0,238600001 |
| 40 | 0,232999995 0,233899996 |  | 0,256900012 |  | 0,255299985 |  | 0,2377 |  | 0,238700002 |  | 0,256300002 |  | 0,259600013 |  | 0,254599988 |  | 0,252299994 |  | 0,246700004 |  | 0,25 |  | 0,2368 |  | 0,239099994 |
| 60 | 0,230499998 0,231999993 |  | 0,260399997 |  | 0,259299994 |  | 0,241699994 |  | 0,237100005 |  | 0,260399997 |  | 0,264899999 |  | 0,25909999 |  | 0,255899996 |  | 0,249899998 |  | 0,251300007 |  | 0,238999993 |  | 0,240400001 |
| 80 | 0,232899994 0,234999999 |  | 0,2667 |  | 0,264899999 |  | 0,242200002 |  | 0,240400001 |  | 0,266000003 |  | 0,2685 |  | 0,263500005 |  | 0,262899995 |  | 0,254000008 |  | 0,257200003 |  | 0,239899993 |  | 0,241300002 |
| 100 | 0,231299996 0,229699999 |  | 0,267699987 |  | 0,26789999 |  | 0,240400001 |  | 0,237900004 |  | 0,267699987 |  | 0,270799994 |  | 0,265500009 |  | 0,263599992 |  | 0,255699992 |  | 0,259799987 |  | 0,238600001 |  | 0,240700006 |
| 120 | 0,233099997 0,232899994 |  | 0,27610001 |  | 0,2764 |  | 0,243699998 |  | 0,243000001 |  | 0,273999989 |  | 0,278499991 |  | 0,273799986 |  | 0,269600004 |  | 0,260699987 |  | 0,264800012 |  | 0,241300002 |  | 0,243200004 |
| 140 | 0,233099997 0,233199999 |  | 0,280900002 |  | 0,280999988 |  | 0,245399997 |  | 0,243699998 |  | 0,278699994 |  | 0,283399999 |  | 0,27759999 |  | 0,274100006 |  | 0,263799995 |  | 0,271499991 |  | 0,241899997 |  | 0,243699998 |
| 160 | 0,2315 0,232800007 |  | 0,289200008 |  | 0,285600007 |  | 0,244599998 |  | 0,242699996 |  | 0,283800006 |  | 0,286599994 |  | 0,281399995 |  | 0,276199996 |  | 0,266400009 |  | 0,273000002 |  | 0,244299993 |  | 0,246199995 |
| 180 | 0,230900005 0,231700003 |  | 0,287600011 |  | 0,287800014 |  | 0,246000007 |  | 0,243699998 |  | 0,287400007 |  | 0,289600015 |  | 0,284200013 |  | 0,280600011 |  | 0,266200006 |  | 0,273299992 |  | 0,243799999 |  | 0,245499998 |
| 200 | 0,2315 0,2315 |  | 0,293199986 |  | 0,292699993 |  | 0,245800003 |  | 0,243599996 |  | 0,291000009 |  | 0,295300007 |  | 0,288100004 |  | 0,28580001 |  | 0,269800007 |  | 0,278899997 |  | 0,242400005 |  | 0,246800005 |
| 220 | 0,233099997 0,2324 |  | 0,301999986 |  | 0,300000012 |  | 0,249599993 |  | 0,247400001 |  | 0,296299994 |  | 0,300999999 |  | 0,295100003 |  | 0,290199995 |  | 0,276199996 |  | 0,282999992 |  | 0,246999994 |  | 0,249200001 |
| 240 | 0,231800005 0,231999993 |  | 0,306300014 |  | 0,30430001 |  | 0,248600006 |  | 0,246999994 |  | 0,299699992 |  | 0,302399993 |  | 0,297300011 |  | 0,293099999 |  | 0,277500004 |  | 0,287099987 |  | 0,246299997 |  | 0,248799995 |
| 260 | 0,232899994 0,231999993 |  | 0,310600013 |  | 0,308400005 |  | 0,249699995 |  | 0,247700006 |  | 0,305599988 |  | 0,308600008 |  | 0,301800013 |  | 0,299299985 |  | 0,279900014 |  | 0,290300012 |  | 0,247400001 |  | 0,250099987 |
| 280 | 0,233899996 0,235200003 |  | 0,316900015 |  | 0,314999998 |  | 0,251199991 |  | 0,249599993 |  | 0,311800003 |  | 0,315600008 |  | 0,309599996 |  | 0,304500014 |  | 0,285400003 |  | 0,29519999 |  | 0,251199991 |  | 0,253899992 |
| 300 | 0,233600006 0,232500002 |  | 0,321700007 |  | 0,319700003 |  | 0,252000004 |  | 0,250200003 |  | 0,313699991 |  | 0,317499995 |  | 0,310900003 |  | 0,305700004 |  | 0,287400007 |  | 0,298000008 |  | 0,252600014 |  | 0,251700014 |
| 320 | 0,2324 0,232700005 |  | 0,325599998 |  | 0,323199987 |  | 0,253100008 |  | 0,251300007 |  | 0,317600012 |  | 0,32100001 |  | 0,314599991 |  | 0,307799995 |  | 0,287299991 |  | 0,300300002 |  | 0,25060001 |  | 0,251100004 |
| 340 | 0,233899996 0,233400002 |  | 0,330199987 |  | 0,329600006 |  | 0,255199999 |  | 0,253500015 |  | 0,324200004 |  | 0,327300012 |  | 0,319999993 |  | 0,317000002 |  | 0,29339999 |  | 0,305599988 |  | 0,253500015 |  | 0,254500002 |
| 360 | 0,231900007 0,230599999 |  | 0,335299999 |  | 0,33160001 |  | 0,255299985 |  | 0,254500002 |  | 0,328999996 |  | 0,332899988 |  | 0,325899988 |  | 0,317999989 |  | 0,294999987 |  | 0,309100002 |  | 0,252700001 |  | 0,256300002 |
| slope | 5,52127E-07 -9,80378E-07 |  | 0,000246873 |  | 0,000244629 |  | 5,01754E-05 |  | 5,0774E-05 |  | 0,000224623 |  | 0,000226244 |  | 0,000217136 |  | 0,000208086 |  | 0,000149752 |  | 0,00018531 |  | 4,98194E-05 |  | 5,09959E-05 |
| slope w/o |  |  | 0,000247087 |  | 0,000244843 |  | 5,03896E-05 |  | 5,09881E-05 |  | 0,000224837 |  | 0,000226458 |  | 0,00021735 |  | 0,0002083 |  | 0,000149966 |  | 0,000185524 |  | 5,00336E-05 |  | 5,121E-05 |
| blank | |  |  | 0,000245965 | |  | | 5,06888E-05 | |  | | 0,000225648 | |  | | 0,000212825 | |  | | 0,000167745 | |  | | 5,06218E-05 | |
|  | |  |  | 0,014757895 | |  | | 0,003041331 | |  | | 0,013538854 | |  | | 0,012769504 | |  | | 0,010064704 | |  | | 0,003037307 | |
|  | |  |  | 2,202670857 | |  | | 0,45392993 | |  | | 2,02072447 | |  | | 1,905896119 | |  | | 1,502194698 | |  | | 0,453329363 | |
|  | |  |  | 15,73336326 | |  | | 3,242356641 | |  | | 14,43374621 | |  | | 13,61354371 | |  | | 10,72996213 | |  | | 3,238066877 | |
|  | | **min -1** |  | 15,73336326 | |  | | 3,242356641 | |  | | 14,43374621 | |  | | 13,61354371 | |  | | 10,72996213 | |  | | 3,238066877 | |

|  | **5 min** |  |  |  |  |  | 30 |  | | |
| --- | --- | --- | --- | --- | --- | --- | --- | --- | --- | --- |
| **no H2O2** | 15,73336326 | 100 | 100 |  |  |  | 76,05790021 |  |  |  |
| **+ H2O2** | 3,242356641 | 20,60815978 | 20,60815978 |  |  |  | 4,469147798 |  |  |  |
| **+ DTT** | 14,43374621 | 91,73973786 | 91,73973786 |  |  |  | 97,26724967 |  |  |  |
| **TrxR1+NADPH** | 13,61354371 | 86,52659625 | 86,52659625 |  |  |  | 31,22145889 |  |  |  |
| **TrxR1+Trx1+NADPH** | 10,72996213 | 68,1987821 | 68,1987821 |  |  |  |  |  |  |  |
| **TrxR1+Trx1** | 3,238066877 | 20,58089439 | 20,58089439 |  |  |  |  |  |  |  |
|  | #DIV/0! |  |  |  |  |  |  |  |  |  |
|  | #DIV/0! |  |  | **no H2O2** | **+ H2O2** | **+ DTT** |  | **TrxR1+NADPH** | **TrxR1+Trx1+NADPH** | **TrxR1+Trx1** |

| Application: Tecan i-control  Device: infinite 200Pro | | Tecan i-control , 2.0.10.0  Serial number: 1307001123 | Serial number of connected stacker: |
| --- | --- | --- | --- |
| Firmware: V_3.40_01/15_Infinite (Dec 23 201 MAI, V_3.40_01/15_Infinite (Dec 23 2014/12.45.11) | | | |
| Date: | ######### | | |
| Time: | 14:25:37 | | |

System MTC-MU059-S

User MTC-MU059-S\fretho

Plate Greiner 96 Flat Bottom Transparent Polystyrene Cat. No.: 655101/655161/655192 [GRE96ft.pdfx] Plate-ID (Stacker)

List of actions in this measurement script: Kinetic

Absorbance

Shaking (Linear) Duration: Shaking (Linear) Amplitude:

3 s

1 mm

Label: Label1

Kinetic Measurement

Kinetic duration 00:06:00

Interval Time 00:00:20

Measurement Wavelength 405 nm

Bandwidth 10 nm

Number of Flashes 5

Settle Time 0 ms

Part of Plate A1-B12; C1-C2

Start Time: 2020 08-11 14:25:40

| Cycle Nr. | 1 | 2 | 3 | 4 | 5 | 6 | 7 | 8 | 9 | 10 | 11 | 12 | 13 | 14 | 15 | 16 | 17 | 18 | 19 |
| --- | --- | --- | --- | --- | --- | --- | --- | --- | --- | --- | --- | --- | --- | --- | --- | --- | --- | --- | --- |
| Time [s] | 0 | 20 | 40 | 60 | 80 | 100 | 120 | 140 | 160 | 180 | 200 | 220 | 240 | 260 | 280 | 300 | 320 | 340 | 360 |
| Temp. [°C] | 25,9 | 26,1 | 26,3 | 25,9 | 26,1 | 26,1 | 26,3 | 26,1 | 25,9 | 25,8 | 26,3 | 26,3 | 26 | 26,3 | 26,1 | 26,1 | 26,1 | 26 | 25,8 |
| A1 | 0,2504 | 0,2543 | 0,2569 | 0,2604 | 0,2667 | 0,2677 | 0,2761 | 0,2809 | 0,2892 | 0,2876 | 0,2932 | 0,302 | 0,3063 | 0,3106 | 0,3169 | 0,3217 | 0,3256 | 0,3302 | 0,3353 |
| A2 | 0,2483 | 0,2504 | 0,2553 | 0,2593 | 0,2649 | 0,2679 | 0,2764 | 0,281 | 0,2856 | 0,2878 | 0,2927 | 0,3 | 0,3043 | 0,3084 | 0,315 | 0,3197 | 0,3232 | 0,3296 | 0,3316 |
| A3 | 0,2402 | 0,2386 | 0,2377 | 0,2417 | 0,2422 | 0,2404 | 0,2437 | 0,2454 | 0,2446 | 0,246 | 0,2458 | 0,2496 | 0,2486 | 0,2497 | 0,2512 | 0,252 | 0,2531 | 0,2552 | 0,2553 |
| A4 | 0,2386 | 0,2368 | 0,2387 | 0,2371 | 0,2404 | 0,2379 | 0,243 | 0,2437 | 0,2427 | 0,2437 | 0,2436 | 0,2474 | 0,247 | 0,2477 | 0,2496 | 0,2502 | 0,2513 | 0,2535 | 0,2545 |
| A5 | 0,2504 | 0,2524 | 0,2563 | 0,2604 | 0,266 | 0,2677 | 0,274 | 0,2787 | 0,2838 | 0,2874 | 0,291 | 0,2963 | 0,2997 | 0,3056 | 0,3118 | 0,3137 | 0,3176 | 0,3242 | 0,329 |
| A6 | 0,2544 | 0,2539 | 0,2596 | 0,2649 | 0,2685 | 0,2708 | 0,2785 | 0,2834 | 0,2866 | 0,2896 | 0,2953 | 0,301 | 0,3024 | 0,3086 | 0,3156 | 0,3175 | 0,321 | 0,3273 | 0,3329 |
| A7 | 0,249 | 0,2525 | 0,2546 | 0,2591 | 0,2635 | 0,2655 | 0,2738 | 0,2776 | 0,2814 | 0,2842 | 0,2881 | 0,2951 | 0,2973 | 0,3018 | 0,3096 | 0,3109 | 0,3146 | 0,32 | 0,3259 |
| A8 | 0,2471 | 0,2475 | 0,2523 | 0,2559 | 0,2629 | 0,2636 | 0,2696 | 0,2741 | 0,2762 | 0,2806 | 0,2858 | 0,2902 | 0,2931 | 0,2993 | 0,3045 | 0,3057 | 0,3078 | 0,317 | 0,318 |
| A9 | 0,2456 | 0,2453 | 0,2467 | 0,2499 | 0,254 | 0,2557 | 0,2607 | 0,2638 | 0,2664 | 0,2662 | 0,2698 | 0,2762 | 0,2775 | 0,2799 | 0,2854 | 0,2874 | 0,2873 | 0,2934 | 0,295 |
| A10 | 0,2489 | 0,2467 | 0,25 | 0,2513 | 0,2572 | 0,2598 | 0,2648 | 0,2715 | 0,273 | 0,2733 | 0,2789 | 0,283 | 0,2871 | 0,2903 | 0,2952 | 0,298 | 0,3003 | 0,3056 | 0,3091 |
| A11 | 0,2374 | 0,2386 | 0,2368 | 0,239 | 0,2399 | 0,2386 | 0,2413 | 0,2419 | 0,2443 | 0,2438 | 0,2424 | 0,247 | 0,2463 | 0,2474 | 0,2512 | 0,2526 | 0,2506 | 0,2535 | 0,2527 |
| A12 | 0,239 | 0,2386 | 0,2391 | 0,2404 | 0,2413 | 0,2407 | 0,2432 | 0,2437 | 0,2462 | 0,2455 | 0,2468 | 0,2492 | 0,2488 | 0,2501 | 0,2539 | 0,2517 | 0,2511 | 0,2545 | 0,2563 |
| B1 | 0,3034 | 0,3698 | 0,4265 | 0,4826 | 0,5416 | 0,5955 | 0,6579 | 0,7148 | 0,77 | 0,8252 | 0,8859 | 0,945 | 0,9992 | 1,0573 | 1,116 | 1,1733 | 1,2295 | 1,2864 | 1,3458 |
| B2 | 0,3207 | 0,384 | 0,4451 | 0,5099 | 0,5707 | 0,6306 | 0,6948 | 0,7577 | 0,8199 | 0,8784 | 0,9406 | 1,0065 | 1,0672 | 1,1286 | 1,1923 | 1,2535 | 1,3145 | 1,3774 | 1,4407 |
| B3 | 0,2408 | 0,2331 | 0,2326 | 0,2321 | 0,234 | 0,2307 | 0,2346 | 0,2357 | 0,2333 | 0,2319 | 0,2326 | 0,2356 | 0,2321 | 0,2366 | 0,2355 | 0,2352 | 0,233 | 0,2352 | 0,2345 |
| B4 | 0,2364 | 0,2341 | 0,2333 | 0,2346 | 0,2355 | 0,2315 | 0,2355 | 0,2351 | 0,2361 | 0,2339 | 0,2342 | 0,2365 | 0,2321 | 0,2345 | 0,2433 | 0,2351 | 0,2336 | 0,2349 | 0,2359 |
| B5 | 0,2935 | 0,3392 | 0,3833 | 0,428 | 0,4731 | 0,5142 | 0,5639 | 0,6069 | 0,6525 | 0,6941 | 0,7382 | 0,7856 | 0,8281 | 0,8731 | 0,9187 | 0,9624 | 1,0054 | 1,0524 | 1,0977 |
| B6 | 0,296 | 0,3408 | 0,3832 | 0,4282 | 0,475 | 0,5133 | 0,5619 | 0,6074 | 0,6542 | 0,6896 | 0,7352 | 0,7818 | 0,8243 | 0,8716 | 0,9152 | 0,9573 | 1,0005 | 1,0436 | 1,0891 |
| B7 | 0,2628 | 0,273 | 0,2876 | 0,3013 | 0,3166 | 0,3301 | 0,3468 | 0,3625 | 0,3765 | 0,3893 | 0,4028 | 0,4219 | 0,4346 | 0,4492 | 0,4663 | 0,4782 | 0,4908 | 0,5096 | 0,5245 |
| B8 | 0,258 | 0,2685 | 0,2814 | 0,2932 | 0,3094 | 0,3189 | 0,3356 | 0,3476 | 0,362 | 0,3728 | 0,3862 | 0,4027 | 0,4164 | 0,4289 | 0,4443 | 0,4562 | 0,4681 | 0,4827 | 0,496 |
| B9 | 0,2893 | 0,3195 | 0,3526 | 0,3887 | 0,4234 | 0,4577 | 0,4943 | 0,5296 | 0,5643 | 0,599 | 0,6342 | 0,6707 | 0,7052 | 0,7418 | 0,7777 | 0,8113 | 0,8444 | 0,8827 | 0,9187 |
| B10 | 0,2811 | 0,3064 | 0,3352 | 0,3666 | 0,3963 | 0,4252 | 0,4583 | 0,4896 | 0,5209 | 0,5484 | 0,5785 | 0,6122 | 0,6403 | 0,6722 | 0,7044 | 0,7309 | 0,7606 | 0,795 | 0,8248 |
| B11 | 0,2372 | 0,2345 | 0,2354 | 0,237 | 0,2445 | 0,235 | 0,2397 | 0,2399 | 0,239 | 0,2374 | 0,2391 | 0,2417 | 0,2421 | 0,2434 | 0,245 | 0,2438 | 0,2439 | 0,2473 | 0,2434 |
| B12 | 0,2399 | 0,2393 | 0,2393 | 0,2375 | 0,2401 | 0,2391 | 0,2414 | 0,2447 | 0,2425 | 0,2427 | 0,2429 | 0,2449 | 0,2447 | 0,2471 | 0,2505 | 0,2469 | 0,247 | 0,2509 | 0,2492 |
| C1 | 0,2387 | 0,2352 | 0,233 | 0,2305 | 0,2329 | 0,2313 | 0,2331 | 0,2331 | 0,2315 | 0,2309 | 0,2315 | 0,2331 | 0,2318 | 0,2329 | 0,2339 | 0,2336 | 0,2324 | 0,2339 | 0,2319 |
| C2 | 0,2407 | 0,2323 | 0,2339 | 0,232 | 0,235 | 0,2297 | 0,2329 | 0,2332 | 0,2328 | 0,2317 | 0,2315 | 0,2324 | 0,232 | 0,232 | 0,2352 | 0,2325 | 0,2327 | 0,2334 | 0,2306 |

| **Time (sec) Blank** | **Blank** | **no H2O2** |  | **no H2O2** |  | **+ H2O2** |  | **+ H2O2** |  | **+ DTT** |  | **+ DTT** |  | **TrxR1+NADPH** |  | **TrxR1+NADPH** |  | **TrxR1+Trx1+NADPH** |  | **TrxR1+Trx1+NADPH** |  | **TrxR1+Trx1** |  | **TrxR1+Trx1** |  |
| --- | --- | --- | --- | --- | --- | --- | --- | --- | --- | --- | --- | --- | --- | --- | --- | --- | --- | --- | --- | --- | --- | --- | --- | --- | --- |
| 20 | 0,229599997 0,233500004 |  | 0,247199997 |  | 0,248199999 |  | 0,235599995 |  | 0,238299996 |  | 0,247500002 |  | 0,25060001 |  | 0,252400011 |  | 0,256799996 |  | 0,252900004 |  | 0,253600001 |  | 0,246099994 |  | 0,244599998 |
| 40 | 0,229499996 0,230499998 |  | 0,249699995 |  | 0,247500002 |  | 0,234999999 |  | 0,234400004 |  | 0,248500004 |  | 0,250800014 |  | 0,253399998 |  | 0,255299985 |  | 0,251599997 |  | 0,253500015 |  | 0,242200002 |  | 0,240500003 |
| 60 | 0,225999996 0,229499996 |  | 0,251399994 |  | 0,253199995 |  | 0,236399993 |  | 0,236499995 |  | 0,251800001 |  | 0,253399998 |  | 0,257200003 |  | 0,260399997 |  | 0,256799996 |  | 0,255299985 |  | 0,245299995 |  | 0,243699998 |
| 80 | 0,227799997 0,229399994 |  | 0,256700009 |  | 0,256799996 |  | 0,236699998 |  | 0,237299994 |  | 0,254599988 |  | 0,257099986 |  | 0,259600013 |  | 0,262400001 |  | 0,256199986 |  | 0,258700013 |  | 0,246999994 |  | 0,244900003 |
| 100 | 0,226899996 0,227200001 |  | 0,260199994 |  | 0,257999986 |  | 0,235699996 |  | 0,234799996 |  | 0,258100003 |  | 0,26030001 |  | 0,263599992 |  | 0,264499992 |  | 0,25819999 |  | 0,25850001 |  | 0,244599998 |  | 0,243499994 |
| 120 | 0,225299999 0,227400005 |  | 0,262499988 |  | 0,262699991 |  | 0,238399997 |  | 0,236300007 |  | 0,260899991 |  | 0,261400014 |  | 0,265300006 |  | 0,268099993 |  | 0,261200011 |  | 0,260600001 |  | 0,242899999 |  | 0,245499998 |
| 140 | 0,226600006 0,229499996 |  | 0,266600013 |  | 0,265399992 |  | 0,240799993 |  | 0,237599999 |  | 0,265399992 |  | 0,265599996 |  | 0,270099998 |  | 0,272199988 |  | 0,263599992 |  | 0,264600009 |  | 0,245199993 |  | 0,2456 |
| 160 | 0,227699995 0,229699999 |  | 0,270000011 |  | 0,272799999 |  | 0,244599998 |  | 0,235699996 |  | 0,265300006 |  | 0,268999994 |  | 0,272700012 |  | 0,27700001 |  | 0,266099989 |  | 0,26699999 |  | 0,246900007 |  | 0,244900003 |
| 180 | 0,226300001 0,230700001 |  | 0,275400013 |  | 0,274500012 |  | 0,242799997 |  | 0,239199996 |  | 0,269300014 |  | 0,269800007 |  | 0,273799986 |  | 0,279100001 |  | 0,26879999 |  | 0,268999994 |  | 0,245299995 |  | 0,247099996 |
| 200 | 0,227500007 0,228599995 |  | 0,278800011 |  | 0,276300013 |  | 0,243799999 |  | 0,2368 |  | 0,271800011 |  | 0,272700012 |  | 0,278400004 |  | 0,282099992 |  | 0,270999998 |  | 0,271400005 |  | 0,247700006 |  | 0,246000007 |
| 220 | 0,227599993 0,230599999 |  | 0,282900006 |  | 0,28459999 |  | 0,243900001 |  | 0,239500001 |  | 0,275200009 |  | 0,277799994 |  | 0,281800002 |  | 0,284700006 |  | 0,273999989 |  | 0,275700003 |  | 0,2491 |  | 0,246099994 |
| 240 | 0,228599995 0,230900005 |  | 0,286900014 |  | 0,287400007 |  | 0,244000003 |  | 0,2412 |  | 0,279000014 |  | 0,285100013 |  | 0,28639999 |  | 0,288100004 |  | 0,27700001 |  | 0,277700007 |  | 0,251700014 |  | 0,247999996 |
| 260 | 0,227699995 0,231299996 |  | 0,288800001 |  | 0,288399994 |  | 0,242500007 |  | 0,238700002 |  | 0,279700011 |  | 0,284399986 |  | 0,28490001 |  | 0,288700014 |  | 0,278699994 |  | 0,278899997 |  | 0,249500006 |  | 0,244900003 |
| 280 | 0,227200001 0,227899998 |  | 0,290399998 |  | 0,290399998 |  | 0,238299996 |  | 0,238999993 |  | 0,280800015 |  | 0,28459999 |  | 0,287400007 |  | 0,291500002 |  | 0,277500004 |  | 0,278800011 |  | 0,247500002 |  | 0,246900007 |
| 300 | 0,228599995 0,231999993 |  | 0,297100008 |  | 0,298299998 |  | 0,243499994 |  | 0,241899997 |  | 0,286300004 |  | 0,289700001 |  | 0,294200003 |  | 0,296700001 |  | 0,283199996 |  | 0,283399999 |  | 0,251300007 |  | 0,248199999 |
| 320 | 0,226699993 0,228400007 |  | 0,298500001 |  | 0,297800004 |  | 0,240199998 |  | 0,240099996 |  | 0,289099991 |  | 0,289799988 |  | 0,293599993 |  | 0,296700001 |  | 0,283399999 |  | 0,285699993 |  | 0,248699993 |  | 0,249400005 |
| 340 | 0,227899998 0,228799999 |  | 0,30430001 |  | 0,307300001 |  | 0,243399993 |  | 0,2412 |  | 0,290100008 |  | 0,296999991 |  | 0,298999995 |  | 0,300799996 |  | 0,287099987 |  | 0,288899988 |  | 0,249500006 |  | 0,251100004 |
| 360 | 0,229100004 0,229699999 |  | 0,306800008 |  | 0,307700008 |  | 0,243399993 |  | 0,240999997 |  | 0,293599993 |  | 0,296400011 |  | 0,301899999 |  | 0,304899991 |  | 0,287600011 |  | 0,291200012 |  | 0,250800014 |  | 0,249599993 |
| slope | 8,15277E-07 -1,78534E-06 |  | 0,00018 |  | 0,000183875 |  | 2,32095E-05 |  | 1,68679E-05 |  | 0,000138153 |  | 0,00014499 |  | 0,000146754 |  | 0,000146656 |  | 0,000111161 |  | 0,000115681 |  | 2,03664E-05 |  | 2,03973E-05 |
| slope w/o |  |  | 0,000180485 |  | 0,00018436 |  | 2,36945E-05 |  | 1,73529E-05 |  | 0,000138638 |  | 0,000145475 |  | 0,000147239 |  | 0,000147141 |  | 0,000111646 |  | 0,000116166 |  | 2,08514E-05 |  | 2,08824E-05 |
| blank | |  |  |  | 0,000182423 |  |  |  | 2,05237E-05 |  |  |  | 0,000142056 |  |  |  | 0,00014719 |  |  |  | 0,000113906 |  |  |  | 2,08669E-05 |
|  | |  |  |  | 0,010945358 |  |  |  | 0,001231424 |  |  |  | 0,008523374 |  |  |  | 0,008831424 |  |  |  | 0,006834365 |  |  |  | 0,001252013 |
|  | |  |  |  | 1,633635458 |  |  |  | 0,183794624 |  |  |  | 1,272145382 |  |  |  | 1,318122997 |  |  |  | 1,0200545 |  |  |  | 0,186867685 |
|  | |  |  |  | 11,6688247 |  |  |  | 1,312818742 |  |  |  | 9,086752727 |  |  |  | 9,415164264 |  |  |  | 7,286103572 |  |  |  | 1,334769175 |
|  | | **min -1** |  |  | 11,6688247 |  |  |  | 1,312818742 |  |  |  | 9,086752727 |  |  |  | 9,415164264 |  |  |  | 7,286103572 |  |  |  | 1,334769175 |

|  | **5 min** |  |  |  |  |  | 30 |  | | |
| --- | --- | --- | --- | --- | --- | --- | --- | --- | --- | --- |
| **no H2O2** | 11,6688247 | 100 | 100 |  |  |  | 76,05790021 |  |  |  |
| **+ H2O2** | 1,312818742 | 11,25065099 | 11,25065099 |  |  |  | 4,469147798 |  |  |  |
| **+ DTT** | 9,086752727 | 77,87204763 | 77,87204763 |  |  |  | 97,26724967 |  |  |  |
| **TrxR1+NADPH** | 9,415164264 | 80,68648306 | 80,68648306 |  |  |  | 31,22145889 |  |  |  |
| **TrxR1+Trx1+NADPH** | 7,286103572 | 62,44076641 | 62,44076641 |  |  |  |  |  |  |  |
| **TrxR1+Trx1** | 1,334769175 | 11,43876277 | 11,43876277 |  |  |  |  |  |  |  |
|  | #DIV/0! |  |  |  |  |  |  |  |  |  |
|  | #DIV/0! |  |  | **no H2O2** | **+ H2O2** | **+ DTT** |  | **TrxR1+NADPH** | **TrxR1+Trx1+NADPH** | **TrxR1+Trx1** |

| Application: Tecan i-control  Device: infinite 200Pro | | Tecan i-control , 2.0.10.0  Serial number: 1307001123 | Serial number of connected stacker: |
| --- | --- | --- | --- |
| Firmware: V_3.40_01/15_Infinite (Dec 23 201 MAI, V_3.40_01/15_Infinite (Dec 23 2014/12.45.11) | | | |
| Date: | ######### | | |
| Time: | 15:08:20 | | |

System MTC-MU059-S

User MTC-MU059-S\fretho

Plate Greiner 96 Flat Bottom Transparent Polystyrene Cat. No.: 655101/655161/655192 [GRE96ft.pdfx] Plate-ID (Stacker)

List of actions in this measurement script: Kinetic

Absorbance

Shaking (Linear) Duration: Shaking (Linear) Amplitude:

3 s

1 mm

Label: Label1

Kinetic Measurement

Kinetic duration 00:06:00

Interval Time 00:00:20

Measurement Wavelength 405 nm

Bandwidth 10 nm

Number of Flashes 5

Settle Time 0 ms

Part of Plate A1-B12; C1-C2

Start Time: 2020 08-13 15:08:22

| Cycle Nr. | 1 | 2 | 3 | 4 | 5 | 6 | 7 | 8 | 9 | 10 | 11 | 12 | 13 | 14 | 15 | 16 | 17 | 18 | 19 |
| --- | --- | --- | --- | --- | --- | --- | --- | --- | --- | --- | --- | --- | --- | --- | --- | --- | --- | --- | --- |
| Time [s] | 0 | 20 | 40 | 60 | 80 | 100 | 120 | 140 | 160 | 180 | 200 | 220 | 240 | 260 | 280 | 300 | 320 | 340 | 360 |
| Temp. [°C] | 25,5 | 25,7 | 25,6 | 25,7 | 25,6 | 25,7 | 25,8 | 25,7 | 25,9 | 25,8 | 25,8 | 25,8 | 26,1 | 25,8 | 25,8 | 25,9 | 25,8 | 25,8 | 26,1 |
| A1 | 0,2478 | 0,2472 | 0,2497 | 0,2514 | 0,2567 | 0,2602 | 0,2625 | 0,2666 | 0,27 | 0,2754 | 0,2788 | 0,2829 | 0,2869 | 0,2888 | 0,2904 | 0,2971 | 0,2985 | 0,3043 | 0,3068 |
| A2 | 0,2473 | 0,2482 | 0,2475 | 0,2532 | 0,2568 | 0,258 | 0,2627 | 0,2654 | 0,2728 | 0,2745 | 0,2763 | 0,2846 | 0,2874 | 0,2884 | 0,2904 | 0,2983 | 0,2978 | 0,3073 | 0,3077 |
| A3 | 0,2411 | 0,2356 | 0,235 | 0,2364 | 0,2367 | 0,2357 | 0,2384 | 0,2408 | 0,2446 | 0,2428 | 0,2438 | 0,2439 | 0,244 | 0,2425 | 0,2383 | 0,2435 | 0,2402 | 0,2434 | 0,2434 |
| A4 | 0,2425 | 0,2383 | 0,2344 | 0,2365 | 0,2373 | 0,2348 | 0,2363 | 0,2376 | 0,2357 | 0,2392 | 0,2368 | 0,2395 | 0,2412 | 0,2387 | 0,239 | 0,2419 | 0,2401 | 0,2412 | 0,241 |
| A5 | 0,2474 | 0,2475 | 0,2485 | 0,2518 | 0,2546 | 0,2581 | 0,2609 | 0,2654 | 0,2653 | 0,2693 | 0,2718 | 0,2752 | 0,279 | 0,2797 | 0,2808 | 0,2863 | 0,2891 | 0,2901 | 0,2936 |
| A6 | 0,2485 | 0,2506 | 0,2508 | 0,2534 | 0,2571 | 0,2603 | 0,2614 | 0,2656 | 0,269 | 0,2698 | 0,2727 | 0,2778 | 0,2851 | 0,2844 | 0,2846 | 0,2897 | 0,2898 | 0,297 | 0,2964 |
| A7 | 0,2506 | 0,2524 | 0,2534 | 0,2572 | 0,2596 | 0,2636 | 0,2653 | 0,2701 | 0,2727 | 0,2738 | 0,2784 | 0,2818 | 0,2864 | 0,2849 | 0,2874 | 0,2942 | 0,2936 | 0,299 | 0,3019 |
| A8 | 0,2529 | 0,2568 | 0,2553 | 0,2604 | 0,2624 | 0,2645 | 0,2681 | 0,2722 | 0,277 | 0,2791 | 0,2821 | 0,2847 | 0,2881 | 0,2887 | 0,2915 | 0,2967 | 0,2967 | 0,3008 | 0,3049 |
| A9 | 0,2483 | 0,2529 | 0,2516 | 0,2568 | 0,2562 | 0,2582 | 0,2612 | 0,2636 | 0,2661 | 0,2688 | 0,271 | 0,274 | 0,277 | 0,2787 | 0,2775 | 0,2832 | 0,2834 | 0,2871 | 0,2876 |
| A10 | 0,2508 | 0,2536 | 0,2535 | 0,2553 | 0,2587 | 0,2585 | 0,2606 | 0,2646 | 0,267 | 0,269 | 0,2714 | 0,2757 | 0,2777 | 0,2789 | 0,2788 | 0,2834 | 0,2857 | 0,2889 | 0,2912 |
| A11 | 0,2455 | 0,2461 | 0,2422 | 0,2453 | 0,247 | 0,2446 | 0,2429 | 0,2452 | 0,2469 | 0,2453 | 0,2477 | 0,2491 | 0,2517 | 0,2495 | 0,2475 | 0,2513 | 0,2487 | 0,2495 | 0,2508 |
| A12 | 0,2439 | 0,2446 | 0,2405 | 0,2437 | 0,2449 | 0,2435 | 0,2455 | 0,2456 | 0,2449 | 0,2471 | 0,246 | 0,2461 | 0,248 | 0,2449 | 0,2469 | 0,2482 | 0,2494 | 0,2511 | 0,2496 |
| B1 | 0,3537 | 0,4181 | 0,47 | 0,5266 | 0,5835 | 0,639 | 0,6953 | 0,7521 | 0,808 | 0,8655 | 0,9195 | 0,9767 | 1,0332 | 1,0884 | 1,1416 | 1,2 | 1,2546 | 1,3136 | 1,3678 |
| B2 | 0,3648 | 0,4307 | 0,4886 | 0,5491 | 0,6084 | 0,67 | 0,7296 | 0,7901 | 0,8489 | 0,913 | 0,9727 | 1,0335 | 1,0928 | 1,1516 | 1,2095 | 1,2714 | 1,3286 | 1,3908 | 1,4494 |
| B3 | 0,2387 | 0,2364 | 0,2326 | 0,2338 | 0,2328 | 0,2333 | 0,2349 | 0,2369 | 0,2352 | 0,2372 | 0,2342 | 0,2347 | 0,2358 | 0,2339 | 0,2328 | 0,2349 | 0,2324 | 0,2332 | 0,2333 |
| B4 | 0,2405 | 0,2376 | 0,2344 | 0,2353 | 0,2356 | 0,2332 | 0,2319 | 0,2341 | 0,2341 | 0,234 | 0,2352 | 0,2343 | 0,236 | 0,2347 | 0,2327 | 0,2362 | 0,234 | 0,2351 | 0,2345 |
| B5 | 0,317 | 0,3547 | 0,3868 | 0,4214 | 0,4599 | 0,4947 | 0,5324 | 0,5684 | 0,6036 | 0,6394 | 0,6741 | 0,7086 | 0,7493 | 0,7818 | 0,8157 | 0,8541 | 0,8866 | 0,9259 | 0,9608 |
| B6 | 0,3194 | 0,3592 | 0,3918 | 0,4293 | 0,4626 | 0,4992 | 0,5361 | 0,5713 | 0,6074 | 0,6434 | 0,6784 | 0,7148 | 0,7511 | 0,7852 | 0,8181 | 0,8565 | 0,8903 | 0,9279 | 0,9622 |
| B7 | 0,2613 | 0,2689 | 0,2755 | 0,2812 | 0,2865 | 0,2946 | 0,3006 | 0,3095 | 0,315 | 0,3249 | 0,334 | 0,3394 | 0,3492 | 0,3548 | 0,3581 | 0,3676 | 0,3753 | 0,3837 | 0,3903 |
| B8 | 0,26 | 0,267 | 0,2688 | 0,2762 | 0,2844 | 0,2896 | 0,2953 | 0,3024 | 0,3108 | 0,3159 | 0,3237 | 0,3298 | 0,3379 | 0,3421 | 0,3478 | 0,3562 | 0,3609 | 0,3691 | 0,3741 |
| B9 | 0,3033 | 0,3258 | 0,346 | 0,3708 | 0,3937 | 0,4195 | 0,4424 | 0,4709 | 0,4952 | 0,5208 | 0,5404 | 0,5697 | 0,5945 | 0,6181 | 0,6406 | 0,6657 | 0,6899 | 0,7131 | 0,7399 |
| B10 | 0,3122 | 0,3355 | 0,3608 | 0,39 | 0,418 | 0,4444 | 0,4753 | 0,5063 | 0,5341 | 0,5617 | 0,589 | 0,6199 | 0,649 | 0,6757 | 0,7021 | 0,7343 | 0,7602 | 0,7903 | 0,8176 |
| B11 | 0,2464 | 0,2468 | 0,2441 | 0,2439 | 0,2437 | 0,2422 | 0,2435 | 0,2458 | 0,244 | 0,2461 | 0,2449 | 0,2488 | 0,2483 | 0,247 | 0,244 | 0,2474 | 0,2454 | 0,2471 | 0,245 |
| B12 | 0,2478 | 0,2483 | 0,2445 | 0,2457 | 0,2448 | 0,2442 | 0,2431 | 0,2446 | 0,2546 | 0,2438 | 0,2449 | 0,2489 | 0,2478 | 0,2462 | 0,2457 | 0,2479 | 0,25 | 0,2452 | 0,2501 |
| C1 | 0,2354 | 0,2296 | 0,2295 | 0,226 | 0,2278 | 0,2269 | 0,2253 | 0,2266 | 0,2277 | 0,2263 | 0,2275 | 0,2276 | 0,2286 | 0,2277 | 0,2272 | 0,2286 | 0,2267 | 0,2279 | 0,2291 |
| C2 | 0,2402 | 0,2335 | 0,2305 | 0,2295 | 0,2294 | 0,2272 | 0,2274 | 0,2295 | 0,2297 | 0,2307 | 0,2286 | 0,2306 | 0,2309 | 0,2313 | 0,2279 | 0,232 | 0,2284 | 0,2288 | 0,2297 |

| **Time (sec) Blank** | **Blank** | **no H2O2** |  | **no H2O2** |  | **+ H2O2** |  | **+ H2O2** |  | **+ DTT** |  | **+ DTT** |  | **TrxR1+NADPH** |  | **TrxR1+NADPH** |  | **TrxR1+Trx1+NADPH** |  | **TrxR1+Trx1+NADPH** |  | **TrxR1+Trx1** |  | **TrxR1+Trx1** |  |
| --- | --- | --- | --- | --- | --- | --- | --- | --- | --- | --- | --- | --- | --- | --- | --- | --- | --- | --- | --- | --- | --- | --- | --- | --- | --- |
| 20 | 0,260600001 0,233500004 |  | 0,250200003 |  | 0,247500002 |  | 0,239099994 |  | 0,231999993 |  | 0,244900003 |  | 0,244100004 |  | 0,246800005 |  | 0,243399993 |  | 0,244000003 |  | 0,241099998 |  | 0,237599999 |  | 0,236100003 |
| 40 | 0,260399997 0,231299996 |  | 0,253199995 |  | 0,250999987 |  | 0,241600007 |  | 0,233700007 |  | 0,247799993 |  | 0,246900007 |  | 0,249300003 |  | 0,248300001 |  | 0,2456 |  | 0,242500007 |  | 0,240400001 |  | 0,236200005 |
| 60 | 0,259499997 0,233899996 |  | 0,257299989 |  | 0,254299998 |  | 0,240400001 |  | 0,2333 |  | 0,252400011 |  | 0,249699995 |  | 0,251700014 |  | 0,252200007 |  | 0,247700006 |  | 0,244200006 |  | 0,239500001 |  | 0,236000001 |
| 80 | 0,260899991 0,233500004 |  | 0,262699991 |  | 0,261400014 |  | 0,242400005 |  | 0,236300007 |  | 0,255699992 |  | 0,254000008 |  | 0,254400015 |  | 0,254000008 |  | 0,249400005 |  | 0,246999994 |  | 0,241400003 |  | 0,236699998 |
| 100 | 0,257999986 0,234699994 |  | 0,263900012 |  | 0,260500014 |  | 0,239399999 |  | 0,235799998 |  | 0,256199986 |  | 0,256000012 |  | 0,254299998 |  | 0,255699992 |  | 0,252499998 |  | 0,248799995 |  | 0,238600001 |  | 0,236699998 |
| 120 | 0,261599988 0,233799994 |  | 0,269400001 |  | 0,263799995 |  | 0,244200006 |  | 0,238600001 |  | 0,260899991 |  | 0,2588 |  | 0,2597 |  | 0,25940001 |  | 0,255499989 |  | 0,254200011 |  | 0,240799993 |  | 0,238000005 |
| 140 | 0,259000003 0,233600006 |  | 0,273000002 |  | 0,2685 |  | 0,239899993 |  | 0,235499993 |  | 0,263399988 |  | 0,2597 |  | 0,263399988 |  | 0,260899991 |  | 0,256000012 |  | 0,253500015 |  | 0,240700006 |  | 0,237299994 |
| 160 | 0,25999999 0,2333 |  | 0,276600003 |  | 0,273900002 |  | 0,2421 |  | 0,235499993 |  | 0,265399992 |  | 0,263700008 |  | 0,265199989 |  | 0,262300014 |  | 0,258700013 |  | 0,255699992 |  | 0,239299998 |  | 0,240099996 |
| 180 | 0,256900012 0,232099995 |  | 0,281500012 |  | 0,275700003 |  | 0,239899993 |  | 0,234999999 |  | 0,268599987 |  | 0,266099989 |  | 0,264899999 |  | 0,271600008 |  | 0,259600013 |  | 0,257600009 |  | 0,241099998 |  | 0,238900006 |
| 200 | 0,25909999 0,231999993 |  | 0,283499986 |  | 0,280000001 |  | 0,239500001 |  | 0,237499997 |  | 0,271200001 |  | 0,26789999 |  | 0,266600013 |  | 0,272300005 |  | 0,259299994 |  | 0,258599997 |  | 0,242599994 |  | 0,248199999 |
| 220 | 0,260500014 0,234599993 |  | 0,288700014 |  | 0,28639999 |  | 0,242599994 |  | 0,236399993 |  | 0,2773 |  | 0,273299992 |  | 0,273299992 |  | 0,275400013 |  | 0,263900012 |  | 0,265100002 |  | 0,243300006 |  | 0,242200002 |
| 240 | 0,258899987 0,235499993 |  | 0,292600006 |  | 0,288199991 |  | 0,240899995 |  | 0,236599997 |  | 0,278899997 |  | 0,276199996 |  | 0,27700001 |  | 0,275599986 |  | 0,265700012 |  | 0,267199993 |  | 0,244299993 |  | 0,244200006 |
| 260 | 0,257600009 0,230399996 |  | 0,293500006 |  | 0,290100008 |  | 0,242300004 |  | 0,233700007 |  | 0,280299991 |  | 0,277399987 |  | 0,276199996 |  | 0,27790001 |  | 0,266600013 |  | 0,266200006 |  | 0,242799997 |  | 0,243100002 |
| 280 | 0,258700013 0,232500002 |  | 0,299800009 |  | 0,294400007 |  | 0,243399993 |  | 0,236399993 |  | 0,284200013 |  | 0,280400008 |  | 0,280400008 |  | 0,279500008 |  | 0,26879999 |  | 0,269600004 |  | 0,243599996 |  | 0,244399995 |
| 300 | 0,2579 0,230399996 |  | 0,303200006 |  | 0,296200007 |  | 0,242300004 |  | 0,237200007 |  | 0,2852 |  | 0,283399999 |  | 0,280200005 |  | 0,282299995 |  | 0,272000015 |  | 0,271600008 |  | 0,243100002 |  | 0,241899997 |
| 320 | 0,258399993 0,233400002 |  | 0,305900007 |  | 0,300399989 |  | 0,243900001 |  | 0,236100003 |  | 0,288500011 |  | 0,287900001 |  | 0,284099996 |  | 0,285100013 |  | 0,272300005 |  | 0,272300005 |  | 0,243900001 |  | 0,243599996 |
| 340 | 0,25909999 0,233199999 |  | 0,309399992 |  | 0,306600004 |  | 0,244599998 |  | 0,240199998 |  | 0,296000004 |  | 0,290300012 |  | 0,288800001 |  | 0,289299995 |  | 0,275400013 |  | 0,274899989 |  | 0,2456 |  | 0,248799995 |
| 360 | 0,261900008 0,234200001 |  | 0,316700011 |  | 0,310099989 |  | 0,244599998 |  | 0,239399999 |  | 0,296099991 |  | 0,293000013 |  | 0,290399998 |  | 0,293099999 |  | 0,278600007 |  | 0,280499995 |  | 0,246800005 |  | 0,249599993 |
| slope | -3,26107E-06 -1,57379E-06 |  | 0,00018983 |  | 0,000181104 |  | 1,09236E-05 |  | 1,29721E-05 |  | 0,000149164 |  | 0,000142683 |  | 0,000128127 |  | 0,000137054 |  | 9,76161E-05 |  | 0,000111486 |  | 2,06811E-05 |  | 3,7647E-05 |
| slope w/o |  |  | 0,000192247 |  | 0,000183522 |  | 1,33411E-05 |  | 1,53896E-05 |  | 0,000151582 |  | 0,000145101 |  | 0,000130544 |  | 0,000139471 |  | 0,000100034 |  | 0,000113903 |  | 2,30985E-05 |  | 4,00645E-05 |
| blank | |  |  | 0,000187884 | |  | | 1,43653E-05 | |  | | 0,000148341 | |  | | 0,000135008 | |  | | 0,000106969 | |  | | 3,15815E-05 | |
|  | |  |  | 0,011273065 | |  | | 0,000861919 | |  | | 0,008900464 | |  | | 0,008100463 | |  | | 0,006418111 | |  | | 0,00189489 | |
|  | |  |  | 1,682546999 | |  | | 0,128644577 | |  | | 1,328427514 | |  | | 1,209024391 | |  | | 0,957927041 | |  | | 0,282819471 | |
|  | |  |  | 12,01819285 | |  | | 0,918889833 | |  | | 9,488767956 | |  | | 8,635888505 | |  | | 6,84233601 | |  | | 2,020139078 | |
|  | | **min -1** |  | 12,01819285 | |  | | 0,918889833 | |  | | 9,488767956 | |  | | 8,635888505 | |  | | 6,84233601 | |  | | 2,020139078 | |

|  | **5 min** |  |  |  |  |  | 30 |  | | |
| --- | --- | --- | --- | --- | --- | --- | --- | --- | --- | --- |
| **no H2O2** | 12,01819285 | 100 | 100 |  |  |  | 76,05790021 |  |  |  |
| **+ H2O2** | 0,918889833 | 7,645823662 | 7,645823662 |  |  |  | 4,469147798 |  |  |  |
| **+ DTT** | 9,488767956 | 78,95336738 | 78,95336738 |  |  |  | 97,26724967 |  |  |  |
| **TrxR1+NADPH** | 8,635888505 | 71,85679753 | 71,85679753 |  |  |  | 31,22145889 |  |  |  |
| **TrxR1+Trx1+NADPH** | 6,84233601 | 56,93315205 | 56,93315205 |  |  |  |  |  |  |  |
| **TrxR1+Trx1** | 2,020139078 | 16,80900866 | 16,80900866 |  |  |  |  |  |  |  |
|  | #DIV/0! |  |  |  |  |  |  |  |  |  |
|  | #DIV/0! |  |  | **no H2O2** | **+ H2O2** | **+ DTT** |  | **TrxR1+NADPH** | **TrxR1+Trx1+NADPH** | **TrxR1+Trx1** |

| Application: Tecan i-control  Device: infinite 200Pro | | Tecan i-control , 2.0.10.0  Serial number: 1307001123 | Serial number of connected stacker: |
| --- | --- | --- | --- |
| Firmware: V_3.40_01/15_Infinite (Dec 23 201 MAI, V_3.40_01/15_Infinite (Dec 23 2014/12.45.11) | | | |
| Date: | ######### | | |
| Time: | 14:54:08 | | |

System MTC-MU059-S

User MTC-MU059-S\fretho

Plate Greiner 96 Flat Bottom Transparent Polystyrene Cat. No.: 655101/655161/655192 [GRE96ft.pdfx] Plate-ID (Stacker)

List of actions in this measurement script: Kinetic

Absorbance

Shaking (Linear) Duration: Shaking (Linear) Amplitude:

3 s

1 mm

Label: Label1

Kinetic Measurement

Kinetic duration 00:06:00

Interval Time 00:00:20

Measurement Wavelength 405 nm

Bandwidth 10 nm

Number of Flashes 5

Settle Time 0 ms

Part of Plate F1-G12; H1-H2

Start Time: 2020 08-13 14:54:10

| Cycle Nr. | 1 | 2 | 3 | 4 | 5 | 6 | 7 | 8 | 9 | 10 | 11 | 12 | 13 | 14 | 15 | 16 | 17 | 18 | 19 |
| --- | --- | --- | --- | --- | --- | --- | --- | --- | --- | --- | --- | --- | --- | --- | --- | --- | --- | --- | --- |
| Time [s] | 0 | 20 | 40 | 60 | 80 | 100 | 120 | 140 | 160 | 180 | 200 | 220 | 240 | 260 | 280 | 300 | 320 | 340 | 360 |
| Temp. [°C] | 25,4 | 25,6 | 25,7 | 25,7 | 25,7 | 25,3 | 25,4 | 25,5 | 25,6 | 25,7 | 25,5 | 25,5 | 25,7 | 25,8 | 25,6 | 25,8 | 25,8 | 25,8 | 25,7 |
| F1 | 0,2527 | 0,2502 | 0,2532 | 0,2573 | 0,2627 | 0,2639 | 0,2694 | 0,273 | 0,2766 | 0,2815 | 0,2835 | 0,2887 | 0,2926 | 0,2935 | 0,2998 | 0,3032 | 0,3059 | 0,3094 | 0,3167 |
| F2 | 0,2489 | 0,2475 | 0,251 | 0,2543 | 0,2614 | 0,2605 | 0,2638 | 0,2685 | 0,2739 | 0,2757 | 0,28 | 0,2864 | 0,2882 | 0,2901 | 0,2944 | 0,2962 | 0,3004 | 0,3066 | 0,3101 |
| F3 | 0,2443 | 0,2391 | 0,2416 | 0,2404 | 0,2424 | 0,2394 | 0,2442 | 0,2399 | 0,2421 | 0,2399 | 0,2395 | 0,2426 | 0,2409 | 0,2423 | 0,2434 | 0,2423 | 0,2439 | 0,2446 | 0,2446 |
| F4 | 0,2399 | 0,232 | 0,2337 | 0,2333 | 0,2363 | 0,2358 | 0,2386 | 0,2355 | 0,2355 | 0,235 | 0,2375 | 0,2364 | 0,2366 | 0,2337 | 0,2364 | 0,2372 | 0,2361 | 0,2402 | 0,2394 |
| F5 | 0,2457 | 0,2449 | 0,2478 | 0,2524 | 0,2557 | 0,2562 | 0,2609 | 0,2634 | 0,2654 | 0,2686 | 0,2712 | 0,2773 | 0,2789 | 0,2803 | 0,2842 | 0,2852 | 0,2885 | 0,296 | 0,2961 |
| F6 | 0,2473 | 0,2441 | 0,2469 | 0,2497 | 0,254 | 0,256 | 0,2588 | 0,2597 | 0,2637 | 0,2661 | 0,2679 | 0,2733 | 0,2762 | 0,2774 | 0,2804 | 0,2834 | 0,2879 | 0,2903 | 0,293 |
| F7 | 0,2474 | 0,2468 | 0,2493 | 0,2517 | 0,2544 | 0,2543 | 0,2597 | 0,2634 | 0,2652 | 0,2649 | 0,2666 | 0,2733 | 0,277 | 0,2762 | 0,2804 | 0,2802 | 0,2841 | 0,2888 | 0,2904 |
| F8 | 0,2444 | 0,2434 | 0,2483 | 0,2522 | 0,254 | 0,2557 | 0,2594 | 0,2609 | 0,2623 | 0,2716 | 0,2723 | 0,2754 | 0,2756 | 0,2779 | 0,2795 | 0,2823 | 0,2851 | 0,2893 | 0,2931 |
| F9 | 0,2454 | 0,244 | 0,2456 | 0,2477 | 0,2494 | 0,2525 | 0,2555 | 0,256 | 0,2587 | 0,2596 | 0,2593 | 0,2639 | 0,2657 | 0,2666 | 0,2688 | 0,272 | 0,2723 | 0,2754 | 0,2786 |
| F10 | 0,2436 | 0,2411 | 0,2425 | 0,2442 | 0,247 | 0,2488 | 0,2542 | 0,2535 | 0,2557 | 0,2576 | 0,2586 | 0,2651 | 0,2672 | 0,2662 | 0,2696 | 0,2716 | 0,2723 | 0,2749 | 0,2805 |
| F11 | 0,2385 | 0,2376 | 0,2404 | 0,2395 | 0,2414 | 0,2386 | 0,2408 | 0,2407 | 0,2393 | 0,2411 | 0,2426 | 0,2433 | 0,2443 | 0,2428 | 0,2436 | 0,2431 | 0,2439 | 0,2456 | 0,2468 |
| F12 | 0,235 | 0,2361 | 0,2362 | 0,236 | 0,2367 | 0,2367 | 0,238 | 0,2373 | 0,2401 | 0,2389 | 0,2482 | 0,2422 | 0,2442 | 0,2431 | 0,2444 | 0,2419 | 0,2436 | 0,2488 | 0,2496 |
| G1 | 0,3557 | 0,4256 | 0,4869 | 0,5476 | 0,6131 | 0,673 | 0,7346 | 0,7953 | 0,8566 | 0,9182 | 0,9796 | 1,0427 | 1,1035 | 1,1598 | 1,2231 | 1,2841 | 1,346 | 1,406 | 1,469 |
| G2 | 0,3604 | 0,4231 | 0,4837 | 0,5464 | 0,6108 | 0,6713 | 0,7335 | 0,7951 | 0,8554 | 0,9152 | 0,9758 | 1,0383 | 1,0992 | 1,1575 | 1,221 | 1,28 | 1,3426 | 1,4021 | 1,463 |
| G3 | 0,2386 | 0,2396 | 0,2352 | 0,2328 | 0,2354 | 0,2324 | 0,2353 | 0,2343 | 0,2361 | 0,2341 | 0,2332 | 0,2345 | 0,2358 | 0,2327 | 0,2337 | 0,2319 | 0,2337 | 0,2334 | 0,2347 |
| G4 | 0,2367 | 0,2347 | 0,2331 | 0,2328 | 0,2353 | 0,2332 | 0,2354 | 0,2334 | 0,2336 | 0,2364 | 0,234 | 0,2373 | 0,2347 | 0,2313 | 0,2331 | 0,2334 | 0,234 | 0,2347 | 0,235 |
| G5 | 0,3085 | 0,3464 | 0,3823 | 0,4211 | 0,4627 | 0,4958 | 0,5407 | 0,573 | 0,6112 | 0,6546 | 0,6868 | 0,7258 | 0,7622 | 0,7951 | 0,8371 | 0,871 | 0,9079 | 0,9476 | 0,9841 |
| G6 | 0,3183 | 0,3447 | 0,3848 | 0,4206 | 0,4584 | 0,4962 | 0,5334 | 0,5671 | 0,6058 | 0,642 | 0,6768 | 0,7158 | 0,7521 | 0,7876 | 0,8259 | 0,8635 | 0,902 | 0,9399 | 0,9751 |
| G7 | 0,2439 | 0,2463 | 0,2481 | 0,2504 | 0,2541 | 0,2574 | 0,2639 | 0,2657 | 0,2698 | 0,2719 | 0,2768 | 0,2787 | 0,2819 | 0,2819 | 0,2842 | 0,2898 | 0,2921 | 0,2973 | 0,2999 |
| G8 | 0,2457 | 0,2444 | 0,248 | 0,2509 | 0,2543 | 0,2573 | 0,2608 | 0,2649 | 0,2666 | 0,27 | 0,2713 | 0,278 | 0,2798 | 0,2792 | 0,2833 | 0,2874 | 0,2935 | 0,2964 | 0,3002 |
| G9 | 0,2981 | 0,3142 | 0,34 | 0,3674 | 0,3969 | 0,4218 | 0,451 | 0,4783 | 0,5063 | 0,5318 | 0,558 | 0,5883 | 0,6168 | 0,6402 | 0,6703 | 0,6969 | 0,7207 | 0,752 | 0,7787 |
| G10 | 0,2981 | 0,3143 | 0,3413 | 0,368 | 0,3993 | 0,4243 | 0,4558 | 0,4813 | 0,509 | 0,5386 | 0,5629 | 0,5942 | 0,6207 | 0,6457 | 0,6753 | 0,7018 | 0,7318 | 0,7595 | 0,7896 |
| G11 | 0,2397 | 0,2381 | 0,2391 | 0,2385 | 0,2412 | 0,2384 | 0,2411 | 0,241 | 0,2415 | 0,2428 | 0,243 | 0,2453 | 0,2449 | 0,2419 | 0,247 | 0,245 | 0,2478 | 0,2487 | 0,2504 |
| G12 | 0,2387 | 0,2364 | 0,2368 | 0,2382 | 0,2412 | 0,2402 | 0,2417 | 0,2418 | 0,2423 | 0,2403 | 0,2418 | 0,2442 | 0,2443 | 0,2432 | 0,2449 | 0,2432 | 0,2481 | 0,2448 | 0,2489 |
| H1 | 0,2586 | 0,2606 | 0,2604 | 0,2595 | 0,2609 | 0,258 | 0,2616 | 0,259 | 0,26 | 0,2569 | 0,2591 | 0,2605 | 0,2589 | 0,2576 | 0,2587 | 0,2579 | 0,2584 | 0,2591 | 0,2619 |
| H2 | 0,2373 | 0,2335 | 0,2313 | 0,2339 | 0,2335 | 0,2347 | 0,2338 | 0,2336 | 0,2333 | 0,2321 | 0,232 | 0,2346 | 0,2355 | 0,2304 | 0,2325 | 0,2304 | 0,2334 | 0,2332 | 0,2342 |
