# Supplementary material for: Redox regulation of PTPN22 affects the severity of T-cell-dependent autoimmune inflammation
Source: eLife. 2022 May 19;11:e74549. doi: 10.7554/eLife.74549 (PMC9119677; doi:10.7554/eLife.74549)
Supplement: Figure 2—source data 2. [file elife-74549-fig2-data2.docx]

| **Time (sec) Blank** | **Blank** | **no H2O2** | **no H2O2** | **+ H2O2** |  | **+ H2O2** | **+ DTT** |  | **+ DTT** | **+NADPH** | **+NADPH** |  | **NADPH+0.01 μM TrxR1** | **NADPH+0.01 μM TrxR1** | **NADPH+0.1 μM TrxR1** |  | **NADPH+0.1 μM TrxR1** | **NADPH+1 μM TrxR1** | **NADPH+1 μM TrxR1** |  | **NADPH+2 μM TrxR1** | **NADPH+2 μM TrxR1** |  |
| --- | --- | --- | --- | --- | --- | --- | --- | --- | --- | --- | --- | --- | --- | --- | --- | --- | --- | --- | --- | --- | --- | --- | --- |
| 20 | 0,233400002 0,234300002 | 0,260500014 |  | 0,263300002 | 0,255899996 |  | 0,254400015 | 0,254999995 | 0,255600005 |  | 0,243000001 | 0,238299996 | 0,238900006 |  | 0,235699996 | 0,237599999 | 0,234699994 |  | 0,247899994 | 0,248099998 | 0,243900001 |  | 0,243000001 |
| 40 | 0,234699994 0,233199999 | 0,25999999 |  | 0,263200015 | 0,255299985 |  | 0,254200011 | 0,256300002 | 0,257699996 |  | 0,240799993 | 0,238999993 | 0,239600003 |  | 0,235400006 | 0,2377 | 0,235799998 |  | 0,25029999 | 0,248799995 | 0,245499998 |  | 0,243300006 |
| 60 | 0,234500006 0,232600003 | 0,264800012 |  | 0,265500009 | 0,254099995 |  | 0,253100008 | 0,259000003 | 0,259299994 |  | 0,242200002 | 0,239700004 | 0,239500001 |  | 0,235599995 | 0,237499997 | 0,235100001 |  | 0,252499998 | 0,250200003 | 0,246900007 |  | 0,244599998 |
| 80 | 0,233999997 0,231900007 | 0,2667 |  | 0,26699999 | 0,253699988 |  | 0,252799988 | 0,261000007 | 0,261999995 |  | 0,242300004 | 0,238000005 | 0,238800004 |  | 0,236399993 | 0,2377 | 0,236100003 |  | 0,253399998 | 0,249500006 | 0,247299999 |  | 0,244800001 |
| 100 | 0,234699994 0,231700003 | 0,2685 |  | 0,269899994 | 0,253300011 |  | 0,252900004 | 0,261900008 | 0,264299989 |  | 0,241300002 | 0,238999993 | 0,239999995 |  | 0,236000001 | 0,236300007 | 0,235799998 |  | 0,255400002 | 0,251800001 | 0,248799995 |  | 0,247099996 |
| 120 | 0,233400002 0,231399998 | 0,271100014 |  | 0,270900011 | 0,253199995 |  | 0,253199995 | 0,264999986 | 0,265899986 |  | 0,239999995 | 0,239099994 | 0,239399999 |  | 0,236399993 | 0,238399997 | 0,236100003 |  | 0,257099986 | 0,252299994 | 0,249899998 |  | 0,247099996 |
| 140 | 0,232999995 0,230700001 | 0,272700012 |  | 0,273099989 | 0,252400011 |  | 0,252299994 | 0,266900003 | 0,268299997 |  | 0,240899995 | 0,237499997 | 0,239600003 |  | 0,236300007 | 0,239199996 | 0,236599997 |  | 0,257600009 | 0,254400015 | 0,251700014 |  | 0,2465 |
| 160 | 0,234200001 0,230299994 | 0,273799986 |  | 0,2764 | 0,253300011 |  | 0,253500015 | 0,269400001 | 0,270900011 |  | 0,240099996 | 0,237800002 | 0,239600003 |  | 0,2359 | 0,239399999 | 0,236599997 |  | 0,25940001 | 0,254599988 | 0,25150001 |  | 0,249899998 |
| 180 | 0,234599993 0,230499998 | 0,275299996 |  | 0,276600003 | 0,252200007 |  | 0,253699988 | 0,272100002 | 0,271899998 |  | 0,241099998 | 0,238399997 | 0,240199998 |  | 0,236100003 | 0,239800006 | 0,237200007 |  | 0,261000007 | 0,256700009 | 0,254400015 |  | 0,251300007 |
| 200 | 0,233899996 0,230399996 | 0,278100014 |  | 0,279100001 | 0,252000004 |  | 0,253199995 | 0,275099993 | 0,273900002 |  | 0,240700006 | 0,237299994 | 0,240400001 |  | 0,236499995 | 0,239999995 | 0,236599997 |  | 0,262699991 | 0,256799996 | 0,255400002 |  | 0,252400011 |
| 220 | 0,2315 0,230399996 | 0,280499995 |  | 0,280699998 | 0,251899987 |  | 0,252299994 | 0,276300013 | 0,276600003 |  | 0,240400001 | 0,237100005 | 0,240500003 |  | 0,236599997 | 0,241400003 | 0,238600001 |  | 0,264200002 | 0,25940001 | 0,256700009 |  | 0,254299998 |
| 240 | 0,231399998 0,229000002 | 0,282700002 |  | 0,282799989 | 0,250999987 |  | 0,252299994 | 0,279799998 | 0,279599994 |  | 0,239500001 | 0,236699998 | 0,240500003 |  | 0,236699998 | 0,241699994 | 0,238900006 |  | 0,265799999 | 0,260100007 | 0,258899987 |  | 0,255199999 |
| 260 | 0,230900005 0,230299994 | 0,285299987 |  | 0,28490001 | 0,251100004 |  | 0,253399998 | 0,282200009 | 0,282000005 |  | 0,239999995 | 0,237599999 | 0,241600007 |  | 0,237200007 | 0,241999999 | 0,239700004 |  | 0,268099993 | 0,261099994 | 0,260699987 |  | 0,256399989 |
| 280 | 0,229599997 0,230000004 | 0,287400007 |  | 0,287299991 | 0,2509 |  | 0,252999991 | 0,28459999 | 0,284700006 |  | 0,240099996 | 0,238299996 | 0,241999999 |  | 0,238199994 | 0,243000001 | 0,238399997 |  | 0,268400013 | 0,263799995 | 0,261799991 |  | 0,257999986 |
| 300 | 0,234300002 0,229399994 | 0,288899988 |  | 0,289000005 | 0,25150001 |  | 0,251899987 | 0,286199987 | 0,286199987 |  | 0,239700004 | 0,237900004 | 0,241400003 |  | 0,236599997 | 0,244800001 | 0,239800006 |  | 0,270300001 | 0,264800012 | 0,263000011 |  | 0,2588 |
| 320 | 0,231800005 0,231299996 | 0,291599989 |  | 0,291900009 | 0,252200007 |  | 0,252999991 | 0,289499998 | 0,289200008 |  | 0,240999997 | 0,237900004 | 0,242200002 |  | 0,237800002 | 0,243599996 | 0,240899995 |  | 0,272199988 | 0,266499996 | 0,265199989 |  | 0,261299998 |
| 340 | 0,231299996 0,230599999 | 0,292800009 |  | 0,293500006 | 0,250499994 |  | 0,252499998 | 0,291299999 | 0,291399986 |  | 0,238999993 | 0,237399995 | 0,242400005 |  | 0,238900006 | 0,247099996 | 0,241899997 |  | 0,273499995 | 0,268099993 | 0,266000003 |  | 0,262499988 |
| 360 | 0,231900007 0,230299994 | 0,296900004 |  | 0,296999991 | 0,251599997 |  | 0,253300011 | 0,294 | 0,293099999 |  | 0,240199998 | 0,2377 | 0,242500007 |  | 0,238299996 | 0,246399999 | 0,242300004 |  | 0,276300013 | 0,269800007 | 0,26699999 |  | 0,264400005 |
| slope | -9,72651E-06 -9,42726E-06 | 0,000105562 |  | 9,9644E-05 | -1,24716E-05 |  | -2,95153E-06 | 0,000117368 | 0,000111961 |  | -7,02271E-06 | -4,26728E-06 | 1,07172E-05 |  | 8,12178E-06 | 2,84933E-05 | 2,07585E-05 |  | 7,86326E-05 | 6,45614E-05 | 6,9969E-05 |  | 6,41847E-05 |
| slope w/o blank |  | 0,000115139 |  | 0,000109221 | -2,89472E-06 |  | 6,62536E-06 | 0,000126945 | 0,000121538 |  | 2,55418E-06 | 5,30961E-06 | 2,02941E-05 |  | 1,76987E-05 | 3,80702E-05 | 3,03354E-05 |  | 8,82095E-05 | 7,41383E-05 | 7,95459E-05 |  | 7,37616E-05 |
|  |  |  |  | 0,00011218 |  |  | 1,86532E-06 |  | 0,000124241 |  |  | 3,93189E-06 |  |  | 1,89964E-05 |  | 3,42028E-05 |  |  | 8,11739E-05 |  |  | 7,66537E-05 |

0,006730804 0,000111919 0,007454489 0,000235914 0,001139784 0,002052167 0,004870434 0,004599225

1,004597642 0,01670437 1,112610321 0,035210991 0,170117002 0,306293651 0,726930505 0,686451488

7,175697444 0,119316926 7,947216581 0,251507077 1,215121444 2,18781179 5,192360754 4,903224917

**min -1** 7,175697444 0,119316926 7,947216581 0,251507077 1,215121444 2,18781179 5,192360754 4,903224917

|  | **5 min** |  |  |  |  | 30 |  | | | | | |
| --- | --- | --- | --- | --- | --- | --- | --- | --- | --- | --- | --- | --- |
| **no H2O2** | 7,175697444 | 100 | 100 | 7,175697444 |  | 76,05790021 |  |  |  |  |  |  |
| **+ H2O2** | 0,119316926 | 1,662792048 | 1,662792048 | 0,119316926 |  | 4,469147798 |  |  |  |  |  |  |
| **+ DTT** | 7,947216581 | 110,7518348 | 110,7518348 | 7,947216581 |  | 97,26724967 |  |  |  |  |  |  |
| **+NADPH** | 0,251507077 | 3,504984419 | 3,504984419 | 0,251507077 |  | 31,22145889 |  |  |  |  |  |  |
| **NADPH+0.01 μM TrxR1** | 1,215121444 | 16,93384446 | 16,93384446 | 1,215121444 |  |  |  |  |  |  |  |  |
| **NADPH+0.1 μM TrxR1** | 2,18781179 | 30,48918669 | 30,48918669 | 2,18781179 |  |  |  |  |  |  |  |  |
| **NADPH+1 μM TrxR1** | 5,192360754 | 72,36036349 | 72,36036349 | 5,192360754 |  |  |  |  |  |  |  |  |
| **NADPH+2 μM TrxR1** | 4,903224917 | 68,33098741 | 68,33098741 | **4,903224917 + H2O2** | **+ DTT** |  | **+NADPH** | **NADPH+0.01 μM TrxR1** | **NADPH+0.1 μM TrxR1** | **NADPH+1 μM TrxR1** | | |
| **no H2O2 + H2O2** | | | | | |  | **+ DTT** | **+NADPH** | **NADPH+0.01 μM TrxR1** | **NADPH+0.1 μM TrxR1** | **NADPH+1 μM TrxR1** | **NADPH+2 μM TrxR1** |

Application: Tecan i-control Tecan i-control , 2.0.10.0

Device: infinite 200Pro Serial number: 1307001124 Serial number of connected stacker: Firmware: V_3.37_07/12_Infinite (Jul 20 2012 MAI, V_3.37_07/12_Infinite (Jul 20 2012/13.56.47)

Date: #########

Time: 17:31:13

System MTC-MU063-S

User MTC-MU063-S\TECAN

Plate Greiner 96 Flat Bottom Transparent Polystyrene Cat. No.: 655101/655161/655192 [GRE96ft.pdfx] Plate-ID (Stacker)

List of actions in this measurement script: Kinetic

Absorbance

Label: Label1

Kinetic Measurement

Kinetic duration 00:06:00

Interval Time 00:00:20

Measurement Wavelength 405 nm

Bandwidth 9 nm

Number of Flashes 5

Settle Time 0 ms

Part of Plate A1-A12; B1-B6

Start Time: 2019 12-08 17:31:14

| Cycle Nr. | 1 | 2 | 3 | 4 | 5 | 6 | 7 | 8 | 9 | 10 | 11 | 12 | 13 | 14 | 15 | 16 | 17 | 18 | 19 |
| --- | --- | --- | --- | --- | --- | --- | --- | --- | --- | --- | --- | --- | --- | --- | --- | --- | --- | --- | --- |
| Time [s] | 0 | 20 | 40 | 60 | 80 | 100 | 120 | 140 | 160 | 180 | 200 | 220 | 240 | 260 | 280 | 300 | 320 | 340 | 360 |
| Temp. [°C] | 23,5 | 23,7 | 23,5 | 23,4 | 23,8 | 23,9 | 23,8 | 23,9 | 23,6 | 24,1 | 23,7 | 24,1 | 23,8 | 23,8 | 24 | 24 | 24 | 23,9 | 23,8 |
| A1 | 0,2592 | 0,2605 | 0,26 | 0,2648 | 0,2667 | 0,2685 | 0,2711 | 0,2727 | 0,2738 | 0,2753 | 0,2781 | 0,2805 | 0,2827 | 0,2853 | 0,2874 | 0,2889 | 0,2916 | 0,2928 | 0,2969 |
| A2 | 0,262 | 0,2633 | 0,2632 | 0,2655 | 0,267 | 0,2699 | 0,2709 | 0,2731 | 0,2764 | 0,2766 | 0,2791 | 0,2807 | 0,2828 | 0,2849 | 0,2873 | 0,289 | 0,2919 | 0,2935 | 0,297 |
| A3 | 0,2563 | 0,2559 | 0,2553 | 0,2541 | 0,2537 | 0,2533 | 0,2532 | 0,2524 | 0,2533 | 0,2522 | 0,252 | 0,2519 | 0,251 | 0,2511 | 0,2509 | 0,2515 | 0,2522 | 0,2505 | 0,2516 |
| A4 | 0,2541 | 0,2544 | 0,2542 | 0,2531 | 0,2528 | 0,2529 | 0,2532 | 0,2523 | 0,2535 | 0,2537 | 0,2532 | 0,2523 | 0,2523 | 0,2534 | 0,253 | 0,2519 | 0,253 | 0,2525 | 0,2533 |
| A5 | 0,2541 | 0,255 | 0,2563 | 0,259 | 0,261 | 0,2619 | 0,265 | 0,2669 | 0,2694 | 0,2721 | 0,2751 | 0,2763 | 0,2798 | 0,2822 | 0,2846 | 0,2862 | 0,2895 | 0,2913 | 0,294 |
| A6 | 0,2552 | 0,2556 | 0,2577 | 0,2593 | 0,262 | 0,2643 | 0,2659 | 0,2683 | 0,2709 | 0,2719 | 0,2739 | 0,2766 | 0,2796 | 0,282 | 0,2847 | 0,2862 | 0,2892 | 0,2914 | 0,2931 |
| A7 | 0,2433 | 0,243 | 0,2408 | 0,2422 | 0,2423 | 0,2413 | 0,24 | 0,2409 | 0,2401 | 0,2411 | 0,2407 | 0,2404 | 0,2395 | 0,24 | 0,2401 | 0,2397 | 0,241 | 0,239 | 0,2402 |
| A8 | 0,2403 | 0,2383 | 0,239 | 0,2397 | 0,238 | 0,239 | 0,2391 | 0,2375 | 0,2378 | 0,2384 | 0,2373 | 0,2371 | 0,2367 | 0,2376 | 0,2383 | 0,2379 | 0,2379 | 0,2374 | 0,2377 |
| A9 | 0,2396 | 0,2389 | 0,2396 | 0,2395 | 0,2388 | 0,24 | 0,2394 | 0,2396 | 0,2396 | 0,2402 | 0,2404 | 0,2405 | 0,2405 | 0,2416 | 0,242 | 0,2414 | 0,2422 | 0,2424 | 0,2425 |
| A10 | 0,2373 | 0,2357 | 0,2354 | 0,2356 | 0,2364 | 0,236 | 0,2364 | 0,2363 | 0,2359 | 0,2361 | 0,2365 | 0,2366 | 0,2367 | 0,2372 | 0,2382 | 0,2366 | 0,2378 | 0,2389 | 0,2383 |
| A11 | 0,2368 | 0,2376 | 0,2377 | 0,2375 | 0,2377 | 0,2363 | 0,2384 | 0,2392 | 0,2394 | 0,2398 | 0,24 | 0,2414 | 0,2417 | 0,242 | 0,243 | 0,2448 | 0,2436 | 0,2471 | 0,2464 |
| A12 | 0,2358 | 0,2347 | 0,2358 | 0,2351 | 0,2361 | 0,2358 | 0,2361 | 0,2366 | 0,2366 | 0,2372 | 0,2366 | 0,2386 | 0,2389 | 0,2397 | 0,2384 | 0,2398 | 0,2409 | 0,2419 | 0,2423 |
| B1 | 0,2484 | 0,2479 | 0,2503 | 0,2525 | 0,2534 | 0,2554 | 0,2571 | 0,2576 | 0,2594 | 0,261 | 0,2627 | 0,2642 | 0,2658 | 0,2681 | 0,2684 | 0,2703 | 0,2722 | 0,2735 | 0,2763 |
| B2 | 0,2464 | 0,2481 | 0,2488 | 0,2502 | 0,2495 | 0,2518 | 0,2523 | 0,2544 | 0,2546 | 0,2567 | 0,2568 | 0,2594 | 0,2601 | 0,2611 | 0,2638 | 0,2648 | 0,2665 | 0,2681 | 0,2698 |
| B3 | 0,2439 | 0,2439 | 0,2455 | 0,2469 | 0,2473 | 0,2488 | 0,2499 | 0,2517 | 0,2515 | 0,2544 | 0,2554 | 0,2567 | 0,2589 | 0,2607 | 0,2618 | 0,263 | 0,2652 | 0,266 | 0,267 |
| B4 | 0,2423 | 0,243 | 0,2433 | 0,2446 | 0,2448 | 0,2471 | 0,2471 | 0,2465 | 0,2499 | 0,2513 | 0,2524 | 0,2543 | 0,2552 | 0,2564 | 0,258 | 0,2588 | 0,2613 | 0,2625 | 0,2644 |
| B5 | 0,2352 | 0,2334 | 0,2347 | 0,2345 | 0,234 | 0,2347 | 0,2334 | 0,233 | 0,2342 | 0,2346 | 0,2339 | 0,2315 | 0,2314 | 0,2309 | 0,2296 | 0,2343 | 0,2318 | 0,2313 | 0,2319 |
| B6 | 0,2352 | 0,2343 | 0,2332 | 0,2326 | 0,2319 | 0,2317 | 0,2314 | 0,2307 | 0,2303 | 0,2305 | 0,2304 | 0,2304 | 0,229 | 0,2303 | 0,23 | 0,2294 | 0,2313 | 0,2306 | 0,2303 |

End Time: 2019 12-08 17:37:24

| **Time (sec) Blank** | **Blank** | **no H2O2** | **no H2O2** | **+ H2O2** |  | **+ H2O2** | **+ DTT** |  | **+ DTT** | **+NADPH** | **+NADPH** |  | **NADPH+0.01 μM TrxR1** | **NADPH+0.01 μM TrxR1** | **NADPH+0.1 μM TrxR1** |  | **NADPH+0.1 μM TrxR1** | **NADPH+1 μM TrxR1** | **NADPH+1 μM TrxR1** |  | **NADPH+2 μM TrxR1** | **NADPH+2 μM TrxR1** |  |
| --- | --- | --- | --- | --- | --- | --- | --- | --- | --- | --- | --- | --- | --- | --- | --- | --- | --- | --- | --- | --- | --- | --- | --- |
| 20 | 0,245000005 0,247799993 | 0,252499998 |  | 0,248699993 | 0,239700004 |  | 0,237599999 | 0,243200004 | 0,244299993 |  | 0,243900001 | 0,246199995 | 0,2447 |  | 0,252099991 | 0,252299994 | 0,250099987 |  | 0,242400005 | 0,2447 | 0,247400001 |  | 0,251800001 |
| 40 | 0,244800001 0,248799995 | 0,255299985 |  | 0,250200003 | 0,238700002 |  | 0,238100007 | 0,2456 | 0,246900007 |  | 0,243399993 | 0,246199995 | 0,246299997 |  | 0,250699997 | 0,252900004 | 0,252099991 |  | 0,245700002 | 0,247999996 | 0,249699995 |  | 0,253300011 |
| 60 | 0,245199993 0,248099998 | 0,256399989 |  | 0,252600014 | 0,237299994 |  | 0,237599999 | 0,246600002 | 0,248699993 |  | 0,242500007 | 0,243900001 | 0,246700004 |  | 0,25060001 | 0,253800005 | 0,251700014 |  | 0,246900007 | 0,249300003 | 0,251100004 |  | 0,254900008 |
| 80 | 0,244599998 0,247799993 | 0,259799987 |  | 0,253800005 | 0,237599999 |  | 0,237200007 | 0,247299999 | 0,248899996 |  | 0,242300004 | 0,245199993 | 0,245900005 |  | 0,250400007 | 0,253399998 | 0,25150001 |  | 0,249300003 | 0,249799997 | 0,253199995 |  | 0,256999999 |
| 100 | 0,244499996 0,248099998 | 0,260800004 |  | 0,256399989 | 0,238600001 |  | 0,238600001 | 0,249799997 | 0,252299994 |  | 0,243499994 | 0,245100006 | 0,247299999 |  | 0,251899987 | 0,254900008 | 0,254000008 |  | 0,251100004 | 0,251700014 | 0,254000008 |  | 0,259900004 |
| 120 | 0,243100002 0,246399999 | 0,264099985 |  | 0,258700013 | 0,238700002 |  | 0,238199994 | 0,252000004 | 0,254000008 |  | 0,242699996 | 0,245499998 | 0,246299997 |  | 0,252099991 | 0,254500002 | 0,255199999 |  | 0,254999995 | 0,253300011 | 0,256500006 |  | 0,262300014 |
| 140 | 0,242500007 0,247400001 | 0,264299989 |  | 0,261099994 | 0,237499997 |  | 0,236599997 | 0,253600001 | 0,255899996 |  | 0,2421 | 0,243499994 | 0,247899994 |  | 0,252299994 | 0,256000012 | 0,255899996 |  | 0,256099999 | 0,254500002 | 0,258899987 |  | 0,262400001 |
| 160 | 0,242899999 0,246800005 | 0,26789999 |  | 0,261599988 | 0,238499999 |  | 0,236100003 | 0,255100012 | 0,255299985 |  | 0,243000001 | 0,243799999 | 0,247799993 |  | 0,252099991 | 0,256999999 | 0,255699992 |  | 0,2588 | 0,256900012 | 0,260100007 |  | 0,261599988 |
| 180 | 0,242799997 0,247700006 | 0,269199997 |  | 0,263799995 | 0,238100007 |  | 0,236300007 | 0,255600005 | 0,258300006 |  | 0,241400003 | 0,244399995 | 0,248899996 |  | 0,252400011 | 0,256799996 | 0,256300002 |  | 0,259499997 | 0,259299994 | 0,262899995 |  | 0,265199989 |
| 200 | 0,2421 0,247400001 | 0,273400009 |  | 0,267500013 | 0,238600001 |  | 0,238299996 | 0,25940001 | 0,262199998 |  | 0,243900001 | 0,243599996 | 0,248699993 |  | 0,251899987 | 0,25850001 | 0,257400006 |  | 0,262699991 | 0,261400014 | 0,264999986 |  | 0,26879999 |
| 220 | 0,241600007 0,248099998 | 0,274100006 |  | 0,26879999 | 0,237800002 |  | 0,2377 | 0,260600001 | 0,262899995 |  | 0,242500007 | 0,244299993 | 0,249400005 |  | 0,250999987 | 0,25940001 | 0,25850001 |  | 0,264400005 | 0,263200015 | 0,266200006 |  | 0,270900011 |
| 240 | 0,243699998 0,247099996 | 0,2764 |  | 0,269699991 | 0,237299994 |  | 0,236900002 | 0,263300002 | 0,264800012 |  | 0,241799995 | 0,244900003 | 0,248600006 |  | 0,253199995 | 0,261599988 | 0,259900004 |  | 0,266400009 | 0,265100002 | 0,270000011 |  | 0,273699999 |
| 260 | 0,243599996 0,248799995 | 0,279000014 |  | 0,274399996 | 0,2368 |  | 0,2368 | 0,265300006 | 0,266299993 |  | 0,244599998 | 0,244100004 | 0,248600006 |  | 0,253699988 | 0,261000007 | 0,261999995 |  | 0,270200014 | 0,265199989 | 0,271499991 |  | 0,276199996 |
| 280 | 0,243599996 0,247400001 | 0,281800002 |  | 0,274800003 | 0,237100005 |  | 0,237499997 | 0,26820001 | 0,26820001 |  | 0,243399993 | 0,245900005 | 0,249599993 |  | 0,253699988 | 0,264400005 | 0,261900008 |  | 0,272399992 | 0,269199997 | 0,272399992 |  | 0,278100014 |
| 300 | 0,242699996 0,248400003 | 0,283699989 |  | 0,278800011 | 0,238000005 |  | 0,236100003 | 0,269800007 | 0,270599991 |  | 0,243599996 | 0,244900003 | 0,2509 |  | 0,255100012 | 0,264299989 | 0,263099998 |  | 0,274399996 | 0,271200001 | 0,27669999 |  | 0,280299991 |
| 320 | 0,243100002 0,248400003 | 0,286199987 |  | 0,280400008 | 0,238499999 |  | 0,2368 | 0,272500008 | 0,272599995 |  | 0,243799999 | 0,2447 | 0,249599993 |  | 0,254099995 | 0,265799999 | 0,264800012 |  | 0,276800007 | 0,273699999 | 0,278600007 |  | 0,281699985 |
| 340 | 0,244100004 0,248699993 | 0,287200004 |  | 0,282099992 | 0,238199994 |  | 0,238000005 | 0,274899989 | 0,275299996 |  | 0,244800001 | 0,245299995 | 0,251199991 |  | 0,255199999 | 0,266000003 | 0,265399992 |  | 0,27759999 | 0,274100006 | 0,281800002 |  | 0,282299995 |
| 360 | 0,244100004 0,248400003 | 0,290300012 |  | 0,28459999 | 0,238700002 |  | 0,238299996 | 0,276899993 | 0,27759999 |  | 0,244800001 | 0,246800005 | 0,252400011 |  | 0,256599993 | 0,267500013 | 0,267500013 |  | 0,280400008 | 0,276300013 | 0,281599998 |  | 0,287200004 |
| slope | -3,98348E-06 1,64088E-06 | 0,000110836 |  | 0,00010676 | -1,74923E-06 |  | -1,21259E-06 | 9,92828E-05 | 9,50413E-05 |  | 4,097E-06 | 4,59253E-07 | 1,76471E-05 |  | 1,38958E-05 | 4,63726E-05 | 4,85759E-05 |  | 0,000111398 | 9,2678E-05 | 0,00010451 |  | 0,000102714 |
| slope w/o blank |  | 0,000112007 |  | 0,000107931 | -5,77924E-07 |  | -4,12927E-08 | 0,000100454 | 9,62126E-05 |  | 5,26831E-06 | 1,63055E-06 | 1,88184E-05 |  | 1,50671E-05 | 4,75439E-05 | 4,97472E-05 |  | 0,00011257 | 9,38493E-05 | 0,000105681 |  | 0,000103885 |
|  |  |  |  | 0,000109969 |  |  | -3,09609E-07 |  | 9,83333E-05 |  |  | 3,44943E-06 |  |  | 1,69427E-05 |  | 4,86455E-05 |  |  | 0,000103209 |  |  | 0,000104783 |

0,006598143 -1,85765E-05 0,005899999 0,000206966 0,001016563 0,002918731 0,006192569 0,006286995

0,984797431 -0,002772614 0,8805969 0,030890422 0,151725784 0,435631464 0,924264044 0,938357526

7,034267362 -0,019804384 6,28997786 0,22064587 1,0837556 3,111653316 6,601886027 6,702553755

**min -1** 7,034267362 -0,019804384 6,28997786 0,22064587 1,0837556 3,111653316 6,601886027 6,702553755

|  | **5 min** |  |  |  |  | 30 |  | | | |
| --- | --- | --- | --- | --- | --- | --- | --- | --- | --- | --- |
| **no H2O2** | 7,034267362 | 100 | 100 | 7,034267362 |  | 76,05790021 |  |  |  |  |
| **+ H2O2** | -0,019804384 | -0,281541525 | -0,281541525 | -0,019804384 |  | 4,469147798 |  |  |  |  |
| **+ DTT** | 6,28997786 | 89,41908995 | 89,41908995 | 6,28997786 |  | 97,26724967 |  |  |  |  |
| **+NADPH** | 0,22064587 | 3,136728515 | 3,136728515 | 0,22064587 |  | 31,22145889 |  |  |  |  |
| **NADPH+0.01 μM TrxR1** | 1,0837556 | 15,40680136 | 15,40680136 | 1,0837556 |  |  |  |  |  |  |
| **NADPH+0.1 μM TrxR1** | 3,111653316 | 44,23564183 | 44,23564183 | 3,111653316 |  |  |  |  |  |  |
| **NADPH+1 μM TrxR1** | 6,601886027 | 93,85321437 | 93,85321437 | 6,601886027 |  |  |  |  |  |  |
| **NADPH+2 μM TrxR1** | 6,702553755 | 95,28431903 | 95,28431903 | **6,702553755 + H2O2** | **+ DTT** |  | **+NADPH** | **NADPH+0.01 μM TrxR1** | **NADPH+0.1 μM TrxR1** | **NADPH+1 μM TrxR1** |

Application: Tecan i-control Tecan i-control , 2.0.10.0

Device: infinite 200Pro Serial number: 1307001124 Serial number of connected stacker: Firmware: V_3.37_07/12_Infinite (Jul 20 2012 MAI, V_3.37_07/12_Infinite (Jul 20 2012/13.56.47)

Date: #########

Time: 17:58:44

System MTC-MU063-S

User MTC-MU063-S\TECAN

Plate Greiner 96 Flat Bottom Transparent Polystyrene Cat. No.: 655101/655161/655192 [GRE96ft.pdfx] Plate-ID (Stacker)

List of actions in this measurement script: Kinetic

Absorbance

Label: Label1

Kinetic Measurement

Kinetic duration 00:06:00

Interval Time 00:00:20

Measurement Wavelength 405 nm

Bandwidth 9 nm

Number of Flashes 5

Settle Time 0 ms

Part of Plate D1-D12; E1-E6

Start Time: 2019 12-08 17:58:45

| Cycle Nr. | 1 | 2 | 3 | 4 | 5 | 6 | 7 | 8 | 9 | 10 | 11 | 12 | 13 | 14 | 15 | 16 | 17 | 18 | 19 |
| --- | --- | --- | --- | --- | --- | --- | --- | --- | --- | --- | --- | --- | --- | --- | --- | --- | --- | --- | --- |
| Time [s] | 0 | 20 | 40 | 60 | 80 | 100 | 120 | 140 | 160 | 180 | 200 | 220 | 240 | 260 | 280 | 300 | 320 | 340 | 360 |
| Temp. [°C] | 24,6 | 24,6 | 24,6 | 24,8 | 24,8 | 24,8 | 24,7 | 24,8 | 24,7 | 24,9 | 24,8 | 24,7 | 24,7 | 24,8 | 24,9 | 24,7 | 24,7 | 24,8 | 24,8 |
| D1 | 0,2514 | 0,2525 | 0,2553 | 0,2564 | 0,2598 | 0,2608 | 0,2641 | 0,2643 | 0,2679 | 0,2692 | 0,2734 | 0,2741 | 0,2764 | 0,279 | 0,2818 | 0,2837 | 0,2862 | 0,2872 | 0,2903 |
| D2 | 0,2488 | 0,2487 | 0,2502 | 0,2526 | 0,2538 | 0,2564 | 0,2587 | 0,2611 | 0,2616 | 0,2638 | 0,2675 | 0,2688 | 0,2697 | 0,2744 | 0,2748 | 0,2788 | 0,2804 | 0,2821 | 0,2846 |
| D3 | 0,2406 | 0,2397 | 0,2387 | 0,2373 | 0,2376 | 0,2386 | 0,2387 | 0,2375 | 0,2385 | 0,2381 | 0,2386 | 0,2378 | 0,2373 | 0,2368 | 0,2371 | 0,238 | 0,2385 | 0,2382 | 0,2387 |
| D4 | 0,2388 | 0,2376 | 0,2381 | 0,2376 | 0,2372 | 0,2386 | 0,2382 | 0,2366 | 0,2361 | 0,2363 | 0,2383 | 0,2377 | 0,2369 | 0,2368 | 0,2375 | 0,2361 | 0,2368 | 0,238 | 0,2383 |
| D5 | 0,2437 | 0,2432 | 0,2456 | 0,2466 | 0,2473 | 0,2498 | 0,252 | 0,2536 | 0,2551 | 0,2556 | 0,2594 | 0,2606 | 0,2633 | 0,2653 | 0,2682 | 0,2698 | 0,2725 | 0,2749 | 0,2769 |
| D6 | 0,2446 | 0,2443 | 0,2469 | 0,2487 | 0,2489 | 0,2523 | 0,254 | 0,2559 | 0,2553 | 0,2583 | 0,2622 | 0,2629 | 0,2648 | 0,2663 | 0,2682 | 0,2706 | 0,2726 | 0,2753 | 0,2776 |
| D7 | 0,2437 | 0,2439 | 0,2434 | 0,2425 | 0,2423 | 0,2435 | 0,2427 | 0,2421 | 0,243 | 0,2414 | 0,2439 | 0,2425 | 0,2418 | 0,2446 | 0,2434 | 0,2436 | 0,2438 | 0,2448 | 0,2448 |
| D8 | 0,2463 | 0,2462 | 0,2462 | 0,2439 | 0,2452 | 0,2451 | 0,2455 | 0,2435 | 0,2438 | 0,2444 | 0,2436 | 0,2443 | 0,2449 | 0,2441 | 0,2459 | 0,2449 | 0,2447 | 0,2453 | 0,2468 |
| D9 | 0,2459 | 0,2447 | 0,2463 | 0,2467 | 0,2459 | 0,2473 | 0,2463 | 0,2479 | 0,2478 | 0,2489 | 0,2487 | 0,2494 | 0,2486 | 0,2486 | 0,2496 | 0,2509 | 0,2496 | 0,2512 | 0,2524 |
| D10 | 0,2524 | 0,2521 | 0,2507 | 0,2506 | 0,2504 | 0,2519 | 0,2521 | 0,2523 | 0,2521 | 0,2524 | 0,2519 | 0,251 | 0,2532 | 0,2537 | 0,2537 | 0,2551 | 0,2541 | 0,2552 | 0,2566 |
| D11 | 0,2528 | 0,2523 | 0,2529 | 0,2538 | 0,2534 | 0,2549 | 0,2545 | 0,256 | 0,257 | 0,2568 | 0,2585 | 0,2594 | 0,2616 | 0,261 | 0,2644 | 0,2643 | 0,2658 | 0,266 | 0,2675 |
| D12 | 0,2485 | 0,2501 | 0,2521 | 0,2517 | 0,2515 | 0,254 | 0,2552 | 0,2559 | 0,2557 | 0,2563 | 0,2574 | 0,2585 | 0,2599 | 0,262 | 0,2619 | 0,2631 | 0,2648 | 0,2654 | 0,2675 |
| E1 | 0,2416 | 0,2424 | 0,2457 | 0,2469 | 0,2493 | 0,2511 | 0,255 | 0,2561 | 0,2588 | 0,2595 | 0,2627 | 0,2644 | 0,2664 | 0,2702 | 0,2724 | 0,2744 | 0,2768 | 0,2776 | 0,2804 |
| E2 | 0,2431 | 0,2447 | 0,248 | 0,2493 | 0,2498 | 0,2517 | 0,2533 | 0,2545 | 0,2569 | 0,2593 | 0,2614 | 0,2632 | 0,2651 | 0,2652 | 0,2692 | 0,2712 | 0,2737 | 0,2741 | 0,2763 |
| E3 | 0,2445 | 0,2474 | 0,2497 | 0,2511 | 0,2532 | 0,254 | 0,2565 | 0,2589 | 0,2601 | 0,2629 | 0,265 | 0,2662 | 0,27 | 0,2715 | 0,2724 | 0,2767 | 0,2786 | 0,2818 | 0,2816 |
| E4 | 0,248 | 0,2518 | 0,2533 | 0,2549 | 0,257 | 0,2599 | 0,2623 | 0,2624 | 0,2616 | 0,2652 | 0,2688 | 0,2709 | 0,2737 | 0,2762 | 0,2781 | 0,2803 | 0,2817 | 0,2823 | 0,2872 |
| E5 | 0,2443 | 0,245 | 0,2448 | 0,2452 | 0,2446 | 0,2445 | 0,2431 | 0,2425 | 0,2429 | 0,2428 | 0,2421 | 0,2416 | 0,2437 | 0,2436 | 0,2436 | 0,2427 | 0,2431 | 0,2441 | 0,2441 |
| E6 | 0,2471 | 0,2478 | 0,2488 | 0,2481 | 0,2478 | 0,2481 | 0,2464 | 0,2474 | 0,2468 | 0,2477 | 0,2474 | 0,2481 | 0,2471 | 0,2488 | 0,2474 | 0,2484 | 0,2484 | 0,2487 | 0,2484 |

End Time: 2019 12-08 18:04:55

| **Time (sec) Blank** | **Blank** | **no H2O2** | **no H2O2** | **+ H2O2** |  | **+ H2O2** | **+ DTT** |  | **+ DTT** | **+NADPH** | **+NADPH** |  | **NADPH+0.01 μM TrxR1** | **NADPH+0.01 μM TrxR1** | **NADPH+0.1 μM TrxR1** |  | **NADPH+0.1 μM TrxR1** | **NADPH+1 μM TrxR1** | **NADPH+1 μM TrxR1** |  | **NADPH+2 μM TrxR1** | **NADPH+2 μM TrxR1** |  |
| --- | --- | --- | --- | --- | --- | --- | --- | --- | --- | --- | --- | --- | --- | --- | --- | --- | --- | --- | --- | --- | --- | --- | --- |
| 20 | 0,250800014 0,250699997 | 0,254700005 |  | 0,254799992 | 0,2456 |  | 0,242699996 | 0,243100002 | 0,245900005 |  | 0,243100002 | 0,244900003 | 0,2465 |  | 0,249500006 | 0,252400011 | 0,253899992 |  | 0,243200004 | 0,249300003 | 0,254599988 |  | 0,252900004 |
| 40 | 0,251700014 0,251300007 | 0,256399989 |  | 0,257299989 | 0,245900005 |  | 0,241799995 | 0,244000003 | 0,247999996 |  | 0,242599994 | 0,242899999 | 0,246900007 |  | 0,249899998 | 0,253199995 | 0,253800005 |  | 0,246800005 | 0,249500006 | 0,256399989 |  | 0,255800009 |
| 60 | 0,250499994 0,250099987 | 0,257499993 |  | 0,258700013 | 0,2456 |  | 0,2421 | 0,245299995 | 0,250099987 |  | 0,242400005 | 0,243799999 | 0,247700006 |  | 0,248799995 | 0,253300011 | 0,255299985 |  | 0,248400003 | 0,250200003 | 0,259900004 |  | 0,260199994 |
| 80 | 0,249899998 0,252200007 | 0,260500014 |  | 0,26030001 | 0,244800001 |  | 0,242400005 | 0,247500002 | 0,248799995 |  | 0,243200004 | 0,244000003 | 0,2465 |  | 0,25060001 | 0,256099999 | 0,256700009 |  | 0,250200003 | 0,252799988 | 0,262699991 |  | 0,261099994 |
| 100 | 0,251399994 0,25150001 | 0,262699991 |  | 0,262600005 | 0,244599998 |  | 0,241300002 | 0,250099987 | 0,252799988 |  | 0,242400005 | 0,244000003 | 0,245700002 |  | 0,250999987 | 0,254999995 | 0,255699992 |  | 0,252200007 | 0,253500015 | 0,264899999 |  | 0,265799999 |
| 120 | 0,251700014 0,251899987 | 0,264099985 |  | 0,264400005 | 0,244399995 |  | 0,241799995 | 0,2509 | 0,254799992 |  | 0,241699994 | 0,243499994 | 0,246299997 |  | 0,248400003 | 0,254000008 | 0,256799996 |  | 0,251599997 | 0,255600005 | 0,26699999 |  | 0,267100006 |
| 140 | 0,25029999 0,250699997 | 0,266799986 |  | 0,265799999 | 0,244800001 |  | 0,239800006 | 0,253699988 | 0,255699992 |  | 0,242300004 | 0,242200002 | 0,246600002 |  | 0,25 | 0,25850001 | 0,2579 |  | 0,256199986 | 0,257099986 | 0,268299997 |  | 0,270599991 |
| 160 | 0,251399994 0,252600014 | 0,269899994 |  | 0,268900007 | 0,246099994 |  | 0,241500005 | 0,256199986 | 0,257999986 |  | 0,243200004 | 0,244399995 | 0,247199997 |  | 0,249300003 | 0,259600013 | 0,25819999 |  | 0,25909999 | 0,260100007 | 0,272500008 |  | 0,273600012 |
| 180 | 0,251100004 0,251700014 | 0,272899985 |  | 0,270599991 | 0,245199993 |  | 0,241099998 | 0,257099986 | 0,259499997 |  | 0,2421 | 0,243599996 | 0,247400001 |  | 0,249300003 | 0,25940001 | 0,260100007 |  | 0,260399997 | 0,260800004 | 0,275400013 |  | 0,274199992 |
| 200 | 0,250800014 0,250400007 | 0,27579999 |  | 0,272599995 | 0,245299995 |  | 0,239399999 | 0,258599997 | 0,261500001 |  | 0,243599996 | 0,243200004 | 0,247500002 |  | 0,25029999 | 0,260500014 | 0,261900008 |  | 0,261900008 | 0,263300002 | 0,277099997 |  | 0,278400004 |
| 220 | 0,251199991 0,250999987 | 0,277999997 |  | 0,275000006 | 0,245399997 |  | 0,241999999 | 0,25940001 | 0,263999999 |  | 0,243300006 | 0,243900001 | 0,248099998 |  | 0,251599997 | 0,261999995 | 0,261999995 |  | 0,263200015 | 0,264299989 | 0,280299991 |  | 0,280099988 |
| 240 | 0,251100004 0,249599993 | 0,280299991 |  | 0,277200013 | 0,244599998 |  | 0,240999997 | 0,262699991 | 0,263799995 |  | 0,243100002 | 0,245100006 | 0,247700006 |  | 0,251899987 | 0,262100011 | 0,262699991 |  | 0,265899986 | 0,266400009 | 0,283100009 |  | 0,283300012 |
| 260 | 0,250800014 0,251700014 | 0,281800002 |  | 0,280200005 | 0,245700002 |  | 0,2412 | 0,264699996 | 0,2667 |  | 0,243200004 | 0,242300004 | 0,248199999 |  | 0,252600014 | 0,263799995 | 0,264699996 |  | 0,268099993 | 0,269199997 | 0,285699993 |  | 0,287299991 |
| 280 | 0,249799997 0,250999987 | 0,285299987 |  | 0,281599998 | 0,246000007 |  | 0,241300002 | 0,266299993 | 0,269899994 |  | 0,243699998 | 0,243799999 | 0,248300001 |  | 0,253399998 | 0,264200002 | 0,265700012 |  | 0,270999998 | 0,271400005 | 0,289400011 |  | 0,289400011 |
| 300 | 0,251100004 0,252099991 | 0,286599994 |  | 0,283399999 | 0,2456 |  | 0,241799995 | 0,266799986 | 0,270700008 |  | 0,243699998 | 0,2447 | 0,249200001 |  | 0,252700001 | 0,265799999 | 0,264800012 |  | 0,269600004 | 0,272700012 | 0,292699993 |  | 0,292299986 |
| 320 | 0,250200003 0,25 | 0,290199995 |  | 0,286000013 | 0,246000007 |  | 0,241300002 | 0,270200014 | 0,272599995 |  | 0,244100004 | 0,244499996 | 0,250099987 |  | 0,252700001 | 0,26699999 | 0,265899986 |  | 0,272399992 | 0,272899985 | 0,293500006 |  | 0,295100003 |
| 340 | 0,251199991 0,25 | 0,292800009 |  | 0,289900005 | 0,245299995 |  | 0,241500005 | 0,272199988 | 0,274500012 |  | 0,243900001 | 0,244599998 | 0,249500006 |  | 0,253399998 | 0,26789999 | 0,266099989 |  | 0,275400013 | 0,276499987 | 0,296799988 |  | 0,297899991 |
| 360 | 0,250099987 0,251199991 | 0,295300007 |  | 0,291500002 | 0,246900007 |  | 0,241300002 | 0,274500012 | 0,278299987 |  | 0,243100002 | 0,244399995 | 0,248999998 |  | 0,253399998 | 0,268999994 | 0,268599987 |  | 0,2764 | 0,277399987 | 0,300000012 |  | 0,300799996 |
| slope | -1,42934E-06 -1,59445E-06 | 0,000122312 |  | 0,000107368 | 2,36327E-06 |  | -2,2549E-06 | 9,24303E-05 | 9,18163E-05 |  | 3,87513E-06 | 1,8679E-06 | 9,66975E-06 |  | 1,34675E-05 | 4,98039E-05 | 4,35397E-05 |  | 9,62745E-05 | 8,84726E-05 | 0,000133906 |  | 0,000138478 |
| slope w/o blank |  | 0,000123824 |  | 0,00010888 | 3,87516E-06 |  | -7,43005E-07 | 9,39422E-05 | 9,33282E-05 |  | 5,38702E-06 | 3,37979E-06 | 1,11816E-05 |  | 1,49794E-05 | 5,13158E-05 | 4,50516E-05 |  | 9,77864E-05 | 8,99845E-05 | 0,000135418 |  | 0,00013999 |
|  |  |  |  | 0,000116352 |  |  | 1,56608E-06 |  | 9,36352E-05 |  |  | 4,3834E-06 |  |  | 1,30805E-05 |  | 4,81837E-05 |  |  | 9,38855E-05 |  |  | 0,000137704 |

0,006981117 9,39647E-05 0,005618114 0,000263004 0,000784831 0,002891022 0,005633127 0,008262231

1,041957727 0,014024578 0,838524421 0,039254369 0,117138892 0,431495788 0,840765245 1,233168778

7,44255519 0,100175554 5,98946015 0,28038835 0,836706369 3,082112769 6,005466035 8,808348418

**min -1** 7,44255519 0,100175554 5,98946015 0,28038835 0,836706369 3,082112769 6,005466035 8,808348418

|  | **5 min** |  |  |  |  | 30 |  | | | |
| --- | --- | --- | --- | --- | --- | --- | --- | --- | --- | --- |
| **no H2O2** | 7,44255519 | 100 | 100 | 7,44255519 |  | 76,05790021 |  |  |  |  |
| **+ H2O2** | 0,100175554 | 1,345983353 | 1,345983353 | 0,100175554 |  | 4,469147798 |  |  |  |  |
| **+ DTT** | 5,98946015 | 80,47585805 | 80,47585805 | 5,98946015 |  | 97,26724967 |  |  |  |  |
| **+NADPH** | 0,28038835 | 3,767366759 | 3,767366759 | 0,28038835 |  | 31,22145889 |  |  |  |  |
| **NADPH+0.01 μM TrxR1** | 0,836706369 | 11,24219233 | 11,24219233 | 0,836706369 |  |  |  |  |  |  |
| **NADPH+0.1 μM TrxR1** | 3,082112769 | 41,41202437 | 41,41202437 | 3,082112769 |  |  |  |  |  |  |
| **NADPH+1 μM TrxR1** | 6,005466035 | 80,69091704 | 80,69091704 | 6,005466035 |  |  |  |  |  |  |
| **NADPH+2 μM TrxR1** | 8,808348418 | 118,3511333 | 118,3511333 | **8,808348418 + H2O2** | **+ DTT** |  | **+NADPH** | **NADPH+0.01 μM TrxR1** | **NADPH+0.1 μM TrxR1** | **NADPH+1 μM TrxR1** |

Application: Tecan i-control Tecan i-control , 2.0.10.0

Device: infinite 200Pro Serial number: 1307001124 Serial number of connected stacker: Firmware: V_3.37_07/12_Infinite (Jul 20 2012 MAI, V_3.37_07/12_Infinite (Jul 20 2012/13.56.47)

Date: #########

Time: 18:08:34

System MTC-MU063-S

User MTC-MU063-S\TECAN

Plate Greiner 96 Flat Bottom Transparent Polystyrene Cat. No.: 655101/655161/655192 [GRE96ft.pdfx] Plate-ID (Stacker)

List of actions in this measurement script: Kinetic

Absorbance

Label: Label1

Kinetic Measurement

Kinetic duration 00:06:00

Interval Time 00:00:20

Measurement Wavelength 405 nm

Bandwidth 9 nm

Number of Flashes 5

Settle Time 0 ms

Part of Plate D1-D12; E1-E6

Start Time: 2019 12-08 18:08:36

| Cycle Nr. | 1 | 2 | 3 | 4 | 5 | 6 | 7 | 8 | 9 | 10 | 11 | 12 | 13 | 14 | 15 | 16 | 17 | 18 | 19 |
| --- | --- | --- | --- | --- | --- | --- | --- | --- | --- | --- | --- | --- | --- | --- | --- | --- | --- | --- | --- |
| Time [s] | 0 | 20 | 40 | 60 | 80 | 100 | 120 | 140 | 160 | 180 | 200 | 220 | 240 | 260 | 280 | 300 | 320 | 340 | 360 |
| Temp. [°C] | 24,6 | 24,8 | 24,8 | 24,7 | 24,8 | 24,8 | 24,7 | 24,8 | 24,9 | 24,7 | 24,9 | 24,8 | 24,8 | 24,7 | 24,7 | 24,7 | 24,9 | 24,7 | 24,8 |
| D1 | 0,2519 | 0,2547 | 0,2564 | 0,2575 | 0,2605 | 0,2627 | 0,2641 | 0,2668 | 0,2699 | 0,2729 | 0,2758 | 0,278 | 0,2803 | 0,2818 | 0,2853 | 0,2866 | 0,2902 | 0,2928 | 0,2953 |
| D2 | 0,2541 | 0,2548 | 0,2573 | 0,2587 | 0,2603 | 0,2626 | 0,2644 | 0,2658 | 0,2689 | 0,2706 | 0,2726 | 0,275 | 0,2772 | 0,2802 | 0,2816 | 0,2834 | 0,286 | 0,2899 | 0,2915 |
| D3 | 0,2479 | 0,2456 | 0,2459 | 0,2456 | 0,2448 | 0,2446 | 0,2444 | 0,2448 | 0,2461 | 0,2452 | 0,2453 | 0,2454 | 0,2446 | 0,2457 | 0,246 | 0,2456 | 0,246 | 0,2453 | 0,2469 |
| D4 | 0,2432 | 0,2427 | 0,2418 | 0,2421 | 0,2424 | 0,2413 | 0,2418 | 0,2398 | 0,2415 | 0,2411 | 0,2394 | 0,242 | 0,241 | 0,2412 | 0,2413 | 0,2418 | 0,2413 | 0,2415 | 0,2413 |
| D5 | 0,242 | 0,2431 | 0,244 | 0,2453 | 0,2475 | 0,2501 | 0,2509 | 0,2537 | 0,2562 | 0,2571 | 0,2586 | 0,2594 | 0,2627 | 0,2647 | 0,2663 | 0,2668 | 0,2702 | 0,2722 | 0,2745 |
| D6 | 0,2454 | 0,2459 | 0,248 | 0,2501 | 0,2488 | 0,2528 | 0,2548 | 0,2557 | 0,258 | 0,2595 | 0,2615 | 0,264 | 0,2638 | 0,2667 | 0,2699 | 0,2707 | 0,2726 | 0,2745 | 0,2783 |
| D7 | 0,2438 | 0,2431 | 0,2426 | 0,2424 | 0,2432 | 0,2424 | 0,2417 | 0,2423 | 0,2432 | 0,2421 | 0,2436 | 0,2433 | 0,2431 | 0,2432 | 0,2437 | 0,2437 | 0,2441 | 0,2439 | 0,2431 |
| D8 | 0,2446 | 0,2449 | 0,2429 | 0,2438 | 0,244 | 0,244 | 0,2435 | 0,2422 | 0,2444 | 0,2436 | 0,2432 | 0,2439 | 0,2451 | 0,2423 | 0,2438 | 0,2447 | 0,2445 | 0,2446 | 0,2444 |
| D9 | 0,2458 | 0,2465 | 0,2469 | 0,2477 | 0,2465 | 0,2457 | 0,2463 | 0,2466 | 0,2472 | 0,2474 | 0,2475 | 0,2481 | 0,2477 | 0,2482 | 0,2483 | 0,2492 | 0,2501 | 0,2495 | 0,249 |
| D10 | 0,2496 | 0,2495 | 0,2499 | 0,2488 | 0,2506 | 0,251 | 0,2484 | 0,25 | 0,2493 | 0,2493 | 0,2503 | 0,2516 | 0,2519 | 0,2526 | 0,2534 | 0,2527 | 0,2527 | 0,2534 | 0,2534 |
| D11 | 0,2518 | 0,2524 | 0,2532 | 0,2533 | 0,2561 | 0,255 | 0,254 | 0,2585 | 0,2596 | 0,2594 | 0,2605 | 0,262 | 0,2621 | 0,2638 | 0,2642 | 0,2658 | 0,267 | 0,2679 | 0,269 |
| D12 | 0,2537 | 0,2539 | 0,2538 | 0,2553 | 0,2567 | 0,2557 | 0,2568 | 0,2579 | 0,2582 | 0,2601 | 0,2619 | 0,262 | 0,2627 | 0,2647 | 0,2657 | 0,2648 | 0,2659 | 0,2661 | 0,2686 |
| E1 | 0,2442 | 0,2432 | 0,2468 | 0,2484 | 0,2502 | 0,2522 | 0,2516 | 0,2562 | 0,2591 | 0,2604 | 0,2619 | 0,2632 | 0,2659 | 0,2681 | 0,271 | 0,2696 | 0,2724 | 0,2754 | 0,2764 |
| E2 | 0,2483 | 0,2493 | 0,2495 | 0,2502 | 0,2528 | 0,2535 | 0,2556 | 0,2571 | 0,2601 | 0,2608 | 0,2633 | 0,2643 | 0,2664 | 0,2692 | 0,2714 | 0,2727 | 0,2729 | 0,2765 | 0,2774 |
| E3 | 0,2517 | 0,2546 | 0,2564 | 0,2599 | 0,2627 | 0,2649 | 0,267 | 0,2683 | 0,2725 | 0,2754 | 0,2771 | 0,2803 | 0,2831 | 0,2857 | 0,2894 | 0,2927 | 0,2935 | 0,2968 | 0,3 |
| E4 | 0,2513 | 0,2529 | 0,2558 | 0,2602 | 0,2611 | 0,2658 | 0,2671 | 0,2706 | 0,2736 | 0,2742 | 0,2784 | 0,2801 | 0,2833 | 0,2873 | 0,2894 | 0,2923 | 0,2951 | 0,2979 | 0,3008 |
| E5 | 0,2518 | 0,2508 | 0,2517 | 0,2505 | 0,2499 | 0,2514 | 0,2517 | 0,2503 | 0,2514 | 0,2511 | 0,2508 | 0,2512 | 0,2511 | 0,2508 | 0,2498 | 0,2511 | 0,2502 | 0,2512 | 0,2501 |
| E6 | 0,2522 | 0,2507 | 0,2513 | 0,2501 | 0,2522 | 0,2515 | 0,2519 | 0,2507 | 0,2526 | 0,2517 | 0,2504 | 0,251 | 0,2496 | 0,2517 | 0,251 | 0,2521 | 0,25 | 0,25 | 0,2512 |
