# Supplementary material for: Redox regulation of PTPN22 affects the severity of T-cell-dependent autoimmune inflammation
Source: eLife. 2022 May 19;11:e74549. doi: 10.7554/eLife.74549 (PMC9119677; doi:10.7554/eLife.74549)
Supplement: Figure 5—source data 1. [file elife-74549-fig5-data1.pptx]

## Slide 1
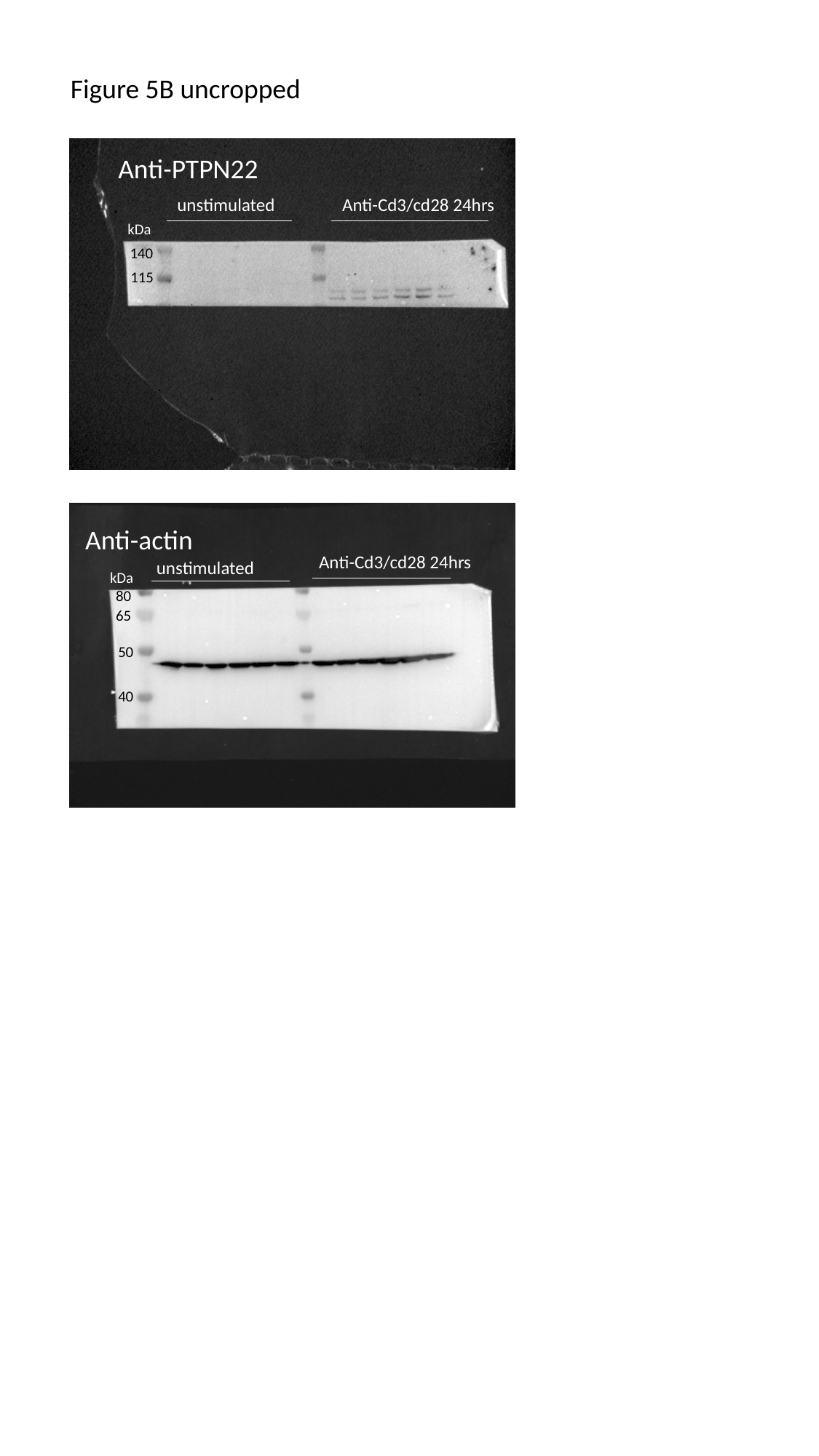

Figure 5B uncropped
Anti-PTPN22
Anti-Cd3/cd28 24hrs
unstimulated
kDa
140
115
Anti-actin
kDa
80
65
50
40
Anti-Cd3/cd28 24hrs
unstimulated
