# Supplementary material for: Redox regulation of PTPN22 affects the severity of T-cell-dependent autoimmune inflammation
Source: eLife. 2022 May 19;11:e74549. doi: 10.7554/eLife.74549 (PMC9119677; doi:10.7554/eLife.74549)
Supplement: Figure 6—source data 1. [file elife-74549-fig6-data1.pptx]

## Slide 1
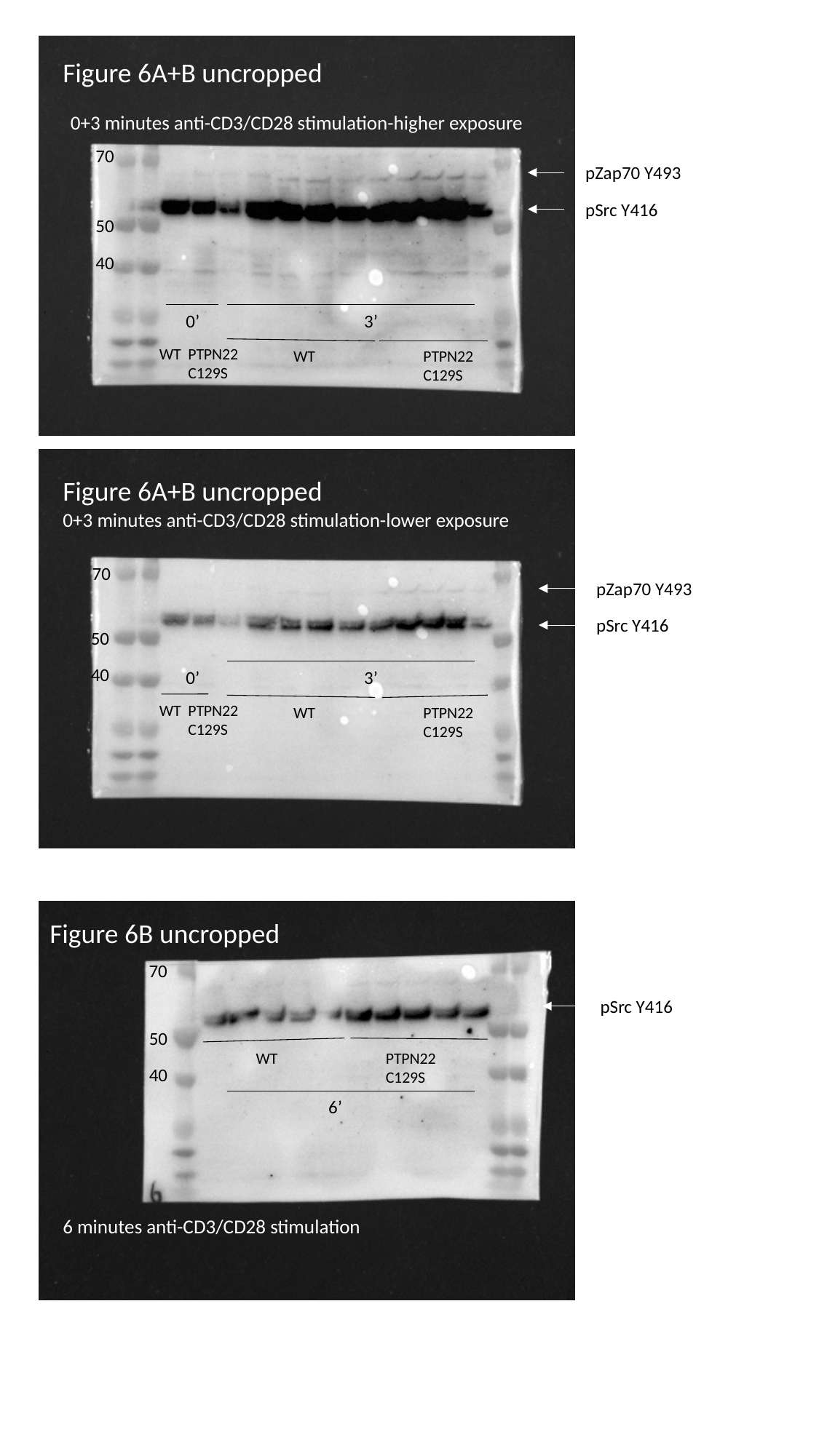

Figure 6A+B uncropped
0+3 minutes anti-CD3/CD28 stimulation-higher exposure
70
50
40
3’
0’
PTPN22 C129S
WT
WT
PTPN22 C129S
pZap70 Y493
pSrc Y416
Figure 6A+B uncropped
0+3 minutes anti-CD3/CD28 stimulation-lower exposure
70
pZap70 Y493
pSrc Y416
50
40
3’
0’
PTPN22 C129S
WT
WT
PTPN22 C129S
Figure 6B uncropped
70
pSrc Y416
50
WT
PTPN22 C129S
40
6’
6 minutes anti-CD3/CD28 stimulation

## Slide 2
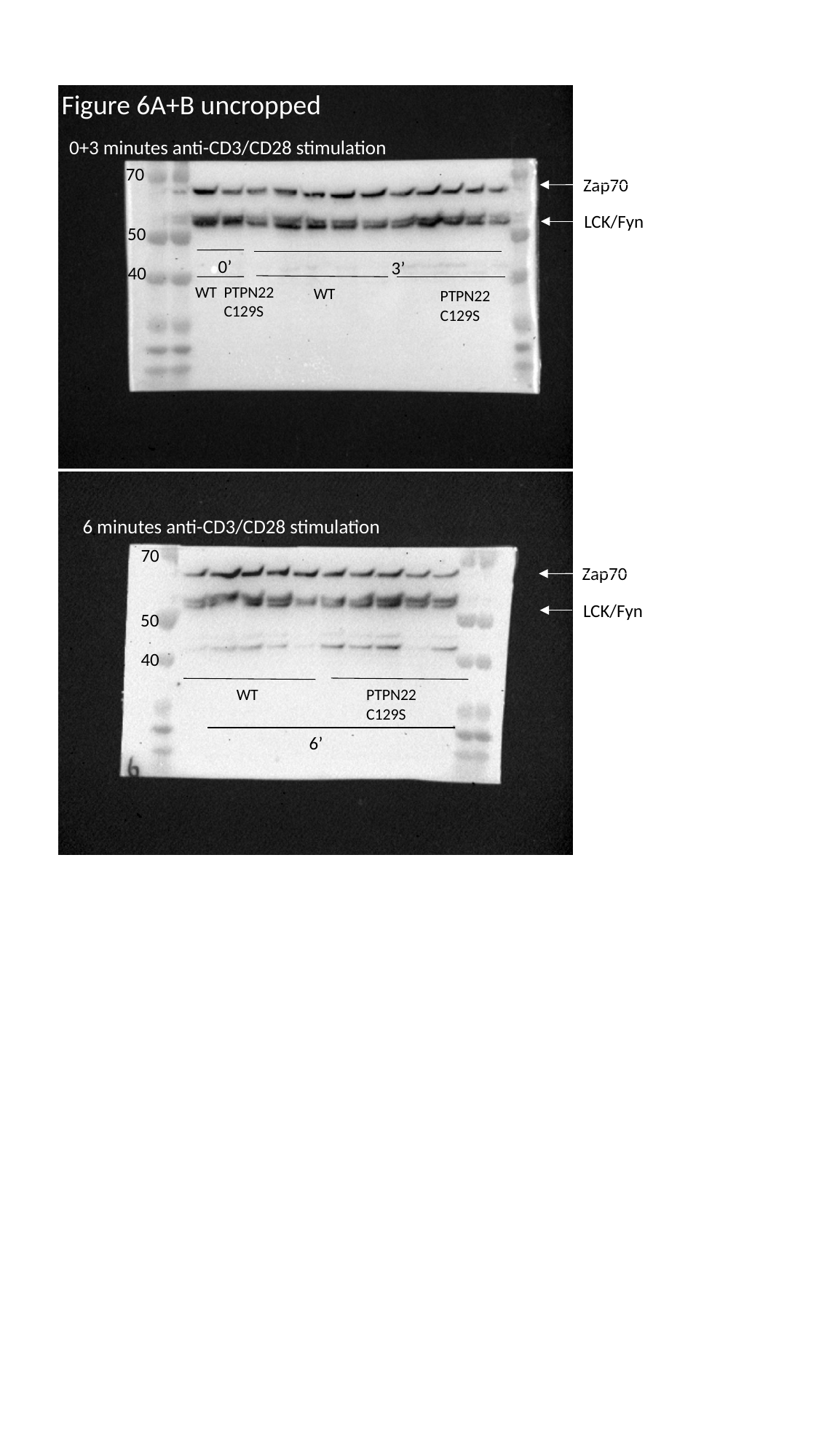

Figure 6A+B uncropped
0+3 minutes anti-CD3/CD28 stimulation
70
Zap70
LCK/Fyn
50
0’
3’
40
PTPN22 C129S
WT
WT
PTPN22
C129S
6 minutes anti-CD3/CD28 stimulation
70
Zap70
LCK/Fyn
50
40
WT
PTPN22
C129S
6’

## Slide 3
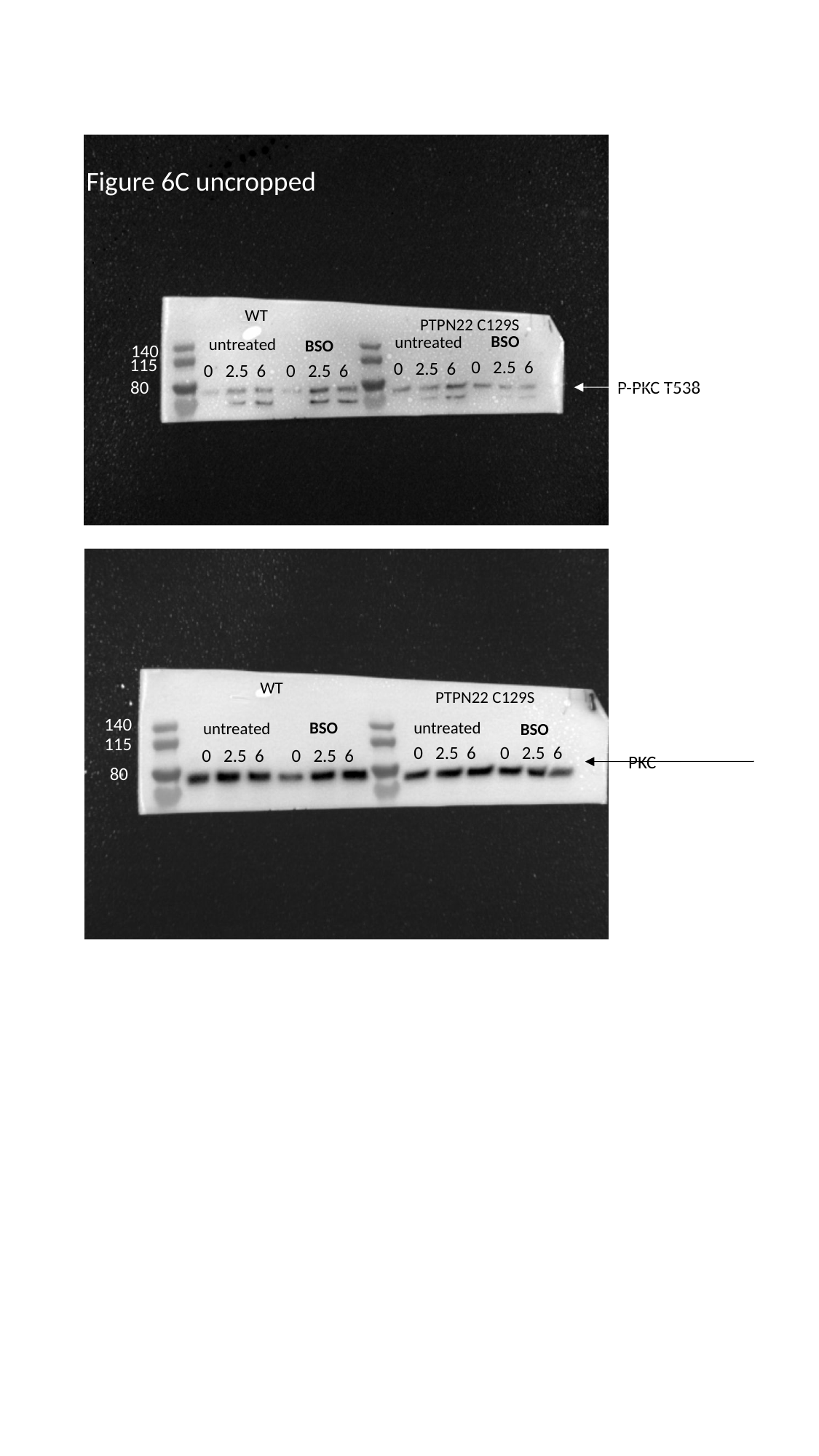

Figure 6C uncropped
WT
PTPN22 C129S
BSO
untreated
untreated
BSO
140
115
0 2.5 6
0 2.5 6
0 2.5 6
0 2.5 6
80
P-PKC T538
WT
PTPN22 C129S
140
BSO
untreated
untreated
BSO
115
0 2.5 6
0 2.5 6
0 2.5 6
0 2.5 6
PKC
80
