# Supplementary material for: Redox regulation of PTPN22 affects the severity of T-cell-dependent autoimmune inflammation
Source: eLife. 2022 May 19;11:e74549. doi: 10.7554/eLife.74549 (PMC9119677; doi:10.7554/eLife.74549)
Supplement: Supplementary file 1. [file elife-74549-supp1.docx]

**Supplemental Table 1: Primer list**

|  | Forward (5'-->3') | Reverse (3'-->5') |
| --- | --- | --- |
| PTPN22 | ACAAGGGGCTGAAGCGGAGAGC | AAAGCGCCGGGGCTGTG |
| Actin | AACCATGAAAAGATGACCCAGAT | GTCCATCACAATGCCTGTGGTA |
| CXCR3 | TACCTTGAGGTTAGTGAACGTCA | CGCTCTCGTTTTCCCCATAATC |
