# Supplementary material for: Redox regulation of PTPN22 affects the severity of T-cell-dependent autoimmune inflammation
Source: eLife. 2022 May 19;11:e74549. doi: 10.7554/eLife.74549 (PMC9119677; doi:10.7554/eLife.74549)
Supplement: Supplementary file 2. [file elife-74549-supp2.docx]

| **Marker** | **Fluorophore** | **Manufacturer** | **Cat.No.** |
| --- | --- | --- | --- |
| CD45 | APC | Biolegend | 103111 |
| CD4 | Qdot605 | BD | 563151 |
| CD4 | Pacific Blue | BD | 558107 |
| TCRb | PercpCy5.5 | BD | 560657 |
| TCRgd | PE-Cy7 | Biolegend | 118123 |
| CD44 | AF700 | Biolegend | 103026 |
| CD44 | FITC | BD | 561859 |
| CD44 | PercpCy5.5 | BD | 560570 |
| CD25 | PE | BD | 553075 |
| CD25 | FITC | Biolegend | 102017 |
| CD62L | PE-Cy7 | Biolegend | 104417 |
| FOXP3 | APC | Invitrogen | 17-5773-82 |
| FOXP3 | PE | Invitrogen | 12-5773-82 |
| CD69 | PE | Biolegend | 104507 |
| CD103 | PE | BD | 557495 |
| IFNγ | APC | BD | 505807 |
| B22a-biotin | Streptavidin-BV421 (Biolegend  405226) | In-house |  |

**Supplemental Table 2: antibody list**
